# PR2ALIGN: a stand-alone software program and a web-server for protein sequence alignment using weighted biochemical properties of amino acids

**DOI:** 10.1186/s13104-015-1152-6

**Published:** 2015-05-07

**Authors:** Igor B Kuznetsov, Michael McDuffie

**Affiliations:** Cancer Research Center and Department of Epidemiology and Biostatistics, University at Albany, State University of New York, One Discovery Drive, Rensselaer, NY 12144 USA

**Keywords:** Amino acid, Protein, Sequence, Physico-chemical attributes, Property profile, Minimal distance global alignment, Dynamic programming

## Abstract

**Background:**

Alignment of amino acid sequences is the main sequence comparison method used in computational molecular biology. The selection of the amino acid substitution matrix best suitable for a given alignment problem is one of the most important decisions the user has to make. In a conventional amino acid substitution matrix all elements are fixed and their values cannot be easily adjusted. Moreover, most existing amino acid substitution matrices account for the average (dis)similarities between amino acid types and do not distinguish the contribution of a specific biochemical property to these (dis)similarities.

**Findings:**

PR2ALIGN is a stand-alone software program and a web-server that provide the functionality for implementing flexible user-specified alignment scoring functions and aligning pairs of amino acid sequences based on the comparison of the profiles of biochemical properties of these sequences. Unlike the conventional sequence alignment methods that use 20x20 fixed amino acid substitution matrices, PR2ALIGN uses a set of weighted biochemical properties of amino acids to measure the distance between pairs of aligned residues and to find an optimal minimal distance global alignment. The user can provide any number of amino acid properties and specify a weight for each property. The higher the weight for a given property, the more this property affects the final alignment. We show that in many cases the approach implemented in PR2ALIGN produces better quality pair-wise alignments than the conventional matrix-based approach.

**Conclusions:**

PR2ALIGN will be helpful for researchers who wish to align amino acid sequences by using flexible user-specified alignment scoring functions based on the biochemical properties of amino acids instead of the amino acid substitution matrix. To the best of the authors’ knowledge, there are no existing stand-alone software programs or web-servers analogous to PR2ALIGN. The software is freely available from http://pr2align.rit.albany.edu.

**Electronic supplementary material:**

The online version of this article (doi:10.1186/s13104-015-1152-6) contains supplementary material, which is available to authorized users.

## Findings

### Background

Alignment of amino acid sequences is the main sequence comparison method used in computational molecular biology. Dynamic programming provides a computationally efficient way of finding an optimal sequence alignment of two amino acid sequences, given an alignment scoring function [1,2]. This optimal alignment found by dynamic programming depends on the choice of the alignment scoring function, which typically consists of an amino acid substitution matrix used to account for matches/mismatches and gap penalties used to account for insertions/deletions [3,4]. After the advent of sequence alignment algorithms that use dynamic programming and substitution matrix-based scoring functions, several novel alignment algorithms that use more sophisticated scoring functions based on Hidden Markov Models (HMMs) have been developed [5-10]. However, global pair-wise alignment with dynamic programming and substitution matrices is still extensively used in sequence analysis, including such fundamental applications as homology modeling [11] and multiple sequence alignment algorithms [12,13].

Most amino acid substitution matrices are based on the same basic assumption: if two given amino acid types are frequently observed in the equivalent positions in related proteins, they have similar biochemical properties and *vice versa* [14-21]. The individual elements of a substitution matrix are obtained by averaging the amino acid frequencies over all sequence positions in a large collection of protein sequences. As a result of such averaging, most existing amino acid substitution matrices are general-purpose matrices that account for the average (dis)similarities between amino acid types and do not distinguish the contribution of a specific biochemical property to these (dis)similarities. The selection of the amino acid substitution matrix best suitable for a given alignment problem is one of the most important decisions the user has to make [22], because all matrix elements are fixed and their values cannot be easily adjusted. This lack of flexibility in the conventional substitution matrix-based sequence alignment may limit the options of a user who wishes to align and compare amino acid sequences by applying a user-specified scoring function based on the biochemical properties of amino acids, such as hydrophobicity, size, charge, etc. The biochemical properties of the amino acids have been extensively used to construct global and local descriptors of protein sequences in various applications for alignment-free comparison and classification of proteins, including prediction of DNA-binding proteins, sub-cellular localization, and protein disorder [23-26]. Hundreds of numerical indices that represent various properties of the amino acids are available from the AAindex database [27]. However, applications that use biochemical properties of the amino acids for alignment-based comparison of protein sequences are lacking. ProtScale [28] is an on-line tool that allows the user to construct a graphical plot that displays the profile of some user-selected biochemical property of the amino acids, such as the hydrophobicity profile, for the input protein sequence. The user can construct plots that show profiles for two protein sequences and perform a qualitative visual comparison of these profiles. However, ProtScale does not provide capabilities for quantitative alignment-based comparison of two protein sequences based on the profiles of biochemical properties.

PR2ALIGN is a stand-alone software program and a web-server that provide the functionality for implementing flexible user-specified alignment scoring functions and aligning pairs of amino acid sequences based on the comparison of the profiles of biochemical properties of these sequences. Unlike the conventional sequence alignment methods that use 20×20 fixed amino acid substitution matrices, PR2ALIGN uses a flexible set of weighted biochemical properties of amino acids to measure the distance between pairs of aligned residues and to find an optimal minimal distance global alignment. The user can provide any number of amino acid properties and specify a weight for each property. The higher the weight for a given property, the more this property affects the final alignment. To the best of the authors’ knowledge, there is no existing stand-alone or on-line software analogous to PR2ALIGN.

### Algorithm

Let *X* = {*x*_*1*_*,…,x*_*n*_} and *Y* = {*y*_*1*_*,…,y*_*m*_} be two amino acid sequences of length *n* and *m* residues, respectively. PR2ALIGN uses the following function to calculate the score for a global alignment between *X* and *Y*:

1 $$ \mathrm{Score}\left(\mathrm{X},\mathrm{Y}\right) = {\displaystyle \sum_{\mathrm{all}\ \mathrm{aligned}\ \mathrm{pairs}\ \left(i,j\right)\ }\mathrm{d}\left({\mathrm{x}}_{\mathrm{i}},{\mathrm{y}}_{\mathrm{j}}\right)+{\displaystyle \sum_{L\in all\kern0.5em  gaps}g\left({r}_L\right)}} $$

Where *d*(*x*_*i*_*,y*_*j*_) is the distance between aligned pair of characters *x*_*i*_ and *y*_*j*_ (the *i*-th character in sequence *X* and the *j*-th character in sequence *Y*), and *g(r*_*L*_*)* is the affine gap penalty for the *L*^th^ gap:

2 $$ g\left({r}_L\right)=\alpha +\left({r}_L-1\right)*\beta $$

Where *α* is the gap opening penalty, *β* is the gap extension penalty, and *r*_*L*_ is the length of the *L*^th^ gap (*α* ≥ 0, *β* ≥ 0, *r*_*L*_ ≥ 1).

Biochemical properties of each of the 20 amino acid types are represented by a numerical property vector, *P*, of dimension *k* (where *k* is the number of amino acid properties used for the alignment, *k* ≥ 1). A residue *x*_*i*_ in sequence position *i* is represented by a *k*–dimensional biochemical property vector:

3 $$ P\left({x}_i\right) = \left\{{p}_1\left({x}_i\right),\dots, {p}_k\left({x}_i\right)\right\} $$

The total distance between a pair of amino acid residues, *d*(*x*_*i*_*,y*_*j*_), is computed according to the following equation:

4 $$ d\left({x}_i,{y}_j\right)={\displaystyle \sum_{b=1}^k\left|{p}_b\left({x}_i\right)-{p}_b\left({y}_j\right)\right|}\cdot w(b) $$

$$ {\displaystyle \sum_{b=1}^kw(b)=1.0} $$

Where *w*(*b*) is the weight assigned to the amino acid property *b*.

The optimal minimal distance global alignment score, *D*_*n,m*_, for sequences *X* and *Y* is found by applying the dynamic programming recursion [2]:

5 $$ {D}_{i,j}= \min \left\{\begin{array}{l}{D}_{i-1,j-1}+d\left({x}_i,{y}_j\right),\\ {}{ \min}_{1\le t\le j}\left\{{D}_{i,j-t}+g(t)\right\},\\ {}{ \min}_{1\le r\le i}\left\{{D}_{i-r,j}+g(r)\right\}\end{array}\right\},\kern0.5em 1\le i\le n,\kern0.5em 1\le j\le m $$

Where *D*_*0,0*_ *=* 0*, D*_*i,0*_ *= g(i), D*_*0,j*_ *= g(j)*.

The alignment corresponding to the optimal score is found by tracing back from *D*_*n,m*_ to *D*_*0,0*_.

By default, each biochemical property is normalized in such a way that all its values are in range [0, 1] according to the following equation:

6 $$ n{p}_i\left({x}_j\right)=\frac{p_i\left({x}_j\right)-{ \min}_i}{{ \max}_i-{ \min}_i} $$

Where *p*_*i*_*(x*_*j*_*)* is the value of biochemical property *i* for amino acid *x*_*j*_; min_*i*_ and max_*i*_ are the minimum and maximum values of the *i*-th biochemical property. The user may choose to disable normalization and use the raw biochemical properties (not recommended).

### Default amino acid properties, property weights, and gap penalties

By default, PR2ALIGN uses the following four amino acid properties: hydrophobicity [29], size [30], coil propensity [31], and the presence of thiol group. These four properties were selected for the following two reasons. First, the total number of properties was limited to four due to the computational complexity of the process of optimization of property weights and gap penalties (described below). This process involves a grid search that has computational complexity n*N^(k+2)^, where *n* is the number of sequence pairs in the benchmark dataset, *N* is the number of grid points, *k* is the number of amino acid properties, and the factor of 2 accounts for gap initiation and gap extension penalties. For instance, using 6,000 sequence pairs, a coarse 20-point grid, and 4 amino acid properties involves performing 6*10^3^*20^4+2^ ≈ 3.8*10^11^ pair-wise alignments. For more than 4 properties the procedure becomes computationally too expensive. Second, it was shown that the three main factors that explain a significant proportion of the total variability in amino acid properties are related to hydrophobicity, size, and structural propensity [32]. Coil propensity was selected because it is correlated with other structural propensities and helps to distinguish such structurally important amino acids as glycine and proline [33]. The presence of the thiol group was selected because it is a unique property of the amino acid cysteine, which tends to be highly conservative in homologous proteins and plays a special role in sequence alignment [34].

The optimized property weights and gap penalties for the four default properties are listed in Table 1.

**Table 1:** **The optimized property weights and gap penalties for the four default amino acid properties**

**Pair-wise sequence identity**	**Weight for hydrophobicity**	**Weight for size**	**Weight for coil propensity**	**Weight for thiol group**	**Gap initiation penalty**	**Gap extension penalty**	**N pairs**
0-10%	0.7	0.15	0.1	0.05	0.8	0.2	1,282
10-20%	0.3	0.2	0.15	0.35	0.6	0.1	2,023
20-30%	0.3	0.2	0.15	0.35	0.7	0.1	1,674
**30-40%**	**0.25**	**0.2**	**0.15**	**0.4**	**0.7**	**0.1**	**1,100**
Above 40%	0.2	0.2	0.25	0.35	0.6	0.1	705

The four default amino acid properties are hydrophobicity [29], size [30], coil propensity [31] and the presence of the thiol group. By default, PR2ALIGN uses the combination of property weights and gap penalties optimized for aligning sequences with pair-wise sequence identity between 30 and 40 percent (highlighted in boldface type).the column “**N** pairs” shows the total number of sequence pairs in each benchmark dataset.

These property weights and gap penalties have been optimized on the SABmark database of benchmark sequence alignments [35] using our previously described approach [22]. In this approach, a grid search is used to find an optimal combination of the property weights and gap penalties that produces pair-wise sequence alignments most similar to the reference structure-based pair-wise alignments of homologous proteins from the Superfamily (SUP) sub-set of SABmark. Such an optimal combination is defined as the one that maximizes the average alignment accuracy score calculated over all pairs of sequences in the benchmark dataset, *Q*_*AVER*_:

7 $$ {Q}_{AVER}=\frac{{\displaystyle \sum_{\left(i,j\right)}Q\left(i,j\right)}}{N_{pairs}} $$

Where *N*_pairs_ is the total number of SABmark sequence pairs in the benchmark dataset and *Q(i,j)* is the accuracy of the test alignment between sequences *i* and *j. Q(i,j)* is calculated by comparing the test alignment to the reference SABmark alignment for the same pair of sequences (*i, j*) [36]:

8 $$ Q\left(i,j\right)=\frac{f_D\left(i,j\right)+{f}_M\left(i,j\right)}{2} $$

9 $$ {f}_D\left(i,j\right)=\frac{n_I\left(i,j\right)}{l_R\left(i,j\right)}*100\% $$

10 $$ {f}_M\left(i,j\right)=\frac{n_I\left(i,j\right)}{l_T\left(i,j\right)}*100\% $$

Where *n*_*I*_*(i,j)* is the number of residue pairs aligned identically in the test and the reference alignments; *l*_*R*_*(i,j)* is the length of the reference alignment; *l*_*T*_*(i,j)* is the length of the test alignment.

In order to minimize the effect of over-represented protein families and to reduce the computational time required for the grid search, if some SABmark super-family had more than 10 sequence pairs, only 10 pairs were randomly selected from this super-family. For property weights, a 20-point grid with 0.05 increments was used. For gap penalties, a 10-point grid with 0.1 increments was used.

### Comparison of PR2ALIGN and matrix-based alignment

We compared the quality of sequence alignments produced by the PR2ALIGN algorithm to the quality of sequence alignments produced by the matrix-based global alignment algorithm [2] performed with the VTML200 amino acid substitution matrix [19] and the affine gap penalty function. VTML200 was selected because it is a state-of-the-art matrix that has been shown to outperform other amino acid substitution matrices [19,22]. PR2ALIGN was used with the four default amino acid properties and property weights and gap penalties optimized for each of the five ranges of pair-wise sequence identity listed in Table 1 using the benchmark dataset and grid search procedure described in the previous section. VTML200 was used with gap penalties (Table 2) optimized for the same five ranges of pair-wise sequence identity using the same benchmark dataset and a 50-point integer grid with the increments of 1. All sequence pairs used in the optimization procedure are listed in Additional files 1, 2, 3, 4 and 5.

**Table 2:** **The optimized gap penalties for the VTML200 matrix**

**Pair-wise sequence identity**	**Gap initiation penalty**	**Gap extension penalty**	**N pairs**
0-10%	−17	−1	1,282
10-20%	−17	−1	2,023
20-30%	−16	−1	1,674
30-40%	−16	−1	1,100
Above 40%	−15	−1	705

The column “**N pairs**” shows the total number of sequence pairs in each benchmark dataset.

**Figure 1:**
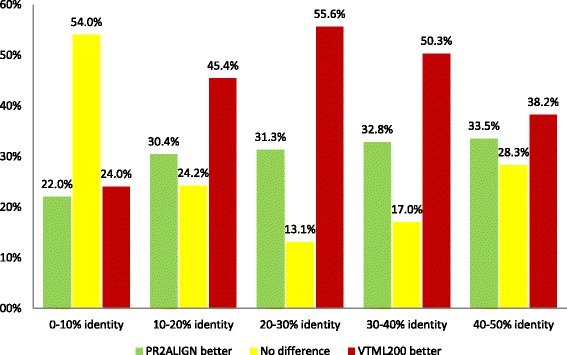
Comparison of the PR2ALIGN alignments with the alignments obtained using the VTML200 substitution matrix. Results are reported for five ranges of the percentage of pair-wise sequence identity. Green bars show the percentage of sequence pairs on which PR2ALIGN performs better than VTML200. Yellow bars show the percentage of sequence pairs on which PR2ALIGN and VTML200 show identical performance. Red bars show the percentage of sequence pairs on which VTML200 performs better than PR2ALIGN. The comparison was performed using the entire super-family (SUP) subset of the SABmark database [35]: 3,761 sequence pairs for the 0-10% range dataset, 8,521 for 10-20%, 4,181 for 20-30%, 1,952 for 30-40%, and 884 for 40-50%.

The comparison of the two alignment methods was performed using the following procedure. First, PR2ALIGN is used to align a sequence pair (*i,j*) from the benchmark dataset and the first alignment accuracy score *Q*_1_(*i,j*) is calculated using Eq.8. Second, the matrix-based alignment with VTML200 is used to align the same sequence pair and the second alignment accuracy score *Q*_2_(*i,j*) is calculated. Then, the difference between these two scores is calculated, *D*(*i,j*) = *Q*_1_(*i,j*) – *Q*_2_(*i,j*). If the difference is positive, it means that PR2ALIGN performed better than VTML200 on the pair (*i,j*). If the difference is zero, it means that PR2ALIGN and VTML200 showed identical performance. If the difference is negative, it means that VTML200 performed better than PR2ALIGN. All sequence pairs used for the comparison are listed in Additional files 6, 7, 8, 9 and 10. The results of this comparison for each of the five ranges of sequence identity are shown in Figure 1. Depending on the range of sequence identity, PR2ALIGN outperforms VTML200 on 22.0% to 33.5% of the sequence pairs. Performance is identical for 13.1% to 54% of the sequence pairs. VTML200 outperforms PR2ALIGN on 24.0% to 55.6% of the sequence pairs. The main conclusion from this comparison is that even with the four default amino acid properties, which account only for a fraction of the total variability among the amino acid types, PR2ALIGN outperforms the best amino acid substitution matrix on a considerable percentage of the test cases. Additional file 11: Figures S1-S5 show five specific examples of PR2ALIGN and VTML200 alignments (one alignment for each of the five ranges of sequence identity). In these examples, PR2ALIGN correctly aligns most structurally equivalent positions, whereas alignment with VTML200 either completely or nearly completely fails.

### Stand-alone software program

The stand-alone alignment program is written in C++. The source code and pre-compiled Windows and Linux executables are freely available under a GNU General Public License (http://www.gnu.org/licenses/) from http://pr2align.rit.albany.edu/download.html. The program reads amino acid sequences in the FASTA format (see “Example of FASTA file” below). The user has options to save the alignment as an HTML-formatted file or as a FASTA-formatted text file. The compilation instructions and command line options are described in the README file included in the distribution.

### Example of FASTA file

FASTA file consists of a header line that begins with ">" character, followed by an optional sequence name and the sequence itself:

>Sequence name goes here…

MARLLTTCCLLALLLAACTDVALSKKGKGKPSGGGWGAGSHRQPSYPRQPGYPHNPGYPHNPGYPHNPGYPHNPGYPHNPGYPQNPGYPHNPGYPGWGQGYNPSSGGSYHNQKPWKPPKTNFKHVAGAAAAGAVVGGLGGYAMGRVMSGMNYHFDSPDEYRWWSENSARYPNRVYYRDYSSPVPQDVFVADCFNITVTEYSIGPAAKKNTSEAVAAANQTEVEMENKVVTKVIREMCVQQYREYRLASGIQLHPADTWLAVLLLLLT

### Web-server implementation

**Figure 2:**
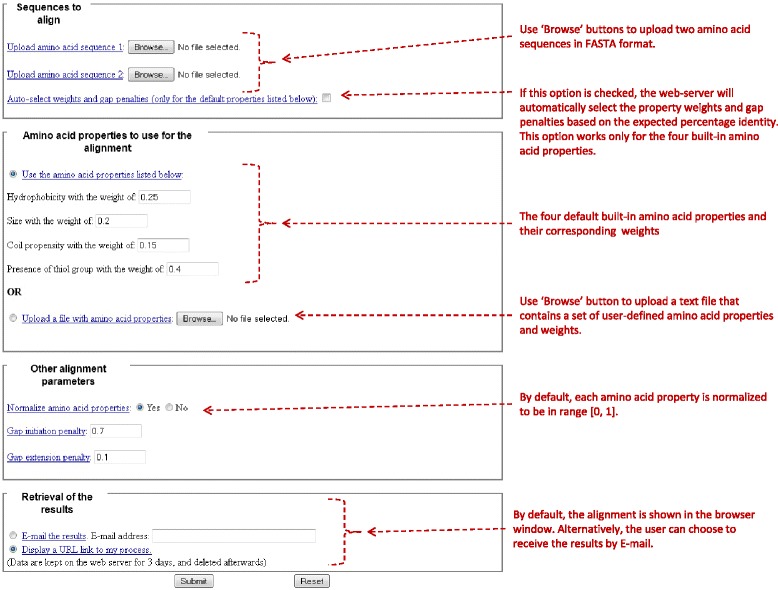
The input page of the web-server implementation of PR2ALIGN.

The alignment program was also implemented as a freely available web-server (http://pr2align.rit.albany.edu). The web-server has a simple user interface (Figure 2) that consists of the four input fields described below. Instructions for each field and general information about the method and the output format can be found in the help pages.

1. **“Sequences to align” –** the user should provide two amino acid sequences in FASTA format. In this input field the user can also choose the option to automatically select property weights and gap penalties. If this option is checked, the web-server will attempt to estimate the expected percentage of sequence identity and will select the property weights and gap penalties based on this expected percentage identity. This option works only for the four default amino acid properties. The expected percentage of sequence identity is estimated by aligning the input sequences using the conventional global sequence alignment with the VTML200 amino acid similarity matrix [19] and gap initiation penalty of −15 and gap extension penalty of −1 (gap penalties for this matrix optimized on the benchmark dataset that consists of the entire SUP sub-set of SABmark [35]). The user should be aware that this option provides just a rough estimate which may differ from the final percentage sequence identity displayed in PR2ALIGN output.
2. **“Amino acid properties to use for the alignment”** – the user can either use the four default amino acid properties and weights listed in Table 1 or upload a text file that contains any set of user-defined properties and weights. The file format is described in the “Help” pages of the web-server and is shown below in “Example of file with amino acid properties and weights”.

### Example of file with amino acid properties and weights

An example of the file with amino acid properties and weights that can be uploaded to PR2ALIGN web-server. The file must begin with a header line. The second line must contain 20 comma-delimited standard amino acid letters in the specified order. For each property, the file must contain a line that begins with "#PROPERTY" followed by the property name. The line after the property name must contain 20 comma-delimited numbers quantifying this property for each individual amino acid. These numbers must be in the same order as the 20 amino acid letters listed in line 2. For instance, in this example the first hydrophobicity number of 0.25 corresponds to A, the second hydrophobicity number of -1.76 corresponds to R, etc. The line that begins with "W:"' after each property gives the weight assigned to this property. For instance, in this example "Hydrophobicity" has the weight of 0.6 and "Size" has the weight of 0.4:

Header line goes here…

A,R,N,D,C,Q,E,G,H,I,L,K,M,F,P,S,T,W,Y,V

#PROPERTY Hydrophobicity

0.25,-1.76,-0.6,-0.7,0.1,-0.7,-0.6,0.2,-0.4,0.7,0.5,-1.1,0.3,0.6,-0.1,-0.3,-0.2,0.4,0.1,0.5

W:0.6

#PROPERTY Size

28,105,59,40,45,81,62,0,79,94,94,100,94,112,42,23,51,146,117,72

W:0.4

3. **“Other alignment parameters”** – the user can enter the gap initiation and gap extension penalties and choose whether or not to normalize the amino acid properties. By default, properties are normalized to be in range [0, 1] according to Eq.6.
4. **“Retrieval of the results”** – By default, the alignment is displayed in the web-browser window. Alternatively, the user can choose to receive the results by E-mail.

**Figure 3:**
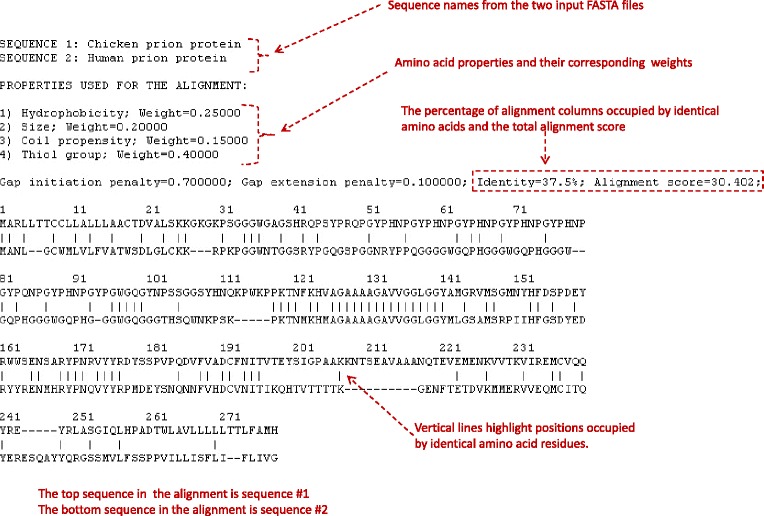
An example of the PR2ALIGN output for the human prion protein and chicken prion protein.

An example of PR2ALIGN output is shown in Figure 3.

## Availability and requirements

**Project name:** PR2ALIGN

**Project home page:**http://pr2align.rit.albany.edu

**Operating system(s):** Platform independent

**Programming language:** C++ (stand-alone program), JavaScript and Perl (web-server)

**Other requirements:** None

**License:** GNU GPL

**Any restrictions to use by non-academics:** license needed

## Additional files

Additional file 1:
**SABmark SUP sequence pairs for 0-10% sequence identity range used to optimize property weights and gap penalties.** Maximum of 10 randomly sampled pairs per superfamily. Additional file 2:
**SABmark SUP sequence pairs for 10-20% sequence identity range used to optimize property weights and gap penalties.** Maximum of 10 randomly sampled pairs per superfamily. Additional file 3:
**SABmark SUP sequence pairs for 20-30% sequence identity range used to optimize property weights and gap penalties.** Maximum of 10 randomly sampled pairs per superfamily. Additional file 4:
**SABmark SUP sequence pairs for 30-40% sequence identity range used to optimize property weights and gap penalties.** Maximum of 10 randomly sampled pairs per superfamily. Additional file 5:
**SABmark SUP sequence pairs for 40-50% sequence identity range used to optimize property weights and gap penalties.** Maximum of 10 randomly sampled pairs per superfamily. Additional file 6:
**All SABmark SUP sequence pairs for 0-10% sequence identity range.**
Additional file 7:
**All SABmark SUP sequence pairs for 10-20% sequence identity range.**
Additional file 8:
**All SABmark SUP sequence pairs for 20-30% sequence identity range.**
Additional file 9:
**All SABmark SUP sequence pairs for 30-40% sequence identity range.**
Additional file 10:
**All SABmark SUP sequence pairs for 40-50% sequence identity range.**
Additional file 11:
**Supplementary Figures S1-S5.**
