# Supplementary material for: PR2ALIGN: a stand-alone software program and a web-server for protein sequence alignment using weighted biochemical properties of amino acids
Source: BMC Res Notes. 2015 May 7;8:187. doi: 10.1186/s13104-015-1152-6 (PMC4477417; doi:10.1186/s13104-015-1152-6)
Supplement: Additional file 2: — SABmark SUP sequence pairs for 10-20% sequence identity range used to optimize property weights and gap penalties. Maximum of 10 randomly sampled pairs per superfamily. [file 13104_2015_1152_MOESM2_ESM.docx]

SABmark SUP sequence pairs for 10-20% sequence identity range used to optimize property weights and gap penalties.

Maximum of 10 randomly sampled pairs per superfamily.

./group1/reference/d1a6m__-d1itha_.fasta

./group1/reference/d1it2a_-d3sdha_.fasta

./group1/reference/d1a6m__-d1cqxa1.fasta

./group1/reference/d1ash__-d1itha_.fasta

./group1/reference/d1ew6a_-d1mba__.fasta

./group1/reference/d1gcva_-d1itha_.fasta

./group1/reference/d1cg5b_-d1mba__.fasta

./group1/reference/d1ash__-d1cg5b_.fasta

./group1/reference/d1ash__-d2lhb__.fasta

./group1/reference/d1gvha1-d1irda_.fasta

./group2/reference/d1gtea1-d1kf6b1.fasta

./group2/reference/d1gtea1-d1nekb1.fasta

./group3/reference/d1fafa_-d1fpoa1.fasta

./group3/reference/d1fafa_-d1gh6a_.fasta

./group3/reference/d1fafa_-d1hdj__.fasta

./group4/reference/d1eiya1-d1ivsa1.fasta

./group4/reference/d1eiya1-d1lrza1.fasta

./group4/reference/d1lrza1-d1seta1.fasta

./group6/reference/d1h32a1-d1iqca2.fasta

./group6/reference/d1e29a_-d1kv9a1.fasta

./group6/reference/d1kv9a1-d1qn2a_.fasta

./group6/reference/d1h32a1-d1iqca1.fasta

./group6/reference/d1fcdc2-d1h32a1.fasta

./group6/reference/d1fcdc2-d1qn2a_.fasta

./group6/reference/d1h32a1-d1qn2a_.fasta

./group6/reference/d1eb7a1-d1gu2a_.fasta

./group6/reference/d1gu2a_-d1kb0a1.fasta

./group6/reference/d1iqca2-d1ql3a_.fasta

./group7/reference/d1d5ya2-d1jt6a1.fasta

./group7/reference/d1b72b_-d1bl0a2.fasta

./group7/reference/d1fjla_-d1hlva1.fasta

./group7/reference/d1e3oc1-d1hlva1.fasta

./group7/reference/d1k78a2-d2ezi__.fasta

./group7/reference/d1bl0a2-d1g2ha_.fasta

./group7/reference/d1bl0a2-d1bw5__.fasta

./group7/reference/d1irza_-d1k61a_.fasta

./group7/reference/d1iufa1-d1k61a_.fasta

./group7/reference/d1bw5__-d1fexa_.fasta

./group10/reference/d1p4xa1-d1smta_.fasta

./group10/reference/d1hkqa_-d1smta_.fasta

./group10/reference/d1ka8a_-d1smta_.fasta

./group10/reference/d1bm9a_-d2irfg_.fasta

./group10/reference/d1hks__-d1omia1.fasta

./group10/reference/d1hsja1-d1smta_.fasta

./group10/reference/d1e17a_-d1p4xa1.fasta

./group10/reference/d1bjaa_-d1e17a_.fasta

./group10/reference/d1ldja1-d1o7fa1.fasta

./group10/reference/d1ldja1-d1p4xa1.fasta

./group11/reference/d1a04a1-d1gxqa_.fasta

./group11/reference/d1a04a1-d1opc__.fasta

./group11/reference/d1fc3a_-d1fsea_.fasta

./group11/reference/d1fc3a_-d1gxqa_.fasta

./group11/reference/d1fsea_-d1gxqa_.fasta

./group11/reference/d1fsea_-d1opc__.fasta

./group11/reference/d1gxqa_-d1p4wa_.fasta

./group11/reference/d1opc__-d1p4wa_.fasta

./group12/reference/d1efub3-d1f4ia_.fasta

./group12/reference/d1efub3-d1ifya_.fasta

./group12/reference/d1efub3-d1oaia_.fasta

./group12/reference/d1efub3-d1otra_.fasta

./group12/reference/d1f4ia_-d1otra_.fasta

./group12/reference/d1ifya_-d1otra_.fasta

./group12/reference/d1oaia_-d1otra_.fasta

./group14/reference/d1j9ia_-d1tns__.fasta

./group14/reference/d1g4da_-d1lx8a_.fasta

./group14/reference/d1j9ia_-d1lx8a_.fasta

./group14/reference/d1g4da_-d1nd9a_.fasta

./group14/reference/d1g4da_-d1j9ia_.fasta

./group14/reference/d1nd9a_-d1tns__.fasta

./group14/reference/d1exja1-d1g4da_.fasta

./group14/reference/d1d4ua1-d1g4da_.fasta

./group14/reference/d1jjcb2-d1tns__.fasta

./group14/reference/d1lx8a_-d1nd9a_.fasta

./group15/reference/d1quua1-d2spca_.fasta

./group15/reference/d1hcia1-d1quua1.fasta

./group15/reference/d1cuna1-d1hcia1.fasta

./group15/reference/d1cuna2-d1quua2.fasta

./group15/reference/d1cuna2-d1hcia4.fasta

./group15/reference/d1hcia1-d1quua2.fasta

./group15/reference/d1cuna1-d1hcia4.fasta

./group15/reference/d1hcia1-d1hcia4.fasta

./group15/reference/d1hcia4-d1quua1.fasta

./group15/reference/d1cuna2-d1hcia1.fasta

./group16/reference/d1jnra1-d1qlaa1.fasta

./group17/reference/d1deeg_-d1gab__.fasta

./group18/reference/d1erd__-d1hd6a_.fasta

./group18/reference/d1hd6a_-d2erl__.fasta

./group20/reference/d1a32__-d1fyja_.fasta

./group21/reference/d1ckta_-d1i11a_.fasta

./group21/reference/d1ckta_-d2lefa_.fasta

./group21/reference/d1i11a_-d1k99a_.fasta

./group21/reference/d1k99a_-d1qrva_.fasta

./group22/reference/d1kx5b_-d1n1ja_.fasta

./group22/reference/d1bh9b_-d1kx5a_.fasta

./group22/reference/d1jfib_-d1kx5d_.fasta

./group22/reference/d1kx5a_-d1n1jb_.fasta

./group22/reference/d1bh9a_-d1kx5c_.fasta

./group22/reference/d1bh9b_-d1n1ja_.fasta

./group22/reference/d1kx5a_-d1n1ja_.fasta

./group22/reference/d1jfib_-d1kx5b_.fasta

./group22/reference/d1bh9a_-d1kx5d_.fasta

./group22/reference/d1bh9a_-d1kx5a_.fasta

./group23/reference/d1jafa_-d1mqva_.fasta

./group23/reference/d1jafa_-d256ba_.fasta

./group23/reference/d256ba_-d2ccya_.fasta

./group25/reference/d1is2a1-d3mdda1.fasta

./group26/reference/d1jkva_-d1nfva_.fasta

./group26/reference/d1jkva_-d1lkoa1.fasta

./group26/reference/d1jkva_-d1qgha_.fasta

./group26/reference/d1jiga_-d1nfva_.fasta

./group26/reference/d1ji4a_-d1rcd__.fasta

./group26/reference/d1h0oa_-d1qgha_.fasta

./group26/reference/d1jgca_-d1mtyd_.fasta

./group26/reference/d1lkoa1-d1qgha_.fasta

./group26/reference/d1jgca_-d1qgha_.fasta

./group26/reference/d1afra_-d1ji4a_.fasta

./group27/reference/d1lki__-d2gmfa_.fasta

./group27/reference/d1huw__-d1m4ra_.fasta

./group27/reference/d1au1a_-d2ilk__.fasta

./group27/reference/d1d9ca_-d1jli__.fasta

./group27/reference/d1hzia_-d1lqsl_.fasta

./group27/reference/d1au1a_-d1hula_.fasta

./group27/reference/d1eera_-d1lqsl_.fasta

./group27/reference/d1lqsl_-d2gmfa_.fasta

./group27/reference/d1au1a_-d2gmfa_.fasta

./group27/reference/d1jli__-d1lki__.fasta

./group28/reference/d1f4la1-d1iq0a1.fasta

./group28/reference/d1a8h_1-d1iq0a1.fasta

./group28/reference/d1f4la1-d1li5a1.fasta

./group28/reference/d1a8h_1-d1f4la1.fasta

./group28/reference/d1iq0a1-d1li5a1.fasta

./group28/reference/d1ile_1-d1iq0a1.fasta

./group28/reference/d1f7ua1-d1li5a1.fasta

./group28/reference/d1ile_1-d1li5a1.fasta

./group28/reference/d1a8h_1-d1f7ua1.fasta

./group28/reference/d1f4la1-d1ffya1.fasta

./group29/reference/d1af8__-d1dnya_.fasta

./group29/reference/d1af8__-d1dv5a_.fasta

./group29/reference/d1dnya_-d1dv5a_.fasta

./group29/reference/d1dnya_-d1klpa_.fasta

./group29/reference/d1dnya_-d1n8la_.fasta

./group29/reference/d1dv5a_-d1klpa_.fasta

./group29/reference/d1dv5a_-d1n8la_.fasta

./group29/reference/d1klpa_-d1n8la_.fasta

./group29/reference/d1l0ia_-d1n8la_.fasta

./group30/reference/d1d1da1-d2eiaa1.fasta

./group31/reference/d1fts_1-d1j8mf1.fasta

./group32/reference/d1eqfa1-d1jspb_.fasta

./group33/reference/d1efaa1-d1lmb3_.fasta

./group33/reference/d1e3oc2-d1zug__.fasta

./group33/reference/d1ic8a2-d1uxd__.fasta

./group33/reference/d1adr__-d1lmb3_.fasta

./group33/reference/d1d1la_-d1efaa1.fasta

./group33/reference/d1d1la_-d1lmb3_.fasta

./group33/reference/d1d1la_-d1ic8a2.fasta

./group33/reference/d1b0na2-d1d1la_.fasta

./group33/reference/d1ner__-d1uxd__.fasta

./group33/reference/d1lmb3_-d1uxd__.fasta

./group34/reference/d1an4a_-d1mdya_.fasta

./group35/reference/d1wdcb_-d2sas__.fasta

./group35/reference/d2pvba_-d2sas__.fasta

./group35/reference/d1psra_-d1rro__.fasta

./group35/reference/d1jfja_-d1m31a_.fasta

./group35/reference/d1eg3a1-d2sas__.fasta

./group35/reference/d1c07a_-d2sas__.fasta

./group35/reference/d1ggwa_-d1k94a_.fasta

./group35/reference/d1alva_-d1exra_.fasta

./group35/reference/d1ggwa_-d2sas__.fasta

./group35/reference/d1f8ha_-d1m31a_.fasta

./group36/reference/d1aoa_1-d1mb8a2.fasta

./group36/reference/d1aoa_2-d1bkra_.fasta

./group36/reference/d1bhda_-d1h67a_.fasta

./group36/reference/d1aoa_1-d1bhda_.fasta

./group36/reference/d1h67a_-d1mb8a2.fasta

./group36/reference/d1mb8a1-d1mb8a2.fasta

./group36/reference/d1bhda_-d1mb8a1.fasta

./group36/reference/d1bkra_-d1h67a_.fasta

./group36/reference/d1aoa_2-d1h67a_.fasta

./group36/reference/d1bkra_-d1mb8a1.fasta

./group37/reference/d1baza_-d1cmba_.fasta

./group37/reference/d1baza_-d1irqa_.fasta

./group37/reference/d1cmba_-d1irqa_.fasta

./group37/reference/d1cmba_-d2cpga_.fasta

./group38/reference/d1gnwa1-d1oe8a1.fasta

./group38/reference/d1eema1-d1g7oa1.fasta

./group38/reference/d1f2ea1-d1m0ua1.fasta

./group38/reference/d1gnwa1-d2gsta1.fasta

./group38/reference/d1f2ea1-d1gwca1.fasta

./group38/reference/d1a0fa1-d1glqa1.fasta

./group38/reference/d1ljra1-d1pmt_1.fasta

./group38/reference/d1axda1-d1duga1.fasta

./group38/reference/d1fw1a1-d1glqa1.fasta

./group38/reference/d1f2ea1-d1jlva1.fasta

./group39/reference/d1brwa1-d1o17a1.fasta

./group39/reference/d1khda1-d2tpt_1.fasta

./group40/reference/d1ez3a_-d1hs7a_.fasta

./group40/reference/d1ez3a_-d1lvfa_.fasta

./group40/reference/d1hs7a_-d1lvfa_.fasta

./group41/reference/d1bea__-d1fk5a_.fasta

./group41/reference/d1bea__-d1hyp__.fasta

./group41/reference/d1fk5a_-d1hyp__.fasta

./group41/reference/d1hyp__-d1l6ha_.fasta

./group44/reference/d1b0xa_-d1dxsa_.fasta

./group44/reference/d1b0xa_-d1oxja1.fasta

./group44/reference/d1b4fa_-d1bqv__.fasta

./group44/reference/d1b4fa_-d1dxsa_.fasta

./group44/reference/d1bqv__-d1dxsa_.fasta

./group44/reference/d1bqv__-d1oxja1.fasta

./group44/reference/d1dxsa_-d1oxja1.fasta

./group45/reference/d1cuk_2-d1dgsa1.fasta

./group45/reference/d1dgsa1-d1ixra1.fasta

./group45/reference/d1dgsa1-d1kfta_.fasta

./group46/reference/d1b43a1-d1tfr_1.fasta

./group46/reference/d1bgxt1-d1tfr_1.fasta

./group47/reference/d1a6s__-d1ed1a_.fasta

./group47/reference/d1a6s__-d1hiwa_.fasta

./group47/reference/d1ed1a_-d1mn8a_.fasta

./group47/reference/d1hiwa_-d1mn8a_.fasta

./group48/reference/d1l9la_-d1m12a_.fasta

./group48/reference/d1m12a_-d1nkl__.fasta

./group50/reference/d1e79a1-d1e79d1.fasta

./group50/reference/d1e79d1-d1fx0a1.fasta

./group51/reference/d1em9a_-d1m9fc_.fasta

./group51/reference/d1em9a_-d2eiaa2.fasta

./group52/reference/d1bu2a1-d1bu2a2.fasta

./group52/reference/d1f5qb1-d1jkw_2.fasta

./group52/reference/d1jkw_2-d1vin_2.fasta

./group52/reference/d1g3nc1-d1jkw_1.fasta

./group52/reference/d1g3nc1-d1h4ld_.fasta

./group52/reference/d1aisb2-d1g3nc1.fasta

./group52/reference/d1h4ld_-d1jkw_2.fasta

./group52/reference/d1bu2a2-d1f5qb1.fasta

./group52/reference/d1aisb1-d1f5qb2.fasta

./group52/reference/d1h4ld_-d1jkw_1.fasta

./group53/reference/d1a1w__-d1d2za_.fasta

./group53/reference/d1d2zb_-d1fada_.fasta

./group53/reference/d1n3ka_-d3ygsp_.fasta

./group53/reference/d1d2zb_-d1icha_.fasta

./group53/reference/d1icha_-d3ygsp_.fasta

./group53/reference/d1ddf__-d3ygsp_.fasta

./group53/reference/d1a1w__-d1d2zb_.fasta

./group53/reference/d1d2za_-d3crd__.fasta

./group53/reference/d1ddf__-d1ngr__.fasta

./group53/reference/d1a1w__-d1icha_.fasta

./group54/reference/d1iqpa1-d1jr3a1.fasta

./group54/reference/d1iqpa1-d1jr3d1.fasta

./group54/reference/d1jr3a1-d1jr3d1.fasta

./group56/reference/d1agre_-d1iapa_.fasta

./group56/reference/d1agre_-d1omwa1.fasta

./group56/reference/d1cmza_-d1iapa_.fasta

./group56/reference/d1cmza_-d1omwa1.fasta

./group57/reference/d1aru__-d1jdra_.fasta

./group57/reference/d1aru__-d1mwva1.fasta

./group57/reference/d1bgp__-d1llp__.fasta

./group57/reference/d1bgp__-d1mn2__.fasta

./group57/reference/d1bgp__-d1mwva1.fasta

./group57/reference/d1bgp__-d1mwva2.fasta

./group57/reference/d1cvua1-d1mwva2.fasta

./group57/reference/d1jdra_-d1llp__.fasta

./group57/reference/d1llp__-d1mwva2.fasta

./group57/reference/d1mn2__-d1mwva2.fasta

./group58/reference/d1mpga1-d1nkua_.fasta

./group58/reference/d1mun__-d1orna_.fasta

./group58/reference/d1nkua_-d2abk__.fasta

./group58/reference/d1ko9a1-d1nkua_.fasta

./group58/reference/d1mun__-d2abk__.fasta

./group58/reference/d1keaa_-d1mpga1.fasta

./group58/reference/d1ko9a1-d1mun__.fasta

./group58/reference/d1mpga1-d1mun__.fasta

./group58/reference/d1mpga1-d1ngna_.fasta

./group58/reference/d1mun__-d1nkua_.fasta

./group60/reference/d1dlja1-d1ks9a1.fasta

./group60/reference/d1ks9a1-d1mv8a1.fasta

./group60/reference/d1ks9a1-d1n1ea1.fasta

./group60/reference/d1ks9a1-d2pgd_1.fasta

./group60/reference/d1mv8a1-d1pgja1.fasta

./group60/reference/d1mv8a1-d2pgd_1.fasta

./group60/reference/d1n1ea1-d2pgd_1.fasta

./group61/reference/d1g9ga_-d1ks8a_.fasta

./group61/reference/d1kwfa_-d1nc5a_.fasta

./group61/reference/d1fp3a_-d1nc5a_.fasta

./group61/reference/d1clc_1-d1fp3a_.fasta

./group61/reference/d1h54a1-d1ks8a_.fasta

./group61/reference/d1fp3a_-d1g87a1.fasta

./group61/reference/d1ks8a_-d1kwfa_.fasta

./group61/reference/d1g87a1-d1kwfa_.fasta

./group61/reference/d1clc_1-d1nc5a_.fasta

./group61/reference/d1g9ga_-d1kwfa_.fasta

./group62/reference/d1cb8a1-d1hn0a1.fasta

./group62/reference/d1cb8a1-d1j0ma1.fasta

./group62/reference/d1cb8a1-d1qaza_.fasta

./group62/reference/d1hn0a1-d1j0ma1.fasta

./group62/reference/d1hn0a1-d1n7oa1.fasta

./group63/reference/d1c3d__-d1dceb_.fasta

./group63/reference/d1c3d__-d1ld8b_.fasta

./group63/reference/d1c3d__-d2sqca2.fasta

./group63/reference/d1dceb_-d2sqca2.fasta

./group63/reference/d1ld8b_-d2sqca2.fasta

./group63/reference/d2sqca2-d5eau_1.fasta

./group65/reference/d1dz4a_-d1e9xa_.fasta

./group65/reference/d1dz4a_-d1izoa_.fasta

./group65/reference/d1dz4a_-d1n97a_.fasta

./group65/reference/d1izoa_-d1n97a_.fasta

./group65/reference/d1io7a_-d1izoa_.fasta

./group65/reference/d1cpt__-d1izoa_.fasta

./group65/reference/d1e9xa_-d1izoa_.fasta

./group65/reference/d1izoa_-d1jipa_.fasta

./group65/reference/d1io7a_-d1n6ba_.fasta

./group65/reference/d1izoa_-d1jfba_.fasta

./group67/reference/d1pbwa_-d1wer__.fasta

./group67/reference/d1tx4a_-d1wer__.fasta

./group68/reference/d1b3ua_-d1bpoa1.fasta

./group68/reference/d1b3ua_-d1n8va_.fasta

./group68/reference/d1bpoa1-d1h6ka1.fasta

./group68/reference/d1bpoa1-d1n8va_.fasta

./group68/reference/d1bpoa1-d1oxja2.fasta

./group68/reference/d1h6ka1-d1h6ka2.fasta

./group69/reference/d1bd8__-d1dcqa1.fasta

./group69/reference/d1bi7b_-d1dcqa1.fasta

./group69/reference/d1bi7b_-d1ycsb1.fasta

./group69/reference/d1dcqa1-d1ihba_.fasta

./group70/reference/d1iyga_-d1kt1a1.fasta

./group70/reference/d1hh8a_-d1hz4a_.fasta

./group70/reference/d1elra_-d1ihga1.fasta

./group70/reference/d1elwa_-d1ihga1.fasta

./group70/reference/d1a17__-d1iyga_.fasta

./group70/reference/d1elra_-d1iyga_.fasta

./group70/reference/d1elra_-d1hz4a_.fasta

./group70/reference/d1hz4a_-d1kt1a1.fasta

./group70/reference/d1a17__-d1hh8a_.fasta

./group70/reference/d1hh8a_-d1iyga_.fasta

./group71/reference/d1dvpa1-d1eyha_.fasta

./group72/reference/d1a28a_-d1fcya_.fasta

./group72/reference/d1a28a_-d1ie9a_.fasta

./group72/reference/d1a28a_-d1n83a_.fasta

./group72/reference/d1a28a_-d2prga_.fasta

./group72/reference/d1ie9a_-d1pk5a_.fasta

./group72/reference/d1kv6a_-d1n83a_.fasta

./group72/reference/d1pk5a_-d2prga_.fasta

./group73/reference/d1ah7__-d1ak0__.fasta

./group73/reference/d1ak0__-d1ca1_1.fasta

./group74/reference/d1kxpd2-d1kxpd3.fasta

./group74/reference/d1kxpd2-d1n5ua3.fasta

./group74/reference/d1kxpd3-d1n5ua1.fasta

./group74/reference/d1kxpd3-d1n5ua3.fasta

./group74/reference/d1n5ua1-d1n5ua3.fasta

./group74/reference/d1n5ua2-d1n5ua3.fasta

./group75/reference/d1hy0a_-d1jswa_.fasta

./group76/reference/d1aokb_-d1lwba_.fasta

./group76/reference/d1buna_-d1lwba_.fasta

./group76/reference/d1kvoa_-d1lwba_.fasta

./group76/reference/d1le6a_-d1lwba_.fasta

./group76/reference/d1lfja_-d1lwba_.fasta

./group76/reference/d1lwba_-d1mc2a_.fasta

./group76/reference/d1lwba_-d1psj__.fasta

./group77/reference/d1ft5a_-d1wad__.fasta

./group77/reference/d19hca_-d1qo8a1.fasta

./group77/reference/d1qo8a1-d2ctha_.fasta

./group77/reference/d1dxrc_-d1fs7a_.fasta

./group77/reference/d19hca_-d1kssa1.fasta

./group77/reference/d1ft5a_-d3caoa_.fasta

./group77/reference/d1eysc_-d1gu6a_.fasta

./group77/reference/d1eysc_-d1kssa1.fasta

./group77/reference/d1m1qa_-d3cyr__.fasta

./group77/reference/d1aqe__-d1qo8a1.fasta

./group78/reference/d1eaja_-d1h5ba_.fasta

./group78/reference/d1hxmb1-d1nezg_.fasta

./group78/reference/d1eaja_-d2rhe__.fasta

./group78/reference/d1gsma2-d1nlbh1.fasta

./group78/reference/d1b88a_-d1nezg_.fasta

./group78/reference/d1eaja_-d1ncwh1.fasta

./group78/reference/d1gxea_-d2f5bh1.fasta

./group78/reference/d1eaja_-d1hxmb1.fasta

./group78/reference/d1g9mh1-d1nezg_.fasta

./group78/reference/d1dqta_-d1hxmb1.fasta

./group79/reference/d1bqua1-d1gh7a2.fasta

./group79/reference/d1iarb1-d1n26a2.fasta

./group79/reference/d1f6fb1-d2fnba_.fasta

./group79/reference/d1egja_-d1lqsr1.fasta

./group79/reference/d1bpv__-d1cd9b1.fasta

./group79/reference/d1lqsr2-d1n6va2.fasta

./group79/reference/d1gh7a2-d1lwra_.fasta

./group79/reference/d1f6fb1-d1lqsr1.fasta

./group79/reference/d1gh7a1-d1n6va1.fasta

./group79/reference/d1cfb_1-d1fyhb1.fasta

./group80/reference/d1bhga1-d1jz8a2.fasta

./group81/reference/d1kv3a2-d1l9na3.fasta

./group81/reference/d1f13a2-d1l9na2.fasta

./group81/reference/d1kv3a2-d1kv3a3.fasta

./group81/reference/d1kv3a3-d1l9na2.fasta

./group81/reference/d1f13a2-d1g0da3.fasta

./group81/reference/d1f13a3-d1l9na2.fasta

./group81/reference/d1g0da3-d1kv3a2.fasta

./group81/reference/d1l9na2-d1l9na3.fasta

./group81/reference/d1f13a3-d1kv3a2.fasta

./group81/reference/d1f13a2-d1l9na3.fasta

./group82/reference/d1edha2-d1l3wa5.fasta

./group82/reference/d1l3wa4-d1l3wa5.fasta

./group84/reference/d1ej8a_-d1eso__.fasta

./group84/reference/d1ej8a_-d1oala_.fasta

./group85/reference/d1e42a1-d1gyva_.fasta

./group85/reference/d1e42a1-d1kyfa1.fasta

./group85/reference/d1e42a1-d1p4ua_.fasta

./group85/reference/d1gyva_-d1kyfa1.fasta

./group86/reference/d1cwva1-d1cwva3.fasta

./group86/reference/d1cwva1-d1cwva4.fasta

./group86/reference/d1cwva1-d1f00i2.fasta

./group86/reference/d1cwva2-d1cwva4.fasta

./group86/reference/d1cwva2-d1f00i2.fasta

./group86/reference/d1cwva3-d1cwva4.fasta

./group86/reference/d1cwva3-d1f00i1.fasta

./group86/reference/d1cwva3-d1f00i2.fasta

./group86/reference/d1cwva4-d1f00i1.fasta

./group86/reference/d1f00i1-d1f00i2.fasta

./group87/reference/d1exh__-d1qba_2.fasta

./group87/reference/d1aoha_-d1g43a_.fasta

./group87/reference/d1aoha_-d1nbca_.fasta

./group87/reference/d1nbca_-d1qba_2.fasta

./group87/reference/d1e5ba_-d1qba_2.fasta

./group87/reference/d1e5ba_-d1g43a_.fasta

./group87/reference/d1g43a_-d1qba_2.fasta

./group87/reference/d1exh__-d1nbca_.fasta

./group87/reference/d1e5ba_-d1g1ka_.fasta

./group87/reference/d1exh__-d1g1ka_.fasta

./group88/reference/d1klfb1-d1pdkb_.fasta

./group88/reference/d1amx__-d1n67a1.fasta

./group88/reference/d1amx__-d1klfb2.fasta

./group88/reference/d1amx__-d1n67a2.fasta

./group88/reference/d1klfb2-d1n67a1.fasta

./group88/reference/d1klfb2-d1pdkb_.fasta

./group88/reference/d1p5vb_-d1pdkb_.fasta

./group88/reference/d1amx__-d1pdkb_.fasta

./group88/reference/d1n67a1-d1n67a2.fasta

./group88/reference/d1n67a1-d1p5vb_.fasta

./group89/reference/d1a02n2-d1bg1a2.fasta

./group89/reference/d1a02n2-d1h6fa_.fasta

./group89/reference/d1a3qa2-d1bg1a2.fasta

./group89/reference/d1bg1a2-d1bvoa_.fasta

./group89/reference/d1bg1a2-d1h6fa_.fasta

./group89/reference/d1bg1a2-d1imhc2.fasta

./group92/reference/d1hfua3-d1kv7a1.fasta

./group92/reference/d1hfua3-d1qhqa_.fasta

./group92/reference/d1gw0a1-d1gw0a2.fasta

./group92/reference/d1aoza2-d1ocrb1.fasta

./group92/reference/d1hfua3-d1kcw_5.fasta

./group92/reference/d1kcw_2-d1kcw_5.fasta

./group92/reference/d1aoza3-d1kcw_4.fasta

./group92/reference/d1gska2-d1gw0a1.fasta

./group92/reference/d1fwxa1-d1ikop_.fasta

./group92/reference/d1aoza2-d1qhqa_.fasta

./group93/reference/d1dqva1-d1rlw__.fasta

./group93/reference/d1rlw__-d3rpba_.fasta

./group94/reference/d1k2fa_-d1lb6a_.fasta

./group95/reference/d1bhu__-d1h4ax1.fasta

./group95/reference/d1bhu__-d1h4ax2.fasta

./group95/reference/d1c01a_-d1h4ax1.fasta

./group95/reference/d1g6ea_-d2bb2_2.fasta

./group95/reference/d1g6ea_-d1h4ax1.fasta

./group95/reference/d1f53a_-d1ha4a_.fasta

./group95/reference/d1g6ea_-d1h4ax2.fasta

./group95/reference/d1c01a_-d1g6ea_.fasta

./group95/reference/d1f53a_-d1h4ax2.fasta

./group95/reference/d1c01a_-d1f53a_.fasta

./group97/reference/d1hx6a1-d1m3ya1.fasta

./group97/reference/d1hx6a1-d1m3ya2.fasta

./group97/reference/d1hx6a2-d1m3ya2.fasta

./group98/reference/d1ejfa_-d1gmea_.fasta

./group98/reference/d1ejfa_-d1shsa_.fasta

./group99/reference/d1gnya_-d1guia_.fasta

./group99/reference/d1gu3a_-d1kgya_.fasta

./group99/reference/d1bhga2-d1gnya_.fasta

./group99/reference/d1jhja_-d1k42a_.fasta

./group99/reference/d1jz8a3-d1of4a_.fasta

./group99/reference/d1d7pm_-d1dlc_1.fasta

./group99/reference/d1i5pa1-d1jz8a3.fasta

./group99/reference/d1jz8a3-d1lnsa2.fasta

./group99/reference/d1gnya_-d1of4a_.fasta

./group99/reference/d1gwma_-d1of4a_.fasta

./group100/reference/d1ahsa_-d1flca1.fasta

./group100/reference/d1ahsa_-d1jsda_.fasta

./group100/reference/d1bvp12-d1jsma_.fasta

./group100/reference/d1flca1-d1jsda_.fasta

./group100/reference/d1flca1-d1jsma_.fasta

./group100/reference/d1jsma_-d1qhda2.fasta

./group101/reference/d1h7za_-d1kkea1.fasta

./group101/reference/d1kaca_-d1kkea1.fasta

./group101/reference/d1kkea1-d1qhva_.fasta

./group102/reference/d1aly__-d1gr3a_.fasta

./group102/reference/d1gr3a_-d1kxga_.fasta

./group102/reference/d1gr3a_-d1tnra_.fasta

./group102/reference/d1gr3a_-d2tnfa_.fasta

./group103/reference/d1f1sa3-d1hn0a3.fasta

./group103/reference/d1f1sa3-d1j0ma2.fasta

./group104/reference/d1dmza_-d1gxca_.fasta

./group104/reference/d1dmza_-d1lgpa_.fasta

./group105/reference/d1bkza_-d1nls__.fasta

./group105/reference/d1d2sa_-d2sli_1.fasta

./group105/reference/d1kit_1-d2ayh__.fasta

./group105/reference/d1dyka2-d1kit_2.fasta

./group105/reference/d1d2sa_-d2pela_.fasta

./group105/reference/d1g86a_-d1xnb__.fasta

./group105/reference/d1d2sa_-d1kit_1.fasta

./group105/reference/d1c4ra_-d1d2sa_.fasta

./group105/reference/d1n1ta1-d2sli_1.fasta

./group105/reference/d1saca_-d1xnb__.fasta

./group106/reference/d1fqta_-d1g8kb_.fasta

./group106/reference/d1fqta_-d1o7na1.fasta

./group106/reference/d1fqta_-d1rfs__.fasta

./group106/reference/d1fqta_-d1rie__.fasta

./group106/reference/d1g8kb_-d1o7na1.fasta

./group106/reference/d1g8kb_-d1rie__.fasta

./group106/reference/d1nyka_-d1o7na1.fasta

./group106/reference/d1o7na1-d1rie__.fasta

./group107/reference/d1bia_2-d1igqa_.fasta

./group107/reference/d1fx7a3-d1igqa_.fasta

./group108/reference/d1awj__-d1gcqc_.fasta

./group108/reference/d1h92a_-d1i07a_.fasta

./group108/reference/d1awj__-d1i07a_.fasta

./group108/reference/d1gcqc_-d1neb__.fasta

./group108/reference/d1awj__-d1neb__.fasta

./group108/reference/d1bbza_-d1gcqc_.fasta

./group108/reference/d1i07a_-d1ng2a2.fasta

./group108/reference/d1bbza_-d1ng2a1.fasta

./group108/reference/d1i1ja_-d1ycsb2.fasta

./group108/reference/d1gcqc_-d1jqqa_.fasta

./group109/reference/d1jb0e_-d2ahjb_.fasta

./group110/reference/d1jj2a1-d1jj2p_.fasta

./group110/reference/d1jj2a1-d1khia1.fasta

./group110/reference/d1jj2a1-d1m1ga2.fasta

./group110/reference/d1jj2p_-d1jj2s_.fasta

./group110/reference/d1jj2p_-d1khia1.fasta

./group110/reference/d1jj2p_-d2eifa1.fasta

./group110/reference/d1jj2s_-d1khia1.fasta

./group110/reference/d1jj2s_-d2eifa1.fasta

./group110/reference/d1khia1-d1m1ga2.fasta

./group110/reference/d1m1ga2-d2eifa1.fasta

./group111/reference/d1aono_-d1g31a_.fasta

./group111/reference/d1g31a_-d1p3ha_.fasta

./group112/reference/d1m5za_-d1ntea_.fasta

./group112/reference/d1ihja_-d1qaua_.fasta

./group112/reference/d1be9a_-d1k32a1.fasta

./group112/reference/d1kwaa_-d1m5za_.fasta

./group112/reference/d1ntea_-d1qava_.fasta

./group112/reference/d1kwaa_-d1qaua_.fasta

./group112/reference/d1ntea_-d1qlca_.fasta

./group112/reference/d1k32a1-d1qava_.fasta

./group112/reference/d1d5ga_-d1k32a1.fasta

./group112/reference/d1ihja_-d1k32a1.fasta

./group113/reference/d1d3ba_-d1n9ra_.fasta

./group113/reference/d1d3ba_-d1kq1a_.fasta

./group113/reference/d1d3ba_-d1d3bb_.fasta

./group113/reference/d1i8fa_-d1mxma1.fasta

./group113/reference/d1d3ba_-d1mxma1.fasta

./group113/reference/d1mxma1-d1n9ra_.fasta

./group113/reference/d1d3bb_-d1kq1a_.fasta

./group113/reference/d1i8fa_-d1kq1a_.fasta

./group113/reference/d1mgqa_-d1mxma1.fasta

./group113/reference/d1d3bb_-d1mxma1.fasta

./group114/reference/d1eu3a1-d3chbd_.fasta

./group114/reference/d1enfa1-d3chbd_.fasta

./group114/reference/d1prtf_-d3tss_1.fasta

./group114/reference/d1prtb1-d1prtd_.fasta

./group114/reference/d1eu3a1-d1prtd_.fasta

./group114/reference/d1prtf_-d3seb_1.fasta

./group114/reference/d1fnua1-d1prtb1.fasta

./group114/reference/d1c4qa_-d1fnua1.fasta

./group114/reference/d1enfa1-d1eu3a1.fasta

./group114/reference/d1an8_1-d3tss_1.fasta

./group115/reference/d1br9__-d1jb3a_.fasta

./group115/reference/d1br9__-d1uapa_.fasta

./group115/reference/d1jb3a_-d1uapa_.fasta

./group116/reference/d1jjcb3-d1qvca_.fasta

./group116/reference/d1jb7a2-d1jb7b_.fasta

./group116/reference/d1fgua2-d1gm5a2.fasta

./group116/reference/d1eova1-d1jb7a1.fasta

./group116/reference/d1gd7a_-d1qvca_.fasta

./group116/reference/d1fjgq_-d1kxla_.fasta

./group116/reference/d1gd7a_-d1o7ia_.fasta

./group116/reference/d1fgua2-d1kxla_.fasta

./group116/reference/d1fgua1-d1o7ia_.fasta

./group116/reference/d1fl0a_-d1ltla_.fasta

./group118/reference/d1g2913-d1oxsc1.fasta

./group118/reference/d1fr3a_-d1oxsc1.fasta

./group118/reference/d1g2914-d1h9ma1.fasta

./group118/reference/d1g2913-d1h9ma1.fasta

./group118/reference/d1g2913-d1h9ra2.fasta

./group118/reference/d1g2914-d1oxsc1.fasta

./group118/reference/d1h9ma1-d1oxsc1.fasta

./group118/reference/d1fr3a_-d1g2914.fasta

./group118/reference/d1g2913-d1h9ma2.fasta

./group118/reference/d1guta_-d1oxsc1.fasta

./group119/reference/d1bfg__-d1ilr1_.fasta

./group119/reference/d1ijta_-d1ilr1_.fasta

./group119/reference/d1ilr1_-d1qqla_.fasta

./group120/reference/d1abrb2-d1m2tb1.fasta

./group120/reference/d1dqga_-d1ggpb1.fasta

./group120/reference/d1dqga_-d1hwmb1.fasta

./group120/reference/d1dqga_-d1hwmb2.fasta

./group120/reference/d1dqga_-d1m2tb1.fasta

./group121/reference/d1eyla_-d3btaa2.fasta

./group121/reference/d1a8d_2-d1avwb_.fasta

./group121/reference/d1avac_-d1epwa2.fasta

./group121/reference/d1a8d_2-d1wba__.fasta

./group121/reference/d1a8d_2-d1avac_.fasta

./group121/reference/d1avwb_-d1epwa2.fasta

./group121/reference/d1wba__-d3btaa2.fasta

./group121/reference/d1avwb_-d3btaa2.fasta

./group121/reference/d1epwa2-d1eyla_.fasta

./group121/reference/d1avac_-d3btaa2.fasta

./group122/reference/d1dfca2-d1hcd__.fasta

./group123/reference/d1dar_1-d1f60a1.fasta

./group123/reference/d1f60a1-d1n0ua1.fasta

./group125/reference/d1ci0a_-d1i0ra_.fasta

./group125/reference/d1ejea_-d1flma_.fasta

./group125/reference/d1ejea_-d1i0ra_.fasta

./group125/reference/d1flma_-d1i0ra_.fasta

./group126/reference/d1arb__-d1gvkb_.fasta

./group126/reference/d1arb__-d1ekbb_.fasta

./group126/reference/d1hj9a_-d1qtfa_.fasta

./group126/reference/d1agja_-d1ekbb_.fasta

./group126/reference/d1ltoa_-d1qtfa_.fasta

./group126/reference/d1arb__-d1azza_.fasta

./group126/reference/d1gvkb_-d1qtfa_.fasta

./group126/reference/d1arb__-d1eaxa_.fasta

./group126/reference/d1arb__-d1bio__.fasta

./group126/reference/d1agja_-d1mzaa_.fasta

./group127/reference/d1e79a2-d1e79d2.fasta

./group127/reference/d1e79a2-d1fx0b2.fasta

./group128/reference/d1fkna_-d1fmb__.fasta

./group128/reference/d1j71a_-d1kzka_.fasta

./group128/reference/d1fmb__-d1mpp__.fasta

./group128/reference/d1nsoa_-d2apr__.fasta

./group128/reference/d1lf2a_-d1nsoa_.fasta

./group128/reference/d1idaa_-d2apr__.fasta

./group128/reference/d1dpja_-d1idaa_.fasta

./group128/reference/d1kzka_-d2apr__.fasta

./group128/reference/d1fmb__-d2apr__.fasta

./group128/reference/d2apr__-d4fiv__.fasta

./group130/reference/d1cz4a1-d1tmo_1.fasta

./group130/reference/d1e32a1-d1eu1a1.fasta

./group130/reference/d1cz4a1-d1eu1a1.fasta

./group130/reference/d1cr5a1-d1e32a1.fasta

./group130/reference/d1cr5a1-d1eu1a1.fasta

./group130/reference/d1cr5a1-d1tmo_1.fasta

./group130/reference/d1e32a1-d1kqfa1.fasta

./group130/reference/d1cr5a1-d1h0ha1.fasta

./group130/reference/d1eu1a1-d1g8ka1.fasta

./group130/reference/d1e32a1-d2napa1.fasta

./group131/reference/d1h4ra2-d1shca_.fasta

./group131/reference/d1evha_-d1k5db_.fasta

./group131/reference/d1dro__-d1eaza_.fasta

./group131/reference/d1dyna_-d1qqga2.fasta

./group131/reference/d1k5db_-d1ntva_.fasta

./group131/reference/d1mai__-d1mixa2.fasta

./group131/reference/d1faoa_-d1ntva_.fasta

./group131/reference/d1dyna_-d1fhoa_.fasta

./group131/reference/d1eaza_-d1qqga2.fasta

./group131/reference/d1faoa_-d1mkea1.fasta

./group133/reference/d1ggla_-d1i4ua_.fasta

./group133/reference/d1jv4a_-d1p6pa_.fasta

./group133/reference/d1euoa_-d1qqsa_.fasta

./group133/reference/d1gkab_-d1o1va_.fasta

./group133/reference/d1euoa_-d1ew3a_.fasta

./group133/reference/d1avgi_-d1jzua_.fasta

./group133/reference/d1bj7__-d1mdc__.fasta

./group133/reference/d1b56__-d1bj7__.fasta

./group133/reference/d1dzka_-d1gkab_.fasta

./group133/reference/d1jzua_-d1qqsa_.fasta

./group136/reference/d1e8ua_-d1nsca_.fasta

./group136/reference/d1eur__-d1nsca_.fasta

./group136/reference/d1n1ta2-d1nsca_.fasta

./group136/reference/d1eur__-d2bat__.fasta

./group136/reference/d1e8ua_-d2bat__.fasta

./group136/reference/d1nsca_-d2sli_2.fasta

./group136/reference/d1e8ua_-d2sli_2.fasta

./group136/reference/d1n1ta2-d2bat__.fasta

./group136/reference/d1e8ua_-d1n1ta2.fasta

./group136/reference/d1eur__-d1f8ea_.fasta

./group137/reference/d1nr0a1-d1nr0a2.fasta

./group137/reference/d1nr0a1-d1tbga_.fasta

./group137/reference/d1nr0a2-d1p22a2.fasta

./group137/reference/d1nr0a2-d1tbga_.fasta

./group138/reference/d1e43a1-d7taa_1.fasta

./group138/reference/d1m53a1-d1mxga1.fasta

./group138/reference/d1gjwa1-d1iv8a1.fasta

./group138/reference/d1g5aa1-d1qhoa3.fasta

./group138/reference/d1g94a1-d1ji2a2.fasta

./group138/reference/d1jae_1-d1ji2a2.fasta

./group138/reference/d1bag_1-d7taa_1.fasta

./group138/reference/d1j0ha2-d1mxga1.fasta

./group138/reference/d1kwga1-d1qhoa3.fasta

./group138/reference/d1jae_1-d1m53a1.fasta

./group141/reference/d1ciy_2-d1i5pa2.fasta

./group143/reference/d1czfa_-d1rmg__.fasta

./group143/reference/d1bn8a_-d1dbga_.fasta

./group143/reference/d1czfa_-d1qcxa_.fasta

./group143/reference/d1k5ca_-d1qjva_.fasta

./group143/reference/d1bn8a_-d1czfa_.fasta

./group143/reference/d1bhe__-d1qcxa_.fasta

./group143/reference/d1hg8a_-d1qjva_.fasta

./group143/reference/d1czfa_-d1daba_.fasta

./group143/reference/d1ee6a_-d1jtaa_.fasta

./group143/reference/d1bn8a_-d1k5ca_.fasta

./group144/reference/d1kk6a_-d1qrea_.fasta

./group144/reference/d1krra_-d3tdt__.fasta

./group144/reference/d1qrea_-d1xat__.fasta

./group144/reference/d1xat__-d3tdt__.fasta

./group145/reference/d1dgwa_-d1dzra_.fasta

./group145/reference/d1dgwa_-d1ep0a_.fasta

./group145/reference/d1ep0a_-d1od5a2.fasta

./group145/reference/d1fi2a_-d1o4ta_.fasta

./group145/reference/d1dzra_-d1lkna_.fasta

./group145/reference/d1ep0a_-d1fi2a_.fasta

./group145/reference/d1fxza2-d2phla1.fasta

./group145/reference/d1dgwa_-d1m4oa_.fasta

./group145/reference/d1fi2a_-d1lkna_.fasta

./group145/reference/d1lkna_-d1o4ta_.fasta

./group146/reference/d1ds1a_-d1gp6a_.fasta

./group146/reference/d1ds1a_-d1gy9a_.fasta

./group146/reference/d1ds1a_-d1odma_.fasta

./group146/reference/d1gp6a_-d1gy9a_.fasta

./group147/reference/d1cx4a1-d1ft9a2.fasta

./group147/reference/d1cx4a1-d1i5za2.fasta

./group147/reference/d1ft9a2-d1o7fa2.fasta

./group147/reference/d1ft9a2-d1o7fa3.fasta

./group147/reference/d1ft9a2-d1rgs_1.fasta

./group147/reference/d1ft9a2-d1rgs_2.fasta

./group147/reference/d1i5za2-d1rgs_2.fasta

./group148/reference/d1fyc__-d1htp__.fasta

./group148/reference/d1ghk__-d1htp__.fasta

./group148/reference/d1htp__-d1k8ma_.fasta

./group149/reference/d1b6ra1-d1dv1a1.fasta

./group149/reference/d1b6ra1-d1e2wa2.fasta

./group149/reference/d1dv1a1-d1hcz_2.fasta

./group149/reference/d1e2wa2-d1kjqa1.fasta

./group152/reference/d1dun__-d1ogha_.fasta

./group154/reference/d1a53__-d1nsj__.fasta

./group154/reference/d1eixa_-d1kv8a_.fasta

./group154/reference/d1i4na_-d1thfd_.fasta

./group154/reference/d1pii_1-d1rpxa_.fasta

./group154/reference/d1a53__-d1dqwa_.fasta

./group154/reference/d1i4na_-d1nsj__.fasta

./group154/reference/d1eixa_-d1pii_2.fasta

./group154/reference/d1nsj__-d1thfd_.fasta

./group154/reference/d1dbta_-d1i4na_.fasta

./group154/reference/d1eixa_-d1thfd_.fasta

./group155/reference/d1ep3a_-d1o94a1.fasta

./group155/reference/d1ep3a_-d1oyb__.fasta

./group155/reference/d1gvoa_-d2dora_.fasta

./group155/reference/d1oyb__-d2dora_.fasta

./group157/reference/d1eswa_-d1ug6a_.fasta

./group157/reference/d1gjwa2-d1qhoa4.fasta

./group157/reference/d1iexa1-d1m53a2.fasta

./group157/reference/d1j18a2-d1qvba_.fasta

./group157/reference/d1hxja_-d1m53a2.fasta

./group157/reference/d1cbg__-d1qhoa4.fasta

./group157/reference/d1bf2_3-d1qvba_.fasta

./group157/reference/d1cz1a_-d1lwha2.fasta

./group157/reference/d1bf2_3-d1cz1a_.fasta

./group157/reference/d1qvba_-d7taa_2.fasta

./group158/reference/d1a4ma_-d1j5sa_.fasta

./group158/reference/d1i0da_-d1p1ma2.fasta

./group158/reference/d1m7ja3-d1p1ma2.fasta

./group158/reference/d1itua_-d1p1ma2.fasta

./group158/reference/d1i0da_-d1itua_.fasta

./group158/reference/d1itua_-d1k6wa2.fasta

./group158/reference/d1bf6a_-d1j5sa_.fasta

./group158/reference/d1bf6a_-d1m7ja3.fasta

./group158/reference/d1i0da_-d1k6wa2.fasta

./group158/reference/d1a4ma_-d1bf6a_.fasta

./group159/reference/d1mzha_-d1qfea_.fasta

./group159/reference/d1jcla_-d1nvma2.fasta

./group159/reference/d1i2oa_-d1jcla_.fasta

./group159/reference/d1o0ya_-d1qfea_.fasta

./group159/reference/d1dosa_-d1n8fa_.fasta

./group159/reference/d1gzga_-d1qfea_.fasta

./group159/reference/d1nvma2-d1ub3a_.fasta

./group159/reference/d1dhpa_-d1ohla_.fasta

./group159/reference/d1f74a_-d1i2oa_.fasta

./group159/reference/d1epxa_-d1o0ya_.fasta

./group160/reference/d1onea1-d2mnr_1.fasta

./group160/reference/d1muca1-d1onea1.fasta

./group160/reference/d1ec7a1-d1muca1.fasta

./group160/reference/d2chr_1-d2mnr_1.fasta

./group160/reference/d1e9ia1-d1ec7a1.fasta

./group160/reference/d1jpma1-d1onea1.fasta

./group160/reference/d1e9ia1-d1jpma1.fasta

./group160/reference/d1jpdx1-d1onea1.fasta

./group160/reference/d1e9ia1-d1muca1.fasta

./group160/reference/d1e9ia1-d1jpdx1.fasta

./group161/reference/d1dxea_-d1f8ma_.fasta

./group161/reference/d1izca_-d1m3ua_.fasta

./group161/reference/d1izca_-d1muma_.fasta

./group161/reference/d1kbla1-d1m3ua_.fasta

./group161/reference/d1izca_-d1kbla1.fasta

./group161/reference/d1dxea_-d1kbla1.fasta

./group161/reference/d1m3ua_-d1muma_.fasta

./group161/reference/d1f8ma_-d1m3ua_.fasta

./group161/reference/d1f8ma_-d1izca_.fasta

./group161/reference/d1dxea_-d1muma_.fasta

./group162/reference/d1a0ca_-d1qtwa_.fasta

./group162/reference/d1muwa_-d1qtwa_.fasta

./group163/reference/d1ezwa_-d1lucb_.fasta

./group164/reference/d1o1za_-d2plc__.fasta

./group164/reference/d1o1za_-d2ptd__.fasta

./group165/reference/d1ccwb_-d1eexa_.fasta

./group165/reference/d1ccwb_-d7reqa1.fasta

./group165/reference/d1ccwb_-d7reqb1.fasta

./group165/reference/d1eexa_-d7reqb1.fasta

./group166/reference/d1gega_-d1gz3a1.fasta

./group166/reference/d1gcoa_-d1n2sa_.fasta

./group166/reference/d1gz3a1-d1hwxa1.fasta

./group166/reference/d1hwxa1-d1n5da_.fasta

./group166/reference/d1eny__-d1kewa_.fasta

./group166/reference/d1bgva1-d1o0sa1.fasta

./group166/reference/d1hwxa1-d2ae2a_.fasta

./group166/reference/d1iy8a_-d1kepa_.fasta

./group166/reference/d1hwxa1-d1iy8a_.fasta

./group166/reference/d1gcoa_-d1qg6a_.fasta

./group167/reference/d1ojt_2-d1trb_2.fasta

./group167/reference/d1fl2a2-d1onfa2.fasta

./group167/reference/d1mo9a2-d3lada2.fasta

./group167/reference/d1fl2a2-d1o94a2.fasta

./group167/reference/d1cjca1-d1m6ia2.fasta

./group167/reference/d1mo9a2-d1trb_2.fasta

./group167/reference/d1ojt_2-d1onfa2.fasta

./group167/reference/d1m6ia2-d1trb_2.fasta

./group167/reference/d1cjca1-d1ojt_2.fasta

./group167/reference/d1ebda2-d1m6ia2.fasta

./group170/reference/d1a4ya_-d1io0a_.fasta

./group170/reference/d1a4ya_-d1pgva_.fasta

./group170/reference/d1fqva2-d1io0a_.fasta

./group170/reference/d1fqva2-d1pgva_.fasta

./group170/reference/d1fqva2-d1yrga_.fasta

./group170/reference/d1io0a_-d1yrga_.fasta

./group170/reference/d1pgva_-d1yrga_.fasta

./group171/reference/d1igra1-d1p9ag_.fasta

./group171/reference/d1koha1-d1nqla1.fasta

./group171/reference/d1m6ba2-d1ozna_.fasta

./group171/reference/d1h6ta2-d1nqla2.fasta

./group171/reference/d1dcea3-d1nqla1.fasta

./group171/reference/d1igra1-d1koha1.fasta

./group171/reference/d1a9na_-d1n8yc2.fasta

./group171/reference/d1jl5a_-d1m6ba2.fasta

./group171/reference/d1h6ta2-d1jl5a_.fasta

./group171/reference/d1n8yc2-d1ogqa_.fasta

./group172/reference/d1o8ua_-d1on3a2.fasta

./group172/reference/d1hzda_-d1on3a1.fasta

./group172/reference/d1nzya_-d1on3a1.fasta

./group172/reference/d1on3a2-d1tyfa_.fasta

./group172/reference/d1o8ua_-d1on3a1.fasta

./group172/reference/d1nzya_-d1on3a2.fasta

./group172/reference/d1hzda_-d1on3a2.fasta

./group172/reference/d1dcia_-d1on3a1.fasta

./group172/reference/d1hzda_-d1tyfa_.fasta

./group172/reference/d1dcia_-d1tyfa_.fasta

./group173/reference/d1cdza_-d1l0ba1.fasta

./group173/reference/d1dgtb3-d1l0ba1.fasta

./group173/reference/d1in1a_-d1l0ba1.fasta

./group175/reference/d1l9ga_-d1laue_.fasta

./group175/reference/d1l9ga_-d1oe4a_.fasta

./group175/reference/d1l9ga_-d3euga_.fasta

./group175/reference/d1laue_-d1muga_.fasta

./group175/reference/d1laue_-d1oe4a_.fasta

./group175/reference/d1muga_-d1oe4a_.fasta

./group175/reference/d1muga_-d3euga_.fasta

./group175/reference/d1oe4a_-d3euga_.fasta

./group176/reference/d1b00a_-d1m2fa_.fasta

./group176/reference/d1m2fa_-d1nat__.fasta

./group176/reference/d1ntr__-d1qo0d_.fasta

./group176/reference/d1dbwa_-d1qo0d_.fasta

./group176/reference/d1a04a2-d1m2fa_.fasta

./group176/reference/d1m2fa_-d1ntr__.fasta

./group176/reference/d1qo0d_-d1tmy__.fasta

./group176/reference/d1dbwa_-d1dz3a_.fasta

./group176/reference/d1dz3a_-d1qo0d_.fasta

./group176/reference/d1mvoa_-d1qo0d_.fasta

./group177/reference/d1e5da1-d1ja1a2.fasta

./group177/reference/d1qr2a_-d5nul__.fasta

./group177/reference/d1e5da1-d1oboa_.fasta

./group177/reference/d1d4aa_-d1f4pa_.fasta

./group177/reference/d1d4aa_-d1e5da1.fasta

./group177/reference/d1ag9a_-d1d4aa_.fasta

./group177/reference/d1e5da1-d2fcr__.fasta

./group177/reference/d1e5da1-d1qr2a_.fasta

./group177/reference/d1ja1a2-d5nul__.fasta

./group177/reference/d1fuea_-d5nul__.fasta

./group178/reference/d1bmta2-d7reqa2.fasta

./group178/reference/d1bmta2-d7reqb2.fasta

./group178/reference/d1ccwa_-d7reqb2.fasta

./group178/reference/d7reqa2-d7reqb2.fasta

./group179/reference/d1esc__-d1k7ca_.fasta

./group181/reference/d1i7qb_-d1k9vf_.fasta

./group181/reference/d1cf9a1-d1ka9h_.fasta

./group181/reference/d1kwga3-d1l9xa_.fasta

./group181/reference/d1pe0a_-d1qdlb_.fasta

./group181/reference/d1i7qb_-d1pe0a_.fasta

./group181/reference/d1cf9a1-d1qdlb_.fasta

./group181/reference/d1k9vf_-d1kwga3.fasta

./group181/reference/d1ka9h_-d1kwga3.fasta

./group181/reference/d1cf9a1-d1gpma2.fasta

./group181/reference/d1l9xa_-d1qdlb_.fasta

./group182/reference/d1fdr_2-d2pia_2.fasta

./group182/reference/d1gvha3-d1que_2.fasta

./group182/reference/d1qfja2-d2pia_2.fasta

./group182/reference/d1ep3b2-d2cnd_2.fasta

./group182/reference/d1cqxa3-d1ep3b2.fasta

./group182/reference/d1a8p_2-d2cnd_2.fasta

./group182/reference/d1f20a2-d2pia_2.fasta

./group182/reference/d1ddga2-d1qfja2.fasta

./group182/reference/d1ja1a3-d2pia_2.fasta

./group182/reference/d1cqxa3-d1ja1a3.fasta

./group183/reference/d1j09a2-d1jhda2.fasta

./group183/reference/d1ej2a_-d1qjca_.fasta

./group183/reference/d1k4ma_-d1n2ea_.fasta

./group183/reference/d1k4ma_-d1qjca_.fasta

./group183/reference/d1f7ua2-d1qjca_.fasta

./group183/reference/d1ihoa_-d1qjca_.fasta

./group183/reference/d1jila_-d1k4ma_.fasta

./group183/reference/d1g8fa2-d1qjca_.fasta

./group183/reference/d1a8h_2-d1ihoa_.fasta

./group183/reference/d1a8h_2-d1jila_.fasta

./group184/reference/d1jmva_-d1k92a1.fasta

./group184/reference/d1efva1-d1efvb_.fasta

./group184/reference/d1jmva_-d1o97d1.fasta

./group184/reference/d1efpa1-d1jmva_.fasta

./group184/reference/d1efpa1-d1efvb_.fasta

./group184/reference/d1efvb_-d1kqpa_.fasta

./group184/reference/d1j20a1-d1kqpa_.fasta

./group184/reference/d1j20a1-d1sur__.fasta

./group184/reference/d1efva1-d1jmva_.fasta

./group184/reference/d1j20a1-d1jmva_.fasta

./group186/reference/d1b6ra2-d1i7na1.fasta

./group186/reference/d1b6ra2-d2hgsa1.fasta

./group186/reference/d1a9xa4-d1e4ea1.fasta

./group186/reference/d1a9xa3-d1kjqa2.fasta

./group186/reference/d1e4ea1-d1kjqa2.fasta

./group186/reference/d1i7na1-d2hgsa1.fasta

./group186/reference/d1a9xa3-d1iow_1.fasta

./group186/reference/d1e4ea1-d1gsoa2.fasta

./group186/reference/d1a9xa3-d1gsa_1.fasta

./group186/reference/d1kjqa2-d1m0wa1.fasta

./group187/reference/d1bfd_1-d1m2ka_.fasta

./group187/reference/d1efva2-d1poxa1.fasta

./group187/reference/d1d4oa_-d1o97d2.fasta

./group187/reference/d1dhs__-d1o97d2.fasta

./group187/reference/d1o97d2-d1poxa1.fasta

./group187/reference/d1efva2-d1hzzc_.fasta

./group187/reference/d1ovma1-d1poxa1.fasta

./group187/reference/d1dhs__-d1efva2.fasta

./group187/reference/d1bfd_1-d1o97d2.fasta

./group187/reference/d1bfd_1-d1d4oa_.fasta

./group188/reference/d1fsz_1-d1tuba1.fasta

./group188/reference/d1fsz_1-d1tubb1.fasta

./group188/reference/d1ofua1-d1tuba1.fasta

./group188/reference/d1ofua1-d1tubb1.fasta

./group188/reference/d1oi2a1-d1tuba1.fasta

./group189/reference/d1nbaa_-d1nf9a_.fasta

./group189/reference/d1nbaa_-d1yaca_.fasta

./group190/reference/d1poxa2-d1poxa3.fasta

./group190/reference/d1poxa3-d1pvda3.fasta

./group190/reference/d1keka1-d1zpda2.fasta

./group190/reference/d1gpua2-d1qgda2.fasta

./group190/reference/d1bfd_2-d1ovma3.fasta

./group190/reference/d1dtwb1-d1qgda2.fasta

./group190/reference/d1jsca2-d1zpda3.fasta

./group190/reference/d1bfd_2-d1keka1.fasta

./group190/reference/d1bfd_3-d1keka2.fasta

./group190/reference/d1keka1-d1ovma3.fasta

./group191/reference/d1hyqa_-d1n25a_.fasta

./group191/reference/d1f60a3-d1fnna2.fasta

./group191/reference/d1hyqa_-d1mt0a_.fasta

./group191/reference/d1d2na_-d1g2912.fasta

./group191/reference/d1bg2__-d1d2na_.fasta

./group191/reference/d1bg2__-d1eg7a_.fasta

./group191/reference/d1cp2a_-d1g6ha_.fasta

./group191/reference/d1e32a2-d1mt0a_.fasta

./group191/reference/d1g2912-d1ofha_.fasta

./group191/reference/d1g2912-d1njfa_.fasta

./group192/reference/d1ga6a_-d1ic6a_.fasta

./group192/reference/d1ga6a_-d1ot5a2.fasta

./group192/reference/d1ga6a_-d1p8ja2.fasta

./group193/reference/d1c3pa_-d2ceva_.fasta

./group194/reference/d1l5aa1-d1l5aa2.fasta

./group194/reference/d1b5sa_-d1nocb_.fasta

./group194/reference/d1eaf__-d1l5aa1.fasta

./group194/reference/d1eaf__-d1nocb_.fasta

./group194/reference/d1l5aa2-d1nocb_.fasta

./group194/reference/d1eaf__-d1l5aa2.fasta

./group194/reference/d1l5aa1-d1nocb_.fasta

./group194/reference/d1b5sa_-d3cla__.fasta

./group194/reference/d1b5sa_-d1l5aa1.fasta

./group194/reference/d1l5aa2-d3cla__.fasta

./group195/reference/d1jf8a_-d1phr__.fasta

./group196/reference/d1d5ra2-d1eeoa_.fasta

./group196/reference/d1lara1-d1vhra_.fasta

./group196/reference/d1lyva_-d1mkp__.fasta

./group196/reference/d1eeoa_-d1lyva_.fasta

./group196/reference/d1eeoa_-d1vhra_.fasta

./group196/reference/d1eeoa_-d1fpza_.fasta

./group196/reference/d1lyva_-d1ohea1.fasta

./group196/reference/d1fpza_-d1lyva_.fasta

./group196/reference/d1fpza_-d1vhra_.fasta

./group196/reference/d1fpza_-d1ohea1.fasta

./group197/reference/d1hzma_-d1rhs_1.fasta

./group198/reference/d1ep7a_-d1m2da_.fasta

./group198/reference/d1f9ma_-d1hd2a_.fasta

./group198/reference/d1a8l_1-d1gh2a_.fasta

./group198/reference/d1eeja1-d1mek__.fasta

./group198/reference/d1a8l_1-d1quwa_.fasta

./group198/reference/d1hyua3-d1m2da_.fasta

./group198/reference/d1a8y_1-d1kte__.fasta

./group198/reference/d1hyua3-d1jfua_.fasta

./group198/reference/d1hd2a_-d1m2da_.fasta

./group198/reference/d1eeja1-d1knga_.fasta

./group199/reference/d1dtwb2-d1gpua3.fasta

./group199/reference/d1dtwb2-d1keka3.fasta

./group199/reference/d1dtwb2-d1l8aa3.fasta

./group199/reference/d1gpua3-d1keka3.fasta

./group199/reference/d1itza3-d1keka3.fasta

./group199/reference/d1keka3-d1l8aa3.fasta

./group199/reference/d1keka3-d1qgda3.fasta

./group201/reference/d1gyta1-d1lam_1.fasta

./group201/reference/d1hjza_-d1lam_1.fasta

./group202/reference/d1h4vb1-d1nj8a1.fasta

./group202/reference/d1h4vb1-d1qf6a1.fasta

./group202/reference/d1atia1-d1qe0a1.fasta

./group202/reference/d1qe0a1-d1qf6a1.fasta

./group202/reference/d1h4vb1-d1nj1a1.fasta

./group202/reference/d1kmma1-d1qf6a1.fasta

./group202/reference/d1hc7a1-d1qe0a1.fasta

./group202/reference/d1kmma1-d1nj1a1.fasta

./group202/reference/d1nj8a1-d1qe0a1.fasta

./group202/reference/d1kmma1-d1nj8a1.fasta

./group203/reference/d1j23a_-d2foka4.fasta

./group203/reference/d1ckqa_-d1vsra_.fasta

./group203/reference/d1f1za2-d1fiua_.fasta

./group203/reference/d1fiua_-d1j23a_.fasta

./group203/reference/d1fiua_-d1vsra_.fasta

./group203/reference/d1avqa_-d2foka4.fasta

./group203/reference/d1dmua_-d1vsra_.fasta

./group203/reference/d1m0da_-d2foka4.fasta

./group203/reference/d1cfr__-d1vsra_.fasta

./group203/reference/d1f1za2-d1j23a_.fasta

./group205/reference/d1huxa_-d1j6za2.fasta

./group205/reference/d1czan3-d1mwma2.fasta

./group205/reference/d1huxa_-d1ig8a1.fasta

./group205/reference/d1bdg_2-d1j6za2.fasta

./group205/reference/d1e4ft1-d1nbwa3.fasta

./group205/reference/d1ig8a1-d1j6za2.fasta

./group205/reference/d1bupa1-d1g99a2.fasta

./group205/reference/d1czan1-d1ig8a2.fasta

./group205/reference/d1bupa2-d1j6za2.fasta

./group205/reference/d1czan1-d1g99a1.fasta

./group206/reference/d1hjra_-d1io2a_.fasta

./group206/reference/d1hjra_-d1l3sa1.fasta

./group206/reference/d1i39a_-d1tgoa1.fasta

./group206/reference/d1ih7a1-d1io2a_.fasta

./group206/reference/d1qtma1-d1t7pa1.fasta

./group206/reference/d1l3sa1-d1qtma1.fasta

./group206/reference/d1qtma1-d1tgoa1.fasta

./group206/reference/d1l3sa1-d1tgoa1.fasta

./group206/reference/d1ih7a1-d1t7pa1.fasta

./group206/reference/d1jl1a_-d1qtma1.fasta

./group207/reference/d1dt9a1-d1jj2m_.fasta

./group207/reference/d1fjgk_-d1jj2m_.fasta

./group208/reference/d1b8oa_-d1k9sa_.fasta

./group208/reference/d1g2oa_-d1k9sa_.fasta

./group209/reference/d1gyta2-d1loka_.fasta

./group209/reference/d1h8la2-d1lam_2.fasta

./group209/reference/d1h8la2-d1loka_.fasta

./group209/reference/d1lam_2-d1loka_.fasta

./group209/reference/d1loka_-d1m4la_.fasta

./group210/reference/d1b0aa2-d1npya2.fasta

./group210/reference/d1b0aa2-d1nvta2.fasta

./group210/reference/d1a4ia2-d1lu9a2.fasta

./group210/reference/d1lu9a2-d1nvta2.fasta

./group210/reference/d1edza2-d1gtma2.fasta

./group210/reference/d1edza2-d1leha2.fasta

./group210/reference/d1hwxa2-d1nvta2.fasta

./group210/reference/d1a4ia2-d1nvta2.fasta

./group210/reference/d1a4ia2-d1bgva2.fasta

./group210/reference/d1edza2-d1npya2.fasta

./group211/reference/d1bif_2-d1nd6a_.fasta

./group211/reference/d1e58a_-d1nd6a_.fasta

./group211/reference/d1fzta_-d1nd6a_.fasta

./group211/reference/d1h2ea_-d1nd6a_.fasta

./group212/reference/d1dqna_-d1ecfa1.fasta

./group212/reference/d1dqna_-d1lh0a_.fasta

./group212/reference/d1l1qa_-d1lh0a_.fasta

./group212/reference/d1dqna_-d1l1qa_.fasta

./group212/reference/d1fsga_-d1l1qa_.fasta

./group212/reference/d1g2qa_-d1lh0a_.fasta

./group212/reference/d1bzya_-d1qb7a_.fasta

./group212/reference/d1bzya_-d1lh0a_.fasta

./group212/reference/d1fsga_-d1gph11.fasta

./group212/reference/d1fsga_-d1lh0a_.fasta

./group213/reference/d1atza_-d1m1xb2.fasta

./group213/reference/d1ijba_-d1m1xb2.fasta

./group213/reference/d1ijba_-d1mf7a_.fasta

./group213/reference/d1ijba_-d1qc5a_.fasta

./group213/reference/d1m1xb2-d1mf7a_.fasta

./group213/reference/d1m1xb2-d1mjna_.fasta

./group213/reference/d1m1xb2-d1qc5a_.fasta

./group214/reference/d1hnna_-d1nv8a_.fasta

./group214/reference/d1kywa2-d1nw3a_.fasta

./group214/reference/d1fp2a2-d1oria_.fasta

./group214/reference/d1jg1a_-d1yub__.fasta

./group214/reference/d1fp2a2-d1xvaa_.fasta

./group214/reference/d1fp2a2-d1qama_.fasta

./group214/reference/d1dl5a1-d1f3la_.fasta

./group214/reference/d1dl5a1-d1oria_.fasta

./group214/reference/d1jg1a_-d1jqea_.fasta

./group214/reference/d1af7_2-d1i1na_.fasta

./group215/reference/d1c7na_-d1j32a_.fasta

./group215/reference/d1tpla_-d2ay1a_.fasta

./group215/reference/d1c7na_-d1gtxa_.fasta

./group215/reference/d1e5ea_-d1elua_.fasta

./group215/reference/d1bs0a_-d1kl1a_.fasta

./group215/reference/d1j32a_-d1kl1a_.fasta

./group215/reference/d1ajsa_-d1jf9a_.fasta

./group215/reference/d1n8pa_-d3tata_.fasta

./group215/reference/d1qisa_-d1tpla_.fasta

./group215/reference/d1n8pa_-d2gsaa_.fasta

./group216/reference/d1h7ea_-d1hm9a2.fasta

./group216/reference/d1fxoa_-d1nf5b_.fasta

./group216/reference/d1h7ea_-d1nf5b_.fasta

./group216/reference/d1fxoa_-d1gx4a_.fasta

./group216/reference/d1h7ea_-d1hv9a2.fasta

./group216/reference/d1fxoa_-d1hm9a2.fasta

./group216/reference/d1hm9a2-d1i52a_.fasta

./group216/reference/d1fxoa_-d1i52a_.fasta

./group216/reference/d1hv9a2-d1i52a_.fasta

./group216/reference/d1e5ka_-d1fxoa_.fasta

./group217/reference/d1c4xa_-d1qlwa_.fasta

./group217/reference/d1ac5__-d1bn7a_.fasta

./group217/reference/d1ju3a2-d1ku0a_.fasta

./group217/reference/d1iz7a_-d1jjia_.fasta

./group217/reference/d1ek1a2-d1ex9a_.fasta

./group217/reference/d1l7aa_-d1tca__.fasta

./group217/reference/d1bu8a2-d1llfa_.fasta

./group217/reference/d1gkla_-d1lnsa3.fasta

./group217/reference/d1ex9a_-d1mtza_.fasta

./group217/reference/d1jjia_-d1lnsa3.fasta

./group219/reference/d1bx4a_-d1o14a_.fasta

./group219/reference/d1o14a_-d1rkd__.fasta

./group221/reference/d1e4bp_-d1gt7a_.fasta

./group221/reference/d1gt7a_-d1k0wa_.fasta

./group223/reference/d1cnza_-d1itwa_.fasta

./group223/reference/d1itwa_-d1lwda_.fasta

./group223/reference/d1itwa_-d1xaa__.fasta

./group224/reference/d1otha1-d1otha2.fasta

./group224/reference/d1js1x1-d1otha2.fasta

./group224/reference/d1duvg1-d1duvg2.fasta

./group224/reference/d1a1s_2-d1ml4a1.fasta

./group224/reference/d1ml4a1-d1otha2.fasta

./group224/reference/d1duvg1-d1otha2.fasta

./group224/reference/d1duvg2-d1js1x1.fasta

./group224/reference/d1ekxa2-d1js1x2.fasta

./group224/reference/d1duvg1-d1ml4a2.fasta

./group224/reference/d1ml4a1-d1ml4a2.fasta

./group225/reference/d1b74a1-d1b74a2.fasta

./group225/reference/d1b74a1-d1jfla2.fasta

./group225/reference/d1b74a2-d1jfla1.fasta

./group225/reference/d1b74a2-d1jfla2.fasta

./group226/reference/d1f2da_-d1j6na_.fasta

./group226/reference/d1f2da_-d1qopb_.fasta

./group226/reference/d1f2da_-d1tdj_1.fasta

./group226/reference/d1j0aa_-d1j6na_.fasta

./group226/reference/d1j0aa_-d1qopb_.fasta

./group226/reference/d1j0aa_-d1tdj_1.fasta

./group227/reference/d1iata_-d1moq__.fasta

./group229/reference/d1ad3a_-d1ez0a_.fasta

./group229/reference/d1ez0a_-d1ky8a_.fasta

./group230/reference/d1k2yx1-d1k2yx2.fasta

./group230/reference/d1k2yx1-d1kfia1.fasta

./group230/reference/d1k2yx2-d1k2yx3.fasta

./group230/reference/d1k2yx2-d1kfia1.fasta

./group230/reference/d1k2yx2-d3pmga1.fasta

./group230/reference/d1k2yx2-d3pmga3.fasta

./group230/reference/d1k2yx3-d3pmga1.fasta

./group230/reference/d1k2yx3-d3pmga2.fasta

./group230/reference/d1k2yx3-d3pmga3.fasta

./group232/reference/d1jixa_-d1l5wa_.fasta

./group234/reference/d1doza_-d1qgoa_.fasta

./group234/reference/d1lbqa_-d1qgoa_.fasta

./group235/reference/d1efdn_-d1miob_.fasta

./group235/reference/d1n2za_-d1toaa_.fasta

./group235/reference/d1m1na_-d1m1nb_.fasta

./group235/reference/d1m1na_-d1n2za_.fasta

./group235/reference/d1efdn_-d1m1na_.fasta

./group235/reference/d1efdn_-d1psza_.fasta

./group235/reference/d1miob_-d1toaa_.fasta

./group235/reference/d1efdn_-d1toaa_.fasta

./group235/reference/d1m1na_-d1toaa_.fasta

./group235/reference/d1efdn_-d1m1nb_.fasta

./group236/reference/d2dri__-d2liv__.fasta

./group236/reference/d2liv__-d8abp__.fasta

./group236/reference/d1pea__-d8abp__.fasta

./group236/reference/d1dp4a_-d1gca__.fasta

./group236/reference/d1byka_-d2dri__.fasta

./group236/reference/d1byka_-d1rpja_.fasta

./group236/reference/d1pea__-d2dri__.fasta

./group236/reference/d1rpja_-d2liv__.fasta

./group236/reference/d1byka_-d1gca__.fasta

./group236/reference/d1jx6a_-d1pea__.fasta

./group237/reference/d1amf__-d1pot__.fasta

./group237/reference/d1al3__-d1sbp__.fasta

./group237/reference/d1a8e__-d1a99a_.fasta

./group237/reference/d1a99a_-d3thia_.fasta

./group237/reference/d1sbp__-d3thia_.fasta

./group237/reference/d1jeta_-d3thia_.fasta

./group237/reference/d1i6aa_-d1lst__.fasta

./group237/reference/d1a99a_-d1amf__.fasta

./group237/reference/d1al3__-d1eu8a_.fasta

./group237/reference/d1a99a_-d1sbp__.fasta

./group238/reference/d1ek4a1-d1m3ka1.fasta

./group238/reference/d1ek4a2-d1mzja1.fasta

./group238/reference/d1ek4a2-d1m3ka2.fasta

./group238/reference/d1e5ma2-d1hzpa2.fasta

./group238/reference/d1e5ma2-d1m3ka1.fasta

./group238/reference/d1e5ma2-d1ox0a1.fasta

./group238/reference/d1hzpa2-d1m3ka1.fasta

./group238/reference/d1ek4a1-d1ox0a2.fasta

./group238/reference/d1m3ka1-d1ox0a1.fasta

./group238/reference/d1mzja1-d1mzja2.fasta

./group239/reference/d1aln_1-d1aln_2.fasta

./group239/reference/d1aln_1-d1uaqa_.fasta

./group239/reference/d1aln_2-d1uaqa_.fasta

./group240/reference/d1a2pa_-d1i0va_.fasta

./group240/reference/d1i0va_-d1lnia_.fasta

./group241/reference/d1k28a3-d3lzt__.fasta

./group241/reference/d1gd6a_-d1k28a3.fasta

./group241/reference/d1k28a3-d1qgia_.fasta

./group241/reference/d1lw9a_-d3lzt__.fasta

./group241/reference/d1qsaa2-d3lzt__.fasta

./group241/reference/d1chka_-d1dxja_.fasta

./group241/reference/d1chka_-d1lw9a_.fasta

./group241/reference/d1dxja_-d3lzt__.fasta

./group241/reference/d1qgia_-d3lzt__.fasta

./group241/reference/d153l__-d2eql__.fasta

./group242/reference/d1cvza_-d1qmya_.fasta

./group242/reference/d1cvza_-d1f13a4.fasta

./group242/reference/d1fh0a_-d2cb5a_.fasta

./group242/reference/d1gx3a_-d3gcb__.fasta

./group242/reference/d1me4a_-d1nbfa_.fasta

./group242/reference/d1avpa_-d1f13a4.fasta

./group242/reference/d1nbfa_-d7pcka_.fasta

./group242/reference/d1qmya_-d3gcb__.fasta

./group242/reference/d1iwda_-d1nbfa_.fasta

./group242/reference/d1avpa_-d3gcb__.fasta

./group243/reference/d1a73a_-d1ql0a_.fasta

./group243/reference/d1e7la2-d1fr2b_.fasta

./group243/reference/d1e7la2-d1ql0a_.fasta

./group243/reference/d1fr2b_-d1ql0a_.fasta

./group245/reference/d1el0a_-d1qg7a_.fasta

./group245/reference/d1doka_-d1qg7a_.fasta

./group245/reference/d1el0a_-d1f2la_.fasta

./group245/reference/d1m8aa_-d1qg7a_.fasta

./group245/reference/d1el0a_-d1tvxa_.fasta

./group245/reference/d1el0a_-d1m8aa_.fasta

./group245/reference/d1g2ta_-d1qg7a_.fasta

./group245/reference/d1g2ta_-d3il8__.fasta

./group245/reference/d1f2la_-d1tvxa_.fasta

./group245/reference/d1j9oa_-d1qg7a_.fasta

./group246/reference/d1bf4a_-d1dz1a_.fasta

./group246/reference/d1bf4a_-d1g6za_.fasta

./group246/reference/d1bf4a_-d1knaa_.fasta

./group246/reference/d1dz1a_-d1g6za_.fasta

./group247/reference/d1bb8__-d1d9na_.fasta

./group247/reference/d1bb8__-d1gcca_.fasta

./group247/reference/d1bb8__-d1kjka_.fasta

./group247/reference/d1d9na_-d1kjka_.fasta

./group247/reference/d1gcca_-d1kjka_.fasta

./group247/reference/d1kjka_-d1qk9a_.fasta

./group248/reference/d1jj2l_-d1jj2r_.fasta

./group248/reference/d1jj2l_-d1n88a_.fasta

./group249/reference/d1guqa2-d1kpf__.fasta

./group250/reference/d1h72c1-d1p42a1.fasta

./group250/reference/d1kkha1-d1p42a1.fasta

./group250/reference/d1dar_3-d1p42a2.fasta

./group250/reference/d1dar_3-d1n0ua3.fasta

./group250/reference/d1d6ta_-d1h72c1.fasta

./group250/reference/d1fjgi_-d1p42a2.fasta

./group250/reference/d1kija1-d1kkha1.fasta

./group250/reference/d1p42a2-d1ueka1.fasta

./group250/reference/d1b63a1-d1ueka1.fasta

./group250/reference/d1dar_3-d1pkp_1.fasta

./group251/reference/d1c1yb_-d1rlf__.fasta

./group251/reference/d1h4ra3-d1i42a_.fasta

./group251/reference/d1lfda_-d1mg8a_.fasta

./group251/reference/d1i42a_-d1l7ya_.fasta

./group251/reference/d1a5r__-d1m94a_.fasta

./group251/reference/d1euvb_-d1j8ca_.fasta

./group251/reference/d1h8ca_-d1rlf__.fasta

./group251/reference/d1l7ya_-d1lm8b_.fasta

./group251/reference/d1c1yb_-d1j8ca_.fasta

./group251/reference/d1lm8b_-d1m94a_.fasta

./group252/reference/d1c9fa_-d1ip9a_.fasta

./group252/reference/d1c9fa_-d1pqsa_.fasta

./group252/reference/d1d4ba_-d1ip9a_.fasta

./group252/reference/d1f2ri_-d1ip9a_.fasta

./group252/reference/d1ip9a_-d1pqsa_.fasta

./group253/reference/d1fm0d_-d1jsba_.fasta

./group254/reference/d1krha3-d1n62a2.fasta

./group254/reference/d1doi__-d1nekb2.fasta

./group254/reference/d1czpa_-d1fo4a2.fasta

./group254/reference/d1feha2-d1l5pa_.fasta

./group254/reference/d1fo4a2-d1qlab2.fasta

./group254/reference/d1feha2-d1nekb2.fasta

./group254/reference/d1jq4a_-d1put__.fasta

./group254/reference/d1fo4a2-d2pia_3.fasta

./group254/reference/d1kf6b2-d2pia_3.fasta

./group254/reference/d1doi__-d1n62a2.fasta

./group255/reference/d1bmlc3-d1qqra_.fasta

./group255/reference/d1qqra_-d2sak__.fasta

./group256/reference/d1esfa2-d1m4va2.fasta

./group256/reference/d1esfa2-d3tss_2.fasta

./group256/reference/d1et9a2-d1m4va2.fasta

./group256/reference/d1eu3a2-d1m4va2.fasta

./group257/reference/d1d5ta2-d1l9ea2.fasta

./group257/reference/d1an9a2-d1ju2a2.fasta

./group257/reference/d1gosa2-d1gpea2.fasta

./group257/reference/d1c0pa2-d1kdga2.fasta

./group257/reference/d1gpea2-d1mxta2.fasta

./group257/reference/d1k0ia2-d1mxta2.fasta

./group257/reference/d1c0pa2-d1l9ea2.fasta

./group257/reference/d1an9a2-d1gosa2.fasta

./group257/reference/d1d5ta2-d1gosa2.fasta

./group257/reference/d1kdga2-d1l9ea2.fasta

./group258/reference/d1mola_-d1stfi_.fasta

./group259/reference/d1ivwa2-d1ksia2.fasta

./group259/reference/d1ivwa2-d1ivwa3.fasta

./group259/reference/d1ivwa2-d1oaca3.fasta

./group259/reference/d1a2va2-d1oaca3.fasta

./group259/reference/d1ksia2-d1oaca3.fasta

./group259/reference/d1ivwa2-d1ksia3.fasta

./group259/reference/d1ksia2-d1ksia3.fasta

./group259/reference/d1ksia3-d1oaca2.fasta

./group259/reference/d1a2va2-d1a2va3.fasta

./group259/reference/d1a2va3-d1ksia3.fasta

./group260/reference/d1gy7a_-d1nwwa_.fasta

./group260/reference/d1idpa_-d1m98a2.fasta

./group260/reference/d1gy7a_-d1m98a2.fasta

./group260/reference/d1jkga_-d1nwwa_.fasta

./group260/reference/d1gy6a_-d1ocva_.fasta

./group260/reference/d1nwwa_-d1ocva_.fasta

./group260/reference/d1nwwa_-d1oh0a_.fasta

./group260/reference/d1idpa_-d1jkga_.fasta

./group260/reference/d1gy6a_-d1idpa_.fasta

./group260/reference/d1jkga_-d1mwxa1.fasta

./group261/reference/d1c16a2-d1lqva_.fasta

./group261/reference/d1hdma2-d1jfma_.fasta

./group261/reference/d1fnga2-d1zaga2.fasta

./group261/reference/d1fngb2-d1hdma2.fasta

./group261/reference/d1hyrc2-d1jfma_.fasta

./group261/reference/d1fngb2-d1gzqa2.fasta

./group261/reference/d1fngb2-d1k5na2.fasta

./group261/reference/d1fnga2-d1hdma2.fasta

./group261/reference/d1hdmb2-d1lqva_.fasta

./group261/reference/d1c16a2-d1iaka2.fasta

./group262/reference/d1fzya_-d1kppa_.fasta

./group262/reference/d1i7ka_-d1kppa_.fasta

./group262/reference/d1j7da_-d1kppa_.fasta

./group262/reference/d1jata_-d1kppa_.fasta

./group262/reference/d1kppa_-d1qcqa_.fasta

./group262/reference/d1kppa_-d2aak__.fasta

./group262/reference/d1kppa_-d2ucz__.fasta

./group263/reference/d1dzoa_-d1hpwa_.fasta

./group263/reference/d1dzoa_-d1oqva_.fasta

./group264/reference/d1j6ya_-d1l1pa_.fasta

./group264/reference/d1l1pa_-d1m5ya3.fasta

./group264/reference/d1jnsa_-d1kt1a3.fasta

./group264/reference/d1eq3a_-d1fd9a_.fasta

./group264/reference/d1m5ya3-d1pbk__.fasta

./group264/reference/d1bkf__-d1j6ya_.fasta

./group264/reference/d1jnsa_-d1l1pa_.fasta

./group264/reference/d1fd9a_-d1jnsa_.fasta

./group264/reference/d1jvwa_-d1l1pa_.fasta

./group264/reference/d1bkf__-d1m5ya3.fasta

./group265/reference/d1edqa3-d1jnda2.fasta

./group265/reference/d1goia3-d1kfwa2.fasta

./group265/reference/d1itxa2-d1jnda2.fasta

./group265/reference/d1jnda2-d1kfwa2.fasta

./group265/reference/d1jnda2-d1ll7a2.fasta

./group266/reference/d1cjxa1-d1f1ua1.fasta

./group266/reference/d1cjxa1-d1kw3b1.fasta

./group266/reference/d1f1ua1-d1f1ua2.fasta

./group266/reference/d1kw3b1-d1qtoa_.fasta

./group266/reference/d1ecsa_-d1mpya1.fasta

./group266/reference/d1mpya2-d1qtoa_.fasta

./group266/reference/d1cjxa1-d1ecsa_.fasta

./group266/reference/d1f1ua1-d1kw3b1.fasta

./group266/reference/d1ecsa_-d1mpya2.fasta

./group266/reference/d1cjxa1-d1kw3b2.fasta

./group267/reference/d1c8ua1-d1c8ua2.fasta

./group267/reference/d1c8ua2-d1iq6a_.fasta

./group267/reference/d1c8ua2-d1mkaa_.fasta

./group267/reference/d1iq6a_-d1lo7a_.fasta

./group267/reference/d1iq6a_-d1mkaa_.fasta

./group271/reference/d1buoa_-d1hv2a_.fasta

./group271/reference/d1buoa_-d1nn7a_.fasta

./group271/reference/d1fs1b2-d1nn7a_.fasta

./group274/reference/d1fjge2-d1qu6a2.fasta

./group274/reference/d1fjge2-d1qu6a1.fasta

./group274/reference/d1di2a_-d1pkp_2.fasta

./group274/reference/d1kn0a_-d1qu6a1.fasta

./group274/reference/d1kn0a_-d1o0wa2.fasta

./group274/reference/d1kn0a_-d1pkp_2.fasta

./group274/reference/d1fjge2-d1kn0a_.fasta

./group274/reference/d1di2a_-d1kn0a_.fasta

./group274/reference/d1pkp_2-d1stu__.fasta

./group274/reference/d1di2a_-d1fjge2.fasta

./group275/reference/d1dtja_-d1vig__.fasta

./group275/reference/d1dtja_-d2fmr__.fasta

./group275/reference/d1j4wa1-d2fmr__.fasta

./group275/reference/d1k1ga_-d1vig__.fasta

./group275/reference/d1k1ga_-d2fmr__.fasta

./group275/reference/d1vig__-d2fmr__.fasta

./group276/reference/d1egaa2-d1k0ra3.fasta

./group276/reference/d1fjgc1-d1k0ra3.fasta

./group276/reference/d1hh2p2-d1k0ra3.fasta

./group277/reference/d1jpma2-d1onea2.fasta

./group277/reference/d1onea2-d2chr_2.fasta

./group277/reference/d2chr_2-d2mnr_2.fasta

./group278/reference/d1h0hb_-d1keka5.fasta

./group278/reference/d1fxd__-d1hfel2.fasta

./group278/reference/d1h0hb_-d1jb0c_.fasta

./group278/reference/d1keka5-d7fd1a_.fasta

./group278/reference/d1fxra_-d1h0hb_.fasta

./group278/reference/d1h0hb_-d1h98a_.fasta

./group278/reference/d1h0hb_-d7fd1a_.fasta

./group278/reference/d1fxra_-d2fdn__.fasta

./group278/reference/d1feha3-d1iqza_.fasta

./group278/reference/d1h98a_-d1iqza_.fasta

./group279/reference/d1jqga2-d1scjb_.fasta

./group279/reference/d1kn6a_-d1kwma2.fasta

./group279/reference/d1kn6a_-d1scjb_.fasta

./group279/reference/d1kwma2-d1scjb_.fasta

./group280/reference/d1lq9a_-d1nwja_.fasta

./group281/reference/d1nh8a2-d1p1la_.fasta

./group281/reference/d1nh8a2-d2pii__.fasta

./group281/reference/d1p1la_-d2pii__.fasta

./group283/reference/d1koha2-d1l3ka1.fasta

./group283/reference/d1fj7a_-d1l3ka2.fasta

./group283/reference/d1b7fa2-d1iqta_.fasta

./group283/reference/d1owxa_-d2msta_.fasta

./group283/reference/d1oo0b_-d1qm9a2.fasta

./group283/reference/d1hd1a_-d1oo0b_.fasta

./group283/reference/d1fj7a_-d1koha2.fasta

./group283/reference/d1fxla1-d1u2fa_.fasta

./group283/reference/d1hd1a_-d2u1a__.fasta

./group283/reference/d1fjeb2-d1koha2.fasta

./group284/reference/d1dar_4-d1n0ua4.fasta

./group285/reference/d1aw0__-d1mwza_.fasta

./group285/reference/d1cc8a_-d1cpza_.fasta

./group285/reference/d1cc8a_-d1mwza_.fasta

./group285/reference/d1cpza_-d1fe0a_.fasta

./group285/reference/d1fe0a_-d1k0va_.fasta

./group285/reference/d1fe0a_-d1mwza_.fasta

./group286/reference/d1phza1-d1tdj_2.fasta

./group287/reference/d1h72c2-d1kkha2.fasta

./group287/reference/d1h72c2-d1kvka2.fasta

./group287/reference/d1h72c2-d1ueka2.fasta

./group287/reference/d1kkha2-d1ueka2.fasta

./group287/reference/d1kvka2-d1ueka2.fasta

./group289/reference/d1dj0a1-d1dj0a2.fasta

./group289/reference/d1dj0a2-d1k8wa4.fasta

./group290/reference/d1fjgd_-d1h3fa2.fasta

./group294/reference/d1dbfa_-d1onia_.fasta

./group294/reference/d1dbfa_-d1qd9a_.fasta

./group294/reference/d1onia_-d1ufya_.fasta

./group295/reference/d1fsz_2-d1tuba2.fasta

./group295/reference/d1fsz_2-d1tubb2.fasta

./group295/reference/d1ofua2-d1tuba2.fasta

./group295/reference/d1ofua2-d1tubb2.fasta

./group296/reference/d1ck9a_-d1e7ka_.fasta

./group296/reference/d1ck9a_-d1jj2f_.fasta

./group297/reference/d1dpta_-d1gyxa_.fasta

./group297/reference/d1dpta_-d1otfa_.fasta

./group297/reference/d1dpta_-d1otga_.fasta

./group297/reference/d1gd0a_-d1otfa_.fasta

./group297/reference/d1gd0a_-d1otga_.fasta

./group297/reference/d1gyxa_-d1otfa_.fasta

./group297/reference/d1gyxa_-d1otga_.fasta

./group297/reference/d1hfoa_-d1otfa_.fasta

./group297/reference/d1otfa_-d1otga_.fasta

./group298/reference/d1h6da2-d1lc0a2.fasta

./group298/reference/d1b7go2-d1ebfa2.fasta

./group298/reference/d1j5pa3-d1mb4a2.fasta

./group298/reference/d1b7go2-d1dih_2.fasta

./group298/reference/d1ebfa2-d1lc0a2.fasta

./group298/reference/d1b7go2-d1mb4a2.fasta

./group298/reference/d1e5qa2-d1f06a2.fasta

./group298/reference/d1b7go2-d1j5pa3.fasta

./group298/reference/d1cf2o2-d1f06a2.fasta

./group298/reference/d1f06a2-d1mb4a2.fasta

./group299/reference/d1feca3-d1mo9a3.fasta

./group299/reference/d1d7ya3-d1feca3.fasta

./group299/reference/d1nhp_3-d3grs_3.fasta

./group299/reference/d1ebda3-d1fcda3.fasta

./group299/reference/d1d7ya3-d1ebda3.fasta

./group299/reference/d1lvl_3-d1nhp_3.fasta

./group299/reference/d1d7ya3-d3lada3.fasta

./group299/reference/d1d7ya3-d1lvl_3.fasta

./group299/reference/d1d7ya3-d1nhp_3.fasta

./group299/reference/d1feca3-d1nhp_3.fasta

./group300/reference/d1fo4a4-d1jroa3.fasta

./group301/reference/d1f08a_-d1l2ma_.fasta

./group301/reference/d1l2ma_-d1m55a_.fasta

./group302/reference/d1f5va_-d1vfra_.fasta

./group303/reference/d1bqba_-d1buda_.fasta

./group303/reference/d1dmta_-d1kufa_.fasta

./group303/reference/d1hs6a3-d1kapp2.fasta

./group303/reference/d1bqba_-d1c7ka_.fasta

./group303/reference/d1k7ia2-d1keia_.fasta

./group303/reference/d1ezm__-d1gkda_.fasta

./group303/reference/d1dmta_-d1k7ia2.fasta

./group303/reference/d1ast__-d1kufa_.fasta

./group303/reference/d1g12a_-d1j7na2.fasta

./group303/reference/d1buda_-d1kapp2.fasta

./group305/reference/d1m61a_-d2cbla3.fasta

./group305/reference/d1opka2-d2cbla3.fasta

./group305/reference/d1a81a2-d2cbla3.fasta

./group305/reference/d1jyra_-d2cbla3.fasta

./group305/reference/d1lkka_-d2cbla3.fasta

./group305/reference/d1a81a1-d2cbla3.fasta

./group305/reference/d1ayaa_-d2cbla3.fasta

./group305/reference/d1fu6a_-d2cbla3.fasta

./group305/reference/d2cbla3-d2plda_.fasta

./group305/reference/d1d4ta_-d2cbla3.fasta

./group307/reference/d1dq3a3-d1dq3a4.fasta

./group307/reference/d1dq3a3-d1g9za_.fasta

./group307/reference/d1dq3a3-d1m5xa_.fasta

./group307/reference/d1dq3a4-d1g9za_.fasta

./group307/reference/d1dq3a4-d1m5xa_.fasta

./group308/reference/d1a8ra_-d1b66a_.fasta

./group308/reference/d1a8ra_-d1b9la_.fasta

./group308/reference/d1a8ra_-d1dhn__.fasta

./group308/reference/d1b66a_-d1b9la_.fasta

./group308/reference/d1b66a_-d1dhn__.fasta

./group308/reference/d1b66a_-d1uox_1.fasta

./group308/reference/d1b9la_-d1uox_1.fasta

./group308/reference/d1dhn__-d1uox_1.fasta

./group309/reference/d1jjca_-d1nj1a3.fasta

./group309/reference/d1b8aa2-d1nj1a3.fasta

./group309/reference/d1nj1a3-d1qf6a4.fasta

./group309/reference/d1eova2-d1jjca_.fasta

./group309/reference/d1jjca_-d1seta2.fasta

./group309/reference/d1jjcb5-d1seta2.fasta

./group309/reference/d12asa_-d1seta2.fasta

./group309/reference/d1b8aa2-d1jjcb5.fasta

./group309/reference/d12asa_-d1qf6a4.fasta

./group309/reference/d1eova2-d1qf6a4.fasta

./group310/reference/d1iyka1-d1lrza2.fasta

./group310/reference/d1cjwa_-d1iica2.fasta

./group310/reference/d1iyka2-d1qsta_.fasta

./group310/reference/d1fy7a_-d1qsta_.fasta

./group310/reference/d1cjwa_-d1qsta_.fasta

./group310/reference/d1cjwa_-d1ufha_.fasta

./group310/reference/d1fy7a_-d1iyka1.fasta

./group310/reference/d1lrza2-d1mk4a_.fasta

./group310/reference/d1cjwa_-d1iyka1.fasta

./group310/reference/d1cjwa_-d1m4ia_.fasta

./group311/reference/d1d0na3-d1d4xg_.fasta

./group311/reference/d1cfya_-d1hqz1_.fasta

./group311/reference/d1d0na5-d1d4xg_.fasta

./group311/reference/d1cfya_-d1jhwa3.fasta

./group311/reference/d1d4xg_-d1f7sa_.fasta

./group311/reference/d1d4xg_-d1hqz1_.fasta

./group311/reference/d1d0na3-d1d0na4.fasta

./group311/reference/d1jhwa3-d1m4ja_.fasta

./group311/reference/d1d0na4-d1hqz1_.fasta

./group311/reference/d1d0na4-d1f7sa_.fasta

./group313/reference/d1f5ma_-d1mc0a1.fasta

./group313/reference/d1f5ma_-d1mc0a2.fasta

./group313/reference/d1f5ma_-d1mkma2.fasta

./group313/reference/d1mc0a1-d1mkma2.fasta

./group314/reference/d1lswa_-d3pyp__.fasta

./group314/reference/d1bywa_-d1lswa_.fasta

./group314/reference/d1bywa_-d3pyp__.fasta

./group314/reference/d1ew0a_-d3pyp__.fasta

./group314/reference/d1bywa_-d1ll8a_.fasta

./group314/reference/d1jnua_-d3pyp__.fasta

./group314/reference/d1ll8a_-d1n9la_.fasta

./group314/reference/d1ll8a_-d3pyp__.fasta

./group314/reference/d1bywa_-d1ew0a_.fasta

./group314/reference/d1jnua_-d1ll8a_.fasta

./group315/reference/d1a3aa_-d1hynp_.fasta

./group315/reference/d1a6ja_-d1hynp_.fasta

./group316/reference/d1hzta_-d1jkna_.fasta

./group316/reference/d1hzta_-d1k2ea_.fasta

./group316/reference/d1jkna_-d1k2ea_.fasta

./group316/reference/d1k2ea_-d1ktga_.fasta

./group317/reference/d1b5ea_-d1f28a_.fasta

./group319/reference/d1b63a2-d1bxda_.fasta

./group319/reference/d1b63a2-d1ei1a2.fasta

./group319/reference/d1b63a2-d1i58a_.fasta

./group319/reference/d1b63a2-d1id0a_.fasta

./group319/reference/d1b63a2-d1kija2.fasta

./group319/reference/d1bxda_-d1byqa_.fasta

./group319/reference/d1bxda_-d1kija2.fasta

./group319/reference/d1byqa_-d1ei1a2.fasta

./group319/reference/d1byqa_-d1id0a_.fasta

./group319/reference/d1byqa_-d1kija2.fasta

./group321/reference/d1bwda_-d1g61a_.fasta

./group321/reference/d1bwda_-d1g62a_.fasta

./group321/reference/d1bwda_-d1h70a_.fasta

./group321/reference/d1h70a_-d1jdw__.fasta

./group324/reference/d1aisa2-d1mpga2.fasta

./group324/reference/d1ko9a2-d1mpga2.fasta

./group324/reference/d1ko9a2-d1ytba1.fasta

./group324/reference/d1mpga2-d1ytba1.fasta

./group325/reference/d1k2yx4-d1kfia4.fasta

./group325/reference/d1k2yx4-d3pmga4.fasta

./group326/reference/d1fm4a_-d1kcma_.fasta

./group326/reference/d1icxa_-d1kcma_.fasta

./group326/reference/d1jssa_-d1ln1a_.fasta

./group327/reference/d1mxa_2-d1mxa_3.fasta

./group327/reference/d1mxa_3-d1qm4a2.fasta

./group328/reference/d1b77a2-d2pola1.fasta

./group328/reference/d1b77a1-d2pola3.fasta

./group328/reference/d1plq_1-d2pola2.fasta

./group328/reference/d1iz5a1-d1iz5a2.fasta

./group328/reference/d1iz5a2-d2pola1.fasta

./group328/reference/d1dmla1-d2pola1.fasta

./group328/reference/d1iz5a1-d2pola3.fasta

./group328/reference/d1plq_2-d2pola3.fasta

./group328/reference/d1iz5a1-d1plq_2.fasta

./group328/reference/d1b77a1-d1plq_2.fasta

./group330/reference/d1ckv__-d1g10a_.fasta

./group330/reference/d1ckv__-d1hqi__.fasta

./group330/reference/d1g10a_-d1hqi__.fasta

./group331/reference/d1jj2e1-d1jj2e2.fasta

./group331/reference/d1jj2e1-d1rl6a2.fasta

./group331/reference/d1rl6a1-d1rl6a2.fasta

./group332/reference/d1gsa_2-d1i7na2.fasta

./group332/reference/d1ehia2-d1i7na2.fasta

./group332/reference/d1i7na2-d1jkjb2.fasta

./group332/reference/d1a9xa6-d1e4ea2.fasta

./group332/reference/d1a9xa5-d1iow_2.fasta

./group332/reference/d1a9xa5-d1jkjb2.fasta

./group332/reference/d1a9xa5-d1i7na2.fasta

./group332/reference/d1ehia2-d1gsa_2.fasta

./group332/reference/d1ehia2-d1jkjb2.fasta

./group332/reference/d1ehia2-d1kbla3.fasta

./group333/reference/d1csn__-d1p38__.fasta

./group333/reference/d1csn__-d1phk__.fasta

./group333/reference/d1j7la_-d1opja_.fasta

./group333/reference/d1j7la_-d1tkia_.fasta

./group333/reference/d1j7la_-d1m2ra_.fasta

./group333/reference/d1j7la_-d1k2pa_.fasta

./group333/reference/d1csn__-d1opja_.fasta

./group333/reference/d1apme_-d1j7la_.fasta

./group333/reference/d1csn__-d1tkia_.fasta

./group333/reference/d1j7la_-d1p38__.fasta

./group334/reference/d1fo4a6-d1uxy_1.fasta

./group334/reference/d1f0xa2-d1fo4a6.fasta

./group334/reference/d1f0xa2-d1uxy_1.fasta

./group334/reference/d1diqa2-d1uxy_1.fasta

./group334/reference/d1f0xa2-d1hska1.fasta

./group334/reference/d1diqa2-d1f0xa2.fasta

./group334/reference/d1i19a2-d1jroa4.fasta

./group334/reference/d1jroa4-d1uxy_1.fasta

./group334/reference/d1n62c2-d1uxy_1.fasta

./group334/reference/d1hska1-d1jroa4.fasta

./group335/reference/d1f7la_-d1qr0a1.fasta

./group335/reference/d1ftha_-d1qr0a1.fasta

./group336/reference/d1gdoa_-d1ryp2_.fasta

./group336/reference/d1ecfa2-d1iruk_.fasta

./group336/reference/d1ct9a2-d1ryph_.fasta

./group336/reference/d1ct9a2-d1ryp2_.fasta

./group336/reference/d1rypa_-d1rypj_.fasta

./group336/reference/d1iru1_-d1pmaa_.fasta

./group336/reference/d1gdoa_-d1j2pa_.fasta

./group336/reference/d1rypg_-d1rypk_.fasta

./group336/reference/d1pmaa_-d1rypk_.fasta

./group336/reference/d1gdoa_-d1rypa_.fasta

./group337/reference/d1a7ta_-d1smla_.fasta

./group337/reference/d1jjea_-d1k07a_.fasta

./group337/reference/d1m2xa_-d1qh5a_.fasta

./group337/reference/d1k07a_-d1qh5a_.fasta

./group337/reference/d1a7ta_-d1e5da2.fasta

./group337/reference/d1e5da2-d1jjea_.fasta

./group337/reference/d1jjea_-d1smla_.fasta

./group337/reference/d1e5da2-d1qh5a_.fasta

./group337/reference/d1k07a_-d1m2xa_.fasta

./group337/reference/d1qh5a_-d1smla_.fasta

./group338/reference/d1g5ba_-d1utea_.fasta

./group338/reference/d1nnwa_-d1utea_.fasta

./group338/reference/d1jk7a_-d4kbpa2.fasta

./group338/reference/d1g5ba_-d1nnwa_.fasta

./group338/reference/d1ii7a_-d4kbpa2.fasta

./group338/reference/d1utea_-d4kbpa2.fasta

./group338/reference/d1ii7a_-d1jk7a_.fasta

./group338/reference/d1jk7a_-d1utea_.fasta

./group338/reference/d1g5ba_-d1ii7a_.fasta

./group338/reference/d1g5ba_-d4kbpa2.fasta

./group339/reference/d1b8pa2-d1ez4a2.fasta

./group339/reference/d1b8pa2-d1i0za2.fasta

./group339/reference/d1b8pa2-d1ldna2.fasta

./group339/reference/d1ceqa2-d7mdha2.fasta

./group339/reference/d1b8pa2-d1ceqa2.fasta

./group339/reference/d1a5z_2-d1b8pa2.fasta

./group339/reference/d1i0za2-d5mdha2.fasta

./group339/reference/d1guya2-d5mdha2.fasta

./group339/reference/d1ldna2-d5mdha2.fasta

./group339/reference/d2cmd_2-d5mdha2.fasta

./group341/reference/d1gxya_-d1ikpa2.fasta

./group341/reference/d1gxya_-d1qs1a2.fasta

./group341/reference/d1f0la2-d1qs1a2.fasta

./group341/reference/d1giqa2-d1gxya_.fasta

./group341/reference/d1a26_2-d1qs1a1.fasta

./group341/reference/d1giqa1-d1gxya_.fasta

./group341/reference/d1a26_2-d1ikpa2.fasta

./group341/reference/d1giqa2-d1ikpa2.fasta

./group341/reference/d1f0la2-d1qs1a1.fasta

./group341/reference/d1giqa1-d1ikpa2.fasta

./group342/reference/d1chua3-d1jnra3.fasta

./group342/reference/d1jnra3-d1kf6a3.fasta

./group342/reference/d1jnra3-d1kssa3.fasta

./group342/reference/d1jnra3-d1neka3.fasta

./group342/reference/d1jnra3-d1qlaa3.fasta

./group342/reference/d1jnra3-d1qo8a3.fasta

./group343/reference/d1li1a1-d1tsg__.fasta

./group343/reference/d1kg0c_-d1tsg__.fasta

./group343/reference/d1kg0c_-d1tn3__.fasta

./group343/reference/d1dv8a_-d1tsg__.fasta

./group343/reference/d1cwva5-d1j34a_.fasta

./group343/reference/d1e87a_-d1h8ua_.fasta

./group343/reference/d1koe__-d1prtb2.fasta

./group343/reference/d1byfa_-d1tn3__.fasta

./group343/reference/d1jwib_-d1koe__.fasta

./group343/reference/d1li1a1-d1prea1.fasta

./group346/reference/d1ei5a3-d1g6aa_.fasta

./group346/reference/d1bsg__-d1qmea4.fasta

./group346/reference/d1es5a_-d1qmea4.fasta

./group346/reference/d1ei5a3-d1es5a_.fasta

./group346/reference/d1e25a_-d1qmea4.fasta

./group346/reference/d1es5a_-d1m40a_.fasta

./group346/reference/d1ghpa_-d1qmea4.fasta

./group346/reference/d1buea_-d1es5a_.fasta

./group346/reference/d1mfoa_-d1qmea4.fasta

./group346/reference/d1ci9a_-d1g6aa_.fasta

./group348/reference/d1is2a3-d1ivha2.fasta

./group348/reference/d1is2a3-d3mdda2.fasta

./group349/reference/d1g0ha_-d1inp__.fasta

./group349/reference/d1inp__-d1lbva_.fasta

./group350/reference/d1hhsa_-d1mml__.fasta

./group350/reference/d1hhsa_-d1tgoa2.fasta

./group350/reference/d1hhsa_-d1vrta2.fasta

./group350/reference/d1jiha_-d1l3sa2.fasta

./group350/reference/d1jiha_-d1tgoa2.fasta

./group350/reference/d1l3sa2-d1mml__.fasta

./group350/reference/d1mml__-d1tgoa2.fasta

./group350/reference/d1tgoa2-d1vrta2.fasta

./group351/reference/d1daaa_-d1ekfa_.fasta

./group352/reference/d1l6ra_-d1mh9a_.fasta

./group352/reference/d1mh9a_-d1zrn__.fasta

./group352/reference/d1k1ea_-d1o08a_.fasta

./group352/reference/d1feza_-d1o08a_.fasta

./group352/reference/d1mh9a_-d1qq5a_.fasta

./group352/reference/d1feza_-d1zrn__.fasta

./group352/reference/d1feza_-d1qq5a_.fasta

./group352/reference/d1feza_-d1k1ea_.fasta

./group352/reference/d1l6ra_-d1o08a_.fasta

./group352/reference/d1feza_-d1mh9a_.fasta

./group356/reference/d1ddba_-d1f16a_.fasta

./group356/reference/d1k3ka_-d1o0la_.fasta

./group358/reference/d1mm4a_-d1qj8a_.fasta

./group358/reference/d1p4ta_-d1qj8a_.fasta

./group359/reference/d1a0tp_-d1hxxa_.fasta

./group359/reference/d1a0tp_-d2por__.fasta

./group359/reference/d1by5a_-d1kmoa_.fasta

./group359/reference/d1by5a_-d3prn__.fasta

./group359/reference/d1hxxa_-d2mpra_.fasta

./group359/reference/d1hxxa_-d2por__.fasta

./group359/reference/d1hxxa_-d3prn__.fasta

./group359/reference/d1kmoa_-d2por__.fasta

./group359/reference/d2mpra_-d2por__.fasta

./group361/reference/d1dl0a_-d1lqra_.fasta

./group361/reference/d1cixa_-d1ju8a_.fasta

./group361/reference/d1ju8a_-d1qk7a_.fasta

./group361/reference/d1g9pa_-d1ju8a_.fasta

./group361/reference/d1i25a_-d1niya_.fasta

./group361/reference/d1dl0a_-d1kqha_.fasta

./group361/reference/d1ju8a_-d1lupa_.fasta

./group361/reference/d1eit__-d1ju8a_.fasta

./group361/reference/d1agg__-d1vtx__.fasta

./group361/reference/d1agg__-d1g9pa_.fasta

./group362/reference/d1gps__-d1npia_.fasta

./group362/reference/d1jkza_-d1qkya_.fasta

./group362/reference/d1c55a_-d1sis__.fasta

./group362/reference/d1aho__-d1mm0a_.fasta

./group362/reference/d1sis__-d1tsk__.fasta

./group362/reference/d1bcg__-d1jkza_.fasta

./group362/reference/d1i2ua_-d1mm0a_.fasta

./group362/reference/d1mm0a_-d1nrb__.fasta

./group362/reference/d1mm0a_-d1scy__.fasta

./group362/reference/d1bcg__-d1gps__.fasta

./group363/reference/d1h59b_-d1n8yc4.fasta

./group363/reference/d1h59b_-d1nqla3.fasta

./group363/reference/d1h59b_-d1nqla4.fasta

./group364/reference/d1imt_1-d1imt_2.fasta

./group365/reference/d1b9wa2-d1ob1c1.fasta

./group365/reference/d1b9wa2-d1tpg_1.fasta

./group365/reference/d1fjsl_-d1m1xb5.fasta

./group365/reference/d1klo_2-d1m1xb5.fasta

./group365/reference/d1cvua2-d1klil_.fasta

./group365/reference/d1ioxa_-d1ob1c1.fasta

./group365/reference/d1m1xb5-d1rfnb_.fasta

./group365/reference/d1b9wa1-d1ijqa2.fasta

./group365/reference/d1ijqa2-d1urk_1.fasta

./group365/reference/d1klo_2-d1xdtr_.fasta

./group366/reference/d1dec__-d1skz_1.fasta

./group366/reference/d1dec__-d1skz_2.fasta

./group366/reference/d1e0fi_-d1skz_2.fasta

./group367/reference/d1f94a_-d1m9za_.fasta

./group367/reference/d1jgka_-d1m9za_.fasta

./group367/reference/d1m9za_-d3ebx__.fasta

./group368/reference/d1bik_2-d1d0da_.fasta

./group368/reference/d1ktha_-d1tocr1.fasta

./group368/reference/d1bf0__-d1tocr2.fasta

./group368/reference/d1bik_1-d1d0da_.fasta

./group368/reference/d1d0da_-d1ktha_.fasta

./group368/reference/d1d0da_-d1jc6a_.fasta

./group368/reference/d1jc6a_-d1tocr1.fasta

./group368/reference/d1d0da_-d1tocr2.fasta

./group368/reference/d1aapa_-d1tocr1.fasta

./group368/reference/d1aapa_-d1d0da_.fasta

./group369/reference/d1ewsa_-d1h5oa_.fasta

./group369/reference/d1atx__-d1dfna_.fasta

./group369/reference/d1d6ba_-d1dfna_.fasta

./group369/reference/d1ewsa_-d1fd3a_.fasta

./group369/reference/d1b8wa_-d1ewsa_.fasta

./group369/reference/d1dfna_-d2bds__.fasta

./group369/reference/d1fd3a_-d1sh1__.fasta

./group369/reference/d1atx__-d1ewsa_.fasta

./group369/reference/d1e4ta_-d1ewsa_.fasta

./group369/reference/d1bnb__-d1ewsa_.fasta

./group370/reference/d1bhta1-d1hkya_.fasta

./group370/reference/d1bhta1-d1i8na_.fasta

./group370/reference/d1hkya_-d1i8na_.fasta

./group373/reference/d1h8pa1-d2hpqp_.fasta

./group373/reference/d1l6ja5-d2hpqp_.fasta

./group374/reference/d1ce3a_-d1pce__.fasta

./group374/reference/d1pce__-d4sgbi_.fasta

./group374/reference/d1ldtl_-d4sgbi_.fasta

./group374/reference/d1ce3a_-d1tbrr2.fasta

./group374/reference/d1ce3a_-d1tbrr1.fasta

./group374/reference/d1ce3a_-d1ldtl_.fasta

./group374/reference/d1iw4a_-d1nuba3.fasta

./group374/reference/d1iw4a_-d1tbrr1.fasta

./group374/reference/d1ce3a_-d1tgsi_.fasta

./group374/reference/d1iw4a_-d1pce__.fasta

./group376/reference/d1hcna_-d1hcnb_.fasta

./group376/reference/d1hcna_-d1jpya_.fasta

./group376/reference/d1jpya_-d1lxia_.fasta

./group376/reference/d1fltv_-d1lxia_.fasta

./group376/reference/d1aoca_-d2tgi__.fasta

./group376/reference/d1fltv_-d1hcnb_.fasta

./group376/reference/d1bnda_-d1hcnb_.fasta

./group376/reference/d1bnda_-d1lxia_.fasta

./group376/reference/d1aoca_-d1hcnb_.fasta

./group376/reference/d1fltv_-d1jpya_.fasta

./group377/reference/d1gkga2-d1quba5.fasta

./group377/reference/d1g40a4-d1gkna1.fasta

./group377/reference/d1gkna1-d1quba4.fasta

./group377/reference/d1g40a2-d1quba3.fasta

./group377/reference/d1g40a2-d1quba5.fasta

./group377/reference/d1quba1-d1quba5.fasta

./group377/reference/d1g40a1-d1ly2a2.fasta

./group377/reference/d1ly2a1-d1quba3.fasta

./group377/reference/d1ckla1-d1quba4.fasta

./group377/reference/d1gkna1-d1gpza2.fasta

./group380/reference/d1d4va3-d1exta3.fasta

./group380/reference/d1d4va3-d1jmab1.fasta

./group380/reference/d1exta2-d1oqdk_.fasta

./group380/reference/d1exta2-d1oqek_.fasta

./group380/reference/d1exta3-d1oqdk_.fasta

./group380/reference/d1exta3-d1oqek_.fasta

./group380/reference/d1jmab1-d1oqek_.fasta

./group381/reference/d1e88a3-d1tpg_2.fasta

./group383/reference/d1fu9a_-d2drpa1.fasta

./group383/reference/d1rmd_1-d1ubdc3.fasta

./group383/reference/d1fu9a_-d2glia4.fasta

./group383/reference/d1tf3a3-d2glia2.fasta

./group383/reference/d1bhi__-d2drpa1.fasta

./group383/reference/d1fu9a_-d1tf3a3.fasta

./group383/reference/d1yuja_-d2glia1.fasta

./group383/reference/d1tf3a1-d2glia1.fasta

./group383/reference/d2drpa1-d2glia2.fasta

./group383/reference/d1rmd_1-d1zfd__.fasta

./group385/reference/d1g47a1-d1jj2t_.fasta

./group385/reference/d1lata_-d1nypa1.fasta

./group385/reference/d1lata_-d3gata_.fasta

./group385/reference/d1k3xa3-d1lv3a_.fasta

./group385/reference/d1iml_2-d1nypa1.fasta

./group385/reference/d1nypa1-d3gata_.fasta

./group385/reference/d1a7i_2-d1nypa1.fasta

./group385/reference/d1d4ua2-d1m3va2.fasta

./group385/reference/d1k3xa3-d3gata_.fasta

./group385/reference/d1dsza_-d3gata_.fasta

./group388/reference/d1i50i1-d1qyp__.fasta

./group388/reference/d1d0qa_-d1qyp__.fasta

./group388/reference/d1d0qa_-d1i50i1.fasta

./group388/reference/d1i50i2-d1yua_1.fasta

./group388/reference/d1dl6a_-d1yua_1.fasta

./group388/reference/d1d0qa_-d1yua_2.fasta

./group388/reference/d1tfi__-d1yua_1.fasta

./group388/reference/d1d0qa_-d1dl6a_.fasta

./group388/reference/d1d0qa_-d1tfi__.fasta

./group388/reference/d1dl6a_-d1i50i1.fasta

./group389/reference/d1dxga_-d1ocrf_.fasta

./group389/reference/d1h7va_-d1ocrf_.fasta

./group389/reference/d1lkoa2-d1ocrf_.fasta

./group390/reference/d1jj2y_-d1jj2z_.fasta

./group390/reference/d1jj2z_-d1nvha_.fasta

./group391/reference/d1chc__-d1n87a_.fasta

./group391/reference/d1ldjb_-d1n87a_.fasta

./group391/reference/d1e4ua_-d1n87a_.fasta

./group391/reference/d1iyma_-d1n87a_.fasta

./group391/reference/d1fbva4-d1n87a_.fasta

./group391/reference/d1g25a_-d1ldjb_.fasta

./group391/reference/d1jm7b_-d1n87a_.fasta

./group391/reference/d1n87a_-d1rmd_2.fasta

./group391/reference/d1e4ua_-d1ldjb_.fasta

./group391/reference/d1g25a_-d1n87a_.fasta

./group392/reference/d1dmc__-d1m0ga_.fasta

./group392/reference/d1dmc__-d1qjka_.fasta

./group392/reference/d1fmya_-d1m0ga_.fasta

./group392/reference/d1fmya_-d1qjka_.fasta

./group392/reference/d1m0ga_-d1qjka_.fasta

./group393/reference/d1e53a_-d1kbea_.fasta

./group394/reference/d1dvpa2-d1mm2a_.fasta

./group394/reference/d1f62a_-d1vfya_.fasta

./group394/reference/d1mm2a_-d1vfya_.fasta

./group396/reference/d1ep3b1-d1ja1a1.fasta

./group396/reference/d1cqxa2-d1que_1.fasta

./group396/reference/d1a8p_1-d1i7pa1.fasta

./group396/reference/d1cqxa2-d1i8da1.fasta

./group396/reference/d2cnd_1-d2pia_1.fasta

./group396/reference/d1ddga1-d2cnd_1.fasta

./group396/reference/d1ja1a1-d2pia_1.fasta

./group396/reference/d1krha1-d2pia_1.fasta

./group396/reference/d1cqxa2-d1ddga1.fasta

./group396/reference/d1i7pa1-d2pia_1.fasta

./group397/reference/d1ezva1-d1l0la2.fasta

./group397/reference/d1ezvb2-d1hr6b2.fasta

./group397/reference/d1ezva1-d1l0lb2.fasta

./group397/reference/d1ezva1-d1hr6b2.fasta

./group397/reference/d1hr6b1-d1l0lb2.fasta

./group397/reference/d1ezvb2-d1hr6a2.fasta

./group397/reference/d1ezva1-d1ezvb1.fasta

./group397/reference/d1ezva1-d1hr6a1.fasta

./group397/reference/d1hr6b2-d1l0la1.fasta

./group397/reference/d1ezva2-d1ezvb1.fasta

./group398/reference/d1gjja1-d1jeia_.fasta

./group400/reference/d1h3za_-d1mhna_.fasta

./group400/reference/d1h3za_-d1oi1a1.fasta

./group400/reference/d1h3za_-d1oi1a2.fasta

./group400/reference/d1mhna_-d1oi1a1.fasta

./group401/reference/d1jdqa_-d1pava_.fasta

./group401/reference/d1je3a_-d1pava_.fasta

./group402/reference/d1gw5m2-d1gw5s_.fasta

./group402/reference/d1gw5m2-d1h8ma_.fasta

./group403/reference/d1hc7a3-d1nj1a2.fasta

./group404/reference/d1jeqa1-d1kcfa1.fasta

./group405/reference/d1ayl_2-d1khba2.fasta

./group405/reference/d1ii2a2-d1khba2.fasta

./group407/reference/d1hn0a4-d1jova_.fasta

./group407/reference/d1hn0a4-d1nsza_.fasta

./group407/reference/d1j0ma3-d1nsza_.fasta

./group407/reference/d1jova_-d1n7oa3.fasta

./group407/reference/d1jova_-d1nsza_.fasta

./group407/reference/d1jz8a4-d1nsza_.fasta

./group407/reference/d1n7oa3-d1nsza_.fasta

./group408/reference/d1edqa1-d1g0da1.fasta

./group408/reference/d1clc_2-d1edqa1.fasta

./group408/reference/d1eut_1-d1nepa_.fasta

./group408/reference/d1ji2a1-d1qfha2.fasta

./group408/reference/d1clc_2-d1qfha1.fasta

./group408/reference/d1jjua4-d1kmta_.fasta

./group408/reference/d1jmxa4-d1m7xa1.fasta

./group408/reference/d1edqa1-d1l9na1.fasta

./group408/reference/d1j0ha1-d1m7xa1.fasta

./group408/reference/d1g0da1-d1m7xa1.fasta

./group409/reference/d1f5aa4-d1jaja_.fasta

./group409/reference/d1f5aa4-d1knya2.fasta

./group409/reference/d1fa0a4-d1jaja_.fasta

./group409/reference/d1fa0a4-d1knya2.fasta

./group409/reference/d1jaja_-d1knya2.fasta

./group411/reference/d1k4cc_-d1orsc_.fasta

./group411/reference/d1orsc_-d1p7ba2.fasta

./group412/reference/d1kf6c_-d1kf6d_.fasta

./group412/reference/d1kf6c_-d1nekc_.fasta

./group412/reference/d1kf6c_-d1nekd_.fasta

./group412/reference/d1kf6d_-d1nekc_.fasta

./group412/reference/d1kf6d_-d1nekd_.fasta

./group412/reference/d1kf6d_-d1qlac_.fasta

./group412/reference/d1nekc_-d1nekd_.fasta

./group412/reference/d1nekc_-d1qlac_.fasta

./group414/reference/d1fftb2-d1m56b2.fasta

./group414/reference/d1fftb2-d1ocrb2.fasta

./group416/reference/d1k3xa2-d1k82a2.fasta

./group419/reference/d1f2na_-d1qqp3_.fasta

./group419/reference/d1b35a_-d1b35b_.fasta

./group419/reference/d1b35b_-d1k5ma_.fasta

./group419/reference/d1bev3_-d1bmv11.fasta

./group419/reference/d1bev3_-d1tmf1_.fasta

./group419/reference/d1smva_-d2mev3_.fasta

./group419/reference/d1b35b_-d1bev1_.fasta

./group419/reference/d1bev1_-d1ihma_.fasta

./group419/reference/d1b35a_-d1ny711.fasta

./group419/reference/d1ny722-d1pvc3_.fasta

./group420/reference/d1dnv__-d1lp3a_.fasta

./group420/reference/d1gff2_-d1lp3a_.fasta

./group421/reference/d1dzla_-d1vpsa_.fasta

./group423/reference/d1iw7f1-d1ku3a_.fasta

./group423/reference/d1ku3a_-d1l0oc_.fasta

./group423/reference/d1l0oc_-d1or7a1.fasta

./group424/reference/d1g8fa1-d1jhda1.fasta

./group424/reference/d1iq8a3-d1jhda1.fasta
