# Supplementary material for: PR2ALIGN: a stand-alone software program and a web-server for protein sequence alignment using weighted biochemical properties of amino acids
Source: BMC Res Notes. 2015 May 7;8:187. doi: 10.1186/s13104-015-1152-6 (PMC4477417; doi:10.1186/s13104-015-1152-6)
Supplement: Additional file 3: — SABmark SUP sequence pairs for 20-30% sequence identity range used to optimize property weights and gap penalties. Maximum of 10 randomly sampled pairs per superfamily. [file 13104_2015_1152_MOESM3_ESM.docx]

SABmark SUP sequence pairs for 20-30% sequence identity range used to optimize property weights and gap penalties.

Maximum of 10 randomly sampled pairs per superfamily.

./group1/reference/d1irdb_-d2lhb__.fasta

./group1/reference/d1d8ua_-d1mba__.fasta

./group1/reference/d1a6m__-d1jl7a_.fasta

./group1/reference/d1gcva_-d1it2a_.fasta

./group1/reference/d1a6m__-d2lhb__.fasta

./group1/reference/d1cg5a_-d1hlb__.fasta

./group1/reference/d1a6m__-d1irdb_.fasta

./group1/reference/d1irda_-d1it2a_.fasta

./group1/reference/d1irdb_-d1itha_.fasta

./group1/reference/d1irda_-d1jl7a_.fasta

./group2/reference/d1kf6b1-d1qlab1.fasta

./group2/reference/d1nekb1-d1qlab1.fasta

./group3/reference/d1fafa_-d1gh6a_.fasta

./group3/reference/d1fafa_-d1xbl__.fasta

./group3/reference/d1fpoa1-d1hdj__.fasta

./group3/reference/d1fpoa1-d1xbl__.fasta

./group3/reference/d1gh6a_-d1hdj__.fasta

./group3/reference/d1gh6a_-d1xbl__.fasta

./group4/reference/d1ivsa1-d1seta1.fasta

./group5/reference/d1ap6a1-d1coja1.fasta

./group5/reference/d1coja1-d1gv3a1.fasta

./group5/reference/d1coja1-d1ix9a1.fasta

./group6/reference/d1gu2a_-d1ql3a_.fasta

./group6/reference/d1dw0a_-d1e29a_.fasta

./group6/reference/d1f1ca_-d1nira1.fasta

./group6/reference/d1c52__-d3c2c__.fasta

./group6/reference/d1cxc__-d1dw0a_.fasta

./group6/reference/d1dw0a_-d1qn2a_.fasta

./group6/reference/d1qksa1-d1qn2a_.fasta

./group6/reference/d1hroa_-d1qksa1.fasta

./group6/reference/d1qksa1-d1ql3a_.fasta

./group6/reference/d1gu2a_-d1qksa1.fasta

./group7/reference/d1d5ya2-d1k78a2.fasta

./group7/reference/d1bw5__-d1e3oc1.fasta

./group7/reference/d1bl0a2-d1pb6a1.fasta

./group7/reference/d1b72b_-d1jgga_.fasta

./group7/reference/d1bw5__-d1pb6a1.fasta

./group7/reference/d1hlva1-d1iufa1.fasta

./group7/reference/d1b72b_-d1e3oc1.fasta

./group7/reference/d1iufa2-d1pb6a1.fasta

./group7/reference/d1hlva1-d1k78a2.fasta

./group7/reference/d1pb6a1-d2tct_1.fasta

./group9/reference/d1c20a_-d1ig6a_.fasta

./group9/reference/d1c20a_-d1kkxa_.fasta

./group9/reference/d1ig6a_-d1kkxa_.fasta

./group10/reference/d1puee_-d2irfg_.fasta

./group10/reference/d1fp1d1-d1fp2a1.fasta

./group10/reference/d1d5va_-d1fp2a1.fasta

./group10/reference/d1hks__-d1md0a_.fasta

./group10/reference/d1p4xa1-d1p4xa2.fasta

./group10/reference/d1p4xa2-d1puee_.fasta

./group10/reference/d1fp1d1-d1hks__.fasta

./group10/reference/d1fp1d1-d1jgsa_.fasta

./group10/reference/d1e17a_-d1repc2.fasta

./group10/reference/d1f1za1-d1hks__.fasta

./group11/reference/d1a04a1-d1p4wa_.fasta

./group12/reference/d1f4ia_-d1ifya_.fasta

./group13/reference/d1ee8a1-d1k3xa1.fasta

./group13/reference/d1fjgm_-d1k82a1.fasta

./group13/reference/d1k82a1-d1mu5a1.fasta

./group13/reference/d1l1za1-d1mu5a1.fasta

./group13/reference/d1k3xa1-d1l1za1.fasta

./group13/reference/d1ee8a1-d1fjgm_.fasta

./group13/reference/d1fjgm_-d1k3xa1.fasta

./group13/reference/d1fjgm_-d1nnja1.fasta

./group13/reference/d1k3xa1-d1nnja1.fasta

./group13/reference/d1ee8a1-d1mu5a1.fasta

./group14/reference/d1j9ia_-d1jbga_.fasta

./group15/reference/d1cuna1-d1quua2.fasta

./group15/reference/d1cuna2-d1quua1.fasta

./group16/reference/d1chua1-d1jnra1.fasta

./group16/reference/d1chua1-d1kf6a1.fasta

./group16/reference/d1chua1-d1neka1.fasta

./group16/reference/d1chua1-d1qlaa1.fasta

./group16/reference/d1jnra1-d1kf6a1.fasta

./group16/reference/d1jnra1-d1neka1.fasta

./group16/reference/d1jnra1-d1qlaa1.fasta

./group18/reference/d1erd__-d1erp__.fasta

./group19/reference/d1gg3a1-d1h4ra1.fasta

./group19/reference/d1gg3a1-d1mixa1.fasta

./group19/reference/d1h4ra1-d1mixa1.fasta

./group21/reference/d1hsm__-d1i11a_.fasta

./group21/reference/d1lwma_-d2lefa_.fasta

./group21/reference/d1ckta_-d1qrva_.fasta

./group21/reference/d1k99a_-d1lwma_.fasta

./group21/reference/d1i11a_-d1qrva_.fasta

./group21/reference/d1hsm__-d1k99a_.fasta

./group21/reference/d1ckta_-d1hsm__.fasta

./group21/reference/d1k99a_-d2lefa_.fasta

./group21/reference/d1qrva_-d2lefa_.fasta

./group21/reference/d1ckta_-d1k99a_.fasta

./group22/reference/d1b67a_-d1kx5b_.fasta

./group22/reference/d1kx5c_-d1n1jb_.fasta

./group22/reference/d1bh9b_-d1kx5c_.fasta

./group22/reference/d1bh9b_-d1jfib_.fasta

./group22/reference/d1kx5c_-d1kx5d_.fasta

./group22/reference/d1b67a_-d1kx5d_.fasta

./group22/reference/d1b67a_-d1bh9b_.fasta

./group22/reference/d1kx5b_-d1n1jb_.fasta

./group22/reference/d1kx5d_-d1n1ja_.fasta

./group22/reference/d1kx5a_-d1kx5d_.fasta

./group23/reference/d1bbha_-d1cpq__.fasta

./group23/reference/d1cpq__-d2ccya_.fasta

./group23/reference/d1cpq__-d256ba_.fasta

./group23/reference/d1jafa_-d2ccya_.fasta

./group23/reference/d1bbha_-d1jafa_.fasta

./group23/reference/d1bbha_-d2ccya_.fasta

./group23/reference/d1bbha_-d1gqaa_.fasta

./group23/reference/d1mqva_-d2ccya_.fasta

./group23/reference/d1bbha_-d256ba_.fasta

./group23/reference/d1cpq__-d1jafa_.fasta

./group25/reference/d1is2a1-d1ivha1.fasta

./group25/reference/d1is2a1-d1jqia1.fasta

./group26/reference/d1afra_-d1nfva_.fasta

./group26/reference/d1jiga_-d1o9ra_.fasta

./group26/reference/d1h0oa_-d1kgna_.fasta

./group26/reference/d1o9ra_-d1qgha_.fasta

./group26/reference/d1h0oa_-d1mxra_.fasta

./group26/reference/d1afra_-d1euma_.fasta

./group26/reference/d1ji4a_-d1o9ra_.fasta

./group26/reference/d1afra_-d1lkoa1.fasta

./group26/reference/d1lkoa1-d1nfva_.fasta

./group26/reference/d1euma_-d1rcd__.fasta

./group27/reference/d1lqsl_-d2ilk__.fasta

./group27/reference/d1m4ra_-d2ilk__.fasta

./group28/reference/d1a8h_1-d1ivsa2.fasta

./group28/reference/d1f4la1-d1ivsa2.fasta

./group28/reference/d1ffya1-d1ile_1.fasta

./group28/reference/d1ffya1-d1ivsa2.fasta

./group28/reference/d1ile_1-d1ivsa2.fasta

./group29/reference/d1af8__-d1klpa_.fasta

./group29/reference/d1dnya_-d1l0ia_.fasta

./group29/reference/d1dv5a_-d1l0ia_.fasta

./group30/reference/d1d1da1-d1qrjb1.fasta

./group31/reference/d1fts_1-d1ls1a1.fasta

./group31/reference/d1j8mf1-d1ls1a1.fasta

./group32/reference/d1eqfa1-d1f68a_.fasta

./group32/reference/d1eqfa2-d1jspb_.fasta

./group32/reference/d1f68a_-d1jspb_.fasta

./group33/reference/d1adr__-d1b0na2.fasta

./group33/reference/d1adr__-d1lmb3_.fasta

./group33/reference/d1e3oc2-d1efaa1.fasta

./group33/reference/d1adr__-d1d1la_.fasta

./group33/reference/d1lmb3_-d1zug__.fasta

./group33/reference/d1ic8a2-d1lmb3_.fasta

./group33/reference/d1d1la_-d1zug__.fasta

./group33/reference/d1d1la_-d1vpwa1.fasta

./group33/reference/d1ic8a2-d1r69__.fasta

./group33/reference/d1e3oc2-d1r69__.fasta

./group34/reference/d1a0aa_-d1mdya_.fasta

./group34/reference/d1a0aa_-d1nkpb_.fasta

./group34/reference/d1a0aa_-d1nlwa_.fasta

./group34/reference/d1am9a_-d1nlwa_.fasta

./group34/reference/d1mdya_-d1nkpb_.fasta

./group34/reference/d1mdya_-d1nlwa_.fasta

./group35/reference/d1rro__-d2scpa_.fasta

./group35/reference/d1dgua_-d5pal__.fasta

./group35/reference/d1el4a_-d1sra__.fasta

./group35/reference/d1dgua_-d1f8ha_.fasta

./group35/reference/d1exra_-d2scpa_.fasta

./group35/reference/d1rro__-d1sra__.fasta

./group35/reference/d1el4a_-d1ncx__.fasta

./group35/reference/d1exra_-d1psra_.fasta

./group35/reference/d1exra_-d1rro__.fasta

./group35/reference/d1auib_-d1m31a_.fasta

./group36/reference/d1aoa_1-d1aoa_2.fasta

./group36/reference/d1aoa_1-d1h67a_.fasta

./group36/reference/d1aoa_2-d1bhda_.fasta

./group36/reference/d1aoa_2-d1mb8a1.fasta

./group36/reference/d1aoa_2-d1mb8a2.fasta

./group36/reference/d1h67a_-d1mb8a1.fasta

./group37/reference/d1cmba_-d1mnta_.fasta

./group38/reference/d1f2ea1-d1k3ya1.fasta

./group38/reference/d1fw1a1-d1jlva1.fasta

./group38/reference/d1glqa1-d1oe8a1.fasta

./group38/reference/d1f2ea1-d2gsq_1.fasta

./group38/reference/d1aw9_1-d1jlva1.fasta

./group38/reference/d1jlva1-d1ljra1.fasta

./group38/reference/d1axda1-d1oe8a1.fasta

./group38/reference/d1oe8a1-d2gsq_1.fasta

./group38/reference/d1f2ea1-d1g7oa1.fasta

./group38/reference/d1axda1-d1k3ya1.fasta

./group39/reference/d1brwa1-d1khda1.fasta

./group39/reference/d1o17a1-d2tpt_1.fasta

./group41/reference/d1fk5a_-d1l6ha_.fasta

./group42/reference/d1dp3a_-d1exea_.fasta

./group42/reference/d1exea_-d1owfa_.fasta

./group42/reference/d1exea_-d1owfb_.fasta

./group42/reference/d1owfa_-d1owfb_.fasta

./group44/reference/d1b4fa_-d1oxja1.fasta

./group46/reference/d1a77_1-d1bgxt1.fasta

./group46/reference/d1a77_1-d1xo1a1.fasta

./group46/reference/d1b43a1-d1bgxt1.fasta

./group46/reference/d1b43a1-d1xo1a1.fasta

./group46/reference/d1bgxt1-d1xo1a1.fasta

./group46/reference/d1tfr_1-d1xo1a1.fasta

./group48/reference/d1l9la_-d1n69a_.fasta

./group49/reference/d1g5na_-d1n00a_.fasta

./group51/reference/d1m9fc_-d2eiaa2.fasta

./group52/reference/d1aisb1-d1aisb2.fasta

./group52/reference/d1aisb2-d1vola1.fasta

./group52/reference/d1bu2a1-d1f5qb1.fasta

./group52/reference/d1bu2a1-d1vin_1.fasta

./group52/reference/d1f5qb1-d1vin_1.fasta

./group52/reference/d1f5qb2-d1vola1.fasta

./group52/reference/d1jkw_1-d1vin_1.fasta

./group53/reference/d1a1w__-d1n3ka_.fasta

./group53/reference/d1d2za_-d1d2zb_.fasta

./group53/reference/d1d2za_-d1ngr__.fasta

./group53/reference/d1ddf__-d1fada_.fasta

./group53/reference/d1dgna_-d3crd__.fasta

./group53/reference/d1dgna_-d3ygsp_.fasta

./group53/reference/d1fada_-d1icha_.fasta

./group53/reference/d1fada_-d1ngr__.fasta

./group53/reference/d1icha_-d1ngr__.fasta

./group53/reference/d3crd__-d3ygsp_.fasta

./group55/reference/d1by1a_-d1f5xa_.fasta

./group55/reference/d1by1a_-d1ki1b1.fasta

./group55/reference/d1f5xa_-d1ki1b1.fasta

./group56/reference/d1dk8a_-d1omwa1.fasta

./group57/reference/d1mn2__-d1mwva1.fasta

./group57/reference/d1aru__-d1bgp__.fasta

./group57/reference/d1mn2__-d1oafa_.fasta

./group57/reference/d1bgp__-d1llp__.fasta

./group57/reference/d1aru__-d1oafa_.fasta

./group57/reference/d1mwva2-d1oafa_.fasta

./group57/reference/d1llp__-d1oafa_.fasta

./group57/reference/d1jdra_-d1mn2__.fasta

./group57/reference/d1aru__-d1mwva2.fasta

./group57/reference/d1bgp__-d1jdra_.fasta

./group58/reference/d1nkua_-d1orna_.fasta

./group58/reference/d1ko9a1-d1mpga1.fasta

./group58/reference/d1keaa_-d1ko9a1.fasta

./group58/reference/d1keaa_-d1orna_.fasta

./group58/reference/d1keaa_-d1ngna_.fasta

./group58/reference/d1keaa_-d1mun__.fasta

./group58/reference/d1mun__-d2abk__.fasta

./group58/reference/d1keaa_-d2abk__.fasta

./group58/reference/d1ko9a1-d2abk__.fasta

./group58/reference/d1mun__-d1ngna_.fasta

./group60/reference/d1dlja1-d1mv8a1.fasta

./group60/reference/d1mv8a1-d1n1ea1.fasta

./group60/reference/d1pgja1-d2pgd_1.fasta

./group61/reference/d1clc_1-d1g87a1.fasta

./group61/reference/d1clc_1-d1ks8a_.fasta

./group62/reference/d1cb8a1-d1n7oa1.fasta

./group62/reference/d1j0ma1-d1n7oa1.fasta

./group64/reference/d1a59__-d1csh__.fasta

./group64/reference/d1aj8a_-d1csh__.fasta

./group64/reference/d1csh__-d1ioma_.fasta

./group64/reference/d1csh__-d1k3pa_.fasta

./group64/reference/d1csh__-d1o7xa_.fasta

./group65/reference/d1cpt__-d1e9xa_.fasta

./group65/reference/d1jipa_-d1n6ba_.fasta

./group65/reference/d1n6ba_-d1n97a_.fasta

./group65/reference/d1e9xa_-d1n6ba_.fasta

./group65/reference/d1cpt__-d1dz4a_.fasta

./group65/reference/d1dz4a_-d1n6ba_.fasta

./group65/reference/d1io7a_-d1jfba_.fasta

./group65/reference/d1e9xa_-d1jfba_.fasta

./group65/reference/d1dz4a_-d1io7a_.fasta

./group65/reference/d1dz4a_-d1jipa_.fasta

./group67/reference/d1pbwa_-d1tx4a_.fasta

./group67/reference/d1tx4a_-d1wer__.fasta

./group68/reference/d1b3ua_-d1h6ka1.fasta

./group69/reference/d1ihba_-d1myo__.fasta

./group69/reference/d1awcb_-d1ycsb1.fasta

./group69/reference/d1ihba_-d1k1aa_.fasta

./group69/reference/d1awcb_-d1bi7b_.fasta

./group69/reference/d1awcb_-d1dcqa1.fasta

./group69/reference/d1k1aa_-d1myo__.fasta

./group69/reference/d1ihba_-d1ycsb1.fasta

./group69/reference/d1bi7b_-d1myo__.fasta

./group69/reference/d1myo__-d1ycsb1.fasta

./group69/reference/d1bd8__-d1ycsb1.fasta

./group70/reference/d1a17__-d1ihga1.fasta

./group70/reference/d1a17__-d1kt1a1.fasta

./group70/reference/d1elra_-d1hh8a_.fasta

./group70/reference/d1elra_-d1kt1a1.fasta

./group70/reference/d1elwa_-d1kt1a1.fasta

./group70/reference/d1hh8a_-d1ihga1.fasta

./group70/reference/d1ihga1-d1kt1a1.fasta

./group71/reference/d1elka_-d1eyha_.fasta

./group72/reference/d1fcya_-d1pk5a_.fasta

./group72/reference/d1pdua_-d1pk5a_.fasta

./group72/reference/d1fcya_-d2prga_.fasta

./group72/reference/d1ie9a_-d1pdua_.fasta

./group72/reference/d1n83a_-d2prga_.fasta

./group72/reference/d1a28a_-d1pdua_.fasta

./group72/reference/d1a28a_-d1kv6a_.fasta

./group72/reference/d1a28a_-d1pk5a_.fasta

./group72/reference/d1ie9a_-d1kv6a_.fasta

./group72/reference/d1ie9a_-d2prga_.fasta

./group74/reference/d1kxpd2-d1n5ua1.fasta

./group74/reference/d1kxpd2-d1n5ua2.fasta

./group74/reference/d1kxpd3-d1n5ua2.fasta

./group74/reference/d1kxpd3-d1n5ua3.fasta

./group75/reference/d1fura_-d1hy0a_.fasta

./group75/reference/d1hy0a_-d1j3ua_.fasta

./group76/reference/d1g4ia_-d1lwba_.fasta

./group77/reference/d1fs7a_-d1ft5a_.fasta

./group77/reference/d1m1qa_-d2ctha_.fasta

./group77/reference/d1kssa1-d3caoa_.fasta

./group77/reference/d1eysc_-d1m1qa_.fasta

./group77/reference/d1gu6a_-d2ctha_.fasta

./group77/reference/d1qo8a1-d1wad__.fasta

./group77/reference/d1qo8a1-d3caoa_.fasta

./group77/reference/d19hca_-d1wad__.fasta

./group77/reference/d1ft5a_-d1gu6a_.fasta

./group77/reference/d1ft5a_-d2cy3__.fasta

./group78/reference/d1ktke1-d1ncwh1.fasta

./group78/reference/d1b88a_-d1g9mh1.fasta

./group78/reference/d1dqta_-d1h5ba_.fasta

./group78/reference/d1g9mh1-d1hxmb1.fasta

./group78/reference/d1mfa_2-d2rhe__.fasta

./group78/reference/d1b88a_-d1fo0b_.fasta

./group78/reference/d1h5ba_-d1mqkh_.fasta

./group78/reference/d1fo0b_-d1h5ba_.fasta

./group78/reference/d1fo0a_-d1hxmb1.fasta

./group78/reference/d1dqta_-d1tvda_.fasta

./group79/reference/d1axib2-d1eerb2.fasta

./group79/reference/d1n26a2-d2fnba_.fasta

./group79/reference/d1bqua1-d1n26a2.fasta

./group79/reference/d1cd9b1-d1n26a2.fasta

./group79/reference/d1eerb2-d1f6fb1.fasta

./group79/reference/d1bpv__-d2fnba_.fasta

./group79/reference/d1eerb2-d1lqsr1.fasta

./group79/reference/d1fnf_1-d2fnba_.fasta

./group79/reference/d1cfb_2-d1lwra_.fasta

./group79/reference/d1axib2-d1qg3a2.fasta

./group80/reference/d1bhga1-d1jz8a1.fasta

./group81/reference/d1f13a2-d1kv3a2.fasta

./group81/reference/d1f13a3-d1kv3a2.fasta

./group81/reference/d1f13a3-d1kv3a3.fasta

./group81/reference/d1f13a3-d1l9na3.fasta

./group81/reference/d1g0da3-d1l9na3.fasta

./group81/reference/d1kv3a2-d1l9na2.fasta

./group81/reference/d1kv3a3-d1l9na3.fasta

./group82/reference/d1edha2-d1l3wa3.fasta

./group82/reference/d1l3wa3-d1l3wa4.fasta

./group82/reference/d1l3wa3-d1l3wa5.fasta

./group84/reference/d1do5a_-d1eso__.fasta

./group84/reference/d1do5a_-d1oala_.fasta

./group86/reference/d1cwva2-d1cwva3.fasta

./group86/reference/d1cwva4-d1f00i2.fasta

./group87/reference/d1e5ba_-d1exh__.fasta

./group87/reference/d1exh__-d1g43a_.fasta

./group87/reference/d1g43a_-d1tf4a2.fasta

./group87/reference/d1nbca_-d1tf4a2.fasta

./group89/reference/d1a02n2-d1a3qa2.fasta

./group89/reference/d1a02n2-d1bvoa_.fasta

./group89/reference/d1a3qa2-d1imhc2.fasta

./group89/reference/d1bvoa_-d1imhc2.fasta

./group90/reference/d1acz__-d1j18a1.fasta

./group91/reference/d1dmha_-d1eo9a_.fasta

./group92/reference/d1kbva2-d1kcw_5.fasta

./group92/reference/d1aoza2-d1gska2.fasta

./group92/reference/d1e30a_-d1kbva1.fasta

./group92/reference/d1gw0a1-d1oe1a2.fasta

./group92/reference/d1gw0a3-d1kcw_2.fasta

./group92/reference/d1aoza3-d1kcw_6.fasta

./group92/reference/d1kbva1-d1oe1a2.fasta

./group92/reference/d1fwxa1-d1m56b1.fasta

./group92/reference/d1gw0a1-d1kv7a1.fasta

./group92/reference/d1hfua3-d1kcw_6.fasta

./group95/reference/d1f53a_-d1g6ea_.fasta

./group99/reference/d1bhga2-d1jhja_.fasta

./group99/reference/d1bhga2-d1jz8a3.fasta

./group99/reference/d1cx1a_-d1guia_.fasta

./group99/reference/d1cx1a_-d1k42a_.fasta

./group99/reference/d1d7pm_-d1eut_2.fasta

./group99/reference/d1eut_2-d1kexa_.fasta

./group99/reference/d1gu3a_-d1guia_.fasta

./group99/reference/d1gu3a_-d1k42a_.fasta

./group99/reference/d1guia_-d1k42a_.fasta

./group99/reference/d1k12a_-d1kexa_.fasta

./group101/reference/d1h7za_-d1kkea1.fasta

./group101/reference/d1kaca_-d1kkea1.fasta

./group101/reference/d1kkea1-d1qhva_.fasta

./group102/reference/d1aly__-d1kxga_.fasta

./group102/reference/d1aly__-d1tnra_.fasta

./group102/reference/d1aly__-d2tnfa_.fasta

./group102/reference/d1kxga_-d1tnra_.fasta

./group102/reference/d1kxga_-d2tnfa_.fasta

./group103/reference/d1hn0a3-d1j0ma2.fasta

./group104/reference/d1dmza_-d1g6ga_.fasta

./group104/reference/d1dmza_-d1lgpa_.fasta

./group104/reference/d1g6ga_-d1gxca_.fasta

./group105/reference/d1a3k__-d1g86a_.fasta

./group105/reference/d1bkza_-d1g86a_.fasta

./group105/reference/d1bkza_-d1nls__.fasta

./group105/reference/d1d2sa_-d1dyka1.fasta

./group105/reference/d1d2sa_-d1dyka2.fasta

./group105/reference/d1dyka1-d1dyka2.fasta

./group105/reference/d1kit_1-d1kit_2.fasta

./group106/reference/d1g8kb_-d1nyka_.fasta

./group106/reference/d1nyka_-d1rie__.fasta

./group106/reference/d1jm1a_-d1rie__.fasta

./group106/reference/d1jm1a_-d1o7na1.fasta

./group106/reference/d1jm1a_-d1nyka_.fasta

./group106/reference/d1fqta_-d1nyka_.fasta

./group106/reference/d1fqta_-d1rie__.fasta

./group106/reference/d1fqta_-d1jm1a_.fasta

./group106/reference/d1o7na1-d1rfs__.fasta

./group106/reference/d1g8kb_-d1rfs__.fasta

./group108/reference/d1gl5a_-d1i1ja_.fasta

./group108/reference/d1awj__-d1ng2a1.fasta

./group108/reference/d1ng2a2-d1ycsb2.fasta

./group108/reference/d1pht__-d1qcfa1.fasta

./group108/reference/d1gcqc_-d1k4us_.fasta

./group108/reference/d1k4us_-d1ng2a2.fasta

./group108/reference/d1gcqa_-d1pht__.fasta

./group108/reference/d1gcqa_-d1ng2a2.fasta

./group108/reference/d1bb9__-d1jqqa_.fasta

./group108/reference/d1i07a_-d1neb__.fasta

./group109/reference/d1dj7b_-d1ireb_.fasta

./group109/reference/d1dj7b_-d1vie__.fasta

./group109/reference/d1dj7b_-d2ahjb_.fasta

./group109/reference/d1ireb_-d1jb0e_.fasta

./group109/reference/d1ireb_-d1vie__.fasta

./group109/reference/d1vie__-d2ahjb_.fasta

./group110/reference/d1jj2a1-d1jj2s_.fasta

./group110/reference/d1jj2p_-d1jj2s_.fasta

./group110/reference/d1jj2p_-d1m1ga2.fasta

./group110/reference/d1jj2s_-d1m1ga2.fasta

./group110/reference/d1khia1-d2eifa1.fasta

./group112/reference/d1m5za_-d1obza1.fasta

./group112/reference/d1k32a1-d1ntea_.fasta

./group112/reference/d1kwaa_-d1ntea_.fasta

./group112/reference/d1d5ga_-d1obza1.fasta

./group112/reference/d1g9oa_-d1nf3c_.fasta

./group112/reference/d1g9oa_-d1qlca_.fasta

./group112/reference/d1lcya1-d1obza1.fasta

./group112/reference/d1lcya1-d1nf3c_.fasta

./group112/reference/d1i16__-d1m5za_.fasta

./group112/reference/d1m5za_-d1qlca_.fasta

./group113/reference/d1kq1a_-d1mgqa_.fasta

./group113/reference/d1b34a_-d1d3bb_.fasta

./group113/reference/d1d3bb_-d1i8fa_.fasta

./group113/reference/d1b34a_-d1mgqa_.fasta

./group113/reference/d1d3ba_-d1h641_.fasta

./group113/reference/d1b34a_-d1d3ba_.fasta

./group113/reference/d1d3ba_-d1mgqa_.fasta

./group113/reference/d1d3ba_-d1n9ra_.fasta

./group113/reference/d1b34a_-d1ljoa_.fasta

./group113/reference/d1b34a_-d1h641_.fasta

./group114/reference/d1an8_1-d1enfa1.fasta

./group114/reference/d1an8_1-d1fnua1.fasta

./group114/reference/d1an8_1-d3seb_1.fasta

./group114/reference/d1eu3a1-d1fnua1.fasta

./group115/reference/d1uapa_-d1ueab_.fasta

./group116/reference/d1eova1-d1fjgl_.fasta

./group116/reference/d1fgua2-d1o7ia_.fasta

./group116/reference/d1gd7a_-d1jb7a3.fasta

./group116/reference/d1e1oa1-d1fjgq_.fasta

./group116/reference/d1gd7a_-d1jjcb3.fasta

./group116/reference/d1ltla_-d1qvca_.fasta

./group116/reference/d1c0aa1-d1e1oa1.fasta

./group116/reference/d1fl0a_-d1jjcb3.fasta

./group116/reference/d1eova1-d1ltla_.fasta

./group116/reference/d1c0aa1-d1eova1.fasta

./group117/reference/d1e9ga_-d1i40a_.fasta

./group117/reference/d1e9ga_-d2prd__.fasta

./group118/reference/d1fr3a_-d1g2913.fasta

./group118/reference/d1fr3a_-d1h9ra1.fasta

./group118/reference/d1fr3a_-d1h9ra2.fasta

./group118/reference/d1g2913-d1guta_.fasta

./group118/reference/d1g2913-d1h9ra1.fasta

./group118/reference/d1g2914-d1guta_.fasta

./group118/reference/d1guta_-d1h9ra1.fasta

./group118/reference/d1guta_-d1h9ra2.fasta

./group118/reference/d1h9ra1-d1h9ra2.fasta

./group120/reference/d1ggpb1-d1knma_.fasta

./group120/reference/d1dqga_-d1knma_.fasta

./group120/reference/d1abrb2-d1knma_.fasta

./group120/reference/d1ggpb1-d1hwmb2.fasta

./group120/reference/d1abrb2-d1dqga_.fasta

./group120/reference/d1hwmb2-d1knma_.fasta

./group120/reference/d1hwmb2-d1m2tb1.fasta

./group120/reference/d1hwmb1-d1hwmb2.fasta

./group120/reference/d1m2tb1-d1m2tb2.fasta

./group120/reference/d1dqga_-d1m2tb2.fasta

./group121/reference/d1avac_-d1wba__.fasta

./group122/reference/d1dfca2-d1dfca4.fasta

./group122/reference/d1dfca4-d1hcd__.fasta

./group123/reference/d1dar_1-d1exma1.fasta

./group123/reference/d1dar_1-d1n0ua1.fasta

./group123/reference/d1exma1-d1n0ua1.fasta

./group124/reference/d1d2ea2-d1f60a2.fasta

./group126/reference/d1eq9a_-d1orfa_.fasta

./group126/reference/d1arb__-d1m9ua_.fasta

./group126/reference/d1klih_-d1orfa_.fasta

./group126/reference/d1bqya_-d1eq9a_.fasta

./group126/reference/d1bio__-d1sgt__.fasta

./group126/reference/d1orfa_-d2hlca_.fasta

./group126/reference/d1gvza_-d1m9ua_.fasta

./group126/reference/d1bqya_-d2hlca_.fasta

./group126/reference/d1fjsa_-d1gvza_.fasta

./group126/reference/d1bqya_-d1m9ua_.fasta

./group128/reference/d1j71a_-d1lf2a_.fasta

./group128/reference/d1j71a_-d2apr__.fasta

./group128/reference/d1j71a_-d1mpp__.fasta

./group128/reference/d1fkna_-d1idaa_.fasta

./group128/reference/d1idaa_-d1lf2a_.fasta

./group128/reference/d1fkna_-d1mpp__.fasta

./group128/reference/d1dpja_-d1fkna_.fasta

./group128/reference/d1fmb__-d1nsoa_.fasta

./group128/reference/d1fkna_-d1j71a_.fasta

./group128/reference/d1dpja_-d1j71a_.fasta

./group129/reference/d1ffya2-d1h3na2.fasta

./group129/reference/d1ffya2-d1ivsa3.fasta

./group129/reference/d1ile_2-d1ivsa3.fasta

./group130/reference/d1cz4a1-d1h0ha1.fasta

./group130/reference/d1g8ka1-d1h0ha1.fasta

./group130/reference/d1cz4a1-d1g8ka1.fasta

./group130/reference/d1g8ka1-d2napa1.fasta

./group130/reference/d1tmo_1-d2napa1.fasta

./group130/reference/d1eu1a1-d1kqfa1.fasta

./group130/reference/d1h0ha1-d1tmo_1.fasta

./group130/reference/d1kqfa1-d1tmo_1.fasta

./group130/reference/d1eu1a1-d1h0ha1.fasta

./group130/reference/d1g8ka1-d1tmo_1.fasta

./group131/reference/d1btn__-d1qqga1.fasta

./group131/reference/d1ddma_-d1mixa2.fasta

./group131/reference/d1eaza_-d1qqga1.fasta

./group131/reference/d1eaza_-d1mai__.fasta

./group131/reference/d1ddma_-d1gg3a2.fasta

./group131/reference/d1btn__-d1pls__.fasta

./group131/reference/d1eaza_-d1fhoa_.fasta

./group131/reference/d1pls__-d1qqga1.fasta

./group131/reference/d1dyna_-d1pls__.fasta

./group131/reference/d1h4ra2-d1mixa2.fasta

./group133/reference/d1jv4a_-d1jzua_.fasta

./group133/reference/d1beba_-d1dzka_.fasta

./group133/reference/d1ifc__-d1o1va_.fasta

./group133/reference/d1cbs__-d1mdc__.fasta

./group133/reference/d1ew3a_-d1jzua_.fasta

./group133/reference/d1hms__-d1o1va_.fasta

./group133/reference/d1beba_-d1jzua_.fasta

./group133/reference/d1kqwa_-d1mdc__.fasta

./group133/reference/d1ggla_-d1o1va_.fasta

./group133/reference/d1b56__-d1ifc__.fasta

./group135/reference/d1fbl_1-d1itva_.fasta

./group135/reference/d1itva_-d1pex__.fasta

./group136/reference/d1eur__-d1n1ta2.fasta

./group136/reference/d1eur__-d2sli_2.fasta

./group136/reference/d1eur__-d3sil__.fasta

./group136/reference/d1n1ta2-d2sli_2.fasta

./group136/reference/d2sli_2-d3sil__.fasta

./group137/reference/d1nr0a1-d1p22a2.fasta

./group137/reference/d1p22a2-d1tbga_.fasta

./group138/reference/d1ea9c2-d1hx0a1.fasta

./group138/reference/d1gcya1-d1qhoa3.fasta

./group138/reference/d1bag_1-d1ji1a2.fasta

./group138/reference/d1g5aa1-d1m7xa2.fasta

./group138/reference/d1iv8a1-d7taa_1.fasta

./group138/reference/d1hx0a1-d1ji1a2.fasta

./group138/reference/d1bag_1-d1jae_1.fasta

./group138/reference/d1m53a1-d1m7xa2.fasta

./group138/reference/d1ktba1-d1m53a1.fasta

./group138/reference/d1bag_1-d1g94a1.fasta

./group139/reference/d1eg3a3-d1i5hw_.fasta

./group141/reference/d1ciy_2-d1ji6a2.fasta

./group143/reference/d1bhe__-d1hg8a_.fasta

./group143/reference/d1bhe__-d1k5ca_.fasta

./group143/reference/d1bn8a_-d1qcxa_.fasta

./group143/reference/d1hg8a_-d1jtaa_.fasta

./group143/reference/d1hg8a_-d1rmg__.fasta

./group144/reference/d1kk6a_-d1lxa__.fasta

./group144/reference/d1kk6a_-d3tdt__.fasta

./group144/reference/d1krra_-d1lxa__.fasta

./group144/reference/d1krra_-d1qrea_.fasta

./group144/reference/d1lxa__-d1ocxa_.fasta

./group144/reference/d1ocxa_-d1qrea_.fasta

./group144/reference/d1ocxa_-d3tdt__.fasta

./group144/reference/d1qrea_-d3tdt__.fasta

./group145/reference/d1fi2a_-d1lrha_.fasta

./group145/reference/d1fi2a_-d1od5a2.fasta

./group145/reference/d1fxza2-d1m4oa_.fasta

./group146/reference/d1gp6a_-d1odma_.fasta

./group147/reference/d1cx4a1-d1o7fa2.fasta

./group147/reference/d1cx4a1-d1o7fa3.fasta

./group147/reference/d1ft9a2-d1i5za2.fasta

./group147/reference/d1i5za2-d1o7fa2.fasta

./group147/reference/d1i5za2-d1o7fa3.fasta

./group147/reference/d1i5za2-d1rgs_1.fasta

./group147/reference/d1o7fa2-d1rgs_1.fasta

./group147/reference/d1o7fa2-d1rgs_2.fasta

./group147/reference/d1o7fa3-d1rgs_1.fasta

./group148/reference/d1fyc__-d1k8ma_.fasta

./group148/reference/d1fyc__-d1qjoa_.fasta

./group148/reference/d1bdo__-d1k8ma_.fasta

./group148/reference/d1dd2a_-d1k8ma_.fasta

./group148/reference/d1dd2a_-d1fyc__.fasta

./group148/reference/d1bdo__-d1ghk__.fasta

./group148/reference/d1bdo__-d1lac__.fasta

./group148/reference/d1ghk__-d1lac__.fasta

./group148/reference/d1dd2a_-d1htp__.fasta

./group148/reference/d1bdo__-d1htp__.fasta

./group149/reference/d1dv1a1-d1e2wa2.fasta

./group149/reference/d1dv1a1-d1kjqa1.fasta

./group152/reference/d1dun__-d1euwa_.fasta

./group152/reference/d1euwa_-d1f7da_.fasta

./group152/reference/d1euwa_-d1ogha_.fasta

./group152/reference/d1f7da_-d1ogha_.fasta

./group152/reference/d1mq7a_-d1ogha_.fasta

./group153/reference/d1hg3a_-d1lyxa_.fasta

./group153/reference/d1hg3a_-d1n55a_.fasta

./group154/reference/d1a53__-d1thfd_.fasta

./group154/reference/d1dbta_-d1dqwa_.fasta

./group154/reference/d1dbta_-d1km3a_.fasta

./group154/reference/d1dqwa_-d1eixa_.fasta

./group154/reference/d1eixa_-d1km3a_.fasta

./group154/reference/d1km3a_-d1kv8a_.fasta

./group154/reference/d1rpxa_-d1thfd_.fasta

./group155/reference/d1ep3a_-d1gvoa_.fasta

./group155/reference/d1gvoa_-d1o94a1.fasta

./group156/reference/d1exba_-d1gvea_.fasta

./group156/reference/d1exba_-d1hqta_.fasta

./group156/reference/d1exba_-d1j96a_.fasta

./group156/reference/d1gvea_-d1hqta_.fasta

./group156/reference/d1gvea_-d1j96a_.fasta

./group156/reference/d1gvea_-d1lqaa_.fasta

./group156/reference/d1hqta_-d1lqaa_.fasta

./group156/reference/d1j96a_-d1lqaa_.fasta

./group157/reference/d1e43a2-d1lwha2.fasta

./group157/reference/d1g5aa2-d1uok_2.fasta

./group157/reference/d1hxja_-d1qvba_.fasta

./group157/reference/d1ji1a3-d1pama4.fasta

./group157/reference/d1m53a2-d1qhoa4.fasta

./group157/reference/d1e43a2-d1ji1a3.fasta

./group157/reference/d1e43a2-d1pama4.fasta

./group157/reference/d1lwha2-d7taa_2.fasta

./group157/reference/d1eh9a3-d1ji1a3.fasta

./group157/reference/d1bf2_3-d1gjwa2.fasta

./group159/reference/d1dhpa_-d1f74a_.fasta

./group159/reference/d1dhpa_-d1hl2a_.fasta

./group159/reference/d1i2oa_-d1l6wa_.fasta

./group159/reference/d1jcla_-d1mzha_.fasta

./group159/reference/d1jcla_-d1n7ka_.fasta

./group160/reference/d1ec7a1-d2mnr_1.fasta

./group160/reference/d1jpdx1-d1muca1.fasta

./group160/reference/d1jpdx1-d2chr_1.fasta

./group160/reference/d1jpma1-d1muca1.fasta

./group160/reference/d1jpma1-d2chr_1.fasta

./group160/reference/d1jpma1-d2mnr_1.fasta

./group160/reference/d1muca1-d2mnr_1.fasta

./group162/reference/d1a0ca_-d1muwa_.fasta

./group166/reference/d1eny__-d1g0oa_.fasta

./group166/reference/d1ek6a_-d1ja9a_.fasta

./group166/reference/d1b16a_-d1ja9a_.fasta

./group166/reference/d1g0oa_-d1hxha_.fasta

./group166/reference/d1kewa_-d1n5da_.fasta

./group166/reference/d1eny__-d1fmca_.fasta

./group166/reference/d1g0oa_-d1h5qa_.fasta

./group166/reference/d1eny__-d1ja9a_.fasta

./group166/reference/d1eno__-d1ja9a_.fasta

./group166/reference/d1n5da_-d1oaa__.fasta

./group167/reference/d1ebda2-d1fl2a2.fasta

./group167/reference/d1ebda2-d1gesa2.fasta

./group167/reference/d1jeha2-d1vdc_2.fasta

./group167/reference/d1feca2-d1jeha2.fasta

./group167/reference/d1cjca1-d3grs_2.fasta

./group167/reference/d1d7ya2-d1feca2.fasta

./group167/reference/d1gesa2-d1nhp_2.fasta

./group167/reference/d1h6va2-d1ojt_2.fasta

./group167/reference/d1mo9a2-d3grs_2.fasta

./group167/reference/d1d7ya2-d1ebda2.fasta

./group168/reference/d1oc7a_-d1tml__.fasta

./group170/reference/d1a4ya_-d1fqva2.fasta

./group170/reference/d1a4ya_-d1yrga_.fasta

./group171/reference/d1h6ta2-d1ozna_.fasta

./group171/reference/d1a9na_-d1p9ag_.fasta

./group171/reference/d1h6ua2-d1nqla1.fasta

./group171/reference/d1h6ua2-d1p9ag_.fasta

./group171/reference/d1a9na_-d1jl5a_.fasta

./group171/reference/d1igra1-d1m6ba2.fasta

./group171/reference/d1h6ua2-d1koha1.fasta

./group171/reference/d1m6ba2-d1nqla1.fasta

./group171/reference/d1h6ua2-d1ozna_.fasta

./group171/reference/d1a9na_-d1h6ua2.fasta

./group172/reference/d1dcia_-d1hzda_.fasta

./group172/reference/d1dcia_-d1nzya_.fasta

./group172/reference/d1dcia_-d1o8ua_.fasta

./group172/reference/d1hzda_-d1nzya_.fasta

./group172/reference/d1hzda_-d1o8ua_.fasta

./group172/reference/d1nzya_-d1o8ua_.fasta

./group172/reference/d1on3a1-d1on3a2.fasta

./group173/reference/d1cdza_-d1dgtb3.fasta

./group173/reference/d1cdza_-d1in1a_.fasta

./group174/reference/d1c2ya_-d1di0a_.fasta

./group174/reference/d1c2ya_-d1ejba_.fasta

./group174/reference/d1di0a_-d1ejba_.fasta

./group174/reference/d1di0a_-d1hqka_.fasta

./group174/reference/d1di0a_-d1kz1a_.fasta

./group175/reference/d1l9ga_-d1muga_.fasta

./group176/reference/d1mvoa_-d1tmy__.fasta

./group176/reference/d1a04a2-d1a2oa1.fasta

./group176/reference/d1a04a2-d1nat__.fasta

./group176/reference/d1a04a2-d1dz3a_.fasta

./group176/reference/d1b00a_-d1dbwa_.fasta

./group176/reference/d1a04a2-d1b00a_.fasta

./group176/reference/d1dz3a_-d1ntr__.fasta

./group176/reference/d1dbwa_-d1ntr__.fasta

./group176/reference/d1a2oa1-d1nat__.fasta

./group176/reference/d1dbwa_-d1mvoa_.fasta

./group177/reference/d2fcr__-d5nul__.fasta

./group177/reference/d1f4pa_-d1ja1a2.fasta

./group177/reference/d1e5da1-d5nul__.fasta

./group177/reference/d1ag9a_-d5nul__.fasta

./group177/reference/d1ja1a2-d2fcr__.fasta

./group177/reference/d1bvyf_-d1f4pa_.fasta

./group177/reference/d1f4pa_-d5nul__.fasta

./group177/reference/d1oboa_-d5nul__.fasta

./group177/reference/d1ja1a2-d1oboa_.fasta

./group177/reference/d1fuea_-d1ja1a2.fasta

./group178/reference/d1bmta2-d1ccwa_.fasta

./group178/reference/d1ccwa_-d7reqa2.fasta

./group179/reference/d1es9a_-d1esc__.fasta

./group179/reference/d1es9a_-d1k7ca_.fasta

./group181/reference/d1gpma2-d1i7qb_.fasta

./group181/reference/d1gpma2-d1k9vf_.fasta

./group181/reference/d1gpma2-d1ka9h_.fasta

./group181/reference/d1gpma2-d1l9xa_.fasta

./group181/reference/d1gpma2-d1qdlb_.fasta

./group181/reference/d1i7qb_-d1ka9h_.fasta

./group181/reference/d1k9vf_-d1l9xa_.fasta

./group181/reference/d1k9vf_-d1qdlb_.fasta

./group181/reference/d1ka9h_-d1pe0a_.fasta

./group182/reference/d1a8p_2-d1f20a2.fasta

./group182/reference/d1ddga2-d2cnd_2.fasta

./group182/reference/d1cqxa3-d1krha2.fasta

./group182/reference/d1a8p_2-d1que_2.fasta

./group182/reference/d1i7pa2-d1que_2.fasta

./group182/reference/d1gvha3-d2pia_2.fasta

./group182/reference/d1que_2-d2cnd_2.fasta

./group182/reference/d1a8p_2-d1cqxa3.fasta

./group182/reference/d1cqxa3-d2pia_2.fasta

./group182/reference/d2cnd_2-d2pia_2.fasta

./group183/reference/d1coza_-d1ej2a_.fasta

./group183/reference/d1coza_-d1ihoa_.fasta

./group183/reference/d1coza_-d1k4ma_.fasta

./group183/reference/d1coza_-d1qjca_.fasta

./group183/reference/d1ej2a_-d1k4ma_.fasta

./group183/reference/d1gtra2-d1j09a2.fasta

./group183/reference/d1jhda2-d1qjca_.fasta

./group184/reference/d1efpa1-d1j20a1.fasta

./group184/reference/d1efva1-d1o97d1.fasta

./group185/reference/d1dnpa2-d1iqra2.fasta

./group185/reference/d1dnpa2-d1np7a2.fasta

./group185/reference/d1dnpa2-d1qnf_2.fasta

./group185/reference/d1iqra2-d1np7a2.fasta

./group185/reference/d1np7a2-d1qnf_2.fasta

./group186/reference/d1a9xa4-d1iow_1.fasta

./group186/reference/d1b6ra2-d1kjqa2.fasta

./group186/reference/d1e4ea1-d1gsa_1.fasta

./group186/reference/d1gsoa2-d1i7na1.fasta

./group186/reference/d1gsoa2-d1kjqa2.fasta

./group187/reference/d1efva2-d1jsca1.fasta

./group187/reference/d1d4oa_-d1m2ka_.fasta

./group187/reference/d1d4oa_-d1poxa1.fasta

./group187/reference/d1jsca1-d1o97d2.fasta

./group187/reference/d1efva2-d1zpda1.fasta

./group187/reference/d1d4oa_-d1efva2.fasta

./group187/reference/d1ovma1-d1zpda1.fasta

./group187/reference/d1m2ka_-d1pvda1.fasta

./group187/reference/d1jsca1-d1poxa1.fasta

./group187/reference/d1efva2-d1m2ka_.fasta

./group189/reference/d1im5a_-d1nbaa_.fasta

./group189/reference/d1im5a_-d1nf9a_.fasta

./group189/reference/d1im5a_-d1yaca_.fasta

./group189/reference/d1nf9a_-d1yaca_.fasta

./group190/reference/d1poxa2-d1pvda2.fasta

./group190/reference/d1bfd_2-d1ovma2.fasta

./group190/reference/d1poxa3-d1zpda3.fasta

./group190/reference/d1jsca2-d1poxa2.fasta

./group190/reference/d1ovma2-d1poxa2.fasta

./group190/reference/d1jsca2-d1ovma2.fasta

./group190/reference/d1bfd_2-d1pvda2.fasta

./group190/reference/d1jsca2-d1pvda2.fasta

./group190/reference/d1bfd_2-d1poxa2.fasta

./group190/reference/d1bfd_3-d1zpda3.fasta

./group191/reference/d1bg2__-d1mt0a_.fasta

./group191/reference/d1g6ha_-d1pf4a1.fasta

./group191/reference/d1d2na_-d1eg7a_.fasta

./group191/reference/d1f60a3-d1hyqa_.fasta

./group191/reference/d1g2912-d1g6ha_.fasta

./group191/reference/d1cp2a_-d1hyqa_.fasta

./group191/reference/d1f60a3-d1mt0a_.fasta

./group191/reference/d1bg2__-d1oxsc2.fasta

./group191/reference/d1oxsc2-d1uaaa1.fasta

./group191/reference/d1bg2__-d1eg7a_.fasta

./group192/reference/d1ea7a_-d1ga6a_.fasta

./group192/reference/d1ic6a_-d1ot5a2.fasta

./group192/reference/d1gt91_-d1ic6a_.fasta

./group192/reference/d1ea7a_-d1gt91_.fasta

./group192/reference/d1ga6a_-d1gci__.fasta

./group192/reference/d1p8ja2-d1thm__.fasta

./group192/reference/d1ic6a_-d1p8ja2.fasta

./group192/reference/d1gt91_-d1p8ja2.fasta

./group192/reference/d1gci__-d1p8ja2.fasta

./group192/reference/d1ga6a_-d1thm__.fasta

./group194/reference/d1eaf__-d3cla__.fasta

./group195/reference/d1d1qa_-d1jf8a_.fasta

./group196/reference/d1d5ra2-d1jlna_.fasta

./group196/reference/d1d5ra2-d1lara2.fasta

./group196/reference/d1d5ra2-d1lyva_.fasta

./group196/reference/d1fpza_-d1mkp__.fasta

./group196/reference/d1jlna_-d1lyva_.fasta

./group196/reference/d1lara1-d1lyva_.fasta

./group196/reference/d1lara2-d1lyva_.fasta

./group196/reference/d1lyva_-d1vhra_.fasta

./group198/reference/d1g7ea_-d1quwa_.fasta

./group198/reference/d1hyua3-d2trxa_.fasta

./group198/reference/d1a8l_2-d1mek__.fasta

./group198/reference/d1a8l_2-d1kte__.fasta

./group198/reference/d1eeja1-d2trxa_.fasta

./group198/reference/d1g7ea_-d1mek__.fasta

./group198/reference/d1fb6a_-d1jfua_.fasta

./group198/reference/d1eeja1-d1kte__.fasta

./group198/reference/d1mek__-d1quwa_.fasta

./group198/reference/d1a8l_1-d1g7ea_.fasta

./group199/reference/d1gpua3-d1l8aa3.fasta

./group199/reference/d1itza3-d1l8aa3.fasta

./group199/reference/d1l8aa3-d1qgda3.fasta

./group202/reference/d1atia1-d1kmma1.fasta

./group202/reference/d1atia1-d1nj1a1.fasta

./group202/reference/d1atia1-d1nj8a1.fasta

./group202/reference/d1atia1-d1qf6a1.fasta

./group202/reference/d1h4vb1-d1hc7a1.fasta

./group202/reference/d1h4vb1-d1qe0a1.fasta

./group202/reference/d1hc7a1-d1qe0a1.fasta

./group202/reference/d1kmma1-d1qe0a1.fasta

./group202/reference/d1nj8a1-d1qf6a1.fasta

./group203/reference/d1ckqa_-d1d02a_.fasta

./group203/reference/d1f1za2-d1m0da_.fasta

./group203/reference/d1f1za2-d1vsra_.fasta

./group203/reference/d1gefa_-d1vsra_.fasta

./group203/reference/d1j23a_-d3pvia_.fasta

./group203/reference/d1m0da_-d1vsra_.fasta

./group204/reference/d1ddza1-d1ekja_.fasta

./group204/reference/d1ddza1-d1g5ca_.fasta

./group204/reference/d1ekja_-d1g5ca_.fasta

./group204/reference/d1ekja_-d1i6pa_.fasta

./group205/reference/d1bu6o1-d1ig8a1.fasta

./group205/reference/d1bupa2-d1nbwa3.fasta

./group205/reference/d1czan1-d1czan2.fasta

./group205/reference/d1czan1-d1g99a2.fasta

./group205/reference/d1e4ft1-d1huxa_.fasta

./group206/reference/d1ih7a1-d1tgoa1.fasta

./group206/reference/d1jl1a_-d1t7pa1.fasta

./group206/reference/d1kfsa1-d1l3sa1.fasta

./group206/reference/d1kfsa1-d1qtma1.fasta

./group206/reference/d1kfsa1-d1t7pa1.fasta

./group206/reference/d1kfsa1-d1tgoa1.fasta

./group207/reference/d1fjgk_-d1ilya_.fasta

./group209/reference/d1h8la2-d1jqga1.fasta

./group209/reference/d1h8la2-d1kwma1.fasta

./group209/reference/d1h8la2-d1m4la_.fasta

./group210/reference/d1a4ia2-d1edza2.fasta

./group210/reference/d1b0aa2-d1edza2.fasta

./group210/reference/d1bgva2-d1hwxa2.fasta

./group210/reference/d1c1da2-d1gtma2.fasta

./group210/reference/d1c1da2-d1hwxa2.fasta

./group210/reference/d1gtma2-d1leha2.fasta

./group210/reference/d1hwxa2-d1leha2.fasta

./group210/reference/d1npya2-d1nyta2.fasta

./group211/reference/d1bif_2-d1e58a_.fasta

./group211/reference/d1bif_2-d1fzta_.fasta

./group211/reference/d1bif_2-d1h2ea_.fasta

./group211/reference/d1bif_2-d3pgm__.fasta

./group211/reference/d1e58a_-d1h2ea_.fasta

./group211/reference/d1fzta_-d1h2ea_.fasta

./group211/reference/d1h2ea_-d3pgm__.fasta

./group211/reference/d1nd6a_-d3pgm__.fasta

./group212/reference/d1bzya_-d1dqna_.fasta

./group212/reference/d1ecfa1-d1lh0a_.fasta

./group212/reference/d1bzya_-d1qb7a_.fasta

./group212/reference/d1gph11-d1lh0a_.fasta

./group212/reference/d1bd3a_-d1l1qa_.fasta

./group212/reference/d1dqna_-d1fsga_.fasta

./group212/reference/d1bzya_-d1ecfa1.fasta

./group212/reference/d1lh0a_-d1qb7a_.fasta

./group212/reference/d1fsga_-d1i5ea_.fasta

./group212/reference/d1l1qa_-d1qb7a_.fasta

./group213/reference/d1atza_-d1ijba_.fasta

./group213/reference/d1atza_-d1mf7a_.fasta

./group213/reference/d1atza_-d1mjna_.fasta

./group213/reference/d1atza_-d1qc5a_.fasta

./group213/reference/d1ijba_-d1mjna_.fasta

./group213/reference/d1mjna_-d1qc5a_.fasta

./group214/reference/d1i1na_-d1iy9a_.fasta

./group214/reference/d1g6q1_-d1nw3a_.fasta

./group214/reference/d1i1na_-d1kywa2.fasta

./group214/reference/d1dcta_-d1f3la_.fasta

./group214/reference/d1i1na_-d1oria_.fasta

./group214/reference/d1g6q1_-d1i1na_.fasta

./group214/reference/d1g6q1_-d1nv8a_.fasta

./group214/reference/d1dl5a1-d1nv8a_.fasta

./group214/reference/d1f3la_-d1i9ga_.fasta

./group214/reference/d1g38a_-d1jg1a_.fasta

./group215/reference/d1gdea_-d3tata_.fasta

./group215/reference/d1gtxa_-d2gsaa_.fasta

./group215/reference/d1c7na_-d1o4sa_.fasta

./group215/reference/d2dkb__-d2gsaa_.fasta

./group215/reference/d1elua_-d1jf9a_.fasta

./group215/reference/d1elua_-d1j32a_.fasta

./group215/reference/d1gtxa_-d2dkb__.fasta

./group215/reference/d1gtxa_-d2oata_.fasta

./group215/reference/d1c7na_-d1gdea_.fasta

./group215/reference/d1ibja_-d1o4sa_.fasta

./group216/reference/d1e5ka_-d1h7ea_.fasta

./group216/reference/d1e5ka_-d1hv9a2.fasta

./group217/reference/d1ex9a_-d1ku0a_.fasta

./group217/reference/d1mtza_-d1qtra_.fasta

./group217/reference/d1llfa_-d1mx1a_.fasta

./group217/reference/d1ju3a2-d1l7aa_.fasta

./group217/reference/d1ju3a2-d1lnsa3.fasta

./group217/reference/d1l7aa_-d1lzla_.fasta

./group217/reference/d1jkma_-d1llfa_.fasta

./group217/reference/d1c4xa_-d1ku0a_.fasta

./group217/reference/d1ea5a_-d1jkma_.fasta

./group217/reference/d1lzla_-d1mx1a_.fasta

./group218/reference/d1aoea_-d1d1ga_.fasta

./group218/reference/d1aoea_-d1vdra_.fasta

./group218/reference/d1d1ga_-d1df7a_.fasta

./group218/reference/d1d1ga_-d1dyr__.fasta

./group218/reference/d1d1ga_-d1ra9__.fasta

./group218/reference/d1d1ga_-d1vdra_.fasta

./group218/reference/d1dyr__-d1vdra_.fasta

./group218/reference/d1dyr__-d3dfr__.fasta

./group218/reference/d1ra9__-d3dfr__.fasta

./group218/reference/d1vdra_-d3dfr__.fasta

./group220/reference/d1b7ba_-d1gs5a_.fasta

./group220/reference/d1e19a_-d1gs5a_.fasta

./group221/reference/d1e4bp_-d1k0wa_.fasta

./group223/reference/d1cnza_-d1lwda_.fasta

./group223/reference/d1lwda_-d1xaa__.fasta

./group224/reference/d1a1s_2-d1ekxa2.fasta

./group224/reference/d1a1s_2-d1ml4a2.fasta

./group224/reference/d1duvg2-d1ml4a2.fasta

./group224/reference/d1ekxa2-d1js1x1.fasta

./group224/reference/d1js1x1-d1otha1.fasta

./group224/reference/d1js1x2-d1otha2.fasta

./group224/reference/d1ml4a2-d1otha2.fasta

./group225/reference/d1b74a1-d1jfla1.fasta

./group225/reference/d1b74a2-d1jfla2.fasta

./group225/reference/d1jfla1-d1jfla2.fasta

./group226/reference/d1j6na_-d1qopb_.fasta

./group226/reference/d1j6na_-d1tdj_1.fasta

./group226/reference/d1qopb_-d1tdj_1.fasta

./group227/reference/d1c7qa_-d1iata_.fasta

./group228/reference/d1g8ka2-d1tmo_2.fasta

./group228/reference/d1g8ka2-d2napa2.fasta

./group228/reference/d1tmo_2-d2napa2.fasta

./group229/reference/d1a4sa_-d1ad3a_.fasta

./group229/reference/d1a4sa_-d1ez0a_.fasta

./group229/reference/d1ad3a_-d1euha_.fasta

./group229/reference/d1ad3a_-d1ky8a_.fasta

./group229/reference/d1ad3a_-d1o04a_.fasta

./group229/reference/d1euha_-d1ez0a_.fasta

./group229/reference/d1ez0a_-d1o04a_.fasta

./group229/reference/d1ky8a_-d1o04a_.fasta

./group230/reference/d1k2yx1-d3pmga1.fasta

./group230/reference/d1k2yx2-d3pmga2.fasta

./group234/reference/d1doza_-d1hrka_.fasta

./group234/reference/d1doza_-d1lbqa_.fasta

./group235/reference/d1m1na_-d1miob_.fasta

./group236/reference/d1gca__-d1jx6a_.fasta

./group236/reference/d1gca__-d1jyea_.fasta

./group236/reference/d1gca__-d1rpja_.fasta

./group236/reference/d1gca__-d2dri__.fasta

./group236/reference/d1gca__-d8abp__.fasta

./group236/reference/d1jx6a_-d2dri__.fasta

./group236/reference/d1jyea_-d2dri__.fasta

./group236/reference/d1rpja_-d8abp__.fasta

./group236/reference/d2dri__-d8abp__.fasta

./group237/reference/d1a8e__-d1sbp__.fasta

./group237/reference/d1a99a_-d1j1na_.fasta

./group237/reference/d1a99a_-d3mbp__.fasta

./group237/reference/d1al3__-d1i6aa_.fasta

./group237/reference/d1amf__-d1atg__.fasta

./group237/reference/d1dpe__-d1jeta_.fasta

./group237/reference/d1eu8a_-d3mbp__.fasta

./group237/reference/d1jeta_-d1pot__.fasta

./group237/reference/d1lst__-d1mqda_.fasta

./group237/reference/d1mqda_-d1wdna_.fasta

./group238/reference/d1hzpa1-d1kas_1.fasta

./group238/reference/d1hzpa1-d1ox0a1.fasta

./group238/reference/d1e5ma1-d1hzpa1.fasta

./group238/reference/d1kas_1-d1m3ka1.fasta

./group238/reference/d1e5ma2-d1ub7a2.fasta

./group238/reference/d1e5ma1-d1hnja1.fasta

./group238/reference/d1kas_1-d1mzja1.fasta

./group238/reference/d1m3ka2-d1mzja1.fasta

./group238/reference/d1m3ka1-d1ub7a2.fasta

./group238/reference/d1afwa2-d1m3ka1.fasta

./group239/reference/d1aln_1-d1uaqa_.fasta

./group239/reference/d1jtka_-d1uaqa_.fasta

./group240/reference/d1a2pa_-d1lnia_.fasta

./group241/reference/d153l__-d1qsaa2.fasta

./group241/reference/d1chka_-d1qgia_.fasta

./group242/reference/d2cb5a_-d7pcka_.fasta

./group242/reference/d1gmya_-d1me4a_.fasta

./group242/reference/d1cvza_-d3gcb__.fasta

./group242/reference/d1deua_-d1me4a_.fasta

./group242/reference/d1f13a4-d1iwda_.fasta

./group242/reference/d1deua_-d1l9na4.fasta

./group242/reference/d1g0da4-d1iwda_.fasta

./group242/reference/d1deua_-d1iwda_.fasta

./group242/reference/d1gmya_-d2cb5a_.fasta

./group242/reference/d1gmya_-d7pcka_.fasta

./group245/reference/d1b3aa_-d1qg7a_.fasta

./group245/reference/d1doka_-d3il8__.fasta

./group245/reference/d1b3aa_-d3il8__.fasta

./group245/reference/d1b3aa_-d1doka_.fasta

./group245/reference/d1f2la_-d2hcc__.fasta

./group245/reference/d1m8aa_-d2hcc__.fasta

./group245/reference/d1el0a_-d3il8__.fasta

./group245/reference/d1g2ta_-d1m8aa_.fasta

./group245/reference/d1j9oa_-d1tvxa_.fasta

./group245/reference/d1b3aa_-d1f2la_.fasta

./group246/reference/d1dz1a_-d1e0ba_.fasta

./group246/reference/d1dz1a_-d1knaa_.fasta

./group246/reference/d1e0ba_-d1knaa_.fasta

./group246/reference/d1g6za_-d1knaa_.fasta

./group247/reference/d1d9na_-d1qk9a_.fasta

./group250/reference/d1h72c1-d1kkha1.fasta

./group250/reference/d1p42a1-d1pkp_1.fasta

./group251/reference/d1a5r__-d1mg8a_.fasta

./group251/reference/d1euvb_-d1mg8a_.fasta

./group251/reference/d1gg3a3-d1h4ra3.fasta

./group251/reference/d1gnua_-d1j8ca_.fasta

./group251/reference/d1gnua_-d1mg8a_.fasta

./group251/reference/d1h4ra3-d1h8ca_.fasta

./group251/reference/d1h8ca_-d1i42a_.fasta

./group251/reference/d1h8ca_-d1lm8b_.fasta

./group251/reference/d1j8ca_-d1lm8b_.fasta

./group251/reference/d1j8ca_-d1m94a_.fasta

./group253/reference/d1f0za_-d1fm0d_.fasta

./group253/reference/d1f0za_-d1jsba_.fasta

./group254/reference/d1b9ra_-d1czpa_.fasta

./group254/reference/d1doi__-d1put__.fasta

./group254/reference/d1hlra2-d2pia_3.fasta

./group254/reference/d1doi__-d1feha2.fasta

./group254/reference/d1hlra2-d1nekb2.fasta

./group254/reference/d1czpa_-d2pia_3.fasta

./group254/reference/d1doi__-d2pia_3.fasta

./group254/reference/d1fo4a2-d1nekb2.fasta

./group254/reference/d1feha2-d1l5pa_.fasta

./group254/reference/d1e9ma_-d1l5pa_.fasta

./group256/reference/d1eu3a2-d3tss_2.fasta

./group256/reference/d1et9a2-d3tss_2.fasta

./group256/reference/d1an8_2-d1m4va2.fasta

./group256/reference/d3seb_2-d3tss_2.fasta

./group256/reference/d1eu3a2-d3seb_2.fasta

./group256/reference/d1enfa2-d3tss_2.fasta

./group256/reference/d1an8_2-d3seb_2.fasta

./group256/reference/d1an8_2-d3tss_2.fasta

./group256/reference/d1an8_2-d1et9a2.fasta

./group256/reference/d1et9a2-d1m4va2.fasta

./group257/reference/d1an9a2-d1ng4a2.fasta

./group257/reference/d1c0pa2-d1ng4a2.fasta

./group258/reference/d1cewi_-d1eqka_.fasta

./group258/reference/d1cewi_-d1mola_.fasta

./group258/reference/d1cewi_-d1stfi_.fasta

./group258/reference/d1eqka_-d1g96a_.fasta

./group258/reference/d1eqka_-d1mola_.fasta

./group258/reference/d1eqka_-d1stfi_.fasta

./group258/reference/d1g96a_-d1stfi_.fasta

./group259/reference/d1a2va2-d1ivwa2.fasta

./group259/reference/d1a2va2-d1ksia2.fasta

./group259/reference/d1a2va2-d1oaca2.fasta

./group259/reference/d1a2va3-d1ivwa3.fasta

./group259/reference/d1a2va3-d1oaca3.fasta

./group259/reference/d1ivwa2-d1oaca2.fasta

./group259/reference/d1ksia2-d1oaca2.fasta

./group259/reference/d1ksia3-d1oaca3.fasta

./group259/reference/d1oaca2-d1oaca3.fasta

./group260/reference/d1gy6a_-d1jkga_.fasta

./group260/reference/d1gy7a_-d1jkga_.fasta

./group260/reference/d1m98a2-d1nwwa_.fasta

./group260/reference/d1m98a2-d1ocva_.fasta

./group261/reference/d1k5na2-d3frua2.fasta

./group261/reference/d1c16a2-d3frua2.fasta

./group261/reference/d1hdma2-d1iaka2.fasta

./group261/reference/d1c16a2-d1hyrc2.fasta

./group261/reference/d1gzqa2-d1lqva_.fasta

./group261/reference/d1hyrc2-d1zaga2.fasta

./group261/reference/d1c16a2-d1de4a2.fasta

./group261/reference/d1de4a2-d3frua2.fasta

./group261/reference/d1cd1a2-d3frua2.fasta

./group261/reference/d1gzqa2-d1zaga2.fasta

./group262/reference/d1j7da_-d2aak__.fasta

./group262/reference/d1c4zd_-d1i7ka_.fasta

./group262/reference/d1j7da_-d2ucz__.fasta

./group262/reference/d1fzya_-d1j7da_.fasta

./group262/reference/d1c4zd_-d1kppa_.fasta

./group262/reference/d1i7ka_-d1kppa_.fasta

./group262/reference/d1j7da_-d1jata_.fasta

./group262/reference/d1j7da_-d1qcqa_.fasta

./group262/reference/d1c4zd_-d1j7da_.fasta

./group262/reference/d1i7ka_-d1j7da_.fasta

./group264/reference/d1j6ya_-d1m5ya2.fasta

./group264/reference/d1jnsa_-d1m5ya3.fasta

./group264/reference/d1ix5a_-d1l1pa_.fasta

./group264/reference/d1jvwa_-d1kt1a3.fasta

./group264/reference/d1l1pa_-d1pbk__.fasta

./group264/reference/d1hxva_-d1ix5a_.fasta

./group264/reference/d1ix5a_-d1jnsa_.fasta

./group264/reference/d1fd9a_-d1l1pa_.fasta

./group264/reference/d1eq3a_-d1m5ya2.fasta

./group264/reference/d1m5ya2-d1pina2.fasta

./group265/reference/d1goia3-d1hjxa2.fasta

./group265/reference/d1hjxa2-d1itxa2.fasta

./group265/reference/d1hjxa2-d1jnda2.fasta

./group265/reference/d1hjxa2-d1kfwa2.fasta

./group265/reference/d1goia3-d1jnda2.fasta

./group265/reference/d1goia3-d1ll7a2.fasta

./group265/reference/d1edqa3-d1hjxa2.fasta

./group265/reference/d1edqa3-d1goia3.fasta

./group265/reference/d1kfwa2-d1ll7a2.fasta

./group265/reference/d1jnda2-d1kfwa2.fasta

./group266/reference/d1f1ua1-d1kw3b2.fasta

./group266/reference/d1f1ua1-d1mpya1.fasta

./group266/reference/d1kw3b2-d1mpya1.fasta

./group266/reference/d1ecsa_-d1lqpa_.fasta

./group266/reference/d1f1ua2-d1mpya2.fasta

./group266/reference/d1f1ua1-d1qipa_.fasta

./group266/reference/d1lqpa_-d1qipa_.fasta

./group266/reference/d1cjxa1-d1qipa_.fasta

./group266/reference/d1kw3b2-d1mpya2.fasta

./group266/reference/d1ecsa_-d1qtoa_.fasta

./group270/reference/d1o4ua2-d1qapa2.fasta

./group270/reference/d1o4ua2-d1qpoa2.fasta

./group271/reference/d1buoa_-d1t1da_.fasta

./group271/reference/d1buoa_-d3kvt__.fasta

./group271/reference/d1fs1b2-d1hv2a_.fasta

./group272/reference/d1efub4-d1tfe__.fasta

./group273/reference/d1coja2-d1gv3a2.fasta

./group273/reference/d1coja2-d1kkca2.fasta

./group274/reference/d1qu6a1-d1stu__.fasta

./group274/reference/d1di2a_-d1qu6a1.fasta

./group274/reference/d1kn0a_-d1o0wa2.fasta

./group274/reference/d1di2a_-d1o0wa2.fasta

./group274/reference/d1fjge2-d1qu6a1.fasta

./group274/reference/d1di2a_-d1stu__.fasta

./group274/reference/d1o0wa2-d1pkp_2.fasta

./group274/reference/d1pkp_2-d1qu6a1.fasta

./group274/reference/d1o0wa2-d1stu__.fasta

./group274/reference/d1o0wa2-d1qu6a2.fasta

./group275/reference/d1k1ga_-d1khma_.fasta

./group275/reference/d1khma_-d2fmr__.fasta

./group275/reference/d1dtja_-d1j4wa1.fasta

./group275/reference/d1j4wa2-d1k1ga_.fasta

./group275/reference/d1j4wa2-d2fmr__.fasta

./group275/reference/d1j4wa1-d1khma_.fasta

./group275/reference/d1j4wa2-d1vig__.fasta

./group275/reference/d1j4wa1-d1vig__.fasta

./group275/reference/d1khma_-d1vig__.fasta

./group275/reference/d1j4wa1-d1j4wa2.fasta

./group276/reference/d1egaa2-d1fjgc1.fasta

./group276/reference/d1egaa2-d1hh2p2.fasta

./group276/reference/d1fjgc1-d1k0ra2.fasta

./group276/reference/d1k0ra2-d1k0ra3.fasta

./group277/reference/d1jpma2-d2mnr_2.fasta

./group277/reference/d1onea2-d2mnr_2.fasta

./group278/reference/d1fxra_-d1kqfb1.fasta

./group278/reference/d1blu__-d1keka5.fasta

./group278/reference/d1fxra_-d7fd1a_.fasta

./group278/reference/d1hfel2-d1jnrb_.fasta

./group278/reference/d1fxd__-d1h0hb_.fasta

./group278/reference/d1jnrb_-d1keka5.fasta

./group278/reference/d1fxra_-d1h0hb_.fasta

./group278/reference/d1fxra_-d1jnrb_.fasta

./group278/reference/d1feha3-d7fd1a_.fasta

./group278/reference/d1feha3-d1fxra_.fasta

./group279/reference/d1aye_2-d1jqga2.fasta

./group279/reference/d1aye_2-d1kwma2.fasta

./group279/reference/d1itpa_-d1scjb_.fasta

./group279/reference/d1jqga2-d1kwma2.fasta

./group283/reference/d1o0pa_-d2msta_.fasta

./group283/reference/d1fxla1-d1qm9a1.fasta

./group283/reference/d1nu4a_-d1o0pa_.fasta

./group283/reference/d1fjeb2-d1nu4a_.fasta

./group283/reference/d1cvja2-d1u2fa_.fasta

./group283/reference/d1b7fa2-d1hd1a_.fasta

./group283/reference/d1l3ka1-d1o0pa_.fasta

./group283/reference/d1cvja2-d2msta_.fasta

./group283/reference/d1u2fa_-d2u1a__.fasta

./group283/reference/d1oo0b_-d2u2fa_.fasta

./group285/reference/d1aw0__-d1k0va_.fasta

./group285/reference/d1cc8a_-d1jwwa_.fasta

./group285/reference/d1fvqa_-d1jwwa_.fasta

./group285/reference/d1aw0__-d1fvqa_.fasta

./group285/reference/d1cc8a_-d1k0va_.fasta

./group285/reference/d1afj__-d1fe0a_.fasta

./group285/reference/d1jwwa_-d1k0va_.fasta

./group285/reference/d1fe0a_-d1fvqa_.fasta

./group285/reference/d1afj__-d1aw0__.fasta

./group285/reference/d1cpza_-d1fvqa_.fasta

./group287/reference/d1kkha2-d1kvka2.fasta

./group290/reference/d1dm9a_-d1fjgd_.fasta

./group290/reference/d1dm9a_-d1h3fa2.fasta

./group290/reference/d1dm9a_-d1jh3a_.fasta

./group290/reference/d1fjgd_-d1jh3a_.fasta

./group296/reference/d1ck9a_-d1jj2f_.fasta

./group298/reference/d1j5pa3-d1nvmb2.fasta

./group299/reference/d1feca3-d1lvl_3.fasta

./group299/reference/d1dxla3-d3grs_3.fasta

./group299/reference/d1h6va3-d1mo9a3.fasta

./group299/reference/d1mo9a3-d3lada3.fasta

./group299/reference/d1mo9a3-d3grs_3.fasta

./group299/reference/d1h6va3-d1ojt_3.fasta

./group299/reference/d1ebda3-d1mo9a3.fasta

./group299/reference/d1lvl_3-d3grs_3.fasta

./group299/reference/d1h6va3-d3lada3.fasta

./group299/reference/d1nhp_3-d1ojt_3.fasta

./group300/reference/d1jroa3-d1n62c1.fasta

./group302/reference/d1f5va_-d1icra_.fasta

./group302/reference/d1f5va_-d1nox__.fasta

./group302/reference/d1icra_-d1nox__.fasta

./group302/reference/d1nox__-d1vfra_.fasta

./group303/reference/d1ast__-d1g12a_.fasta

./group303/reference/d1ast__-d1kapp2.fasta

./group303/reference/d1eb6a_-d1k9xa_.fasta

./group303/reference/d1eb6a_-d1i1ip_.fasta

./group303/reference/d1eb6a_-d1g12a_.fasta

./group303/reference/d1jk3a_-d1k7ia2.fasta

./group303/reference/d1ast__-d1k7ia2.fasta

./group303/reference/d1gkda_-d1kapp2.fasta

./group303/reference/d1c7ka_-d1jk3a_.fasta

./group303/reference/d1c7ka_-d1kapp2.fasta

./group304/reference/d1h41a2-d1jaka2.fasta

./group304/reference/d1h41a2-d1qba_4.fasta

./group304/reference/d1jaka2-d1qba_4.fasta

./group305/reference/d1ayaa_-d1mil__.fasta

./group305/reference/d1ju5a_-d1m61a_.fasta

./group305/reference/d1d4ta_-d1jwoa_.fasta

./group305/reference/d1d4ta_-d1opka2.fasta

./group305/reference/d1a81a1-d1d4ta_.fasta

./group305/reference/d1a09a_-d2plda_.fasta

./group305/reference/d1fu6a_-d2plda_.fasta

./group305/reference/d1ayaa_-d1m61a_.fasta

./group305/reference/d1ju5a_-d2cbla3.fasta

./group305/reference/d1d4ta_-d1lkka_.fasta

./group308/reference/d1b9la_-d1dhn__.fasta

./group309/reference/d12asa_-d1b8aa2.fasta

./group309/reference/d12asa_-d1eova2.fasta

./group309/reference/d1eova2-d1jjcb5.fasta

./group309/reference/d1jjca_-d1jjcb5.fasta

./group310/reference/d1mk4a_-d1qsma_.fasta

./group310/reference/d1n71a_-d1qsma_.fasta

./group311/reference/d1d0na5-d1jhwa3.fasta

./group311/reference/d1d0na5-d1d0na6.fasta

./group311/reference/d1d0na6-d1d4xg_.fasta

./group311/reference/d1d0na6-d1jhwa3.fasta

./group311/reference/d1ak7__-d1m4ja_.fasta

./group311/reference/d1f7sa_-d1m4ja_.fasta

./group311/reference/d1d0na3-d1d0na5.fasta

./group311/reference/d1d0na4-d1d0na5.fasta

./group311/reference/d1ak7__-d1hqz1_.fasta

./group311/reference/d1d4xg_-d1jhwa3.fasta

./group312/reference/d1acf__-d1pne__.fasta

./group312/reference/d1pne__-d1ypra_.fasta

./group313/reference/d1mc0a1-d1mc0a2.fasta

./group314/reference/d1ew0a_-d1jnua_.fasta

./group314/reference/d1ew0a_-d1n9la_.fasta

./group314/reference/d1ll8a_-d1lswa_.fasta

./group314/reference/d1lswa_-d1n9la_.fasta

./group315/reference/d1a3aa_-d1a6ja_.fasta

./group316/reference/d1hzta_-d1ktga_.fasta

./group316/reference/d1hzta_-d1mut__.fasta

./group316/reference/d1jkna_-d1k2ea_.fasta

./group316/reference/d1jkna_-d1ktga_.fasta

./group316/reference/d1jkna_-d1mut__.fasta

./group316/reference/d1ktga_-d1mut__.fasta

./group317/reference/d1b5ea_-d1bkpa_.fasta

./group317/reference/d1b5ea_-d1tis__.fasta

./group319/reference/d1bxda_-d1ei1a2.fasta

./group319/reference/d1i58a_-d1kija2.fasta

./group319/reference/d1ei1a2-d1i58a_.fasta

./group319/reference/d1i58a_-d1id0a_.fasta

./group319/reference/d1ei1a2-d1l0oa_.fasta

./group319/reference/d1ei1a2-d1id0a_.fasta

./group319/reference/d1bxda_-d1i58a_.fasta

./group319/reference/d1bxda_-d1l0oa_.fasta

./group319/reference/d1b63a2-d1l0oa_.fasta

./group319/reference/d1kija2-d1l0oa_.fasta

./group320/reference/d1bola_-d1iooa_.fasta

./group320/reference/d1bola_-d1iqqa_.fasta

./group320/reference/d1iooa_-d1iqqa_.fasta

./group320/reference/d1iooa_-d1ucaa_.fasta

./group322/reference/d1az9_2-d1c22a_.fasta

./group322/reference/d1az9_2-d1chma2.fasta

./group322/reference/d1az9_2-d1o0xa_.fasta

./group322/reference/d1c22a_-d1chma2.fasta

./group322/reference/d1chma2-d1o0xa_.fasta

./group324/reference/d1aisa1-d1ko9a2.fasta

./group328/reference/d1iz5a2-d1plq_2.fasta

./group328/reference/d1iz5a2-d2pola3.fasta

./group329/reference/d1fo4a5-d1n62b2.fasta

./group329/reference/d1hlra4-d1n62b2.fasta

./group330/reference/d1ckv__-d1hqi__.fasta

./group331/reference/d1jj2e1-d1rl6a1.fasta

./group331/reference/d1jj2e2-d1rl6a1.fasta

./group331/reference/d1jj2e2-d1rl6a2.fasta

./group332/reference/d1a9xa6-d1e4ea2.fasta

./group332/reference/d1a9xa6-d1iow_2.fasta

./group332/reference/d1ehia2-d1iow_2.fasta

./group333/reference/d1apme_-d1b6cb_.fasta

./group333/reference/d1koba_-d1opja_.fasta

./group333/reference/d1m2ra_-d1p4oa_.fasta

./group333/reference/d1b6cb_-d1p4oa_.fasta

./group333/reference/d1apme_-d1m2ra_.fasta

./group333/reference/d1blxa_-d1opja_.fasta

./group333/reference/d1apme_-d1k2pa_.fasta

./group333/reference/d1p4oa_-d1phk__.fasta

./group333/reference/d1apme_-d1csn__.fasta

./group333/reference/d1b6cb_-d1k2pa_.fasta

./group334/reference/d1hska1-d1uxy_1.fasta

./group334/reference/d1i19a2-d1n62c2.fasta

./group334/reference/d1jroa4-d1n62c2.fasta

./group335/reference/d1f7la_-d1qr0a2.fasta

./group335/reference/d1ftha_-d1qr0a2.fasta

./group336/reference/d1ryp1_-d1rypa_.fasta

./group336/reference/d1pmaa_-d1ryp1_.fasta

./group336/reference/d1iruk_-d1rypb_.fasta

./group336/reference/d1iruk_-d1j2qh_.fasta

./group336/reference/d1ryp1_-d1ryph_.fasta

./group336/reference/d1rypb_-d1rypi_.fasta

./group336/reference/d1ecfa2-d1gdoa_.fasta

./group336/reference/d1rypf_-d1rypk_.fasta

./group336/reference/d1j2qh_-d1rypj_.fasta

./group336/reference/d1rypf_-d1rypi_.fasta

./group337/reference/d1a7ta_-d1m2xa_.fasta

./group337/reference/d1a7ta_-d1qh5a_.fasta

./group338/reference/d1g5ba_-d1jk7a_.fasta

./group338/reference/d1g5ba_-d1utea_.fasta

./group338/reference/d1ii7a_-d1nnwa_.fasta

./group338/reference/d1ii7a_-d1utea_.fasta

./group339/reference/d1a5z_2-d7mdha2.fasta

./group339/reference/d1ez4a2-d7mdha2.fasta

./group339/reference/d1llda2-d1o6za2.fasta

./group339/reference/d1llda2-d2cmd_2.fasta

./group339/reference/d1guya2-d7mdha2.fasta

./group339/reference/d1ez4a2-d2cmd_2.fasta

./group339/reference/d1ceqa2-d1ldna2.fasta

./group339/reference/d1ceqa2-d5mdha2.fasta

./group339/reference/d1ldna2-d7mdha2.fasta

./group339/reference/d1i0za2-d7mdha2.fasta

./group340/reference/d1abra_-d1d6aa_.fasta

./group340/reference/d1abra_-d1qi7a_.fasta

./group340/reference/d1d6aa_-d1ggpa_.fasta

./group340/reference/d1d6aa_-d1hwma_.fasta

./group340/reference/d1d6aa_-d1m2ta_.fasta

./group340/reference/d1ggpa_-d1qi7a_.fasta

./group340/reference/d1hwma_-d1qi7a_.fasta

./group340/reference/d1ift__-d1qi7a_.fasta

./group340/reference/d1m2ta_-d1qi7a_.fasta

./group340/reference/d1mrj__-d1qi7a_.fasta

./group341/reference/d1a26_2-d1f0la2.fasta

./group341/reference/d1a26_2-d1ikpa2.fasta

./group341/reference/d1f0la2-d1gxya_.fasta

./group341/reference/d1f0la2-d1ikpa2.fasta

./group341/reference/d1giqa1-d1qs1a1.fasta

./group341/reference/d1giqa1-d1qs1a2.fasta

./group341/reference/d1giqa2-d1qs1a1.fasta

./group341/reference/d1qs1a1-d1qs1a2.fasta

./group342/reference/d1chua3-d1qo8a3.fasta

./group342/reference/d1kssa3-d1qlaa3.fasta

./group342/reference/d1neka3-d1qo8a3.fasta

./group343/reference/d1g1ta1-d1tsg__.fasta

./group343/reference/d1b6e__-d1byfa_.fasta

./group343/reference/d1k9ja_-d1tn3__.fasta

./group343/reference/d1b08a1-d1jzna_.fasta

./group343/reference/d1hq8a_-d1qdda_.fasta

./group343/reference/d1e87a_-d1hq8a_.fasta

./group343/reference/d1hq8a_-d1jwib_.fasta

./group343/reference/d1b08a1-d1j34a_.fasta

./group343/reference/d1g1ta1-d1j34b_.fasta

./group343/reference/d1jzna_-d1tn3__.fasta

./group346/reference/d1bsg__-d1e25a_.fasta

./group346/reference/d1e25a_-d1mfoa_.fasta

./group346/reference/d1e25a_-d1g6aa_.fasta

./group346/reference/d1e25a_-d4blma_.fasta

./group346/reference/d1ci9a_-d1mfoa_.fasta

./group346/reference/d1buea_-d1e25a_.fasta

./group346/reference/d1es5a_-d1mfoa_.fasta

./group346/reference/d1es5a_-d1ghpa_.fasta

./group346/reference/d1e25a_-d1iyoa_.fasta

./group346/reference/d1e25a_-d1m40a_.fasta

./group348/reference/d1buca2-d1is2a3.fasta

./group348/reference/d1is2a3-d1jqia2.fasta

./group348/reference/d1is2a3-d3mdda2.fasta

./group349/reference/d1g0ha_-d1ka1a_.fasta

./group349/reference/d1g0ha_-d2hhma_.fasta

./group349/reference/d1inp__-d1ka1a_.fasta

./group349/reference/d1inp__-d2hhma_.fasta

./group349/reference/d1ka1a_-d1lbva_.fasta

./group349/reference/d1ka1a_-d2hhma_.fasta

./group349/reference/d1lbva_-d2hhma_.fasta

./group351/reference/d1daaa_-d1iyea_.fasta

./group351/reference/d1ekfa_-d1iyea_.fasta

./group352/reference/d1k1ea_-d1l6ra_.fasta

./group352/reference/d1l6ra_-d1qq5a_.fasta

./group352/reference/d1o08a_-d1qq5a_.fasta

./group354/reference/d1a87__-d1cii_1.fasta

./group354/reference/d1cii_1-d1cola_.fasta

./group355/reference/d1ciy_3-d1i5pa3.fasta

./group355/reference/d1i5pa3-d1ji6a3.fasta

./group356/reference/d1ddba_-d1o0la_.fasta

./group356/reference/d1f16a_-d1k3ka_.fasta

./group356/reference/d1f16a_-d1o0la_.fasta

./group359/reference/d1a0tp_-d2mpra_.fasta

./group359/reference/d2por__-d3prn__.fasta

./group361/reference/d1agg__-d1nixa_.fasta

./group361/reference/d1cixa_-d1i25a_.fasta

./group361/reference/d1axh__-d1eit__.fasta

./group361/reference/d1koza_-d1qk7a_.fasta

./group361/reference/d1agg__-d1qk6a_.fasta

./group361/reference/d1i26a_-d1qk6a_.fasta

./group361/reference/d1nixa_-d1vtx__.fasta

./group361/reference/d1axh__-d1nixa_.fasta

./group361/reference/d1i26a_-d1lupa_.fasta

./group361/reference/d1agg__-d1koza_.fasta

./group362/reference/d1myn__-d1qkya_.fasta

./group362/reference/d1jxca_-d1sis__.fasta

./group362/reference/d1npia_-d1qkya_.fasta

./group362/reference/d1cmr__-d1npia_.fasta

./group362/reference/d1bcg__-d1sco__.fasta

./group362/reference/d1aho__-d1jkza_.fasta

./group362/reference/d1nrb__-d1tsk__.fasta

./group362/reference/d1fjna_-d1qkya_.fasta

./group362/reference/d1gps__-d1jxca_.fasta

./group362/reference/d1ne5a_-d1tsk__.fasta

./group363/reference/d1h59b_-d1nqla3.fasta

./group363/reference/d1igra3-d1nqla4.fasta

./group363/reference/d1m6ba4-d1nqla3.fasta

./group363/reference/d1n8yc4-d1nqla3.fasta

./group363/reference/d1nqla3-d1nqla4.fasta

./group364/reference/d1imt_1-d1lpba1.fasta

./group364/reference/d1imt_1-d1lpba2.fasta

./group364/reference/d1imt_2-d1lpba2.fasta

./group364/reference/d1lpba1-d1lpba2.fasta

./group365/reference/d1hz8a2-d1ijqa2.fasta

./group365/reference/d1hae__-d1klo_3.fasta

./group365/reference/d1fjsl_-d1xdtr_.fasta

./group365/reference/d1jl9a_-d1klo_3.fasta

./group365/reference/d1b9wa1-d1l3ya_.fasta

./group365/reference/d1hz8a2-d1urk_1.fasta

./group365/reference/d1ijqa2-d3tgf__.fasta

./group365/reference/d1klil_-d1klo_3.fasta

./group365/reference/d1hz8a1-d1klo_3.fasta

./group365/reference/d1klo_3-d1l3ya_.fasta

./group367/reference/d1f94a_-d1kbaa_.fasta

./group367/reference/d1f94a_-d1m9za_.fasta

./group367/reference/d1f94a_-d3ebx__.fasta

./group367/reference/d1fas__-d1m9za_.fasta

./group367/reference/d1ff4a_-d1m9za_.fasta

./group367/reference/d1ff4a_-d3ebx__.fasta

./group367/reference/d1hc9a_-d1m9za_.fasta

./group367/reference/d1kbaa_-d1m9za_.fasta

./group367/reference/d1m9za_-d1tfs__.fasta

./group367/reference/d1m9za_-d1tgxa_.fasta

./group368/reference/d1bf0__-d1tfxc_.fasta

./group368/reference/d1bunb_-d1tocr2.fasta

./group368/reference/d1d0da_-d1g6xa_.fasta

./group368/reference/d1bik_1-d1d0da_.fasta

./group368/reference/d1ktha_-d1tocr2.fasta

./group368/reference/d1aapa_-d1tocr2.fasta

./group368/reference/d1bunb_-d1tfxc_.fasta

./group368/reference/d1bik_2-d1tocr1.fasta

./group368/reference/d1bik_1-d1bunb_.fasta

./group368/reference/d1bf0__-d1g6xa_.fasta

./group369/reference/d1b8wa_-d1fd3a_.fasta

./group369/reference/d1h5oa_-d1ijva_.fasta

./group369/reference/d1d6ba_-d2bds__.fasta

./group369/reference/d1b8wa_-d1kj6a_.fasta

./group369/reference/d1b8wa_-d1sh1__.fasta

./group369/reference/d1dfna_-d2bds__.fasta

./group369/reference/d1h5oa_-d1kj6a_.fasta

./group369/reference/d1ewsa_-d1ijva_.fasta

./group369/reference/d1dfna_-d1h5oa_.fasta

./group369/reference/d1fd3a_-d1h5oa_.fasta

./group372/reference/d1ejga_-d1nbla_.fasta

./group373/reference/d1i71a_-d1l6ja3.fasta

./group373/reference/d1kdu__-d1l6ja3.fasta

./group373/reference/d1l6ja3-d1pmla_.fasta

./group373/reference/d1bhta2-d1h8pa1.fasta

./group373/reference/d1h8pa2-d1kdu__.fasta

./group373/reference/d1ki0a3-d1l6ja5.fasta

./group373/reference/d1h8pa2-d1pmla_.fasta

./group373/reference/d1l6ja5-d5hpga_.fasta

./group373/reference/d1l6ja5-d1pmla_.fasta

./group373/reference/d1kdu__-d1ki0a3.fasta

./group374/reference/d1ce3a_-d1tbrr1.fasta

./group374/reference/d1iw4a_-d1ldtl_.fasta

./group374/reference/d1iw4a_-d1lr7a2.fasta

./group374/reference/d1iw4a_-d1sgpi_.fasta

./group374/reference/d1nuba3-d1pce__.fasta

./group374/reference/d1nuba3-d1tbrr1.fasta

./group374/reference/d1nuba3-d1tgsi_.fasta

./group376/reference/d1hcnb_-d1jpya_.fasta

./group376/reference/d1fl7b_-d2tgi__.fasta

./group376/reference/d1fl7b_-d1hcna_.fasta

./group376/reference/d1bnda_-d1hcna_.fasta

./group376/reference/d1fl7b_-d1lxia_.fasta

./group376/reference/d1fltv_-d1hcna_.fasta

./group376/reference/d1aoca_-d1jpya_.fasta

./group376/reference/d1aoca_-d1bnda_.fasta

./group376/reference/d1hcna_-d2tgi__.fasta

./group376/reference/d1hcnb_-d1lxia_.fasta

./group377/reference/d1g40a1-d1gpza2.fasta

./group377/reference/d1g40a3-d1quba5.fasta

./group377/reference/d1g40a3-d1h03p1.fasta

./group377/reference/d1hfi__-d1quba3.fasta

./group377/reference/d1quba1-d1quba3.fasta

./group377/reference/d1gkna2-d1nwva1.fasta

./group377/reference/d1ckla1-d1gkga2.fasta

./group377/reference/d1h03p1-d1hfi__.fasta

./group377/reference/d1g40a4-d1nwva1.fasta

./group377/reference/d1g40a1-d1gkna2.fasta

./group379/reference/d1atb__-d1hx2a_.fasta

./group379/reference/d1coua_-d1hx2a_.fasta

./group379/reference/d1eaic_-d1hx2a_.fasta

./group380/reference/d1d4va1-d1d4va3.fasta

./group380/reference/d1d4va1-d1exta2.fasta

./group380/reference/d1d4va1-d1oqdk_.fasta

./group380/reference/d1d4va1-d1oqek_.fasta

./group380/reference/d1d4va2-d1jmab1.fasta

./group380/reference/d1exta1-d1exta3.fasta

./group380/reference/d1exta2-d1jmab1.fasta

./group381/reference/d1e88a3-d1fbr_2.fasta

./group381/reference/d1e88a3-d1o9aa2.fasta

./group381/reference/d1fbr_1-d1tpg_2.fasta

./group381/reference/d1fbr_2-d1tpg_2.fasta

./group381/reference/d1o9aa1-d1o9aa2.fasta

./group381/reference/d1o9aa2-d1tpg_2.fasta

./group382/reference/d1hlqa_-d1isua_.fasta

./group382/reference/d1hpi__-d1isua_.fasta

./group382/reference/d1isua_-d1iuaa_.fasta

./group382/reference/d1isua_-d2hipa_.fasta

./group383/reference/d1tf3a1-d2glia2.fasta

./group383/reference/d1yuja_-d5znf__.fasta

./group383/reference/d1tf3a2-d2glia1.fasta

./group383/reference/d1tf3a3-d5znf__.fasta

./group383/reference/d1njqa_-d1tf3a2.fasta

./group383/reference/d1ncs__-d2glia4.fasta

./group383/reference/d1paa__-d1ubdc3.fasta

./group383/reference/d1tf3a1-d1ubdc3.fasta

./group383/reference/d1njqa_-d1paa__.fasta

./group383/reference/d1zfd__-d2glia1.fasta

./group385/reference/d1dsza_-d1g47a1.fasta

./group385/reference/d1g47a1-d1m3va1.fasta

./group385/reference/d1lo1a_-d1nypa2.fasta

./group385/reference/d1a7i_2-d1kb2a_.fasta

./group385/reference/d1g47a1-d7gata_.fasta

./group385/reference/d1g47a2-d1m3va2.fasta

./group385/reference/d1iml_2-d1kb2a_.fasta

./group385/reference/d1j2oa2-d1m3va1.fasta

./group385/reference/d1nypa1-d1nypa2.fasta

./group385/reference/d1g47a2-d7gata_.fasta

./group386/reference/d1dsva_-d1eska_.fasta

./group388/reference/d1d0qa_-d1i50i2.fasta

./group388/reference/d1d0qa_-d1pft__.fasta

./group388/reference/d1dl6a_-d1i50i1.fasta

./group388/reference/d1dl6a_-d1tfi__.fasta

./group388/reference/d1i50i1-d1i50i2.fasta

./group388/reference/d1i50i1-d1qyp__.fasta

./group388/reference/d1i50i2-d1qyp__.fasta

./group388/reference/d1i50i2-d1tfi__.fasta

./group388/reference/d1pft__-d1qyp__.fasta

./group388/reference/d1pft__-d1tfi__.fasta

./group389/reference/d1dxga_-d1h7va_.fasta

./group389/reference/d1dxga_-d1lkoa2.fasta

./group389/reference/d1dxga_-d1rb9__.fasta

./group389/reference/d1h7va_-d1ocrf_.fasta

./group390/reference/d1jj22_-d1nvha_.fasta

./group390/reference/d1jj2y_-d1jj2z_.fasta

./group391/reference/d1g25a_-d1iyma_.fasta

./group391/reference/d1g25a_-d1jm7a_.fasta

./group391/reference/d1jm7a_-d1rmd_2.fasta

./group391/reference/d1chc__-d1rmd_2.fasta

./group391/reference/d1chc__-d1fbva4.fasta

./group391/reference/d1jm7b_-d1rmd_2.fasta

./group391/reference/d1g25a_-d1ldjb_.fasta

./group391/reference/d1fbva4-d1ldjb_.fasta

./group391/reference/d1bor__-d1iyma_.fasta

./group391/reference/d1chc__-d1g25a_.fasta

./group392/reference/d1dmc__-d1m0ga_.fasta

./group392/reference/d1dmc__-d1qjka_.fasta

./group392/reference/d1fmya_-d1m0ga_.fasta

./group392/reference/d1fmya_-d1qjka_.fasta

./group392/reference/d1jjda_-d1qjka_.fasta

./group392/reference/d1m0ga_-d1qjka_.fasta

./group393/reference/d1e53a_-d1ptq__.fasta

./group393/reference/d1kbea_-d1ptq__.fasta

./group394/reference/d1dvpa2-d1f62a_.fasta

./group394/reference/d1f62a_-d1joca1.fasta

./group394/reference/d1f62a_-d1vfya_.fasta

./group394/reference/d1joca1-d1mm2a_.fasta

./group396/reference/d1cqxa2-d1kzla2.fasta

./group396/reference/d1a8p_1-d2cnd_1.fasta

./group396/reference/d1ddga1-d1jb9a1.fasta

./group396/reference/d1i7pa1-d1que_1.fasta

./group396/reference/d1a8p_1-d1ddga1.fasta

./group396/reference/d1que_1-d2cnd_1.fasta

./group396/reference/d1ep3b1-d2pia_1.fasta

./group396/reference/d1a8p_1-d2pia_1.fasta

./group396/reference/d1ep3b1-d2cnd_1.fasta

./group396/reference/d1f20a1-d1i7pa1.fasta

./group397/reference/d1ezva2-d1hr6a2.fasta

./group397/reference/d1hr6a2-d1hr6b2.fasta

./group397/reference/d1hr6a2-d1l0la2.fasta

./group397/reference/d1ezva1-d1hr6b1.fasta

./group397/reference/d1hr6a1-d1l0lb1.fasta

./group397/reference/d1ezvb1-d1hr6a1.fasta

./group397/reference/d1hr6b2-d1l0la2.fasta

./group397/reference/d1ezva2-d1l0lb2.fasta

./group397/reference/d1hr6a1-d1l0la1.fasta

./group397/reference/d1ezvb1-d1l0lb1.fasta

./group398/reference/d1gjja1-d1gjja2.fasta

./group400/reference/d1mhna_-d1oi1a1.fasta

./group400/reference/d1mhna_-d1oi1a2.fasta

./group401/reference/d1dcja_-d1jdqa_.fasta

./group401/reference/d1dcja_-d1je3a_.fasta

./group401/reference/d1dcja_-d1pava_.fasta

./group401/reference/d1jdqa_-d1je3a_.fasta

./group403/reference/d1hc7a3-d1nj8a2.fasta

./group404/reference/d1h1js_-d1kcfa1.fasta

./group406/reference/d1jida_-d1lnga_.fasta

./group407/reference/d1cb8a3-d1hn0a4.fasta

./group407/reference/d1cb8a3-d1n7oa3.fasta

./group407/reference/d1hn0a4-d1j0ma3.fasta

./group407/reference/d1hn0a4-d1n7oa3.fasta

./group408/reference/d1jjua4-d1ktja_.fasta

./group408/reference/d1g0da1-d1l9na1.fasta

./group408/reference/d1a02n1-d1my7a_.fasta

./group408/reference/d1bf2_1-d1eut_1.fasta

./group408/reference/d1imhc1-d1my7a_.fasta

./group408/reference/d1j0ha1-d1ji1a1.fasta

./group408/reference/d1m7xa1-d1soxa1.fasta

./group408/reference/d1bf2_1-d1m7xa1.fasta

./group408/reference/d1ktja_-d1nepa_.fasta

./group408/reference/d1g0da1-d1ktja_.fasta

./group411/reference/d1k4cc_-d1lnqa2.fasta

./group411/reference/d1lnqa2-d1p7ba2.fasta

./group414/reference/d1m56b2-d1ocrb2.fasta

./group416/reference/d1ee8a2-d1k3xa2.fasta

./group416/reference/d1k3xa2-d1k82a2.fasta

./group416/reference/d1k3xa2-d1l1za2.fasta

./group416/reference/d1k3xa2-d1nnja2.fasta

./group417/reference/d1m9sa2-d1m9sa3.fasta

./group417/reference/d1m9sa3-d1m9sa4.fasta

./group418/reference/d1h3ia2-d1mvha_.fasta

./group418/reference/d1h3ia2-d1n3ja_.fasta

./group418/reference/d1mvha_-d1n3ja_.fasta

./group419/reference/d1opoa_-d1smva_.fasta

./group419/reference/d1bev3_-d1qqp3_.fasta

./group419/reference/d1c8na_-d1ng0a_.fasta

./group419/reference/d1bev1_-d1tmf1_.fasta

./group419/reference/d1pvc3_-d1qqp3_.fasta

./group419/reference/d1pvc3_-d1tmf1_.fasta

./group419/reference/d1ng0a_-d1opoa_.fasta

./group419/reference/d1f2na_-d1smva_.fasta

./group419/reference/d1ng0a_-d1smva_.fasta

./group419/reference/d1ihma_-d1pvc3_.fasta

./group420/reference/d1c8da_-d1lp3a_.fasta

./group421/reference/d1dzla_-d1sva1_.fasta

./group423/reference/d1iw7f1-d1l0oc_.fasta

./group423/reference/d1iw7f1-d1or7a1.fasta

./group423/reference/d1ku3a_-d1or7a1.fasta

./group425/reference/d1iw7f3-d1or7a2.fasta
