# Supplementary material for: PR2ALIGN: a stand-alone software program and a web-server for protein sequence alignment using weighted biochemical properties of amino acids
Source: BMC Res Notes. 2015 May 7;8:187. doi: 10.1186/s13104-015-1152-6 (PMC4477417; doi:10.1186/s13104-015-1152-6)
Supplement: Additional file 4: — SABmark SUP sequence pairs for 30-40% sequence identity range used to optimize property weights and gap penalties. Maximum of 10 randomly sampled pairs per superfamily. [file 13104_2015_1152_MOESM4_ESM.docx]

SABmark SUP sequence pairs for 30-40% sequence identity range used to optimize property weights and gap penalties.

Maximum of 10 randomly sampled pairs per superfamily.

./group1/reference/d1alla_-d1jboa_.fasta

./group1/reference/d1gcva_-d2lhb__.fasta

./group1/reference/d1alla_-d1b8da_.fasta

./group1/reference/d1mba__-d2lhb__.fasta

./group1/reference/d1cg5b_-d1irda_.fasta

./group1/reference/d1gcva_-d1irdb_.fasta

./group1/reference/d1cg5b_-d1irdb_.fasta

./group1/reference/d1alla_-d1allb_.fasta

./group1/reference/d1cg5a_-d1irdb_.fasta

./group1/reference/d1irda_-d2lhb__.fasta

./group2/reference/d1kf6b1-d1nekb1.fasta

./group5/reference/d1bsma1-d1qnna1.fasta

./group5/reference/d1b06a1-d1coja1.fasta

./group5/reference/d1coja1-d1qnna1.fasta

./group5/reference/d1ap6a1-d1b06a1.fasta

./group5/reference/d1b06a1-d1my6a1.fasta

./group5/reference/d1b06a1-d1gv3a1.fasta

./group5/reference/d1ap6a1-d1qnna1.fasta

./group5/reference/d1gv3a1-d1qnna1.fasta

./group5/reference/d1gv3a1-d1my6a1.fasta

./group5/reference/d1ix9a1-d1my6a1.fasta

./group6/reference/d1ql3a_-d1ycc__.fasta

./group6/reference/d1etpa2-d1qn2a_.fasta

./group6/reference/d1dw0a_-d1gu2a_.fasta

./group6/reference/d1e29a_-d3c2c__.fasta

./group6/reference/d1dw0a_-d3c2c__.fasta

./group6/reference/d1hroa_-d3c2c__.fasta

./group6/reference/d1cxc__-d1ql3a_.fasta

./group6/reference/d1cxc__-d1eb7a1.fasta

./group6/reference/d1e29a_-d1ycc__.fasta

./group6/reference/d1f1ca_-d1ql3a_.fasta

./group7/reference/d1fexa_-d1igna1.fasta

./group7/reference/d1k61a_-d1qrya_.fasta

./group7/reference/d1fjla_-d1qrya_.fasta

./group7/reference/d1jgga_-d1k61a_.fasta

./group7/reference/d1bw5__-d1k61a_.fasta

./group7/reference/d1b72b_-d1fjla_.fasta

./group7/reference/d1bw5__-d1jgga_.fasta

./group7/reference/d1bw5__-d1fjla_.fasta

./group7/reference/d1e3oc1-d1jgga_.fasta

./group7/reference/d1e3oc1-d1ig7a_.fasta

./group8/reference/d1mgta1-d1sfe_1.fasta

./group10/reference/d1md0a_-d1puee_.fasta

./group11/reference/d1a04a1-d1fsea_.fasta

./group11/reference/d1fsea_-d1p4wa_.fasta

./group11/reference/d1gxqa_-d1opc__.fasta

./group13/reference/d1ee8a1-d1k82a1.fasta

./group13/reference/d1ee8a1-d1nnja1.fasta

./group13/reference/d1fjgm_-d1mu5a1.fasta

./group13/reference/d1k82a1-d1nnja1.fasta

./group13/reference/d1l1za1-d1nnja1.fasta

./group14/reference/d1exja1-d1jbga_.fasta

./group15/reference/d1cuna1-d1cuna2.fasta

./group16/reference/d1kf6a1-d1neka1.fasta

./group16/reference/d1kf6a1-d1qlaa1.fasta

./group16/reference/d1neka1-d1qlaa1.fasta

./group18/reference/d1erd__-d2erl__.fasta

./group21/reference/d1ckta_-d1lwma_.fasta

./group21/reference/d1i11a_-d1lwma_.fasta

./group21/reference/d1i11a_-d2lefa_.fasta

./group22/reference/d1b67a_-d1jfib_.fasta

./group22/reference/d1b67a_-d1n1ja_.fasta

./group22/reference/d1jfib_-d1n1ja_.fasta

./group23/reference/d1cpq__-d1gqaa_.fasta

./group23/reference/d1gqaa_-d1mqva_.fasta

./group24/reference/d1cgme_-d1ei7a_.fasta

./group24/reference/d1cgme_-d1rmva_.fasta

./group25/reference/d1ivha1-d1jqia1.fasta

./group25/reference/d1ivha1-d3mdda1.fasta

./group26/reference/d1jgca_-d1nfva_.fasta

./group26/reference/d1ji4a_-d1jiga_.fasta

./group26/reference/d1ji4a_-d1qgha_.fasta

./group28/reference/d1f7ua1-d1iq0a1.fasta

./group29/reference/d1af8__-d1l0ia_.fasta

./group29/reference/d1klpa_-d1l0ia_.fasta

./group30/reference/d1qrjb1-d2eiaa1.fasta

./group32/reference/d1eqfa2-d1f68a_.fasta

./group33/reference/d1adr__-d1r69__.fasta

./group33/reference/d1adr__-d1zug__.fasta

./group33/reference/d1b0na2-d1r69__.fasta

./group33/reference/d1b0na2-d1uxd__.fasta

./group33/reference/d1lmb3_-d1r69__.fasta

./group33/reference/d1uxd__-d1vpwa1.fasta

./group34/reference/d1nkpa_-d1nkpb_.fasta

./group34/reference/d1am9a_-d1nkpb_.fasta

./group34/reference/d1am9a_-d1an4a_.fasta

./group34/reference/d1nkpa_-d1nlwa_.fasta

./group34/reference/d1mdya_-d1nkpa_.fasta

./group34/reference/d1a0aa_-d1an4a_.fasta

./group34/reference/d1am9a_-d1mdya_.fasta

./group34/reference/d1an4a_-d1nlwa_.fasta

./group34/reference/d1nkpb_-d1nlwa_.fasta

./group34/reference/d1a0aa_-d1nkpa_.fasta

./group35/reference/d1k94a_-d1wdcc_.fasta

./group35/reference/d1alva_-d1k94a_.fasta

./group35/reference/d1f8ha_-d1qjta_.fasta

./group35/reference/d1auib_-d1dgua_.fasta

./group35/reference/d1jbaa_-d1ncx__.fasta

./group35/reference/d1exra_-d2pvba_.fasta

./group35/reference/d1auib_-d2pvba_.fasta

./group35/reference/d1auib_-d1wdcc_.fasta

./group35/reference/d1exra_-d1ncx__.fasta

./group35/reference/d1dgua_-d1exra_.fasta

./group36/reference/d1aoa_1-d1mb8a1.fasta

./group38/reference/d1a0fa1-d1f2ea1.fasta

./group38/reference/d1axda1-d1gnwa1.fasta

./group38/reference/d1duga1-d1glqa1.fasta

./group38/reference/d1duga1-d2gsta1.fasta

./group38/reference/d1f2ea1-d1pmt_1.fasta

./group38/reference/d1glqa1-d1gula1.fasta

./group38/reference/d1glqa1-d1k3ya1.fasta

./group38/reference/d1iyha1-d2gsq_1.fasta

./group39/reference/d1khda1-d1o17a1.fasta

./group43/reference/d1hlra1-d1n62a1.fasta

./group45/reference/d1cuk_2-d1ixra1.fasta

./group45/reference/d1cuk_2-d1kfta_.fasta

./group45/reference/d1ixra1-d1kfta_.fasta

./group48/reference/d1l9la_-d1nkl__.fasta

./group49/reference/d1axn__-d1n00a_.fasta

./group49/reference/d1bo9a_-d1n00a_.fasta

./group49/reference/d1dm5a_-d1n00a_.fasta

./group49/reference/d1hm6a_-d1n00a_.fasta

./group52/reference/d1aisb1-d1vola1.fasta

./group52/reference/d1bu2a1-d1g3nc1.fasta

./group52/reference/d1f5qb1-d1g3nc1.fasta

./group52/reference/d1g3nc1-d1vin_1.fasta

./group56/reference/d1agre_-d1dk8a_.fasta

./group56/reference/d1cmza_-d1dk8a_.fasta

./group57/reference/d1jdra_-d1mwva1.fasta

./group57/reference/d1mn2__-d1oafa_.fasta

./group57/reference/d1mwva1-d1oafa_.fasta

./group59/reference/d1dnpa1-d1np7a1.fasta

./group59/reference/d1iqra1-d1np7a1.fasta

./group59/reference/d1np7a1-d1qnf_1.fasta

./group60/reference/d1np3a1-d1qmga1.fasta

./group63/reference/d1dceb_-d1ld8b_.fasta

./group64/reference/d1a59__-d1k3pa_.fasta

./group64/reference/d1a59__-d1o7xa_.fasta

./group64/reference/d1aj8a_-d1k3pa_.fasta

./group64/reference/d1ioma_-d1k3pa_.fasta

./group64/reference/d1k3pa_-d1o7xa_.fasta

./group65/reference/d1cpt__-d1jfba_.fasta

./group65/reference/d1io7a_-d1jipa_.fasta

./group65/reference/d1jfba_-d1jipa_.fasta

./group68/reference/d1b3ua_-d1h6ka1.fasta

./group69/reference/d1awcb_-d1bd8__.fasta

./group69/reference/d1awcb_-d1myo__.fasta

./group69/reference/d1bd8__-d1myo__.fasta

./group69/reference/d1bi7b_-d1ihba_.fasta

./group70/reference/d1a17__-d1elra_.fasta

./group70/reference/d1a17__-d1elwa_.fasta

./group70/reference/d1elra_-d1elwa_.fasta

./group72/reference/d1fcya_-d1kv6a_.fasta

./group72/reference/d1fcya_-d1n83a_.fasta

./group72/reference/d1fcya_-d1pdua_.fasta

./group72/reference/d1kv6a_-d1pk5a_.fasta

./group73/reference/d1ah7__-d1ca1_1.fasta

./group74/reference/d1n5ua1-d1n5ua2.fasta

./group75/reference/d1fura_-d1j3ua_.fasta

./group76/reference/d1lfja_-d1psj__.fasta

./group76/reference/d1buna_-d1kvoa_.fasta

./group76/reference/d1aokb_-d1le6a_.fasta

./group76/reference/d1kvoa_-d1poc__.fasta

./group76/reference/d1kvoa_-d1lfja_.fasta

./group76/reference/d1le6a_-d1psj__.fasta

./group76/reference/d1g4ia_-d1le6a_.fasta

./group76/reference/d1le6a_-d1mc2a_.fasta

./group76/reference/d1kvoa_-d1le6a_.fasta

./group76/reference/d1lfja_-d1mc2a_.fasta

./group77/reference/d1aqe__-d2cy3__.fasta

./group77/reference/d1hh5a_-d2cy3__.fasta

./group77/reference/d1gyoa_-d1qo8a1.fasta

./group77/reference/d2ctha_-d2cy3__.fasta

./group77/reference/d1aqe__-d3caoa_.fasta

./group77/reference/d19hca_-d1aqe__.fasta

./group77/reference/d19hca_-d2cy3__.fasta

./group77/reference/d1aqe__-d2ctha_.fasta

./group77/reference/d1hh5a_-d1qo8a1.fasta

./group77/reference/d1fs7a_-d1m1qa_.fasta

./group78/reference/d1ogae1-d1tvda_.fasta

./group78/reference/d1nfdb1-d2rhe__.fasta

./group78/reference/d1ogad1-d2rhe__.fasta

./group78/reference/d1tvda_-d2rhe__.fasta

./group78/reference/d1nfdb1-d1tvda_.fasta

./group78/reference/d1hxma1-d1tvda_.fasta

./group78/reference/d1b88a_-d2f5bh1.fasta

./group78/reference/d1nlbh1-d2f5bh1.fasta

./group78/reference/d1fo0a_-d1tvda_.fasta

./group78/reference/d1nfdb1-d1ogae1.fasta

./group79/reference/d1bpv__-d1cfb_1.fasta

./group79/reference/d1egja_-d1gh7a2.fasta

./group81/reference/d1f13a3-d1g0da3.fasta

./group82/reference/d1edha2-d1l3wa4.fasta

./group83/reference/d1akp__-d1noa__.fasta

./group83/reference/d1akp__-d2mcm__.fasta

./group83/reference/d1j48a_-d2mcm__.fasta

./group84/reference/d1do5a_-d1ej8a_.fasta

./group85/reference/d1gyva_-d1p4ua_.fasta

./group86/reference/d1cwva1-d1cwva2.fasta

./group86/reference/d1cwva1-d1f00i1.fasta

./group86/reference/d1cwva2-d1f00i1.fasta

./group87/reference/d1aoha_-d1g1ka_.fasta

./group90/reference/d1acz__-d1qhoa2.fasta

./group90/reference/d1j18a1-d1pama2.fasta

./group91/reference/d1dmha_-d1eo9b_.fasta

./group91/reference/d1eo9a_-d1eo9b_.fasta

./group92/reference/d1aoza3-d1gw0a3.fasta

./group92/reference/d1aoza3-d1hfua3.fasta

./group92/reference/d1fwxa1-d1kbva1.fasta

./group92/reference/d1gw0a1-d1kcw_1.fasta

./group92/reference/d1gw0a3-d1hfua3.fasta

./group92/reference/d1kbva2-d1oe1a2.fasta

./group93/reference/d1dqva1-d1dsya_.fasta

./group93/reference/d1dqva1-d1k5wa_.fasta

./group93/reference/d1dqva1-d3rpba_.fasta

./group93/reference/d1dsya_-d1k5wa_.fasta

./group93/reference/d1dsya_-d1rlw__.fasta

./group93/reference/d1dsya_-d3rpba_.fasta

./group93/reference/d1k5wa_-d1rlw__.fasta

./group94/reference/d1czya1-d1lb6a_.fasta

./group95/reference/d1h4ax1-d2bb2_1.fasta

./group95/reference/d1h4ax2-d2bb2_1.fasta

./group95/reference/d1h4ax2-d2bb2_2.fasta

./group95/reference/d1ha4a_-d2bb2_1.fasta

./group95/reference/d2bb2_1-d2bb2_2.fasta

./group98/reference/d1gmea_-d1shsa_.fasta

./group99/reference/d1ciy_1-d1ji6a1.fasta

./group99/reference/d1cx1a_-d1gu3a_.fasta

./group99/reference/d1eut_2-d1k3ia2.fasta

./group100/reference/d1ahsa_-d1bvp12.fasta

./group100/reference/d1jsda_-d2viua_.fasta

./group104/reference/d1g6ga_-d1lgpa_.fasta

./group104/reference/d1gxca_-d1lgpa_.fasta

./group105/reference/d1a3k__-d1bkza_.fasta

./group105/reference/d1a8d_1-d1epwa1.fasta

./group106/reference/d1g8kb_-d1jm1a_.fasta

./group106/reference/d1jm1a_-d1rfs__.fasta

./group106/reference/d1nyka_-d1rfs__.fasta

./group106/reference/d1rfs__-d1rie__.fasta

./group108/reference/d1jo8a_-d1pwt__.fasta

./group108/reference/d1gcqc_-d1oeba_.fasta

./group108/reference/d1bb9__-d1neb__.fasta

./group108/reference/d1h92a_-d1ng2a1.fasta

./group108/reference/d1k4us_-d1pht__.fasta

./group108/reference/d1gl5a_-d2hsp__.fasta

./group108/reference/d1fmk_1-d1jqqa_.fasta

./group108/reference/d1fmk_1-d2hsp__.fasta

./group108/reference/d1h92a_-d1oeba_.fasta

./group108/reference/d1oeba_-d2hsp__.fasta

./group109/reference/d1dj7b_-d1jb0e_.fasta

./group109/reference/d1ireb_-d2ahjb_.fasta

./group112/reference/d1d5ga_-d1mfga_.fasta

./group112/reference/d1qaua_-d1qlca_.fasta

./group112/reference/d1be9a_-d1qlca_.fasta

./group112/reference/d1mfga_-d1qlca_.fasta

./group112/reference/d1be9a_-d1nf3c_.fasta

./group112/reference/d1be9a_-d1qaua_.fasta

./group112/reference/d1i16__-d1qava_.fasta

./group112/reference/d1ihja_-d1qava_.fasta

./group112/reference/d1mfga_-d1qava_.fasta

./group112/reference/d1qaua_-d1qava_.fasta

./group113/reference/d1d3bb_-d1mgqa_.fasta

./group113/reference/d1h641_-d1ljoa_.fasta

./group113/reference/d1h641_-d1n9ra_.fasta

./group113/reference/d1i8fa_-d1ljoa_.fasta

./group113/reference/d1i8fa_-d1n9ra_.fasta

./group113/reference/d1ljoa_-d1mgqa_.fasta

./group113/reference/d1ljoa_-d1n9ra_.fasta

./group114/reference/d1an8_1-d1eu3a1.fasta

./group114/reference/d1enfa1-d1fnua1.fasta

./group114/reference/d1enfa1-d3seb_1.fasta

./group116/reference/d1fl0a_-d1pxfa_.fasta

./group116/reference/d1gd7a_-d1pxfa_.fasta

./group118/reference/d1fr3a_-d1guta_.fasta

./group118/reference/d1fr3a_-d1h9ma1.fasta

./group118/reference/d1fr3a_-d1h9ma2.fasta

./group118/reference/d1h9ma1-d1h9ra2.fasta

./group118/reference/d1h9ma2-d1h9ra1.fasta

./group118/reference/d1h9ma2-d1h9ra2.fasta

./group119/reference/d1bfg__-d1nuna_.fasta

./group119/reference/d1ijta_-d1nuna_.fasta

./group119/reference/d1ijta_-d1qqla_.fasta

./group120/reference/d1hwmb1-d1knma_.fasta

./group120/reference/d1hwmb2-d1m2tb2.fasta

./group120/reference/d1knma_-d1m2tb1.fasta

./group121/reference/d1a8d_2-d1epwa2.fasta

./group121/reference/d1a8d_2-d3btaa2.fasta

./group121/reference/d1avac_-d1avwb_.fasta

./group121/reference/d1avac_-d1eyla_.fasta

./group121/reference/d1epwa2-d3btaa2.fasta

./group121/reference/d1eyla_-d1wba__.fasta

./group124/reference/d1exma2-d1f60a2.fasta

./group126/reference/d1gvkb_-d1mzaa_.fasta

./group126/reference/d1ddja_-d1klih_.fasta

./group126/reference/d1eq9a_-d1gvkb_.fasta

./group126/reference/d1ddja_-d1gvkb_.fasta

./group126/reference/d1azza_-d1eaxa_.fasta

./group126/reference/d1ekbb_-d1gvkb_.fasta

./group126/reference/d1fjsa_-d1orfa_.fasta

./group126/reference/d1ltoa_-d2hlca_.fasta

./group126/reference/d1bqya_-d1rfna_.fasta

./group126/reference/d1mzaa_-d1sgt__.fasta

./group128/reference/d1dpja_-d1lf2a_.fasta

./group128/reference/d1dpja_-d2apr__.fasta

./group128/reference/d1fmb__-d1idaa_.fasta

./group128/reference/d1fmb__-d1kzka_.fasta

./group128/reference/d1fmb__-d4fiv__.fasta

./group128/reference/d1kzka_-d4fiv__.fasta

./group128/reference/d1mpp__-d2apr__.fasta

./group129/reference/d1ffya2-d1ile_2.fasta

./group129/reference/d1h3na2-d1ile_2.fasta

./group129/reference/d1h3na2-d1ivsa3.fasta

./group130/reference/d1eu1a1-d2napa1.fasta

./group130/reference/d1h0ha1-d2napa1.fasta

./group130/reference/d1kqfa1-d2napa1.fasta

./group131/reference/d1gg3a2-d1h4ra2.fasta

./group132/reference/d1a49a1-d1pkla1.fasta

./group132/reference/d1e0ta1-d1pkla1.fasta

./group133/reference/d1bj7__-d1dzka_.fasta

./group133/reference/d1ggla_-d1hms__.fasta

./group133/reference/d1gkab_-d1i4ua_.fasta

./group133/reference/d1cbs__-d1ftpa_.fasta

./group133/reference/d1ftpa_-d1mdc__.fasta

./group133/reference/d1cbs__-d1ggla_.fasta

./group133/reference/d1bj7__-d1ew3a_.fasta

./group133/reference/d1b56__-d1ggla_.fasta

./group133/reference/d1hms__-d1ifc__.fasta

./group133/reference/d1cbs__-d1ifc__.fasta

./group134/reference/d1a33__-d1lopa_.fasta

./group134/reference/d1lopa_-d2cpl__.fasta

./group135/reference/d1fbl_1-d1gen__.fasta

./group135/reference/d1gen__-d1itva_.fasta

./group135/reference/d1gen__-d1pex__.fasta

./group136/reference/d1f8ea_-d1nsca_.fasta

./group136/reference/d1n1ta2-d3sil__.fasta

./group136/reference/d1nsca_-d2bat__.fasta

./group138/reference/d1g5aa1-d1m53a1.fasta

./group139/reference/d1eg3a3-d1jmqa_.fasta

./group139/reference/d1eg3a3-d1o6wa2.fasta

./group139/reference/d1eg3a3-d1pina1.fasta

./group139/reference/d1i5hw_-d1o6wa2.fasta

./group139/reference/d1jmqa_-d1o6wa2.fasta

./group139/reference/d1o6wa2-d1pina1.fasta

./group140/reference/d1jd0a_-d1kopa_.fasta

./group140/reference/d1jd0a_-d1mooa_.fasta

./group140/reference/d1kopa_-d1mooa_.fasta

./group141/reference/d1ciy_2-d1ji6a2.fasta

./group143/reference/d1hg8a_-d1k5ca_.fasta

./group143/reference/d1jtaa_-d1qcxa_.fasta

./group144/reference/d1krra_-d1xat__.fasta

./group144/reference/d1ocxa_-d1xat__.fasta

./group145/reference/d1dzra_-d1nxma_.fasta

./group145/reference/d1ep0a_-d1nxma_.fasta

./group147/reference/d1cx4a1-d1rgs_2.fasta

./group147/reference/d1o7fa2-d1o7fa3.fasta

./group147/reference/d1o7fa3-d1rgs_2.fasta

./group147/reference/d1rgs_1-d1rgs_2.fasta

./group148/reference/d1dd2a_-d1qjoa_.fasta

./group148/reference/d1htp__-d1lac__.fasta

./group148/reference/d1fyc__-d1lac__.fasta

./group148/reference/d1k8ma_-d1qjoa_.fasta

./group148/reference/d1bdo__-d1fyc__.fasta

./group148/reference/d1fyc__-d1ghk__.fasta

./group148/reference/d1lac__-d1qjoa_.fasta

./group148/reference/d1k8ma_-d1lac__.fasta

./group148/reference/d1bdo__-d1dd2a_.fasta

./group148/reference/d1dd2a_-d1lac__.fasta

./group150/reference/d2f3ga_-d2gpr__.fasta

./group152/reference/d1dun__-d1mq7a_.fasta

./group152/reference/d1euwa_-d1mq7a_.fasta

./group152/reference/d1f7da_-d1mq7a_.fasta

./group154/reference/d1a53__-d1i4na_.fasta

./group154/reference/d1a53__-d1pii_1.fasta

./group154/reference/d1i4na_-d1pii_1.fasta

./group154/reference/d1nsj__-d1pii_2.fasta

./group155/reference/d1ep3a_-d2dora_.fasta

./group155/reference/d1o94a1-d1oyb__.fasta

./group156/reference/d1exba_-d1lqaa_.fasta

./group157/reference/d1qhoa4-d7taa_2.fasta

./group157/reference/d1j0ha3-d1pama4.fasta

./group157/reference/d1qvba_-d1ug6a_.fasta

./group157/reference/d1cbg__-d1ug6a_.fasta

./group157/reference/d1cbg__-d1e4ia_.fasta

./group157/reference/d1lwha2-d1uok_2.fasta

./group157/reference/d1j0ha3-d1uok_2.fasta

./group157/reference/d1lwha2-d1pama4.fasta

./group157/reference/d1e4mm_-d1ug6a_.fasta

./group157/reference/d1qhoa4-d1uok_2.fasta

./group158/reference/d1bf6a_-d1i0da_.fasta

./group159/reference/d1f74a_-d1hl2a_.fasta

./group159/reference/d1gzga_-d1ohla_.fasta

./group159/reference/d1jcla_-d1o0ya_.fasta

./group159/reference/d1jcla_-d1ub3a_.fasta

./group159/reference/d1mzha_-d1n7ka_.fasta

./group159/reference/d1n7ka_-d1o0ya_.fasta

./group159/reference/d1n7ka_-d1ub3a_.fasta

./group160/reference/d1jpdx1-d1jpma1.fasta

./group161/reference/d1dxea_-d1izca_.fasta

./group161/reference/d1f8ma_-d1muma_.fasta

./group163/reference/d1lucb_-d1nfp__.fasta

./group164/reference/d2plc__-d2ptd__.fasta

./group165/reference/d7reqa1-d7reqb1.fasta

./group166/reference/d1bgva1-d1hwxa1.fasta

./group166/reference/d1hdca_-d1hxha_.fasta

./group166/reference/d1fmca_-d1hdca_.fasta

./group166/reference/d1fmca_-d1gega_.fasta

./group166/reference/d1hxha_-d1iy8a_.fasta

./group166/reference/d1fmca_-d1hxha_.fasta

./group166/reference/d1h5qa_-d1hdca_.fasta

./group166/reference/d1eno__-d1fmca_.fasta

./group166/reference/d1g0oa_-d1iy8a_.fasta

./group166/reference/d1gega_-d1hdca_.fasta

./group167/reference/d1feca2-d1gesa2.fasta

./group167/reference/d1jeha2-d1ojt_2.fasta

./group167/reference/d1gesa2-d1lvl_2.fasta

./group167/reference/d1ebda2-d1lvl_2.fasta

./group167/reference/d1lvl_2-d1nhp_2.fasta

./group167/reference/d1lvl_2-d1ojt_2.fasta

./group167/reference/d1feca2-d1h6va2.fasta

./group167/reference/d1lvl_2-d3lada2.fasta

./group167/reference/d1fl2a2-d1vdc_2.fasta

./group167/reference/d1d7ya2-d1lvl_2.fasta

./group168/reference/d1dysa_-d1tml__.fasta

./group168/reference/d1oc7a_-d1tml__.fasta

./group170/reference/d1io0a_-d1pgva_.fasta

./group171/reference/d1h6ta2-d1p9ag_.fasta

./group171/reference/d1koha1-d1p9ag_.fasta

./group171/reference/d1igra1-d1m6ba2.fasta

./group171/reference/d1nqla1-d1nqla2.fasta

./group171/reference/d1igra1-d1n8yc2.fasta

./group171/reference/d1igra1-d1nqla2.fasta

./group171/reference/d1dcea3-d1p9ag_.fasta

./group171/reference/d1a9na_-d1koha1.fasta

./group171/reference/d1a9na_-d1h6ta2.fasta

./group171/reference/d1h6ta2-d1h6ua2.fasta

./group173/reference/d1dgtb3-d1in1a_.fasta

./group174/reference/d1c2ya_-d1kz1a_.fasta

./group174/reference/d1di0a_-d1rvv1_.fasta

./group174/reference/d1ejba_-d1rvv1_.fasta

./group174/reference/d1hqka_-d1kz1a_.fasta

./group174/reference/d1kz1a_-d1rvv1_.fasta

./group176/reference/d1a2oa1-d1dz3a_.fasta

./group176/reference/d1a2oa1-d1tmy__.fasta

./group176/reference/d1b00a_-d1nat__.fasta

./group176/reference/d1b00a_-d1ntr__.fasta

./group176/reference/d1dbwa_-d1tmy__.fasta

./group176/reference/d1dz3a_-d1mvoa_.fasta

./group176/reference/d1dz3a_-d1nat__.fasta

./group176/reference/d1mvoa_-d1nat__.fasta

./group176/reference/d1mvoa_-d1ntr__.fasta

./group176/reference/d1nat__-d1tmy__.fasta

./group177/reference/d1ag9a_-d1f4pa_.fasta

./group177/reference/d1ag9a_-d2fcr__.fasta

./group177/reference/d1bvyf_-d1ja1a2.fasta

./group177/reference/d1f4pa_-d1fuea_.fasta

./group177/reference/d1f4pa_-d1oboa_.fasta

./group177/reference/d1f4pa_-d2fcr__.fasta

./group177/reference/d1fuea_-d2fcr__.fasta

./group181/reference/d1i7qb_-d1qdlb_.fasta

./group181/reference/d1k9vf_-d1ka9h_.fasta

./group182/reference/d1a8p_2-d1fdr_2.fasta

./group182/reference/d1cqxa3-d1gvha3.fasta

./group182/reference/d1ddga2-d1que_2.fasta

./group182/reference/d1f20a2-d1ja1a3.fasta

./group182/reference/d1ja1a3-d1que_2.fasta

./group182/reference/d1krha2-d1que_2.fasta

./group183/reference/d1g8fa2-d1jhda2.fasta

./group184/reference/d1j20a1-d1k92a1.fasta

./group185/reference/d1iqra2-d1qnf_2.fasta

./group186/reference/d1a9xa3-d1i7na1.fasta

./group186/reference/d1e4ea1-d1iow_1.fasta

./group186/reference/d1ehia1-d1iow_1.fasta

./group190/reference/d1bfd_2-d1jsca2.fasta

./group190/reference/d1bfd_3-d1poxa3.fasta

./group190/reference/d1ovma3-d1pvda3.fasta

./group190/reference/d1ovma3-d1zpda3.fasta

./group190/reference/d1pvda2-d1zpda2.fasta

./group190/reference/d1pvda3-d1zpda3.fasta

./group191/reference/d1g6ha_-d1mt0a_.fasta

./group191/reference/d1mt0a_-d1oxsc2.fasta

./group191/reference/d1mt0a_-d1pf4a1.fasta

./group192/reference/d1ea7a_-d1ic6a_.fasta

./group192/reference/d1ga6a_-d1gt91_.fasta

./group192/reference/d1gci__-d1ic6a_.fasta

./group192/reference/d1ic6a_-d1thm__.fasta

./group192/reference/d1ot5a2-d1thm__.fasta

./group196/reference/d1eeoa_-d1jlna_.fasta

./group196/reference/d1eeoa_-d1lara2.fasta

./group196/reference/d1jlna_-d1lara1.fasta

./group196/reference/d1jlna_-d1lara2.fasta

./group196/reference/d1mkp__-d1vhra_.fasta

./group197/reference/d1e0ca1-d1hzma_.fasta

./group197/reference/d1e0ca1-d1rhs_1.fasta

./group198/reference/d1erv__-d1f9ma_.fasta

./group198/reference/d1fb6a_-d1mek__.fasta

./group198/reference/d1ep7a_-d1f9ma_.fasta

./group198/reference/d1f9ma_-d2trxa_.fasta

./group198/reference/d1erv__-d1mek__.fasta

./group198/reference/d1quwa_-d1thx__.fasta

./group198/reference/d1ep7a_-d1fb6a_.fasta

./group198/reference/d1erv__-d1quwa_.fasta

./group198/reference/d1ep7a_-d1quwa_.fasta

./group198/reference/d1fb6a_-d1quwa_.fasta

./group200/reference/d1a3wa3-d1e0ta3.fasta

./group200/reference/d1a49a3-d1e0ta3.fasta

./group202/reference/d1atia1-d1h4vb1.fasta

./group202/reference/d1atia1-d1hc7a1.fasta

./group202/reference/d1h4vb1-d1kmma1.fasta

./group202/reference/d1hc7a1-d1qf6a1.fasta

./group202/reference/d1nj1a1-d1qf6a1.fasta

./group203/reference/d1cfr__-d1knva_.fasta

./group204/reference/d1g5ca_-d1i6pa_.fasta

./group205/reference/d1bdg_2-d1ig8a2.fasta

./group205/reference/d1bupa1-d1jcfa1.fasta

./group205/reference/d1czan1-d1ig8a1.fasta

./group205/reference/d1czan2-d1ig8a2.fasta

./group207/reference/d1ilya_-d1jj2m_.fasta

./group209/reference/d1gyta2-d1lam_2.fasta

./group209/reference/d1h8la2-d1obr__.fasta

./group209/reference/d1jqga1-d1kwma1.fasta

./group209/reference/d1jqga1-d1m4la_.fasta

./group209/reference/d1jqga1-d1obr__.fasta

./group209/reference/d1kwma1-d1obr__.fasta

./group209/reference/d1m4la_-d1obr__.fasta

./group210/reference/d1a4ia2-d1b0aa2.fasta

./group210/reference/d1bgva2-d1gtma2.fasta

./group210/reference/d1c1da2-d1leha2.fasta

./group210/reference/d1gtma2-d1hwxa2.fasta

./group210/reference/d1npya2-d1nvta2.fasta

./group210/reference/d1nvta2-d1nyta2.fasta

./group211/reference/d1fzta_-d3pgm__.fasta

./group212/reference/d1bd3a_-d1i5ea_.fasta

./group212/reference/d1g2qa_-d1l1qa_.fasta

./group212/reference/d1g2qa_-d1qb7a_.fasta

./group213/reference/d1mf7a_-d1mjna_.fasta

./group213/reference/d1mf7a_-d1qc5a_.fasta

./group214/reference/d1dcta_-d6mhta_.fasta

./group214/reference/d1dl5a1-d1jg1a_.fasta

./group214/reference/d1i1na_-d1jg1a_.fasta

./group215/reference/d1ajsa_-d1qisa_.fasta

./group215/reference/d1ajsa_-d2ay1a_.fasta

./group215/reference/d1e5ea_-d1ibja_.fasta

./group215/reference/d1j32a_-d1o4sa_.fasta

./group215/reference/d1qisa_-d1yaaa_.fasta

./group215/reference/d1qisa_-d7aata_.fasta

./group215/reference/d1yaaa_-d2ay1a_.fasta

./group215/reference/d2ay1a_-d3tata_.fasta

./group215/reference/d2ay1a_-d7aata_.fasta

./group215/reference/d2dkb__-d2oata_.fasta

./group217/reference/d1ac5__-d1cpy__.fasta

./group217/reference/d1ac5__-d1ivya_.fasta

./group217/reference/d1cpy__-d1ivya_.fasta

./group217/reference/d1ea5a_-d1llfa_.fasta

./group217/reference/d1jjia_-d1jkma_.fasta

./group217/reference/d1jjia_-d1lzla_.fasta

./group217/reference/d1jkma_-d1lzla_.fasta

./group218/reference/d1aoea_-d1df7a_.fasta

./group218/reference/d1aoea_-d1ra9__.fasta

./group218/reference/d1aoea_-d3dfr__.fasta

./group218/reference/d1d1ga_-d3dfr__.fasta

./group218/reference/d1df7a_-d1dyr__.fasta

./group218/reference/d1df7a_-d1ra9__.fasta

./group218/reference/d1df7a_-d1vdra_.fasta

./group218/reference/d1df7a_-d3dfr__.fasta

./group218/reference/d1dyr__-d1ra9__.fasta

./group218/reference/d1ra9__-d1vdra_.fasta

./group219/reference/d1bx4a_-d1rkd__.fasta

./group222/reference/d1ed8a_-d1ew2a_.fasta

./group222/reference/d1ed8a_-d1k7ha_.fasta

./group224/reference/d1a1s_2-d1duvg2.fasta

./group224/reference/d1a1s_2-d1js1x2.fasta

./group224/reference/d1duvg1-d1js1x1.fasta

./group224/reference/d1duvg1-d1ml4a1.fasta

./group224/reference/d1duvg2-d1js1x2.fasta

./group224/reference/d1duvg2-d1otha2.fasta

./group224/reference/d1js1x1-d1ml4a1.fasta

./group224/reference/d1ml4a1-d1otha1.fasta

./group226/reference/d1f2da_-d1j0aa_.fasta

./group229/reference/d1a4sa_-d1euha_.fasta

./group229/reference/d1a4sa_-d1ky8a_.fasta

./group229/reference/d1euha_-d1ky8a_.fasta

./group229/reference/d1euha_-d1o04a_.fasta

./group235/reference/d1psza_-d1toaa_.fasta

./group236/reference/d1rpja_-d2dri__.fasta

./group237/reference/d1a99a_-d1pot__.fasta

./group237/reference/d1lst__-d1wdna_.fasta

./group238/reference/d1e5ma1-d1ek4a1.fasta

./group238/reference/d1ek4a1-d1kas_1.fasta

./group238/reference/d1ek4a1-d1ox0a1.fasta

./group238/reference/d1hnja2-d1hzpa2.fasta

./group238/reference/d1hnja2-d1mzja2.fasta

./group238/reference/d1hzpa2-d1ub7a2.fasta

./group239/reference/d1aln_1-d1jtka_.fasta

./group239/reference/d1aln_2-d1jtka_.fasta

./group241/reference/d1b9oa_-d1gd6a_.fasta

./group241/reference/d1b9oa_-d3lzt__.fasta

./group242/reference/d1cvza_-d1gmya_.fasta

./group242/reference/d1cvza_-d1me4a_.fasta

./group242/reference/d1deua_-d1fh0a_.fasta

./group242/reference/d1deua_-d1gmya_.fasta

./group242/reference/d1deua_-d7pcka_.fasta

./group242/reference/d1fh0a_-d1gmya_.fasta

./group242/reference/d1gmya_-d1iwda_.fasta

./group242/reference/d1iwda_-d1me4a_.fasta

./group244/reference/d1agi__-d1gqva_.fasta

./group244/reference/d1agi__-d1rnfa_.fasta

./group244/reference/d1gqva_-d1rnfa_.fasta

./group245/reference/d1b3aa_-d1eiha_.fasta

./group245/reference/d1g2ta_-d2hcc__.fasta

./group245/reference/d1doka_-d1el0a_.fasta

./group245/reference/d1eiha_-d1j9oa_.fasta

./group245/reference/d1el0a_-d1j9oa_.fasta

./group245/reference/d1eiha_-d1f2la_.fasta

./group245/reference/d1j9oa_-d2hcc__.fasta

./group245/reference/d1doka_-d1j9oa_.fasta

./group245/reference/d1doka_-d2hcc__.fasta

./group245/reference/d1b3aa_-d1m8aa_.fasta

./group248/reference/d1jj2r_-d1n88a_.fasta

./group251/reference/d1j8ca_-d1mg8a_.fasta

./group251/reference/d1lfda_-d1rlf__.fasta

./group252/reference/d1c9fa_-d1d4ba_.fasta

./group252/reference/d1c9fa_-d1f2ri_.fasta

./group254/reference/d1jroa2-d1n62a2.fasta

./group254/reference/d1ayfa_-d1put__.fasta

./group254/reference/d1ayfa_-d1b9ra_.fasta

./group254/reference/d1kf6b2-d1qlab2.fasta

./group254/reference/d1czpa_-d1e9ma_.fasta

./group254/reference/d1fo4a2-d1n62a2.fasta

./group254/reference/d1b9ra_-d1put__.fasta

./group254/reference/d1czpa_-d1krha3.fasta

./group254/reference/d1l5pa_-d1put__.fasta

./group254/reference/d1i7ha_-d1krha3.fasta

./group256/reference/d1an8_2-d1enfa2.fasta

./group256/reference/d1an8_2-d1esfa2.fasta

./group256/reference/d1an8_2-d1eu3a2.fasta

./group256/reference/d1enfa2-d1et9a2.fasta

./group256/reference/d1enfa2-d1eu3a2.fasta

./group256/reference/d1enfa2-d3seb_2.fasta

./group256/reference/d1esfa2-d1et9a2.fasta

./group256/reference/d1esfa2-d3seb_2.fasta

./group256/reference/d1et9a2-d1eu3a2.fasta

./group256/reference/d1m4va2-d3tss_2.fasta

./group257/reference/d1an9a2-d1c0pa2.fasta

./group258/reference/d1cewi_-d1g96a_.fasta

./group258/reference/d1g96a_-d1stfi_.fasta

./group259/reference/d1ivwa3-d1oaca3.fasta

./group260/reference/d1ocva_-d1oh0a_.fasta

./group261/reference/d1c16a2-d1zaga2.fasta

./group261/reference/d1cd1a2-d1gzqa2.fasta

./group261/reference/d1de4a2-d1k5na2.fasta

./group261/reference/d1de4a2-d1zaga2.fasta

./group261/reference/d1k5na2-d1zaga2.fasta

./group262/reference/d1i7ka_-d2ucz__.fasta

./group262/reference/d1i7ka_-d2aak__.fasta

./group262/reference/d1c4zd_-d1jata_.fasta

./group262/reference/d1jata_-d2aak__.fasta

./group262/reference/d1c4zd_-d1j7da_.fasta

./group262/reference/d1c4zd_-d1fzya_.fasta

./group262/reference/d1qcqa_-d2ucz__.fasta

./group262/reference/d1qcqa_-d2aak__.fasta

./group262/reference/d1fzya_-d2ucz__.fasta

./group262/reference/d1jata_-d2ucz__.fasta

./group264/reference/d1m5ya3-d1pina2.fasta

./group264/reference/d1ix5a_-d1jvwa_.fasta

./group264/reference/d1bkf__-d1ix5a_.fasta

./group264/reference/d1bkf__-d1fd9a_.fasta

./group264/reference/d1ix5a_-d1j6ya_.fasta

./group264/reference/d1ix5a_-d1pbk__.fasta

./group264/reference/d1eq3a_-d1pina2.fasta

./group264/reference/d1j6ya_-d1jnsa_.fasta

./group264/reference/d1kt1a3-d1pbk__.fasta

./group264/reference/d1j6ya_-d1m5ya3.fasta

./group265/reference/d1edqa3-d1itxa2.fasta

./group265/reference/d1edqa3-d1kfwa2.fasta

./group265/reference/d1edqa3-d1ll7a2.fasta

./group265/reference/d1itxa2-d1ll7a2.fasta

./group266/reference/d1f1ua1-d1lqpa_.fasta

./group266/reference/d1f1ua2-d1lqpa_.fasta

./group266/reference/d1f1ua2-d1qipa_.fasta

./group266/reference/d1kw3b1-d1lqpa_.fasta

./group266/reference/d1kw3b2-d1qipa_.fasta

./group266/reference/d1lqpa_-d1mpya1.fasta

./group266/reference/d1lqpa_-d1mpya2.fasta

./group268/reference/d1csei_-d1dwma_.fasta

./group268/reference/d1csei_-d1lw6i_.fasta

./group268/reference/d1dwma_-d1lw6i_.fasta

./group269/reference/d1fo4a3-d1hlra3.fasta

./group269/reference/d1fo4a3-d1jrob1.fasta

./group269/reference/d1fo4a3-d1n62b1.fasta

./group269/reference/d1hlra3-d1jrob1.fasta

./group269/reference/d1hlra3-d1n62b1.fasta

./group269/reference/d1jrob1-d1n62b1.fasta

./group270/reference/d1qapa2-d1qpoa2.fasta

./group272/reference/d1efub2-d1tfe__.fasta

./group273/reference/d1kkca2-d1ma1a2.fasta

./group273/reference/d1b06a2-d1bsma2.fasta

./group273/reference/d1b06a2-d1ix9a2.fasta

./group273/reference/d1coja2-d1isaa2.fasta

./group273/reference/d1bsma2-d1coja2.fasta

./group273/reference/d1bsma2-d1gv3a2.fasta

./group273/reference/d1coja2-d1ix9a2.fasta

./group273/reference/d1b06a2-d1isaa2.fasta

./group273/reference/d1b06a2-d1coja2.fasta

./group273/reference/d1coja2-d1qnna2.fasta

./group274/reference/d1di2a_-d1o0wa2.fasta

./group274/reference/d1o0wa2-d1qu6a1.fasta

./group275/reference/d1dtja_-d1j4wa2.fasta

./group275/reference/d1dtja_-d1khma_.fasta

./group275/reference/d1j4wa1-d1khma_.fasta

./group275/reference/d1j4wa2-d1khma_.fasta

./group275/reference/d1khma_-d2fmr__.fasta

./group276/reference/d1fjgc1-d1hh2p2.fasta

./group277/reference/d1jpma2-d2chr_2.fasta

./group278/reference/d2fdn__-d7fd1a_.fasta

./group278/reference/d1feha3-d1h0hb_.fasta

./group278/reference/d1fxd__-d1jnrb_.fasta

./group278/reference/d1fxd__-d7fd1a_.fasta

./group278/reference/d1hfel2-d1keka5.fasta

./group278/reference/d1fxd__-d1iqza_.fasta

./group278/reference/d1hfel2-d1jb0c_.fasta

./group278/reference/d1blu__-d1fxra_.fasta

./group278/reference/d1keka5-d2fdn__.fasta

./group278/reference/d1hfel2-d1kqfb1.fasta

./group283/reference/d1fxla1-d1fxla2.fasta

./group283/reference/d1fxla1-d2u2fa_.fasta

./group283/reference/d1nu4a_-d2u1a__.fasta

./group283/reference/d1cvja1-d1fjeb2.fasta

./group283/reference/d1cvja2-d1hd1a_.fasta

./group283/reference/d1b7fa2-d1l3ka1.fasta

./group283/reference/d1b7fa2-d1qm9a1.fasta

./group283/reference/d1l3ka1-d1l3ka2.fasta

./group283/reference/d1cvja1-d1fxla1.fasta

./group283/reference/d1cvja2-d1fxla1.fasta

./group284/reference/d1fnma4-d1n0ua4.fasta

./group285/reference/d1afj__-d1fvqa_.fasta

./group285/reference/d1aw0__-d1jwwa_.fasta

./group285/reference/d1cc8a_-d1fe0a_.fasta

./group285/reference/d1afj__-d1jwwa_.fasta

./group285/reference/d1afj__-d1cpza_.fasta

./group285/reference/d1afj__-d1mwza_.fasta

./group285/reference/d1cpza_-d1k0va_.fasta

./group285/reference/d1cc8a_-d1fvqa_.fasta

./group285/reference/d1afj__-d1cc8a_.fasta

./group285/reference/d1fe0a_-d1jwwa_.fasta

./group290/reference/d1h3fa2-d1jh3a_.fasta

./group295/reference/d1fsz_2-d1ofua2.fasta

./group295/reference/d1tuba2-d1tubb2.fasta

./group296/reference/d1e7ka_-d1jj2f_.fasta

./group297/reference/d1dpta_-d1gd0a_.fasta

./group297/reference/d1dpta_-d1hfoa_.fasta

./group299/reference/d1dxla3-d1lvl_3.fasta

./group299/reference/d1dxla3-d1ojt_3.fasta

./group299/reference/d1feca3-d1h6va3.fasta

./group299/reference/d1lvl_3-d1ojt_3.fasta

./group302/reference/d1icra_-d1vfra_.fasta

./group303/reference/d1bqba_-d1ezm__.fasta

./group303/reference/d1ezm__-d1keia_.fasta

./group303/reference/d1gkda_-d1k7ia2.fasta

./group305/reference/d1fu6a_-d1jyra_.fasta

./group305/reference/d1jyra_-d1m61a_.fasta

./group305/reference/d1a09a_-d1luia_.fasta

./group305/reference/d1a09a_-d1ju5a_.fasta

./group305/reference/d1opka2-d2plda_.fasta

./group305/reference/d1a09a_-d1jyra_.fasta

./group305/reference/d1a09a_-d1fu6a_.fasta

./group305/reference/d1d4ta_-d1mil__.fasta

./group305/reference/d1a81a2-d1lkka_.fasta

./group305/reference/d1a81a1-d1jyra_.fasta

./group306/reference/d1k1ca_-d1pch__.fasta

./group306/reference/d1opd__-d1pch__.fasta

./group306/reference/d1opd__-d1ptf__.fasta

./group306/reference/d1pch__-d1ptf__.fasta

./group307/reference/d1g9za_-d1m5xa_.fasta

./group311/reference/d1ak7__-d1cfya_.fasta

./group311/reference/d1ak7__-d1f7sa_.fasta

./group311/reference/d1d0na3-d1d0na6.fasta

./group314/reference/d1bywa_-d1jnua_.fasta

./group314/reference/d1bywa_-d1n9la_.fasta

./group316/reference/d1k2ea_-d1mut__.fasta

./group317/reference/d1bkpa_-d1f28a_.fasta

./group317/reference/d1bkpa_-d1tis__.fasta

./group318/reference/d1cxya_-d1cyo__.fasta

./group318/reference/d1cxya_-d1kbia2.fasta

./group318/reference/d1cyo__-d1kbia2.fasta

./group320/reference/d1bola_-d1dixa_.fasta

./group320/reference/d1bola_-d1ucaa_.fasta

./group320/reference/d1dixa_-d1iooa_.fasta

./group320/reference/d1dixa_-d1iqqa_.fasta

./group320/reference/d1iqqa_-d1ucaa_.fasta

./group321/reference/d1g61a_-d1g62a_.fasta

./group324/reference/d1aisa1-d1aisa2.fasta

./group324/reference/d1ytba1-d1ytba2.fasta

./group328/reference/d1iz5a1-d1plq_1.fasta

./group329/reference/d1fo4a5-d1hlra4.fasta

./group332/reference/d1a9xa5-d1a9xa6.fasta

./group332/reference/d1e4ea2-d1ehia2.fasta

./group332/reference/d1e4ea2-d1iow_2.fasta

./group332/reference/d1eucb2-d1jkjb2.fasta

./group333/reference/d1apme_-d1phk__.fasta

./group333/reference/d1blxa_-d1m2ra_.fasta

./group333/reference/d1blxa_-d1p38__.fasta

./group333/reference/d1blxa_-d1phk__.fasta

./group333/reference/d1k2pa_-d1p4oa_.fasta

./group333/reference/d1koba_-d1phk__.fasta

./group333/reference/d1m2ra_-d1p38__.fasta

./group333/reference/d1p38__-d1phk__.fasta

./group333/reference/d1phk__-d1tkia_.fasta

./group334/reference/d1fo4a6-d1jroa4.fasta

./group336/reference/d1j2pa_-d1rypg_.fasta

./group336/reference/d1j2pa_-d1rypf_.fasta

./group336/reference/d1rypc_-d1rypd_.fasta

./group336/reference/d1rypb_-d1rypd_.fasta

./group336/reference/d1rypa_-d1rypb_.fasta

./group336/reference/d1pmaa_-d1rypc_.fasta

./group336/reference/d1rypd_-d1rypg_.fasta

./group336/reference/d1ryph_-d1rypi_.fasta

./group336/reference/d1rype_-d1rypg_.fasta

./group336/reference/d1j2pa_-d1rypc_.fasta

./group337/reference/d1a7ta_-d1jjea_.fasta

./group337/reference/d1jjea_-d1m2xa_.fasta

./group337/reference/d1k07a_-d1smla_.fasta

./group339/reference/d1a5z_2-d1ceqa2.fasta

./group339/reference/d1guya2-d1llda2.fasta

./group339/reference/d1guya2-d1o6za2.fasta

./group339/reference/d1a5z_2-d1ez4a2.fasta

./group339/reference/d1i0za2-d1o6za2.fasta

./group339/reference/d1a5z_2-d1guya2.fasta

./group339/reference/d1ldna2-d1o6za2.fasta

./group339/reference/d1i0za2-d1ldna2.fasta

./group339/reference/d1a5z_2-d1i0za2.fasta

./group339/reference/d1b8pa2-d7mdha2.fasta

./group340/reference/d1m2ta_-d1mrj__.fasta

./group340/reference/d1ggpa_-d1m2ta_.fasta

./group340/reference/d1d6aa_-d1mrj__.fasta

./group340/reference/d1ift__-d1mrj__.fasta

./group340/reference/d1hwma_-d1mrj__.fasta

./group340/reference/d1ggpa_-d1mrj__.fasta

./group340/reference/d1ift__-d1m2ta_.fasta

./group340/reference/d1hwma_-d1ift__.fasta

./group340/reference/d1abra_-d1mrj__.fasta

./group340/reference/d1ggpa_-d1ift__.fasta

./group342/reference/d1chua3-d1kssa3.fasta

./group342/reference/d1chua3-d1neka3.fasta

./group342/reference/d1chua3-d1qlaa3.fasta

./group342/reference/d1kf6a3-d1kssa3.fasta

./group342/reference/d1kf6a3-d1qo8a3.fasta

./group342/reference/d1kssa3-d1neka3.fasta

./group342/reference/d1qlaa3-d1qo8a3.fasta

./group343/reference/d1hq8a_-d1k9ja_.fasta

./group343/reference/d1j34a_-d1jzna_.fasta

./group343/reference/d1j34b_-d1qdda_.fasta

./group343/reference/d1j34b_-d1jzna_.fasta

./group343/reference/d1jwib_-d1qdda_.fasta

./group343/reference/d1dv8a_-d1jwib_.fasta

./group343/reference/d1b6e__-d1k9ja_.fasta

./group343/reference/d1dv8a_-d1j34a_.fasta

./group343/reference/d1h8ua_-d1j34b_.fasta

./group343/reference/d1jzna_-d1qdda_.fasta

./group345/reference/d1k9oi_-d1lj5a_.fasta

./group345/reference/d1k9oi_-d1qlpa_.fasta

./group345/reference/d1lj5a_-d1qlpa_.fasta

./group346/reference/d1buea_-d1g6aa_.fasta

./group346/reference/d1g6aa_-d4blma_.fasta

./group346/reference/d1bsg__-d1ghpa_.fasta

./group346/reference/d1ghpa_-d1m40a_.fasta

./group346/reference/d1ghpa_-d1iyoa_.fasta

./group346/reference/d1bsg__-d1g6aa_.fasta

./group346/reference/d1g6aa_-d1iyoa_.fasta

./group346/reference/d1buea_-d1ghpa_.fasta

./group346/reference/d1ghpa_-d1mfoa_.fasta

./group346/reference/d1buea_-d1mfoa_.fasta

./group347/reference/d1a4ea_-d1m7sa_.fasta

./group347/reference/d1cf9a2-d1gwea_.fasta

./group348/reference/d1buca2-d1jqia2.fasta

./group348/reference/d1buca2-d3mdda2.fasta

./group348/reference/d1ivha2-d1jqia2.fasta

./group348/reference/d1ivha2-d3mdda2.fasta

./group348/reference/d1jqia2-d3mdda2.fasta

./group349/reference/d1g0ha_-d1lbva_.fasta

./group350/reference/d1mml__-d1vrta2.fasta

./group355/reference/d1ciy_3-d1ji6a3.fasta

./group361/reference/d1emxa_-d1i25a_.fasta

./group361/reference/d1lupa_-d1qk6a_.fasta

./group361/reference/d1dl0a_-d1koza_.fasta

./group361/reference/d1emxa_-d1g9pa_.fasta

./group361/reference/d1cixa_-d1nixa_.fasta

./group361/reference/d1g9pa_-d1i25a_.fasta

./group361/reference/d1emxa_-d1koza_.fasta

./group361/reference/d1axh__-d1c6wa_.fasta

./group361/reference/d1d1ha_-d1nixa_.fasta

./group361/reference/d1dl0a_-d1emxa_.fasta

./group362/reference/d1aho__-d1tsk__.fasta

./group362/reference/d1myn__-d1sco__.fasta

./group362/reference/d1sco__-d1scy__.fasta

./group362/reference/d1ica__-d1nrb__.fasta

./group362/reference/d1jxca_-d1myn__.fasta

./group362/reference/d1cmr__-d1tsk__.fasta

./group362/reference/d1fjna_-d1sis__.fasta

./group362/reference/d1bcg__-d1tsk__.fasta

./group362/reference/d1ica__-d1tsk__.fasta

./group362/reference/d1ne5a_-d2sn3__.fasta

./group363/reference/d1h59b_-d1m6ba3.fasta

./group363/reference/d1igra3-d1m6ba4.fasta

./group363/reference/d1igra3-d1nqla3.fasta

./group363/reference/d1m6ba3-d1m6ba4.fasta

./group363/reference/d1m6ba3-d1nqla4.fasta

./group364/reference/d1imt_2-d1lpba1.fasta

./group365/reference/d1ioxa_-d1klo_1.fasta

./group365/reference/d1hz8a1-d1ijqa2.fasta

./group365/reference/d1hae__-d1hz8a1.fasta

./group365/reference/d1hz8a2-d1rfnb_.fasta

./group365/reference/d1fjsl_-d1ijqa2.fasta

./group365/reference/d1ijqa2-d1rfnb_.fasta

./group365/reference/d1emo_1-d1rfnb_.fasta

./group365/reference/d1hz8a1-d1rfnb_.fasta

./group365/reference/d1hae__-d1ioxa_.fasta

./group365/reference/d1b9wa2-d1dx5i1.fasta

./group366/reference/d1bx7__-d1skz_1.fasta

./group367/reference/d1f94a_-d1hc9a_.fasta

./group367/reference/d1kbaa_-d1tgxa_.fasta

./group367/reference/d1f94a_-d1jgka_.fasta

./group367/reference/d1fas__-d1tgxa_.fasta

./group367/reference/d1fas__-d1ff4a_.fasta

./group367/reference/d1jgka_-d3ebx__.fasta

./group367/reference/d1kbaa_-d1tfs__.fasta

./group367/reference/d1ff4a_-d1tfs__.fasta

./group367/reference/d1kbaa_-d3ebx__.fasta

./group367/reference/d1fas__-d1tfs__.fasta

./group368/reference/d1bf0__-d1ktha_.fasta

./group368/reference/d1bunb_-d1irha_.fasta

./group368/reference/d1bf0__-d1irha_.fasta

./group368/reference/d1bik_1-d1ktha_.fasta

./group368/reference/d1bik_2-d1jc6a_.fasta

./group368/reference/d1g6xa_-d1ktha_.fasta

./group368/reference/d1bik_2-d1g6xa_.fasta

./group368/reference/d1bik_1-d1irha_.fasta

./group368/reference/d1tocr1-d1tocr2.fasta

./group368/reference/d1bf0__-d1jc6a_.fasta

./group369/reference/d1dfna_-d1ewsa_.fasta

./group369/reference/d1b8wa_-d1bnb__.fasta

./group369/reference/d1d6ba_-d1e4ta_.fasta

./group369/reference/d1bnb__-d1d6ba_.fasta

./group369/reference/d1b8wa_-d2bds__.fasta

./group369/reference/d1e4ta_-d1kj6a_.fasta

./group369/reference/d1d6ba_-d1ijva_.fasta

./group369/reference/d1kj6a_-d1sh1__.fasta

./group369/reference/d1atx__-d2bds__.fasta

./group369/reference/d1d6ba_-d2bds__.fasta

./group371/reference/d1d2ja_-d1f5ya2.fasta

./group371/reference/d1d2ja_-d1n7daa.fasta

./group371/reference/d1d2la_-d1n7daa.fasta

./group371/reference/d1f5ya1-d1n7daa.fasta

./group371/reference/d1f5ya2-d1n7daa.fasta

./group371/reference/d1j8ea_-d1n7daa.fasta

./group371/reference/d1n7da7-d1n7daa.fasta

./group372/reference/d1bhp__-d1ejga_.fasta

./group373/reference/d1kdu__-d2hpqp_.fasta

./group373/reference/d1h8pa1-d1pmla_.fasta

./group373/reference/d1i71a_-d2hpqp_.fasta

./group373/reference/d1kdu__-d1ki0a2.fasta

./group373/reference/d1ki0a2-d1l6ja3.fasta

./group373/reference/d1h8pa1-d1l6ja5.fasta

./group373/reference/d1l6ja3-d5hpga_.fasta

./group373/reference/d1bhta2-d1l6ja3.fasta

./group373/reference/d1i71a_-d1kdu__.fasta

./group373/reference/d1ki0a2-d1l6ja5.fasta

./group374/reference/d1sgpi_-d1tbrr1.fasta

./group374/reference/d1sgpi_-d1tgsi_.fasta

./group374/reference/d1pce__-d1sgpi_.fasta

./group374/reference/d1nuba3-d1sgpi_.fasta

./group374/reference/d1iw4a_-d1tgsi_.fasta

./group374/reference/d1nuba3-d1tbrr2.fasta

./group374/reference/d1lr7a2-d1sgpi_.fasta

./group374/reference/d1iw4a_-d1tbrr2.fasta

./group374/reference/d1ldtl_-d1sgpi_.fasta

./group374/reference/d1ldtl_-d1lr7a2.fasta

./group376/reference/d1hcna_-d1lxia_.fasta

./group376/reference/d1lxia_-d2tgi__.fasta

./group377/reference/d1g40a3-d1quba2.fasta

./group377/reference/d1ly2a2-d1quba2.fasta

./group377/reference/d1ly2a2-d1quba5.fasta

./group377/reference/d1g40a4-d1quba2.fasta

./group377/reference/d1h03p2-d1hcc__.fasta

./group377/reference/d1gpza2-d1quba4.fasta

./group377/reference/d1gkga2-d1hcc__.fasta

./group377/reference/d1h03p2-d1ly2a1.fasta

./group377/reference/d1hcc__-d1hfi__.fasta

./group377/reference/d1g40a2-d1g40a4.fasta

./group379/reference/d1atb__-d1coua_.fasta

./group379/reference/d1ccva_-d1eaic_.fasta

./group379/reference/d1ccva_-d1hx2a_.fasta

./group379/reference/d1coua_-d1eaic_.fasta

./group380/reference/d1jmab1-d1oqdk_.fasta

./group380/reference/d1d4va2-d1exta1.fasta

./group380/reference/d1d4va1-d1exta1.fasta

./group380/reference/d1exta1-d1exta2.fasta

./group380/reference/d1oqdk_-d1oqek_.fasta

./group380/reference/d1d4va2-d1d4va3.fasta

./group380/reference/d1exta3-d1jmab1.fasta

./group380/reference/d1exta1-d1jmab1.fasta

./group380/reference/d1d4va1-d1exta3.fasta

./group380/reference/d1d4va1-d1jmab1.fasta

./group381/reference/d1e88a3-d1o9aa1.fasta

./group381/reference/d1fbr_1-d1fbr_2.fasta

./group381/reference/d1fbr_1-d1o9aa1.fasta

./group381/reference/d1fbr_2-d1o9aa1.fasta

./group381/reference/d1fbr_2-d1o9aa2.fasta

./group381/reference/d1o9aa1-d1o9aa2.fasta

./group381/reference/d1o9aa1-d1tpg_2.fasta

./group382/reference/d1hlqa_-d1iuaa_.fasta

./group382/reference/d1hlqa_-d2hipa_.fasta

./group382/reference/d1iuaa_-d2hipa_.fasta

./group383/reference/d1tf3a1-d1tf6a4.fasta

./group383/reference/d1njqa_-d1ubdc3.fasta

./group383/reference/d1fv5a_-d1ubdc3.fasta

./group383/reference/d1paa__-d1tf6a4.fasta

./group383/reference/d1ncs__-d1zfd__.fasta

./group383/reference/d1bhi__-d2glia2.fasta

./group383/reference/d1njqa_-d1tf6a4.fasta

./group383/reference/d1ncs__-d1tf3a2.fasta

./group383/reference/d1ubdc3-d2glia2.fasta

./group383/reference/d1bhi__-d1zfd__.fasta

./group384/reference/d1d66a1-d1zmec1.fasta

./group384/reference/d1d66a1-d2alca_.fasta

./group384/reference/d1hwtc1-d1pyia1.fasta

./group384/reference/d1hwtc1-d1zmec1.fasta

./group384/reference/d1pyia1-d1zmec1.fasta

./group384/reference/d1zmec1-d2alca_.fasta

./group385/reference/d1d4ua2-d1lata_.fasta

./group385/reference/d1gnf__-d1l1za3.fasta

./group385/reference/d1k3xa3-d1lo1a_.fasta

./group385/reference/d1iml_2-d1lv3a_.fasta

./group385/reference/d1g47a1-d1nypa1.fasta

./group385/reference/d1d4ua2-d1kb2a_.fasta

./group385/reference/d1lv3a_-d1nypa1.fasta

./group385/reference/d1gnf__-d1k3xa3.fasta

./group385/reference/d1d4ua2-d1lo1a_.fasta

./group385/reference/d1fjgn_-d1m3va2.fasta

./group386/reference/d1a6bb_-d1dsva_.fasta

./group387/reference/d1aky_2-d1zaka2.fasta

./group388/reference/d1dl6a_-d1pft__.fasta

./group388/reference/d1i50i1-d1pft__.fasta

./group388/reference/d1i50i2-d1pft__.fasta

./group388/reference/d1qyp__-d1tfi__.fasta

./group391/reference/d1bor__-d1rmd_2.fasta

./group391/reference/d1chc__-d1e4ua_.fasta

./group391/reference/d1fbva4-d1jm7a_.fasta

./group391/reference/d1e4ua_-d1fbva4.fasta

./group391/reference/d1bor__-d1jm7a_.fasta

./group391/reference/d1iyma_-d1ldjb_.fasta

./group391/reference/d1g25a_-d1jm7b_.fasta

./group391/reference/d1e4ua_-d1g25a_.fasta

./group391/reference/d1chc__-d1iyma_.fasta

./group391/reference/d1chc__-d1ldjb_.fasta

./group392/reference/d1fmya_-d1jjda_.fasta

./group393/reference/d1e53a_-d1faq__.fasta

./group393/reference/d1faq__-d1ptq__.fasta

./group394/reference/d1f62a_-d1fp0a1.fasta

./group395/reference/d1e31a_-d1i3oe_.fasta

./group395/reference/d1e31a_-d1jd5a_.fasta

./group396/reference/d1cqxa2-d2pia_1.fasta

./group396/reference/d1ddga1-d1f20a1.fasta

./group396/reference/d1ddga1-d1ja1a1.fasta

./group396/reference/d1f20a1-d1ja1a1.fasta

./group396/reference/d1i7pa1-d1krha1.fasta

./group396/reference/d1i8da2-d1kzla2.fasta

./group396/reference/d1jb9a1-d1krha1.fasta

./group397/reference/d1ezva2-d1l0la2.fasta

./group397/reference/d1hr6a1-d1hr6b1.fasta

./group398/reference/d1gjja2-d1jeia_.fasta

./group399/reference/d1i2ta_-d1ifwa_.fasta

./group399/reference/d1ifwa_-d1jgna_.fasta

./group403/reference/d1nj1a2-d1nj8a2.fasta

./group404/reference/d1h1js_-d1jeqa1.fasta

./group405/reference/d1ayl_2-d1ii2a2.fasta

./group406/reference/d1jida_-d1kvna_.fasta

./group406/reference/d1kvna_-d1lnga_.fasta

./group407/reference/d1cb8a3-d1j0ma3.fasta

./group407/reference/d1j0ma3-d1n7oa3.fasta

./group408/reference/d1a02n1-d1imhc1.fasta

./group408/reference/d1ea9c1-d1ji2a1.fasta

./group408/reference/d1j0ha1-d1ji2a1.fasta

./group408/reference/d1qfha1-d1qfha2.fasta

./group410/reference/d1e12a_-d1h2sa_.fasta

./group411/reference/d1k4cc_-d1p7ba2.fasta

./group415/reference/d1dxrm_-d1qovl_.fasta

./group415/reference/d1qovl_-d1qovm_.fasta

./group416/reference/d1ee8a2-d1l1za2.fasta

./group416/reference/d1ee8a2-d1nnja2.fasta

./group416/reference/d1k82a2-d1l1za2.fasta

./group416/reference/d1k82a2-d1nnja2.fasta

./group419/reference/d1aym3_-d2mev3_.fasta

./group419/reference/d1bev3_-d2mev3_.fasta

./group419/reference/d1bmv11-d1ny711.fasta

./group419/reference/d1f2na_-d1ng0a_.fasta

./group419/reference/d1pvc3_-d2mev3_.fasta

./group419/reference/d1qqp3_-d2mev3_.fasta

./group420/reference/d1gff2_-d1m06g_.fasta

./group420/reference/d1m06g_-d2bpa2_.fasta

./group425/reference/d1h3la_-d1or7a2.fasta
