# Supplementary material for: PR2ALIGN: a stand-alone software program and a web-server for protein sequence alignment using weighted biochemical properties of amino acids
Source: BMC Res Notes. 2015 May 7;8:187. doi: 10.1186/s13104-015-1152-6 (PMC4477417; doi:10.1186/s13104-015-1152-6)
Supplement: Additional file 6: — All SABmark SUP sequence pairs for 0-10% sequence identity range. [file 13104_2015_1152_MOESM6_ESM.docx]

All SABmark SUP sequence pairs for 0-10% sequence identity range.

./group1/reference/d1a6m__-d1alla_.fasta

./group1/reference/d1a6m__-d1allb_.fasta

./group1/reference/d1a6m__-d1b8da_.fasta

./group1/reference/d1a6m__-d1jboa_.fasta

./group1/reference/d1alla_-d1ash__.fasta

./group1/reference/d1alla_-d1cg5a_.fasta

./group1/reference/d1alla_-d1cg5b_.fasta

./group1/reference/d1alla_-d1d8ua_.fasta

./group1/reference/d1alla_-d1gcva_.fasta

./group1/reference/d1alla_-d1gvha1.fasta

./group1/reference/d1alla_-d1hlb__.fasta

./group1/reference/d1alla_-d1irda_.fasta

./group1/reference/d1alla_-d1it2a_.fasta

./group1/reference/d1alla_-d1itha_.fasta

./group1/reference/d1alla_-d1jl7a_.fasta

./group1/reference/d1alla_-d1la6a_.fasta

./group1/reference/d1alla_-d2gdm__.fasta

./group1/reference/d1alla_-d2lhb__.fasta

./group1/reference/d1allb_-d1ash__.fasta

./group1/reference/d1allb_-d1cqxa1.fasta

./group1/reference/d1allb_-d1gvha1.fasta

./group1/reference/d1allb_-d1h97a_.fasta

./group1/reference/d1allb_-d1irdb_.fasta

./group1/reference/d1allb_-d1it2a_.fasta

./group1/reference/d1allb_-d1jl7a_.fasta

./group1/reference/d1allb_-d1la6a_.fasta

./group1/reference/d1allb_-d2lhb__.fasta

./group1/reference/d1allb_-d3sdha_.fasta

./group1/reference/d1ash__-d1b8da_.fasta

./group1/reference/d1ash__-d1jboa_.fasta

./group1/reference/d1b8da_-d1cg5b_.fasta

./group1/reference/d1b8da_-d1cqxa1.fasta

./group1/reference/d1b8da_-d1d8ua_.fasta

./group1/reference/d1b8da_-d1gvha1.fasta

./group1/reference/d1b8da_-d1irda_.fasta

./group1/reference/d1b8da_-d1irdb_.fasta

./group1/reference/d1b8da_-d1it2a_.fasta

./group1/reference/d1b8da_-d1itha_.fasta

./group1/reference/d1b8da_-d1jl7a_.fasta

./group1/reference/d1b8da_-d2gdm__.fasta

./group1/reference/d1b8da_-d2lhb__.fasta

./group1/reference/d1b8da_-d3sdha_.fasta

./group1/reference/d1cqxa1-d1ew6a_.fasta

./group1/reference/d1d8ua_-d1jboa_.fasta

./group1/reference/d1ew6a_-d1h97a_.fasta

./group1/reference/d1ew6a_-d1jboa_.fasta

./group1/reference/d1gvha1-d1jboa_.fasta

./group1/reference/d1h97a_-d1jboa_.fasta

./group1/reference/d1hlb__-d1jboa_.fasta

./group1/reference/d1irda_-d1jboa_.fasta

./group1/reference/d1it2a_-d1jboa_.fasta

./group1/reference/d1itha_-d1jboa_.fasta

./group1/reference/d1jboa_-d1jl7a_.fasta

./group1/reference/d1jboa_-d2gdm__.fasta

./group1/reference/d1jboa_-d3sdha_.fasta

./group2/reference/d1gtea1-d1qlab1.fasta

./group3/reference/d1fpoa1-d1gh6a_.fasta

./group4/reference/d1eiya1-d1seta1.fasta

./group4/reference/d1ivsa1-d1lrza1.fasta

./group6/reference/d1co6a_-d1h32a1.fasta

./group6/reference/d1co6a_-d1iqca2.fasta

./group6/reference/d1cxc__-d1mg2d_.fasta

./group6/reference/d1dw0a_-d1f1ca_.fasta

./group6/reference/d1e29a_-d1h32a1.fasta

./group6/reference/d1eb7a1-d1qn2a_.fasta

./group6/reference/d1etpa2-d1qksa1.fasta

./group6/reference/d1ezvd1-d1h32a1.fasta

./group6/reference/d1ezvd1-d1iqca2.fasta

./group6/reference/d1f1ca_-d1h32a1.fasta

./group6/reference/d1f1ca_-d1mg2d_.fasta

./group6/reference/d1f1ca_-d3c2c__.fasta

./group6/reference/d1fcdc2-d1gu2a_.fasta

./group6/reference/d1h32a1-d1hroa_.fasta

./group6/reference/d1h32a1-d1nira1.fasta

./group6/reference/d1h32a1-d1qksa1.fasta

./group6/reference/d1h32a1-d1ql3a_.fasta

./group6/reference/d1h32a1-d1ycc__.fasta

./group6/reference/d1h32a1-d3c2c__.fasta

./group6/reference/d1hroa_-d1iqca2.fasta

./group6/reference/d1iqca1-d1ycc__.fasta

./group6/reference/d1iqca2-d1qn2a_.fasta

./group6/reference/d1iqca2-d3c2c__.fasta

./group6/reference/d1kb0a1-d3c2c__.fasta

./group7/reference/d1b72b_-d1d5ya2.fasta

./group7/reference/d1b72b_-d1fexa_.fasta

./group7/reference/d1b72b_-d1hlva1.fasta

./group7/reference/d1b72b_-d1irza_.fasta

./group7/reference/d1b72b_-d1k78a2.fasta

./group7/reference/d1b72b_-d2tct_1.fasta

./group7/reference/d1bl0a2-d1e3oc1.fasta

./group7/reference/d1bl0a2-d1hlva2.fasta

./group7/reference/d1bl0a2-d1ig7a_.fasta

./group7/reference/d1bl0a2-d1irza_.fasta

./group7/reference/d1bl0a2-d1iufa2.fasta

./group7/reference/d1bl0a2-d1jt6a1.fasta

./group7/reference/d1bl0a2-d1k61a_.fasta

./group7/reference/d1bl0a2-d1k78a1.fasta

./group7/reference/d1bl0a2-d1qrya_.fasta

./group7/reference/d1bl0a2-d2ezi__.fasta

./group7/reference/d1bw5__-d1g2ha_.fasta

./group7/reference/d1bw5__-d1igna1.fasta

./group7/reference/d1bw5__-d1irza_.fasta

./group7/reference/d1bw5__-d1iufa2.fasta

./group7/reference/d1bw5__-d1jt6a1.fasta

./group7/reference/d1bw5__-d1k78a1.fasta

./group7/reference/d1bw5__-d2tct_1.fasta

./group7/reference/d1d5ya2-d1fexa_.fasta

./group7/reference/d1d5ya2-d1ig7a_.fasta

./group7/reference/d1d5ya2-d1igna1.fasta

./group7/reference/d1d5ya2-d1irza_.fasta

./group7/reference/d1d5ya2-d1iufa1.fasta

./group7/reference/d1d5ya2-d1iufa2.fasta

./group7/reference/d1d5ya2-d1jgga_.fasta

./group7/reference/d1d5ya2-d1k61a_.fasta

./group7/reference/d1d5ya2-d1qrya_.fasta

./group7/reference/d1d5ya2-d2ezi__.fasta

./group7/reference/d1e3oc1-d1g2ha_.fasta

./group7/reference/d1e3oc1-d1hlva2.fasta

./group7/reference/d1e3oc1-d1igna1.fasta

./group7/reference/d1e3oc1-d1irza_.fasta

./group7/reference/d1e3oc1-d1iufa1.fasta

./group7/reference/d1e3oc1-d1jt6a1.fasta

./group7/reference/d1e3oc1-d1k78a1.fasta

./group7/reference/d1e3oc1-d1k78a2.fasta

./group7/reference/d1e3oc1-d2ezi__.fasta

./group7/reference/d1e3oc1-d2ezl__.fasta

./group7/reference/d1fexa_-d1fjla_.fasta

./group7/reference/d1fexa_-d1g2ha_.fasta

./group7/reference/d1fexa_-d1iufa1.fasta

./group7/reference/d1fexa_-d1iufa2.fasta

./group7/reference/d1fjla_-d1g2ha_.fasta

./group7/reference/d1fjla_-d1iufa1.fasta

./group7/reference/d1fjla_-d1jt6a1.fasta

./group7/reference/d1fjla_-d1k78a1.fasta

./group7/reference/d1fjla_-d1k78a2.fasta

./group7/reference/d1fjla_-d1pb6a1.fasta

./group7/reference/d1fjla_-d2ezl__.fasta

./group7/reference/d1g2ha_-d1hlva1.fasta

./group7/reference/d1g2ha_-d1hlva2.fasta

./group7/reference/d1g2ha_-d1ig7a_.fasta

./group7/reference/d1g2ha_-d1igna1.fasta

./group7/reference/d1g2ha_-d1irza_.fasta

./group7/reference/d1g2ha_-d1iufa2.fasta

./group7/reference/d1g2ha_-d1jgga_.fasta

./group7/reference/d1g2ha_-d1jt6a1.fasta

./group7/reference/d1g2ha_-d1k78a1.fasta

./group7/reference/d1g2ha_-d1pb6a1.fasta

./group7/reference/d1g2ha_-d1qrya_.fasta

./group7/reference/d1g2ha_-d2ezi__.fasta

./group7/reference/d1hlva1-d1hlva2.fasta

./group7/reference/d1hlva1-d1ig7a_.fasta

./group7/reference/d1hlva1-d1igna1.fasta

./group7/reference/d1hlva1-d1irza_.fasta

./group7/reference/d1hlva1-d1jgga_.fasta

./group7/reference/d1hlva1-d1k61a_.fasta

./group7/reference/d1hlva1-d1qrya_.fasta

./group7/reference/d1hlva1-d2ezi__.fasta

./group7/reference/d1hlva1-d2ezl__.fasta

./group7/reference/d1hlva2-d1irza_.fasta

./group7/reference/d1hlva2-d1jt6a1.fasta

./group7/reference/d1hlva2-d1pb6a1.fasta

./group7/reference/d1hlva2-d2ezl__.fasta

./group7/reference/d1hlva2-d2tct_1.fasta

./group7/reference/d1ig7a_-d1irza_.fasta

./group7/reference/d1ig7a_-d1iufa1.fasta

./group7/reference/d1ig7a_-d1jt6a1.fasta

./group7/reference/d1ig7a_-d1pb6a1.fasta

./group7/reference/d1ig7a_-d2ezi__.fasta

./group7/reference/d1ig7a_-d2ezl__.fasta

./group7/reference/d1ig7a_-d2tct_1.fasta

./group7/reference/d1igna1-d1iufa1.fasta

./group7/reference/d1igna1-d1iufa2.fasta

./group7/reference/d1igna1-d1jgga_.fasta

./group7/reference/d1igna1-d1jt6a1.fasta

./group7/reference/d1igna1-d1k61a_.fasta

./group7/reference/d1igna1-d1pb6a1.fasta

./group7/reference/d1igna1-d1qrya_.fasta

./group7/reference/d1igna1-d2ezi__.fasta

./group7/reference/d1igna1-d2ezl__.fasta

./group7/reference/d1igna1-d2tct_1.fasta

./group7/reference/d1irza_-d1iufa1.fasta

./group7/reference/d1irza_-d1jgga_.fasta

./group7/reference/d1irza_-d1jt6a1.fasta

./group7/reference/d1irza_-d1k78a1.fasta

./group7/reference/d1irza_-d1k78a2.fasta

./group7/reference/d1irza_-d2ezl__.fasta

./group7/reference/d1irza_-d2tct_1.fasta

./group7/reference/d1iufa1-d1iufa2.fasta

./group7/reference/d1iufa1-d1jgga_.fasta

./group7/reference/d1iufa1-d1jt6a1.fasta

./group7/reference/d1iufa1-d2ezl__.fasta

./group7/reference/d1iufa1-d2tct_1.fasta

./group7/reference/d1iufa2-d1jgga_.fasta

./group7/reference/d1iufa2-d1jt6a1.fasta

./group7/reference/d1iufa2-d1k61a_.fasta

./group7/reference/d1iufa2-d1k78a1.fasta

./group7/reference/d1iufa2-d1k78a2.fasta

./group7/reference/d1iufa2-d1qrya_.fasta

./group7/reference/d1iufa2-d2ezi__.fasta

./group7/reference/d1iufa2-d2tct_1.fasta

./group7/reference/d1jgga_-d1jt6a1.fasta

./group7/reference/d1jgga_-d1k78a1.fasta

./group7/reference/d1jgga_-d1k78a2.fasta

./group7/reference/d1jgga_-d1pb6a1.fasta

./group7/reference/d1jgga_-d2ezi__.fasta

./group7/reference/d1jgga_-d2ezl__.fasta

./group7/reference/d1jgga_-d2tct_1.fasta

./group7/reference/d1jt6a1-d1k78a1.fasta

./group7/reference/d1jt6a1-d1qrya_.fasta

./group7/reference/d1jt6a1-d2ezi__.fasta

./group7/reference/d1k61a_-d1k78a1.fasta

./group7/reference/d1k61a_-d2ezl__.fasta

./group7/reference/d1k61a_-d2tct_1.fasta

./group7/reference/d1k78a1-d1qrya_.fasta

./group7/reference/d1k78a1-d2ezi__.fasta

./group7/reference/d1k78a1-d2ezl__.fasta

./group7/reference/d1k78a1-d2tct_1.fasta

./group7/reference/d1k78a2-d1pb6a1.fasta

./group7/reference/d1pb6a1-d1qrya_.fasta

./group7/reference/d1pb6a1-d2ezi__.fasta

./group7/reference/d1qrya_-d2ezl__.fasta

./group7/reference/d1qrya_-d2tct_1.fasta

./group10/reference/d1bjaa_-d1bm9a_.fasta

./group10/reference/d1bjaa_-d1fsha_.fasta

./group10/reference/d1bjaa_-d1lj9a_.fasta

./group10/reference/d1bjaa_-d1md0a_.fasta

./group10/reference/d1bjaa_-d1omia1.fasta

./group10/reference/d1bjaa_-d1puee_.fasta

./group10/reference/d1bjaa_-d1repc2.fasta

./group10/reference/d1bjaa_-d2hts__.fasta

./group10/reference/d1bm9a_-d1d5va_.fasta

./group10/reference/d1bm9a_-d1f1za1.fasta

./group10/reference/d1bm9a_-d1hkqa_.fasta

./group10/reference/d1bm9a_-d1hsja1.fasta

./group10/reference/d1bm9a_-d1ka8a_.fasta

./group10/reference/d1bm9a_-d1p4xa1.fasta

./group10/reference/d1bm9a_-d2hts__.fasta

./group10/reference/d1d5va_-d1f1za1.fasta

./group10/reference/d1d5va_-d1fsha_.fasta

./group10/reference/d1d5va_-d1hkqa_.fasta

./group10/reference/d1d5va_-d1lj9a_.fasta

./group10/reference/d1d5va_-d1md0a_.fasta

./group10/reference/d1d5va_-d1o7fa1.fasta

./group10/reference/d1d5va_-d1omia1.fasta

./group10/reference/d1d5va_-d1puee_.fasta

./group10/reference/d1d5va_-d1repc2.fasta

./group10/reference/d1d5va_-d1smta_.fasta

./group10/reference/d1d5va_-d2irfg_.fasta

./group10/reference/d1e17a_-d1f1za1.fasta

./group10/reference/d1e17a_-d1fsha_.fasta

./group10/reference/d1e17a_-d1hkqa_.fasta

./group10/reference/d1e17a_-d1hsja1.fasta

./group10/reference/d1e17a_-d1jgsa_.fasta

./group10/reference/d1e17a_-d1lj9a_.fasta

./group10/reference/d1e17a_-d1md0a_.fasta

./group10/reference/d1e17a_-d1omia1.fasta

./group10/reference/d1e17a_-d1puee_.fasta

./group10/reference/d1e17a_-d1smta_.fasta

./group10/reference/d1e17a_-d2hts__.fasta

./group10/reference/d1f1za1-d1hsja1.fasta

./group10/reference/d1f1za1-d1ka8a_.fasta

./group10/reference/d1f1za1-d1ldja1.fasta

./group10/reference/d1f1za1-d1o7fa1.fasta

./group10/reference/d1f1za1-d1omia1.fasta

./group10/reference/d1f1za1-d2irfg_.fasta

./group10/reference/d1fp1d1-d1fsha_.fasta

./group10/reference/d1fp1d1-d1ka8a_.fasta

./group10/reference/d1fp1d1-d1ldja1.fasta

./group10/reference/d1fp2a1-d1hkqa_.fasta

./group10/reference/d1fp2a1-d1hks__.fasta

./group10/reference/d1fp2a1-d1ka8a_.fasta

./group10/reference/d1fp2a1-d1md0a_.fasta

./group10/reference/d1fp2a1-d1p4xa1.fasta

./group10/reference/d1fsha_-d1hkqa_.fasta

./group10/reference/d1fsha_-d1hsja1.fasta

./group10/reference/d1fsha_-d1jgsa_.fasta

./group10/reference/d1fsha_-d1ka8a_.fasta

./group10/reference/d1fsha_-d1ldja1.fasta

./group10/reference/d1fsha_-d1lj9a_.fasta

./group10/reference/d1fsha_-d1md0a_.fasta

./group10/reference/d1fsha_-d1omia1.fasta

./group10/reference/d1fsha_-d1p4xa1.fasta

./group10/reference/d1fsha_-d1puee_.fasta

./group10/reference/d1fsha_-d1repc2.fasta

./group10/reference/d1fsha_-d2hts__.fasta

./group10/reference/d1fsha_-d2irfg_.fasta

./group10/reference/d1hkqa_-d1jgsa_.fasta

./group10/reference/d1hkqa_-d1ka8a_.fasta

./group10/reference/d1hkqa_-d1ldja1.fasta

./group10/reference/d1hkqa_-d1o7fa1.fasta

./group10/reference/d1hkqa_-d1omia1.fasta

./group10/reference/d1hkqa_-d2hts__.fasta

./group10/reference/d1hks__-d1o7fa1.fasta

./group10/reference/d1hks__-d1p4xa2.fasta

./group10/reference/d1hsja1-d1ka8a_.fasta

./group10/reference/d1hsja1-d1omia1.fasta

./group10/reference/d1hsja1-d1repc2.fasta

./group10/reference/d1hsja1-d2hts__.fasta

./group10/reference/d1hsja1-d2irfg_.fasta

./group10/reference/d1jgsa_-d1o7fa1.fasta

./group10/reference/d1jgsa_-d1omia1.fasta

./group10/reference/d1jgsa_-d1puee_.fasta

./group10/reference/d1jgsa_-d1repc2.fasta

./group10/reference/d1jgsa_-d2hts__.fasta

./group10/reference/d1jgsa_-d2irfg_.fasta

./group10/reference/d1ka8a_-d1ldja1.fasta

./group10/reference/d1ka8a_-d1lj9a_.fasta

./group10/reference/d1ka8a_-d1md0a_.fasta

./group10/reference/d1ka8a_-d1p4xa1.fasta

./group10/reference/d1ka8a_-d1puee_.fasta

./group10/reference/d1ka8a_-d1repc2.fasta

./group10/reference/d1ka8a_-d2hts__.fasta

./group10/reference/d1ka8a_-d2irfg_.fasta

./group10/reference/d1ldja1-d1md0a_.fasta

./group10/reference/d1ldja1-d1omia1.fasta

./group10/reference/d1ldja1-d1repc2.fasta

./group10/reference/d1ldja1-d1smta_.fasta

./group10/reference/d1ldja1-d2hts__.fasta

./group10/reference/d1lj9a_-d1md0a_.fasta

./group10/reference/d1lj9a_-d1repc2.fasta

./group10/reference/d1lj9a_-d1smta_.fasta

./group10/reference/d1lj9a_-d2irfg_.fasta

./group10/reference/d1md0a_-d1o7fa1.fasta

./group10/reference/d1md0a_-d1omia1.fasta

./group10/reference/d1md0a_-d1p4xa1.fasta

./group10/reference/d1md0a_-d1p4xa2.fasta

./group10/reference/d1o7fa1-d1omia1.fasta

./group10/reference/d1o7fa1-d2hts__.fasta

./group10/reference/d1omia1-d1p4xa2.fasta

./group10/reference/d1omia1-d1puee_.fasta

./group10/reference/d1omia1-d1repc2.fasta

./group10/reference/d1omia1-d1smta_.fasta

./group10/reference/d1omia1-d2irfg_.fasta

./group10/reference/d1p4xa1-d2irfg_.fasta

./group10/reference/d1p4xa2-d2hts__.fasta

./group10/reference/d1smta_-d2hts__.fasta

./group11/reference/d1a04a1-d1fc3a_.fasta

./group11/reference/d1fc3a_-d1opc__.fasta

./group11/reference/d1fc3a_-d1p4wa_.fasta

./group12/reference/d1f4ia_-d1oaia_.fasta

./group12/reference/d1ifya_-d1oaia_.fasta

./group12/reference/d1ifya_-d1otra_.fasta

./group14/reference/d1d4ua1-d1exja1.fasta

./group14/reference/d1d4ua1-d1g4da_.fasta

./group14/reference/d1d4ua1-d1jbga_.fasta

./group14/reference/d1d4ua1-d1jjcb2.fasta

./group14/reference/d1d4ua1-d1lx8a_.fasta

./group14/reference/d1d4ua1-d1nd9a_.fasta

./group14/reference/d1d4ua1-d1tns__.fasta

./group14/reference/d1exja1-d1j9ia_.fasta

./group14/reference/d1exja1-d1jjcb2.fasta

./group14/reference/d1exja1-d1nd9a_.fasta

./group14/reference/d1j9ia_-d1jjcb2.fasta

./group14/reference/d1jbga_-d1jjcb2.fasta

./group14/reference/d1jbga_-d1lx8a_.fasta

./group14/reference/d1jbga_-d1tns__.fasta

./group14/reference/d1jjcb2-d1lx8a_.fasta

./group14/reference/d1jjcb2-d1nd9a_.fasta

./group14/reference/d1lx8a_-d1tns__.fasta

./group15/reference/d1cuna1-d1quua1.fasta

./group15/reference/d1cuna1-d2spca_.fasta

./group15/reference/d1cuna2-d2spca_.fasta

./group15/reference/d1hcia1-d2spca_.fasta

./group15/reference/d1hcia4-d2spca_.fasta

./group15/reference/d1quua1-d1quua2.fasta

./group15/reference/d1quua2-d2spca_.fasta

./group17/reference/d1deeg_-d1gjta_.fasta

./group18/reference/d1erp__-d1hd6a_.fasta

./group20/reference/d1a32__-d1ail__.fasta

./group20/reference/d1ail__-d1fyja_.fasta

./group22/reference/d1bh9a_-d1jfib_.fasta

./group22/reference/d1bh9a_-d1n1ja_.fasta

./group22/reference/d1bh9a_-d1n1jb_.fasta

./group23/reference/d1gqaa_-d256ba_.fasta

./group23/reference/d1mqva_-d256ba_.fasta

./group25/reference/d1is2a1-d1is2a2.fasta

./group25/reference/d1is2a2-d1ivha1.fasta

./group25/reference/d1is2a2-d1jqia1.fasta

./group25/reference/d1is2a2-d3mdda1.fasta

./group26/reference/d1afra_-d1kgna_.fasta

./group26/reference/d1afra_-d1mtyb_.fasta

./group26/reference/d1afra_-d1mtyd_.fasta

./group26/reference/d1afra_-d1qgha_.fasta

./group26/reference/d1euma_-d1h0oa_.fasta

./group26/reference/d1euma_-d1ji4a_.fasta

./group26/reference/d1euma_-d1jiga_.fasta

./group26/reference/d1euma_-d1jkva_.fasta

./group26/reference/d1euma_-d1kgna_.fasta

./group26/reference/d1euma_-d1mtyb_.fasta

./group26/reference/d1euma_-d1mtyd_.fasta

./group26/reference/d1euma_-d1mxra_.fasta

./group26/reference/d1euma_-d1o9ra_.fasta

./group26/reference/d1euma_-d1qgha_.fasta

./group26/reference/d1h0oa_-d1jgca_.fasta

./group26/reference/d1h0oa_-d1ji4a_.fasta

./group26/reference/d1h0oa_-d1jiga_.fasta

./group26/reference/d1h0oa_-d1lkoa1.fasta

./group26/reference/d1h0oa_-d1mtyb_.fasta

./group26/reference/d1h0oa_-d1mtyd_.fasta

./group26/reference/d1h0oa_-d1nfva_.fasta

./group26/reference/d1h0oa_-d1o9ra_.fasta

./group26/reference/d1h0oa_-d1rcd__.fasta

./group26/reference/d1jgca_-d1jiga_.fasta

./group26/reference/d1jgca_-d1kgna_.fasta

./group26/reference/d1jgca_-d1mtyb_.fasta

./group26/reference/d1jgca_-d1mxra_.fasta

./group26/reference/d1jgca_-d1o9ra_.fasta

./group26/reference/d1ji4a_-d1jkva_.fasta

./group26/reference/d1ji4a_-d1kgna_.fasta

./group26/reference/d1ji4a_-d1lkoa1.fasta

./group26/reference/d1ji4a_-d1mtyd_.fasta

./group26/reference/d1ji4a_-d1mxra_.fasta

./group26/reference/d1jiga_-d1kgna_.fasta

./group26/reference/d1jiga_-d1mtyb_.fasta

./group26/reference/d1jiga_-d1mtyd_.fasta

./group26/reference/d1jiga_-d1mxra_.fasta

./group26/reference/d1jiga_-d1rcd__.fasta

./group26/reference/d1jkva_-d1mtyb_.fasta

./group26/reference/d1jkva_-d1rcd__.fasta

./group26/reference/d1kgna_-d1lkoa1.fasta

./group26/reference/d1kgna_-d1mtyb_.fasta

./group26/reference/d1kgna_-d1mtyd_.fasta

./group26/reference/d1kgna_-d1nfva_.fasta

./group26/reference/d1kgna_-d1qgha_.fasta

./group26/reference/d1kgna_-d1rcd__.fasta

./group26/reference/d1lkoa1-d1mtyb_.fasta

./group26/reference/d1lkoa1-d1mtyd_.fasta

./group26/reference/d1lkoa1-d1mxra_.fasta

./group26/reference/d1mtyb_-d1mxra_.fasta

./group26/reference/d1mtyb_-d1nfva_.fasta

./group26/reference/d1mtyb_-d1o9ra_.fasta

./group26/reference/d1mtyb_-d1qgha_.fasta

./group26/reference/d1mtyb_-d1rcd__.fasta

./group26/reference/d1mtyd_-d1mxra_.fasta

./group26/reference/d1mtyd_-d1o9ra_.fasta

./group26/reference/d1mtyd_-d1qgha_.fasta

./group26/reference/d1mtyd_-d1rcd__.fasta

./group26/reference/d1mxra_-d1nfva_.fasta

./group26/reference/d1mxra_-d1o9ra_.fasta

./group26/reference/d1mxra_-d1qgha_.fasta

./group26/reference/d1mxra_-d1rcd__.fasta

./group26/reference/d1nfva_-d1o9ra_.fasta

./group26/reference/d1nfva_-d1qgha_.fasta

./group26/reference/d1o9ra_-d1rcd__.fasta

./group26/reference/d1qgha_-d1rcd__.fasta

./group27/reference/d1au1a_-d1d9ca_.fasta

./group27/reference/d1au1a_-d1eera_.fasta

./group27/reference/d1au1a_-d1huw__.fasta

./group27/reference/d1au1a_-d1hzia_.fasta

./group27/reference/d1au1a_-d1i1rb_.fasta

./group27/reference/d1au1a_-d1lki__.fasta

./group27/reference/d1au1a_-d1lqsl_.fasta

./group27/reference/d1au1a_-d1n9da_.fasta

./group27/reference/d1d9ca_-d1eera_.fasta

./group27/reference/d1d9ca_-d1hula_.fasta

./group27/reference/d1d9ca_-d1huw__.fasta

./group27/reference/d1d9ca_-d1hzia_.fasta

./group27/reference/d1d9ca_-d1i1rb_.fasta

./group27/reference/d1d9ca_-d1lqsl_.fasta

./group27/reference/d1d9ca_-d1n9da_.fasta

./group27/reference/d1d9ca_-d2gmfa_.fasta

./group27/reference/d1d9ca_-d2ilk__.fasta

./group27/reference/d1eera_-d1etea_.fasta

./group27/reference/d1eera_-d1hula_.fasta

./group27/reference/d1eera_-d1hzia_.fasta

./group27/reference/d1eera_-d1i1rb_.fasta

./group27/reference/d1eera_-d1m4ra_.fasta

./group27/reference/d1eera_-d1n9da_.fasta

./group27/reference/d1etea_-d1hula_.fasta

./group27/reference/d1etea_-d1huw__.fasta

./group27/reference/d1etea_-d1hzia_.fasta

./group27/reference/d1etea_-d1i1rb_.fasta

./group27/reference/d1etea_-d1lki__.fasta

./group27/reference/d1etea_-d1lqsl_.fasta

./group27/reference/d1etea_-d1m4ra_.fasta

./group27/reference/d1hula_-d1huw__.fasta

./group27/reference/d1hula_-d1jli__.fasta

./group27/reference/d1hula_-d1lki__.fasta

./group27/reference/d1hula_-d1lqsl_.fasta

./group27/reference/d1hula_-d1m4ra_.fasta

./group27/reference/d1hula_-d1n9da_.fasta

./group27/reference/d1huw__-d1hzia_.fasta

./group27/reference/d1huw__-d1lki__.fasta

./group27/reference/d1huw__-d2gmfa_.fasta

./group27/reference/d1hzia_-d1jli__.fasta

./group27/reference/d1hzia_-d1m4ra_.fasta

./group27/reference/d1hzia_-d1n9da_.fasta

./group27/reference/d1i1rb_-d1jli__.fasta

./group27/reference/d1i1rb_-d1lqsl_.fasta

./group27/reference/d1i1rb_-d1m4ra_.fasta

./group27/reference/d1i1rb_-d2gmfa_.fasta

./group27/reference/d1i1rb_-d2ilk__.fasta

./group27/reference/d1jli__-d1lqsl_.fasta

./group27/reference/d1lki__-d1lqsl_.fasta

./group27/reference/d1lki__-d1n9da_.fasta

./group27/reference/d1lki__-d2ilk__.fasta

./group27/reference/d1m4ra_-d2gmfa_.fasta

./group27/reference/d1n9da_-d2gmfa_.fasta

./group27/reference/d1n9da_-d2ilk__.fasta

./group28/reference/d1f4la1-d1f7ua1.fasta

./group28/reference/d1f7ua1-d1ffya1.fasta

./group28/reference/d1f7ua1-d1ile_1.fasta

./group28/reference/d1f7ua1-d1ivsa2.fasta

./group28/reference/d1ffya1-d1iq0a1.fasta

./group28/reference/d1ffya1-d1li5a1.fasta

./group28/reference/d1ivsa2-d1li5a1.fasta

./group29/reference/d1af8__-d1n8la_.fasta

./group33/reference/d1d1la_-d1ner__.fasta

./group33/reference/d1ner__-d1vpwa1.fasta

./group33/reference/d1r69__-d1vpwa1.fasta

./group34/reference/d1an4a_-d1nkpa_.fasta

./group34/reference/d1an4a_-d1nkpb_.fasta

./group35/reference/d1alva_-d1eg3a1.fasta

./group35/reference/d1alva_-d1jbaa_.fasta

./group35/reference/d1alva_-d2scpa_.fasta

./group35/reference/d1auib_-d1eg3a1.fasta

./group35/reference/d1c07a_-d1eg3a1.fasta

./group35/reference/d1c07a_-d1m31a_.fasta

./group35/reference/d1c07a_-d1wdcb_.fasta

./group35/reference/d1eg3a1-d1eg3a2.fasta

./group35/reference/d1eg3a1-d1el4a_.fasta

./group35/reference/d1eg3a1-d1f8ha_.fasta

./group35/reference/d1eg3a1-d1ggwa_.fasta

./group35/reference/d1eg3a1-d1jfja_.fasta

./group35/reference/d1eg3a1-d1m31a_.fasta

./group35/reference/d1eg3a1-d1psra_.fasta

./group35/reference/d1eg3a1-d1qjta_.fasta

./group35/reference/d1eg3a1-d1sra__.fasta

./group35/reference/d1eg3a1-d2scpa_.fasta

./group35/reference/d1eg3a2-d1el4a_.fasta

./group35/reference/d1eg3a2-d1k94a_.fasta

./group35/reference/d1eg3a2-d1m31a_.fasta

./group35/reference/d1el4a_-d1m31a_.fasta

./group35/reference/d1f8ha_-d1k94a_.fasta

./group35/reference/d1f8ha_-d2sas__.fasta

./group35/reference/d1ggwa_-d2scpa_.fasta

./group35/reference/d1k94a_-d1psra_.fasta

./group35/reference/d1k94a_-d1wdcb_.fasta

./group35/reference/d1m31a_-d1wdcc_.fasta

./group35/reference/d1m31a_-d2sas__.fasta

./group35/reference/d1m31a_-d2scpa_.fasta

./group35/reference/d1qjta_-d1wdcb_.fasta

./group35/reference/d1qjta_-d2sas__.fasta

./group35/reference/d1sra__-d1wdcb_.fasta

./group35/reference/d1sra__-d2sas__.fasta

./group35/reference/d1wdcb_-d2scpa_.fasta

./group35/reference/d2sas__-d5pal__.fasta

./group37/reference/d1baza_-d2cpga_.fasta

./group37/reference/d1cmba_-d2cpga_.fasta

./group37/reference/d1irqa_-d1mnta_.fasta

./group37/reference/d1irqa_-d2cpga_.fasta

./group37/reference/d1mnta_-d2cpga_.fasta

./group38/reference/d1a0fa1-d1gula1.fasta

./group38/reference/d1eema1-d1oe8a1.fasta

./group38/reference/d1g7oa1-d1gwca1.fasta

./group38/reference/d1g7oa1-d1iyha1.fasta

./group38/reference/d1g7oa1-d2gsta1.fasta

./group38/reference/d1m0ua1-d1pmt_1.fasta

./group41/reference/d1bea__-d1l6ha_.fasta

./group42/reference/d1dp3a_-d1owfa_.fasta

./group42/reference/d1dp3a_-d1owfb_.fasta

./group44/reference/d1b0xa_-d1bqv__.fasta

./group46/reference/d1a77_1-d1tfr_1.fasta

./group47/reference/d1a6s__-d1jvr__.fasta

./group47/reference/d1a6s__-d1mn8a_.fasta

./group47/reference/d1ed1a_-d1jvr__.fasta

./group47/reference/d1hiwa_-d1jvr__.fasta

./group47/reference/d1jvr__-d1mn8a_.fasta

./group48/reference/d1m12a_-d1n69a_.fasta

./group48/reference/d1n69a_-d1nkl__.fasta

./group52/reference/d1aisb1-d1bu2a1.fasta

./group52/reference/d1aisb1-d1f5qb1.fasta

./group52/reference/d1aisb1-d1g3nc1.fasta

./group52/reference/d1aisb1-d1guxb_.fasta

./group52/reference/d1aisb1-d1jkw_1.fasta

./group52/reference/d1aisb1-d1jkw_2.fasta

./group52/reference/d1aisb1-d1vin_2.fasta

./group52/reference/d1aisb2-d1f5qb1.fasta

./group52/reference/d1aisb2-d1guxb_.fasta

./group52/reference/d1aisb2-d1h4ld_.fasta

./group52/reference/d1aisb2-d1jkw_1.fasta

./group52/reference/d1aisb2-d1jkw_2.fasta

./group52/reference/d1aisb2-d1vin_2.fasta

./group52/reference/d1bu2a1-d1h4ld_.fasta

./group52/reference/d1bu2a2-d1h4ld_.fasta

./group52/reference/d1bu2a2-d1vin_1.fasta

./group52/reference/d1bu2a2-d1vola1.fasta

./group52/reference/d1f5qb1-d1f5qb2.fasta

./group52/reference/d1f5qb1-d1h4ld_.fasta

./group52/reference/d1f5qb1-d1vola1.fasta

./group52/reference/d1f5qb2-d1g3nc1.fasta

./group52/reference/d1f5qb2-d1jkw_2.fasta

./group52/reference/d1guxb_-d1h4ld_.fasta

./group52/reference/d1guxb_-d1jkw_2.fasta

./group52/reference/d1guxb_-d1vin_2.fasta

./group52/reference/d1h4ld_-d1vin_2.fasta

./group52/reference/d1h4ld_-d1vola1.fasta

./group52/reference/d1jkw_1-d1vola1.fasta

./group52/reference/d1jkw_2-d1vin_1.fasta

./group52/reference/d1jkw_2-d1vola1.fasta

./group52/reference/d1vin_1-d1vola1.fasta

./group52/reference/d1vin_2-d1vola1.fasta

./group53/reference/d1a1w__-d1ddf__.fasta

./group53/reference/d1a1w__-d3crd__.fasta

./group53/reference/d1d2za_-d1ddf__.fasta

./group53/reference/d1d2za_-d1dgna_.fasta

./group53/reference/d1d2za_-d1n3ka_.fasta

./group53/reference/d1d2zb_-d1n3ka_.fasta

./group53/reference/d1ddf__-d1n3ka_.fasta

./group53/reference/d1dgna_-d1icha_.fasta

./group53/reference/d1dgna_-d1n3ka_.fasta

./group53/reference/d1fada_-d3crd__.fasta

./group53/reference/d1fada_-d3ygsp_.fasta

./group53/reference/d1icha_-d1n3ka_.fasta

./group53/reference/d1icha_-d3crd__.fasta

./group53/reference/d1n3ka_-d3crd__.fasta

./group56/reference/d1dk8a_-d1iapa_.fasta

./group56/reference/d1iapa_-d1omwa1.fasta

./group57/reference/d1aru__-d1cvua1.fasta

./group57/reference/d1bgp__-d1cvua1.fasta

./group57/reference/d1cvua1-d1jdra_.fasta

./group57/reference/d1cvua1-d1llp__.fasta

./group57/reference/d1cvua1-d1mn2__.fasta

./group57/reference/d1cvua1-d1mwva1.fasta

./group57/reference/d1cvua1-d1oafa_.fasta

./group60/reference/d1dlja1-d1f0ya1.fasta

./group60/reference/d1dlja1-d1n1ea1.fasta

./group60/reference/d1dlja1-d1np3a1.fasta

./group60/reference/d1dlja1-d1pgja1.fasta

./group60/reference/d1dlja1-d1qmga1.fasta

./group60/reference/d1dlja1-d2pgd_1.fasta

./group60/reference/d1f0ya1-d1ks9a1.fasta

./group60/reference/d1f0ya1-d1mv8a1.fasta

./group60/reference/d1f0ya1-d1n1ea1.fasta

./group60/reference/d1f0ya1-d1np3a1.fasta

./group60/reference/d1f0ya1-d1pgja1.fasta

./group60/reference/d1f0ya1-d1qmga1.fasta

./group60/reference/d1f0ya1-d2pgd_1.fasta

./group60/reference/d1ks9a1-d1np3a1.fasta

./group60/reference/d1ks9a1-d1pgja1.fasta

./group60/reference/d1ks9a1-d1qmga1.fasta

./group60/reference/d1mv8a1-d1np3a1.fasta

./group60/reference/d1mv8a1-d1qmga1.fasta

./group60/reference/d1n1ea1-d1np3a1.fasta

./group60/reference/d1n1ea1-d1pgja1.fasta

./group60/reference/d1n1ea1-d1qmga1.fasta

./group60/reference/d1np3a1-d1pgja1.fasta

./group60/reference/d1np3a1-d2pgd_1.fasta

./group60/reference/d1pgja1-d1qmga1.fasta

./group60/reference/d1qmga1-d2pgd_1.fasta

./group61/reference/d1ayx__-d1fp3a_.fasta

./group61/reference/d1ayx__-d1g87a1.fasta

./group61/reference/d1ayx__-d1kwfa_.fasta

./group61/reference/d1ayx__-d1nc5a_.fasta

./group61/reference/d1clc_1-d1g9ga_.fasta

./group61/reference/d1clc_1-d1h54a1.fasta

./group61/reference/d1clc_1-d1lf6a1.fasta

./group61/reference/d1fp3a_-d1gai__.fasta

./group61/reference/d1fp3a_-d1h54a1.fasta

./group61/reference/d1fp3a_-d1kwfa_.fasta

./group61/reference/d1g87a1-d1gai__.fasta

./group61/reference/d1g87a1-d1h54a1.fasta

./group61/reference/d1g87a1-d1lf6a1.fasta

./group61/reference/d1g87a1-d1nc5a_.fasta

./group61/reference/d1g9ga_-d1gai__.fasta

./group61/reference/d1g9ga_-d1h54a1.fasta

./group61/reference/d1g9ga_-d1lf6a1.fasta

./group61/reference/d1g9ga_-d1nc5a_.fasta

./group61/reference/d1gai__-d1h54a1.fasta

./group61/reference/d1gai__-d1ks8a_.fasta

./group61/reference/d1gai__-d1kwfa_.fasta

./group61/reference/d1gai__-d1nc5a_.fasta

./group61/reference/d1h54a1-d1lf6a1.fasta

./group61/reference/d1h54a1-d1nc5a_.fasta

./group61/reference/d1ks8a_-d1lf6a1.fasta

./group61/reference/d1ks8a_-d1nc5a_.fasta

./group61/reference/d1kwfa_-d1lf6a1.fasta

./group61/reference/d1lf6a1-d1nc5a_.fasta

./group62/reference/d1hn0a1-d1qaza_.fasta

./group62/reference/d1j0ma1-d1qaza_.fasta

./group62/reference/d1n7oa1-d1qaza_.fasta

./group63/reference/d1c3d__-d5eau_1.fasta

./group63/reference/d1dceb_-d5eau_1.fasta

./group63/reference/d1ld8b_-d5eau_1.fasta

./group66/reference/d1qhba_-d1qi9a_.fasta

./group66/reference/d1qhba_-d1vns__.fasta

./group66/reference/d1qi9a_-d1vns__.fasta

./group68/reference/d1b3ua_-d1ee4a_.fasta

./group68/reference/d1b3ua_-d1h6ka2.fasta

./group68/reference/d1b3ua_-d1hs6a1.fasta

./group68/reference/d1b3ua_-d1lrv__.fasta

./group68/reference/d1b3ua_-d1oxja2.fasta

./group68/reference/d1bpoa1-d1ee4a_.fasta

./group68/reference/d1bpoa1-d1h6ka2.fasta

./group68/reference/d1bpoa1-d1hs6a1.fasta

./group68/reference/d1bpoa1-d1lrv__.fasta

./group68/reference/d1bpoa1-d1n8va_.fasta

./group68/reference/d1ee4a_-d1h6ka1.fasta

./group68/reference/d1ee4a_-d1h6ka2.fasta

./group68/reference/d1ee4a_-d1hs6a1.fasta

./group68/reference/d1ee4a_-d1lrv__.fasta

./group68/reference/d1ee4a_-d1n8va_.fasta

./group68/reference/d1ee4a_-d1oxja2.fasta

./group68/reference/d1h6ka1-d1hs6a1.fasta

./group68/reference/d1h6ka1-d1lrv__.fasta

./group68/reference/d1h6ka1-d1n8va_.fasta

./group68/reference/d1h6ka1-d1oxja2.fasta

./group68/reference/d1h6ka2-d1hs6a1.fasta

./group68/reference/d1h6ka2-d1lrv__.fasta

./group68/reference/d1h6ka2-d1n8va_.fasta

./group68/reference/d1h6ka2-d1oxja2.fasta

./group68/reference/d1hs6a1-d1lrv__.fasta

./group68/reference/d1hs6a1-d1n8va_.fasta

./group68/reference/d1hs6a1-d1oxja2.fasta

./group68/reference/d1lrv__-d1n8va_.fasta

./group68/reference/d1lrv__-d1oxja2.fasta

./group68/reference/d1n8va_-d1oxja2.fasta

./group70/reference/d1elwa_-d1hz4a_.fasta

./group70/reference/d1hz4a_-d1iyga_.fasta

./group71/reference/d1dvpa1-d1eyha_.fasta

./group76/reference/d1lwba_-d1poc__.fasta

./group77/reference/d19hca_-d1dxrc_.fasta

./group77/reference/d19hca_-d1eysc_.fasta

./group77/reference/d19hca_-d1fgja_.fasta

./group77/reference/d19hca_-d1fs7a_.fasta

./group77/reference/d19hca_-d1gu6a_.fasta

./group77/reference/d19hca_-d1h21a_.fasta

./group77/reference/d1aqe__-d1dxrc_.fasta

./group77/reference/d1aqe__-d1eysc_.fasta

./group77/reference/d1aqe__-d1fgja_.fasta

./group77/reference/d1aqe__-d1fs7a_.fasta

./group77/reference/d1aqe__-d1gu6a_.fasta

./group77/reference/d1aqe__-d1h21a_.fasta

./group77/reference/d1dxrc_-d1fgja_.fasta

./group77/reference/d1dxrc_-d1gu6a_.fasta

./group77/reference/d1dxrc_-d1gyoa_.fasta

./group77/reference/d1dxrc_-d1h21a_.fasta

./group77/reference/d1dxrc_-d1hh5a_.fasta

./group77/reference/d1dxrc_-d1m1qa_.fasta

./group77/reference/d1dxrc_-d1wad__.fasta

./group77/reference/d1dxrc_-d2ctha_.fasta

./group77/reference/d1dxrc_-d2cy3__.fasta

./group77/reference/d1dxrc_-d3cyr__.fasta

./group77/reference/d1eysc_-d1fgja_.fasta

./group77/reference/d1eysc_-d1gyoa_.fasta

./group77/reference/d1eysc_-d1h21a_.fasta

./group77/reference/d1eysc_-d1hh5a_.fasta

./group77/reference/d1eysc_-d1qo8a1.fasta

./group77/reference/d1eysc_-d1wad__.fasta

./group77/reference/d1eysc_-d2cy3__.fasta

./group77/reference/d1eysc_-d3cyr__.fasta

./group77/reference/d1fgja_-d1gyoa_.fasta

./group77/reference/d1fgja_-d1h21a_.fasta

./group77/reference/d1fgja_-d1hh5a_.fasta

./group77/reference/d1fgja_-d1kssa1.fasta

./group77/reference/d1fgja_-d1wad__.fasta

./group77/reference/d1fgja_-d2ctha_.fasta

./group77/reference/d1fgja_-d2cy3__.fasta

./group77/reference/d1fgja_-d3caoa_.fasta

./group77/reference/d1fgja_-d3cyr__.fasta

./group77/reference/d1fs7a_-d1gyoa_.fasta

./group77/reference/d1fs7a_-d1h21a_.fasta

./group77/reference/d1fs7a_-d1hh5a_.fasta

./group77/reference/d1fs7a_-d2ctha_.fasta

./group77/reference/d1fs7a_-d2cy3__.fasta

./group77/reference/d1fs7a_-d3caoa_.fasta

./group77/reference/d1fs7a_-d3cyr__.fasta

./group77/reference/d1ft5a_-d1gyoa_.fasta

./group77/reference/d1ft5a_-d1h21a_.fasta

./group77/reference/d1ft5a_-d3cyr__.fasta

./group77/reference/d1gu6a_-d1gyoa_.fasta

./group77/reference/d1gu6a_-d1h21a_.fasta

./group77/reference/d1gu6a_-d1hh5a_.fasta

./group77/reference/d1gu6a_-d1wad__.fasta

./group77/reference/d1gu6a_-d2cy3__.fasta

./group77/reference/d1gu6a_-d3cyr__.fasta

./group77/reference/d1gyoa_-d1h21a_.fasta

./group77/reference/d1h21a_-d1hh5a_.fasta

./group77/reference/d1h21a_-d1kssa1.fasta

./group77/reference/d1h21a_-d1m1qa_.fasta

./group77/reference/d1h21a_-d1qo8a1.fasta

./group77/reference/d1h21a_-d1wad__.fasta

./group77/reference/d1h21a_-d2ctha_.fasta

./group77/reference/d1h21a_-d2cy3__.fasta

./group77/reference/d1h21a_-d3caoa_.fasta

./group77/reference/d1h21a_-d3cyr__.fasta

./group78/reference/d1akjd_-d1jmaa_.fasta

./group78/reference/d1dqta_-d1jmaa_.fasta

./group78/reference/d1fo0a_-d1gsma2.fasta

./group78/reference/d1fo0a_-d1jmaa_.fasta

./group78/reference/d1fo0b_-d1jmaa_.fasta

./group78/reference/d1g9mh1-d1jmaa_.fasta

./group78/reference/d1gsma2-d1gxea_.fasta

./group78/reference/d1gsma2-d1hxma1.fasta

./group78/reference/d1gsma2-d1jmaa_.fasta

./group78/reference/d1gsma2-d1ktke1.fasta

./group78/reference/d1gsma2-d1mfa_2.fasta

./group78/reference/d1gsma2-d1nezg_.fasta

./group78/reference/d1gsma2-d1nfdb1.fasta

./group78/reference/d1gsma2-d1ogad1.fasta

./group78/reference/d1jmaa_-d1nlbh1.fasta

./group78/reference/d1jmaa_-d1ogad1.fasta

./group78/reference/d1jmaa_-d2f5bh1.fasta

./group78/reference/d1jmaa_-d2rhe__.fasta

./group79/reference/d1axib2-d1i1ra1.fasta

./group79/reference/d1axib2-d1iarb1.fasta

./group79/reference/d1bpv__-d1gh7a1.fasta

./group79/reference/d1bpv__-d1i1ra1.fasta

./group79/reference/d1bpv__-d1iarb1.fasta

./group79/reference/d1bqua1-d1lqsr1.fasta

./group79/reference/d1bqua1-d1lqsr2.fasta

./group79/reference/d1bqua1-d1n6va1.fasta

./group79/reference/d1bqua1-d1n6va2.fasta

./group79/reference/d1bqua1-d2fnba_.fasta

./group79/reference/d1cd9b1-d1i1ra1.fasta

./group79/reference/d1cd9b1-d1lqsr2.fasta

./group79/reference/d1cd9b1-d1n6va1.fasta

./group79/reference/d1cfb_1-d1i1ra1.fasta

./group79/reference/d1cfb_1-d1iarb1.fasta

./group79/reference/d1cfb_1-d1lqsr2.fasta

./group79/reference/d1cfb_2-d1egja_.fasta

./group79/reference/d1cfb_2-d1gh7a1.fasta

./group79/reference/d1cfb_2-d1i1ra1.fasta

./group79/reference/d1cfb_2-d1iarb1.fasta

./group79/reference/d1cfb_2-d1lqsr2.fasta

./group79/reference/d1cfb_2-d1n6va2.fasta

./group79/reference/d1eerb1-d1i1ra1.fasta

./group79/reference/d1eerb1-d1lqsr2.fasta

./group79/reference/d1eerb1-d2fnba_.fasta

./group79/reference/d1eerb2-d1i1ra1.fasta

./group79/reference/d1egja_-d1i1ra1.fasta

./group79/reference/d1f6fb1-d1fyhb1.fasta

./group79/reference/d1f6fb1-d1n6va2.fasta

./group79/reference/d1fnf_1-d1iarb1.fasta

./group79/reference/d1fyhb1-d1gh7a1.fasta

./group79/reference/d1fyhb1-d1i1ra1.fasta

./group79/reference/d1fyhb1-d1n6va2.fasta

./group79/reference/d1gh7a1-d1i1ra1.fasta

./group79/reference/d1gh7a1-d1lqsr1.fasta

./group79/reference/d1gh7a1-d1lqsr2.fasta

./group79/reference/d1gh7a1-d1lwra_.fasta

./group79/reference/d1gh7a1-d1n6va2.fasta

./group79/reference/d1gh7a2-d1i1ra1.fasta

./group79/reference/d1gh7a2-d1iarb1.fasta

./group79/reference/d1i1ra1-d1lqsr1.fasta

./group79/reference/d1i1ra1-d1lqsr2.fasta

./group79/reference/d1i1ra1-d1lwra_.fasta

./group79/reference/d1i1ra1-d1n26a3.fasta

./group79/reference/d1i1ra1-d1n6va2.fasta

./group79/reference/d1i1ra1-d1qg3a2.fasta

./group79/reference/d1iarb1-d1lqsr1.fasta

./group79/reference/d1iarb1-d1lqsr2.fasta

./group79/reference/d1iarb1-d1lwra_.fasta

./group79/reference/d1iarb1-d1qg3a2.fasta

./group79/reference/d1iarb1-d2fnba_.fasta

./group79/reference/d1lqsr1-d1n26a2.fasta

./group79/reference/d1lqsr1-d1n26a3.fasta

./group79/reference/d1lqsr1-d1n6va2.fasta

./group79/reference/d1lqsr2-d1n26a2.fasta

./group79/reference/d1lqsr2-d1n6va1.fasta

./group79/reference/d1lqsr2-d2fnba_.fasta

./group79/reference/d1lwra_-d1n6va1.fasta

./group79/reference/d1n26a2-d1qg3a2.fasta

./group79/reference/d1n26a3-d1n6va2.fasta

./group79/reference/d1n26a3-d2fnba_.fasta

./group79/reference/d1n6va1-d1n6va2.fasta

./group79/reference/d1n6va1-d2fnba_.fasta

./group80/reference/d1jz8a1-d1jz8a2.fasta

./group81/reference/d1f13a2-d1kv3a3.fasta

./group81/reference/d1g0da3-d1l9na2.fasta

./group85/reference/d1kyfa1-d1p4ua_.fasta

./group87/reference/d1aoha_-d1e5ba_.fasta

./group87/reference/d1aoha_-d1exh__.fasta

./group87/reference/d1aoha_-d1qba_2.fasta

./group87/reference/d1e5ba_-d1tf4a2.fasta

./group87/reference/d1g1ka_-d1g43a_.fasta

./group87/reference/d1g1ka_-d1nbca_.fasta

./group87/reference/d1g1ka_-d1tf4a2.fasta

./group87/reference/d1qba_2-d1tf4a2.fasta

./group88/reference/d1amx__-d1klfb1.fasta

./group88/reference/d1amx__-d1p5vb_.fasta

./group88/reference/d1klfb1-d1klfb2.fasta

./group88/reference/d1klfb1-d1n67a1.fasta

./group88/reference/d1klfb1-d1n67a2.fasta

./group88/reference/d1klfb1-d1p5vb_.fasta

./group88/reference/d1klfb2-d1n67a2.fasta

./group88/reference/d1n67a1-d1pdkb_.fasta

./group88/reference/d1n67a2-d1p5vb_.fasta

./group88/reference/d1n67a2-d1pdkb_.fasta

./group89/reference/d1a02n2-d1ycsa_.fasta

./group89/reference/d1a3qa2-d1h6fa_.fasta

./group89/reference/d1a3qa2-d1ycsa_.fasta

./group89/reference/d1bg1a2-d1ycsa_.fasta

./group89/reference/d1bvoa_-d1h6fa_.fasta

./group89/reference/d1bvoa_-d1ycsa_.fasta

./group89/reference/d1h6fa_-d1imhc2.fasta

./group89/reference/d1h6fa_-d1ycsa_.fasta

./group89/reference/d1imhc2-d1ycsa_.fasta

./group92/reference/d1aoza2-d1aoza3.fasta

./group92/reference/d1aoza2-d1e30a_.fasta

./group92/reference/d1aoza2-d1ikop_.fasta

./group92/reference/d1aoza2-d1kbva1.fasta

./group92/reference/d1aoza2-d1kcw_2.fasta

./group92/reference/d1aoza2-d1kcw_5.fasta

./group92/reference/d1aoza2-d1m56b1.fasta

./group92/reference/d1aoza2-d1ocrb1.fasta

./group92/reference/d1aoza3-d1kv7a2.fasta

./group92/reference/d1cyx__-d1gska2.fasta

./group92/reference/d1cyx__-d1gw0a2.fasta

./group92/reference/d1cyx__-d1ikop_.fasta

./group92/reference/d1cyx__-d1kcw_5.fasta

./group92/reference/d1cyx__-d1kv7a1.fasta

./group92/reference/d1cyx__-d1kv7a2.fasta

./group92/reference/d1cyx__-d1oe1a2.fasta

./group92/reference/d1e30a_-d1gw0a2.fasta

./group92/reference/d1e30a_-d1kv7a2.fasta

./group92/reference/d1fwxa1-d1kv7a2.fasta

./group92/reference/d1gska2-d1kcw_4.fasta

./group92/reference/d1gska2-d1ocrb1.fasta

./group92/reference/d1gska3-d1ikop_.fasta

./group92/reference/d1gska3-d1kv7a2.fasta

./group92/reference/d1gska3-d1ocrb1.fasta

./group92/reference/d1gw0a1-d1ikop_.fasta

./group92/reference/d1gw0a1-d1ocrb1.fasta

./group92/reference/d1gw0a2-d1kcw_1.fasta

./group92/reference/d1gw0a2-d1kcw_4.fasta

./group92/reference/d1gw0a2-d1kcw_5.fasta

./group92/reference/d1gw0a2-d1kcw_6.fasta

./group92/reference/d1gw0a2-d1m56b1.fasta

./group92/reference/d1gw0a2-d1ocrb1.fasta

./group92/reference/d1gw0a3-d1ikop_.fasta

./group92/reference/d1gw0a3-d1kv7a2.fasta

./group92/reference/d1hfua3-d1ikop_.fasta

./group92/reference/d1ikop_-d1kbva2.fasta

./group92/reference/d1ikop_-d1kcw_2.fasta

./group92/reference/d1ikop_-d1kcw_4.fasta

./group92/reference/d1ikop_-d1kcw_6.fasta

./group92/reference/d1ikop_-d1kv7a2.fasta

./group92/reference/d1ikop_-d1m56b1.fasta

./group92/reference/d1ikop_-d1ocrb1.fasta

./group92/reference/d1kbva1-d1kv7a2.fasta

./group92/reference/d1kcw_1-d1m56b1.fasta

./group92/reference/d1kcw_1-d1ocrb1.fasta

./group92/reference/d1kcw_2-d1kv7a2.fasta

./group92/reference/d1kcw_2-d1ocrb1.fasta

./group92/reference/d1kcw_4-d1kv7a2.fasta

./group92/reference/d1kcw_4-d1ocrb1.fasta

./group92/reference/d1kcw_5-d1m56b1.fasta

./group92/reference/d1kcw_5-d1ocrb1.fasta

./group92/reference/d1kcw_6-d1kv7a2.fasta

./group92/reference/d1kv7a2-d1m56b1.fasta

./group92/reference/d1kv7a2-d1ocrb1.fasta

./group92/reference/d1ocrb1-d1oe1a2.fasta

./group94/reference/d1czya1-d1k2fa_.fasta

./group95/reference/d1bhu__-d1c01a_.fasta

./group95/reference/d1bhu__-d1f53a_.fasta

./group95/reference/d1bhu__-d1g6ea_.fasta

./group95/reference/d1bhu__-d1ha4a_.fasta

./group95/reference/d1bhu__-d2bb2_1.fasta

./group95/reference/d1bhu__-d2bb2_2.fasta

./group95/reference/d1c01a_-d1h4ax2.fasta

./group95/reference/d1c01a_-d1ha4a_.fasta

./group95/reference/d1c01a_-d2bb2_1.fasta

./group95/reference/d1c01a_-d2bb2_2.fasta

./group95/reference/d1f53a_-d1h4ax1.fasta

./group95/reference/d1f53a_-d2bb2_1.fasta

./group95/reference/d1g6ea_-d1ha4a_.fasta

./group95/reference/d1g6ea_-d2bb2_1.fasta

./group96/reference/d1pgs_1-d1pgs_2.fasta

./group96/reference/d1pgs_1-d1phm_2.fasta

./group96/reference/d1pgs_2-d1phm_2.fasta

./group97/reference/d1hx6a1-d1hx6a2.fasta

./group97/reference/d1hx6a1-d1ruxa2.fasta

./group97/reference/d1hx6a2-d1m3ya1.fasta

./group97/reference/d1hx6a2-d1ruxa2.fasta

./group97/reference/d1m3ya1-d1m3ya2.fasta

./group97/reference/d1m3ya1-d1ruxa2.fasta

./group97/reference/d1m3ya2-d1ruxa2.fasta

./group99/reference/d1bhga2-d1d7pm_.fasta

./group99/reference/d1bhga2-d1dlc_1.fasta

./group99/reference/d1bhga2-d1eut_2.fasta

./group99/reference/d1bhga2-d1guia_.fasta

./group99/reference/d1bhga2-d1gwma_.fasta

./group99/reference/d1bhga2-d1h6ya_.fasta

./group99/reference/d1bhga2-d1i5pa1.fasta

./group99/reference/d1bhga2-d1ji6a1.fasta

./group99/reference/d1bhga2-d1ju3a1.fasta

./group99/reference/d1bhga2-d1k12a_.fasta

./group99/reference/d1bhga2-d1k3ia2.fasta

./group99/reference/d1bhga2-d1kgya_.fasta

./group99/reference/d1bhga2-d1of4a_.fasta

./group99/reference/d1ciy_1-d1cx1a_.fasta

./group99/reference/d1ciy_1-d1czsa_.fasta

./group99/reference/d1ciy_1-d1d7pm_.fasta

./group99/reference/d1ciy_1-d1eut_2.fasta

./group99/reference/d1ciy_1-d1gnya_.fasta

./group99/reference/d1ciy_1-d1guia_.fasta

./group99/reference/d1ciy_1-d1h6ya_.fasta

./group99/reference/d1ciy_1-d1jhja_.fasta

./group99/reference/d1ciy_1-d1k12a_.fasta

./group99/reference/d1ciy_1-d1kexa_.fasta

./group99/reference/d1ciy_1-d1kgya_.fasta

./group99/reference/d1ciy_1-d1lnsa2.fasta

./group99/reference/d1ciy_1-d1of4a_.fasta

./group99/reference/d1ciy_1-d1xnaa_.fasta

./group99/reference/d1cx1a_-d1czsa_.fasta

./group99/reference/d1cx1a_-d1d7pm_.fasta

./group99/reference/d1cx1a_-d1dlc_1.fasta

./group99/reference/d1cx1a_-d1eut_2.fasta

./group99/reference/d1cx1a_-d1i5pa1.fasta

./group99/reference/d1cx1a_-d1ji6a1.fasta

./group99/reference/d1cx1a_-d1k12a_.fasta

./group99/reference/d1cx1a_-d1kexa_.fasta

./group99/reference/d1czsa_-d1gnya_.fasta

./group99/reference/d1czsa_-d1gu3a_.fasta

./group99/reference/d1czsa_-d1guia_.fasta

./group99/reference/d1czsa_-d1gwma_.fasta

./group99/reference/d1czsa_-d1h6ya_.fasta

./group99/reference/d1czsa_-d1i5pa1.fasta

./group99/reference/d1czsa_-d1ju3a1.fasta

./group99/reference/d1czsa_-d1jz8a3.fasta

./group99/reference/d1czsa_-d1k42a_.fasta

./group99/reference/d1czsa_-d1xnaa_.fasta

./group99/reference/d1d7pm_-d1gnya_.fasta

./group99/reference/d1d7pm_-d1gu3a_.fasta

./group99/reference/d1d7pm_-d1guia_.fasta

./group99/reference/d1d7pm_-d1gwma_.fasta

./group99/reference/d1d7pm_-d1i5pa1.fasta

./group99/reference/d1d7pm_-d1ju3a1.fasta

./group99/reference/d1d7pm_-d1k42a_.fasta

./group99/reference/d1d7pm_-d1lnsa2.fasta

./group99/reference/d1d7pm_-d1of4a_.fasta

./group99/reference/d1d7pm_-d1xnaa_.fasta

./group99/reference/d1dlc_1-d1eut_2.fasta

./group99/reference/d1dlc_1-d1gnya_.fasta

./group99/reference/d1dlc_1-d1gu3a_.fasta

./group99/reference/d1dlc_1-d1guia_.fasta

./group99/reference/d1dlc_1-d1gwma_.fasta

./group99/reference/d1dlc_1-d1ju3a1.fasta

./group99/reference/d1dlc_1-d1k12a_.fasta

./group99/reference/d1dlc_1-d1k3ia2.fasta

./group99/reference/d1dlc_1-d1of4a_.fasta

./group99/reference/d1dlc_1-d1xnaa_.fasta

./group99/reference/d1eut_2-d1gnya_.fasta

./group99/reference/d1eut_2-d1guia_.fasta

./group99/reference/d1eut_2-d1gwma_.fasta

./group99/reference/d1eut_2-d1h6ya_.fasta

./group99/reference/d1eut_2-d1i5pa1.fasta

./group99/reference/d1eut_2-d1jhja_.fasta

./group99/reference/d1eut_2-d1ji6a1.fasta

./group99/reference/d1eut_2-d1ju3a1.fasta

./group99/reference/d1eut_2-d1kgya_.fasta

./group99/reference/d1eut_2-d1of4a_.fasta

./group99/reference/d1gnya_-d1i5pa1.fasta

./group99/reference/d1gnya_-d1jhja_.fasta

./group99/reference/d1gnya_-d1ji6a1.fasta

./group99/reference/d1gnya_-d1jz8a3.fasta

./group99/reference/d1gnya_-d1k3ia2.fasta

./group99/reference/d1gnya_-d1kexa_.fasta

./group99/reference/d1gnya_-d1lnsa2.fasta

./group99/reference/d1gnya_-d1xnaa_.fasta

./group99/reference/d1gu3a_-d1i5pa1.fasta

./group99/reference/d1gu3a_-d1ji6a1.fasta

./group99/reference/d1gu3a_-d1ju3a1.fasta

./group99/reference/d1gu3a_-d1jz8a3.fasta

./group99/reference/d1gu3a_-d1k12a_.fasta

./group99/reference/d1gu3a_-d1k3ia2.fasta

./group99/reference/d1gu3a_-d1kexa_.fasta

./group99/reference/d1guia_-d1i5pa1.fasta

./group99/reference/d1guia_-d1jhja_.fasta

./group99/reference/d1guia_-d1ju3a1.fasta

./group99/reference/d1guia_-d1jz8a3.fasta

./group99/reference/d1guia_-d1k3ia2.fasta

./group99/reference/d1guia_-d1kexa_.fasta

./group99/reference/d1guia_-d1kgya_.fasta

./group99/reference/d1guia_-d1of4a_.fasta

./group99/reference/d1guia_-d1xnaa_.fasta

./group99/reference/d1gwma_-d1h6ya_.fasta

./group99/reference/d1gwma_-d1jhja_.fasta

./group99/reference/d1gwma_-d1ji6a1.fasta

./group99/reference/d1gwma_-d1ju3a1.fasta

./group99/reference/d1gwma_-d1jz8a3.fasta

./group99/reference/d1gwma_-d1k12a_.fasta

./group99/reference/d1gwma_-d1k3ia2.fasta

./group99/reference/d1gwma_-d1kexa_.fasta

./group99/reference/d1gwma_-d1kgya_.fasta

./group99/reference/d1gwma_-d1lnsa2.fasta

./group99/reference/d1h6ya_-d1jhja_.fasta

./group99/reference/d1h6ya_-d1ji6a1.fasta

./group99/reference/d1h6ya_-d1jz8a3.fasta

./group99/reference/d1h6ya_-d1k12a_.fasta

./group99/reference/d1h6ya_-d1k3ia2.fasta

./group99/reference/d1h6ya_-d1lnsa2.fasta

./group99/reference/d1h6ya_-d1of4a_.fasta

./group99/reference/d1h6ya_-d1xnaa_.fasta

./group99/reference/d1i5pa1-d1ju3a1.fasta

./group99/reference/d1i5pa1-d1k3ia2.fasta

./group99/reference/d1i5pa1-d1k42a_.fasta

./group99/reference/d1i5pa1-d1kexa_.fasta

./group99/reference/d1i5pa1-d1lnsa2.fasta

./group99/reference/d1jhja_-d1ji6a1.fasta

./group99/reference/d1jhja_-d1ju3a1.fasta

./group99/reference/d1jhja_-d1jz8a3.fasta

./group99/reference/d1jhja_-d1kgya_.fasta

./group99/reference/d1jhja_-d1lnsa2.fasta

./group99/reference/d1ji6a1-d1ju3a1.fasta

./group99/reference/d1ji6a1-d1jz8a3.fasta

./group99/reference/d1ji6a1-d1k3ia2.fasta

./group99/reference/d1ji6a1-d1kgya_.fasta

./group99/reference/d1ji6a1-d1lnsa2.fasta

./group99/reference/d1ji6a1-d1xnaa_.fasta

./group99/reference/d1ju3a1-d1jz8a3.fasta

./group99/reference/d1ju3a1-d1k12a_.fasta

./group99/reference/d1ju3a1-d1k3ia2.fasta

./group99/reference/d1ju3a1-d1k42a_.fasta

./group99/reference/d1ju3a1-d1kexa_.fasta

./group99/reference/d1ju3a1-d1kgya_.fasta

./group99/reference/d1ju3a1-d1of4a_.fasta

./group99/reference/d1ju3a1-d1xnaa_.fasta

./group99/reference/d1jz8a3-d1k12a_.fasta

./group99/reference/d1jz8a3-d1k42a_.fasta

./group99/reference/d1jz8a3-d1kexa_.fasta

./group99/reference/d1jz8a3-d1kgya_.fasta

./group99/reference/d1jz8a3-d1lnsa2.fasta

./group99/reference/d1jz8a3-d1xnaa_.fasta

./group99/reference/d1k12a_-d1k42a_.fasta

./group99/reference/d1k12a_-d1of4a_.fasta

./group99/reference/d1k3ia2-d1k42a_.fasta

./group99/reference/d1k3ia2-d1kgya_.fasta

./group99/reference/d1k3ia2-d1lnsa2.fasta

./group99/reference/d1k3ia2-d1of4a_.fasta

./group99/reference/d1k3ia2-d1xnaa_.fasta

./group99/reference/d1k42a_-d1kexa_.fasta

./group99/reference/d1k42a_-d1kgya_.fasta

./group99/reference/d1kexa_-d1kgya_.fasta

./group99/reference/d1kexa_-d1lnsa2.fasta

./group99/reference/d1kexa_-d1of4a_.fasta

./group99/reference/d1kgya_-d1lnsa2.fasta

./group99/reference/d1kgya_-d1xnaa_.fasta

./group99/reference/d1lnsa2-d1of4a_.fasta

./group99/reference/d1of4a_-d1xnaa_.fasta

./group100/reference/d1ahsa_-d1jsma_.fasta

./group100/reference/d1ahsa_-d1qhda2.fasta

./group100/reference/d1ahsa_-d2viua_.fasta

./group100/reference/d1bvp12-d1flca1.fasta

./group100/reference/d1bvp12-d1jsda_.fasta

./group100/reference/d1bvp12-d1qhda2.fasta

./group100/reference/d1bvp12-d2viua_.fasta

./group100/reference/d1flca1-d1qhda2.fasta

./group100/reference/d1flca1-d2viua_.fasta

./group100/reference/d1jsda_-d1qhda2.fasta

./group100/reference/d1qhda2-d2viua_.fasta

./group105/reference/d1a3k__-d1a8d_1.fasta

./group105/reference/d1a3k__-d1bk1__.fasta

./group105/reference/d1a3k__-d1c4ra_.fasta

./group105/reference/d1a3k__-d1dyka2.fasta

./group105/reference/d1a3k__-d1epwa1.fasta

./group105/reference/d1a3k__-d1fx5a_.fasta

./group105/reference/d1a3k__-d1gzca_.fasta

./group105/reference/d1a3k__-d1kit_1.fasta

./group105/reference/d1a3k__-d1kit_2.fasta

./group105/reference/d1a3k__-d1kqra_.fasta

./group105/reference/d1a3k__-d1xnb__.fasta

./group105/reference/d1a3k__-d2sli_1.fasta

./group105/reference/d1a3k__-d3btaa1.fasta

./group105/reference/d1a8d_1-d1bk1__.fasta

./group105/reference/d1a8d_1-d1bkza_.fasta

./group105/reference/d1a8d_1-d1c4ra_.fasta

./group105/reference/d1a8d_1-d1dyka1.fasta

./group105/reference/d1a8d_1-d1dyka2.fasta

./group105/reference/d1a8d_1-d1fx5a_.fasta

./group105/reference/d1a8d_1-d1g86a_.fasta

./group105/reference/d1a8d_1-d1gzca_.fasta

./group105/reference/d1a8d_1-d1kit_1.fasta

./group105/reference/d1a8d_1-d1kit_2.fasta

./group105/reference/d1a8d_1-d1kqra_.fasta

./group105/reference/d1a8d_1-d1xnb__.fasta

./group105/reference/d1a8d_1-d2nlra_.fasta

./group105/reference/d1bk1__-d1bkza_.fasta

./group105/reference/d1bk1__-d1c4ra_.fasta

./group105/reference/d1bk1__-d1d2sa_.fasta

./group105/reference/d1bk1__-d1dyka1.fasta

./group105/reference/d1bk1__-d1dyka2.fasta

./group105/reference/d1bk1__-d1epwa1.fasta

./group105/reference/d1bk1__-d1fx5a_.fasta

./group105/reference/d1bk1__-d1g86a_.fasta

./group105/reference/d1bk1__-d1gzca_.fasta

./group105/reference/d1bk1__-d1kit_1.fasta

./group105/reference/d1bk1__-d1kit_2.fasta

./group105/reference/d1bk1__-d1kqra_.fasta

./group105/reference/d1bk1__-d1n3oa_.fasta

./group105/reference/d1bk1__-d1nls__.fasta

./group105/reference/d1bk1__-d1saca_.fasta

./group105/reference/d1bk1__-d2ayh__.fasta

./group105/reference/d1bk1__-d2pela_.fasta

./group105/reference/d1bk1__-d2sli_1.fasta

./group105/reference/d1bk1__-d3btaa1.fasta

./group105/reference/d1bkza_-d1c4ra_.fasta

./group105/reference/d1bkza_-d1epwa1.fasta

./group105/reference/d1bkza_-d1fx5a_.fasta

./group105/reference/d1bkza_-d1kit_2.fasta

./group105/reference/d1bkza_-d1kqra_.fasta

./group105/reference/d1bkza_-d1n1ta1.fasta

./group105/reference/d1bkza_-d1n3oa_.fasta

./group105/reference/d1bkza_-d1saca_.fasta

./group105/reference/d1bkza_-d1xnb__.fasta

./group105/reference/d1bkza_-d2nlra_.fasta

./group105/reference/d1bkza_-d2sli_1.fasta

./group105/reference/d1bkza_-d3btaa1.fasta

./group105/reference/d1c4ra_-d1epwa1.fasta

./group105/reference/d1c4ra_-d1g86a_.fasta

./group105/reference/d1c4ra_-d1gzca_.fasta

./group105/reference/d1c4ra_-d1kqra_.fasta

./group105/reference/d1c4ra_-d1nls__.fasta

./group105/reference/d1c4ra_-d1xnb__.fasta

./group105/reference/d1c4ra_-d2ayh__.fasta

./group105/reference/d1c4ra_-d2nlra_.fasta

./group105/reference/d1c4ra_-d2pela_.fasta

./group105/reference/d1c4ra_-d3btaa1.fasta

./group105/reference/d1d2sa_-d1fx5a_.fasta

./group105/reference/d1d2sa_-d1gzca_.fasta

./group105/reference/d1d2sa_-d1kqra_.fasta

./group105/reference/d1d2sa_-d1saca_.fasta

./group105/reference/d1d2sa_-d1xnb__.fasta

./group105/reference/d1d2sa_-d2ayh__.fasta

./group105/reference/d1d2sa_-d2nlra_.fasta

./group105/reference/d1dyka1-d1kqra_.fasta

./group105/reference/d1dyka1-d1n3oa_.fasta

./group105/reference/d1dyka1-d1nls__.fasta

./group105/reference/d1dyka1-d1xnb__.fasta

./group105/reference/d1dyka1-d2nlra_.fasta

./group105/reference/d1dyka1-d3btaa1.fasta

./group105/reference/d1dyka2-d1g86a_.fasta

./group105/reference/d1dyka2-d1kit_1.fasta

./group105/reference/d1dyka2-d1kqra_.fasta

./group105/reference/d1dyka2-d1xnb__.fasta

./group105/reference/d1dyka2-d2nlra_.fasta

./group105/reference/d1dyka2-d3btaa1.fasta

./group105/reference/d1epwa1-d1fx5a_.fasta

./group105/reference/d1epwa1-d1gzca_.fasta

./group105/reference/d1epwa1-d1kit_1.fasta

./group105/reference/d1epwa1-d1kit_2.fasta

./group105/reference/d1epwa1-d1kqra_.fasta

./group105/reference/d1epwa1-d1nls__.fasta

./group105/reference/d1epwa1-d1xnb__.fasta

./group105/reference/d1epwa1-d2sli_1.fasta

./group105/reference/d1fx5a_-d1g86a_.fasta

./group105/reference/d1fx5a_-d1kit_1.fasta

./group105/reference/d1fx5a_-d1kqra_.fasta

./group105/reference/d1fx5a_-d1n1ta1.fasta

./group105/reference/d1g86a_-d1gzca_.fasta

./group105/reference/d1g86a_-d1kit_1.fasta

./group105/reference/d1g86a_-d1kqra_.fasta

./group105/reference/d1g86a_-d1n1ta1.fasta

./group105/reference/d1g86a_-d1n3oa_.fasta

./group105/reference/d1g86a_-d1nls__.fasta

./group105/reference/d1g86a_-d1saca_.fasta

./group105/reference/d1g86a_-d2ayh__.fasta

./group105/reference/d1g86a_-d2nlra_.fasta

./group105/reference/d1g86a_-d2pela_.fasta

./group105/reference/d1g86a_-d3btaa1.fasta

./group105/reference/d1gzca_-d1kit_1.fasta

./group105/reference/d1gzca_-d1kit_2.fasta

./group105/reference/d1gzca_-d1saca_.fasta

./group105/reference/d1gzca_-d2nlra_.fasta

./group105/reference/d1gzca_-d3btaa1.fasta

./group105/reference/d1kit_1-d1kqra_.fasta

./group105/reference/d1kit_1-d1n1ta1.fasta

./group105/reference/d1kit_1-d1n3oa_.fasta

./group105/reference/d1kit_1-d1nls__.fasta

./group105/reference/d1kit_1-d1saca_.fasta

./group105/reference/d1kit_1-d1xnb__.fasta

./group105/reference/d1kit_1-d2nlra_.fasta

./group105/reference/d1kit_1-d3btaa1.fasta

./group105/reference/d1kit_2-d1kqra_.fasta

./group105/reference/d1kit_2-d1n3oa_.fasta

./group105/reference/d1kit_2-d1nls__.fasta

./group105/reference/d1kit_2-d1saca_.fasta

./group105/reference/d1kit_2-d1xnb__.fasta

./group105/reference/d1kit_2-d2pela_.fasta

./group105/reference/d1kqra_-d1n1ta1.fasta

./group105/reference/d1kqra_-d1n3oa_.fasta

./group105/reference/d1kqra_-d1saca_.fasta

./group105/reference/d1kqra_-d1xnb__.fasta

./group105/reference/d1kqra_-d2nlra_.fasta

./group105/reference/d1kqra_-d2pela_.fasta

./group105/reference/d1kqra_-d2sli_1.fasta

./group105/reference/d1n1ta1-d1n3oa_.fasta

./group105/reference/d1n1ta1-d1nls__.fasta

./group105/reference/d1n1ta1-d2ayh__.fasta

./group105/reference/d1n1ta1-d2nlra_.fasta

./group105/reference/d1n1ta1-d2pela_.fasta

./group105/reference/d1n1ta1-d3btaa1.fasta

./group105/reference/d1n3oa_-d1saca_.fasta

./group105/reference/d1n3oa_-d1xnb__.fasta

./group105/reference/d1n3oa_-d2sli_1.fasta

./group105/reference/d1nls__-d2ayh__.fasta

./group105/reference/d1nls__-d2nlra_.fasta

./group105/reference/d1nls__-d3btaa1.fasta

./group105/reference/d1saca_-d2ayh__.fasta

./group105/reference/d1saca_-d2nlra_.fasta

./group105/reference/d1xnb__-d2ayh__.fasta

./group105/reference/d1xnb__-d2sli_1.fasta

./group105/reference/d1xnb__-d3btaa1.fasta

./group105/reference/d2ayh__-d3btaa1.fasta

./group105/reference/d2nlra_-d2sli_1.fasta

./group105/reference/d2nlra_-d3btaa1.fasta

./group105/reference/d2pela_-d2sli_1.fasta

./group105/reference/d2sli_1-d3btaa1.fasta

./group107/reference/d1bia_2-d1fx7a3.fasta

./group109/reference/d1jb0e_-d1vie__.fasta

./group110/reference/d1jj2a1-d2eifa1.fasta

./group112/reference/d1i16__-d1k32a1.fasta

./group112/reference/d1k32a1-d1m5za_.fasta

./group112/reference/d1k32a1-d1nf3c_.fasta

./group112/reference/d1k32a1-d1qlca_.fasta

./group113/reference/d1kq1a_-d1mxma1.fasta

./group114/reference/d1an8_1-d1c4qa_.fasta

./group114/reference/d1an8_1-d1prtd_.fasta

./group114/reference/d1an8_1-d1prtf_.fasta

./group114/reference/d1an8_1-d1qb5d_.fasta

./group114/reference/d1an8_1-d3chbd_.fasta

./group114/reference/d1c4qa_-d1eu3a1.fasta

./group114/reference/d1c4qa_-d1prtb1.fasta

./group114/reference/d1c4qa_-d1prtd_.fasta

./group114/reference/d1c4qa_-d3chbd_.fasta

./group114/reference/d1c4qa_-d3seb_1.fasta

./group114/reference/d1enfa1-d1prtb1.fasta

./group114/reference/d1enfa1-d1prtd_.fasta

./group114/reference/d1enfa1-d1prtf_.fasta

./group114/reference/d1enfa1-d1qb5d_.fasta

./group114/reference/d1eu3a1-d1prtf_.fasta

./group114/reference/d1eu3a1-d1qb5d_.fasta

./group114/reference/d1fnua1-d1prtd_.fasta

./group114/reference/d1fnua1-d1prtf_.fasta

./group114/reference/d1fnua1-d1qb5d_.fasta

./group114/reference/d1fnua1-d3chbd_.fasta

./group114/reference/d1prtb1-d3seb_1.fasta

./group114/reference/d1prtb1-d3tss_1.fasta

./group114/reference/d1prtd_-d1qb5d_.fasta

./group114/reference/d1prtd_-d3tss_1.fasta

./group114/reference/d1prtf_-d1qb5d_.fasta

./group114/reference/d1prtf_-d3seb_1.fasta

./group114/reference/d1qb5d_-d3seb_1.fasta

./group114/reference/d1qb5d_-d3tss_1.fasta

./group114/reference/d3chbd_-d3seb_1.fasta

./group114/reference/d3chbd_-d3tss_1.fasta

./group115/reference/d1jb3a_-d1ueab_.fasta

./group116/reference/d1c0aa1-d1d7qa_.fasta

./group116/reference/d1c0aa1-d1fjgq_.fasta

./group116/reference/d1c0aa1-d1gd7a_.fasta

./group116/reference/d1c0aa1-d1gpc__.fasta

./group116/reference/d1c0aa1-d1jb7a2.fasta

./group116/reference/d1c0aa1-d1kxla_.fasta

./group116/reference/d1d7qa_-d1e1oa1.fasta

./group116/reference/d1d7qa_-d1fgua2.fasta

./group116/reference/d1d7qa_-d1fjgl_.fasta

./group116/reference/d1d7qa_-d1fl0a_.fasta

./group116/reference/d1d7qa_-d1gd7a_.fasta

./group116/reference/d1d7qa_-d1gm5a2.fasta

./group116/reference/d1d7qa_-d1iyjb5.fasta

./group116/reference/d1d7qa_-d1jb7a2.fasta

./group116/reference/d1d7qa_-d1jb7a3.fasta

./group116/reference/d1d7qa_-d1jb7b_.fasta

./group116/reference/d1d7qa_-d1kxla_.fasta

./group116/reference/d1d7qa_-d1o7ia_.fasta

./group116/reference/d1d7qa_-d1pxfa_.fasta

./group116/reference/d1d7qa_-d1quqb_.fasta

./group116/reference/d1e1oa1-d1ewia_.fasta

./group116/reference/d1e1oa1-d1fl0a_.fasta

./group116/reference/d1e1oa1-d1jb7a2.fasta

./group116/reference/d1e1oa1-d1jb7a3.fasta

./group116/reference/d1e1oa1-d1jb7b_.fasta

./group116/reference/d1e1oa1-d1jjcb3.fasta

./group116/reference/d1eova1-d1fgua2.fasta

./group116/reference/d1eova1-d1fjgq_.fasta

./group116/reference/d1eova1-d1fl0a_.fasta

./group116/reference/d1eova1-d1gpc__.fasta

./group116/reference/d1eova1-d1jb7a2.fasta

./group116/reference/d1eova1-d1jb7b_.fasta

./group116/reference/d1eova1-d1pxfa_.fasta

./group116/reference/d1ewia_-d1gpc__.fasta

./group116/reference/d1ewia_-d1ltla_.fasta

./group116/reference/d1ewia_-d1qvca_.fasta

./group116/reference/d1fgua1-d1fjgl_.fasta

./group116/reference/d1fgua1-d1fl0a_.fasta

./group116/reference/d1fgua1-d1gpc__.fasta

./group116/reference/d1fgua1-d1jjcb3.fasta

./group116/reference/d1fgua1-d1pxfa_.fasta

./group116/reference/d1fgua2-d1gd7a_.fasta

./group116/reference/d1fgua2-d1jb7a3.fasta

./group116/reference/d1fgua2-d1ltla_.fasta

./group116/reference/d1fgua2-d1pxfa_.fasta

./group116/reference/d1fjgl_-d1gd7a_.fasta

./group116/reference/d1fjgl_-d1gpc__.fasta

./group116/reference/d1fjgl_-d1iyjb5.fasta

./group116/reference/d1fjgl_-d1jb7a1.fasta

./group116/reference/d1fjgl_-d1jjcb3.fasta

./group116/reference/d1fjgl_-d1kxla_.fasta

./group116/reference/d1fjgq_-d1gpc__.fasta

./group116/reference/d1fjgq_-d1jb7a2.fasta

./group116/reference/d1fjgq_-d1jb7a3.fasta

./group116/reference/d1fl0a_-d1gm5a2.fasta

./group116/reference/d1fl0a_-d1iyjb5.fasta

./group116/reference/d1fl0a_-d1jb7a1.fasta

./group116/reference/d1fl0a_-d1jb7a2.fasta

./group116/reference/d1fl0a_-d1jb7b_.fasta

./group116/reference/d1fl0a_-d1kxla_.fasta

./group116/reference/d1fl0a_-d1o7ia_.fasta

./group116/reference/d1fl0a_-d1quqb_.fasta

./group116/reference/d1fl0a_-d1qvca_.fasta

./group116/reference/d1gd7a_-d1iyjb5.fasta

./group116/reference/d1gd7a_-d1jb7a1.fasta

./group116/reference/d1gd7a_-d1jb7a2.fasta

./group116/reference/d1gd7a_-d1kxla_.fasta

./group116/reference/d1gd7a_-d1qvca_.fasta

./group116/reference/d1gm5a2-d1gpc__.fasta

./group116/reference/d1gm5a2-d1qvca_.fasta

./group116/reference/d1gpc__-d1iyjb5.fasta

./group116/reference/d1gpc__-d1jb7a1.fasta

./group116/reference/d1gpc__-d1jb7a3.fasta

./group116/reference/d1gpc__-d1kxla_.fasta

./group116/reference/d1gpc__-d1ltla_.fasta

./group116/reference/d1gpc__-d1pxfa_.fasta

./group116/reference/d1gpc__-d1qvca_.fasta

./group116/reference/d1iyjb5-d1jb7a3.fasta

./group116/reference/d1iyjb5-d1kxla_.fasta

./group116/reference/d1jb7a1-d1jb7a3.fasta

./group116/reference/d1jb7a1-d1kxla_.fasta

./group116/reference/d1jb7a1-d1pxfa_.fasta

./group116/reference/d1jb7a1-d1qvca_.fasta

./group116/reference/d1jb7a3-d1kxla_.fasta

./group116/reference/d1jb7a3-d1o7ia_.fasta

./group116/reference/d1jb7b_-d1jjcb3.fasta

./group116/reference/d1jb7b_-d1ltla_.fasta

./group116/reference/d1jb7b_-d1quqb_.fasta

./group116/reference/d1jb7b_-d1qvca_.fasta

./group116/reference/d1jjcb3-d1kxla_.fasta

./group116/reference/d1kxla_-d1ltla_.fasta

./group116/reference/d1kxla_-d1pxfa_.fasta

./group116/reference/d1ltla_-d1pxfa_.fasta

./group116/reference/d1o7ia_-d1pxfa_.fasta

./group118/reference/d1h9ra1-d1oxsc1.fasta

./group119/reference/d1ihka_-d1ilr1_.fasta

./group119/reference/d1ilr1_-d1nuna_.fasta

./group123/reference/d1dar_1-d1jj2b_.fasta

./group123/reference/d1exma1-d1jj2b_.fasta

./group123/reference/d1f60a1-d1jj2b_.fasta

./group123/reference/d1jj2b_-d1n0ua1.fasta

./group125/reference/d1ci0a_-d1ejea_.fasta

./group125/reference/d1ci0a_-d1flma_.fasta

./group125/reference/d1ci0a_-d1i0ra_.fasta

./group125/reference/d1ejea_-d1nrga_.fasta

./group125/reference/d1flma_-d1nrga_.fasta

./group125/reference/d1i0ra_-d1nrga_.fasta

./group126/reference/d1arb__-d1lvoa_.fasta

./group126/reference/d1azza_-d1lvoa_.fasta

./group126/reference/d1eaxa_-d1lvoa_.fasta

./group126/reference/d1eq9a_-d1lvoa_.fasta

./group126/reference/d1lvoa_-d1rfna_.fasta

./group126/reference/d1lvoa_-d2hlca_.fasta

./group128/reference/d1dpja_-d1nsoa_.fasta

./group128/reference/d1fkna_-d1nsoa_.fasta

./group128/reference/d1kzka_-d1lf2a_.fasta

./group130/reference/d1cr5a1-d1g8ka1.fasta

./group130/reference/d1cr5a1-d1kqfa1.fasta

./group130/reference/d1e32a1-d1h0ha1.fasta

./group131/reference/d1btn__-d1ddma_.fasta

./group131/reference/d1btn__-d1fhoa_.fasta

./group131/reference/d1btn__-d1gg3a2.fasta

./group131/reference/d1btn__-d1h4ra2.fasta

./group131/reference/d1btn__-d1k5db_.fasta

./group131/reference/d1btn__-d1mkea1.fasta

./group131/reference/d1btn__-d1ntva_.fasta

./group131/reference/d1btn__-d1shca_.fasta

./group131/reference/d1ddma_-d1dro__.fasta

./group131/reference/d1ddma_-d1dyna_.fasta

./group131/reference/d1ddma_-d1eaza_.fasta

./group131/reference/d1ddma_-d1evha_.fasta

./group131/reference/d1ddma_-d1faoa_.fasta

./group131/reference/d1ddma_-d1fhoa_.fasta

./group131/reference/d1ddma_-d1mai__.fasta

./group131/reference/d1ddma_-d1qqga1.fasta

./group131/reference/d1ddma_-d1qqga2.fasta

./group131/reference/d1dro__-d1evha_.fasta

./group131/reference/d1dro__-d1gg3a2.fasta

./group131/reference/d1dro__-d1h4ra2.fasta

./group131/reference/d1dro__-d1k5db_.fasta

./group131/reference/d1dro__-d1mai__.fasta

./group131/reference/d1dro__-d1mixa2.fasta

./group131/reference/d1dro__-d1mkea1.fasta

./group131/reference/d1dro__-d1ntva_.fasta

./group131/reference/d1dro__-d1shca_.fasta

./group131/reference/d1dyna_-d1evha_.fasta

./group131/reference/d1dyna_-d1gg3a2.fasta

./group131/reference/d1dyna_-d1h4ra2.fasta

./group131/reference/d1dyna_-d1k5db_.fasta

./group131/reference/d1dyna_-d1mkea1.fasta

./group131/reference/d1dyna_-d1ntva_.fasta

./group131/reference/d1dyna_-d1shca_.fasta

./group131/reference/d1eaza_-d1evha_.fasta

./group131/reference/d1eaza_-d1k5db_.fasta

./group131/reference/d1eaza_-d1ntva_.fasta

./group131/reference/d1eaza_-d1shca_.fasta

./group131/reference/d1evha_-d1faoa_.fasta

./group131/reference/d1evha_-d1fhoa_.fasta

./group131/reference/d1evha_-d1gg3a2.fasta

./group131/reference/d1evha_-d1h4ra2.fasta

./group131/reference/d1evha_-d1mai__.fasta

./group131/reference/d1evha_-d1mixa2.fasta

./group131/reference/d1evha_-d1ntva_.fasta

./group131/reference/d1evha_-d1pls__.fasta

./group131/reference/d1evha_-d1qqga1.fasta

./group131/reference/d1evha_-d1qqga2.fasta

./group131/reference/d1evha_-d1shca_.fasta

./group131/reference/d1faoa_-d1gg3a2.fasta

./group131/reference/d1faoa_-d1h4ra2.fasta

./group131/reference/d1faoa_-d1k5db_.fasta

./group131/reference/d1fhoa_-d1gg3a2.fasta

./group131/reference/d1fhoa_-d1h4ra2.fasta

./group131/reference/d1fhoa_-d1k5db_.fasta

./group131/reference/d1fhoa_-d1mai__.fasta

./group131/reference/d1fhoa_-d1mixa2.fasta

./group131/reference/d1fhoa_-d1mkea1.fasta

./group131/reference/d1fhoa_-d1ntva_.fasta

./group131/reference/d1fhoa_-d1shca_.fasta

./group131/reference/d1gg3a2-d1k5db_.fasta

./group131/reference/d1gg3a2-d1mai__.fasta

./group131/reference/d1gg3a2-d1mkea1.fasta

./group131/reference/d1gg3a2-d1pls__.fasta

./group131/reference/d1gg3a2-d1qqga1.fasta

./group131/reference/d1gg3a2-d1qqga2.fasta

./group131/reference/d1h4ra2-d1k5db_.fasta

./group131/reference/d1h4ra2-d1mkea1.fasta

./group131/reference/d1h4ra2-d1qqga2.fasta

./group131/reference/d1k5db_-d1mixa2.fasta

./group131/reference/d1k5db_-d1pls__.fasta

./group131/reference/d1k5db_-d1qqga2.fasta

./group131/reference/d1k5db_-d1shca_.fasta

./group131/reference/d1mai__-d1mkea1.fasta

./group131/reference/d1mixa2-d1qqga1.fasta

./group131/reference/d1mkea1-d1pls__.fasta

./group131/reference/d1mkea1-d1qqga2.fasta

./group131/reference/d1mkea1-d1shca_.fasta

./group131/reference/d1ntva_-d1qqga2.fasta

./group131/reference/d1pls__-d1shca_.fasta

./group131/reference/d1qqga1-d1qqga2.fasta

./group131/reference/d1qqga1-d1shca_.fasta

./group133/reference/d1avgi_-d1bj7__.fasta

./group133/reference/d1avgi_-d1cbs__.fasta

./group133/reference/d1avgi_-d1kqwa_.fasta

./group133/reference/d1avgi_-d1mdc__.fasta

./group133/reference/d1avgi_-d1o1va_.fasta

./group133/reference/d1avgi_-d1qfta_.fasta

./group133/reference/d1b56__-d1beba_.fasta

./group133/reference/d1b56__-d1euoa_.fasta

./group133/reference/d1b56__-d1ew3a_.fasta

./group133/reference/d1b56__-d1gkab_.fasta

./group133/reference/d1b56__-d1koia_.fasta

./group133/reference/d1b56__-d1kt7a_.fasta

./group133/reference/d1b56__-d1qfta_.fasta

./group133/reference/d1b56__-d1qqsa_.fasta

./group133/reference/d1beba_-d1ftpa_.fasta

./group133/reference/d1beba_-d1ggla_.fasta

./group133/reference/d1beba_-d1hms__.fasta

./group133/reference/d1beba_-d1ifc__.fasta

./group133/reference/d1beba_-d1kqwa_.fasta

./group133/reference/d1beba_-d1mdc__.fasta

./group133/reference/d1beba_-d1o1va_.fasta

./group133/reference/d1beba_-d1p6pa_.fasta

./group133/reference/d1bj7__-d1ftpa_.fasta

./group133/reference/d1bj7__-d1i4ua_.fasta

./group133/reference/d1bj7__-d1koia_.fasta

./group133/reference/d1cbs__-d1gkab_.fasta

./group133/reference/d1cbs__-d1qfta_.fasta

./group133/reference/d1cbs__-d1qqsa_.fasta

./group133/reference/d1dzka_-d1ggla_.fasta

./group133/reference/d1dzka_-d1i4ua_.fasta

./group133/reference/d1dzka_-d1kqwa_.fasta

./group133/reference/d1dzka_-d1mdc__.fasta

./group133/reference/d1dzka_-d1o1va_.fasta

./group133/reference/d1dzka_-d1p6pa_.fasta

./group133/reference/d1euoa_-d1ftpa_.fasta

./group133/reference/d1euoa_-d1ggla_.fasta

./group133/reference/d1euoa_-d1hms__.fasta

./group133/reference/d1euoa_-d1jv4a_.fasta

./group133/reference/d1euoa_-d1kqwa_.fasta

./group133/reference/d1euoa_-d1mdc__.fasta

./group133/reference/d1euoa_-d1o1va_.fasta

./group133/reference/d1euoa_-d1qfta_.fasta

./group133/reference/d1ew3a_-d1ftpa_.fasta

./group133/reference/d1ew3a_-d1hms__.fasta

./group133/reference/d1ew3a_-d1i4ua_.fasta

./group133/reference/d1ew3a_-d1kqwa_.fasta

./group133/reference/d1ew3a_-d1mdc__.fasta

./group133/reference/d1ew3a_-d1p6pa_.fasta

./group133/reference/d1ftpa_-d1gkab_.fasta

./group133/reference/d1ftpa_-d1jv4a_.fasta

./group133/reference/d1ftpa_-d1koia_.fasta

./group133/reference/d1ftpa_-d1qfta_.fasta

./group133/reference/d1g85a_-d1mdc__.fasta

./group133/reference/d1g85a_-d1p6pa_.fasta

./group133/reference/d1ggla_-d1gkab_.fasta

./group133/reference/d1ggla_-d1jv4a_.fasta

./group133/reference/d1ggla_-d1jzua_.fasta

./group133/reference/d1ggla_-d1koia_.fasta

./group133/reference/d1ggla_-d1qfta_.fasta

./group133/reference/d1ggla_-d1qqsa_.fasta

./group133/reference/d1gkab_-d1hms__.fasta

./group133/reference/d1gkab_-d1kqwa_.fasta

./group133/reference/d1gkab_-d1mdc__.fasta

./group133/reference/d1hms__-d1i4ua_.fasta

./group133/reference/d1hms__-d1koia_.fasta

./group133/reference/d1hms__-d1kt7a_.fasta

./group133/reference/d1hms__-d1qfta_.fasta

./group133/reference/d1i4ua_-d1mdc__.fasta

./group133/reference/d1ifc__-d1jzua_.fasta

./group133/reference/d1ifc__-d1koia_.fasta

./group133/reference/d1ifc__-d1qfta_.fasta

./group133/reference/d1ifc__-d1qqsa_.fasta

./group133/reference/d1jv4a_-d1kqwa_.fasta

./group133/reference/d1jv4a_-d1mdc__.fasta

./group133/reference/d1jzua_-d1kqwa_.fasta

./group133/reference/d1jzua_-d1mdc__.fasta

./group133/reference/d1koia_-d1kqwa_.fasta

./group133/reference/d1koia_-d1mdc__.fasta

./group133/reference/d1koia_-d1o1va_.fasta

./group133/reference/d1koia_-d1p6pa_.fasta

./group133/reference/d1kqwa_-d1qfta_.fasta

./group133/reference/d1kqwa_-d1qqsa_.fasta

./group133/reference/d1kt7a_-d1mdc__.fasta

./group133/reference/d1kt7a_-d1qfta_.fasta

./group133/reference/d1o1va_-d1qfta_.fasta

./group133/reference/d1o1va_-d1qqsa_.fasta

./group133/reference/d1p6pa_-d1qfta_.fasta

./group133/reference/d1qfta_-d1qqsa_.fasta

./group136/reference/d1e8ua_-d1f8ea_.fasta

./group136/reference/d1f8ea_-d2sli_2.fasta

./group136/reference/d1f8ea_-d3sil__.fasta

./group136/reference/d2bat__-d3sil__.fasta

./group138/reference/d1bag_1-d1ea9c2.fasta

./group138/reference/d1bag_1-d1eh9a2.fasta

./group138/reference/d1bag_1-d1j0ha2.fasta

./group138/reference/d1bag_1-d1uasa1.fasta

./group138/reference/d1bf2_2-d1e43a1.fasta

./group138/reference/d1bf2_2-d1eh9a2.fasta

./group138/reference/d1bf2_2-d1ht6a1.fasta

./group138/reference/d1bf2_2-d1ktba1.fasta

./group138/reference/d1bf2_2-d1kwga1.fasta

./group138/reference/d1bf2_2-d1mxga1.fasta

./group138/reference/d1e43a1-d1ea9c2.fasta

./group138/reference/d1e43a1-d1eh9a2.fasta

./group138/reference/d1e43a1-d1g94a1.fasta

./group138/reference/d1e43a1-d1ht6a1.fasta

./group138/reference/d1e43a1-d1j0ha2.fasta

./group138/reference/d1e43a1-d1kwga1.fasta

./group138/reference/d1e43a1-d1uok_1.fasta

./group138/reference/d1ea9c2-d1eh9a2.fasta

./group138/reference/d1ea9c2-d1ht6a1.fasta

./group138/reference/d1ea9c2-d1iv8a1.fasta

./group138/reference/d1eh9a2-d1g5aa1.fasta

./group138/reference/d1eh9a2-d1g94a1.fasta

./group138/reference/d1eh9a2-d1gcya1.fasta

./group138/reference/d1eh9a2-d1ht6a1.fasta

./group138/reference/d1eh9a2-d1hx0a1.fasta

./group138/reference/d1eh9a2-d1iv8a1.fasta

./group138/reference/d1eh9a2-d1jae_1.fasta

./group138/reference/d1eh9a2-d1ji1a2.fasta

./group138/reference/d1eh9a2-d1ji2a2.fasta

./group138/reference/d1eh9a2-d1ktba1.fasta

./group138/reference/d1eh9a2-d1m53a1.fasta

./group138/reference/d1eh9a2-d1qhoa3.fasta

./group138/reference/d1eh9a2-d7taa_1.fasta

./group138/reference/d1g5aa1-d1gcya1.fasta

./group138/reference/d1g5aa1-d1ht6a1.fasta

./group138/reference/d1g94a1-d1ktba1.fasta

./group138/reference/d1g94a1-d1uasa1.fasta

./group138/reference/d1g94a1-d1uok_1.fasta

./group138/reference/d1gcya1-d1gjwa1.fasta

./group138/reference/d1gcya1-d1hx0a1.fasta

./group138/reference/d1gcya1-d1iv8a1.fasta

./group138/reference/d1gcya1-d1jae_1.fasta

./group138/reference/d1gcya1-d1kwga1.fasta

./group138/reference/d1gcya1-d1uok_1.fasta

./group138/reference/d1gjwa1-d1ji2a2.fasta

./group138/reference/d1gjwa1-d1ktba1.fasta

./group138/reference/d1gjwa1-d1mxga1.fasta

./group138/reference/d1gjwa1-d1uasa1.fasta

./group138/reference/d1gjwa1-d7taa_1.fasta

./group138/reference/d1ht6a1-d1hx0a1.fasta

./group138/reference/d1ht6a1-d1jae_1.fasta

./group138/reference/d1ht6a1-d1m7xa2.fasta

./group138/reference/d1ht6a1-d1uok_1.fasta

./group138/reference/d1ht6a1-d7taa_1.fasta

./group138/reference/d1hx0a1-d1mxga1.fasta

./group138/reference/d1hx0a1-d1uok_1.fasta

./group138/reference/d1iv8a1-d1mxga1.fasta

./group138/reference/d1j0ha2-d1jae_1.fasta

./group138/reference/d1j0ha2-d7taa_1.fasta

./group138/reference/d1jae_1-d1mxga1.fasta

./group138/reference/d1ji2a2-d1mxga1.fasta

./group138/reference/d1ktba1-d1kwga1.fasta

./group138/reference/d1ktba1-d1m7xa2.fasta

./group138/reference/d1ktba1-d1mxga1.fasta

./group138/reference/d1kwga1-d1mxga1.fasta

./group138/reference/d1kwga1-d1uasa1.fasta

./group138/reference/d1m7xa2-d1uasa1.fasta

./group138/reference/d1m7xa2-d7taa_1.fasta

./group138/reference/d1mxga1-d1uasa1.fasta

./group138/reference/d1mxga1-d1uok_1.fasta

./group138/reference/d1qhoa3-d7taa_1.fasta

./group138/reference/d1uasa1-d7taa_1.fasta

./group138/reference/d1uok_1-d7taa_1.fasta

./group141/reference/d1i5pa2-d1ji6a2.fasta

./group143/reference/d1bhe__-d1dbga_.fasta

./group143/reference/d1bhe__-d1ee6a_.fasta

./group143/reference/d1bn8a_-d1daba_.fasta

./group143/reference/d1bn8a_-d1hg8a_.fasta

./group143/reference/d1bn8a_-d1qjva_.fasta

./group143/reference/d1czfa_-d1dbga_.fasta

./group143/reference/d1daba_-d1ee6a_.fasta

./group143/reference/d1daba_-d1qcxa_.fasta

./group143/reference/d1daba_-d1qjva_.fasta

./group143/reference/d1daba_-d1rmg__.fasta

./group143/reference/d1dbga_-d1ee6a_.fasta

./group143/reference/d1dbga_-d1qjva_.fasta

./group143/reference/d1dbga_-d1rmg__.fasta

./group143/reference/d1ee6a_-d1rmg__.fasta

./group143/reference/d1jtaa_-d1rmg__.fasta

./group143/reference/d1qcxa_-d1rmg__.fasta

./group144/reference/d1hm9a1-d1kk6a_.fasta

./group144/reference/d1hm9a1-d1krra_.fasta

./group144/reference/d1hm9a1-d1lxa__.fasta

./group144/reference/d1hm9a1-d1ocxa_.fasta

./group144/reference/d1hm9a1-d1qrea_.fasta

./group144/reference/d1hm9a1-d1xat__.fasta

./group144/reference/d1hm9a1-d3tdt__.fasta

./group144/reference/d1lxa__-d1qrea_.fasta

./group144/reference/d1lxa__-d1xat__.fasta

./group144/reference/d1lxa__-d3tdt__.fasta

./group145/reference/d1dzra_-d1lrha_.fasta

./group145/reference/d1dzra_-d1m4oa_.fasta

./group145/reference/d1dzra_-d1pmi__.fasta

./group145/reference/d1ep0a_-d1lkna_.fasta

./group145/reference/d1ep0a_-d1m4oa_.fasta

./group145/reference/d1ep0a_-d1pmi__.fasta

./group145/reference/d1fxza2-d1lrha_.fasta

./group145/reference/d1lkna_-d1lrha_.fasta

./group145/reference/d1lkna_-d2phla1.fasta

./group145/reference/d1lrha_-d1nxma_.fasta

./group145/reference/d1m4oa_-d1od5a2.fasta

./group145/reference/d1o4ta_-d2phla1.fasta

./group145/reference/d1pmi__-d2phla1.fasta

./group146/reference/d1gy9a_-d1odma_.fasta

./group149/reference/d1b6ra1-d1hcz_2.fasta

./group149/reference/d1b6ra1-d1kjqa1.fasta

./group149/reference/d1hcz_2-d1kjqa1.fasta

./group154/reference/d1a53__-d1pii_2.fasta

./group154/reference/d1dbta_-d1pii_1.fasta

./group154/reference/d1dbta_-d1pii_2.fasta

./group154/reference/d1dbta_-d1thfd_.fasta

./group154/reference/d1dqwa_-d1nsj__.fasta

./group154/reference/d1dqwa_-d1pii_2.fasta

./group154/reference/d1eixa_-d1pii_1.fasta

./group154/reference/d1i4na_-d1pii_2.fasta

./group154/reference/d1km3a_-d1nsj__.fasta

./group154/reference/d1km3a_-d1pii_2.fasta

./group154/reference/d1kv8a_-d1nsj__.fasta

./group154/reference/d1kv8a_-d1pii_2.fasta

./group155/reference/d1o94a1-d2dora_.fasta

./group157/reference/d1bf2_3-d1cbg__.fasta

./group157/reference/d1bf2_3-d1e4ia_.fasta

./group157/reference/d1bf2_3-d1e4mm_.fasta

./group157/reference/d1bf2_3-d1hxja_.fasta

./group157/reference/d1bf2_3-d1qba_3.fasta

./group157/reference/d1bf2_3-d1ug6a_.fasta

./group157/reference/d1cbg__-d1eh9a3.fasta

./group157/reference/d1cbg__-d1eswa_.fasta

./group157/reference/d1cbg__-d1g5aa2.fasta

./group157/reference/d1cbg__-d1gjwa2.fasta

./group157/reference/d1cbg__-d1iexa1.fasta

./group157/reference/d1cbg__-d1j0ha3.fasta

./group157/reference/d1cbg__-d1j18a2.fasta

./group157/reference/d1cbg__-d1ji1a3.fasta

./group157/reference/d1cbg__-d1lwha2.fasta

./group157/reference/d1cbg__-d1m53a2.fasta

./group157/reference/d1cbg__-d1uok_2.fasta

./group157/reference/d1cbg__-d7taa_2.fasta

./group157/reference/d1cz1a_-d1g5aa2.fasta

./group157/reference/d1cz1a_-d1gjwa2.fasta

./group157/reference/d1cz1a_-d1iexa1.fasta

./group157/reference/d1cz1a_-d1j18a2.fasta

./group157/reference/d1cz1a_-d1m53a2.fasta

./group157/reference/d1cz1a_-d1uok_2.fasta

./group157/reference/d1cz1a_-d7taa_2.fasta

./group157/reference/d1e43a2-d1e4ia_.fasta

./group157/reference/d1e43a2-d1e4mm_.fasta

./group157/reference/d1e43a2-d1hxja_.fasta

./group157/reference/d1e43a2-d1qba_3.fasta

./group157/reference/d1e43a2-d1qvba_.fasta

./group157/reference/d1e43a2-d1ug6a_.fasta

./group157/reference/d1e4ia_-d1eh9a3.fasta

./group157/reference/d1e4ia_-d1gjwa2.fasta

./group157/reference/d1e4ia_-d1iexa1.fasta

./group157/reference/d1e4ia_-d1j18a2.fasta

./group157/reference/d1e4ia_-d1ji1a3.fasta

./group157/reference/d1e4ia_-d1lwha2.fasta

./group157/reference/d1e4ia_-d1m53a2.fasta

./group157/reference/d1e4ia_-d1pama4.fasta

./group157/reference/d1e4ia_-d1qba_3.fasta

./group157/reference/d1e4ia_-d1qhoa4.fasta

./group157/reference/d1e4ia_-d7taa_2.fasta

./group157/reference/d1e4mm_-d1eswa_.fasta

./group157/reference/d1e4mm_-d1g5aa2.fasta

./group157/reference/d1e4mm_-d1gjwa2.fasta

./group157/reference/d1e4mm_-d1iexa1.fasta

./group157/reference/d1e4mm_-d1j0ha3.fasta

./group157/reference/d1e4mm_-d1j18a2.fasta

./group157/reference/d1e4mm_-d1ji1a3.fasta

./group157/reference/d1e4mm_-d1lwha2.fasta

./group157/reference/d1e4mm_-d1m53a2.fasta

./group157/reference/d1e4mm_-d1qhoa4.fasta

./group157/reference/d1e4mm_-d1uok_2.fasta

./group157/reference/d1e4mm_-d7taa_2.fasta

./group157/reference/d1eh9a3-d1hxja_.fasta

./group157/reference/d1eh9a3-d1ug6a_.fasta

./group157/reference/d1eswa_-d1iexa1.fasta

./group157/reference/d1eswa_-d1qba_3.fasta

./group157/reference/d1eswa_-d1qvba_.fasta

./group157/reference/d1g5aa2-d1hxja_.fasta

./group157/reference/d1g5aa2-d1iexa1.fasta

./group157/reference/d1g5aa2-d1j18a2.fasta

./group157/reference/d1g5aa2-d1qba_3.fasta

./group157/reference/d1g5aa2-d1qvba_.fasta

./group157/reference/d1g5aa2-d1ug6a_.fasta

./group157/reference/d1gjwa2-d1hxja_.fasta

./group157/reference/d1gjwa2-d1iexa1.fasta

./group157/reference/d1gjwa2-d1j18a2.fasta

./group157/reference/d1gjwa2-d1kwga2.fasta

./group157/reference/d1gjwa2-d1qba_3.fasta

./group157/reference/d1gjwa2-d1qvba_.fasta

./group157/reference/d1gjwa2-d1ug6a_.fasta

./group157/reference/d1hxja_-d1iexa1.fasta

./group157/reference/d1hxja_-d1j0ha3.fasta

./group157/reference/d1hxja_-d1ji1a3.fasta

./group157/reference/d1hxja_-d7taa_2.fasta

./group157/reference/d1iexa1-d1j18a2.fasta

./group157/reference/d1iexa1-d1ji1a3.fasta

./group157/reference/d1iexa1-d1kwga2.fasta

./group157/reference/d1iexa1-d1lwha2.fasta

./group157/reference/d1iexa1-d1pama4.fasta

./group157/reference/d1iexa1-d1qvba_.fasta

./group157/reference/d1iexa1-d1ug6a_.fasta

./group157/reference/d1iexa1-d1uok_2.fasta

./group157/reference/d1iexa1-d7taa_2.fasta

./group157/reference/d1j18a2-d1ji1a3.fasta

./group157/reference/d1j18a2-d1lwha2.fasta

./group157/reference/d1j18a2-d1m53a2.fasta

./group157/reference/d1j18a2-d1pama4.fasta

./group157/reference/d1j18a2-d1qhoa4.fasta

./group157/reference/d1j18a2-d1uok_2.fasta

./group157/reference/d1ji1a3-d1qba_3.fasta

./group157/reference/d1ji1a3-d1ug6a_.fasta

./group157/reference/d1kwga2-d1m53a2.fasta

./group157/reference/d1kwga2-d1qhoa4.fasta

./group157/reference/d1kwga2-d7taa_2.fasta

./group157/reference/d1lwha2-d1ug6a_.fasta

./group157/reference/d1m53a2-d1qba_3.fasta

./group157/reference/d1m53a2-d1ug6a_.fasta

./group157/reference/d1pama4-d1qba_3.fasta

./group157/reference/d1pama4-d1ug6a_.fasta

./group157/reference/d1qba_3-d1qhoa4.fasta

./group157/reference/d1qba_3-d1ug6a_.fasta

./group157/reference/d1qba_3-d1uok_2.fasta

./group157/reference/d1qba_3-d7taa_2.fasta

./group157/reference/d1qhoa4-d1qvba_.fasta

./group157/reference/d1qvba_-d1uok_2.fasta

./group157/reference/d1ug6a_-d1uok_2.fasta

./group157/reference/d1ug6a_-d7taa_2.fasta

./group158/reference/d1a4ma_-d1itua_.fasta

./group158/reference/d1i0da_-d1j5sa_.fasta

./group158/reference/d1j5sa_-d1k6wa2.fasta

./group158/reference/d1j5sa_-d1p1ma2.fasta

./group159/reference/d1adoa_-d1dhpa_.fasta

./group159/reference/d1adoa_-d1dosa_.fasta

./group159/reference/d1adoa_-d1gzga_.fasta

./group159/reference/d1adoa_-d1i2oa_.fasta

./group159/reference/d1adoa_-d1l6wa_.fasta

./group159/reference/d1adoa_-d1n8fa_.fasta

./group159/reference/d1adoa_-d1nvma2.fasta

./group159/reference/d1adoa_-d1ohla_.fasta

./group159/reference/d1adoa_-d1qfea_.fasta

./group159/reference/d1dhpa_-d1epxa_.fasta

./group159/reference/d1dhpa_-d1i2oa_.fasta

./group159/reference/d1dhpa_-d1l6wa_.fasta

./group159/reference/d1dhpa_-d1nvma2.fasta

./group159/reference/d1dhpa_-d1qfea_.fasta

./group159/reference/d1dosa_-d1euaa_.fasta

./group159/reference/d1dosa_-d1f74a_.fasta

./group159/reference/d1dosa_-d1gzga_.fasta

./group159/reference/d1dosa_-d1hl2a_.fasta

./group159/reference/d1dosa_-d1i2oa_.fasta

./group159/reference/d1dosa_-d1l6wa_.fasta

./group159/reference/d1dosa_-d1mzha_.fasta

./group159/reference/d1dosa_-d1n7ka_.fasta

./group159/reference/d1dosa_-d1nvma2.fasta

./group159/reference/d1dosa_-d1o0ya_.fasta

./group159/reference/d1dosa_-d1ohla_.fasta

./group159/reference/d1dosa_-d1qfea_.fasta

./group159/reference/d1epxa_-d1euaa_.fasta

./group159/reference/d1epxa_-d1hl2a_.fasta

./group159/reference/d1epxa_-d1i2oa_.fasta

./group159/reference/d1epxa_-d1jcla_.fasta

./group159/reference/d1epxa_-d1l6wa_.fasta

./group159/reference/d1epxa_-d1n8fa_.fasta

./group159/reference/d1epxa_-d1nvma2.fasta

./group159/reference/d1epxa_-d1qfea_.fasta

./group159/reference/d1euaa_-d1i2oa_.fasta

./group159/reference/d1f74a_-d1jcla_.fasta

./group159/reference/d1f74a_-d1n8fa_.fasta

./group159/reference/d1f74a_-d1ohla_.fasta

./group159/reference/d1gzga_-d1i2oa_.fasta

./group159/reference/d1gzga_-d1n8fa_.fasta

./group159/reference/d1hl2a_-d1i2oa_.fasta

./group159/reference/d1hl2a_-d1n8fa_.fasta

./group159/reference/d1i2oa_-d1n7ka_.fasta

./group159/reference/d1i2oa_-d1o0ya_.fasta

./group159/reference/d1i2oa_-d1ohla_.fasta

./group159/reference/d1i2oa_-d1qfea_.fasta

./group159/reference/d1jcla_-d1n8fa_.fasta

./group159/reference/d1l6wa_-d1n8fa_.fasta

./group159/reference/d1n7ka_-d1n8fa_.fasta

./group159/reference/d1n8fa_-d1nvma2.fasta

./group159/reference/d1n8fa_-d1o0ya_.fasta

./group159/reference/d1n8fa_-d1ohla_.fasta

./group159/reference/d1n8fa_-d1qfea_.fasta

./group159/reference/d1n8fa_-d1ub3a_.fasta

./group159/reference/d1nvma2-d1ohla_.fasta

./group161/reference/d1dxea_-d1m3ua_.fasta

./group161/reference/d1kbla1-d1muma_.fasta

./group163/reference/d1ezwa_-d1nfp__.fasta

./group165/reference/d1eexa_-d7reqa1.fasta

./group166/reference/d1b16a_-d1o0sa1.fasta

./group166/reference/d1bgva1-d1kepa_.fasta

./group166/reference/d1bgva1-d1kewa_.fasta

./group166/reference/d1eno__-d1gz3a1.fasta

./group166/reference/d1eno__-d1o0sa1.fasta

./group166/reference/d1eny__-d1o0sa1.fasta

./group166/reference/d1fmca_-d1gz3a1.fasta

./group166/reference/d1fmca_-d1o0sa1.fasta

./group166/reference/d1g0oa_-d1gz3a1.fasta

./group166/reference/d1g0oa_-d1o0sa1.fasta

./group166/reference/d1gcoa_-d1o0sa1.fasta

./group166/reference/d1gz3a1-d1iy8a_.fasta

./group166/reference/d1gz3a1-d1ja9a_.fasta

./group166/reference/d1gz3a1-d1n5da_.fasta

./group166/reference/d1gz3a1-d2ae2a_.fasta

./group166/reference/d1hwxa1-d1kewa_.fasta

./group166/reference/d1hxha_-d1o0sa1.fasta

./group166/reference/d1iy8a_-d1o0sa1.fasta

./group166/reference/d1ja9a_-d1o0sa1.fasta

./group166/reference/d1kepa_-d1o0sa1.fasta

./group166/reference/d1o0sa1-d1oaa__.fasta

./group167/reference/d1cjca1-d1ebda2.fasta

./group167/reference/d1cjca1-d3lada2.fasta

./group167/reference/d1fcda2-d1lvl_2.fasta

./group167/reference/d1fcda2-d1ojt_2.fasta

./group167/reference/d1fcda2-d1onfa2.fasta

./group169/reference/d1h16a_-d1hk8a_.fasta

./group169/reference/d1h16a_-d1rlr_2.fasta

./group169/reference/d1hk8a_-d1rlr_2.fasta

./group171/reference/d1dcea3-d1jl5a_.fasta

./group171/reference/d1h6ua2-d1jl5a_.fasta

./group171/reference/d1igra1-d1jl5a_.fasta

./group171/reference/d1jl5a_-d1nqla1.fasta

./group171/reference/d1jl5a_-d1nqla2.fasta

./group171/reference/d1jl5a_-d1ogqa_.fasta

./group171/reference/d1jl5a_-d1ozna_.fasta

./group171/reference/d1nqla1-d1ozna_.fasta

./group176/reference/d1a04a2-d1qo0d_.fasta

./group176/reference/d1a2oa1-d1qo0d_.fasta

./group176/reference/d1m2fa_-d1qo0d_.fasta

./group176/reference/d1m2fa_-d1tmy__.fasta

./group177/reference/d1bvyf_-d1d4aa_.fasta

./group177/reference/d1d4aa_-d1ja1a2.fasta

./group177/reference/d1d4aa_-d2fcr__.fasta

./group177/reference/d1qr2a_-d2fcr__.fasta

./group181/reference/d1cf9a1-d1k9vf_.fasta

./group181/reference/d1cf9a1-d1l9xa_.fasta

./group182/reference/d1ep3b2-d1fdr_2.fasta

./group183/reference/d1a8h_2-d1ej2a_.fasta

./group183/reference/d1a8h_2-d1jhda2.fasta

./group183/reference/d1a8h_2-d1qjca_.fasta

./group183/reference/d1f7ua2-d1jhda2.fasta

./group183/reference/d1f7ua2-d1k4ma_.fasta

./group183/reference/d1f7ua2-d1n2ea_.fasta

./group183/reference/d1g8fa2-d1j09a2.fasta

./group183/reference/d1g8fa2-d1jila_.fasta

./group183/reference/d1gtra2-d1jila_.fasta

./group183/reference/d1gtra2-d1k4ma_.fasta

./group183/reference/d1j09a2-d1k4ma_.fasta

./group183/reference/d1jhda2-d1jila_.fasta

./group183/reference/d1jhda2-d1n2ea_.fasta

./group183/reference/d1jila_-d1qjca_.fasta

./group184/reference/d1efpa1-d1k92a1.fasta

./group184/reference/d1efpa1-d1kqpa_.fasta

./group184/reference/d1efva1-d1k92a1.fasta

./group184/reference/d1efva1-d1kqpa_.fasta

./group184/reference/d1efva1-d1sur__.fasta

./group184/reference/d1efvb_-d1sur__.fasta

./group184/reference/d1j20a1-d1o97d1.fasta

./group184/reference/d1jmva_-d1kqpa_.fasta

./group184/reference/d1k92a1-d1o97d1.fasta

./group184/reference/d1kqpa_-d1o97d1.fasta

./group184/reference/d1kqpa_-d1sur__.fasta

./group184/reference/d1o97d1-d1sur__.fasta

./group186/reference/d1a9xa3-d1m0wa1.fasta

./group186/reference/d1b6ra2-d1e4ea1.fasta

./group186/reference/d1b6ra2-d1ehia1.fasta

./group186/reference/d1b6ra2-d1m0wa1.fasta

./group186/reference/d1e4ea1-d1i7na1.fasta

./group186/reference/d1e4ea1-d1m0wa1.fasta

./group186/reference/d1ehia1-d1gsa_1.fasta

./group186/reference/d1ehia1-d1gsoa2.fasta

./group186/reference/d1ehia1-d1i7na1.fasta

./group186/reference/d1ehia1-d1m0wa1.fasta

./group186/reference/d1gsa_1-d1gsoa2.fasta

./group186/reference/d1gsa_1-d1iow_1.fasta

./group186/reference/d1gsoa2-d1m0wa1.fasta

./group186/reference/d1gsoa2-d2hgsa1.fasta

./group186/reference/d1i7na1-d1kjqa2.fasta

./group186/reference/d1i7na1-d1m0wa1.fasta

./group186/reference/d1kjqa2-d1m0wa1.fasta

./group187/reference/d1dhs__-d1ovma1.fasta

./group187/reference/d1dhs__-d1pvda1.fasta

./group187/reference/d1dhs__-d1zpda1.fasta

./group187/reference/d1efva2-d1ovma1.fasta

./group187/reference/d1hzzc_-d1ovma1.fasta

./group187/reference/d1o97d2-d1ovma1.fasta

./group188/reference/d1fsz_1-d1oi2a1.fasta

./group188/reference/d1ofua1-d1oi2a1.fasta

./group188/reference/d1oi2a1-d1tubb1.fasta

./group190/reference/d1bfd_3-d1pvda2.fasta

./group190/reference/d1dtwb1-d1keka1.fasta

./group190/reference/d1dtwb1-d1keka2.fasta

./group190/reference/d1dtwb1-d1ovma3.fasta

./group190/reference/d1dtwb1-d1poxa2.fasta

./group190/reference/d1dtwb1-d1poxa3.fasta

./group190/reference/d1dtwb1-d1pvda3.fasta

./group190/reference/d1gpua2-d1jsca2.fasta

./group190/reference/d1gpua2-d1keka1.fasta

./group190/reference/d1gpua2-d1keka2.fasta

./group190/reference/d1gpua2-d1poxa2.fasta

./group190/reference/d1gpua2-d1pvda3.fasta

./group190/reference/d1jsca2-d1qgda2.fasta

./group190/reference/d1keka1-d1keka2.fasta

./group190/reference/d1keka1-d1poxa2.fasta

./group190/reference/d1keka1-d1poxa3.fasta

./group190/reference/d1keka1-d1qgda2.fasta

./group190/reference/d1keka2-d1ovma2.fasta

./group190/reference/d1keka2-d1qgda1.fasta

./group190/reference/d1keka2-d1zpda2.fasta

./group190/reference/d1ovma2-d1poxa3.fasta

./group190/reference/d1ovma2-d1pvda3.fasta

./group190/reference/d1ovma2-d1qgda1.fasta

./group190/reference/d1ovma3-d1poxa2.fasta

./group190/reference/d1ovma3-d1pvda2.fasta

./group190/reference/d1ovma3-d1zpda2.fasta

./group190/reference/d1poxa2-d1pvda3.fasta

./group190/reference/d1poxa2-d1qgda1.fasta

./group190/reference/d1poxa2-d1qgda2.fasta

./group190/reference/d1poxa2-d1zpda3.fasta

./group190/reference/d1poxa3-d1qgda1.fasta

./group190/reference/d1pvda2-d1pvda3.fasta

./group190/reference/d1pvda2-d1qgda2.fasta

./group190/reference/d1pvda3-d1qgda1.fasta

./group190/reference/d1qgda1-d1zpda2.fasta

./group191/reference/d1bg2__-d1dj3a_.fasta

./group191/reference/d1bg2__-d1e32a2.fasta

./group191/reference/d1bg2__-d1f60a3.fasta

./group191/reference/d1bg2__-d1g3qa_.fasta

./group191/reference/d1bg2__-d1g6ha_.fasta

./group191/reference/d1bg2__-d1hyqa_.fasta

./group191/reference/d1bg2__-d1iwea_.fasta

./group191/reference/d1bg2__-d1j99a_.fasta

./group191/reference/d1bg2__-d1ofha_.fasta

./group191/reference/d1bg2__-d1pf4a1.fasta

./group191/reference/d1bg2__-d1qf5a_.fasta

./group191/reference/d1cp2a_-d1fmja_.fasta

./group191/reference/d1cp2a_-d1j99a_.fasta

./group191/reference/d1cp2a_-d1pf4a1.fasta

./group191/reference/d1d2na_-d1g3qa_.fasta

./group191/reference/d1d2na_-d1iwea_.fasta

./group191/reference/d1dj3a_-d1e79a3.fasta

./group191/reference/d1dj3a_-d1fmja_.fasta

./group191/reference/d1e32a2-d1iwea_.fasta

./group191/reference/d1e32a2-d1j99a_.fasta

./group191/reference/d1e79a3-d1eg7a_.fasta

./group191/reference/d1e79a3-d1f60a3.fasta

./group191/reference/d1e79a3-d1iwea_.fasta

./group191/reference/d1e79a3-d1pf4a1.fasta

./group191/reference/d1e79a3-d1qf5a_.fasta

./group191/reference/d1eg7a_-d1fmja_.fasta

./group191/reference/d1eg7a_-d1ofha_.fasta

./group191/reference/d1eg7a_-d1pf4a1.fasta

./group191/reference/d1f60a3-d1fmja_.fasta

./group191/reference/d1f60a3-d1j99a_.fasta

./group191/reference/d1f60a3-d1njfa_.fasta

./group191/reference/d1f60a3-d1uaaa1.fasta

./group191/reference/d1fmja_-d1fnna2.fasta

./group191/reference/d1fmja_-d1g6ha_.fasta

./group191/reference/d1fmja_-d1hyqa_.fasta

./group191/reference/d1fmja_-d1mt0a_.fasta

./group191/reference/d1fmja_-d1n25a_.fasta

./group191/reference/d1fmja_-d1pf4a1.fasta

./group191/reference/d1fnna2-d1pf4a1.fasta

./group191/reference/d1g2912-d1iwea_.fasta

./group191/reference/d1g2912-d1j99a_.fasta

./group191/reference/d1g3qa_-d1j99a_.fasta

./group191/reference/d1g3qa_-d1pf4a1.fasta

./group191/reference/d1g6ha_-d1j99a_.fasta

./group191/reference/d1hyqa_-d1j99a_.fasta

./group191/reference/d1hyqa_-d1pf4a1.fasta

./group191/reference/d1iwea_-d1pf4a1.fasta

./group191/reference/d1j99a_-d1ofha_.fasta

./group191/reference/d1j99a_-d1pf4a1.fasta

./group191/reference/d1n25a_-d1pf4a1.fasta

./group191/reference/d1njfa_-d1qf5a_.fasta

./group191/reference/d1ofha_-d1pf4a1.fasta

./group191/reference/d1pf4a1-d1uaaa1.fasta

./group193/reference/d1c3pa_-d1d3va_.fasta

./group196/reference/d1d5ra2-d1ohea1.fasta

./group196/reference/d1eeoa_-d1ohea1.fasta

./group196/reference/d1ikza_-d1jlna_.fasta

./group196/reference/d1jlna_-d1ohea1.fasta

./group196/reference/d1lara1-d1mkp__.fasta

./group196/reference/d1lara1-d1ohea1.fasta

./group196/reference/d1lara2-d1mkp__.fasta

./group196/reference/d1lara2-d1ohea1.fasta

./group196/reference/d1mkp__-d1ohea1.fasta

./group198/reference/d1a8l_1-d1a8y_1.fasta

./group198/reference/d1a8l_1-d1eema2.fasta

./group198/reference/d1a8l_1-d1foha3.fasta

./group198/reference/d1a8l_1-d1hd2a_.fasta

./group198/reference/d1a8l_2-d1a8y_2.fasta

./group198/reference/d1a8l_2-d1hd2a_.fasta

./group198/reference/d1a8l_2-d1jfua_.fasta

./group198/reference/d1a8y_1-d1eeja1.fasta

./group198/reference/d1a8y_1-d1eema2.fasta

./group198/reference/d1a8y_1-d1foha3.fasta

./group198/reference/d1a8y_1-d1jfua_.fasta

./group198/reference/d1a8y_1-d1m2da_.fasta

./group198/reference/d1a8y_2-d1eeja1.fasta

./group198/reference/d1a8y_2-d1eema2.fasta

./group198/reference/d1a8y_2-d1fb6a_.fasta

./group198/reference/d1a8y_2-d1foha3.fasta

./group198/reference/d1a8y_2-d1gh2a_.fasta

./group198/reference/d1a8y_2-d1hd2a_.fasta

./group198/reference/d1a8y_2-d1knga_.fasta

./group198/reference/d1a8y_2-d1kte__.fasta

./group198/reference/d1a8y_2-d1n8ja_.fasta

./group198/reference/d1a8y_2-d1quwa_.fasta

./group198/reference/d1a8y_2-d2trxa_.fasta

./group198/reference/d1bjx__-d1eeja1.fasta

./group198/reference/d1bjx__-d1jfua_.fasta

./group198/reference/d1bjx__-d1knga_.fasta

./group198/reference/d1eeja1-d1erv__.fasta

./group198/reference/d1eeja1-d1n8ja_.fasta

./group198/reference/d1eema2-d1ep7a_.fasta

./group198/reference/d1eema2-d1erv__.fasta

./group198/reference/d1eema2-d1f9ma_.fasta

./group198/reference/d1eema2-d1fb6a_.fasta

./group198/reference/d1eema2-d1g7ea_.fasta

./group198/reference/d1eema2-d1gh2a_.fasta

./group198/reference/d1eema2-d1hd2a_.fasta

./group198/reference/d1eema2-d1hyua3.fasta

./group198/reference/d1eema2-d1jfua_.fasta

./group198/reference/d1eema2-d1m2da_.fasta

./group198/reference/d1eema2-d1mek__.fasta

./group198/reference/d1eema2-d1n8ja_.fasta

./group198/reference/d1eema2-d1quwa_.fasta

./group198/reference/d1erv__-d1foha3.fasta

./group198/reference/d1erv__-d1m2da_.fasta

./group198/reference/d1f9ma_-d1foha3.fasta

./group198/reference/d1f9ma_-d1m2da_.fasta

./group198/reference/d1fb6a_-d1m2da_.fasta

./group198/reference/d1foha3-d1gh2a_.fasta

./group198/reference/d1foha3-d1hyua3.fasta

./group198/reference/d1foha3-d1kte__.fasta

./group198/reference/d1foha3-d1m2da_.fasta

./group198/reference/d1foha3-d1quwa_.fasta

./group198/reference/d1foha3-d2trxa_.fasta

./group198/reference/d1g7ea_-d1knga_.fasta

./group198/reference/d1g7ea_-d1n8ja_.fasta

./group198/reference/d1gh2a_-d1hd2a_.fasta

./group198/reference/d1gh2a_-d1hyua3.fasta

./group198/reference/d1hd2a_-d1kte__.fasta

./group198/reference/d1hyua3-d1mek__.fasta

./group198/reference/d1hyua3-d1thx__.fasta

./group198/reference/d1jfua_-d1m2da_.fasta

./group198/reference/d1knga_-d1m2da_.fasta

./group198/reference/d1kte__-d1n8ja_.fasta

./group198/reference/d1m2da_-d1mek__.fasta

./group198/reference/d1m2da_-d1n8ja_.fasta

./group198/reference/d1m2da_-d1quwa_.fasta

./group198/reference/d1m2da_-d1thx__.fasta

./group198/reference/d1m2da_-d2trxa_.fasta

./group198/reference/d1n8ja_-d1quwa_.fasta

./group199/reference/d1dtwb2-d1itza3.fasta

./group199/reference/d1dtwb2-d1qgda3.fasta

./group201/reference/d1gyta1-d1hjza_.fasta

./group203/reference/d1avqa_-d1ckqa_.fasta

./group203/reference/d1avqa_-d1fiua_.fasta

./group203/reference/d1avqa_-d1vsra_.fasta

./group203/reference/d1cfr__-d1ckqa_.fasta

./group203/reference/d1cfr__-d3pvia_.fasta

./group203/reference/d1ckqa_-d1knva_.fasta

./group203/reference/d1ckqa_-d1m0da_.fasta

./group203/reference/d1ckqa_-d3pvia_.fasta

./group203/reference/d1d02a_-d1f1za2.fasta

./group203/reference/d1d02a_-d1gefa_.fasta

./group203/reference/d1d02a_-d1knva_.fasta

./group203/reference/d1dmua_-d1fiua_.fasta

./group203/reference/d1dmua_-d1gefa_.fasta

./group203/reference/d1dmua_-d3pvia_.fasta

./group203/reference/d1f1za2-d1knva_.fasta

./group203/reference/d1fiua_-d1gefa_.fasta

./group203/reference/d1fiua_-d1j23a_.fasta

./group203/reference/d1fiua_-d3pvia_.fasta

./group203/reference/d1gefa_-d2foka4.fasta

./group203/reference/d1gefa_-d3pvia_.fasta

./group203/reference/d1j23a_-d1m0da_.fasta

./group203/reference/d1knva_-d1vsra_.fasta

./group203/reference/d1knva_-d3pvia_.fasta

./group203/reference/d1m0da_-d3pvia_.fasta

./group203/reference/d1vsra_-d3pvia_.fasta

./group203/reference/d2foka4-d3pvia_.fasta

./group205/reference/d1bdg_2-d1bu6o1.fasta

./group205/reference/d1bdg_2-d1bupa1.fasta

./group205/reference/d1bdg_2-d1czan1.fasta

./group205/reference/d1bdg_2-d1czan3.fasta

./group205/reference/d1bdg_2-d1g99a1.fasta

./group205/reference/d1bdg_2-d1ig8a1.fasta

./group205/reference/d1bdg_2-d1mwma2.fasta

./group205/reference/d1bu6o1-d1bu6o2.fasta

./group205/reference/d1bu6o1-d1czan2.fasta

./group205/reference/d1bu6o1-d1huxa_.fasta

./group205/reference/d1bu6o1-d1ig8a2.fasta

./group205/reference/d1bu6o2-d1czan1.fasta

./group205/reference/d1bu6o2-d1e4ft1.fasta

./group205/reference/d1bu6o2-d1ig8a1.fasta

./group205/reference/d1bu6o2-d1jcfa1.fasta

./group205/reference/d1bu6o2-d1mwma2.fasta

./group205/reference/d1bupa1-d1czan2.fasta

./group205/reference/d1bupa1-d1g99a1.fasta

./group205/reference/d1bupa1-d1ig8a2.fasta

./group205/reference/d1bupa2-d1czan2.fasta

./group205/reference/d1bupa2-d1g99a2.fasta

./group205/reference/d1bupa2-d1ig8a2.fasta

./group205/reference/d1czan1-d1jcfa1.fasta

./group205/reference/d1czan2-d1czan3.fasta

./group205/reference/d1czan2-d1g99a1.fasta

./group205/reference/d1czan3-d1g99a1.fasta

./group205/reference/d1e4ft1-d1g99a2.fasta

./group205/reference/d1g99a1-d1g99a2.fasta

./group205/reference/d1g99a1-d1ig8a2.fasta

./group205/reference/d1g99a1-d1j6za2.fasta

./group205/reference/d1g99a1-d1mwma2.fasta

./group205/reference/d1g99a1-d1nbwa3.fasta

./group205/reference/d1g99a2-d1ig8a2.fasta

./group205/reference/d1g99a2-d1j6za2.fasta

./group205/reference/d1ig8a1-d1ig8a2.fasta

./group205/reference/d1ig8a1-d1nbwa3.fasta

./group205/reference/d1ig8a2-d1mwma2.fasta

./group205/reference/d1ig8a2-d1nbwa3.fasta

./group206/reference/d1hjra_-d1ih7a1.fasta

./group206/reference/d1hjra_-d1jl1a_.fasta

./group206/reference/d1hjra_-d1kfsa1.fasta

./group206/reference/d1hjra_-d1qtma1.fasta

./group206/reference/d1hjra_-d1t7pa1.fasta

./group206/reference/d1i39a_-d1kfsa1.fasta

./group206/reference/d1i39a_-d1l3sa1.fasta

./group206/reference/d1i39a_-d1t7pa1.fasta

./group206/reference/d1i39a_-d1tgoa1.fasta

./group206/reference/d1ih7a1-d1l3sa1.fasta

./group206/reference/d1ih7a1-d1qtma1.fasta

./group206/reference/d1io2a_-d1kfsa1.fasta

./group206/reference/d1io2a_-d1qtma1.fasta

./group206/reference/d1jl1a_-d1kfsa1.fasta

./group206/reference/d1jl1a_-d1l3sa1.fasta

./group206/reference/d1jl1a_-d1tgoa1.fasta

./group206/reference/d1qtma1-d1tgoa1.fasta

./group207/reference/d1dt9a1-d1fjgk_.fasta

./group207/reference/d1dt9a1-d1ilya_.fasta

./group209/reference/d1gyta2-d1h8la2.fasta

./group209/reference/d1gyta2-d1jqga1.fasta

./group209/reference/d1gyta2-d1kwma1.fasta

./group209/reference/d1gyta2-d1m4la_.fasta

./group209/reference/d1gyta2-d1obr__.fasta

./group209/reference/d1jqga1-d1lam_2.fasta

./group209/reference/d1jqga1-d1loka_.fasta

./group209/reference/d1kwma1-d1lam_2.fasta

./group209/reference/d1kwma1-d1loka_.fasta

./group209/reference/d1lam_2-d1m4la_.fasta

./group209/reference/d1lam_2-d1obr__.fasta

./group209/reference/d1loka_-d1obr__.fasta

./group210/reference/d1a4ia2-d1bgva2.fasta

./group210/reference/d1a4ia2-d1c1da2.fasta

./group210/reference/d1a4ia2-d1gtma2.fasta

./group210/reference/d1a4ia2-d1hwxa2.fasta

./group210/reference/d1a4ia2-d1leha2.fasta

./group210/reference/d1a4ia2-d1nvta2.fasta

./group210/reference/d1a4ia2-d1nyta2.fasta

./group210/reference/d1a4ia2-d1o0sa2.fasta

./group210/reference/d1b0aa2-d1bgva2.fasta

./group210/reference/d1b0aa2-d1c1da2.fasta

./group210/reference/d1b0aa2-d1gtma2.fasta

./group210/reference/d1b0aa2-d1hwxa2.fasta

./group210/reference/d1b0aa2-d1nyta2.fasta

./group210/reference/d1bgva2-d1edza2.fasta

./group210/reference/d1bgva2-d1lu9a2.fasta

./group210/reference/d1bgva2-d1npya2.fasta

./group210/reference/d1bgva2-d1nvta2.fasta

./group210/reference/d1bgva2-d1nyta2.fasta

./group210/reference/d1bgva2-d1o0sa2.fasta

./group210/reference/d1c1da2-d1edza2.fasta

./group210/reference/d1c1da2-d1lu9a2.fasta

./group210/reference/d1c1da2-d1npya2.fasta

./group210/reference/d1c1da2-d1nvta2.fasta

./group210/reference/d1c1da2-d1nyta2.fasta

./group210/reference/d1c1da2-d1o0sa2.fasta

./group210/reference/d1edza2-d1hwxa2.fasta

./group210/reference/d1edza2-d1nyta2.fasta

./group210/reference/d1edza2-d1o0sa2.fasta

./group210/reference/d1gtma2-d1lu9a2.fasta

./group210/reference/d1gtma2-d1npya2.fasta

./group210/reference/d1gtma2-d1nyta2.fasta

./group210/reference/d1gtma2-d1o0sa2.fasta

./group210/reference/d1hwxa2-d1lu9a2.fasta

./group210/reference/d1hwxa2-d1nyta2.fasta

./group210/reference/d1hwxa2-d1o0sa2.fasta

./group210/reference/d1leha2-d1lu9a2.fasta

./group210/reference/d1leha2-d1npya2.fasta

./group210/reference/d1leha2-d1nvta2.fasta

./group210/reference/d1leha2-d1nyta2.fasta

./group210/reference/d1leha2-d1o0sa2.fasta

./group210/reference/d1lu9a2-d1npya2.fasta

./group210/reference/d1lu9a2-d1nyta2.fasta

./group210/reference/d1lu9a2-d1o0sa2.fasta

./group210/reference/d1npya2-d1o0sa2.fasta

./group210/reference/d1nvta2-d1o0sa2.fasta

./group212/reference/d1bd3a_-d1dqna_.fasta

./group212/reference/d1bd3a_-d1fsga_.fasta

./group212/reference/d1bd3a_-d1gph11.fasta

./group212/reference/d1dqna_-d1i5ea_.fasta

./group212/reference/d1i5ea_-d1lh0a_.fasta

./group214/reference/d1af7_2-d1i4wa_.fasta

./group214/reference/d1dcta_-d1fp2a2.fasta

./group214/reference/d1dcta_-d1p1ca_.fasta

./group214/reference/d1dcta_-d1xvaa_.fasta

./group214/reference/d1hnna_-d1iy9a_.fasta

./group214/reference/d1i4wa_-d1iy9a_.fasta

./group214/reference/d1i4wa_-d1nv8a_.fasta

./group214/reference/d1i4wa_-d1nw3a_.fasta

./group214/reference/d1i4wa_-d1xvaa_.fasta

./group214/reference/d1i4wa_-d6mhta_.fasta

./group214/reference/d1iy9a_-d6mhta_.fasta

./group214/reference/d1jqea_-d1nw3a_.fasta

./group214/reference/d1jqea_-d1xvaa_.fasta

./group214/reference/d1jqea_-d1yub__.fasta

./group214/reference/d1kywa2-d1qama_.fasta

./group214/reference/d1kywa2-d1yub__.fasta

./group214/reference/d1kywa2-d6mhta_.fasta

./group214/reference/d1l9ka_-d1nv8a_.fasta

./group214/reference/d1l9ka_-d1qama_.fasta

./group214/reference/d1l9ka_-d1yub__.fasta

./group214/reference/d1l9ka_-d6mhta_.fasta

./group214/reference/d1p1ca_-d1qama_.fasta

./group214/reference/d1xvaa_-d6mhta_.fasta

./group214/reference/d1yub__-d6mhta_.fasta

./group215/reference/d1ajsa_-d1gtxa_.fasta

./group215/reference/d1ajsa_-d1ibja_.fasta

./group215/reference/d1b9ha_-d1c4ka2.fasta

./group215/reference/d1b9ha_-d1c7na_.fasta

./group215/reference/d1bs0a_-d1yaaa_.fasta

./group215/reference/d1c4ka2-d1c7na_.fasta

./group215/reference/d1c4ka2-d1gdea_.fasta

./group215/reference/d1c4ka2-d1j32a_.fasta

./group215/reference/d1c4ka2-d2ay1a_.fasta

./group215/reference/d1c4ka2-d2dkb__.fasta

./group215/reference/d1c4ka2-d7aata_.fasta

./group215/reference/d1c7na_-d1ibja_.fasta

./group215/reference/d1c7na_-d7aata_.fasta

./group215/reference/d1elua_-d1tpla_.fasta

./group215/reference/d1gdea_-d1gtxa_.fasta

./group215/reference/d1gtxa_-d1qisa_.fasta

./group215/reference/d1gtxa_-d1yaaa_.fasta

./group215/reference/d1gtxa_-d3tata_.fasta

./group215/reference/d1gtxa_-d7aata_.fasta

./group215/reference/d1kl1a_-d3tata_.fasta

./group215/reference/d2oata_-d7aata_.fasta

./group216/reference/d1e5ka_-d1gx4a_.fasta

./group216/reference/d1e5ka_-d1nf5b_.fasta

./group216/reference/d1gx4a_-d1h7ea_.fasta

./group216/reference/d1gx4a_-d1hm9a2.fasta

./group216/reference/d1gx4a_-d1hv9a2.fasta

./group216/reference/d1gx4a_-d1nf5b_.fasta

./group216/reference/d1hm9a2-d1nf5b_.fasta

./group216/reference/d1hv9a2-d1nf5b_.fasta

./group217/reference/d1ac5__-d1bu8a2.fasta

./group217/reference/d1ac5__-d1ea5a_.fasta

./group217/reference/d1ac5__-d1jkma_.fasta

./group217/reference/d1ac5__-d1lnsa3.fasta

./group217/reference/d1ac5__-d1lzla_.fasta

./group217/reference/d1bn7a_-d1ea5a_.fasta

./group217/reference/d1bn7a_-d1gkla_.fasta

./group217/reference/d1bn7a_-d1llfa_.fasta

./group217/reference/d1bn7a_-d1mx1a_.fasta

./group217/reference/d1bu8a2-d1gkla_.fasta

./group217/reference/d1bu8a2-d1ivya_.fasta

./group217/reference/d1bu8a2-d1ju3a2.fasta

./group217/reference/d1bu8a2-d1lzla_.fasta

./group217/reference/d1cpy__-d1tca__.fasta

./group217/reference/d1dqza_-d1jjia_.fasta

./group217/reference/d1ea5a_-d1iz7a_.fasta

./group217/reference/d1ea5a_-d1lnsa3.fasta

./group217/reference/d1ea5a_-d1qlwa_.fasta

./group217/reference/d1ea5a_-d1tca__.fasta

./group217/reference/d1ex9a_-d1lnsa3.fasta

./group217/reference/d1gkla_-d1iz7a_.fasta

./group217/reference/d1ivya_-d1tca__.fasta

./group217/reference/d1iz7a_-d1llfa_.fasta

./group217/reference/d1iz7a_-d1lnsa3.fasta

./group217/reference/d1iz7a_-d1lzla_.fasta

./group217/reference/d1iz7a_-d1mx1a_.fasta

./group217/reference/d1ju3a2-d1tca__.fasta

./group217/reference/d1ku0a_-d1llfa_.fasta

./group217/reference/d1llfa_-d1qlwa_.fasta

./group217/reference/d1llfa_-d1tca__.fasta

./group217/reference/d1lnsa3-d1mx1a_.fasta

./group217/reference/d1lnsa3-d1tca__.fasta

./group217/reference/d1mx1a_-d1qlwa_.fasta

./group224/reference/d1a1s_2-d1otha1.fasta

./group224/reference/d1duvg1-d1js1x2.fasta

./group224/reference/d1js1x1-d1js1x2.fasta

./group224/reference/d1js1x2-d1ml4a1.fasta

./group224/reference/d1js1x2-d1otha1.fasta

./group227/reference/d1c7qa_-d1moq__.fasta

./group230/reference/d1k2yx1-d1k2yx3.fasta

./group230/reference/d1k2yx1-d3pmga2.fasta

./group230/reference/d1k2yx1-d3pmga3.fasta

./group230/reference/d1k2yx2-d3pmga3.fasta

./group230/reference/d1k2yx3-d1kfia1.fasta

./group230/reference/d1kfia1-d3pmga2.fasta

./group230/reference/d1kfia1-d3pmga3.fasta

./group230/reference/d3pmga1-d3pmga2.fasta

./group230/reference/d3pmga1-d3pmga3.fasta

./group230/reference/d3pmga2-d3pmga3.fasta

./group232/reference/d1f0ka_-d1jixa_.fasta

./group232/reference/d1f0ka_-d1l5wa_.fasta

./group234/reference/d1hrka_-d1qgoa_.fasta

./group235/reference/d1m1na_-d1psza_.fasta

./group235/reference/d1m1nb_-d1n2za_.fasta

./group235/reference/d1miob_-d1n2za_.fasta

./group235/reference/d1n2za_-d1psza_.fasta

./group236/reference/d1dp4a_-d8abp__.fasta

./group236/reference/d1gca__-d2liv__.fasta

./group236/reference/d1jyea_-d1pea__.fasta

./group236/reference/d1jyea_-d2liv__.fasta

./group237/reference/d1a8e__-d1al3__.fasta

./group237/reference/d1a8e__-d1dpe__.fasta

./group237/reference/d1a8e__-d1j1na_.fasta

./group237/reference/d1a8e__-d1jeta_.fasta

./group237/reference/d1a8e__-d1mqda_.fasta

./group237/reference/d1a8e__-d1pot__.fasta

./group237/reference/d1a99a_-d1dpe__.fasta

./group237/reference/d1a99a_-d1lst__.fasta

./group237/reference/d1a99a_-d1wdna_.fasta

./group237/reference/d1al3__-d1atg__.fasta

./group237/reference/d1al3__-d1dpe__.fasta

./group237/reference/d1al3__-d1j1na_.fasta

./group237/reference/d1al3__-d1jeta_.fasta

./group237/reference/d1amf__-d1i6aa_.fasta

./group237/reference/d1amf__-d1jeta_.fasta

./group237/reference/d1atg__-d1i6aa_.fasta

./group237/reference/d1atg__-d1jeta_.fasta

./group237/reference/d1atg__-d1mqda_.fasta

./group237/reference/d1atg__-d1wdna_.fasta

./group237/reference/d1dpe__-d1eu8a_.fasta

./group237/reference/d1dpe__-d1i6aa_.fasta

./group237/reference/d1dpe__-d1ixh__.fasta

./group237/reference/d1dpe__-d1j1na_.fasta

./group237/reference/d1dpe__-d1lst__.fasta

./group237/reference/d1dpe__-d1mqda_.fasta

./group237/reference/d1dpe__-d1wdna_.fasta

./group237/reference/d1dpe__-d3thia_.fasta

./group237/reference/d1eu8a_-d1jeta_.fasta

./group237/reference/d1eu8a_-d1mqda_.fasta

./group237/reference/d1i6aa_-d1ixh__.fasta

./group237/reference/d1i6aa_-d1j1na_.fasta

./group237/reference/d1i6aa_-d1pot__.fasta

./group237/reference/d1i6aa_-d3thia_.fasta

./group237/reference/d1ixh__-d1jeta_.fasta

./group237/reference/d1j1na_-d1mqda_.fasta

./group237/reference/d1j1na_-d1pot__.fasta

./group237/reference/d1jeta_-d1sbp__.fasta

./group237/reference/d1lst__-d1pot__.fasta

./group237/reference/d1mqda_-d3mbp__.fasta

./group237/reference/d1mqda_-d3thia_.fasta

./group237/reference/d1wdna_-d3thia_.fasta

./group238/reference/d1afwa2-d1e5ma1.fasta

./group238/reference/d1afwa2-d1ek4a1.fasta

./group238/reference/d1afwa2-d1kas_1.fasta

./group238/reference/d1afwa2-d1ox0a1.fasta

./group238/reference/d1bi5a2-d1e5ma1.fasta

./group238/reference/d1e5ma1-d1e5ma2.fasta

./group238/reference/d1e5ma2-d1hnja1.fasta

./group238/reference/d1e5ma2-d1mzja1.fasta

./group238/reference/d1ek4a2-d1hnja1.fasta

./group238/reference/d1ek4a2-d1m3ka1.fasta

./group238/reference/d1hnja2-d1kas_1.fasta

./group241/reference/d153l__-d1chka_.fasta

./group241/reference/d153l__-d1lw9a_.fasta

./group241/reference/d1b9oa_-d1chka_.fasta

./group241/reference/d1b9oa_-d1dxja_.fasta

./group241/reference/d1b9oa_-d1k28a3.fasta

./group241/reference/d1b9oa_-d1lw9a_.fasta

./group241/reference/d1b9oa_-d1qgia_.fasta

./group241/reference/d1chka_-d1gd6a_.fasta

./group241/reference/d1chka_-d1qsaa2.fasta

./group241/reference/d1chka_-d2eql__.fasta

./group241/reference/d1dxja_-d1k28a3.fasta

./group241/reference/d1dxja_-d1qgia_.fasta

./group241/reference/d1dxja_-d1qsaa2.fasta

./group241/reference/d1dxja_-d2eql__.fasta

./group241/reference/d1k28a3-d2eql__.fasta

./group241/reference/d1lw9a_-d1qsaa2.fasta

./group242/reference/d1avpa_-d1cvza_.fasta

./group242/reference/d1avpa_-d1deua_.fasta

./group242/reference/d1avpa_-d1e2ta_.fasta

./group242/reference/d1avpa_-d1fh0a_.fasta

./group242/reference/d1avpa_-d1iu4a_.fasta

./group242/reference/d1avpa_-d1l9na4.fasta

./group242/reference/d1avpa_-d1me4a_.fasta

./group242/reference/d1avpa_-d2cb5a_.fasta

./group242/reference/d1avpa_-d7pcka_.fasta

./group242/reference/d1cv8__-d1e2ta_.fasta

./group242/reference/d1cv8__-d1f13a4.fasta

./group242/reference/d1cv8__-d1g0da4.fasta

./group242/reference/d1cv8__-d1gx3a_.fasta

./group242/reference/d1cv8__-d1iu4a_.fasta

./group242/reference/d1cv8__-d1l9na4.fasta

./group242/reference/d1cv8__-d1qmya_.fasta

./group242/reference/d1cv8__-d2cb5a_.fasta

./group242/reference/d1cvza_-d1euva_.fasta

./group242/reference/d1cvza_-d1gx3a_.fasta

./group242/reference/d1cvza_-d1iu4a_.fasta

./group242/reference/d1cvza_-d1l9na4.fasta

./group242/reference/d1cvza_-d1qmya_.fasta

./group242/reference/d1deua_-d1euva_.fasta

./group242/reference/d1deua_-d1iu4a_.fasta

./group242/reference/d1e2ta_-d1gmya_.fasta

./group242/reference/d1e2ta_-d1iwda_.fasta

./group242/reference/d1e2ta_-d1nbfa_.fasta

./group242/reference/d1e2ta_-d1qmya_.fasta

./group242/reference/d1e2ta_-d2cb5a_.fasta

./group242/reference/d1e2ta_-d7pcka_.fasta

./group242/reference/d1euva_-d1f13a4.fasta

./group242/reference/d1euva_-d1g0da4.fasta

./group242/reference/d1euva_-d1gmya_.fasta

./group242/reference/d1euva_-d1iu4a_.fasta

./group242/reference/d1euva_-d1iwda_.fasta

./group242/reference/d1euva_-d1l9na4.fasta

./group242/reference/d1euva_-d1me4a_.fasta

./group242/reference/d1euva_-d1nbfa_.fasta

./group242/reference/d1euva_-d1qmya_.fasta

./group242/reference/d1euva_-d2cb5a_.fasta

./group242/reference/d1euva_-d3gcb__.fasta

./group242/reference/d1euva_-d7pcka_.fasta

./group242/reference/d1f13a4-d1iu4a_.fasta

./group242/reference/d1f13a4-d1nbfa_.fasta

./group242/reference/d1f13a4-d1qmya_.fasta

./group242/reference/d1fh0a_-d1iu4a_.fasta

./group242/reference/d1g0da4-d1iu4a_.fasta

./group242/reference/d1g0da4-d1qmya_.fasta

./group242/reference/d1g0da4-d2cb5a_.fasta

./group242/reference/d1gmya_-d1gx3a_.fasta

./group242/reference/d1gmya_-d1iu4a_.fasta

./group242/reference/d1gmya_-d1nbfa_.fasta

./group242/reference/d1gx3a_-d1iu4a_.fasta

./group242/reference/d1gx3a_-d1nbfa_.fasta

./group242/reference/d1gx3a_-d2cb5a_.fasta

./group242/reference/d1gx3a_-d7pcka_.fasta

./group242/reference/d1iu4a_-d1iwda_.fasta

./group242/reference/d1iu4a_-d1l9na4.fasta

./group242/reference/d1iu4a_-d1me4a_.fasta

./group242/reference/d1iu4a_-d1nbfa_.fasta

./group242/reference/d1iu4a_-d1qmya_.fasta

./group242/reference/d1iu4a_-d2cb5a_.fasta

./group242/reference/d1iu4a_-d3gcb__.fasta

./group242/reference/d1iwda_-d1qmya_.fasta

./group242/reference/d1me4a_-d1qmya_.fasta

./group242/reference/d1nbfa_-d3gcb__.fasta

./group242/reference/d1qmya_-d7pcka_.fasta

./group243/reference/d1a73a_-d1e7la2.fasta

./group243/reference/d1a73a_-d1fr2b_.fasta

./group246/reference/d1bf4a_-d1e0ba_.fasta

./group246/reference/d1bf4a_-d1g6za_.fasta

./group246/reference/d1e0ba_-d1g6za_.fasta

./group247/reference/d1bb8__-d1qk9a_.fasta

./group247/reference/d1d9na_-d1gcca_.fasta

./group247/reference/d1gcca_-d1qk9a_.fasta

./group248/reference/d1jj2l_-d1jj2r_.fasta

./group249/reference/d1guqa1-d1guqa2.fasta

./group249/reference/d1guqa1-d1kpf__.fasta

./group250/reference/d1a6f__-d1dar_3.fasta

./group250/reference/d1a6f__-d1ei1a1.fasta

./group250/reference/d1a6f__-d1fjgi_.fasta

./group250/reference/d1a6f__-d1h72c1.fasta

./group250/reference/d1a6f__-d1kija1.fasta

./group250/reference/d1a6f__-d1kkha1.fasta

./group250/reference/d1a6f__-d1mu5a2.fasta

./group250/reference/d1a6f__-d1p42a1.fasta

./group250/reference/d1a6f__-d1p42a2.fasta

./group250/reference/d1a6f__-d1ueka1.fasta

./group250/reference/d1b63a1-d1d6ta_.fasta

./group250/reference/d1b63a1-d1dar_3.fasta

./group250/reference/d1b63a1-d1ei1a1.fasta

./group250/reference/d1b63a1-d1h72c1.fasta

./group250/reference/d1b63a1-d1n0ua3.fasta

./group250/reference/d1b63a1-d1p42a1.fasta

./group250/reference/d1b63a1-d1p42a2.fasta

./group250/reference/d1b63a1-d1pkp_1.fasta

./group250/reference/d1d6ta_-d1dar_3.fasta

./group250/reference/d1d6ta_-d1ei1a1.fasta

./group250/reference/d1d6ta_-d1kija1.fasta

./group250/reference/d1d6ta_-d1kkha1.fasta

./group250/reference/d1d6ta_-d1mu5a2.fasta

./group250/reference/d1d6ta_-d1p42a1.fasta

./group250/reference/d1d6ta_-d1p42a2.fasta

./group250/reference/d1d6ta_-d1ueka1.fasta

./group250/reference/d1dar_3-d1ei1a1.fasta

./group250/reference/d1dar_3-d1fjgi_.fasta

./group250/reference/d1ei1a1-d1h72c1.fasta

./group250/reference/d1ei1a1-d1kkha1.fasta

./group250/reference/d1ei1a1-d1n0ua3.fasta

./group250/reference/d1ei1a1-d1p42a1.fasta

./group250/reference/d1ei1a1-d1p42a2.fasta

./group250/reference/d1ei1a1-d1pkp_1.fasta

./group250/reference/d1fjgi_-d1mu5a2.fasta

./group250/reference/d1h72c1-d1kija1.fasta

./group250/reference/d1h72c1-d1mu5a2.fasta

./group250/reference/d1kija1-d1n0ua3.fasta

./group250/reference/d1kija1-d1p42a1.fasta

./group250/reference/d1kija1-d1p42a2.fasta

./group250/reference/d1kija1-d1pkp_1.fasta

./group250/reference/d1kkha1-d1mu5a2.fasta

./group250/reference/d1kkha1-d1pkp_1.fasta

./group250/reference/d1mu5a2-d1n0ua3.fasta

./group250/reference/d1mu5a2-d1p42a2.fasta

./group250/reference/d1mu5a2-d1pkp_1.fasta

./group250/reference/d1mu5a2-d1ueka1.fasta

./group250/reference/d1n0ua3-d1pkp_1.fasta

./group250/reference/d1n0ua3-d1ueka1.fasta

./group250/reference/d1pkp_1-d1ueka1.fasta

./group251/reference/d1a5r__-d1c1yb_.fasta

./group251/reference/d1a5r__-d1h8ca_.fasta

./group251/reference/d1a5r__-d1i42a_.fasta

./group251/reference/d1a5r__-d1l7ya_.fasta

./group251/reference/d1a5r__-d1lfda_.fasta

./group251/reference/d1a5r__-d1rlf__.fasta

./group251/reference/d1c1yb_-d1euvb_.fasta

./group251/reference/d1c1yb_-d1gnua_.fasta

./group251/reference/d1c1yb_-d1i42a_.fasta

./group251/reference/d1c1yb_-d1lm8b_.fasta

./group251/reference/d1euvb_-d1gg3a3.fasta

./group251/reference/d1euvb_-d1h8ca_.fasta

./group251/reference/d1euvb_-d1i42a_.fasta

./group251/reference/d1euvb_-d1l7ya_.fasta

./group251/reference/d1euvb_-d1lfda_.fasta

./group251/reference/d1euvb_-d1rlf__.fasta

./group251/reference/d1gg3a3-d1gnua_.fasta

./group251/reference/d1gg3a3-d1h8ca_.fasta

./group251/reference/d1gg3a3-d1i42a_.fasta

./group251/reference/d1gg3a3-d1j8ca_.fasta

./group251/reference/d1gg3a3-d1m94a_.fasta

./group251/reference/d1gnua_-d1h4ra3.fasta

./group251/reference/d1gnua_-d1h8ca_.fasta

./group251/reference/d1gnua_-d1i42a_.fasta

./group251/reference/d1gnua_-d1lm8b_.fasta

./group251/reference/d1gnua_-d1m94a_.fasta

./group251/reference/d1h4ra3-d1l7ya_.fasta

./group251/reference/d1h4ra3-d1lfda_.fasta

./group251/reference/d1i42a_-d1lfda_.fasta

./group251/reference/d1i42a_-d1lm8b_.fasta

./group251/reference/d1j8ca_-d1lfda_.fasta

./group251/reference/d1l7ya_-d1m94a_.fasta

./group251/reference/d1l7ya_-d1rlf__.fasta

./group251/reference/d1lfda_-d1lm8b_.fasta

./group251/reference/d1lfda_-d1m94a_.fasta

./group251/reference/d1lm8b_-d1rlf__.fasta

./group252/reference/d1d4ba_-d1pqsa_.fasta

./group252/reference/d1f2ri_-d1pqsa_.fasta

./group254/reference/d1b9ra_-d1qlab2.fasta

./group254/reference/d1e9ma_-d1nekb2.fasta

./group254/reference/d1i7ha_-d1jroa2.fasta

./group254/reference/d1jq4a_-d1n62a2.fasta

./group254/reference/d1jq4a_-d1nekb2.fasta

./group254/reference/d1jq4a_-d1qlab2.fasta

./group254/reference/d1n62a2-d1qlab2.fasta

./group255/reference/d1bmlc3-d2sak__.fasta

./group257/reference/d1an9a2-d1d5ta2.fasta

./group257/reference/d1an9a2-d1f8ra2.fasta

./group257/reference/d1an9a2-d1gpea2.fasta

./group257/reference/d1an9a2-d1k0ia2.fasta

./group257/reference/d1an9a2-d1kdga2.fasta

./group257/reference/d1b5qa2-d1c0pa2.fasta

./group257/reference/d1b5qa2-d1d5ta2.fasta

./group257/reference/d1b5qa2-d1gosa2.fasta

./group257/reference/d1b5qa2-d1gpea2.fasta

./group257/reference/d1b5qa2-d1i8ta2.fasta

./group257/reference/d1b5qa2-d1ju2a2.fasta

./group257/reference/d1b5qa2-d1l9ea2.fasta

./group257/reference/d1b5qa2-d1mxta2.fasta

./group257/reference/d1c0pa2-d1f8ra2.fasta

./group257/reference/d1c0pa2-d1gosa2.fasta

./group257/reference/d1c0pa2-d1gpea2.fasta

./group257/reference/d1c0pa2-d1ju2a2.fasta

./group257/reference/d1c0pa2-d1mxta2.fasta

./group257/reference/d1d5ta2-d1f8ra2.fasta

./group257/reference/d1d5ta2-d1gpea2.fasta

./group257/reference/d1d5ta2-d1i8ta2.fasta

./group257/reference/d1d5ta2-d1k0ia2.fasta

./group257/reference/d1d5ta2-d1kdga2.fasta

./group257/reference/d1d5ta2-d1ng4a2.fasta

./group257/reference/d1f8ra2-d1gpea2.fasta

./group257/reference/d1f8ra2-d1ju2a2.fasta

./group257/reference/d1f8ra2-d1k0ia2.fasta

./group257/reference/d1f8ra2-d1kdga2.fasta

./group257/reference/d1f8ra2-d1l9ea2.fasta

./group257/reference/d1f8ra2-d1mxta2.fasta

./group257/reference/d1gosa2-d1i8ta2.fasta

./group257/reference/d1gosa2-d1ju2a2.fasta

./group257/reference/d1gosa2-d1k0ia2.fasta

./group257/reference/d1gosa2-d1kdga2.fasta

./group257/reference/d1gosa2-d1mxta2.fasta

./group257/reference/d1gosa2-d1ng4a2.fasta

./group257/reference/d1gpea2-d1i8ta2.fasta

./group257/reference/d1gpea2-d1k0ia2.fasta

./group257/reference/d1gpea2-d1l9ea2.fasta

./group257/reference/d1gpea2-d1ng4a2.fasta

./group257/reference/d1i8ta2-d1ju2a2.fasta

./group257/reference/d1i8ta2-d1k0ia2.fasta

./group257/reference/d1i8ta2-d1kdga2.fasta

./group257/reference/d1i8ta2-d1l9ea2.fasta

./group257/reference/d1i8ta2-d1mxta2.fasta

./group257/reference/d1i8ta2-d1ng4a2.fasta

./group257/reference/d1ju2a2-d1k0ia2.fasta

./group257/reference/d1ju2a2-d1l9ea2.fasta

./group257/reference/d1ju2a2-d1mxta2.fasta

./group257/reference/d1ju2a2-d1ng4a2.fasta

./group257/reference/d1k0ia2-d1kdga2.fasta

./group257/reference/d1k0ia2-d1ng4a2.fasta

./group257/reference/d1kdga2-d1ng4a2.fasta

./group257/reference/d1l9ea2-d1mxta2.fasta

./group258/reference/d1g96a_-d1mola_.fasta

./group258/reference/d1mola_-d1stfi_.fasta

./group259/reference/d1a2va3-d1ivwa2.fasta

./group259/reference/d1ivwa3-d1oaca2.fasta

./group260/reference/d1gy6a_-d1mwxa1.fasta

./group260/reference/d1gy6a_-d1nwwa_.fasta

./group260/reference/d1gy6a_-d1o7nb_.fasta

./group260/reference/d1gy6a_-d1oh0a_.fasta

./group260/reference/d1gy7a_-d1idpa_.fasta

./group260/reference/d1gy7a_-d1mwxa1.fasta

./group260/reference/d1gy7a_-d1o7nb_.fasta

./group260/reference/d1idpa_-d1ocva_.fasta

./group260/reference/d1idpa_-d1oh0a_.fasta

./group260/reference/d1jkga_-d1o7nb_.fasta

./group260/reference/d1m98a2-d1mwxa1.fasta

./group260/reference/d1m98a2-d1o7nb_.fasta

./group260/reference/d1mwxa1-d1nwwa_.fasta

./group260/reference/d1mwxa1-d1o7nb_.fasta

./group260/reference/d1mwxa1-d1ocva_.fasta

./group260/reference/d1mwxa1-d1oh0a_.fasta

./group260/reference/d1nwwa_-d1o7nb_.fasta

./group260/reference/d1o7nb_-d1ocva_.fasta

./group261/reference/d1c16a2-d1fnga2.fasta

./group261/reference/d1c16a2-d1hdmb2.fasta

./group261/reference/d1cd1a2-d1fnga2.fasta

./group261/reference/d1cd1a2-d1fngb2.fasta

./group261/reference/d1cd1a2-d1hdma2.fasta

./group261/reference/d1cd1a2-d1hdmb2.fasta

./group261/reference/d1cd1a2-d1iaka2.fasta

./group261/reference/d1cd1a2-d1jfma_.fasta

./group261/reference/d1de4a2-d1hdmb2.fasta

./group261/reference/d1fnga2-d1gzqa2.fasta

./group261/reference/d1fnga2-d1lqva_.fasta

./group261/reference/d1fngb2-d1gzqa2.fasta

./group261/reference/d1fngb2-d1hdma2.fasta

./group261/reference/d1fngb2-d1jfma_.fasta

./group261/reference/d1gzqa2-d1hdma2.fasta

./group261/reference/d1hdma2-d3frua2.fasta

./group261/reference/d1hdmb2-d1hyrc2.fasta

./group261/reference/d1hdmb2-d1k5na2.fasta

./group261/reference/d1iaka2-d1jfma_.fasta

./group261/reference/d1iaka2-d1lqva_.fasta

./group261/reference/d1iaka2-d3frua2.fasta

./group263/reference/d1hpwa_-d1oqva_.fasta

./group264/reference/d1bkf__-d1eq3a_.fasta

./group264/reference/d1bkf__-d1pina2.fasta

./group264/reference/d1eq3a_-d1hxva_.fasta

./group264/reference/d1eq3a_-d1jvwa_.fasta

./group264/reference/d1eq3a_-d1kt1a3.fasta

./group264/reference/d1eq3a_-d1l1pa_.fasta

./group264/reference/d1eq3a_-d1pbk__.fasta

./group264/reference/d1fd9a_-d1m5ya2.fasta

./group264/reference/d1hxva_-d1m5ya2.fasta

./group264/reference/d1hxva_-d1m5ya3.fasta

./group264/reference/d1hxva_-d1pina2.fasta

./group264/reference/d1j6ya_-d1kt1a3.fasta

./group264/reference/d1jnsa_-d1jvwa_.fasta

./group264/reference/d1jnsa_-d1pbk__.fasta

./group264/reference/d1jvwa_-d1pina2.fasta

./group264/reference/d1kt1a3-d1m5ya2.fasta

./group264/reference/d1kt1a3-d1m5ya3.fasta

./group264/reference/d1l1pa_-d1m5ya2.fasta

./group266/reference/d1cjxa1-d1mpya2.fasta

./group266/reference/d1ecsa_-d1kw3b2.fasta

./group266/reference/d1f1ua2-d1qtoa_.fasta

./group267/reference/d1c8ua1-d1iq6a_.fasta

./group267/reference/d1c8ua1-d1lo7a_.fasta

./group267/reference/d1c8ua1-d1mkaa_.fasta

./group267/reference/d1c8ua2-d1lo7a_.fasta

./group267/reference/d1lo7a_-d1mkaa_.fasta

./group271/reference/d1buoa_-d1fs1b2.fasta

./group271/reference/d1fs1b2-d1t1da_.fasta

./group271/reference/d1fs1b2-d3kvt__.fasta

./group271/reference/d1hv2a_-d1nn7a_.fasta

./group271/reference/d1hv2a_-d1t1da_.fasta

./group271/reference/d1hv2a_-d3kvt__.fasta

./group272/reference/d1efub2-d1efub4.fasta

./group276/reference/d1egaa2-d1k0ra2.fasta

./group279/reference/d1aye_2-d1itpa_.fasta

./group279/reference/d1aye_2-d1kn6a_.fasta

./group279/reference/d1aye_2-d1scjb_.fasta

./group279/reference/d1itpa_-d1jqga2.fasta

./group279/reference/d1itpa_-d1kn6a_.fasta

./group279/reference/d1itpa_-d1kwma2.fasta

./group279/reference/d1jqga2-d1kn6a_.fasta

./group280/reference/d1i1ga2-d1lq9a_.fasta

./group280/reference/d1i1ga2-d1nwja_.fasta

./group281/reference/d1nh8a2-d1nzaa_.fasta

./group281/reference/d1nzaa_-d2pii__.fasta

./group283/reference/d1b7fa2-d1koha2.fasta

./group283/reference/d1cvja1-d1koha2.fasta

./group283/reference/d1fxla1-d1koha2.fasta

./group283/reference/d1fxla2-d1koha2.fasta

./group283/reference/d1hd1a_-d1koha2.fasta

./group283/reference/d1iqta_-d1koha2.fasta

./group283/reference/d1koha2-d1l3ka2.fasta

./group283/reference/d1koha2-d1o0pa_.fasta

./group283/reference/d1koha2-d1owxa_.fasta

./group283/reference/d1koha2-d1qm9a2.fasta

./group283/reference/d1koha2-d1u2fa_.fasta

./group283/reference/d1koha2-d2msta_.fasta

./group283/reference/d1koha2-d2u1a__.fasta

./group283/reference/d1l3ka2-d1qm9a2.fasta

./group283/reference/d1owxa_-d1u2fa_.fasta

./group284/reference/d1dar_4-d1fnma4.fasta

./group286/reference/d1phza1-d1psda3.fasta

./group286/reference/d1psda3-d1tdj_2.fasta

./group288/reference/d1e6yb2-d1hbnc_.fasta

./group288/reference/d1hbnb2-d1hbnc_.fasta

./group289/reference/d1dj0a1-d1dj0a2.fasta

./group289/reference/d1dj0a1-d1k8wa1.fasta

./group289/reference/d1dj0a1-d1k8wa4.fasta

./group289/reference/d1dj0a2-d1k8wa1.fasta

./group289/reference/d1k8wa1-d1k8wa4.fasta

./group293/reference/d1dzfa2-d1i50f_.fasta

./group293/reference/d1eika_-d1i50f_.fasta

./group294/reference/d1dbfa_-d1jd1a_.fasta

./group294/reference/d1jd1a_-d1ufya_.fasta

./group294/reference/d1qd9a_-d1ufya_.fasta

./group297/reference/d1gd0a_-d1gyxa_.fasta

./group297/reference/d1gyxa_-d1hfoa_.fasta

./group297/reference/d1hfoa_-d1otga_.fasta

./group298/reference/d1b7go2-d1e5qa2.fasta

./group298/reference/d1b7go2-d1f06a2.fasta

./group298/reference/d1b7go2-d1h6da2.fasta

./group298/reference/d1b7go2-d1lc0a2.fasta

./group298/reference/d1b7go2-d1p1ja2.fasta

./group298/reference/d1cf2o2-d1dih_2.fasta

./group298/reference/d1cf2o2-d1e5qa2.fasta

./group298/reference/d1cf2o2-d1h6da2.fasta

./group298/reference/d1cf2o2-d1lc0a2.fasta

./group298/reference/d1cf2o2-d1nvmb2.fasta

./group298/reference/d1cf2o2-d1p1ja2.fasta

./group298/reference/d1dih_2-d1ebfa2.fasta

./group298/reference/d1dih_2-d1f06a2.fasta

./group298/reference/d1dih_2-d1h6da2.fasta

./group298/reference/d1dih_2-d1j5pa3.fasta

./group298/reference/d1dih_2-d1lc0a2.fasta

./group298/reference/d1dih_2-d1nvmb2.fasta

./group298/reference/d1e5qa2-d1ebfa2.fasta

./group298/reference/d1e5qa2-d1h6da2.fasta

./group298/reference/d1e5qa2-d1j5pa3.fasta

./group298/reference/d1e5qa2-d1lc0a2.fasta

./group298/reference/d1e5qa2-d1mb4a2.fasta

./group298/reference/d1e5qa2-d1nvmb2.fasta

./group298/reference/d1e5qa2-d1p1ja2.fasta

./group298/reference/d1ebfa2-d1f06a2.fasta

./group298/reference/d1ebfa2-d1h6da2.fasta

./group298/reference/d1ebfa2-d1mb4a2.fasta

./group298/reference/d1ebfa2-d1nvmb2.fasta

./group298/reference/d1f06a2-d1h6da2.fasta

./group298/reference/d1f06a2-d1lc0a2.fasta

./group298/reference/d1f06a2-d1nvmb2.fasta

./group298/reference/d1f06a2-d1p1ja2.fasta

./group298/reference/d1h6da2-d1j5pa3.fasta

./group298/reference/d1h6da2-d1mb4a2.fasta

./group298/reference/d1h6da2-d1nvmb2.fasta

./group298/reference/d1h6da2-d1p1ja2.fasta

./group298/reference/d1j5pa3-d1p1ja2.fasta

./group298/reference/d1lc0a2-d1mb4a2.fasta

./group298/reference/d1lc0a2-d1nvmb2.fasta

./group298/reference/d1lc0a2-d1p1ja2.fasta

./group298/reference/d1mb4a2-d1nvmb2.fasta

./group298/reference/d1mb4a2-d1p1ja2.fasta

./group298/reference/d1nvmb2-d1p1ja2.fasta

./group299/reference/d1d7ya3-d1fcda3.fasta

./group299/reference/d1dxla3-d1fcda3.fasta

./group299/reference/d1fcda3-d1feca3.fasta

./group299/reference/d1fcda3-d1h6va3.fasta

./group299/reference/d1fcda3-d1lvl_3.fasta

./group299/reference/d1fcda3-d1mo9a3.fasta

./group299/reference/d1fcda3-d1nhp_3.fasta

./group299/reference/d1fcda3-d1ojt_3.fasta

./group299/reference/d1fcda3-d3grs_3.fasta

./group299/reference/d1fcda3-d3lada3.fasta

./group300/reference/d1fo4a4-d1n62c1.fasta

./group301/reference/d1f08a_-d1m55a_.fasta

./group301/reference/d1f08a_-d1tbd__.fasta

./group301/reference/d1l2ma_-d1tbd__.fasta

./group301/reference/d1m55a_-d1tbd__.fasta

./group303/reference/d1ast__-d1bqba_.fasta

./group303/reference/d1ast__-d1ezm__.fasta

./group303/reference/d1ast__-d1keia_.fasta

./group303/reference/d1atla_-d1dmta_.fasta

./group303/reference/d1atla_-d1i1ip_.fasta

./group303/reference/d1atla_-d1j36a_.fasta

./group303/reference/d1bkca_-d1i1ip_.fasta

./group303/reference/d1bkca_-d1j36a_.fasta

./group303/reference/d1bqba_-d1j7na2.fasta

./group303/reference/d1bqba_-d1k9xa_.fasta

./group303/reference/d1bqba_-d1kufa_.fasta

./group303/reference/d1buda_-d1dmta_.fasta

./group303/reference/d1buda_-d1eb6a_.fasta

./group303/reference/d1buda_-d1hs6a3.fasta

./group303/reference/d1buda_-d1k9xa_.fasta

./group303/reference/d1c7ka_-d1j36a_.fasta

./group303/reference/d1c7ka_-d1j7na2.fasta

./group303/reference/d1c7ka_-d1keia_.fasta

./group303/reference/d1dmta_-d1j36a_.fasta

./group303/reference/d1dmta_-d1k7ia2.fasta

./group303/reference/d1dmta_-d1k9xa_.fasta

./group303/reference/d1eb6a_-d1hs6a3.fasta

./group303/reference/d1eb6a_-d1j36a_.fasta

./group303/reference/d1ezm__-d1hs6a3.fasta

./group303/reference/d1ezm__-d1j36a_.fasta

./group303/reference/d1ezm__-d1j7na2.fasta

./group303/reference/d1ezm__-d1k7ia2.fasta

./group303/reference/d1ezm__-d1k9xa_.fasta

./group303/reference/d1ezm__-d1kapp2.fasta

./group303/reference/d1g12a_-d1j7na1.fasta

./group303/reference/d1g12a_-d1k9xa_.fasta

./group303/reference/d1gkda_-d1hs6a3.fasta

./group303/reference/d1hs6a3-d1i1ip_.fasta

./group303/reference/d1hs6a3-d1j36a_.fasta

./group303/reference/d1hs6a3-d1j7na1.fasta

./group303/reference/d1hs6a3-d1jk3a_.fasta

./group303/reference/d1hs6a3-d1k7ia2.fasta

./group303/reference/d1hs6a3-d1k9xa_.fasta

./group303/reference/d1hs6a3-d1keia_.fasta

./group303/reference/d1i1ip_-d1j7na1.fasta

./group303/reference/d1i1ip_-d1j7na2.fasta

./group303/reference/d1i1ip_-d1jk3a_.fasta

./group303/reference/d1i1ip_-d1kufa_.fasta

./group303/reference/d1j36a_-d1k7ia2.fasta

./group303/reference/d1j36a_-d1kufa_.fasta

./group303/reference/d1j7na1-d1kapp2.fasta

./group303/reference/d1j7na1-d1keia_.fasta

./group303/reference/d1j7na2-d1kapp2.fasta

./group303/reference/d1jk3a_-d1keia_.fasta

./group308/reference/d1a8ra_-d1uox_1.fasta

./group308/reference/d1a8ra_-d1uox_2.fasta

./group308/reference/d1b66a_-d1uox_2.fasta

./group308/reference/d1b9la_-d1uox_2.fasta

./group308/reference/d1dhn__-d1uox_2.fasta

./group308/reference/d1uox_1-d1uox_2.fasta

./group309/reference/d12asa_-d1nj1a3.fasta

./group310/reference/d1cjwa_-d1fy7a_.fasta

./group310/reference/d1fy7a_-d1lrza2.fasta

./group310/reference/d1fy7a_-d1m4ia_.fasta

./group310/reference/d1fy7a_-d1mk4a_.fasta

./group310/reference/d1iica2-d1lrza2.fasta

./group310/reference/d1iica2-d1ufha_.fasta

./group310/reference/d1iyka1-d1iyka2.fasta

./group310/reference/d1iyka1-d1m4ia_.fasta

./group310/reference/d1iyka1-d1mk4a_.fasta

./group310/reference/d1iyka1-d1ufha_.fasta

./group310/reference/d1iyka2-d1lrza2.fasta

./group310/reference/d1iyka2-d1m4ia_.fasta

./group310/reference/d1iyka2-d1ufha_.fasta

./group310/reference/d1lrza2-d1m4ia_.fasta

./group310/reference/d1lrza2-d1qsma_.fasta

./group310/reference/d1m4ia_-d1n71a_.fasta

./group311/reference/d1ak7__-d1d0na4.fasta

./group311/reference/d1ak7__-d1d0na6.fasta

./group311/reference/d1ak7__-d1d4xg_.fasta

./group311/reference/d1cfya_-d1d0na3.fasta

./group311/reference/d1cfya_-d1d4xg_.fasta

./group311/reference/d1d0na3-d1hqz1_.fasta

./group311/reference/d1d0na5-d1hqz1_.fasta

./group311/reference/d1d0na5-d1m4ja_.fasta

./group311/reference/d1d0na6-d1f7sa_.fasta

./group311/reference/d1d0na6-d1hqz1_.fasta

./group311/reference/d1d0na6-d1m4ja_.fasta

./group311/reference/d1d4xg_-d1m4ja_.fasta

./group311/reference/d1f7sa_-d1jhwa3.fasta

./group313/reference/d1mc0a2-d1mkma2.fasta

./group321/reference/d1g61a_-d1h70a_.fasta

./group321/reference/d1g61a_-d1jdw__.fasta

./group321/reference/d1g62a_-d1h70a_.fasta

./group321/reference/d1g62a_-d1jdw__.fasta

./group323/reference/d1f52a2-d1m15a2.fasta

./group323/reference/d1f52a2-d1qh4a2.fasta

./group324/reference/d1aisa1-d1mpga2.fasta

./group324/reference/d1aisa2-d1ko9a2.fasta

./group324/reference/d1ko9a2-d1ytba2.fasta

./group324/reference/d1mpga2-d1ytba1.fasta

./group324/reference/d1mpga2-d1ytba2.fasta

./group326/reference/d1fm4a_-d1jssa_.fasta

./group326/reference/d1fm4a_-d1ln1a_.fasta

./group326/reference/d1icxa_-d1jssa_.fasta

./group326/reference/d1icxa_-d1ln1a_.fasta

./group326/reference/d1jssa_-d1kcma_.fasta

./group326/reference/d1kcma_-d1ln1a_.fasta

./group328/reference/d1b77a1-d1b77a2.fasta

./group328/reference/d1b77a1-d2pola1.fasta

./group328/reference/d1b77a2-d1dmla1.fasta

./group328/reference/d1b77a2-d1iz5a2.fasta

./group328/reference/d1b77a2-d1plq_1.fasta

./group328/reference/d1b77a2-d2pola2.fasta

./group328/reference/d1dmla1-d1iz5a2.fasta

./group328/reference/d1dmla1-d1plq_2.fasta

./group328/reference/d1dmla1-d2pola2.fasta

./group328/reference/d1iz5a2-d2pola2.fasta

./group328/reference/d1plq_1-d2pola1.fasta

./group328/reference/d1plq_2-d2pola2.fasta

./group328/reference/d2pola1-d2pola3.fasta

./group328/reference/d2pola2-d2pola3.fasta

./group332/reference/d1a9xa5-d1gsa_2.fasta

./group332/reference/d1a9xa5-d1kbla3.fasta

./group332/reference/d1a9xa6-d1gsa_2.fasta

./group332/reference/d1e4ea2-d1kbla3.fasta

./group332/reference/d1eucb2-d1i7na2.fasta

./group332/reference/d1gsa_2-d1kbla3.fasta

./group334/reference/d1diqa2-d1fo4a6.fasta

./group334/reference/d1diqa2-d1n62c2.fasta

./group335/reference/d1qr0a1-d1qr0a2.fasta

./group336/reference/d1ct9a2-d1pmaa_.fasta

./group336/reference/d1ct9a2-d1rypa_.fasta

./group336/reference/d1ct9a2-d1rypb_.fasta

./group336/reference/d1ct9a2-d1rype_.fasta

./group336/reference/d1ct9a2-d1rypg_.fasta

./group336/reference/d1ct9a2-d1rypj_.fasta

./group336/reference/d1ct9a2-d1rypl_.fasta

./group336/reference/d1ecfa2-d1j2pa_.fasta

./group336/reference/d1ecfa2-d1pmaa_.fasta

./group336/reference/d1ecfa2-d1ryp2_.fasta

./group336/reference/d1ecfa2-d1rypa_.fasta

./group336/reference/d1ecfa2-d1rypb_.fasta

./group336/reference/d1ecfa2-d1ryph_.fasta

./group336/reference/d1ecfa2-d1rypk_.fasta

./group336/reference/d1gdoa_-d1iru1_.fasta

./group336/reference/d1gph12-d1iru1_.fasta

./group336/reference/d1gph12-d1ryp2_.fasta

./group336/reference/d1gph12-d1ryph_.fasta

./group336/reference/d1j2pa_-d1ofda3.fasta

./group336/reference/d1j2qh_-d1ofda3.fasta

./group336/reference/d1ofda3-d1pmaa_.fasta

./group336/reference/d1ofda3-d1rypa_.fasta

./group336/reference/d1ofda3-d1rypb_.fasta

./group336/reference/d1ofda3-d1rypc_.fasta

./group336/reference/d1ofda3-d1rypd_.fasta

./group336/reference/d1ofda3-d1rype_.fasta

./group336/reference/d1ofda3-d1rypf_.fasta

./group336/reference/d1ofda3-d1rypg_.fasta

./group336/reference/d1ofda3-d1ryph_.fasta

./group336/reference/d1ofda3-d1rypi_.fasta

./group336/reference/d1ofda3-d1rypk_.fasta

./group338/reference/d1nnwa_-d4kbpa2.fasta

./group341/reference/d1a26_2-d1giqa1.fasta

./group341/reference/d1a26_2-d1gxya_.fasta

./group341/reference/d1a26_2-d1qs1a2.fasta

./group341/reference/d1gxya_-d1qs1a1.fasta

./group343/reference/d1b08a1-d1cwva5.fasta

./group343/reference/d1b08a1-d1kg0c_.fasta

./group343/reference/d1b08a1-d1koe__.fasta

./group343/reference/d1b08a1-d1li1a1.fasta

./group343/reference/d1b08a1-d1li1a2.fasta

./group343/reference/d1b6e__-d1li1a1.fasta

./group343/reference/d1b6e__-d1li1a2.fasta

./group343/reference/d1b6e__-d1tsg__.fasta

./group343/reference/d1byfa_-d1koe__.fasta

./group343/reference/d1byfa_-d1li1a1.fasta

./group343/reference/d1byfa_-d1li1a2.fasta

./group343/reference/d1byfa_-d1prea1.fasta

./group343/reference/d1byfa_-d1prtb2.fasta

./group343/reference/d1cwva5-d1j34b_.fasta

./group343/reference/d1cwva5-d1jwib_.fasta

./group343/reference/d1cwva5-d1k9ja_.fasta

./group343/reference/d1cwva5-d1koe__.fasta

./group343/reference/d1cwva5-d1li1a1.fasta

./group343/reference/d1cwva5-d1li1a2.fasta

./group343/reference/d1cwva5-d1prtb2.fasta

./group343/reference/d1dv8a_-d1koe__.fasta

./group343/reference/d1dv8a_-d1li1a1.fasta

./group343/reference/d1dv8a_-d1li1a2.fasta

./group343/reference/d1dv8a_-d1prea1.fasta

./group343/reference/d1e87a_-d1li1a1.fasta

./group343/reference/d1e87a_-d1li1a2.fasta

./group343/reference/d1e87a_-d1prea1.fasta

./group343/reference/d1e87a_-d1prtb2.fasta

./group343/reference/d1e87a_-d1tsg__.fasta

./group343/reference/d1f00i3-d1li1a1.fasta

./group343/reference/d1f00i3-d1li1a2.fasta

./group343/reference/d1f00i3-d1prea1.fasta

./group343/reference/d1f00i3-d1prtb2.fasta

./group343/reference/d1g1ta1-d1koe__.fasta

./group343/reference/d1g1ta1-d1li1a1.fasta

./group343/reference/d1g1ta1-d1li1a2.fasta

./group343/reference/d1h8ua_-d1li1a1.fasta

./group343/reference/d1h8ua_-d1li1a2.fasta

./group343/reference/d1h8ua_-d1prea1.fasta

./group343/reference/d1h8ua_-d1prtb2.fasta

./group343/reference/d1hq8a_-d1li1a1.fasta

./group343/reference/d1hq8a_-d1li1a2.fasta

./group343/reference/d1hq8a_-d1prea1.fasta

./group343/reference/d1j34a_-d1li1a1.fasta

./group343/reference/d1j34a_-d1li1a2.fasta

./group343/reference/d1j34a_-d1prtb2.fasta

./group343/reference/d1j34b_-d1li1a1.fasta

./group343/reference/d1j34b_-d1li1a2.fasta

./group343/reference/d1j34b_-d1prtb2.fasta

./group343/reference/d1jwib_-d1li1a1.fasta

./group343/reference/d1jwib_-d1li1a2.fasta

./group343/reference/d1jwib_-d1prea1.fasta

./group343/reference/d1jwib_-d1prtb2.fasta

./group343/reference/d1jzna_-d1li1a1.fasta

./group343/reference/d1jzna_-d1li1a2.fasta

./group343/reference/d1jzna_-d1prea1.fasta

./group343/reference/d1k9ja_-d1koe__.fasta

./group343/reference/d1k9ja_-d1li1a1.fasta

./group343/reference/d1k9ja_-d1li1a2.fasta

./group343/reference/d1k9ja_-d1tsg__.fasta

./group343/reference/d1kg0c_-d1li1a1.fasta

./group343/reference/d1kg0c_-d1li1a2.fasta

./group343/reference/d1kg0c_-d1prea1.fasta

./group343/reference/d1koe__-d1li1a1.fasta

./group343/reference/d1koe__-d1li1a2.fasta

./group343/reference/d1koe__-d1tsg__.fasta

./group343/reference/d1li1a2-d1prea1.fasta

./group343/reference/d1li1a2-d1prtb2.fasta

./group343/reference/d1li1a2-d1qdda_.fasta

./group343/reference/d1li1a2-d1tn3__.fasta

./group343/reference/d1li1a2-d1tsg__.fasta

./group343/reference/d1prea1-d1qdda_.fasta

./group343/reference/d1prea1-d1tsg__.fasta

./group343/reference/d1qdda_-d1tsg__.fasta

./group350/reference/d1hhsa_-d1jiha_.fasta

./group350/reference/d1hhsa_-d1l3sa2.fasta

./group350/reference/d1jiha_-d1mml__.fasta

./group350/reference/d1jiha_-d1vrta2.fasta

./group350/reference/d1l3sa2-d1tgoa2.fasta

./group350/reference/d1l3sa2-d1vrta2.fasta

./group353/reference/d1jjya_-d1oaoa_.fasta

./group353/reference/d1jjya_-d1oaoc_.fasta

./group353/reference/d1oaoa_-d1oaoc_.fasta

./group356/reference/d1ddba_-d1k3ka_.fasta

./group357/reference/d1jo5a_-d1lgha_.fasta

./group357/reference/d1jo5a_-d1lghb_.fasta

./group357/reference/d1jo5a_-d1nkzb_.fasta

./group357/reference/d1lgha_-d1lghb_.fasta

./group357/reference/d1lgha_-d1nkzb_.fasta

./group357/reference/d1lghb_-d1nkzb_.fasta

./group358/reference/d1mm4a_-d1p4ta_.fasta

./group359/reference/d1a0tp_-d1by5a_.fasta

./group359/reference/d1a0tp_-d1kmoa_.fasta

./group359/reference/d1a0tp_-d3prn__.fasta

./group359/reference/d1by5a_-d1hxxa_.fasta

./group359/reference/d1by5a_-d2mpra_.fasta

./group359/reference/d1by5a_-d2por__.fasta

./group359/reference/d1hxxa_-d1kmoa_.fasta

./group359/reference/d1kmoa_-d2mpra_.fasta

./group359/reference/d1kmoa_-d3prn__.fasta

./group359/reference/d2mpra_-d3prn__.fasta

./group361/reference/d1agg__-d1emxa_.fasta

./group361/reference/d1axh__-d1lupa_.fasta

./group361/reference/d1c6wa_-d1emxa_.fasta

./group361/reference/d1c6wa_-d1qk7a_.fasta

./group361/reference/d1cixa_-d1g9pa_.fasta

./group361/reference/d1dl0a_-d1lupa_.fasta

./group361/reference/d1i25a_-d1vtx__.fasta

./group361/reference/d1i26a_-d1ju8a_.fasta

./group361/reference/d1ju8a_-d1koza_.fasta

./group361/reference/d1lupa_-d1vtx__.fasta

./group362/reference/d1aho__-d1sis__.fasta

./group362/reference/d1bmr__-d1sis__.fasta

./group362/reference/d1nrb__-d1sis__.fasta

./group362/reference/d1sis__-d1tsk__.fasta

./group363/reference/d1h59b_-d1igra3.fasta

./group363/reference/d1h59b_-d1ivoa4.fasta

./group363/reference/d1h59b_-d1m6ba4.fasta

./group363/reference/d1igra3-d1ivoa4.fasta

./group363/reference/d1ivoa4-d1m6ba3.fasta

./group363/reference/d1ivoa4-d1m6ba4.fasta

./group363/reference/d1ivoa4-d1n8yc4.fasta

./group363/reference/d1ivoa4-d1nqla3.fasta

./group363/reference/d1ivoa4-d1nqla4.fasta

./group365/reference/d1b9wa1-d1jl9a_.fasta

./group365/reference/d1b9wa1-d1tpg_1.fasta

./group365/reference/d1b9wa1-d1xdtr_.fasta

./group365/reference/d1b9wa1-d3tgf__.fasta

./group365/reference/d1dx5i1-d1klo_2.fasta

./group365/reference/d1fjsl_-d1ioxa_.fasta

./group365/reference/d1hz8a1-d1l3ya_.fasta

./group365/reference/d1hz8a1-d1m1xb5.fasta

./group365/reference/d1hz8a2-d1l3ya_.fasta

./group365/reference/d1hz8a2-d1tpg_1.fasta

./group365/reference/d1ijqa2-d1m1xb5.fasta

./group365/reference/d1klo_2-d1urk_1.fasta

./group365/reference/d1klo_3-d1lmja1.fasta

./group365/reference/d1klo_3-d1m1xb5.fasta

./group365/reference/d1klo_3-d1xdtr_.fasta

./group365/reference/d1m1xb5-d1ob1c1.fasta

./group365/reference/d1ob1c1-d1xdtr_.fasta

./group366/reference/d1bx7__-d1dec__.fasta

./group366/reference/d1bx7__-d1e0fi_.fasta

./group366/reference/d1dec__-d1e0fi_.fasta

./group366/reference/d1e0fi_-d1skz_1.fasta

./group369/reference/d1ewsa_-d1sh1__.fasta

./group369/reference/d1ewsa_-d2bds__.fasta

./group374/reference/d1ce3a_-d1iw4a_.fasta

./group374/reference/d1ce3a_-d1lr7a2.fasta

./group374/reference/d1ce3a_-d1nuba3.fasta

./group374/reference/d1ce3a_-d1sgpi_.fasta

./group374/reference/d1ce3a_-d4sgbi_.fasta

./group374/reference/d1iw4a_-d4sgbi_.fasta

./group374/reference/d1lr7a2-d4sgbi_.fasta

./group374/reference/d1nuba3-d4sgbi_.fasta

./group374/reference/d1sgpi_-d4sgbi_.fasta

./group374/reference/d1tbrr1-d4sgbi_.fasta

./group374/reference/d1tbrr2-d4sgbi_.fasta

./group374/reference/d1tgsi_-d4sgbi_.fasta

./group376/reference/d1aoca_-d1fl7b_.fasta

./group376/reference/d1aoca_-d1hcna_.fasta

./group376/reference/d1aoca_-d1lxia_.fasta

./group376/reference/d1bnda_-d1fltv_.fasta

./group376/reference/d1bnda_-d1jpya_.fasta

./group376/reference/d1fl7b_-d1jpya_.fasta

./group376/reference/d1jpya_-d2tgi__.fasta

./group377/reference/d1gkna2-d1quba5.fasta

./group377/reference/d1h03p2-d1quba5.fasta

./group378/reference/d1mpza_-d1n4ya_.fasta

./group378/reference/d1mpza_-d2ech__.fasta

./group380/reference/d1d4va1-d1d4va2.fasta

./group380/reference/d1d4va2-d1oqdk_.fasta

./group380/reference/d1d4va2-d1oqek_.fasta

./group380/reference/d1d4va3-d1oqdk_.fasta

./group380/reference/d1d4va3-d1oqek_.fasta

./group380/reference/d1exta1-d1oqdk_.fasta

./group380/reference/d1exta1-d1oqek_.fasta

./group380/reference/d1exta2-d1oqdk_.fasta

./group380/reference/d1exta2-d1oqek_.fasta

./group383/reference/d1fu9a_-d1tf6a4.fasta

./group383/reference/d1fv5a_-d1ncs__.fasta

./group383/reference/d1fv5a_-d1njqa_.fasta

./group383/reference/d1fv5a_-d1tf3a3.fasta

./group383/reference/d1fv5a_-d1tf6a4.fasta

./group383/reference/d1fv5a_-d1zfd__.fasta

./group383/reference/d1fv5a_-d2drpa1.fasta

./group383/reference/d1fv5a_-d2glia4.fasta

./group383/reference/d1njqa_-d1zfd__.fasta

./group383/reference/d1tf3a1-d1tf3a3.fasta

./group384/reference/d1hwtc1-d2alca_.fasta

./group384/reference/d1pyia1-d2alca_.fasta

./group385/reference/d1a7i_2-d1j2oa2.fasta

./group385/reference/d1a7i_2-d1l1za3.fasta

./group385/reference/d1a7i_2-d1lv3a_.fasta

./group385/reference/d1d4ua2-d1gnf__.fasta

./group385/reference/d1d4ua2-d1j2oa2.fasta

./group385/reference/d1d4ua2-d1k3xa3.fasta

./group385/reference/d1d4ua2-d3gata_.fasta

./group385/reference/d1d4ua2-d7gata_.fasta

./group385/reference/d1dsza_-d1fjgn_.fasta

./group385/reference/d1dsza_-d1gnf__.fasta

./group385/reference/d1dsza_-d1j2oa2.fasta

./group385/reference/d1dsza_-d1jj2t_.fasta

./group385/reference/d1dsza_-d1lv3a_.fasta

./group385/reference/d1fjgn_-d1g47a2.fasta

./group385/reference/d1fjgn_-d1k3xa3.fasta

./group385/reference/d1fjgn_-d1kb2a_.fasta

./group385/reference/d1fjgn_-d1l1za3.fasta

./group385/reference/d1fjgn_-d1lata_.fasta

./group385/reference/d1fjgn_-d1lo1a_.fasta

./group385/reference/d1fjgn_-d1lv3a_.fasta

./group385/reference/d1fjgn_-d1nypa1.fasta

./group385/reference/d1fjgn_-d2nllb_.fasta

./group385/reference/d1g47a1-d1j2oa2.fasta

./group385/reference/d1g47a1-d1kb2a_.fasta

./group385/reference/d1g47a2-d1j2oa2.fasta

./group385/reference/d1g47a2-d1lv3a_.fasta

./group385/reference/d1gnf__-d1j2oa2.fasta

./group385/reference/d1gnf__-d1kb2a_.fasta

./group385/reference/d1gnf__-d1lata_.fasta

./group385/reference/d1gnf__-d1lv3a_.fasta

./group385/reference/d1gnf__-d1nypa1.fasta

./group385/reference/d1gnf__-d2nllb_.fasta

./group385/reference/d1ibia2-d1j2oa2.fasta

./group385/reference/d1ibia2-d1lv3a_.fasta

./group385/reference/d1iml_2-d1j2oa1.fasta

./group385/reference/d1iml_2-d1j2oa2.fasta

./group385/reference/d1iml_2-d1k3xa3.fasta

./group385/reference/d1j2oa1-d1j2oa2.fasta

./group385/reference/d1j2oa1-d1l1za3.fasta

./group385/reference/d1j2oa1-d1lv3a_.fasta

./group385/reference/d1j2oa2-d1jj2t_.fasta

./group385/reference/d1j2oa2-d1k3xa3.fasta

./group385/reference/d1j2oa2-d1kb2a_.fasta

./group385/reference/d1j2oa2-d1l1za3.fasta

./group385/reference/d1j2oa2-d1lata_.fasta

./group385/reference/d1j2oa2-d1lo1a_.fasta

./group385/reference/d1j2oa2-d1m3va2.fasta

./group385/reference/d1j2oa2-d1nypa1.fasta

./group385/reference/d1j2oa2-d1nypa2.fasta

./group385/reference/d1j2oa2-d2nllb_.fasta

./group385/reference/d1j2oa2-d7gata_.fasta

./group385/reference/d1jj2t_-d1k3xa3.fasta

./group385/reference/d1jj2t_-d1lv3a_.fasta

./group385/reference/d1jj2t_-d1m3va2.fasta

./group385/reference/d1k3xa3-d1lata_.fasta

./group385/reference/d1k3xa3-d1lv3a_.fasta

./group385/reference/d1k3xa3-d1m3va2.fasta

./group385/reference/d1k3xa3-d1nypa1.fasta

./group385/reference/d1k3xa3-d2nllb_.fasta

./group385/reference/d1kb2a_-d1lv3a_.fasta

./group385/reference/d1l1za3-d1lv3a_.fasta

./group385/reference/d1l1za3-d1m3va1.fasta

./group385/reference/d1lata_-d1lv3a_.fasta

./group385/reference/d1lo1a_-d1lv3a_.fasta

./group385/reference/d1lo1a_-d1nypa1.fasta

./group385/reference/d1lv3a_-d1m3va1.fasta

./group385/reference/d1lv3a_-d1m3va2.fasta

./group385/reference/d1lv3a_-d1nypa2.fasta

./group385/reference/d1lv3a_-d2nllb_.fasta

./group385/reference/d1lv3a_-d7gata_.fasta

./group385/reference/d1m3va1-d1m3va2.fasta

./group385/reference/d1m3va1-d1nypa2.fasta

./group385/reference/d1m3va1-d3gata_.fasta

./group385/reference/d1nypa1-d2nllb_.fasta

./group388/reference/d1d0qa_-d1qyp__.fasta

./group388/reference/d1d0qa_-d1yua_1.fasta

./group388/reference/d1dl6a_-d1i50i2.fasta

./group388/reference/d1dl6a_-d1qyp__.fasta

./group388/reference/d1dl6a_-d1yua_2.fasta

./group388/reference/d1i50i1-d1yua_1.fasta

./group388/reference/d1i50i1-d1yua_2.fasta

./group388/reference/d1i50i2-d1yua_2.fasta

./group388/reference/d1pft__-d1yua_1.fasta

./group388/reference/d1pft__-d1yua_2.fasta

./group388/reference/d1qyp__-d1yua_1.fasta

./group388/reference/d1qyp__-d1yua_2.fasta

./group388/reference/d1tfi__-d1yua_2.fasta

./group388/reference/d1yua_1-d1yua_2.fasta

./group389/reference/d1ocrf_-d1rb9__.fasta

./group390/reference/d1jj22_-d1jj2y_.fasta

./group390/reference/d1jj22_-d1jj2z_.fasta

./group390/reference/d1jj2y_-d1nvha_.fasta

./group391/reference/d1bor__-d1chc__.fasta

./group391/reference/d1bor__-d1e4ua_.fasta

./group391/reference/d1bor__-d1jm7b_.fasta

./group391/reference/d1bor__-d1ldjb_.fasta

./group391/reference/d1bor__-d1n87a_.fasta

./group391/reference/d1chc__-d1jm7a_.fasta

./group391/reference/d1chc__-d1jm7b_.fasta

./group391/reference/d1e4ua_-d1iyma_.fasta

./group391/reference/d1e4ua_-d1jm7a_.fasta

./group391/reference/d1e4ua_-d1jm7b_.fasta

./group391/reference/d1e4ua_-d1rmd_2.fasta

./group391/reference/d1fbva4-d1jm7b_.fasta

./group391/reference/d1iyma_-d1jm7a_.fasta

./group391/reference/d1iyma_-d1jm7b_.fasta

./group392/reference/d1dmc__-d1fmya_.fasta

./group392/reference/d1dmc__-d1jjda_.fasta

./group392/reference/d1dmc__-d4mt2__.fasta

./group392/reference/d1fmya_-d4mt2__.fasta

./group392/reference/d1jjda_-d1m0ga_.fasta

./group392/reference/d1jjda_-d4mt2__.fasta

./group392/reference/d1m0ga_-d4mt2__.fasta

./group392/reference/d1qjka_-d4mt2__.fasta

./group393/reference/d1faq__-d1kbea_.fasta

./group394/reference/d1dvpa2-d1fp0a1.fasta

./group394/reference/d1fp0a1-d1joca1.fasta

./group394/reference/d1fp0a1-d1mm2a_.fasta

./group394/reference/d1fp0a1-d1vfya_.fasta

./group396/reference/d1cqxa2-d1i8da2.fasta

./group396/reference/d1ddga1-d1kzla1.fasta

./group396/reference/d1ddga1-d1kzla2.fasta

./group396/reference/d1f20a1-d1i8da2.fasta

./group396/reference/d1i7pa1-d1i8da2.fasta

./group396/reference/d1i8da2-d1jb9a1.fasta

./group396/reference/d1i8da2-d1krha1.fasta

./group396/reference/d1i8da2-d2pia_1.fasta

./group396/reference/d1ja1a1-d1kzla1.fasta

./group396/reference/d1kzla1-d2pia_1.fasta

./group397/reference/d1ezva1-d1ezvb2.fasta

./group397/reference/d1ezva1-d1hr6a2.fasta

./group397/reference/d1ezva2-d1hr6a1.fasta

./group397/reference/d1ezva2-d1l0la1.fasta

./group397/reference/d1ezvb1-d1l0lb2.fasta

./group397/reference/d1ezvb2-d1hr6a1.fasta

./group397/reference/d1ezvb2-d1hr6b1.fasta

./group397/reference/d1ezvb2-d1l0la1.fasta

./group397/reference/d1ezvb2-d1l0lb1.fasta

./group397/reference/d1hr6a1-d1hr6a2.fasta

./group397/reference/d1hr6a1-d1hr6b2.fasta

./group397/reference/d1hr6a2-d1hr6b1.fasta

./group397/reference/d1hr6a2-d1l0lb1.fasta

./group397/reference/d1hr6b1-d1hr6b2.fasta

./group397/reference/d1hr6b1-d1l0la2.fasta

./group397/reference/d1hr6b1-d1l0lb2.fasta

./group397/reference/d1l0la1-d1l0la2.fasta

./group397/reference/d1l0lb1-d1l0lb2.fasta

./group402/reference/d1gw5m2-d1h3qa_.fasta

./group402/reference/d1gw5s_-d1h3qa_.fasta

./group402/reference/d1gw5s_-d1h8ma_.fasta

./group402/reference/d1h3qa_-d1h8ma_.fasta

./group407/reference/d1cb8a3-d1jova_.fasta

./group407/reference/d1cb8a3-d1jz8a4.fasta

./group407/reference/d1cb8a3-d1lf6a2.fasta

./group407/reference/d1cb8a3-d1nsza_.fasta

./group407/reference/d1hn0a4-d1jz8a4.fasta

./group407/reference/d1hn0a4-d1lf6a2.fasta

./group407/reference/d1j0ma3-d1jova_.fasta

./group407/reference/d1j0ma3-d1jz8a4.fasta

./group407/reference/d1j0ma3-d1lf6a2.fasta

./group407/reference/d1jova_-d1jz8a4.fasta

./group407/reference/d1jova_-d1lf6a2.fasta

./group407/reference/d1jz8a4-d1lf6a2.fasta

./group407/reference/d1jz8a4-d1n7oa3.fasta

./group407/reference/d1lf6a2-d1n7oa3.fasta

./group407/reference/d1lf6a2-d1nsza_.fasta

./group408/reference/d1a02n1-d1bf2_1.fasta

./group408/reference/d1a02n1-d1clc_2.fasta

./group408/reference/d1a02n1-d1ea9c1.fasta

./group408/reference/d1a02n1-d1edqa1.fasta

./group408/reference/d1a02n1-d1eut_1.fasta

./group408/reference/d1a02n1-d1g0da1.fasta

./group408/reference/d1a02n1-d1g4ma1.fasta

./group408/reference/d1a02n1-d1j0ha1.fasta

./group408/reference/d1a02n1-d1ji1a1.fasta

./group408/reference/d1a02n1-d1ji2a1.fasta

./group408/reference/d1a02n1-d1jjua4.fasta

./group408/reference/d1a02n1-d1jmxa4.fasta

./group408/reference/d1a02n1-d1kmta_.fasta

./group408/reference/d1a02n1-d1ktja_.fasta

./group408/reference/d1a02n1-d1qba_1.fasta

./group408/reference/d1a02n1-d1qfha2.fasta

./group408/reference/d1a02n1-d1soxa1.fasta

./group408/reference/d1bf2_1-d1edqa1.fasta

./group408/reference/d1bf2_1-d1g4ma1.fasta

./group408/reference/d1bf2_1-d1imhc1.fasta

./group408/reference/d1bf2_1-d1jjua4.fasta

./group408/reference/d1bf2_1-d1kmta_.fasta

./group408/reference/d1bf2_1-d1ktja_.fasta

./group408/reference/d1bf2_1-d1nepa_.fasta

./group408/reference/d1bf2_1-d1qba_1.fasta

./group408/reference/d1clc_2-d1ea9c1.fasta

./group408/reference/d1clc_2-d1eut_1.fasta

./group408/reference/d1clc_2-d1g4ma1.fasta

./group408/reference/d1clc_2-d1imhc1.fasta

./group408/reference/d1clc_2-d1j0ha1.fasta

./group408/reference/d1clc_2-d1ji1a1.fasta

./group408/reference/d1clc_2-d1ji2a1.fasta

./group408/reference/d1clc_2-d1jjua4.fasta

./group408/reference/d1clc_2-d1jmxa4.fasta

./group408/reference/d1clc_2-d1ktja_.fasta

./group408/reference/d1clc_2-d1m7xa1.fasta

./group408/reference/d1clc_2-d1my7a_.fasta

./group408/reference/d1clc_2-d1nepa_.fasta

./group408/reference/d1clc_2-d1soxa1.fasta

./group408/reference/d1ea9c1-d1edqa1.fasta

./group408/reference/d1ea9c1-d1g0da1.fasta

./group408/reference/d1ea9c1-d1g4ma1.fasta

./group408/reference/d1ea9c1-d1imhc1.fasta

./group408/reference/d1ea9c1-d1jjua4.fasta

./group408/reference/d1ea9c1-d1jmxa4.fasta

./group408/reference/d1ea9c1-d1k3ia1.fasta

./group408/reference/d1ea9c1-d1kmta_.fasta

./group408/reference/d1ea9c1-d1ktja_.fasta

./group408/reference/d1ea9c1-d1l9na1.fasta

./group408/reference/d1ea9c1-d1nepa_.fasta

./group408/reference/d1ea9c1-d1qba_1.fasta

./group408/reference/d1ea9c1-d1qfha1.fasta

./group408/reference/d1ea9c1-d1qfha2.fasta

./group408/reference/d1ea9c1-d1soxa1.fasta

./group408/reference/d1edqa1-d1ji1a1.fasta

./group408/reference/d1edqa1-d1kmta_.fasta

./group408/reference/d1edqa1-d1ktja_.fasta

./group408/reference/d1edqa1-d1m7xa1.fasta

./group408/reference/d1edqa1-d1my7a_.fasta

./group408/reference/d1edqa1-d1soxa1.fasta

./group408/reference/d1eut_1-d1g4ma1.fasta

./group408/reference/d1eut_1-d1ji2a1.fasta

./group408/reference/d1eut_1-d1k3ia1.fasta

./group408/reference/d1eut_1-d1ktja_.fasta

./group408/reference/d1eut_1-d1m7xa1.fasta

./group408/reference/d1eut_1-d1my7a_.fasta

./group408/reference/d1eut_1-d1qba_1.fasta

./group408/reference/d1eut_1-d1qfha2.fasta

./group408/reference/d1g0da1-d1g4ma1.fasta

./group408/reference/d1g0da1-d1imhc1.fasta

./group408/reference/d1g0da1-d1j0ha1.fasta

./group408/reference/d1g0da1-d1ji1a1.fasta

./group408/reference/d1g0da1-d1ji2a1.fasta

./group408/reference/d1g0da1-d1jjua4.fasta

./group408/reference/d1g0da1-d1jmxa4.fasta

./group408/reference/d1g0da1-d1qba_1.fasta

./group408/reference/d1g0da1-d1qfha2.fasta

./group408/reference/d1g0da1-d1soxa1.fasta

./group408/reference/d1g4ma1-d1imhc1.fasta

./group408/reference/d1g4ma1-d1j0ha1.fasta

./group408/reference/d1g4ma1-d1ji1a1.fasta

./group408/reference/d1g4ma1-d1jjua4.fasta

./group408/reference/d1g4ma1-d1jmxa4.fasta

./group408/reference/d1g4ma1-d1k3ia1.fasta

./group408/reference/d1g4ma1-d1kmta_.fasta

./group408/reference/d1g4ma1-d1ktja_.fasta

./group408/reference/d1g4ma1-d1l9na1.fasta

./group408/reference/d1g4ma1-d1m7xa1.fasta

./group408/reference/d1g4ma1-d1nepa_.fasta

./group408/reference/d1g4ma1-d1qba_1.fasta

./group408/reference/d1g4ma1-d1qfha2.fasta

./group408/reference/d1g4ma1-d1soxa1.fasta

./group408/reference/d1imhc1-d1ji1a1.fasta

./group408/reference/d1imhc1-d1jjua4.fasta

./group408/reference/d1imhc1-d1jmxa4.fasta

./group408/reference/d1imhc1-d1l9na1.fasta

./group408/reference/d1imhc1-d1m7xa1.fasta

./group408/reference/d1imhc1-d1qfha1.fasta

./group408/reference/d1imhc1-d1qfha2.fasta

./group408/reference/d1imhc1-d1soxa1.fasta

./group408/reference/d1j0ha1-d1jjua4.fasta

./group408/reference/d1j0ha1-d1jmxa4.fasta

./group408/reference/d1j0ha1-d1kmta_.fasta

./group408/reference/d1j0ha1-d1ktja_.fasta

./group408/reference/d1j0ha1-d1my7a_.fasta

./group408/reference/d1j0ha1-d1nepa_.fasta

./group408/reference/d1j0ha1-d1qfha1.fasta

./group408/reference/d1j0ha1-d1qfha2.fasta

./group408/reference/d1ji1a1-d1jjua4.fasta

./group408/reference/d1ji1a1-d1jmxa4.fasta

./group408/reference/d1ji1a1-d1k3ia1.fasta

./group408/reference/d1ji1a1-d1kmta_.fasta

./group408/reference/d1ji1a1-d1my7a_.fasta

./group408/reference/d1ji1a1-d1nepa_.fasta

./group408/reference/d1ji1a1-d1qba_1.fasta

./group408/reference/d1ji1a1-d1qfha1.fasta

./group408/reference/d1ji1a1-d1qfha2.fasta

./group408/reference/d1ji1a1-d1soxa1.fasta

./group408/reference/d1ji2a1-d1jjua4.fasta

./group408/reference/d1ji2a1-d1k3ia1.fasta

./group408/reference/d1ji2a1-d1kmta_.fasta

./group408/reference/d1ji2a1-d1nepa_.fasta

./group408/reference/d1ji2a1-d1qfha1.fasta

./group408/reference/d1jjua4-d1m7xa1.fasta

./group408/reference/d1jjua4-d1my7a_.fasta

./group408/reference/d1jjua4-d1nepa_.fasta

./group408/reference/d1jjua4-d1qba_1.fasta

./group408/reference/d1jjua4-d1qfha2.fasta

./group408/reference/d1jjua4-d1soxa1.fasta

./group408/reference/d1jmxa4-d1k3ia1.fasta

./group408/reference/d1jmxa4-d1l9na1.fasta

./group408/reference/d1jmxa4-d1my7a_.fasta

./group408/reference/d1jmxa4-d1nepa_.fasta

./group408/reference/d1jmxa4-d1qba_1.fasta

./group408/reference/d1jmxa4-d1soxa1.fasta

./group408/reference/d1k3ia1-d1ktja_.fasta

./group408/reference/d1k3ia1-d1l9na1.fasta

./group408/reference/d1k3ia1-d1soxa1.fasta

./group408/reference/d1kmta_-d1l9na1.fasta

./group408/reference/d1kmta_-d1m7xa1.fasta

./group408/reference/d1kmta_-d1my7a_.fasta

./group408/reference/d1kmta_-d1qfha1.fasta

./group408/reference/d1kmta_-d1qfha2.fasta

./group408/reference/d1kmta_-d1soxa1.fasta

./group408/reference/d1ktja_-d1l9na1.fasta

./group408/reference/d1ktja_-d1m7xa1.fasta

./group408/reference/d1ktja_-d1my7a_.fasta

./group408/reference/d1ktja_-d1soxa1.fasta

./group408/reference/d1l9na1-d1qba_1.fasta

./group408/reference/d1l9na1-d1qfha1.fasta

./group408/reference/d1l9na1-d1soxa1.fasta

./group408/reference/d1m7xa1-d1my7a_.fasta

./group408/reference/d1m7xa1-d1qba_1.fasta

./group408/reference/d1m7xa1-d1qfha1.fasta

./group408/reference/d1m7xa1-d1qfha2.fasta

./group408/reference/d1my7a_-d1qfha1.fasta

./group408/reference/d1my7a_-d1soxa1.fasta

./group408/reference/d1nepa_-d1qfha1.fasta

./group408/reference/d1nepa_-d1qfha2.fasta

./group408/reference/d1nepa_-d1soxa1.fasta

./group408/reference/d1qba_1-d1qfha2.fasta

./group408/reference/d1qfha1-d1soxa1.fasta

./group408/reference/d1qfha2-d1soxa1.fasta

./group410/reference/d1e12a_-d1h2sb_.fasta

./group410/reference/d1h2sa_-d1h2sb_.fasta

./group411/reference/d1lnqa2-d1orsc_.fasta

./group412/reference/d1kf6c_-d1qlac_.fasta

./group412/reference/d1nekd_-d1qlac_.fasta

./group413/reference/d1fftc_-d1m56c_.fasta

./group413/reference/d1fftc_-d1ocrc_.fasta

./group414/reference/d1ehkb2-d1fftb2.fasta

./group414/reference/d1ehkb2-d1m56b2.fasta

./group414/reference/d1ehkb2-d1ocrb2.fasta

./group419/reference/d1aym1_-d1b35a_.fasta

./group419/reference/d1aym1_-d1bmv11.fasta

./group419/reference/d1aym1_-d1ddla_.fasta

./group419/reference/d1aym1_-d1ng0a_.fasta

./group419/reference/d1aym1_-d1ny711.fasta

./group419/reference/d1aym1_-d1ny722.fasta

./group419/reference/d1aym1_-d2bbva_.fasta

./group419/reference/d1aym3_-d1ddla_.fasta

./group419/reference/d1aym3_-d1f2na_.fasta

./group419/reference/d1aym3_-d1ng0a_.fasta

./group419/reference/d1aym3_-d1ny711.fasta

./group419/reference/d1aym3_-d2bbva_.fasta

./group419/reference/d1b35a_-d1d4m1_.fasta

./group419/reference/d1b35a_-d1f2na_.fasta

./group419/reference/d1b35a_-d1k5ma_.fasta

./group419/reference/d1b35a_-d1ny722.fasta

./group419/reference/d1b35a_-d1opoa_.fasta

./group419/reference/d1b35a_-d1pvc1_.fasta

./group419/reference/d1b35a_-d1qqp3_.fasta

./group419/reference/d1b35a_-d1smva_.fasta

./group419/reference/d1b35a_-d2bbva_.fasta

./group419/reference/d1b35b_-d1c8na_.fasta

./group419/reference/d1b35b_-d1ddla_.fasta

./group419/reference/d1b35b_-d1f2na_.fasta

./group419/reference/d1b35b_-d1ng0a_.fasta

./group419/reference/d1b35b_-d1ny711.fasta

./group419/reference/d1b35b_-d1ny722.fasta

./group419/reference/d1b35b_-d1smva_.fasta

./group419/reference/d1b35b_-d2bbva_.fasta

./group419/reference/d1bev1_-d1bmv11.fasta

./group419/reference/d1bev1_-d1ddla_.fasta

./group419/reference/d1bev1_-d1f2na_.fasta

./group419/reference/d1bev1_-d1ng0a_.fasta

./group419/reference/d1bev1_-d1ny711.fasta

./group419/reference/d1bev1_-d2bbva_.fasta

./group419/reference/d1bev3_-d1ddla_.fasta

./group419/reference/d1bev3_-d1ny722.fasta

./group419/reference/d1bev3_-d2bbva_.fasta

./group419/reference/d1bmv11-d1c8na_.fasta

./group419/reference/d1bmv11-d1d4m1_.fasta

./group419/reference/d1bmv11-d1ddla_.fasta

./group419/reference/d1bmv11-d1k5ma_.fasta

./group419/reference/d1bmv11-d1ng0a_.fasta

./group419/reference/d1bmv11-d1opoa_.fasta

./group419/reference/d1bmv11-d1pvc1_.fasta

./group419/reference/d1bmv11-d1pvc3_.fasta

./group419/reference/d1bmv11-d1smva_.fasta

./group419/reference/d1bmv11-d2bbva_.fasta

./group419/reference/d1c8na_-d1k5ma_.fasta

./group419/reference/d1c8na_-d1ny711.fasta

./group419/reference/d1c8na_-d1qqp3_.fasta

./group419/reference/d1c8na_-d2bbva_.fasta

./group419/reference/d1d4m1_-d1ddla_.fasta

./group419/reference/d1d4m1_-d1f2na_.fasta

./group419/reference/d1d4m1_-d1ihma_.fasta

./group419/reference/d1d4m1_-d1ng0a_.fasta

./group419/reference/d1d4m1_-d1ny711.fasta

./group419/reference/d1d4m1_-d1ny722.fasta

./group419/reference/d1d4m1_-d1smva_.fasta

./group419/reference/d1ddla_-d1k5ma_.fasta

./group419/reference/d1ddla_-d1ng0a_.fasta

./group419/reference/d1ddla_-d1ny711.fasta

./group419/reference/d1ddla_-d1ny722.fasta

./group419/reference/d1ddla_-d1pvc1_.fasta

./group419/reference/d1ddla_-d1pvc3_.fasta

./group419/reference/d1ddla_-d1qqp3_.fasta

./group419/reference/d1ddla_-d2bbva_.fasta

./group419/reference/d1ddla_-d2mev1_.fasta

./group419/reference/d1ddla_-d2mev3_.fasta

./group419/reference/d1f2na_-d1k5ma_.fasta

./group419/reference/d1f2na_-d1ny722.fasta

./group419/reference/d1f2na_-d1pvc1_.fasta

./group419/reference/d1f2na_-d1tmf1_.fasta

./group419/reference/d1k5ma_-d1ng0a_.fasta

./group419/reference/d1k5ma_-d1ny711.fasta

./group419/reference/d1k5ma_-d1ny722.fasta

./group419/reference/d1k5ma_-d2bbva_.fasta

./group419/reference/d1ng0a_-d1ny711.fasta

./group419/reference/d1ng0a_-d1ny722.fasta

./group419/reference/d1ng0a_-d1pvc1_.fasta

./group419/reference/d1ng0a_-d1tmf1_.fasta

./group419/reference/d1ny711-d1ny722.fasta

./group419/reference/d1ny711-d1opoa_.fasta

./group419/reference/d1ny711-d1pvc1_.fasta

./group419/reference/d1ny711-d1pvc3_.fasta

./group419/reference/d1ny711-d1smva_.fasta

./group419/reference/d1ny711-d1tmf1_.fasta

./group419/reference/d1ny711-d2bbva_.fasta

./group419/reference/d1ny711-d2mev3_.fasta

./group419/reference/d1ny722-d1opoa_.fasta

./group419/reference/d1ny722-d1pvc1_.fasta

./group419/reference/d1ny722-d1smva_.fasta

./group419/reference/d1ny722-d2bbva_.fasta

./group419/reference/d1ny722-d2mev3_.fasta

./group419/reference/d1opoa_-d2bbva_.fasta

./group419/reference/d1opoa_-d2mev1_.fasta

./group419/reference/d1pvc1_-d2bbva_.fasta

./group419/reference/d1pvc3_-d1smva_.fasta

./group419/reference/d1qqp3_-d1smva_.fasta

./group419/reference/d1qqp3_-d2bbva_.fasta

./group419/reference/d1qqp3_-d2mev1_.fasta

./group419/reference/d1smva_-d1tmf1_.fasta

./group419/reference/d1tmf1_-d2bbva_.fasta

./group419/reference/d2bbva_-d2mev1_.fasta

./group420/reference/d1c8da_-d1dnv__.fasta

./group420/reference/d1c8da_-d1gff2_.fasta

./group420/reference/d1c8da_-d1m06g_.fasta

./group420/reference/d1c8da_-d2bpa1_.fasta

./group420/reference/d1c8da_-d2bpa2_.fasta

./group420/reference/d1dnv__-d1gff2_.fasta

./group420/reference/d1dnv__-d1m06g_.fasta

./group420/reference/d1dnv__-d2bpa1_.fasta

./group420/reference/d1dnv__-d2bpa2_.fasta

./group420/reference/d1gff2_-d2bpa1_.fasta

./group420/reference/d1lp3a_-d1m06g_.fasta

./group420/reference/d1lp3a_-d2bpa1_.fasta

./group420/reference/d1lp3a_-d2bpa2_.fasta

./group420/reference/d1m06g_-d2bpa1_.fasta

./group420/reference/d2bpa1_-d2bpa2_.fasta

./group422/reference/d1a34a_-d1stma_.fasta

./group422/reference/d1a34a_-d2stv__.fasta

./group422/reference/d1stma_-d2stv__.fasta

./group424/reference/d1g8fa1-d1iq8a3.fasta

./group425/reference/d1h3la_-d1iw7f3.fasta
