# Supplementary material for: PR2ALIGN: a stand-alone software program and a web-server for protein sequence alignment using weighted biochemical properties of amino acids
Source: BMC Res Notes. 2015 May 7;8:187. doi: 10.1186/s13104-015-1152-6 (PMC4477417; doi:10.1186/s13104-015-1152-6)
Supplement: Additional file 7: — All SABmark SUP sequence pairs for 10-20% sequence identity range. [file 13104_2015_1152_MOESM7_ESM.docx]

All SABmark SUP sequence pairs for 10-20% sequence identity range.

./group1/reference/d1a6m__-d1ash__.fasta

./group1/reference/d1a6m__-d1cg5b_.fasta

./group1/reference/d1a6m__-d1cqxa1.fasta

./group1/reference/d1a6m__-d1d8ua_.fasta

./group1/reference/d1a6m__-d1ew6a_.fasta

./group1/reference/d1a6m__-d1gvha1.fasta

./group1/reference/d1a6m__-d1h97a_.fasta

./group1/reference/d1a6m__-d1hlb__.fasta

./group1/reference/d1a6m__-d1it2a_.fasta

./group1/reference/d1a6m__-d1itha_.fasta

./group1/reference/d1a6m__-d2gdm__.fasta

./group1/reference/d1alla_-d1cqxa1.fasta

./group1/reference/d1alla_-d1ew6a_.fasta

./group1/reference/d1alla_-d1h97a_.fasta

./group1/reference/d1alla_-d1irdb_.fasta

./group1/reference/d1alla_-d1it2a_.fasta

./group1/reference/d1alla_-d1mba__.fasta

./group1/reference/d1alla_-d3sdha_.fasta

./group1/reference/d1allb_-d1cg5a_.fasta

./group1/reference/d1allb_-d1cg5b_.fasta

./group1/reference/d1allb_-d1d8ua_.fasta

./group1/reference/d1allb_-d1ew6a_.fasta

./group1/reference/d1allb_-d1gcva_.fasta

./group1/reference/d1allb_-d1hlb__.fasta

./group1/reference/d1allb_-d1irda_.fasta

./group1/reference/d1allb_-d1itha_.fasta

./group1/reference/d1allb_-d1mba__.fasta

./group1/reference/d1allb_-d2gdm__.fasta

./group1/reference/d1ash__-d1cg5a_.fasta

./group1/reference/d1ash__-d1cg5b_.fasta

./group1/reference/d1ash__-d1cqxa1.fasta

./group1/reference/d1ash__-d1d8ua_.fasta

./group1/reference/d1ash__-d1ew6a_.fasta

./group1/reference/d1ash__-d1gcva_.fasta

./group1/reference/d1ash__-d1gvha1.fasta

./group1/reference/d1ash__-d1h97a_.fasta

./group1/reference/d1ash__-d1hlb__.fasta

./group1/reference/d1ash__-d1irda_.fasta

./group1/reference/d1ash__-d1irdb_.fasta

./group1/reference/d1ash__-d1it2a_.fasta

./group1/reference/d1ash__-d1itha_.fasta

./group1/reference/d1ash__-d1jl7a_.fasta

./group1/reference/d1ash__-d1la6a_.fasta

./group1/reference/d1ash__-d1mba__.fasta

./group1/reference/d1ash__-d2gdm__.fasta

./group1/reference/d1ash__-d2lhb__.fasta

./group1/reference/d1ash__-d3sdha_.fasta

./group1/reference/d1b8da_-d1cg5a_.fasta

./group1/reference/d1b8da_-d1ew6a_.fasta

./group1/reference/d1b8da_-d1gcva_.fasta

./group1/reference/d1b8da_-d1h97a_.fasta

./group1/reference/d1b8da_-d1hlb__.fasta

./group1/reference/d1b8da_-d1la6a_.fasta

./group1/reference/d1b8da_-d1mba__.fasta

./group1/reference/d1cg5a_-d1cqxa1.fasta

./group1/reference/d1cg5a_-d1d8ua_.fasta

./group1/reference/d1cg5a_-d1ew6a_.fasta

./group1/reference/d1cg5a_-d1gvha1.fasta

./group1/reference/d1cg5a_-d1h97a_.fasta

./group1/reference/d1cg5a_-d1itha_.fasta

./group1/reference/d1cg5a_-d1jboa_.fasta

./group1/reference/d1cg5a_-d1jl7a_.fasta

./group1/reference/d1cg5a_-d1mba__.fasta

./group1/reference/d1cg5a_-d2gdm__.fasta

./group1/reference/d1cg5b_-d1cqxa1.fasta

./group1/reference/d1cg5b_-d1d8ua_.fasta

./group1/reference/d1cg5b_-d1ew6a_.fasta

./group1/reference/d1cg5b_-d1gvha1.fasta

./group1/reference/d1cg5b_-d1h97a_.fasta

./group1/reference/d1cg5b_-d1itha_.fasta

./group1/reference/d1cg5b_-d1jboa_.fasta

./group1/reference/d1cg5b_-d1jl7a_.fasta

./group1/reference/d1cg5b_-d1mba__.fasta

./group1/reference/d1cg5b_-d3sdha_.fasta

./group1/reference/d1cqxa1-d1h97a_.fasta

./group1/reference/d1cqxa1-d1irda_.fasta

./group1/reference/d1cqxa1-d1irdb_.fasta

./group1/reference/d1cqxa1-d1it2a_.fasta

./group1/reference/d1cqxa1-d1itha_.fasta

./group1/reference/d1cqxa1-d1jboa_.fasta

./group1/reference/d1cqxa1-d1mba__.fasta

./group1/reference/d1cqxa1-d2lhb__.fasta

./group1/reference/d1cqxa1-d3sdha_.fasta

./group1/reference/d1d8ua_-d1ew6a_.fasta

./group1/reference/d1d8ua_-d1gcva_.fasta

./group1/reference/d1d8ua_-d1gvha1.fasta

./group1/reference/d1d8ua_-d1h97a_.fasta

./group1/reference/d1d8ua_-d1irda_.fasta

./group1/reference/d1d8ua_-d1irdb_.fasta

./group1/reference/d1d8ua_-d1it2a_.fasta

./group1/reference/d1d8ua_-d1jl7a_.fasta

./group1/reference/d1d8ua_-d1la6a_.fasta

./group1/reference/d1d8ua_-d2lhb__.fasta

./group1/reference/d1d8ua_-d3sdha_.fasta

./group1/reference/d1ew6a_-d1gcva_.fasta

./group1/reference/d1ew6a_-d1gvha1.fasta

./group1/reference/d1ew6a_-d1hlb__.fasta

./group1/reference/d1ew6a_-d1irda_.fasta

./group1/reference/d1ew6a_-d1irdb_.fasta

./group1/reference/d1ew6a_-d1it2a_.fasta

./group1/reference/d1ew6a_-d1jl7a_.fasta

./group1/reference/d1ew6a_-d1la6a_.fasta

./group1/reference/d1ew6a_-d1mba__.fasta

./group1/reference/d1ew6a_-d2gdm__.fasta

./group1/reference/d1ew6a_-d2lhb__.fasta

./group1/reference/d1ew6a_-d3sdha_.fasta

./group1/reference/d1gcva_-d1gvha1.fasta

./group1/reference/d1gcva_-d1h97a_.fasta

./group1/reference/d1gcva_-d1itha_.fasta

./group1/reference/d1gcva_-d1jboa_.fasta

./group1/reference/d1gcva_-d1jl7a_.fasta

./group1/reference/d1gcva_-d1mba__.fasta

./group1/reference/d1gcva_-d2gdm__.fasta

./group1/reference/d1gvha1-d1h97a_.fasta

./group1/reference/d1gvha1-d1hlb__.fasta

./group1/reference/d1gvha1-d1irda_.fasta

./group1/reference/d1gvha1-d1irdb_.fasta

./group1/reference/d1gvha1-d1it2a_.fasta

./group1/reference/d1gvha1-d1itha_.fasta

./group1/reference/d1gvha1-d1jl7a_.fasta

./group1/reference/d1gvha1-d1la6a_.fasta

./group1/reference/d1gvha1-d1mba__.fasta

./group1/reference/d1gvha1-d2lhb__.fasta

./group1/reference/d1gvha1-d3sdha_.fasta

./group1/reference/d1h97a_-d1hlb__.fasta

./group1/reference/d1h97a_-d1irda_.fasta

./group1/reference/d1h97a_-d1irdb_.fasta

./group1/reference/d1h97a_-d1it2a_.fasta

./group1/reference/d1h97a_-d1itha_.fasta

./group1/reference/d1h97a_-d1jl7a_.fasta

./group1/reference/d1h97a_-d1la6a_.fasta

./group1/reference/d1h97a_-d1mba__.fasta

./group1/reference/d1h97a_-d2gdm__.fasta

./group1/reference/d1h97a_-d2lhb__.fasta

./group1/reference/d1h97a_-d3sdha_.fasta

./group1/reference/d1hlb__-d1it2a_.fasta

./group1/reference/d1hlb__-d1jl7a_.fasta

./group1/reference/d1hlb__-d1la6a_.fasta

./group1/reference/d1hlb__-d1mba__.fasta

./group1/reference/d1irda_-d1itha_.fasta

./group1/reference/d1irda_-d1mba__.fasta

./group1/reference/d1irda_-d2gdm__.fasta

./group1/reference/d1irda_-d3sdha_.fasta

./group1/reference/d1irdb_-d1itha_.fasta

./group1/reference/d1irdb_-d1jboa_.fasta

./group1/reference/d1irdb_-d1mba__.fasta

./group1/reference/d1irdb_-d2gdm__.fasta

./group1/reference/d1irdb_-d3sdha_.fasta

./group1/reference/d1it2a_-d1itha_.fasta

./group1/reference/d1it2a_-d1jl7a_.fasta

./group1/reference/d1it2a_-d2gdm__.fasta

./group1/reference/d1it2a_-d3sdha_.fasta

./group1/reference/d1itha_-d1la6a_.fasta

./group1/reference/d1itha_-d1mba__.fasta

./group1/reference/d1itha_-d2gdm__.fasta

./group1/reference/d1itha_-d2lhb__.fasta

./group1/reference/d1itha_-d3sdha_.fasta

./group1/reference/d1jboa_-d1la6a_.fasta

./group1/reference/d1jboa_-d1mba__.fasta

./group1/reference/d1jboa_-d2lhb__.fasta

./group1/reference/d1jl7a_-d1la6a_.fasta

./group1/reference/d1jl7a_-d2gdm__.fasta

./group1/reference/d1jl7a_-d3sdha_.fasta

./group1/reference/d1la6a_-d1mba__.fasta

./group1/reference/d1la6a_-d2gdm__.fasta

./group1/reference/d1la6a_-d3sdha_.fasta

./group1/reference/d2gdm__-d2lhb__.fasta

./group1/reference/d2gdm__-d3sdha_.fasta

./group1/reference/d2lhb__-d3sdha_.fasta

./group2/reference/d1gtea1-d1kf6b1.fasta

./group2/reference/d1gtea1-d1nekb1.fasta

./group3/reference/d1fafa_-d1fpoa1.fasta

./group3/reference/d1fafa_-d1gh6a_.fasta

./group3/reference/d1fafa_-d1hdj__.fasta

./group4/reference/d1eiya1-d1ivsa1.fasta

./group4/reference/d1eiya1-d1lrza1.fasta

./group4/reference/d1lrza1-d1seta1.fasta

./group6/reference/d1c52__-d1dw0a_.fasta

./group6/reference/d1c52__-d1eb7a1.fasta

./group6/reference/d1c52__-d1f1ca_.fasta

./group6/reference/d1c52__-d1gu2a_.fasta

./group6/reference/d1c52__-d1h32a1.fasta

./group6/reference/d1c52__-d1iqca1.fasta

./group6/reference/d1c52__-d1qksa1.fasta

./group6/reference/d1c52__-d1ycc__.fasta

./group6/reference/d1co6a_-d1dw0a_.fasta

./group6/reference/d1co6a_-d1e29a_.fasta

./group6/reference/d1co6a_-d1eb7a1.fasta

./group6/reference/d1co6a_-d1ezvd1.fasta

./group6/reference/d1co6a_-d1fcdc2.fasta

./group6/reference/d1co6a_-d1gu2a_.fasta

./group6/reference/d1co6a_-d1iqca1.fasta

./group6/reference/d1co6a_-d1kb0a1.fasta

./group6/reference/d1co6a_-d1kv9a1.fasta

./group6/reference/d1co6a_-d1nira1.fasta

./group6/reference/d1co6a_-d1qksa1.fasta

./group6/reference/d1cot__-d1eb7a1.fasta

./group6/reference/d1cot__-d1gu2a_.fasta

./group6/reference/d1cot__-d1h32a1.fasta

./group6/reference/d1cot__-d1iqca2.fasta

./group6/reference/d1cot__-d1kb0a1.fasta

./group6/reference/d1cot__-d1kv9a1.fasta

./group6/reference/d1cot__-d1mg2d_.fasta

./group6/reference/d1cot__-d1nira1.fasta

./group6/reference/d1cot__-d1qksa1.fasta

./group6/reference/d1cxc__-d1dw0a_.fasta

./group6/reference/d1cxc__-d1e29a_.fasta

./group6/reference/d1cxc__-d1etpa2.fasta

./group6/reference/d1cxc__-d1ezvd1.fasta

./group6/reference/d1cxc__-d1gu2a_.fasta

./group6/reference/d1cxc__-d1iqca1.fasta

./group6/reference/d1cxc__-d1mg2d_.fasta

./group6/reference/d1cxc__-d1nira1.fasta

./group6/reference/d1cxc__-d1qksa1.fasta

./group6/reference/d1dw0a_-d1eb7a1.fasta

./group6/reference/d1dw0a_-d1etpa2.fasta

./group6/reference/d1dw0a_-d1ezvd1.fasta

./group6/reference/d1dw0a_-d1iqca1.fasta

./group6/reference/d1dw0a_-d1kv9a1.fasta

./group6/reference/d1dw0a_-d1mg2d_.fasta

./group6/reference/d1dw0a_-d1nira1.fasta

./group6/reference/d1dw0a_-d1qksa1.fasta

./group6/reference/d1dw0a_-d1ycc__.fasta

./group6/reference/d1e29a_-d1eb7a1.fasta

./group6/reference/d1e29a_-d1etpa2.fasta

./group6/reference/d1e29a_-d1ezvd1.fasta

./group6/reference/d1e29a_-d1fcdc2.fasta

./group6/reference/d1e29a_-d1iqca1.fasta

./group6/reference/d1e29a_-d1iqca2.fasta

./group6/reference/d1e29a_-d1kb0a1.fasta

./group6/reference/d1e29a_-d1kv9a1.fasta

./group6/reference/d1e29a_-d1mg2d_.fasta

./group6/reference/d1e29a_-d1nira1.fasta

./group6/reference/d1eb7a1-d1etpa2.fasta

./group6/reference/d1eb7a1-d1ezvd1.fasta

./group6/reference/d1eb7a1-d1f1ca_.fasta

./group6/reference/d1eb7a1-d1fcdc2.fasta

./group6/reference/d1eb7a1-d1gu2a_.fasta

./group6/reference/d1eb7a1-d1h32a1.fasta

./group6/reference/d1eb7a1-d1kb0a1.fasta

./group6/reference/d1eb7a1-d1mg2d_.fasta

./group6/reference/d1eb7a1-d1nira1.fasta

./group6/reference/d1eb7a1-d1ycc__.fasta

./group6/reference/d1etpa2-d1ezvd1.fasta

./group6/reference/d1etpa2-d1h32a1.fasta

./group6/reference/d1etpa2-d1hroa_.fasta

./group6/reference/d1etpa2-d1iqca1.fasta

./group6/reference/d1etpa2-d1iqca2.fasta

./group6/reference/d1etpa2-d1kv9a1.fasta

./group6/reference/d1ezvd1-d1fcdc2.fasta

./group6/reference/d1ezvd1-d1iqca1.fasta

./group6/reference/d1ezvd1-d1kb0a1.fasta

./group6/reference/d1ezvd1-d3c2c__.fasta

./group6/reference/d1f1ca_-d1fcdc2.fasta

./group6/reference/d1f1ca_-d1iqca1.fasta

./group6/reference/d1f1ca_-d1iqca2.fasta

./group6/reference/d1f1ca_-d1kb0a1.fasta

./group6/reference/d1f1ca_-d1kv9a1.fasta

./group6/reference/d1fcdc2-d1h32a1.fasta

./group6/reference/d1fcdc2-d1hroa_.fasta

./group6/reference/d1fcdc2-d1iqca1.fasta

./group6/reference/d1fcdc2-d1iqca2.fasta

./group6/reference/d1fcdc2-d1kv9a1.fasta

./group6/reference/d1fcdc2-d1mg2d_.fasta

./group6/reference/d1fcdc2-d1nira1.fasta

./group6/reference/d1fcdc2-d1qksa1.fasta

./group6/reference/d1fcdc2-d1qn2a_.fasta

./group6/reference/d1fcdc2-d1ycc__.fasta

./group6/reference/d1fcdc2-d3c2c__.fasta

./group6/reference/d1gu2a_-d1h32a1.fasta

./group6/reference/d1gu2a_-d1iqca1.fasta

./group6/reference/d1gu2a_-d1kb0a1.fasta

./group6/reference/d1gu2a_-d1kv9a1.fasta

./group6/reference/d1gu2a_-d1mg2d_.fasta

./group6/reference/d1gu2a_-d1nira1.fasta

./group6/reference/d1gu2a_-d1ycc__.fasta

./group6/reference/d1h32a1-d1iqca1.fasta

./group6/reference/d1h32a1-d1iqca2.fasta

./group6/reference/d1h32a1-d1kv9a1.fasta

./group6/reference/d1h32a1-d1mg2d_.fasta

./group6/reference/d1h32a1-d1qn2a_.fasta

./group6/reference/d1hroa_-d1iqca1.fasta

./group6/reference/d1hroa_-d1kb0a1.fasta

./group6/reference/d1hroa_-d1nira1.fasta

./group6/reference/d1iqca1-d1kb0a1.fasta

./group6/reference/d1iqca1-d1kv9a1.fasta

./group6/reference/d1iqca1-d1mg2d_.fasta

./group6/reference/d1iqca1-d1nira1.fasta

./group6/reference/d1iqca1-d1qksa1.fasta

./group6/reference/d1iqca1-d1ql3a_.fasta

./group6/reference/d1iqca1-d1qn2a_.fasta

./group6/reference/d1iqca1-d3c2c__.fasta

./group6/reference/d1iqca2-d1nira1.fasta

./group6/reference/d1iqca2-d1qksa1.fasta

./group6/reference/d1iqca2-d1ql3a_.fasta

./group6/reference/d1iqca2-d1ycc__.fasta

./group6/reference/d1kb0a1-d1ql3a_.fasta

./group6/reference/d1kb0a1-d1qn2a_.fasta

./group6/reference/d1kb0a1-d1ycc__.fasta

./group6/reference/d1kv9a1-d1qksa1.fasta

./group6/reference/d1kv9a1-d1ql3a_.fasta

./group6/reference/d1kv9a1-d1qn2a_.fasta

./group6/reference/d1kv9a1-d1ycc__.fasta

./group6/reference/d1kv9a1-d3c2c__.fasta

./group6/reference/d1mg2d_-d1ql3a_.fasta

./group6/reference/d1mg2d_-d1qn2a_.fasta

./group6/reference/d1mg2d_-d1ycc__.fasta

./group6/reference/d1nira1-d1ql3a_.fasta

./group6/reference/d1nira1-d1qn2a_.fasta

./group6/reference/d1nira1-d1ycc__.fasta

./group6/reference/d1nira1-d3c2c__.fasta

./group6/reference/d1qksa1-d1ycc__.fasta

./group6/reference/d1qksa1-d3c2c__.fasta

./group7/reference/d1b72b_-d1bl0a2.fasta

./group7/reference/d1b72b_-d1g2ha_.fasta

./group7/reference/d1b72b_-d1hlva2.fasta

./group7/reference/d1b72b_-d1igna1.fasta

./group7/reference/d1b72b_-d1iufa1.fasta

./group7/reference/d1b72b_-d1jt6a1.fasta

./group7/reference/d1b72b_-d1k78a1.fasta

./group7/reference/d1b72b_-d1pb6a1.fasta

./group7/reference/d1b72b_-d2ezi__.fasta

./group7/reference/d1b72b_-d2ezl__.fasta

./group7/reference/d1bl0a2-d1bw5__.fasta

./group7/reference/d1bl0a2-d1fexa_.fasta

./group7/reference/d1bl0a2-d1fjla_.fasta

./group7/reference/d1bl0a2-d1g2ha_.fasta

./group7/reference/d1bl0a2-d1hlva1.fasta

./group7/reference/d1bl0a2-d1igna1.fasta

./group7/reference/d1bl0a2-d1iufa1.fasta

./group7/reference/d1bl0a2-d1jgga_.fasta

./group7/reference/d1bl0a2-d1k78a2.fasta

./group7/reference/d1bl0a2-d2ezl__.fasta

./group7/reference/d1bl0a2-d2tct_1.fasta

./group7/reference/d1bw5__-d1d5ya2.fasta

./group7/reference/d1bw5__-d1fexa_.fasta

./group7/reference/d1bw5__-d1hlva1.fasta

./group7/reference/d1bw5__-d1hlva2.fasta

./group7/reference/d1bw5__-d1iufa1.fasta

./group7/reference/d1bw5__-d1k78a2.fasta

./group7/reference/d1bw5__-d2ezi__.fasta

./group7/reference/d1bw5__-d2ezl__.fasta

./group7/reference/d1d5ya2-d1fjla_.fasta

./group7/reference/d1d5ya2-d1g2ha_.fasta

./group7/reference/d1d5ya2-d1hlva1.fasta

./group7/reference/d1d5ya2-d1hlva2.fasta

./group7/reference/d1d5ya2-d1iufa2.fasta

./group7/reference/d1d5ya2-d1jt6a1.fasta

./group7/reference/d1d5ya2-d1k78a1.fasta

./group7/reference/d1d5ya2-d1k78a2.fasta

./group7/reference/d1d5ya2-d1pb6a1.fasta

./group7/reference/d1d5ya2-d2ezl__.fasta

./group7/reference/d1d5ya2-d2tct_1.fasta

./group7/reference/d1e3oc1-d1fexa_.fasta

./group7/reference/d1e3oc1-d1hlva1.fasta

./group7/reference/d1e3oc1-d1iufa2.fasta

./group7/reference/d1e3oc1-d2tct_1.fasta

./group7/reference/d1fexa_-d1hlva1.fasta

./group7/reference/d1fexa_-d1hlva2.fasta

./group7/reference/d1fexa_-d1ig7a_.fasta

./group7/reference/d1fexa_-d1irza_.fasta

./group7/reference/d1fexa_-d1jgga_.fasta

./group7/reference/d1fexa_-d1jt6a1.fasta

./group7/reference/d1fexa_-d1k61a_.fasta

./group7/reference/d1fexa_-d1k78a1.fasta

./group7/reference/d1fexa_-d1k78a2.fasta

./group7/reference/d1fexa_-d1pb6a1.fasta

./group7/reference/d1fexa_-d1qrya_.fasta

./group7/reference/d1fexa_-d2ezi__.fasta

./group7/reference/d1fexa_-d2ezl__.fasta

./group7/reference/d1fexa_-d2tct_1.fasta

./group7/reference/d1fjla_-d1hlva1.fasta

./group7/reference/d1fjla_-d1hlva2.fasta

./group7/reference/d1fjla_-d1igna1.fasta

./group7/reference/d1fjla_-d1irza_.fasta

./group7/reference/d1fjla_-d1iufa2.fasta

./group7/reference/d1fjla_-d2ezi__.fasta

./group7/reference/d1fjla_-d2tct_1.fasta

./group7/reference/d1g2ha_-d1iufa1.fasta

./group7/reference/d1g2ha_-d1k61a_.fasta

./group7/reference/d1g2ha_-d1k78a2.fasta

./group7/reference/d1g2ha_-d1pb6a1.fasta

./group7/reference/d1g2ha_-d2ezl__.fasta

./group7/reference/d1g2ha_-d2tct_1.fasta

./group7/reference/d1hlva1-d1iufa1.fasta

./group7/reference/d1hlva1-d1iufa2.fasta

./group7/reference/d1hlva1-d1jgga_.fasta

./group7/reference/d1hlva1-d1jt6a1.fasta

./group7/reference/d1hlva1-d1k78a1.fasta

./group7/reference/d1hlva1-d1pb6a1.fasta

./group7/reference/d1hlva1-d2tct_1.fasta

./group7/reference/d1hlva2-d1ig7a_.fasta

./group7/reference/d1hlva2-d1igna1.fasta

./group7/reference/d1hlva2-d1iufa1.fasta

./group7/reference/d1hlva2-d1iufa2.fasta

./group7/reference/d1hlva2-d1jgga_.fasta

./group7/reference/d1hlva2-d1jt6a1.fasta

./group7/reference/d1hlva2-d1k61a_.fasta

./group7/reference/d1hlva2-d1k78a1.fasta

./group7/reference/d1hlva2-d1k78a2.fasta

./group7/reference/d1hlva2-d1qrya_.fasta

./group7/reference/d1ig7a_-d1igna1.fasta

./group7/reference/d1ig7a_-d1iufa2.fasta

./group7/reference/d1ig7a_-d1jt6a1.fasta

./group7/reference/d1ig7a_-d1k78a1.fasta

./group7/reference/d1ig7a_-d1k78a2.fasta

./group7/reference/d1igna1-d1irza_.fasta

./group7/reference/d1igna1-d1iufa1.fasta

./group7/reference/d1igna1-d1k78a1.fasta

./group7/reference/d1igna1-d1k78a2.fasta

./group7/reference/d1igna1-d1pb6a1.fasta

./group7/reference/d1igna1-d2ezi__.fasta

./group7/reference/d1irza_-d1iufa2.fasta

./group7/reference/d1irza_-d1k61a_.fasta

./group7/reference/d1irza_-d1pb6a1.fasta

./group7/reference/d1irza_-d1qrya_.fasta

./group7/reference/d1irza_-d2ezi__.fasta

./group7/reference/d1irza_-d2tct_1.fasta

./group7/reference/d1iufa1-d1k61a_.fasta

./group7/reference/d1iufa1-d1k78a1.fasta

./group7/reference/d1iufa1-d1k78a2.fasta

./group7/reference/d1iufa1-d1pb6a1.fasta

./group7/reference/d1iufa1-d1qrya_.fasta

./group7/reference/d1iufa1-d2ezi__.fasta

./group7/reference/d1iufa2-d1jgga_.fasta

./group7/reference/d1iufa2-d2ezl__.fasta

./group7/reference/d1jgga_-d1jt6a1.fasta

./group7/reference/d1jt6a1-d1k61a_.fasta

./group7/reference/d1jt6a1-d1k78a2.fasta

./group7/reference/d1jt6a1-d2ezl__.fasta

./group7/reference/d1k61a_-d1k78a2.fasta

./group7/reference/d1k61a_-d1pb6a1.fasta

./group7/reference/d1k61a_-d2ezi__.fasta

./group7/reference/d1k78a1-d1pb6a1.fasta

./group7/reference/d1k78a1-d2ezl__.fasta

./group7/reference/d1k78a2-d1qrya_.fasta

./group7/reference/d1k78a2-d2ezi__.fasta

./group7/reference/d1k78a2-d2ezl__.fasta

./group7/reference/d1k78a2-d2tct_1.fasta

./group7/reference/d1pb6a1-d2ezl__.fasta

./group7/reference/d1qrya_-d2ezi__.fasta

./group7/reference/d2ezi__-d2ezl__.fasta

./group7/reference/d2ezi__-d2tct_1.fasta

./group10/reference/d1bjaa_-d1d5va_.fasta

./group10/reference/d1bjaa_-d1e17a_.fasta

./group10/reference/d1bjaa_-d1f1za1.fasta

./group10/reference/d1bjaa_-d1fp1d1.fasta

./group10/reference/d1bjaa_-d1fp2a1.fasta

./group10/reference/d1bjaa_-d1hkqa_.fasta

./group10/reference/d1bjaa_-d1hks__.fasta

./group10/reference/d1bjaa_-d1hsja1.fasta

./group10/reference/d1bjaa_-d1jgsa_.fasta

./group10/reference/d1bjaa_-d1ka8a_.fasta

./group10/reference/d1bjaa_-d1ldja1.fasta

./group10/reference/d1bjaa_-d1o7fa1.fasta

./group10/reference/d1bjaa_-d1p4xa1.fasta

./group10/reference/d1bjaa_-d1p4xa2.fasta

./group10/reference/d1bjaa_-d1puee_.fasta

./group10/reference/d1bjaa_-d1smta_.fasta

./group10/reference/d1bjaa_-d2irfg_.fasta

./group10/reference/d1bm9a_-d1e17a_.fasta

./group10/reference/d1bm9a_-d1fp1d1.fasta

./group10/reference/d1bm9a_-d1fp2a1.fasta

./group10/reference/d1bm9a_-d1fsha_.fasta

./group10/reference/d1bm9a_-d1hks__.fasta

./group10/reference/d1bm9a_-d1jgsa_.fasta

./group10/reference/d1bm9a_-d1ldja1.fasta

./group10/reference/d1bm9a_-d1lj9a_.fasta

./group10/reference/d1bm9a_-d1md0a_.fasta

./group10/reference/d1bm9a_-d1omia1.fasta

./group10/reference/d1bm9a_-d1p4xa2.fasta

./group10/reference/d1bm9a_-d1puee_.fasta

./group10/reference/d1bm9a_-d1repc2.fasta

./group10/reference/d1bm9a_-d1smta_.fasta

./group10/reference/d1bm9a_-d2irfg_.fasta

./group10/reference/d1d5va_-d1fp1d1.fasta

./group10/reference/d1d5va_-d1hks__.fasta

./group10/reference/d1d5va_-d1hsja1.fasta

./group10/reference/d1d5va_-d1jgsa_.fasta

./group10/reference/d1d5va_-d1ka8a_.fasta

./group10/reference/d1d5va_-d1ldja1.fasta

./group10/reference/d1d5va_-d1p4xa1.fasta

./group10/reference/d1d5va_-d1p4xa2.fasta

./group10/reference/d1d5va_-d2hts__.fasta

./group10/reference/d1e17a_-d1fp1d1.fasta

./group10/reference/d1e17a_-d1fp2a1.fasta

./group10/reference/d1e17a_-d1hks__.fasta

./group10/reference/d1e17a_-d1ka8a_.fasta

./group10/reference/d1e17a_-d1ldja1.fasta

./group10/reference/d1e17a_-d1o7fa1.fasta

./group10/reference/d1e17a_-d1p4xa1.fasta

./group10/reference/d1e17a_-d1p4xa2.fasta

./group10/reference/d1e17a_-d2irfg_.fasta

./group10/reference/d1f1za1-d1fp1d1.fasta

./group10/reference/d1f1za1-d1fp2a1.fasta

./group10/reference/d1f1za1-d1fsha_.fasta

./group10/reference/d1f1za1-d1hkqa_.fasta

./group10/reference/d1f1za1-d1jgsa_.fasta

./group10/reference/d1f1za1-d1lj9a_.fasta

./group10/reference/d1f1za1-d1md0a_.fasta

./group10/reference/d1f1za1-d1p4xa1.fasta

./group10/reference/d1f1za1-d1p4xa2.fasta

./group10/reference/d1f1za1-d1puee_.fasta

./group10/reference/d1f1za1-d1repc2.fasta

./group10/reference/d1f1za1-d1smta_.fasta

./group10/reference/d1f1za1-d2hts__.fasta

./group10/reference/d1fp1d1-d1hkqa_.fasta

./group10/reference/d1fp1d1-d1hsja1.fasta

./group10/reference/d1fp1d1-d1lj9a_.fasta

./group10/reference/d1fp1d1-d1o7fa1.fasta

./group10/reference/d1fp1d1-d1omia1.fasta

./group10/reference/d1fp1d1-d1p4xa1.fasta

./group10/reference/d1fp1d1-d1p4xa2.fasta

./group10/reference/d1fp1d1-d1puee_.fasta

./group10/reference/d1fp1d1-d1repc2.fasta

./group10/reference/d1fp1d1-d1smta_.fasta

./group10/reference/d1fp1d1-d2hts__.fasta

./group10/reference/d1fp1d1-d2irfg_.fasta

./group10/reference/d1fp2a1-d1fsha_.fasta

./group10/reference/d1fp2a1-d1hsja1.fasta

./group10/reference/d1fp2a1-d1ldja1.fasta

./group10/reference/d1fp2a1-d1lj9a_.fasta

./group10/reference/d1fp2a1-d1o7fa1.fasta

./group10/reference/d1fp2a1-d1p4xa2.fasta

./group10/reference/d1fp2a1-d1puee_.fasta

./group10/reference/d1fp2a1-d1repc2.fasta

./group10/reference/d1fp2a1-d1smta_.fasta

./group10/reference/d1fp2a1-d2hts__.fasta

./group10/reference/d1fp2a1-d2irfg_.fasta

./group10/reference/d1fsha_-d1hks__.fasta

./group10/reference/d1fsha_-d1p4xa2.fasta

./group10/reference/d1fsha_-d1smta_.fasta

./group10/reference/d1hkqa_-d1hks__.fasta

./group10/reference/d1hkqa_-d1hsja1.fasta

./group10/reference/d1hkqa_-d1lj9a_.fasta

./group10/reference/d1hkqa_-d1md0a_.fasta

./group10/reference/d1hkqa_-d1p4xa1.fasta

./group10/reference/d1hkqa_-d1p4xa2.fasta

./group10/reference/d1hkqa_-d1puee_.fasta

./group10/reference/d1hkqa_-d1repc2.fasta

./group10/reference/d1hkqa_-d1smta_.fasta

./group10/reference/d1hkqa_-d2irfg_.fasta

./group10/reference/d1hks__-d1hsja1.fasta

./group10/reference/d1hks__-d1jgsa_.fasta

./group10/reference/d1hks__-d1ka8a_.fasta

./group10/reference/d1hks__-d1ldja1.fasta

./group10/reference/d1hks__-d1lj9a_.fasta

./group10/reference/d1hks__-d1omia1.fasta

./group10/reference/d1hks__-d1p4xa1.fasta

./group10/reference/d1hks__-d1puee_.fasta

./group10/reference/d1hks__-d1repc2.fasta

./group10/reference/d1hks__-d1smta_.fasta

./group10/reference/d1hks__-d2irfg_.fasta

./group10/reference/d1hsja1-d1jgsa_.fasta

./group10/reference/d1hsja1-d1ldja1.fasta

./group10/reference/d1hsja1-d1lj9a_.fasta

./group10/reference/d1hsja1-d1md0a_.fasta

./group10/reference/d1hsja1-d1o7fa1.fasta

./group10/reference/d1hsja1-d1p4xa1.fasta

./group10/reference/d1hsja1-d1p4xa2.fasta

./group10/reference/d1hsja1-d1puee_.fasta

./group10/reference/d1hsja1-d1smta_.fasta

./group10/reference/d1jgsa_-d1ka8a_.fasta

./group10/reference/d1jgsa_-d1ldja1.fasta

./group10/reference/d1jgsa_-d1lj9a_.fasta

./group10/reference/d1jgsa_-d1md0a_.fasta

./group10/reference/d1jgsa_-d1p4xa1.fasta

./group10/reference/d1jgsa_-d1p4xa2.fasta

./group10/reference/d1jgsa_-d1puee_.fasta

./group10/reference/d1jgsa_-d1smta_.fasta

./group10/reference/d1ka8a_-d1o7fa1.fasta

./group10/reference/d1ka8a_-d1omia1.fasta

./group10/reference/d1ka8a_-d1p4xa2.fasta

./group10/reference/d1ka8a_-d1smta_.fasta

./group10/reference/d1ldja1-d1lj9a_.fasta

./group10/reference/d1ldja1-d1o7fa1.fasta

./group10/reference/d1ldja1-d1p4xa1.fasta

./group10/reference/d1ldja1-d1p4xa2.fasta

./group10/reference/d1ldja1-d1puee_.fasta

./group10/reference/d1ldja1-d2irfg_.fasta

./group10/reference/d1lj9a_-d1o7fa1.fasta

./group10/reference/d1lj9a_-d1p4xa1.fasta

./group10/reference/d1lj9a_-d1p4xa2.fasta

./group10/reference/d1lj9a_-d1puee_.fasta

./group10/reference/d1lj9a_-d2hts__.fasta

./group10/reference/d1md0a_-d1o7fa1.fasta

./group10/reference/d1md0a_-d1repc2.fasta

./group10/reference/d1md0a_-d1smta_.fasta

./group10/reference/d1md0a_-d2hts__.fasta

./group10/reference/d1md0a_-d2irfg_.fasta

./group10/reference/d1o7fa1-d1p4xa1.fasta

./group10/reference/d1o7fa1-d1p4xa2.fasta

./group10/reference/d1o7fa1-d1puee_.fasta

./group10/reference/d1o7fa1-d1repc2.fasta

./group10/reference/d1o7fa1-d1smta_.fasta

./group10/reference/d1o7fa1-d2irfg_.fasta

./group10/reference/d1omia1-d1p4xa1.fasta

./group10/reference/d1omia1-d2hts__.fasta

./group10/reference/d1p4xa1-d1puee_.fasta

./group10/reference/d1p4xa1-d1repc2.fasta

./group10/reference/d1p4xa1-d1smta_.fasta

./group10/reference/d1p4xa1-d2hts__.fasta

./group10/reference/d1p4xa2-d1repc2.fasta

./group10/reference/d1p4xa2-d1smta_.fasta

./group10/reference/d1p4xa2-d2irfg_.fasta

./group10/reference/d1puee_-d1repc2.fasta

./group10/reference/d1puee_-d2hts__.fasta

./group10/reference/d1repc2-d1smta_.fasta

./group10/reference/d1repc2-d2hts__.fasta

./group10/reference/d1repc2-d2irfg_.fasta

./group10/reference/d1smta_-d2irfg_.fasta

./group10/reference/d2hts__-d2irfg_.fasta

./group11/reference/d1a04a1-d1gxqa_.fasta

./group11/reference/d1a04a1-d1opc__.fasta

./group11/reference/d1fc3a_-d1fsea_.fasta

./group11/reference/d1fc3a_-d1gxqa_.fasta

./group11/reference/d1fsea_-d1gxqa_.fasta

./group11/reference/d1fsea_-d1opc__.fasta

./group11/reference/d1gxqa_-d1p4wa_.fasta

./group11/reference/d1opc__-d1p4wa_.fasta

./group12/reference/d1efub3-d1f4ia_.fasta

./group12/reference/d1efub3-d1ifya_.fasta

./group12/reference/d1efub3-d1oaia_.fasta

./group12/reference/d1efub3-d1otra_.fasta

./group12/reference/d1f4ia_-d1otra_.fasta

./group12/reference/d1ifya_-d1otra_.fasta

./group12/reference/d1oaia_-d1otra_.fasta

./group14/reference/d1d4ua1-d1g4da_.fasta

./group14/reference/d1d4ua1-d1j9ia_.fasta

./group14/reference/d1exja1-d1g4da_.fasta

./group14/reference/d1exja1-d1lx8a_.fasta

./group14/reference/d1exja1-d1tns__.fasta

./group14/reference/d1g4da_-d1j9ia_.fasta

./group14/reference/d1g4da_-d1jbga_.fasta

./group14/reference/d1g4da_-d1jjcb2.fasta

./group14/reference/d1g4da_-d1lx8a_.fasta

./group14/reference/d1g4da_-d1nd9a_.fasta

./group14/reference/d1j9ia_-d1lx8a_.fasta

./group14/reference/d1j9ia_-d1nd9a_.fasta

./group14/reference/d1j9ia_-d1tns__.fasta

./group14/reference/d1jbga_-d1nd9a_.fasta

./group14/reference/d1jjcb2-d1tns__.fasta

./group14/reference/d1lx8a_-d1nd9a_.fasta

./group14/reference/d1nd9a_-d1tns__.fasta

./group15/reference/d1cuna1-d1hcia1.fasta

./group15/reference/d1cuna1-d1hcia4.fasta

./group15/reference/d1cuna2-d1hcia1.fasta

./group15/reference/d1cuna2-d1hcia4.fasta

./group15/reference/d1cuna2-d1quua2.fasta

./group15/reference/d1hcia1-d1hcia4.fasta

./group15/reference/d1hcia1-d1quua1.fasta

./group15/reference/d1hcia1-d1quua2.fasta

./group15/reference/d1hcia4-d1quua1.fasta

./group15/reference/d1hcia4-d1quua2.fasta

./group15/reference/d1quua1-d2spca_.fasta

./group16/reference/d1jnra1-d1qlaa1.fasta

./group17/reference/d1deeg_-d1gab__.fasta

./group18/reference/d1erd__-d1hd6a_.fasta

./group18/reference/d1hd6a_-d2erl__.fasta

./group20/reference/d1a32__-d1fyja_.fasta

./group21/reference/d1ckta_-d1i11a_.fasta

./group21/reference/d1ckta_-d2lefa_.fasta

./group21/reference/d1i11a_-d1k99a_.fasta

./group21/reference/d1k99a_-d1qrva_.fasta

./group22/reference/d1b67a_-d1bh9a_.fasta

./group22/reference/d1bh9a_-d1bh9b_.fasta

./group22/reference/d1bh9a_-d1kx5a_.fasta

./group22/reference/d1bh9a_-d1kx5b_.fasta

./group22/reference/d1bh9a_-d1kx5c_.fasta

./group22/reference/d1bh9a_-d1kx5d_.fasta

./group22/reference/d1bh9b_-d1kx5a_.fasta

./group22/reference/d1bh9b_-d1kx5c_.fasta

./group22/reference/d1bh9b_-d1kx5d_.fasta

./group22/reference/d1bh9b_-d1n1ja_.fasta

./group22/reference/d1bh9b_-d1n1jb_.fasta

./group22/reference/d1jfib_-d1kx5a_.fasta

./group22/reference/d1jfib_-d1kx5b_.fasta

./group22/reference/d1jfib_-d1kx5c_.fasta

./group22/reference/d1jfib_-d1kx5d_.fasta

./group22/reference/d1kx5a_-d1kx5b_.fasta

./group22/reference/d1kx5a_-d1kx5d_.fasta

./group22/reference/d1kx5a_-d1n1ja_.fasta

./group22/reference/d1kx5a_-d1n1jb_.fasta

./group22/reference/d1kx5b_-d1kx5c_.fasta

./group22/reference/d1kx5b_-d1kx5d_.fasta

./group22/reference/d1kx5b_-d1n1ja_.fasta

./group22/reference/d1kx5c_-d1n1ja_.fasta

./group22/reference/d1kx5d_-d1n1jb_.fasta

./group23/reference/d1jafa_-d1mqva_.fasta

./group23/reference/d1jafa_-d256ba_.fasta

./group23/reference/d256ba_-d2ccya_.fasta

./group25/reference/d1is2a1-d3mdda1.fasta

./group26/reference/d1afra_-d1h0oa_.fasta

./group26/reference/d1afra_-d1jgca_.fasta

./group26/reference/d1afra_-d1ji4a_.fasta

./group26/reference/d1afra_-d1jiga_.fasta

./group26/reference/d1afra_-d1jkva_.fasta

./group26/reference/d1afra_-d1mxra_.fasta

./group26/reference/d1afra_-d1o9ra_.fasta

./group26/reference/d1afra_-d1rcd__.fasta

./group26/reference/d1euma_-d1jgca_.fasta

./group26/reference/d1euma_-d1lkoa1.fasta

./group26/reference/d1euma_-d1nfva_.fasta

./group26/reference/d1h0oa_-d1jkva_.fasta

./group26/reference/d1h0oa_-d1qgha_.fasta

./group26/reference/d1jgca_-d1ji4a_.fasta

./group26/reference/d1jgca_-d1jkva_.fasta

./group26/reference/d1jgca_-d1lkoa1.fasta

./group26/reference/d1jgca_-d1mtyd_.fasta

./group26/reference/d1jgca_-d1qgha_.fasta

./group26/reference/d1jgca_-d1rcd__.fasta

./group26/reference/d1ji4a_-d1lkoa1.fasta

./group26/reference/d1ji4a_-d1mtyb_.fasta

./group26/reference/d1ji4a_-d1nfva_.fasta

./group26/reference/d1ji4a_-d1rcd__.fasta

./group26/reference/d1jiga_-d1jkva_.fasta

./group26/reference/d1jiga_-d1lkoa1.fasta

./group26/reference/d1jiga_-d1nfva_.fasta

./group26/reference/d1jkva_-d1kgna_.fasta

./group26/reference/d1jkva_-d1lkoa1.fasta

./group26/reference/d1jkva_-d1mtyd_.fasta

./group26/reference/d1jkva_-d1mxra_.fasta

./group26/reference/d1jkva_-d1nfva_.fasta

./group26/reference/d1jkva_-d1o9ra_.fasta

./group26/reference/d1jkva_-d1qgha_.fasta

./group26/reference/d1kgna_-d1o9ra_.fasta

./group26/reference/d1kgna_-d1rcd__.fasta

./group26/reference/d1lkoa1-d1o9ra_.fasta

./group26/reference/d1lkoa1-d1qgha_.fasta

./group26/reference/d1lkoa1-d1rcd__.fasta

./group26/reference/d1mtyb_-d1mtyd_.fasta

./group26/reference/d1mtyb_-d1nfva_.fasta

./group26/reference/d1mtyd_-d1nfva_.fasta

./group26/reference/d1nfva_-d1rcd__.fasta

./group27/reference/d1au1a_-d1etea_.fasta

./group27/reference/d1au1a_-d1hula_.fasta

./group27/reference/d1au1a_-d1jli__.fasta

./group27/reference/d1au1a_-d1m4ra_.fasta

./group27/reference/d1au1a_-d2gmfa_.fasta

./group27/reference/d1au1a_-d2ilk__.fasta

./group27/reference/d1d9ca_-d1etea_.fasta

./group27/reference/d1d9ca_-d1jli__.fasta

./group27/reference/d1d9ca_-d1lki__.fasta

./group27/reference/d1d9ca_-d1m4ra_.fasta

./group27/reference/d1eera_-d1huw__.fasta

./group27/reference/d1eera_-d1jli__.fasta

./group27/reference/d1eera_-d1lki__.fasta

./group27/reference/d1eera_-d1lqsl_.fasta

./group27/reference/d1eera_-d2gmfa_.fasta

./group27/reference/d1eera_-d2ilk__.fasta

./group27/reference/d1etea_-d1jli__.fasta

./group27/reference/d1etea_-d1n9da_.fasta

./group27/reference/d1etea_-d2gmfa_.fasta

./group27/reference/d1etea_-d2ilk__.fasta

./group27/reference/d1hula_-d1hzia_.fasta

./group27/reference/d1hula_-d1i1rb_.fasta

./group27/reference/d1hula_-d2gmfa_.fasta

./group27/reference/d1hula_-d2ilk__.fasta

./group27/reference/d1huw__-d1i1rb_.fasta

./group27/reference/d1huw__-d1jli__.fasta

./group27/reference/d1huw__-d1lqsl_.fasta

./group27/reference/d1huw__-d1m4ra_.fasta

./group27/reference/d1huw__-d1n9da_.fasta

./group27/reference/d1huw__-d2ilk__.fasta

./group27/reference/d1hzia_-d1i1rb_.fasta

./group27/reference/d1hzia_-d1lki__.fasta

./group27/reference/d1hzia_-d1lqsl_.fasta

./group27/reference/d1hzia_-d2gmfa_.fasta

./group27/reference/d1hzia_-d2ilk__.fasta

./group27/reference/d1i1rb_-d1lki__.fasta

./group27/reference/d1i1rb_-d1n9da_.fasta

./group27/reference/d1jli__-d1lki__.fasta

./group27/reference/d1jli__-d1m4ra_.fasta

./group27/reference/d1jli__-d1n9da_.fasta

./group27/reference/d1jli__-d2gmfa_.fasta

./group27/reference/d1jli__-d2ilk__.fasta

./group27/reference/d1lki__-d1m4ra_.fasta

./group27/reference/d1lki__-d2gmfa_.fasta

./group27/reference/d1lki__-d2ilk__.fasta

./group27/reference/d1lqsl_-d1m4ra_.fasta

./group27/reference/d1lqsl_-d1n9da_.fasta

./group27/reference/d1lqsl_-d2gmfa_.fasta

./group27/reference/d1m4ra_-d1n9da_.fasta

./group27/reference/d1n9da_-d2ilk__.fasta

./group27/reference/d2gmfa_-d2ilk__.fasta

./group28/reference/d1a8h_1-d1f4la1.fasta

./group28/reference/d1a8h_1-d1f7ua1.fasta

./group28/reference/d1a8h_1-d1ffya1.fasta

./group28/reference/d1a8h_1-d1ile_1.fasta

./group28/reference/d1a8h_1-d1iq0a1.fasta

./group28/reference/d1a8h_1-d1li5a1.fasta

./group28/reference/d1f4la1-d1ffya1.fasta

./group28/reference/d1f4la1-d1ile_1.fasta

./group28/reference/d1f4la1-d1iq0a1.fasta

./group28/reference/d1f4la1-d1li5a1.fasta

./group28/reference/d1f7ua1-d1ile_1.fasta

./group28/reference/d1f7ua1-d1li5a1.fasta

./group28/reference/d1ile_1-d1iq0a1.fasta

./group28/reference/d1ile_1-d1li5a1.fasta

./group28/reference/d1iq0a1-d1ivsa2.fasta

./group28/reference/d1iq0a1-d1li5a1.fasta

./group29/reference/d1af8__-d1dnya_.fasta

./group29/reference/d1af8__-d1dv5a_.fasta

./group29/reference/d1dnya_-d1dv5a_.fasta

./group29/reference/d1dnya_-d1klpa_.fasta

./group29/reference/d1dnya_-d1n8la_.fasta

./group29/reference/d1dv5a_-d1klpa_.fasta

./group29/reference/d1dv5a_-d1n8la_.fasta

./group29/reference/d1klpa_-d1n8la_.fasta

./group29/reference/d1l0ia_-d1n8la_.fasta

./group30/reference/d1d1da1-d2eiaa1.fasta

./group31/reference/d1fts_1-d1j8mf1.fasta

./group32/reference/d1eqfa1-d1jspb_.fasta

./group33/reference/d1adr__-d1e3oc2.fasta

./group33/reference/d1adr__-d1efaa1.fasta

./group33/reference/d1adr__-d1ic8a2.fasta

./group33/reference/d1adr__-d1lmb3_.fasta

./group33/reference/d1adr__-d1ner__.fasta

./group33/reference/d1adr__-d1uxd__.fasta

./group33/reference/d1adr__-d1vpwa1.fasta

./group33/reference/d1b0na2-d1d1la_.fasta

./group33/reference/d1b0na2-d1e3oc2.fasta

./group33/reference/d1b0na2-d1efaa1.fasta

./group33/reference/d1b0na2-d1ic8a2.fasta

./group33/reference/d1b0na2-d1lmb3_.fasta

./group33/reference/d1b0na2-d1ner__.fasta

./group33/reference/d1b0na2-d1vpwa1.fasta

./group33/reference/d1d1la_-d1e3oc2.fasta

./group33/reference/d1d1la_-d1efaa1.fasta

./group33/reference/d1d1la_-d1ic8a2.fasta

./group33/reference/d1d1la_-d1lmb3_.fasta

./group33/reference/d1d1la_-d1ner__.fasta

./group33/reference/d1d1la_-d1r69__.fasta

./group33/reference/d1d1la_-d1uxd__.fasta

./group33/reference/d1e3oc2-d1ic8a2.fasta

./group33/reference/d1e3oc2-d1lmb3_.fasta

./group33/reference/d1e3oc2-d1ner__.fasta

./group33/reference/d1e3oc2-d1uxd__.fasta

./group33/reference/d1e3oc2-d1vpwa1.fasta

./group33/reference/d1e3oc2-d1zug__.fasta

./group33/reference/d1efaa1-d1ic8a2.fasta

./group33/reference/d1efaa1-d1lmb3_.fasta

./group33/reference/d1efaa1-d1ner__.fasta

./group33/reference/d1efaa1-d1r69__.fasta

./group33/reference/d1efaa1-d1zug__.fasta

./group33/reference/d1ic8a2-d1ner__.fasta

./group33/reference/d1ic8a2-d1uxd__.fasta

./group33/reference/d1ic8a2-d1vpwa1.fasta

./group33/reference/d1ic8a2-d1zug__.fasta

./group33/reference/d1lmb3_-d1ner__.fasta

./group33/reference/d1lmb3_-d1uxd__.fasta

./group33/reference/d1lmb3_-d1vpwa1.fasta

./group33/reference/d1ner__-d1r69__.fasta

./group33/reference/d1ner__-d1uxd__.fasta

./group33/reference/d1ner__-d1zug__.fasta

./group33/reference/d1r69__-d1uxd__.fasta

./group33/reference/d1uxd__-d1zug__.fasta

./group33/reference/d1vpwa1-d1zug__.fasta

./group34/reference/d1an4a_-d1mdya_.fasta

./group35/reference/d1alva_-d1c07a_.fasta

./group35/reference/d1alva_-d1dgua_.fasta

./group35/reference/d1alva_-d1eg3a2.fasta

./group35/reference/d1alva_-d1el4a_.fasta

./group35/reference/d1alva_-d1exra_.fasta

./group35/reference/d1alva_-d1f8ha_.fasta

./group35/reference/d1alva_-d1ggwa_.fasta

./group35/reference/d1alva_-d1jbaa_.fasta

./group35/reference/d1alva_-d1m31a_.fasta

./group35/reference/d1alva_-d1psra_.fasta

./group35/reference/d1alva_-d1qjta_.fasta

./group35/reference/d1alva_-d1rro__.fasta

./group35/reference/d1alva_-d1sra__.fasta

./group35/reference/d1alva_-d1wdcb_.fasta

./group35/reference/d1alva_-d2pvba_.fasta

./group35/reference/d1alva_-d2sas__.fasta

./group35/reference/d1auib_-d1c07a_.fasta

./group35/reference/d1auib_-d1eg3a2.fasta

./group35/reference/d1auib_-d1ggwa_.fasta

./group35/reference/d1auib_-d1k94a_.fasta

./group35/reference/d1auib_-d1qjta_.fasta

./group35/reference/d1auib_-d1wdcb_.fasta

./group35/reference/d1auib_-d2sas__.fasta

./group35/reference/d1c07a_-d1dgua_.fasta

./group35/reference/d1c07a_-d1eg3a2.fasta

./group35/reference/d1c07a_-d1el4a_.fasta

./group35/reference/d1c07a_-d1ggwa_.fasta

./group35/reference/d1c07a_-d1jbaa_.fasta

./group35/reference/d1c07a_-d1k94a_.fasta

./group35/reference/d1c07a_-d1ncx__.fasta

./group35/reference/d1c07a_-d1psra_.fasta

./group35/reference/d1c07a_-d1sra__.fasta

./group35/reference/d1c07a_-d2pvba_.fasta

./group35/reference/d1c07a_-d2sas__.fasta

./group35/reference/d1dgua_-d1eg3a1.fasta

./group35/reference/d1dgua_-d1eg3a2.fasta

./group35/reference/d1dgua_-d1el4a_.fasta

./group35/reference/d1dgua_-d1ggwa_.fasta

./group35/reference/d1dgua_-d1k94a_.fasta

./group35/reference/d1dgua_-d1m31a_.fasta

./group35/reference/d1dgua_-d1psra_.fasta

./group35/reference/d1dgua_-d1qjta_.fasta

./group35/reference/d1dgua_-d1wdcb_.fasta

./group35/reference/d1dgua_-d1wdcc_.fasta

./group35/reference/d1dgua_-d2sas__.fasta

./group35/reference/d1eg3a1-d1exra_.fasta

./group35/reference/d1eg3a1-d1jbaa_.fasta

./group35/reference/d1eg3a1-d1k94a_.fasta

./group35/reference/d1eg3a1-d1ncx__.fasta

./group35/reference/d1eg3a1-d1rro__.fasta

./group35/reference/d1eg3a1-d1wdcb_.fasta

./group35/reference/d1eg3a1-d1wdcc_.fasta

./group35/reference/d1eg3a1-d2pvba_.fasta

./group35/reference/d1eg3a1-d2sas__.fasta

./group35/reference/d1eg3a1-d5pal__.fasta

./group35/reference/d1eg3a2-d1exra_.fasta

./group35/reference/d1eg3a2-d1f8ha_.fasta

./group35/reference/d1eg3a2-d1ggwa_.fasta

./group35/reference/d1eg3a2-d1jbaa_.fasta

./group35/reference/d1eg3a2-d1jfja_.fasta

./group35/reference/d1eg3a2-d1ncx__.fasta

./group35/reference/d1eg3a2-d1psra_.fasta

./group35/reference/d1eg3a2-d1rro__.fasta

./group35/reference/d1eg3a2-d1sra__.fasta

./group35/reference/d1eg3a2-d1wdcb_.fasta

./group35/reference/d1eg3a2-d1wdcc_.fasta

./group35/reference/d1eg3a2-d2pvba_.fasta

./group35/reference/d1eg3a2-d2sas__.fasta

./group35/reference/d1eg3a2-d2scpa_.fasta

./group35/reference/d1eg3a2-d5pal__.fasta

./group35/reference/d1el4a_-d1ggwa_.fasta

./group35/reference/d1el4a_-d1jbaa_.fasta

./group35/reference/d1el4a_-d1jfja_.fasta

./group35/reference/d1el4a_-d1k94a_.fasta

./group35/reference/d1el4a_-d1psra_.fasta

./group35/reference/d1el4a_-d1qjta_.fasta

./group35/reference/d1el4a_-d1rro__.fasta

./group35/reference/d1el4a_-d1wdcb_.fasta

./group35/reference/d1el4a_-d2sas__.fasta

./group35/reference/d1exra_-d1ggwa_.fasta

./group35/reference/d1exra_-d1m31a_.fasta

./group35/reference/d1exra_-d1sra__.fasta

./group35/reference/d1exra_-d2sas__.fasta

./group35/reference/d1f8ha_-d1ggwa_.fasta

./group35/reference/d1f8ha_-d1jbaa_.fasta

./group35/reference/d1f8ha_-d1jfja_.fasta

./group35/reference/d1f8ha_-d1m31a_.fasta

./group35/reference/d1f8ha_-d1psra_.fasta

./group35/reference/d1f8ha_-d1wdcb_.fasta

./group35/reference/d1f8ha_-d2scpa_.fasta

./group35/reference/d1ggwa_-d1jbaa_.fasta

./group35/reference/d1ggwa_-d1jfja_.fasta

./group35/reference/d1ggwa_-d1k94a_.fasta

./group35/reference/d1ggwa_-d1m31a_.fasta

./group35/reference/d1ggwa_-d1psra_.fasta

./group35/reference/d1ggwa_-d1qjta_.fasta

./group35/reference/d1ggwa_-d1rro__.fasta

./group35/reference/d1ggwa_-d1sra__.fasta

./group35/reference/d1ggwa_-d2pvba_.fasta

./group35/reference/d1ggwa_-d2sas__.fasta

./group35/reference/d1ggwa_-d5pal__.fasta

./group35/reference/d1jbaa_-d1k94a_.fasta

./group35/reference/d1jbaa_-d1qjta_.fasta

./group35/reference/d1jbaa_-d1wdcb_.fasta

./group35/reference/d1jbaa_-d2sas__.fasta

./group35/reference/d1jfja_-d1m31a_.fasta

./group35/reference/d1jfja_-d1psra_.fasta

./group35/reference/d1jfja_-d1qjta_.fasta

./group35/reference/d1jfja_-d1wdcb_.fasta

./group35/reference/d1jfja_-d1wdcc_.fasta

./group35/reference/d1jfja_-d2sas__.fasta

./group35/reference/d1k94a_-d1m31a_.fasta

./group35/reference/d1k94a_-d1ncx__.fasta

./group35/reference/d1k94a_-d1qjta_.fasta

./group35/reference/d1k94a_-d1rro__.fasta

./group35/reference/d1k94a_-d2sas__.fasta

./group35/reference/d1k94a_-d2scpa_.fasta

./group35/reference/d1m31a_-d1ncx__.fasta

./group35/reference/d1m31a_-d1qjta_.fasta

./group35/reference/d1m31a_-d1rro__.fasta

./group35/reference/d1m31a_-d1sra__.fasta

./group35/reference/d1m31a_-d1wdcb_.fasta

./group35/reference/d1m31a_-d1wdcc_.fasta

./group35/reference/d1m31a_-d2pvba_.fasta

./group35/reference/d1m31a_-d5pal__.fasta

./group35/reference/d1ncx__-d1qjta_.fasta

./group35/reference/d1ncx__-d1sra__.fasta

./group35/reference/d1ncx__-d2sas__.fasta

./group35/reference/d1psra_-d1qjta_.fasta

./group35/reference/d1psra_-d1rro__.fasta

./group35/reference/d1psra_-d1wdcb_.fasta

./group35/reference/d1psra_-d2pvba_.fasta

./group35/reference/d1psra_-d2sas__.fasta

./group35/reference/d1psra_-d2scpa_.fasta

./group35/reference/d1psra_-d5pal__.fasta

./group35/reference/d1qjta_-d1sra__.fasta

./group35/reference/d1qjta_-d1wdcc_.fasta

./group35/reference/d1qjta_-d2pvba_.fasta

./group35/reference/d1qjta_-d5pal__.fasta

./group35/reference/d1rro__-d1wdcb_.fasta

./group35/reference/d1rro__-d1wdcc_.fasta

./group35/reference/d1rro__-d2sas__.fasta

./group35/reference/d1sra__-d1wdcc_.fasta

./group35/reference/d1sra__-d2scpa_.fasta

./group35/reference/d1wdcb_-d1wdcc_.fasta

./group35/reference/d1wdcb_-d2sas__.fasta

./group35/reference/d1wdcb_-d5pal__.fasta

./group35/reference/d1wdcc_-d2pvba_.fasta

./group35/reference/d1wdcc_-d2sas__.fasta

./group35/reference/d1wdcc_-d2scpa_.fasta

./group35/reference/d2pvba_-d2sas__.fasta

./group35/reference/d2pvba_-d2scpa_.fasta

./group35/reference/d2sas__-d2scpa_.fasta

./group35/reference/d2sas__-d5pal__.fasta

./group36/reference/d1aoa_1-d1bhda_.fasta

./group36/reference/d1aoa_1-d1bkra_.fasta

./group36/reference/d1aoa_1-d1mb8a2.fasta

./group36/reference/d1aoa_2-d1bkra_.fasta

./group36/reference/d1aoa_2-d1h67a_.fasta

./group36/reference/d1bhda_-d1h67a_.fasta

./group36/reference/d1bhda_-d1mb8a1.fasta

./group36/reference/d1bkra_-d1h67a_.fasta

./group36/reference/d1bkra_-d1mb8a1.fasta

./group36/reference/d1h67a_-d1mb8a2.fasta

./group36/reference/d1mb8a1-d1mb8a2.fasta

./group37/reference/d1baza_-d1cmba_.fasta

./group37/reference/d1baza_-d1irqa_.fasta

./group37/reference/d1cmba_-d1irqa_.fasta

./group37/reference/d1cmba_-d2cpga_.fasta

./group38/reference/d1a0fa1-d1axda1.fasta

./group38/reference/d1a0fa1-d1duga1.fasta

./group38/reference/d1a0fa1-d1eema1.fasta

./group38/reference/d1a0fa1-d1fw1a1.fasta

./group38/reference/d1a0fa1-d1g7oa1.fasta

./group38/reference/d1a0fa1-d1glqa1.fasta

./group38/reference/d1a0fa1-d1gnwa1.fasta

./group38/reference/d1a0fa1-d1gula1.fasta

./group38/reference/d1a0fa1-d1iyha1.fasta

./group38/reference/d1a0fa1-d1jlva1.fasta

./group38/reference/d1a0fa1-d1k0ma1.fasta

./group38/reference/d1a0fa1-d1k3ya1.fasta

./group38/reference/d1a0fa1-d1m0ua1.fasta

./group38/reference/d1a0fa1-d1oe8a1.fasta

./group38/reference/d1a0fa1-d2gsq_1.fasta

./group38/reference/d1a0fa1-d2gsta1.fasta

./group38/reference/d1aw9_1-d1duga1.fasta

./group38/reference/d1aw9_1-d1eema1.fasta

./group38/reference/d1aw9_1-d1fw1a1.fasta

./group38/reference/d1aw9_1-d1g7oa1.fasta

./group38/reference/d1aw9_1-d1gula1.fasta

./group38/reference/d1aw9_1-d1gwca1.fasta

./group38/reference/d1aw9_1-d1iyha1.fasta

./group38/reference/d1aw9_1-d1m0ua1.fasta

./group38/reference/d1aw9_1-d1oe8a1.fasta

./group38/reference/d1aw9_1-d1pmt_1.fasta

./group38/reference/d1aw9_1-d2gsq_1.fasta

./group38/reference/d1aw9_1-d2gsta1.fasta

./group38/reference/d1axda1-d1duga1.fasta

./group38/reference/d1axda1-d1eema1.fasta

./group38/reference/d1axda1-d1f2ea1.fasta

./group38/reference/d1axda1-d1fw1a1.fasta

./group38/reference/d1axda1-d1g7oa1.fasta

./group38/reference/d1axda1-d1iyha1.fasta

./group38/reference/d1axda1-d1ljra1.fasta

./group38/reference/d1axda1-d1m0ua1.fasta

./group38/reference/d1axda1-d1pmt_1.fasta

./group38/reference/d1axda1-d2gsq_1.fasta

./group38/reference/d1axda1-d2gsta1.fasta

./group38/reference/d1duga1-d1eema1.fasta

./group38/reference/d1duga1-d1f2ea1.fasta

./group38/reference/d1duga1-d1fw1a1.fasta

./group38/reference/d1duga1-d1g7oa1.fasta

./group38/reference/d1duga1-d1gnwa1.fasta

./group38/reference/d1duga1-d1jlva1.fasta

./group38/reference/d1duga1-d1k0ma1.fasta

./group38/reference/d1duga1-d1m0ua1.fasta

./group38/reference/d1eema1-d1f2ea1.fasta

./group38/reference/d1eema1-d1fw1a1.fasta

./group38/reference/d1eema1-d1g7oa1.fasta

./group38/reference/d1eema1-d1glqa1.fasta

./group38/reference/d1eema1-d1gnwa1.fasta

./group38/reference/d1eema1-d1gula1.fasta

./group38/reference/d1eema1-d1iyha1.fasta

./group38/reference/d1eema1-d1jlva1.fasta

./group38/reference/d1eema1-d1k0ma1.fasta

./group38/reference/d1eema1-d1ljra1.fasta

./group38/reference/d1eema1-d1m0ua1.fasta

./group38/reference/d1eema1-d1pmt_1.fasta

./group38/reference/d1eema1-d2gsta1.fasta

./group38/reference/d1f2ea1-d1fw1a1.fasta

./group38/reference/d1f2ea1-d1g7oa1.fasta

./group38/reference/d1f2ea1-d1glqa1.fasta

./group38/reference/d1f2ea1-d1gnwa1.fasta

./group38/reference/d1f2ea1-d1gula1.fasta

./group38/reference/d1f2ea1-d1gwca1.fasta

./group38/reference/d1f2ea1-d1iyha1.fasta

./group38/reference/d1f2ea1-d1jlva1.fasta

./group38/reference/d1f2ea1-d1k0ma1.fasta

./group38/reference/d1f2ea1-d1ljra1.fasta

./group38/reference/d1f2ea1-d1m0ua1.fasta

./group38/reference/d1f2ea1-d1oe8a1.fasta

./group38/reference/d1f2ea1-d2gsq_1.fasta

./group38/reference/d1f2ea1-d2gsta1.fasta

./group38/reference/d1fw1a1-d1g7oa1.fasta

./group38/reference/d1fw1a1-d1glqa1.fasta

./group38/reference/d1fw1a1-d1gnwa1.fasta

./group38/reference/d1fw1a1-d1gula1.fasta

./group38/reference/d1fw1a1-d1iyha1.fasta

./group38/reference/d1fw1a1-d1k0ma1.fasta

./group38/reference/d1fw1a1-d1ljra1.fasta

./group38/reference/d1fw1a1-d1m0ua1.fasta

./group38/reference/d1fw1a1-d1oe8a1.fasta

./group38/reference/d1fw1a1-d1pmt_1.fasta

./group38/reference/d1fw1a1-d2gsq_1.fasta

./group38/reference/d1fw1a1-d2gsta1.fasta

./group38/reference/d1g7oa1-d1glqa1.fasta

./group38/reference/d1g7oa1-d1gnwa1.fasta

./group38/reference/d1g7oa1-d1gula1.fasta

./group38/reference/d1g7oa1-d1jlva1.fasta

./group38/reference/d1g7oa1-d1k3ya1.fasta

./group38/reference/d1g7oa1-d1ljra1.fasta

./group38/reference/d1g7oa1-d1m0ua1.fasta

./group38/reference/d1g7oa1-d1oe8a1.fasta

./group38/reference/d1g7oa1-d1pmt_1.fasta

./group38/reference/d1g7oa1-d2gsq_1.fasta

./group38/reference/d1glqa1-d1gnwa1.fasta

./group38/reference/d1glqa1-d1jlva1.fasta

./group38/reference/d1glqa1-d1k0ma1.fasta

./group38/reference/d1glqa1-d1oe8a1.fasta

./group38/reference/d1gnwa1-d1gula1.fasta

./group38/reference/d1gnwa1-d1gwca1.fasta

./group38/reference/d1gnwa1-d1iyha1.fasta

./group38/reference/d1gnwa1-d1k0ma1.fasta

./group38/reference/d1gnwa1-d1k3ya1.fasta

./group38/reference/d1gnwa1-d1ljra1.fasta

./group38/reference/d1gnwa1-d1m0ua1.fasta

./group38/reference/d1gnwa1-d1oe8a1.fasta

./group38/reference/d1gnwa1-d1pmt_1.fasta

./group38/reference/d1gnwa1-d2gsq_1.fasta

./group38/reference/d1gnwa1-d2gsta1.fasta

./group38/reference/d1gula1-d1gwca1.fasta

./group38/reference/d1gula1-d1jlva1.fasta

./group38/reference/d1gula1-d1k0ma1.fasta

./group38/reference/d1gula1-d1ljra1.fasta

./group38/reference/d1gula1-d1m0ua1.fasta

./group38/reference/d1gula1-d1oe8a1.fasta

./group38/reference/d1gula1-d1pmt_1.fasta

./group38/reference/d1gula1-d2gsta1.fasta

./group38/reference/d1gwca1-d1iyha1.fasta

./group38/reference/d1gwca1-d1k0ma1.fasta

./group38/reference/d1gwca1-d1m0ua1.fasta

./group38/reference/d1gwca1-d1oe8a1.fasta

./group38/reference/d1gwca1-d1pmt_1.fasta

./group38/reference/d1iyha1-d1jlva1.fasta

./group38/reference/d1iyha1-d1k0ma1.fasta

./group38/reference/d1iyha1-d1k3ya1.fasta

./group38/reference/d1iyha1-d1pmt_1.fasta

./group38/reference/d1jlva1-d1k3ya1.fasta

./group38/reference/d1jlva1-d1m0ua1.fasta

./group38/reference/d1jlva1-d1oe8a1.fasta

./group38/reference/d1jlva1-d1pmt_1.fasta

./group38/reference/d1jlva1-d2gsta1.fasta

./group38/reference/d1k0ma1-d1k3ya1.fasta

./group38/reference/d1k0ma1-d1ljra1.fasta

./group38/reference/d1k0ma1-d1m0ua1.fasta

./group38/reference/d1k0ma1-d1oe8a1.fasta

./group38/reference/d1k0ma1-d1pmt_1.fasta

./group38/reference/d1k0ma1-d2gsq_1.fasta

./group38/reference/d1k0ma1-d2gsta1.fasta

./group38/reference/d1k3ya1-d1pmt_1.fasta

./group38/reference/d1k3ya1-d2gsta1.fasta

./group38/reference/d1ljra1-d1m0ua1.fasta

./group38/reference/d1ljra1-d1oe8a1.fasta

./group38/reference/d1ljra1-d1pmt_1.fasta

./group38/reference/d1ljra1-d2gsq_1.fasta

./group38/reference/d1ljra1-d2gsta1.fasta

./group38/reference/d1m0ua1-d1oe8a1.fasta

./group38/reference/d1oe8a1-d1pmt_1.fasta

./group38/reference/d1pmt_1-d2gsq_1.fasta

./group38/reference/d1pmt_1-d2gsta1.fasta

./group39/reference/d1brwa1-d1o17a1.fasta

./group39/reference/d1khda1-d2tpt_1.fasta

./group40/reference/d1ez3a_-d1hs7a_.fasta

./group40/reference/d1ez3a_-d1lvfa_.fasta

./group40/reference/d1hs7a_-d1lvfa_.fasta

./group41/reference/d1bea__-d1fk5a_.fasta

./group41/reference/d1bea__-d1hyp__.fasta

./group41/reference/d1fk5a_-d1hyp__.fasta

./group41/reference/d1hyp__-d1l6ha_.fasta

./group44/reference/d1b0xa_-d1dxsa_.fasta

./group44/reference/d1b0xa_-d1oxja1.fasta

./group44/reference/d1b4fa_-d1bqv__.fasta

./group44/reference/d1b4fa_-d1dxsa_.fasta

./group44/reference/d1bqv__-d1dxsa_.fasta

./group44/reference/d1bqv__-d1oxja1.fasta

./group44/reference/d1dxsa_-d1oxja1.fasta

./group45/reference/d1cuk_2-d1dgsa1.fasta

./group45/reference/d1dgsa1-d1ixra1.fasta

./group45/reference/d1dgsa1-d1kfta_.fasta

./group46/reference/d1b43a1-d1tfr_1.fasta

./group46/reference/d1bgxt1-d1tfr_1.fasta

./group47/reference/d1a6s__-d1ed1a_.fasta

./group47/reference/d1a6s__-d1hiwa_.fasta

./group47/reference/d1ed1a_-d1mn8a_.fasta

./group47/reference/d1hiwa_-d1mn8a_.fasta

./group48/reference/d1l9la_-d1m12a_.fasta

./group48/reference/d1m12a_-d1nkl__.fasta

./group50/reference/d1e79a1-d1e79d1.fasta

./group50/reference/d1e79d1-d1fx0a1.fasta

./group51/reference/d1em9a_-d1m9fc_.fasta

./group51/reference/d1em9a_-d2eiaa2.fasta

./group52/reference/d1aisb1-d1bu2a2.fasta

./group52/reference/d1aisb1-d1f5qb2.fasta

./group52/reference/d1aisb1-d1h4ld_.fasta

./group52/reference/d1aisb1-d1vin_1.fasta

./group52/reference/d1aisb2-d1bu2a1.fasta

./group52/reference/d1aisb2-d1bu2a2.fasta

./group52/reference/d1aisb2-d1f5qb2.fasta

./group52/reference/d1aisb2-d1g3nc1.fasta

./group52/reference/d1aisb2-d1vin_1.fasta

./group52/reference/d1bu2a1-d1bu2a2.fasta

./group52/reference/d1bu2a1-d1f5qb2.fasta

./group52/reference/d1bu2a1-d1guxb_.fasta

./group52/reference/d1bu2a1-d1jkw_1.fasta

./group52/reference/d1bu2a1-d1jkw_2.fasta

./group52/reference/d1bu2a1-d1vin_2.fasta

./group52/reference/d1bu2a1-d1vola1.fasta

./group52/reference/d1bu2a2-d1f5qb1.fasta

./group52/reference/d1bu2a2-d1f5qb2.fasta

./group52/reference/d1bu2a2-d1g3nc1.fasta

./group52/reference/d1bu2a2-d1guxb_.fasta

./group52/reference/d1bu2a2-d1jkw_1.fasta

./group52/reference/d1bu2a2-d1jkw_2.fasta

./group52/reference/d1bu2a2-d1vin_2.fasta

./group52/reference/d1f5qb1-d1guxb_.fasta

./group52/reference/d1f5qb1-d1jkw_1.fasta

./group52/reference/d1f5qb1-d1jkw_2.fasta

./group52/reference/d1f5qb1-d1vin_2.fasta

./group52/reference/d1f5qb2-d1guxb_.fasta

./group52/reference/d1f5qb2-d1h4ld_.fasta

./group52/reference/d1f5qb2-d1jkw_1.fasta

./group52/reference/d1f5qb2-d1vin_1.fasta

./group52/reference/d1f5qb2-d1vin_2.fasta

./group52/reference/d1g3nc1-d1guxb_.fasta

./group52/reference/d1g3nc1-d1h4ld_.fasta

./group52/reference/d1g3nc1-d1jkw_1.fasta

./group52/reference/d1g3nc1-d1jkw_2.fasta

./group52/reference/d1g3nc1-d1vin_2.fasta

./group52/reference/d1g3nc1-d1vola1.fasta

./group52/reference/d1guxb_-d1jkw_1.fasta

./group52/reference/d1guxb_-d1vin_1.fasta

./group52/reference/d1guxb_-d1vola1.fasta

./group52/reference/d1h4ld_-d1jkw_1.fasta

./group52/reference/d1h4ld_-d1jkw_2.fasta

./group52/reference/d1h4ld_-d1vin_1.fasta

./group52/reference/d1jkw_1-d1jkw_2.fasta

./group52/reference/d1jkw_1-d1vin_2.fasta

./group52/reference/d1jkw_2-d1vin_2.fasta

./group52/reference/d1vin_1-d1vin_2.fasta

./group53/reference/d1a1w__-d1d2za_.fasta

./group53/reference/d1a1w__-d1d2zb_.fasta

./group53/reference/d1a1w__-d1dgna_.fasta

./group53/reference/d1a1w__-d1fada_.fasta

./group53/reference/d1a1w__-d1icha_.fasta

./group53/reference/d1a1w__-d1ngr__.fasta

./group53/reference/d1a1w__-d3ygsp_.fasta

./group53/reference/d1d2za_-d1fada_.fasta

./group53/reference/d1d2za_-d1icha_.fasta

./group53/reference/d1d2za_-d3crd__.fasta

./group53/reference/d1d2za_-d3ygsp_.fasta

./group53/reference/d1d2zb_-d1ddf__.fasta

./group53/reference/d1d2zb_-d1dgna_.fasta

./group53/reference/d1d2zb_-d1fada_.fasta

./group53/reference/d1d2zb_-d1icha_.fasta

./group53/reference/d1d2zb_-d1ngr__.fasta

./group53/reference/d1d2zb_-d3crd__.fasta

./group53/reference/d1d2zb_-d3ygsp_.fasta

./group53/reference/d1ddf__-d1dgna_.fasta

./group53/reference/d1ddf__-d1icha_.fasta

./group53/reference/d1ddf__-d1ngr__.fasta

./group53/reference/d1ddf__-d3crd__.fasta

./group53/reference/d1ddf__-d3ygsp_.fasta

./group53/reference/d1dgna_-d1fada_.fasta

./group53/reference/d1dgna_-d1ngr__.fasta

./group53/reference/d1fada_-d1n3ka_.fasta

./group53/reference/d1icha_-d3ygsp_.fasta

./group53/reference/d1n3ka_-d1ngr__.fasta

./group53/reference/d1n3ka_-d3ygsp_.fasta

./group53/reference/d1ngr__-d3crd__.fasta

./group53/reference/d1ngr__-d3ygsp_.fasta

./group54/reference/d1iqpa1-d1jr3a1.fasta

./group54/reference/d1iqpa1-d1jr3d1.fasta

./group54/reference/d1jr3a1-d1jr3d1.fasta

./group56/reference/d1agre_-d1iapa_.fasta

./group56/reference/d1agre_-d1omwa1.fasta

./group56/reference/d1cmza_-d1iapa_.fasta

./group56/reference/d1cmza_-d1omwa1.fasta

./group57/reference/d1aru__-d1jdra_.fasta

./group57/reference/d1aru__-d1mwva1.fasta

./group57/reference/d1bgp__-d1llp__.fasta

./group57/reference/d1bgp__-d1mn2__.fasta

./group57/reference/d1bgp__-d1mwva1.fasta

./group57/reference/d1bgp__-d1mwva2.fasta

./group57/reference/d1cvua1-d1mwva2.fasta

./group57/reference/d1jdra_-d1llp__.fasta

./group57/reference/d1llp__-d1mwva2.fasta

./group57/reference/d1mn2__-d1mwva2.fasta

./group58/reference/d1keaa_-d1mpga1.fasta

./group58/reference/d1keaa_-d1nkua_.fasta

./group58/reference/d1ko9a1-d1mun__.fasta

./group58/reference/d1ko9a1-d1ngna_.fasta

./group58/reference/d1ko9a1-d1nkua_.fasta

./group58/reference/d1ko9a1-d1orna_.fasta

./group58/reference/d1mpga1-d1mun__.fasta

./group58/reference/d1mpga1-d1ngna_.fasta

./group58/reference/d1mpga1-d1nkua_.fasta

./group58/reference/d1mpga1-d1orna_.fasta

./group58/reference/d1mpga1-d2abk__.fasta

./group58/reference/d1mun__-d1nkua_.fasta

./group58/reference/d1mun__-d1orna_.fasta

./group58/reference/d1mun__-d2abk__.fasta

./group58/reference/d1ngna_-d1nkua_.fasta

./group58/reference/d1ngna_-d2abk__.fasta

./group58/reference/d1nkua_-d2abk__.fasta

./group60/reference/d1dlja1-d1ks9a1.fasta

./group60/reference/d1ks9a1-d1mv8a1.fasta

./group60/reference/d1ks9a1-d1n1ea1.fasta

./group60/reference/d1ks9a1-d2pgd_1.fasta

./group60/reference/d1mv8a1-d1pgja1.fasta

./group60/reference/d1mv8a1-d2pgd_1.fasta

./group60/reference/d1n1ea1-d2pgd_1.fasta

./group61/reference/d1ayx__-d1clc_1.fasta

./group61/reference/d1ayx__-d1g9ga_.fasta

./group61/reference/d1ayx__-d1h54a1.fasta

./group61/reference/d1ayx__-d1ks8a_.fasta

./group61/reference/d1ayx__-d1lf6a1.fasta

./group61/reference/d1clc_1-d1fp3a_.fasta

./group61/reference/d1clc_1-d1gai__.fasta

./group61/reference/d1clc_1-d1kwfa_.fasta

./group61/reference/d1clc_1-d1nc5a_.fasta

./group61/reference/d1fp3a_-d1g87a1.fasta

./group61/reference/d1fp3a_-d1g9ga_.fasta

./group61/reference/d1fp3a_-d1ks8a_.fasta

./group61/reference/d1fp3a_-d1lf6a1.fasta

./group61/reference/d1fp3a_-d1nc5a_.fasta

./group61/reference/d1g87a1-d1g9ga_.fasta

./group61/reference/d1g87a1-d1kwfa_.fasta

./group61/reference/d1g9ga_-d1ks8a_.fasta

./group61/reference/d1g9ga_-d1kwfa_.fasta

./group61/reference/d1gai__-d1lf6a1.fasta

./group61/reference/d1h54a1-d1ks8a_.fasta

./group61/reference/d1h54a1-d1kwfa_.fasta

./group61/reference/d1ks8a_-d1kwfa_.fasta

./group61/reference/d1kwfa_-d1nc5a_.fasta

./group62/reference/d1cb8a1-d1hn0a1.fasta

./group62/reference/d1cb8a1-d1j0ma1.fasta

./group62/reference/d1cb8a1-d1qaza_.fasta

./group62/reference/d1hn0a1-d1j0ma1.fasta

./group62/reference/d1hn0a1-d1n7oa1.fasta

./group63/reference/d1c3d__-d1dceb_.fasta

./group63/reference/d1c3d__-d1ld8b_.fasta

./group63/reference/d1c3d__-d2sqca2.fasta

./group63/reference/d1dceb_-d2sqca2.fasta

./group63/reference/d1ld8b_-d2sqca2.fasta

./group63/reference/d2sqca2-d5eau_1.fasta

./group65/reference/d1cpt__-d1izoa_.fasta

./group65/reference/d1cpt__-d1n6ba_.fasta

./group65/reference/d1cpt__-d1n97a_.fasta

./group65/reference/d1dz4a_-d1e9xa_.fasta

./group65/reference/d1dz4a_-d1izoa_.fasta

./group65/reference/d1dz4a_-d1n97a_.fasta

./group65/reference/d1e9xa_-d1io7a_.fasta

./group65/reference/d1e9xa_-d1izoa_.fasta

./group65/reference/d1io7a_-d1izoa_.fasta

./group65/reference/d1io7a_-d1n6ba_.fasta

./group65/reference/d1izoa_-d1jfba_.fasta

./group65/reference/d1izoa_-d1jipa_.fasta

./group65/reference/d1izoa_-d1n6ba_.fasta

./group65/reference/d1izoa_-d1n97a_.fasta

./group65/reference/d1jfba_-d1n6ba_.fasta

./group65/reference/d1jfba_-d1n97a_.fasta

./group67/reference/d1pbwa_-d1wer__.fasta

./group67/reference/d1tx4a_-d1wer__.fasta

./group68/reference/d1b3ua_-d1bpoa1.fasta

./group68/reference/d1b3ua_-d1n8va_.fasta

./group68/reference/d1bpoa1-d1h6ka1.fasta

./group68/reference/d1bpoa1-d1n8va_.fasta

./group68/reference/d1bpoa1-d1oxja2.fasta

./group68/reference/d1h6ka1-d1h6ka2.fasta

./group69/reference/d1bd8__-d1dcqa1.fasta

./group69/reference/d1bi7b_-d1dcqa1.fasta

./group69/reference/d1bi7b_-d1ycsb1.fasta

./group69/reference/d1dcqa1-d1ihba_.fasta

./group70/reference/d1a17__-d1hh8a_.fasta

./group70/reference/d1a17__-d1hz4a_.fasta

./group70/reference/d1a17__-d1iyga_.fasta

./group70/reference/d1elra_-d1hz4a_.fasta

./group70/reference/d1elra_-d1ihga1.fasta

./group70/reference/d1elra_-d1iyga_.fasta

./group70/reference/d1elwa_-d1hh8a_.fasta

./group70/reference/d1elwa_-d1ihga1.fasta

./group70/reference/d1elwa_-d1iyga_.fasta

./group70/reference/d1hh8a_-d1hz4a_.fasta

./group70/reference/d1hh8a_-d1iyga_.fasta

./group70/reference/d1hh8a_-d1kt1a1.fasta

./group70/reference/d1hz4a_-d1ihga1.fasta

./group70/reference/d1hz4a_-d1kt1a1.fasta

./group70/reference/d1ihga1-d1iyga_.fasta

./group70/reference/d1iyga_-d1kt1a1.fasta

./group71/reference/d1dvpa1-d1eyha_.fasta

./group72/reference/d1a28a_-d1fcya_.fasta

./group72/reference/d1a28a_-d1ie9a_.fasta

./group72/reference/d1a28a_-d1n83a_.fasta

./group72/reference/d1a28a_-d2prga_.fasta

./group72/reference/d1ie9a_-d1pk5a_.fasta

./group72/reference/d1kv6a_-d1n83a_.fasta

./group72/reference/d1pk5a_-d2prga_.fasta

./group73/reference/d1ah7__-d1ak0__.fasta

./group73/reference/d1ak0__-d1ca1_1.fasta

./group74/reference/d1kxpd2-d1kxpd3.fasta

./group74/reference/d1kxpd2-d1n5ua3.fasta

./group74/reference/d1kxpd3-d1n5ua1.fasta

./group74/reference/d1kxpd3-d1n5ua3.fasta

./group74/reference/d1n5ua1-d1n5ua3.fasta

./group74/reference/d1n5ua2-d1n5ua3.fasta

./group75/reference/d1hy0a_-d1jswa_.fasta

./group76/reference/d1aokb_-d1lwba_.fasta

./group76/reference/d1buna_-d1lwba_.fasta

./group76/reference/d1kvoa_-d1lwba_.fasta

./group76/reference/d1le6a_-d1lwba_.fasta

./group76/reference/d1lfja_-d1lwba_.fasta

./group76/reference/d1lwba_-d1mc2a_.fasta

./group76/reference/d1lwba_-d1psj__.fasta

./group77/reference/d19hca_-d1ft5a_.fasta

./group77/reference/d19hca_-d1kssa1.fasta

./group77/reference/d19hca_-d1qo8a1.fasta

./group77/reference/d1aqe__-d1kssa1.fasta

./group77/reference/d1aqe__-d1m1qa_.fasta

./group77/reference/d1aqe__-d1qo8a1.fasta

./group77/reference/d1dxrc_-d1fs7a_.fasta

./group77/reference/d1dxrc_-d1kssa1.fasta

./group77/reference/d1dxrc_-d3caoa_.fasta

./group77/reference/d1eysc_-d1fs7a_.fasta

./group77/reference/d1eysc_-d1gu6a_.fasta

./group77/reference/d1eysc_-d1kssa1.fasta

./group77/reference/d1eysc_-d2ctha_.fasta

./group77/reference/d1eysc_-d3caoa_.fasta

./group77/reference/d1ft5a_-d1wad__.fasta

./group77/reference/d1ft5a_-d3caoa_.fasta

./group77/reference/d1gyoa_-d1kssa1.fasta

./group77/reference/d1m1qa_-d1wad__.fasta

./group77/reference/d1m1qa_-d2cy3__.fasta

./group77/reference/d1m1qa_-d3cyr__.fasta

./group77/reference/d1qo8a1-d2ctha_.fasta

./group77/reference/d1qo8a1-d3cyr__.fasta

./group78/reference/d1akjd_-d1dqta_.fasta

./group78/reference/d1akjd_-d1eaja_.fasta

./group78/reference/d1akjd_-d1fo0a_.fasta

./group78/reference/d1akjd_-d1fo0b_.fasta

./group78/reference/d1akjd_-d1g9mh1.fasta

./group78/reference/d1akjd_-d1gsma2.fasta

./group78/reference/d1akjd_-d1gxea_.fasta

./group78/reference/d1akjd_-d1hxma1.fasta

./group78/reference/d1akjd_-d1mfa_2.fasta

./group78/reference/d1akjd_-d1mqkh_.fasta

./group78/reference/d1akjd_-d1ncwh1.fasta

./group78/reference/d1akjd_-d1ogad1.fasta

./group78/reference/d1akjd_-d1ogae1.fasta

./group78/reference/d1akjd_-d2f5bh1.fasta

./group78/reference/d1b88a_-d1gsma2.fasta

./group78/reference/d1b88a_-d1gxea_.fasta

./group78/reference/d1b88a_-d1jmaa_.fasta

./group78/reference/d1b88a_-d1nezg_.fasta

./group78/reference/d1dqta_-d1eaja_.fasta

./group78/reference/d1dqta_-d1fo0b_.fasta

./group78/reference/d1dqta_-d1gsma2.fasta

./group78/reference/d1dqta_-d1gxea_.fasta

./group78/reference/d1dqta_-d1hxmb1.fasta

./group78/reference/d1dqta_-d1ktke1.fasta

./group78/reference/d1dqta_-d1mfa_2.fasta

./group78/reference/d1dqta_-d1mqkh_.fasta

./group78/reference/d1dqta_-d1nezg_.fasta

./group78/reference/d1dqta_-d1nfdb1.fasta

./group78/reference/d1dqta_-d1ogae1.fasta

./group78/reference/d1dqta_-d2f5bh1.fasta

./group78/reference/d1eaja_-d1fo0a_.fasta

./group78/reference/d1eaja_-d1fo0b_.fasta

./group78/reference/d1eaja_-d1g9mh1.fasta

./group78/reference/d1eaja_-d1gsma2.fasta

./group78/reference/d1eaja_-d1h5ba_.fasta

./group78/reference/d1eaja_-d1hxmb1.fasta

./group78/reference/d1eaja_-d1jmaa_.fasta

./group78/reference/d1eaja_-d1ktke1.fasta

./group78/reference/d1eaja_-d1mfa_2.fasta

./group78/reference/d1eaja_-d1mqkh_.fasta

./group78/reference/d1eaja_-d1ncwh1.fasta

./group78/reference/d1eaja_-d1nezg_.fasta

./group78/reference/d1eaja_-d1ogad1.fasta

./group78/reference/d1eaja_-d1ogae1.fasta

./group78/reference/d1eaja_-d1tvda_.fasta

./group78/reference/d1eaja_-d2f5bh1.fasta

./group78/reference/d1eaja_-d2rhe__.fasta

./group78/reference/d1fo0a_-d1gxea_.fasta

./group78/reference/d1fo0a_-d1mqkh_.fasta

./group78/reference/d1fo0a_-d1nezg_.fasta

./group78/reference/d1fo0b_-d1g9mh1.fasta

./group78/reference/d1fo0b_-d1gsma2.fasta

./group78/reference/d1fo0b_-d1gxea_.fasta

./group78/reference/d1fo0b_-d1nezg_.fasta

./group78/reference/d1g9mh1-d1gxea_.fasta

./group78/reference/d1g9mh1-d1nezg_.fasta

./group78/reference/d1gsma2-d1h5ba_.fasta

./group78/reference/d1gsma2-d1hxmb1.fasta

./group78/reference/d1gsma2-d1mqkh_.fasta

./group78/reference/d1gsma2-d1ncwh1.fasta

./group78/reference/d1gsma2-d1nlbh1.fasta

./group78/reference/d1gsma2-d1ogae1.fasta

./group78/reference/d1gsma2-d1tvda_.fasta

./group78/reference/d1gsma2-d2f5bh1.fasta

./group78/reference/d1gsma2-d2rhe__.fasta

./group78/reference/d1gxea_-d1h5ba_.fasta

./group78/reference/d1gxea_-d1hxma1.fasta

./group78/reference/d1gxea_-d1hxmb1.fasta

./group78/reference/d1gxea_-d1jmaa_.fasta

./group78/reference/d1gxea_-d1ktke1.fasta

./group78/reference/d1gxea_-d1mfa_2.fasta

./group78/reference/d1gxea_-d1mqkh_.fasta

./group78/reference/d1gxea_-d1ncwh1.fasta

./group78/reference/d1gxea_-d1nezg_.fasta

./group78/reference/d1gxea_-d1nfdb1.fasta

./group78/reference/d1gxea_-d1nlbh1.fasta

./group78/reference/d1gxea_-d1ogad1.fasta

./group78/reference/d1gxea_-d1ogae1.fasta

./group78/reference/d1gxea_-d1tvda_.fasta

./group78/reference/d1gxea_-d2f5bh1.fasta

./group78/reference/d1gxea_-d2rhe__.fasta

./group78/reference/d1h5ba_-d1jmaa_.fasta

./group78/reference/d1h5ba_-d1nezg_.fasta

./group78/reference/d1hxma1-d1jmaa_.fasta

./group78/reference/d1hxma1-d1ktke1.fasta

./group78/reference/d1hxma1-d1mfa_2.fasta

./group78/reference/d1hxmb1-d1jmaa_.fasta

./group78/reference/d1hxmb1-d1nezg_.fasta

./group78/reference/d1jmaa_-d1ktke1.fasta

./group78/reference/d1jmaa_-d1mfa_2.fasta

./group78/reference/d1jmaa_-d1mqkh_.fasta

./group78/reference/d1jmaa_-d1ncwh1.fasta

./group78/reference/d1jmaa_-d1nezg_.fasta

./group78/reference/d1jmaa_-d1nfdb1.fasta

./group78/reference/d1jmaa_-d1ogae1.fasta

./group78/reference/d1jmaa_-d1tvda_.fasta

./group78/reference/d1ktke1-d1nezg_.fasta

./group78/reference/d1mfa_2-d1nezg_.fasta

./group78/reference/d1mfa_2-d1ogad1.fasta

./group78/reference/d1mqkh_-d1nezg_.fasta

./group78/reference/d1nezg_-d1nfdb1.fasta

./group78/reference/d1nezg_-d1nlbh1.fasta

./group78/reference/d1nezg_-d1ogad1.fasta

./group78/reference/d1nezg_-d1tvda_.fasta

./group78/reference/d1nezg_-d2f5bh1.fasta

./group79/reference/d1axib2-d1bqua1.fasta

./group79/reference/d1axib2-d1cd9b1.fasta

./group79/reference/d1axib2-d1cfb_1.fasta

./group79/reference/d1axib2-d1cfb_2.fasta

./group79/reference/d1axib2-d1eerb1.fasta

./group79/reference/d1axib2-d1f6fb1.fasta

./group79/reference/d1axib2-d1fnf_1.fasta

./group79/reference/d1axib2-d1fyhb1.fasta

./group79/reference/d1axib2-d1gh7a1.fasta

./group79/reference/d1axib2-d1lqsr1.fasta

./group79/reference/d1axib2-d1lqsr2.fasta

./group79/reference/d1axib2-d1lwra_.fasta

./group79/reference/d1axib2-d1n26a2.fasta

./group79/reference/d1axib2-d1n6va1.fasta

./group79/reference/d1axib2-d1n6va2.fasta

./group79/reference/d1axib2-d2fnba_.fasta

./group79/reference/d1bpv__-d1bqua1.fasta

./group79/reference/d1bpv__-d1cd9b1.fasta

./group79/reference/d1bpv__-d1cfb_2.fasta

./group79/reference/d1bpv__-d1eerb1.fasta

./group79/reference/d1bpv__-d1egja_.fasta

./group79/reference/d1bpv__-d1fnf_1.fasta

./group79/reference/d1bpv__-d1fyhb1.fasta

./group79/reference/d1bpv__-d1gh7a2.fasta

./group79/reference/d1bpv__-d1lqsr1.fasta

./group79/reference/d1bpv__-d1lqsr2.fasta

./group79/reference/d1bpv__-d1lwra_.fasta

./group79/reference/d1bpv__-d1n26a2.fasta

./group79/reference/d1bpv__-d1n26a3.fasta

./group79/reference/d1bpv__-d1n6va1.fasta

./group79/reference/d1bpv__-d1n6va2.fasta

./group79/reference/d1bqua1-d1cfb_2.fasta

./group79/reference/d1bqua1-d1eerb2.fasta

./group79/reference/d1bqua1-d1egja_.fasta

./group79/reference/d1bqua1-d1fnf_1.fasta

./group79/reference/d1bqua1-d1fyhb1.fasta

./group79/reference/d1bqua1-d1gh7a1.fasta

./group79/reference/d1bqua1-d1gh7a2.fasta

./group79/reference/d1bqua1-d1i1ra1.fasta

./group79/reference/d1bqua1-d1iarb1.fasta

./group79/reference/d1bqua1-d1n26a3.fasta

./group79/reference/d1bqua1-d1qg3a2.fasta

./group79/reference/d1cd9b1-d1cfb_1.fasta

./group79/reference/d1cd9b1-d1eerb1.fasta

./group79/reference/d1cd9b1-d1eerb2.fasta

./group79/reference/d1cd9b1-d1egja_.fasta

./group79/reference/d1cd9b1-d1fnf_1.fasta

./group79/reference/d1cd9b1-d1fyhb1.fasta

./group79/reference/d1cd9b1-d1gh7a1.fasta

./group79/reference/d1cd9b1-d1gh7a2.fasta

./group79/reference/d1cd9b1-d1iarb1.fasta

./group79/reference/d1cd9b1-d1lqsr1.fasta

./group79/reference/d1cd9b1-d1lwra_.fasta

./group79/reference/d1cd9b1-d1n26a2.fasta

./group79/reference/d1cd9b1-d1n26a3.fasta

./group79/reference/d1cd9b1-d1n6va2.fasta

./group79/reference/d1cd9b1-d1qg3a2.fasta

./group79/reference/d1cd9b1-d2fnba_.fasta

./group79/reference/d1cfb_1-d1cfb_2.fasta

./group79/reference/d1cfb_1-d1eerb1.fasta

./group79/reference/d1cfb_1-d1eerb2.fasta

./group79/reference/d1cfb_1-d1f6fb1.fasta

./group79/reference/d1cfb_1-d1fnf_1.fasta

./group79/reference/d1cfb_1-d1fyhb1.fasta

./group79/reference/d1cfb_1-d1gh7a1.fasta

./group79/reference/d1cfb_1-d1gh7a2.fasta

./group79/reference/d1cfb_1-d1lqsr1.fasta

./group79/reference/d1cfb_1-d1n26a2.fasta

./group79/reference/d1cfb_1-d1n26a3.fasta

./group79/reference/d1cfb_1-d1n6va1.fasta

./group79/reference/d1cfb_1-d1n6va2.fasta

./group79/reference/d1cfb_1-d1qg3a2.fasta

./group79/reference/d1cfb_1-d2fnba_.fasta

./group79/reference/d1cfb_2-d1eerb1.fasta

./group79/reference/d1cfb_2-d1eerb2.fasta

./group79/reference/d1cfb_2-d1f6fb1.fasta

./group79/reference/d1cfb_2-d1fnf_1.fasta

./group79/reference/d1cfb_2-d1fyhb1.fasta

./group79/reference/d1cfb_2-d1gh7a2.fasta

./group79/reference/d1cfb_2-d1lqsr1.fasta

./group79/reference/d1cfb_2-d1lwra_.fasta

./group79/reference/d1cfb_2-d1n26a2.fasta

./group79/reference/d1cfb_2-d1n26a3.fasta

./group79/reference/d1cfb_2-d1n6va1.fasta

./group79/reference/d1cfb_2-d1qg3a2.fasta

./group79/reference/d1cfb_2-d2fnba_.fasta

./group79/reference/d1eerb1-d1eerb2.fasta

./group79/reference/d1eerb1-d1egja_.fasta

./group79/reference/d1eerb1-d1fnf_1.fasta

./group79/reference/d1eerb1-d1fyhb1.fasta

./group79/reference/d1eerb1-d1gh7a1.fasta

./group79/reference/d1eerb1-d1gh7a2.fasta

./group79/reference/d1eerb1-d1iarb1.fasta

./group79/reference/d1eerb1-d1lqsr1.fasta

./group79/reference/d1eerb1-d1lwra_.fasta

./group79/reference/d1eerb1-d1n26a2.fasta

./group79/reference/d1eerb1-d1n26a3.fasta

./group79/reference/d1eerb1-d1n6va1.fasta

./group79/reference/d1eerb1-d1n6va2.fasta

./group79/reference/d1eerb1-d1qg3a2.fasta

./group79/reference/d1eerb2-d1fnf_1.fasta

./group79/reference/d1eerb2-d1fyhb1.fasta

./group79/reference/d1eerb2-d1gh7a1.fasta

./group79/reference/d1eerb2-d1iarb1.fasta

./group79/reference/d1eerb2-d1lqsr2.fasta

./group79/reference/d1eerb2-d1lwra_.fasta

./group79/reference/d1eerb2-d1n26a2.fasta

./group79/reference/d1eerb2-d1n6va1.fasta

./group79/reference/d1eerb2-d1n6va2.fasta

./group79/reference/d1eerb2-d2fnba_.fasta

./group79/reference/d1egja_-d1f6fb1.fasta

./group79/reference/d1egja_-d1fnf_1.fasta

./group79/reference/d1egja_-d1fyhb1.fasta

./group79/reference/d1egja_-d1gh7a1.fasta

./group79/reference/d1egja_-d1iarb1.fasta

./group79/reference/d1egja_-d1lqsr1.fasta

./group79/reference/d1egja_-d1lqsr2.fasta

./group79/reference/d1egja_-d1lwra_.fasta

./group79/reference/d1egja_-d1n26a2.fasta

./group79/reference/d1egja_-d1n6va1.fasta

./group79/reference/d1egja_-d1n6va2.fasta

./group79/reference/d1egja_-d1qg3a2.fasta

./group79/reference/d1egja_-d2fnba_.fasta

./group79/reference/d1f6fb1-d1fnf_1.fasta

./group79/reference/d1f6fb1-d1gh7a1.fasta

./group79/reference/d1f6fb1-d1gh7a2.fasta

./group79/reference/d1f6fb1-d1i1ra1.fasta

./group79/reference/d1f6fb1-d1iarb1.fasta

./group79/reference/d1f6fb1-d1lqsr1.fasta

./group79/reference/d1f6fb1-d1lqsr2.fasta

./group79/reference/d1f6fb1-d1lwra_.fasta

./group79/reference/d1f6fb1-d1n26a2.fasta

./group79/reference/d1f6fb1-d1n26a3.fasta

./group79/reference/d1f6fb1-d1n6va1.fasta

./group79/reference/d1f6fb1-d1qg3a2.fasta

./group79/reference/d1f6fb1-d2fnba_.fasta

./group79/reference/d1fnf_1-d1fyhb1.fasta

./group79/reference/d1fnf_1-d1gh7a1.fasta

./group79/reference/d1fnf_1-d1gh7a2.fasta

./group79/reference/d1fnf_1-d1i1ra1.fasta

./group79/reference/d1fnf_1-d1lqsr1.fasta

./group79/reference/d1fnf_1-d1lqsr2.fasta

./group79/reference/d1fnf_1-d1lwra_.fasta

./group79/reference/d1fnf_1-d1n26a2.fasta

./group79/reference/d1fnf_1-d1n26a3.fasta

./group79/reference/d1fnf_1-d1n6va1.fasta

./group79/reference/d1fnf_1-d1n6va2.fasta

./group79/reference/d1fnf_1-d1qg3a2.fasta

./group79/reference/d1fyhb1-d1gh7a2.fasta

./group79/reference/d1fyhb1-d1iarb1.fasta

./group79/reference/d1fyhb1-d1lqsr1.fasta

./group79/reference/d1fyhb1-d1lqsr2.fasta

./group79/reference/d1fyhb1-d1lwra_.fasta

./group79/reference/d1fyhb1-d1n26a2.fasta

./group79/reference/d1fyhb1-d1n26a3.fasta

./group79/reference/d1fyhb1-d1n6va1.fasta

./group79/reference/d1fyhb1-d1qg3a2.fasta

./group79/reference/d1fyhb1-d2fnba_.fasta

./group79/reference/d1gh7a1-d1gh7a2.fasta

./group79/reference/d1gh7a1-d1iarb1.fasta

./group79/reference/d1gh7a1-d1n26a2.fasta

./group79/reference/d1gh7a1-d1n26a3.fasta

./group79/reference/d1gh7a1-d1n6va1.fasta

./group79/reference/d1gh7a1-d1qg3a2.fasta

./group79/reference/d1gh7a1-d2fnba_.fasta

./group79/reference/d1gh7a2-d1lqsr2.fasta

./group79/reference/d1gh7a2-d1lwra_.fasta

./group79/reference/d1gh7a2-d1n26a2.fasta

./group79/reference/d1gh7a2-d1n26a3.fasta

./group79/reference/d1gh7a2-d1n6va1.fasta

./group79/reference/d1gh7a2-d1n6va2.fasta

./group79/reference/d1gh7a2-d2fnba_.fasta

./group79/reference/d1i1ra1-d1iarb1.fasta

./group79/reference/d1i1ra1-d1n26a2.fasta

./group79/reference/d1i1ra1-d1n6va1.fasta

./group79/reference/d1i1ra1-d2fnba_.fasta

./group79/reference/d1iarb1-d1n26a2.fasta

./group79/reference/d1iarb1-d1n26a3.fasta

./group79/reference/d1iarb1-d1n6va1.fasta

./group79/reference/d1iarb1-d1n6va2.fasta

./group79/reference/d1lqsr1-d1lqsr2.fasta

./group79/reference/d1lqsr1-d1lwra_.fasta

./group79/reference/d1lqsr1-d1n6va1.fasta

./group79/reference/d1lqsr1-d1qg3a2.fasta

./group79/reference/d1lqsr2-d1lwra_.fasta

./group79/reference/d1lqsr2-d1n26a3.fasta

./group79/reference/d1lqsr2-d1n6va2.fasta

./group79/reference/d1lqsr2-d1qg3a2.fasta

./group79/reference/d1lwra_-d1n26a2.fasta

./group79/reference/d1lwra_-d1n26a3.fasta

./group79/reference/d1lwra_-d1n6va2.fasta

./group79/reference/d1lwra_-d1qg3a2.fasta

./group79/reference/d1lwra_-d2fnba_.fasta

./group79/reference/d1n26a2-d1n26a3.fasta

./group79/reference/d1n26a2-d1n6va1.fasta

./group79/reference/d1n26a2-d1n6va2.fasta

./group79/reference/d1n26a3-d1n6va1.fasta

./group79/reference/d1n26a3-d1qg3a2.fasta

./group79/reference/d1n6va1-d1qg3a2.fasta

./group79/reference/d1n6va2-d1qg3a2.fasta

./group79/reference/d1n6va2-d2fnba_.fasta

./group79/reference/d1qg3a2-d2fnba_.fasta

./group80/reference/d1bhga1-d1jz8a2.fasta

./group81/reference/d1f13a2-d1f13a3.fasta

./group81/reference/d1f13a2-d1g0da3.fasta

./group81/reference/d1f13a2-d1l9na2.fasta

./group81/reference/d1f13a2-d1l9na3.fasta

./group81/reference/d1f13a3-d1kv3a2.fasta

./group81/reference/d1f13a3-d1l9na2.fasta

./group81/reference/d1g0da3-d1kv3a2.fasta

./group81/reference/d1kv3a2-d1kv3a3.fasta

./group81/reference/d1kv3a2-d1l9na3.fasta

./group81/reference/d1kv3a3-d1l9na2.fasta

./group81/reference/d1l9na2-d1l9na3.fasta

./group82/reference/d1edha2-d1l3wa5.fasta

./group82/reference/d1l3wa4-d1l3wa5.fasta

./group84/reference/d1ej8a_-d1eso__.fasta

./group84/reference/d1ej8a_-d1oala_.fasta

./group85/reference/d1e42a1-d1gyva_.fasta

./group85/reference/d1e42a1-d1kyfa1.fasta

./group85/reference/d1e42a1-d1p4ua_.fasta

./group85/reference/d1gyva_-d1kyfa1.fasta

./group86/reference/d1cwva1-d1cwva3.fasta

./group86/reference/d1cwva1-d1cwva4.fasta

./group86/reference/d1cwva1-d1f00i2.fasta

./group86/reference/d1cwva2-d1cwva4.fasta

./group86/reference/d1cwva2-d1f00i2.fasta

./group86/reference/d1cwva3-d1cwva4.fasta

./group86/reference/d1cwva3-d1f00i1.fasta

./group86/reference/d1cwva3-d1f00i2.fasta

./group86/reference/d1cwva4-d1f00i1.fasta

./group86/reference/d1f00i1-d1f00i2.fasta

./group87/reference/d1aoha_-d1g43a_.fasta

./group87/reference/d1aoha_-d1nbca_.fasta

./group87/reference/d1aoha_-d1tf4a2.fasta

./group87/reference/d1e5ba_-d1g1ka_.fasta

./group87/reference/d1e5ba_-d1g43a_.fasta

./group87/reference/d1e5ba_-d1nbca_.fasta

./group87/reference/d1e5ba_-d1qba_2.fasta

./group87/reference/d1exh__-d1g1ka_.fasta

./group87/reference/d1exh__-d1nbca_.fasta

./group87/reference/d1exh__-d1qba_2.fasta

./group87/reference/d1exh__-d1tf4a2.fasta

./group87/reference/d1g1ka_-d1qba_2.fasta

./group87/reference/d1g43a_-d1qba_2.fasta

./group87/reference/d1nbca_-d1qba_2.fasta

./group88/reference/d1amx__-d1klfb2.fasta

./group88/reference/d1amx__-d1n67a1.fasta

./group88/reference/d1amx__-d1n67a2.fasta

./group88/reference/d1amx__-d1pdkb_.fasta

./group88/reference/d1klfb1-d1pdkb_.fasta

./group88/reference/d1klfb2-d1n67a1.fasta

./group88/reference/d1klfb2-d1p5vb_.fasta

./group88/reference/d1klfb2-d1pdkb_.fasta

./group88/reference/d1n67a1-d1n67a2.fasta

./group88/reference/d1n67a1-d1p5vb_.fasta

./group88/reference/d1n67a1-d1pdkb_.fasta

./group88/reference/d1p5vb_-d1pdkb_.fasta

./group89/reference/d1a02n2-d1bg1a2.fasta

./group89/reference/d1a02n2-d1h6fa_.fasta

./group89/reference/d1a3qa2-d1bg1a2.fasta

./group89/reference/d1bg1a2-d1bvoa_.fasta

./group89/reference/d1bg1a2-d1h6fa_.fasta

./group89/reference/d1bg1a2-d1imhc2.fasta

./group92/reference/d1aoza2-d1cyx__.fasta

./group92/reference/d1aoza2-d1fwxa1.fasta

./group92/reference/d1aoza2-d1gska3.fasta

./group92/reference/d1aoza2-d1gw0a1.fasta

./group92/reference/d1aoza2-d1gw0a3.fasta

./group92/reference/d1aoza2-d1hfua3.fasta

./group92/reference/d1aoza2-d1kbva2.fasta

./group92/reference/d1aoza2-d1kcw_1.fasta

./group92/reference/d1aoza2-d1kcw_4.fasta

./group92/reference/d1aoza2-d1kcw_6.fasta

./group92/reference/d1aoza2-d1kv7a1.fasta

./group92/reference/d1aoza2-d1ocrb1.fasta

./group92/reference/d1aoza2-d1oe1a2.fasta

./group92/reference/d1aoza2-d1qhqa_.fasta

./group92/reference/d1aoza3-d1cyx__.fasta

./group92/reference/d1aoza3-d1e30a_.fasta

./group92/reference/d1aoza3-d1gska2.fasta

./group92/reference/d1aoza3-d1gw0a2.fasta

./group92/reference/d1aoza3-d1ikop_.fasta

./group92/reference/d1aoza3-d1kbva1.fasta

./group92/reference/d1aoza3-d1kbva2.fasta

./group92/reference/d1aoza3-d1kcw_1.fasta

./group92/reference/d1aoza3-d1kcw_4.fasta

./group92/reference/d1aoza3-d1kcw_5.fasta

./group92/reference/d1aoza3-d1m56b1.fasta

./group92/reference/d1aoza3-d1ocrb1.fasta

./group92/reference/d1aoza3-d1oe1a2.fasta

./group92/reference/d1aoza3-d1qhqa_.fasta

./group92/reference/d1cyx__-d1fwxa1.fasta

./group92/reference/d1cyx__-d1gska3.fasta

./group92/reference/d1cyx__-d1gw0a1.fasta

./group92/reference/d1cyx__-d1gw0a3.fasta

./group92/reference/d1cyx__-d1hfua3.fasta

./group92/reference/d1cyx__-d1kbva2.fasta

./group92/reference/d1cyx__-d1kcw_1.fasta

./group92/reference/d1cyx__-d1kcw_2.fasta

./group92/reference/d1cyx__-d1kcw_4.fasta

./group92/reference/d1cyx__-d1kcw_6.fasta

./group92/reference/d1e30a_-d1gska2.fasta

./group92/reference/d1e30a_-d1gska3.fasta

./group92/reference/d1e30a_-d1gw0a3.fasta

./group92/reference/d1e30a_-d1hfua3.fasta

./group92/reference/d1e30a_-d1ikop_.fasta

./group92/reference/d1e30a_-d1kbva2.fasta

./group92/reference/d1e30a_-d1kcw_1.fasta

./group92/reference/d1e30a_-d1kcw_2.fasta

./group92/reference/d1e30a_-d1kcw_4.fasta

./group92/reference/d1e30a_-d1kcw_5.fasta

./group92/reference/d1e30a_-d1kcw_6.fasta

./group92/reference/d1e30a_-d1kv7a1.fasta

./group92/reference/d1e30a_-d1m56b1.fasta

./group92/reference/d1e30a_-d1ocrb1.fasta

./group92/reference/d1e30a_-d1oe1a2.fasta

./group92/reference/d1fwxa1-d1gska2.fasta

./group92/reference/d1fwxa1-d1gska3.fasta

./group92/reference/d1fwxa1-d1gw0a1.fasta

./group92/reference/d1fwxa1-d1gw0a2.fasta

./group92/reference/d1fwxa1-d1gw0a3.fasta

./group92/reference/d1fwxa1-d1hfua3.fasta

./group92/reference/d1fwxa1-d1ikop_.fasta

./group92/reference/d1fwxa1-d1kcw_1.fasta

./group92/reference/d1fwxa1-d1kcw_2.fasta

./group92/reference/d1fwxa1-d1kcw_4.fasta

./group92/reference/d1fwxa1-d1kcw_5.fasta

./group92/reference/d1fwxa1-d1kcw_6.fasta

./group92/reference/d1fwxa1-d1kv7a1.fasta

./group92/reference/d1fwxa1-d1ocrb1.fasta

./group92/reference/d1fwxa1-d1oe1a2.fasta

./group92/reference/d1gska2-d1gska3.fasta

./group92/reference/d1gska2-d1gw0a1.fasta

./group92/reference/d1gska2-d1gw0a3.fasta

./group92/reference/d1gska2-d1hfua3.fasta

./group92/reference/d1gska2-d1ikop_.fasta

./group92/reference/d1gska2-d1kbva1.fasta

./group92/reference/d1gska2-d1kbva2.fasta

./group92/reference/d1gska2-d1kcw_1.fasta

./group92/reference/d1gska2-d1kcw_2.fasta

./group92/reference/d1gska2-d1kcw_5.fasta

./group92/reference/d1gska2-d1kcw_6.fasta

./group92/reference/d1gska2-d1kv7a1.fasta

./group92/reference/d1gska2-d1m56b1.fasta

./group92/reference/d1gska2-d1oe1a2.fasta

./group92/reference/d1gska2-d1qhqa_.fasta

./group92/reference/d1gska3-d1gw0a1.fasta

./group92/reference/d1gska3-d1gw0a2.fasta

./group92/reference/d1gska3-d1hfua3.fasta

./group92/reference/d1gska3-d1kbva1.fasta

./group92/reference/d1gska3-d1kbva2.fasta

./group92/reference/d1gska3-d1kcw_1.fasta

./group92/reference/d1gska3-d1kcw_2.fasta

./group92/reference/d1gska3-d1kcw_4.fasta

./group92/reference/d1gska3-d1kcw_5.fasta

./group92/reference/d1gska3-d1kcw_6.fasta

./group92/reference/d1gska3-d1kv7a1.fasta

./group92/reference/d1gska3-d1m56b1.fasta

./group92/reference/d1gska3-d1ocrb1.fasta

./group92/reference/d1gska3-d1oe1a2.fasta

./group92/reference/d1gska3-d1qhqa_.fasta

./group92/reference/d1gw0a1-d1gw0a2.fasta

./group92/reference/d1gw0a1-d1gw0a3.fasta

./group92/reference/d1gw0a1-d1kbva2.fasta

./group92/reference/d1gw0a1-d1kcw_2.fasta

./group92/reference/d1gw0a1-d1kcw_4.fasta

./group92/reference/d1gw0a1-d1kv7a2.fasta

./group92/reference/d1gw0a1-d1m56b1.fasta

./group92/reference/d1gw0a1-d1qhqa_.fasta

./group92/reference/d1gw0a2-d1gw0a3.fasta

./group92/reference/d1gw0a2-d1hfua3.fasta

./group92/reference/d1gw0a2-d1ikop_.fasta

./group92/reference/d1gw0a2-d1kbva1.fasta

./group92/reference/d1gw0a2-d1kbva2.fasta

./group92/reference/d1gw0a2-d1kcw_2.fasta

./group92/reference/d1gw0a2-d1kv7a1.fasta

./group92/reference/d1gw0a2-d1oe1a2.fasta

./group92/reference/d1gw0a2-d1qhqa_.fasta

./group92/reference/d1gw0a3-d1kbva1.fasta

./group92/reference/d1gw0a3-d1kbva2.fasta

./group92/reference/d1gw0a3-d1kcw_1.fasta

./group92/reference/d1gw0a3-d1kcw_5.fasta

./group92/reference/d1gw0a3-d1m56b1.fasta

./group92/reference/d1gw0a3-d1ocrb1.fasta

./group92/reference/d1gw0a3-d1oe1a2.fasta

./group92/reference/d1hfua3-d1kbva2.fasta

./group92/reference/d1hfua3-d1kcw_1.fasta

./group92/reference/d1hfua3-d1kcw_4.fasta

./group92/reference/d1hfua3-d1kcw_5.fasta

./group92/reference/d1hfua3-d1kv7a1.fasta

./group92/reference/d1hfua3-d1kv7a2.fasta

./group92/reference/d1hfua3-d1m56b1.fasta

./group92/reference/d1hfua3-d1ocrb1.fasta

./group92/reference/d1hfua3-d1oe1a2.fasta

./group92/reference/d1hfua3-d1qhqa_.fasta

./group92/reference/d1ikop_-d1kbva1.fasta

./group92/reference/d1ikop_-d1kcw_1.fasta

./group92/reference/d1ikop_-d1kcw_5.fasta

./group92/reference/d1ikop_-d1kv7a1.fasta

./group92/reference/d1ikop_-d1oe1a2.fasta

./group92/reference/d1ikop_-d1qhqa_.fasta

./group92/reference/d1kbva1-d1kbva2.fasta

./group92/reference/d1kbva1-d1kcw_2.fasta

./group92/reference/d1kbva1-d1kcw_4.fasta

./group92/reference/d1kbva1-d1kv7a1.fasta

./group92/reference/d1kbva2-d1kcw_1.fasta

./group92/reference/d1kbva2-d1kcw_2.fasta

./group92/reference/d1kbva2-d1kcw_6.fasta

./group92/reference/d1kbva2-d1kv7a2.fasta

./group92/reference/d1kbva2-d1m56b1.fasta

./group92/reference/d1kbva2-d1ocrb1.fasta

./group92/reference/d1kbva2-d1qhqa_.fasta

./group92/reference/d1kcw_1-d1kcw_2.fasta

./group92/reference/d1kcw_1-d1kcw_4.fasta

./group92/reference/d1kcw_1-d1kcw_6.fasta

./group92/reference/d1kcw_1-d1kv7a2.fasta

./group92/reference/d1kcw_1-d1oe1a2.fasta

./group92/reference/d1kcw_1-d1qhqa_.fasta

./group92/reference/d1kcw_2-d1kcw_5.fasta

./group92/reference/d1kcw_2-d1kv7a1.fasta

./group92/reference/d1kcw_2-d1m56b1.fasta

./group92/reference/d1kcw_2-d1oe1a2.fasta

./group92/reference/d1kcw_2-d1qhqa_.fasta

./group92/reference/d1kcw_4-d1kcw_5.fasta

./group92/reference/d1kcw_4-d1kv7a1.fasta

./group92/reference/d1kcw_4-d1m56b1.fasta

./group92/reference/d1kcw_4-d1oe1a2.fasta

./group92/reference/d1kcw_4-d1qhqa_.fasta

./group92/reference/d1kcw_5-d1kcw_6.fasta

./group92/reference/d1kcw_5-d1kv7a1.fasta

./group92/reference/d1kcw_5-d1kv7a2.fasta

./group92/reference/d1kcw_5-d1qhqa_.fasta

./group92/reference/d1kcw_6-d1kv7a1.fasta

./group92/reference/d1kcw_6-d1m56b1.fasta

./group92/reference/d1kcw_6-d1ocrb1.fasta

./group92/reference/d1kcw_6-d1oe1a2.fasta

./group92/reference/d1kcw_6-d1qhqa_.fasta

./group92/reference/d1kv7a1-d1kv7a2.fasta

./group92/reference/d1kv7a1-d1m56b1.fasta

./group92/reference/d1kv7a1-d1ocrb1.fasta

./group92/reference/d1kv7a1-d1oe1a2.fasta

./group92/reference/d1kv7a1-d1qhqa_.fasta

./group92/reference/d1kv7a2-d1oe1a2.fasta

./group92/reference/d1kv7a2-d1qhqa_.fasta

./group92/reference/d1m56b1-d1oe1a2.fasta

./group92/reference/d1oe1a2-d1qhqa_.fasta

./group93/reference/d1dqva1-d1rlw__.fasta

./group93/reference/d1rlw__-d3rpba_.fasta

./group94/reference/d1k2fa_-d1lb6a_.fasta

./group95/reference/d1bhu__-d1h4ax1.fasta

./group95/reference/d1bhu__-d1h4ax2.fasta

./group95/reference/d1c01a_-d1f53a_.fasta

./group95/reference/d1c01a_-d1g6ea_.fasta

./group95/reference/d1c01a_-d1h4ax1.fasta

./group95/reference/d1f53a_-d1h4ax2.fasta

./group95/reference/d1f53a_-d1ha4a_.fasta

./group95/reference/d1f53a_-d2bb2_2.fasta

./group95/reference/d1g6ea_-d1h4ax1.fasta

./group95/reference/d1g6ea_-d1h4ax2.fasta

./group95/reference/d1g6ea_-d2bb2_2.fasta

./group97/reference/d1hx6a1-d1m3ya1.fasta

./group97/reference/d1hx6a1-d1m3ya2.fasta

./group97/reference/d1hx6a2-d1m3ya2.fasta

./group98/reference/d1ejfa_-d1gmea_.fasta

./group98/reference/d1ejfa_-d1shsa_.fasta

./group99/reference/d1bhga2-d1ciy_1.fasta

./group99/reference/d1bhga2-d1cx1a_.fasta

./group99/reference/d1bhga2-d1czsa_.fasta

./group99/reference/d1bhga2-d1gnya_.fasta

./group99/reference/d1bhga2-d1gu3a_.fasta

./group99/reference/d1bhga2-d1k42a_.fasta

./group99/reference/d1bhga2-d1kexa_.fasta

./group99/reference/d1bhga2-d1lnsa2.fasta

./group99/reference/d1bhga2-d1xnaa_.fasta

./group99/reference/d1ciy_1-d1gu3a_.fasta

./group99/reference/d1ciy_1-d1gwma_.fasta

./group99/reference/d1ciy_1-d1i5pa1.fasta

./group99/reference/d1ciy_1-d1ju3a1.fasta

./group99/reference/d1ciy_1-d1jz8a3.fasta

./group99/reference/d1ciy_1-d1k3ia2.fasta

./group99/reference/d1ciy_1-d1k42a_.fasta

./group99/reference/d1cx1a_-d1gnya_.fasta

./group99/reference/d1cx1a_-d1gwma_.fasta

./group99/reference/d1cx1a_-d1h6ya_.fasta

./group99/reference/d1cx1a_-d1jhja_.fasta

./group99/reference/d1cx1a_-d1ju3a1.fasta

./group99/reference/d1cx1a_-d1jz8a3.fasta

./group99/reference/d1cx1a_-d1k3ia2.fasta

./group99/reference/d1cx1a_-d1kgya_.fasta

./group99/reference/d1cx1a_-d1lnsa2.fasta

./group99/reference/d1cx1a_-d1of4a_.fasta

./group99/reference/d1cx1a_-d1xnaa_.fasta

./group99/reference/d1czsa_-d1dlc_1.fasta

./group99/reference/d1czsa_-d1eut_2.fasta

./group99/reference/d1czsa_-d1gnya_.fasta

./group99/reference/d1czsa_-d1jhja_.fasta

./group99/reference/d1czsa_-d1ji6a1.fasta

./group99/reference/d1czsa_-d1k12a_.fasta

./group99/reference/d1czsa_-d1k3ia2.fasta

./group99/reference/d1czsa_-d1kgya_.fasta

./group99/reference/d1czsa_-d1lnsa2.fasta

./group99/reference/d1czsa_-d1of4a_.fasta

./group99/reference/d1d7pm_-d1dlc_1.fasta

./group99/reference/d1d7pm_-d1h6ya_.fasta

./group99/reference/d1d7pm_-d1jhja_.fasta

./group99/reference/d1d7pm_-d1ji6a1.fasta

./group99/reference/d1d7pm_-d1jz8a3.fasta

./group99/reference/d1d7pm_-d1k12a_.fasta

./group99/reference/d1d7pm_-d1k3ia2.fasta

./group99/reference/d1d7pm_-d1kgya_.fasta

./group99/reference/d1dlc_1-d1h6ya_.fasta

./group99/reference/d1dlc_1-d1i5pa1.fasta

./group99/reference/d1dlc_1-d1jhja_.fasta

./group99/reference/d1dlc_1-d1jz8a3.fasta

./group99/reference/d1dlc_1-d1k42a_.fasta

./group99/reference/d1dlc_1-d1kexa_.fasta

./group99/reference/d1dlc_1-d1kgya_.fasta

./group99/reference/d1dlc_1-d1lnsa2.fasta

./group99/reference/d1eut_2-d1gu3a_.fasta

./group99/reference/d1eut_2-d1jz8a3.fasta

./group99/reference/d1eut_2-d1k12a_.fasta

./group99/reference/d1eut_2-d1k42a_.fasta

./group99/reference/d1eut_2-d1lnsa2.fasta

./group99/reference/d1eut_2-d1xnaa_.fasta

./group99/reference/d1gnya_-d1gu3a_.fasta

./group99/reference/d1gnya_-d1guia_.fasta

./group99/reference/d1gnya_-d1gwma_.fasta

./group99/reference/d1gnya_-d1h6ya_.fasta

./group99/reference/d1gnya_-d1ju3a1.fasta

./group99/reference/d1gnya_-d1k12a_.fasta

./group99/reference/d1gnya_-d1k42a_.fasta

./group99/reference/d1gnya_-d1kgya_.fasta

./group99/reference/d1gnya_-d1of4a_.fasta

./group99/reference/d1gu3a_-d1gwma_.fasta

./group99/reference/d1gu3a_-d1h6ya_.fasta

./group99/reference/d1gu3a_-d1jhja_.fasta

./group99/reference/d1gu3a_-d1kgya_.fasta

./group99/reference/d1gu3a_-d1lnsa2.fasta

./group99/reference/d1gu3a_-d1of4a_.fasta

./group99/reference/d1gu3a_-d1xnaa_.fasta

./group99/reference/d1guia_-d1gwma_.fasta

./group99/reference/d1guia_-d1h6ya_.fasta

./group99/reference/d1guia_-d1i5pa1.fasta

./group99/reference/d1guia_-d1jhja_.fasta

./group99/reference/d1guia_-d1ji6a1.fasta

./group99/reference/d1guia_-d1k12a_.fasta

./group99/reference/d1guia_-d1lnsa2.fasta

./group99/reference/d1gwma_-d1i5pa1.fasta

./group99/reference/d1gwma_-d1jz8a3.fasta

./group99/reference/d1gwma_-d1k42a_.fasta

./group99/reference/d1gwma_-d1of4a_.fasta

./group99/reference/d1gwma_-d1xnaa_.fasta

./group99/reference/d1h6ya_-d1i5pa1.fasta

./group99/reference/d1h6ya_-d1ju3a1.fasta

./group99/reference/d1h6ya_-d1k42a_.fasta

./group99/reference/d1h6ya_-d1kexa_.fasta

./group99/reference/d1h6ya_-d1kgya_.fasta

./group99/reference/d1i5pa1-d1jhja_.fasta

./group99/reference/d1i5pa1-d1ji6a1.fasta

./group99/reference/d1i5pa1-d1jz8a3.fasta

./group99/reference/d1i5pa1-d1k12a_.fasta

./group99/reference/d1i5pa1-d1kgya_.fasta

./group99/reference/d1i5pa1-d1of4a_.fasta

./group99/reference/d1i5pa1-d1xnaa_.fasta

./group99/reference/d1jhja_-d1k12a_.fasta

./group99/reference/d1jhja_-d1k3ia2.fasta

./group99/reference/d1jhja_-d1k42a_.fasta

./group99/reference/d1jhja_-d1kexa_.fasta

./group99/reference/d1jhja_-d1of4a_.fasta

./group99/reference/d1jhja_-d1xnaa_.fasta

./group99/reference/d1ji6a1-d1k12a_.fasta

./group99/reference/d1ji6a1-d1k42a_.fasta

./group99/reference/d1ji6a1-d1kexa_.fasta

./group99/reference/d1ji6a1-d1of4a_.fasta

./group99/reference/d1ju3a1-d1lnsa2.fasta

./group99/reference/d1jz8a3-d1k3ia2.fasta

./group99/reference/d1jz8a3-d1lnsa2.fasta

./group99/reference/d1jz8a3-d1of4a_.fasta

./group99/reference/d1k12a_-d1k3ia2.fasta

./group99/reference/d1k12a_-d1k42a_.fasta

./group99/reference/d1k12a_-d1kgya_.fasta

./group99/reference/d1k12a_-d1lnsa2.fasta

./group99/reference/d1k12a_-d1xnaa_.fasta

./group99/reference/d1k3ia2-d1kexa_.fasta

./group99/reference/d1k42a_-d1lnsa2.fasta

./group99/reference/d1k42a_-d1of4a_.fasta

./group99/reference/d1k42a_-d1xnaa_.fasta

./group99/reference/d1kexa_-d1xnaa_.fasta

./group99/reference/d1kgya_-d1of4a_.fasta

./group99/reference/d1lnsa2-d1xnaa_.fasta

./group100/reference/d1ahsa_-d1flca1.fasta

./group100/reference/d1ahsa_-d1jsda_.fasta

./group100/reference/d1bvp12-d1jsma_.fasta

./group100/reference/d1flca1-d1jsda_.fasta

./group100/reference/d1flca1-d1jsma_.fasta

./group100/reference/d1jsma_-d1qhda2.fasta

./group101/reference/d1h7za_-d1kkea1.fasta

./group101/reference/d1kaca_-d1kkea1.fasta

./group101/reference/d1kkea1-d1qhva_.fasta

./group102/reference/d1aly__-d1gr3a_.fasta

./group102/reference/d1gr3a_-d1kxga_.fasta

./group102/reference/d1gr3a_-d1tnra_.fasta

./group102/reference/d1gr3a_-d2tnfa_.fasta

./group103/reference/d1f1sa3-d1hn0a3.fasta

./group103/reference/d1f1sa3-d1j0ma2.fasta

./group104/reference/d1dmza_-d1gxca_.fasta

./group104/reference/d1dmza_-d1lgpa_.fasta

./group105/reference/d1a3k__-d1d2sa_.fasta

./group105/reference/d1a3k__-d1dyka1.fasta

./group105/reference/d1a3k__-d1gzca_.fasta

./group105/reference/d1a3k__-d1n1ta1.fasta

./group105/reference/d1a3k__-d1n3oa_.fasta

./group105/reference/d1a3k__-d1nls__.fasta

./group105/reference/d1a3k__-d1saca_.fasta

./group105/reference/d1a3k__-d2ayh__.fasta

./group105/reference/d1a3k__-d2nlra_.fasta

./group105/reference/d1a3k__-d2pela_.fasta

./group105/reference/d1a8d_1-d1d2sa_.fasta

./group105/reference/d1a8d_1-d1kit_2.fasta

./group105/reference/d1a8d_1-d1n1ta1.fasta

./group105/reference/d1a8d_1-d1n3oa_.fasta

./group105/reference/d1a8d_1-d1nls__.fasta

./group105/reference/d1a8d_1-d1saca_.fasta

./group105/reference/d1a8d_1-d2ayh__.fasta

./group105/reference/d1a8d_1-d2pela_.fasta

./group105/reference/d1a8d_1-d2sli_1.fasta

./group105/reference/d1bk1__-d1kit_1.fasta

./group105/reference/d1bk1__-d1n1ta1.fasta

./group105/reference/d1bk1__-d2nlra_.fasta

./group105/reference/d1bkza_-d1d2sa_.fasta

./group105/reference/d1bkza_-d1dyka1.fasta

./group105/reference/d1bkza_-d1dyka2.fasta

./group105/reference/d1bkza_-d1gzca_.fasta

./group105/reference/d1bkza_-d1kit_1.fasta

./group105/reference/d1bkza_-d1nls__.fasta

./group105/reference/d1bkza_-d2ayh__.fasta

./group105/reference/d1bkza_-d2pela_.fasta

./group105/reference/d1c4ra_-d1d2sa_.fasta

./group105/reference/d1c4ra_-d1dyka1.fasta

./group105/reference/d1c4ra_-d1dyka2.fasta

./group105/reference/d1c4ra_-d1fx5a_.fasta

./group105/reference/d1c4ra_-d1kit_1.fasta

./group105/reference/d1c4ra_-d1kit_2.fasta

./group105/reference/d1c4ra_-d1n1ta1.fasta

./group105/reference/d1c4ra_-d1n3oa_.fasta

./group105/reference/d1c4ra_-d1saca_.fasta

./group105/reference/d1c4ra_-d2sli_1.fasta

./group105/reference/d1d2sa_-d1epwa1.fasta

./group105/reference/d1d2sa_-d1g86a_.fasta

./group105/reference/d1d2sa_-d1kit_1.fasta

./group105/reference/d1d2sa_-d1kit_2.fasta

./group105/reference/d1d2sa_-d1n1ta1.fasta

./group105/reference/d1d2sa_-d1n3oa_.fasta

./group105/reference/d1d2sa_-d1nls__.fasta

./group105/reference/d1d2sa_-d2pela_.fasta

./group105/reference/d1d2sa_-d2sli_1.fasta

./group105/reference/d1d2sa_-d3btaa1.fasta

./group105/reference/d1dyka1-d1epwa1.fasta

./group105/reference/d1dyka1-d1fx5a_.fasta

./group105/reference/d1dyka1-d1g86a_.fasta

./group105/reference/d1dyka1-d1gzca_.fasta

./group105/reference/d1dyka1-d1kit_1.fasta

./group105/reference/d1dyka1-d1kit_2.fasta

./group105/reference/d1dyka1-d1n1ta1.fasta

./group105/reference/d1dyka1-d1saca_.fasta

./group105/reference/d1dyka1-d2ayh__.fasta

./group105/reference/d1dyka1-d2pela_.fasta

./group105/reference/d1dyka1-d2sli_1.fasta

./group105/reference/d1dyka2-d1epwa1.fasta

./group105/reference/d1dyka2-d1fx5a_.fasta

./group105/reference/d1dyka2-d1gzca_.fasta

./group105/reference/d1dyka2-d1kit_2.fasta

./group105/reference/d1dyka2-d1n1ta1.fasta

./group105/reference/d1dyka2-d1n3oa_.fasta

./group105/reference/d1dyka2-d1nls__.fasta

./group105/reference/d1dyka2-d1saca_.fasta

./group105/reference/d1dyka2-d2ayh__.fasta

./group105/reference/d1dyka2-d2pela_.fasta

./group105/reference/d1dyka2-d2sli_1.fasta

./group105/reference/d1epwa1-d1g86a_.fasta

./group105/reference/d1epwa1-d1n1ta1.fasta

./group105/reference/d1epwa1-d1n3oa_.fasta

./group105/reference/d1epwa1-d1saca_.fasta

./group105/reference/d1epwa1-d2ayh__.fasta

./group105/reference/d1epwa1-d2nlra_.fasta

./group105/reference/d1epwa1-d2pela_.fasta

./group105/reference/d1fx5a_-d1kit_2.fasta

./group105/reference/d1fx5a_-d1saca_.fasta

./group105/reference/d1fx5a_-d1xnb__.fasta

./group105/reference/d1fx5a_-d2ayh__.fasta

./group105/reference/d1fx5a_-d2nlra_.fasta

./group105/reference/d1fx5a_-d2sli_1.fasta

./group105/reference/d1fx5a_-d3btaa1.fasta

./group105/reference/d1g86a_-d1kit_2.fasta

./group105/reference/d1g86a_-d1xnb__.fasta

./group105/reference/d1g86a_-d2sli_1.fasta

./group105/reference/d1gzca_-d1kqra_.fasta

./group105/reference/d1gzca_-d1n1ta1.fasta

./group105/reference/d1gzca_-d1xnb__.fasta

./group105/reference/d1gzca_-d2ayh__.fasta

./group105/reference/d1gzca_-d2sli_1.fasta

./group105/reference/d1kit_1-d2ayh__.fasta

./group105/reference/d1kit_1-d2pela_.fasta

./group105/reference/d1kit_1-d2sli_1.fasta

./group105/reference/d1kit_2-d1n1ta1.fasta

./group105/reference/d1kit_2-d2ayh__.fasta

./group105/reference/d1kit_2-d2nlra_.fasta

./group105/reference/d1kit_2-d2sli_1.fasta

./group105/reference/d1kit_2-d3btaa1.fasta

./group105/reference/d1kqra_-d1nls__.fasta

./group105/reference/d1kqra_-d2ayh__.fasta

./group105/reference/d1kqra_-d3btaa1.fasta

./group105/reference/d1n1ta1-d1saca_.fasta

./group105/reference/d1n1ta1-d1xnb__.fasta

./group105/reference/d1n1ta1-d2sli_1.fasta

./group105/reference/d1n3oa_-d2ayh__.fasta

./group105/reference/d1n3oa_-d2nlra_.fasta

./group105/reference/d1n3oa_-d3btaa1.fasta

./group105/reference/d1nls__-d1saca_.fasta

./group105/reference/d1nls__-d1xnb__.fasta

./group105/reference/d1nls__-d2sli_1.fasta

./group105/reference/d1saca_-d1xnb__.fasta

./group105/reference/d1saca_-d2pela_.fasta

./group105/reference/d1saca_-d2sli_1.fasta

./group105/reference/d1saca_-d3btaa1.fasta

./group105/reference/d1xnb__-d2nlra_.fasta

./group105/reference/d1xnb__-d2pela_.fasta

./group105/reference/d2ayh__-d2nlra_.fasta

./group105/reference/d2ayh__-d2pela_.fasta

./group105/reference/d2ayh__-d2sli_1.fasta

./group105/reference/d2nlra_-d2pela_.fasta

./group105/reference/d2pela_-d3btaa1.fasta

./group106/reference/d1fqta_-d1g8kb_.fasta

./group106/reference/d1fqta_-d1o7na1.fasta

./group106/reference/d1fqta_-d1rfs__.fasta

./group106/reference/d1fqta_-d1rie__.fasta

./group106/reference/d1g8kb_-d1o7na1.fasta

./group106/reference/d1g8kb_-d1rie__.fasta

./group106/reference/d1nyka_-d1o7na1.fasta

./group106/reference/d1o7na1-d1rie__.fasta

./group107/reference/d1bia_2-d1igqa_.fasta

./group107/reference/d1fx7a3-d1igqa_.fasta

./group108/reference/d1awj__-d1gcqc_.fasta

./group108/reference/d1awj__-d1i07a_.fasta

./group108/reference/d1awj__-d1neb__.fasta

./group108/reference/d1bbza_-d1gcqc_.fasta

./group108/reference/d1bbza_-d1ng2a1.fasta

./group108/reference/d1gcqc_-d1h92a_.fasta

./group108/reference/d1gcqc_-d1i07a_.fasta

./group108/reference/d1gcqc_-d1jqqa_.fasta

./group108/reference/d1gcqc_-d1neb__.fasta

./group108/reference/d1gcqc_-d1pwt__.fasta

./group108/reference/d1h92a_-d1i07a_.fasta

./group108/reference/d1h92a_-d1i1ja_.fasta

./group108/reference/d1h92a_-d1ng2a2.fasta

./group108/reference/d1i07a_-d1i1ja_.fasta

./group108/reference/d1i07a_-d1k4us_.fasta

./group108/reference/d1i07a_-d1ng2a2.fasta

./group108/reference/d1i07a_-d1pht__.fasta

./group108/reference/d1i07a_-d2hsp__.fasta

./group108/reference/d1i1ja_-d1ycsb2.fasta

./group108/reference/d1ng2a1-d2hsp__.fasta

./group109/reference/d1jb0e_-d2ahjb_.fasta

./group110/reference/d1jj2a1-d1jj2p_.fasta

./group110/reference/d1jj2a1-d1khia1.fasta

./group110/reference/d1jj2a1-d1m1ga2.fasta

./group110/reference/d1jj2p_-d1jj2s_.fasta

./group110/reference/d1jj2p_-d1khia1.fasta

./group110/reference/d1jj2p_-d2eifa1.fasta

./group110/reference/d1jj2s_-d1khia1.fasta

./group110/reference/d1jj2s_-d2eifa1.fasta

./group110/reference/d1khia1-d1m1ga2.fasta

./group110/reference/d1m1ga2-d2eifa1.fasta

./group111/reference/d1aono_-d1g31a_.fasta

./group111/reference/d1g31a_-d1p3ha_.fasta

./group112/reference/d1be9a_-d1k32a1.fasta

./group112/reference/d1d5ga_-d1k32a1.fasta

./group112/reference/d1d5ga_-d1lcya1.fasta

./group112/reference/d1d5ga_-d1ntea_.fasta

./group112/reference/d1g9oa_-d1k32a1.fasta

./group112/reference/d1ihja_-d1k32a1.fasta

./group112/reference/d1ihja_-d1qaua_.fasta

./group112/reference/d1k32a1-d1lcya1.fasta

./group112/reference/d1k32a1-d1mfga_.fasta

./group112/reference/d1k32a1-d1qaua_.fasta

./group112/reference/d1k32a1-d1qava_.fasta

./group112/reference/d1k32a1-d1qlca_.fasta

./group112/reference/d1kwaa_-d1m5za_.fasta

./group112/reference/d1kwaa_-d1qaua_.fasta

./group112/reference/d1lcya1-d1m5za_.fasta

./group112/reference/d1lcya1-d1nf3c_.fasta

./group112/reference/d1lcya1-d1qava_.fasta

./group112/reference/d1m5za_-d1ntea_.fasta

./group112/reference/d1m5za_-d1qaua_.fasta

./group112/reference/d1ntea_-d1qaua_.fasta

./group112/reference/d1ntea_-d1qava_.fasta

./group112/reference/d1ntea_-d1qlca_.fasta

./group113/reference/d1b34a_-d1i8fa_.fasta

./group113/reference/d1d3ba_-d1d3bb_.fasta

./group113/reference/d1d3ba_-d1i8fa_.fasta

./group113/reference/d1d3ba_-d1kq1a_.fasta

./group113/reference/d1d3ba_-d1mxma1.fasta

./group113/reference/d1d3ba_-d1n9ra_.fasta

./group113/reference/d1d3bb_-d1kq1a_.fasta

./group113/reference/d1d3bb_-d1mxma1.fasta

./group113/reference/d1h641_-d1kq1a_.fasta

./group113/reference/d1h641_-d1mxma1.fasta

./group113/reference/d1i8fa_-d1kq1a_.fasta

./group113/reference/d1i8fa_-d1mxma1.fasta

./group113/reference/d1kq1a_-d1ljoa_.fasta

./group113/reference/d1kq1a_-d1n9ra_.fasta

./group113/reference/d1ljoa_-d1mxma1.fasta

./group113/reference/d1mgqa_-d1mxma1.fasta

./group113/reference/d1mxma1-d1n9ra_.fasta

./group114/reference/d1an8_1-d1prtb1.fasta

./group114/reference/d1an8_1-d3tss_1.fasta

./group114/reference/d1c4qa_-d1enfa1.fasta

./group114/reference/d1c4qa_-d1fnua1.fasta

./group114/reference/d1c4qa_-d1prtf_.fasta

./group114/reference/d1c4qa_-d1qb5d_.fasta

./group114/reference/d1c4qa_-d3tss_1.fasta

./group114/reference/d1enfa1-d1eu3a1.fasta

./group114/reference/d1enfa1-d3chbd_.fasta

./group114/reference/d1enfa1-d3tss_1.fasta

./group114/reference/d1eu3a1-d1prtb1.fasta

./group114/reference/d1eu3a1-d1prtd_.fasta

./group114/reference/d1eu3a1-d3chbd_.fasta

./group114/reference/d1eu3a1-d3seb_1.fasta

./group114/reference/d1eu3a1-d3tss_1.fasta

./group114/reference/d1fnua1-d1prtb1.fasta

./group114/reference/d1fnua1-d3tss_1.fasta

./group114/reference/d1prtb1-d1prtd_.fasta

./group114/reference/d1prtb1-d1prtf_.fasta

./group114/reference/d1prtb1-d1qb5d_.fasta

./group114/reference/d1prtb1-d3chbd_.fasta

./group114/reference/d1prtd_-d1prtf_.fasta

./group114/reference/d1prtd_-d3chbd_.fasta

./group114/reference/d1prtd_-d3seb_1.fasta

./group114/reference/d1prtf_-d3chbd_.fasta

./group114/reference/d1prtf_-d3seb_1.fasta

./group114/reference/d1prtf_-d3tss_1.fasta

./group114/reference/d1qb5d_-d3chbd_.fasta

./group114/reference/d3seb_1-d3tss_1.fasta

./group115/reference/d1br9__-d1jb3a_.fasta

./group115/reference/d1br9__-d1uapa_.fasta

./group115/reference/d1jb3a_-d1uapa_.fasta

./group116/reference/d1c0aa1-d1ewia_.fasta

./group116/reference/d1c0aa1-d1fgua1.fasta

./group116/reference/d1c0aa1-d1fgua2.fasta

./group116/reference/d1c0aa1-d1fjgl_.fasta

./group116/reference/d1c0aa1-d1fl0a_.fasta

./group116/reference/d1c0aa1-d1gm5a2.fasta

./group116/reference/d1c0aa1-d1iyjb5.fasta

./group116/reference/d1c0aa1-d1jb7a1.fasta

./group116/reference/d1c0aa1-d1jb7a3.fasta

./group116/reference/d1c0aa1-d1jb7b_.fasta

./group116/reference/d1c0aa1-d1jjcb3.fasta

./group116/reference/d1c0aa1-d1ltla_.fasta

./group116/reference/d1c0aa1-d1o7ia_.fasta

./group116/reference/d1c0aa1-d1pxfa_.fasta

./group116/reference/d1c0aa1-d1quqb_.fasta

./group116/reference/d1c0aa1-d1qvca_.fasta

./group116/reference/d1d7qa_-d1eova1.fasta

./group116/reference/d1d7qa_-d1ewia_.fasta

./group116/reference/d1d7qa_-d1fgua1.fasta

./group116/reference/d1d7qa_-d1fjgq_.fasta

./group116/reference/d1d7qa_-d1gpc__.fasta

./group116/reference/d1d7qa_-d1jb7a1.fasta

./group116/reference/d1d7qa_-d1jjcb3.fasta

./group116/reference/d1d7qa_-d1ltla_.fasta

./group116/reference/d1d7qa_-d1qvca_.fasta

./group116/reference/d1e1oa1-d1fgua1.fasta

./group116/reference/d1e1oa1-d1fgua2.fasta

./group116/reference/d1e1oa1-d1fjgl_.fasta

./group116/reference/d1e1oa1-d1gd7a_.fasta

./group116/reference/d1e1oa1-d1gpc__.fasta

./group116/reference/d1e1oa1-d1iyjb5.fasta

./group116/reference/d1e1oa1-d1jb7a1.fasta

./group116/reference/d1e1oa1-d1jb7a2.fasta

./group116/reference/d1e1oa1-d1jb7b_.fasta

./group116/reference/d1e1oa1-d1kxla_.fasta

./group116/reference/d1e1oa1-d1ltla_.fasta

./group116/reference/d1e1oa1-d1o7ia_.fasta

./group116/reference/d1e1oa1-d1pxfa_.fasta

./group116/reference/d1e1oa1-d1quqb_.fasta

./group116/reference/d1e1oa1-d1qvca_.fasta

./group116/reference/d1eova1-d1ewia_.fasta

./group116/reference/d1eova1-d1gd7a_.fasta

./group116/reference/d1eova1-d1gm5a2.fasta

./group116/reference/d1eova1-d1iyjb5.fasta

./group116/reference/d1eova1-d1jb7a1.fasta

./group116/reference/d1eova1-d1jb7a3.fasta

./group116/reference/d1eova1-d1jjcb3.fasta

./group116/reference/d1eova1-d1kxla_.fasta

./group116/reference/d1eova1-d1o7ia_.fasta

./group116/reference/d1eova1-d1qvca_.fasta

./group116/reference/d1ewia_-d1fgua1.fasta

./group116/reference/d1ewia_-d1fgua2.fasta

./group116/reference/d1ewia_-d1fjgl_.fasta

./group116/reference/d1ewia_-d1fjgq_.fasta

./group116/reference/d1ewia_-d1fl0a_.fasta

./group116/reference/d1ewia_-d1gd7a_.fasta

./group116/reference/d1ewia_-d1gm5a2.fasta

./group116/reference/d1ewia_-d1iyjb5.fasta

./group116/reference/d1ewia_-d1jb7a1.fasta

./group116/reference/d1ewia_-d1jb7a2.fasta

./group116/reference/d1ewia_-d1jb7a3.fasta

./group116/reference/d1ewia_-d1jb7b_.fasta

./group116/reference/d1ewia_-d1jjcb3.fasta

./group116/reference/d1ewia_-d1kxla_.fasta

./group116/reference/d1ewia_-d1o7ia_.fasta

./group116/reference/d1ewia_-d1pxfa_.fasta

./group116/reference/d1ewia_-d1quqb_.fasta

./group116/reference/d1fgua1-d1fgua2.fasta

./group116/reference/d1fgua1-d1fjgq_.fasta

./group116/reference/d1fgua1-d1gd7a_.fasta

./group116/reference/d1fgua1-d1iyjb5.fasta

./group116/reference/d1fgua1-d1jb7a1.fasta

./group116/reference/d1fgua1-d1jb7a2.fasta

./group116/reference/d1fgua1-d1jb7a3.fasta

./group116/reference/d1fgua1-d1jb7b_.fasta

./group116/reference/d1fgua1-d1kxla_.fasta

./group116/reference/d1fgua1-d1ltla_.fasta

./group116/reference/d1fgua1-d1o7ia_.fasta

./group116/reference/d1fgua1-d1quqb_.fasta

./group116/reference/d1fgua1-d1qvca_.fasta

./group116/reference/d1fgua2-d1fjgq_.fasta

./group116/reference/d1fgua2-d1fl0a_.fasta

./group116/reference/d1fgua2-d1gm5a2.fasta

./group116/reference/d1fgua2-d1gpc__.fasta

./group116/reference/d1fgua2-d1iyjb5.fasta

./group116/reference/d1fgua2-d1jb7a1.fasta

./group116/reference/d1fgua2-d1jb7a2.fasta

./group116/reference/d1fgua2-d1jb7b_.fasta

./group116/reference/d1fgua2-d1jjcb3.fasta

./group116/reference/d1fgua2-d1kxla_.fasta

./group116/reference/d1fgua2-d1quqb_.fasta

./group116/reference/d1fgua2-d1qvca_.fasta

./group116/reference/d1fjgl_-d1fl0a_.fasta

./group116/reference/d1fjgl_-d1gm5a2.fasta

./group116/reference/d1fjgl_-d1jb7a2.fasta

./group116/reference/d1fjgl_-d1jb7a3.fasta

./group116/reference/d1fjgl_-d1jb7b_.fasta

./group116/reference/d1fjgl_-d1ltla_.fasta

./group116/reference/d1fjgl_-d1o7ia_.fasta

./group116/reference/d1fjgl_-d1pxfa_.fasta

./group116/reference/d1fjgl_-d1quqb_.fasta

./group116/reference/d1fjgq_-d1fl0a_.fasta

./group116/reference/d1fjgq_-d1gd7a_.fasta

./group116/reference/d1fjgq_-d1gm5a2.fasta

./group116/reference/d1fjgq_-d1iyjb5.fasta

./group116/reference/d1fjgq_-d1jb7a1.fasta

./group116/reference/d1fjgq_-d1jb7b_.fasta

./group116/reference/d1fjgq_-d1jjcb3.fasta

./group116/reference/d1fjgq_-d1kxla_.fasta

./group116/reference/d1fjgq_-d1ltla_.fasta

./group116/reference/d1fjgq_-d1o7ia_.fasta

./group116/reference/d1fjgq_-d1quqb_.fasta

./group116/reference/d1fl0a_-d1gpc__.fasta

./group116/reference/d1fl0a_-d1iyjb5.fasta

./group116/reference/d1fl0a_-d1jb7a3.fasta

./group116/reference/d1fl0a_-d1ltla_.fasta

./group116/reference/d1gd7a_-d1gm5a2.fasta

./group116/reference/d1gd7a_-d1gpc__.fasta

./group116/reference/d1gd7a_-d1jb7b_.fasta

./group116/reference/d1gd7a_-d1ltla_.fasta

./group116/reference/d1gd7a_-d1o7ia_.fasta

./group116/reference/d1gd7a_-d1quqb_.fasta

./group116/reference/d1gd7a_-d1qvca_.fasta

./group116/reference/d1gm5a2-d1iyjb5.fasta

./group116/reference/d1gm5a2-d1jb7a1.fasta

./group116/reference/d1gm5a2-d1jb7a2.fasta

./group116/reference/d1gm5a2-d1jb7a3.fasta

./group116/reference/d1gm5a2-d1jb7b_.fasta

./group116/reference/d1gm5a2-d1jjcb3.fasta

./group116/reference/d1gm5a2-d1ltla_.fasta

./group116/reference/d1gm5a2-d1o7ia_.fasta

./group116/reference/d1gm5a2-d1pxfa_.fasta

./group116/reference/d1gm5a2-d1quqb_.fasta

./group116/reference/d1gm5a2-d1qvca_.fasta

./group116/reference/d1gpc__-d1jb7a2.fasta

./group116/reference/d1gpc__-d1jb7b_.fasta

./group116/reference/d1gpc__-d1jjcb3.fasta

./group116/reference/d1gpc__-d1o7ia_.fasta

./group116/reference/d1gpc__-d1quqb_.fasta

./group116/reference/d1iyjb5-d1jb7a1.fasta

./group116/reference/d1iyjb5-d1jb7a2.fasta

./group116/reference/d1iyjb5-d1jb7b_.fasta

./group116/reference/d1iyjb5-d1jjcb3.fasta

./group116/reference/d1iyjb5-d1ltla_.fasta

./group116/reference/d1iyjb5-d1o7ia_.fasta

./group116/reference/d1iyjb5-d1pxfa_.fasta

./group116/reference/d1iyjb5-d1quqb_.fasta

./group116/reference/d1iyjb5-d1qvca_.fasta

./group116/reference/d1jb7a1-d1jb7a2.fasta

./group116/reference/d1jb7a1-d1jb7b_.fasta

./group116/reference/d1jb7a1-d1jjcb3.fasta

./group116/reference/d1jb7a1-d1o7ia_.fasta

./group116/reference/d1jb7a1-d1quqb_.fasta

./group116/reference/d1jb7a2-d1jb7a3.fasta

./group116/reference/d1jb7a2-d1jb7b_.fasta

./group116/reference/d1jb7a2-d1kxla_.fasta

./group116/reference/d1jb7a2-d1ltla_.fasta

./group116/reference/d1jb7a2-d1o7ia_.fasta

./group116/reference/d1jb7a2-d1pxfa_.fasta

./group116/reference/d1jb7a2-d1quqb_.fasta

./group116/reference/d1jb7a2-d1qvca_.fasta

./group116/reference/d1jb7a3-d1jb7b_.fasta

./group116/reference/d1jb7a3-d1jjcb3.fasta

./group116/reference/d1jb7a3-d1ltla_.fasta

./group116/reference/d1jb7a3-d1pxfa_.fasta

./group116/reference/d1jb7a3-d1quqb_.fasta

./group116/reference/d1jb7a3-d1qvca_.fasta

./group116/reference/d1jb7b_-d1kxla_.fasta

./group116/reference/d1jb7b_-d1o7ia_.fasta

./group116/reference/d1jb7b_-d1pxfa_.fasta

./group116/reference/d1jjcb3-d1ltla_.fasta

./group116/reference/d1jjcb3-d1quqb_.fasta

./group116/reference/d1jjcb3-d1qvca_.fasta

./group116/reference/d1kxla_-d1o7ia_.fasta

./group116/reference/d1kxla_-d1quqb_.fasta

./group116/reference/d1kxla_-d1qvca_.fasta

./group116/reference/d1ltla_-d1o7ia_.fasta

./group116/reference/d1o7ia_-d1quqb_.fasta

./group116/reference/d1o7ia_-d1qvca_.fasta

./group116/reference/d1pxfa_-d1quqb_.fasta

./group116/reference/d1pxfa_-d1qvca_.fasta

./group116/reference/d1quqb_-d1qvca_.fasta

./group118/reference/d1fr3a_-d1g2914.fasta

./group118/reference/d1fr3a_-d1oxsc1.fasta

./group118/reference/d1g2913-d1g2914.fasta

./group118/reference/d1g2913-d1h9ma1.fasta

./group118/reference/d1g2913-d1h9ma2.fasta

./group118/reference/d1g2913-d1h9ra2.fasta

./group118/reference/d1g2913-d1oxsc1.fasta

./group118/reference/d1g2914-d1h9ma1.fasta

./group118/reference/d1g2914-d1h9ma2.fasta

./group118/reference/d1g2914-d1h9ra1.fasta

./group118/reference/d1g2914-d1h9ra2.fasta

./group118/reference/d1g2914-d1oxsc1.fasta

./group118/reference/d1guta_-d1oxsc1.fasta

./group118/reference/d1h9ma1-d1oxsc1.fasta

./group118/reference/d1h9ma2-d1oxsc1.fasta

./group118/reference/d1h9ra2-d1oxsc1.fasta

./group119/reference/d1bfg__-d1ilr1_.fasta

./group119/reference/d1ijta_-d1ilr1_.fasta

./group119/reference/d1ilr1_-d1qqla_.fasta

./group120/reference/d1abrb2-d1m2tb1.fasta

./group120/reference/d1dqga_-d1ggpb1.fasta

./group120/reference/d1dqga_-d1hwmb1.fasta

./group120/reference/d1dqga_-d1hwmb2.fasta

./group120/reference/d1dqga_-d1m2tb1.fasta

./group121/reference/d1a8d_2-d1avac_.fasta

./group121/reference/d1a8d_2-d1avwb_.fasta

./group121/reference/d1a8d_2-d1eyla_.fasta

./group121/reference/d1a8d_2-d1wba__.fasta

./group121/reference/d1avac_-d1epwa2.fasta

./group121/reference/d1avac_-d3btaa2.fasta

./group121/reference/d1avwb_-d1epwa2.fasta

./group121/reference/d1avwb_-d3btaa2.fasta

./group121/reference/d1epwa2-d1eyla_.fasta

./group121/reference/d1epwa2-d1wba__.fasta

./group121/reference/d1eyla_-d3btaa2.fasta

./group121/reference/d1wba__-d3btaa2.fasta

./group122/reference/d1dfca2-d1hcd__.fasta

./group123/reference/d1dar_1-d1f60a1.fasta

./group123/reference/d1f60a1-d1n0ua1.fasta

./group125/reference/d1ci0a_-d1i0ra_.fasta

./group125/reference/d1ejea_-d1flma_.fasta

./group125/reference/d1ejea_-d1i0ra_.fasta

./group125/reference/d1flma_-d1i0ra_.fasta

./group126/reference/d1agja_-d1arb__.fasta

./group126/reference/d1agja_-d1azza_.fasta

./group126/reference/d1agja_-d1bio__.fasta

./group126/reference/d1agja_-d1bqya_.fasta

./group126/reference/d1agja_-d1cgha_.fasta

./group126/reference/d1agja_-d1ddja_.fasta

./group126/reference/d1agja_-d1eaxa_.fasta

./group126/reference/d1agja_-d1ekbb_.fasta

./group126/reference/d1agja_-d1eq9a_.fasta

./group126/reference/d1agja_-d1fjsa_.fasta

./group126/reference/d1agja_-d1gdna_.fasta

./group126/reference/d1agja_-d1gvkb_.fasta

./group126/reference/d1agja_-d1hj9a_.fasta

./group126/reference/d1agja_-d1klih_.fasta

./group126/reference/d1agja_-d1ltoa_.fasta

./group126/reference/d1agja_-d1lvoa_.fasta

./group126/reference/d1agja_-d1m9ua_.fasta

./group126/reference/d1agja_-d1mzaa_.fasta

./group126/reference/d1agja_-d1orfa_.fasta

./group126/reference/d1agja_-d1rfna_.fasta

./group126/reference/d1agja_-d1sgt__.fasta

./group126/reference/d1agja_-d2hlca_.fasta

./group126/reference/d1arb__-d1azza_.fasta

./group126/reference/d1arb__-d1bio__.fasta

./group126/reference/d1arb__-d1bqya_.fasta

./group126/reference/d1arb__-d1cgha_.fasta

./group126/reference/d1arb__-d1ddja_.fasta

./group126/reference/d1arb__-d1eaxa_.fasta

./group126/reference/d1arb__-d1ekbb_.fasta

./group126/reference/d1arb__-d1eq9a_.fasta

./group126/reference/d1arb__-d1fjsa_.fasta

./group126/reference/d1arb__-d1gvkb_.fasta

./group126/reference/d1arb__-d1gvza_.fasta

./group126/reference/d1arb__-d1hj9a_.fasta

./group126/reference/d1arb__-d1klih_.fasta

./group126/reference/d1arb__-d1ltoa_.fasta

./group126/reference/d1arb__-d1mzaa_.fasta

./group126/reference/d1arb__-d1qtfa_.fasta

./group126/reference/d1arb__-d1rfna_.fasta

./group126/reference/d1arb__-d1sgt__.fasta

./group126/reference/d1arb__-d2hlca_.fasta

./group126/reference/d1azza_-d1qtfa_.fasta

./group126/reference/d1bio__-d1lvoa_.fasta

./group126/reference/d1bio__-d1qtfa_.fasta

./group126/reference/d1bqya_-d1lvoa_.fasta

./group126/reference/d1bqya_-d1qtfa_.fasta

./group126/reference/d1cgha_-d1lvoa_.fasta

./group126/reference/d1cgha_-d1qtfa_.fasta

./group126/reference/d1ddja_-d1lvoa_.fasta

./group126/reference/d1ddja_-d1qtfa_.fasta

./group126/reference/d1eaxa_-d1qtfa_.fasta

./group126/reference/d1ekbb_-d1lvoa_.fasta

./group126/reference/d1ekbb_-d1qtfa_.fasta

./group126/reference/d1eq9a_-d1qtfa_.fasta

./group126/reference/d1fjsa_-d1lvoa_.fasta

./group126/reference/d1fjsa_-d1qtfa_.fasta

./group126/reference/d1gdna_-d1lvoa_.fasta

./group126/reference/d1gvkb_-d1lvoa_.fasta

./group126/reference/d1gvkb_-d1qtfa_.fasta

./group126/reference/d1gvza_-d1lvoa_.fasta

./group126/reference/d1gvza_-d1qtfa_.fasta

./group126/reference/d1hj9a_-d1lvoa_.fasta

./group126/reference/d1hj9a_-d1qtfa_.fasta

./group126/reference/d1klih_-d1lvoa_.fasta

./group126/reference/d1klih_-d1qtfa_.fasta

./group126/reference/d1ltoa_-d1lvoa_.fasta

./group126/reference/d1ltoa_-d1qtfa_.fasta

./group126/reference/d1lvoa_-d1m9ua_.fasta

./group126/reference/d1lvoa_-d1mzaa_.fasta

./group126/reference/d1lvoa_-d1orfa_.fasta

./group126/reference/d1lvoa_-d1qtfa_.fasta

./group126/reference/d1lvoa_-d1sgt__.fasta

./group126/reference/d1m9ua_-d1qtfa_.fasta

./group126/reference/d1mzaa_-d1qtfa_.fasta

./group126/reference/d1orfa_-d1qtfa_.fasta

./group126/reference/d1qtfa_-d1sgt__.fasta

./group126/reference/d1qtfa_-d2hlca_.fasta

./group127/reference/d1e79a2-d1e79d2.fasta

./group127/reference/d1e79a2-d1fx0b2.fasta

./group128/reference/d1dpja_-d1fmb__.fasta

./group128/reference/d1dpja_-d1idaa_.fasta

./group128/reference/d1dpja_-d1kzka_.fasta

./group128/reference/d1dpja_-d4fiv__.fasta

./group128/reference/d1fkna_-d1fmb__.fasta

./group128/reference/d1fkna_-d1kzka_.fasta

./group128/reference/d1fkna_-d4fiv__.fasta

./group128/reference/d1fmb__-d1j71a_.fasta

./group128/reference/d1fmb__-d1lf2a_.fasta

./group128/reference/d1fmb__-d1mpp__.fasta

./group128/reference/d1fmb__-d2apr__.fasta

./group128/reference/d1idaa_-d1j71a_.fasta

./group128/reference/d1idaa_-d1nsoa_.fasta

./group128/reference/d1idaa_-d2apr__.fasta

./group128/reference/d1j71a_-d1kzka_.fasta

./group128/reference/d1j71a_-d1nsoa_.fasta

./group128/reference/d1j71a_-d4fiv__.fasta

./group128/reference/d1kzka_-d1mpp__.fasta

./group128/reference/d1kzka_-d2apr__.fasta

./group128/reference/d1lf2a_-d1nsoa_.fasta

./group128/reference/d1lf2a_-d4fiv__.fasta

./group128/reference/d1mpp__-d1nsoa_.fasta

./group128/reference/d1mpp__-d4fiv__.fasta

./group128/reference/d1nsoa_-d2apr__.fasta

./group128/reference/d1nsoa_-d4fiv__.fasta

./group128/reference/d2apr__-d4fiv__.fasta

./group130/reference/d1cr5a1-d1cz4a1.fasta

./group130/reference/d1cr5a1-d1e32a1.fasta

./group130/reference/d1cr5a1-d1eu1a1.fasta

./group130/reference/d1cr5a1-d1h0ha1.fasta

./group130/reference/d1cr5a1-d1tmo_1.fasta

./group130/reference/d1cr5a1-d2napa1.fasta

./group130/reference/d1cz4a1-d1eu1a1.fasta

./group130/reference/d1cz4a1-d1kqfa1.fasta

./group130/reference/d1cz4a1-d1tmo_1.fasta

./group130/reference/d1cz4a1-d2napa1.fasta

./group130/reference/d1e32a1-d1eu1a1.fasta

./group130/reference/d1e32a1-d1g8ka1.fasta

./group130/reference/d1e32a1-d1h0ha1.fasta

./group130/reference/d1e32a1-d1kqfa1.fasta

./group130/reference/d1e32a1-d1tmo_1.fasta

./group130/reference/d1e32a1-d2napa1.fasta

./group130/reference/d1eu1a1-d1g8ka1.fasta

./group130/reference/d1g8ka1-d1kqfa1.fasta

./group131/reference/d1btn__-d1dyna_.fasta

./group131/reference/d1btn__-d1evha_.fasta

./group131/reference/d1btn__-d1faoa_.fasta

./group131/reference/d1btn__-d1mai__.fasta

./group131/reference/d1btn__-d1mixa2.fasta

./group131/reference/d1btn__-d1qqga2.fasta

./group131/reference/d1ddma_-d1faoa_.fasta

./group131/reference/d1ddma_-d1h4ra2.fasta

./group131/reference/d1ddma_-d1k5db_.fasta

./group131/reference/d1ddma_-d1mkea1.fasta

./group131/reference/d1ddma_-d1ntva_.fasta

./group131/reference/d1ddma_-d1pls__.fasta

./group131/reference/d1dro__-d1dyna_.fasta

./group131/reference/d1dro__-d1eaza_.fasta

./group131/reference/d1dro__-d1faoa_.fasta

./group131/reference/d1dro__-d1fhoa_.fasta

./group131/reference/d1dro__-d1ntva_.fasta

./group131/reference/d1dro__-d1pls__.fasta

./group131/reference/d1dro__-d1qqga2.fasta

./group131/reference/d1dyna_-d1fhoa_.fasta

./group131/reference/d1dyna_-d1mai__.fasta

./group131/reference/d1dyna_-d1mixa2.fasta

./group131/reference/d1dyna_-d1qqga2.fasta

./group131/reference/d1eaza_-d1gg3a2.fasta

./group131/reference/d1eaza_-d1h4ra2.fasta

./group131/reference/d1eaza_-d1mixa2.fasta

./group131/reference/d1eaza_-d1mkea1.fasta

./group131/reference/d1eaza_-d1qqga2.fasta

./group131/reference/d1evha_-d1k5db_.fasta

./group131/reference/d1evha_-d1mkea1.fasta

./group131/reference/d1faoa_-d1fhoa_.fasta

./group131/reference/d1faoa_-d1mixa2.fasta

./group131/reference/d1faoa_-d1mkea1.fasta

./group131/reference/d1faoa_-d1ntva_.fasta

./group131/reference/d1faoa_-d1qqga2.fasta

./group131/reference/d1faoa_-d1shca_.fasta

./group131/reference/d1fhoa_-d1pls__.fasta

./group131/reference/d1fhoa_-d1qqga1.fasta

./group131/reference/d1fhoa_-d1qqga2.fasta

./group131/reference/d1gg3a2-d1mixa2.fasta

./group131/reference/d1gg3a2-d1ntva_.fasta

./group131/reference/d1gg3a2-d1shca_.fasta

./group131/reference/d1h4ra2-d1mai__.fasta

./group131/reference/d1h4ra2-d1ntva_.fasta

./group131/reference/d1h4ra2-d1pls__.fasta

./group131/reference/d1h4ra2-d1qqga1.fasta

./group131/reference/d1h4ra2-d1shca_.fasta

./group131/reference/d1k5db_-d1mai__.fasta

./group131/reference/d1k5db_-d1mkea1.fasta

./group131/reference/d1k5db_-d1ntva_.fasta

./group131/reference/d1k5db_-d1qqga1.fasta

./group131/reference/d1mai__-d1mixa2.fasta

./group131/reference/d1mai__-d1ntva_.fasta

./group131/reference/d1mai__-d1pls__.fasta

./group131/reference/d1mai__-d1qqga1.fasta

./group131/reference/d1mai__-d1qqga2.fasta

./group131/reference/d1mai__-d1shca_.fasta

./group131/reference/d1mixa2-d1mkea1.fasta

./group131/reference/d1mixa2-d1ntva_.fasta

./group131/reference/d1mixa2-d1pls__.fasta

./group131/reference/d1mixa2-d1qqga2.fasta

./group131/reference/d1mixa2-d1shca_.fasta

./group131/reference/d1mkea1-d1ntva_.fasta

./group131/reference/d1mkea1-d1qqga1.fasta

./group131/reference/d1ntva_-d1pls__.fasta

./group131/reference/d1ntva_-d1qqga1.fasta

./group131/reference/d1pls__-d1qqga2.fasta

./group131/reference/d1qqga2-d1shca_.fasta

./group133/reference/d1avgi_-d1b56__.fasta

./group133/reference/d1avgi_-d1beba_.fasta

./group133/reference/d1avgi_-d1dzka_.fasta

./group133/reference/d1avgi_-d1euoa_.fasta

./group133/reference/d1avgi_-d1ew3a_.fasta

./group133/reference/d1avgi_-d1ftpa_.fasta

./group133/reference/d1avgi_-d1g85a_.fasta

./group133/reference/d1avgi_-d1ggla_.fasta

./group133/reference/d1avgi_-d1gkab_.fasta

./group133/reference/d1avgi_-d1hms__.fasta

./group133/reference/d1avgi_-d1i4ua_.fasta

./group133/reference/d1avgi_-d1ifc__.fasta

./group133/reference/d1avgi_-d1jv4a_.fasta

./group133/reference/d1avgi_-d1jzua_.fasta

./group133/reference/d1avgi_-d1koia_.fasta

./group133/reference/d1avgi_-d1kt7a_.fasta

./group133/reference/d1avgi_-d1p6pa_.fasta

./group133/reference/d1avgi_-d1qqsa_.fasta

./group133/reference/d1b56__-d1bj7__.fasta

./group133/reference/d1b56__-d1dzka_.fasta

./group133/reference/d1b56__-d1g85a_.fasta

./group133/reference/d1b56__-d1i4ua_.fasta

./group133/reference/d1b56__-d1jv4a_.fasta

./group133/reference/d1b56__-d1jzua_.fasta

./group133/reference/d1b56__-d1mdc__.fasta

./group133/reference/d1b56__-d1p6pa_.fasta

./group133/reference/d1beba_-d1bj7__.fasta

./group133/reference/d1beba_-d1cbs__.fasta

./group133/reference/d1beba_-d1euoa_.fasta

./group133/reference/d1beba_-d1g85a_.fasta

./group133/reference/d1beba_-d1gkab_.fasta

./group133/reference/d1beba_-d1i4ua_.fasta

./group133/reference/d1beba_-d1jv4a_.fasta

./group133/reference/d1beba_-d1koia_.fasta

./group133/reference/d1beba_-d1qfta_.fasta

./group133/reference/d1beba_-d1qqsa_.fasta

./group133/reference/d1bj7__-d1cbs__.fasta

./group133/reference/d1bj7__-d1euoa_.fasta

./group133/reference/d1bj7__-d1ggla_.fasta

./group133/reference/d1bj7__-d1gkab_.fasta

./group133/reference/d1bj7__-d1hms__.fasta

./group133/reference/d1bj7__-d1ifc__.fasta

./group133/reference/d1bj7__-d1jzua_.fasta

./group133/reference/d1bj7__-d1kqwa_.fasta

./group133/reference/d1bj7__-d1kt7a_.fasta

./group133/reference/d1bj7__-d1mdc__.fasta

./group133/reference/d1bj7__-d1o1va_.fasta

./group133/reference/d1bj7__-d1p6pa_.fasta

./group133/reference/d1bj7__-d1qfta_.fasta

./group133/reference/d1bj7__-d1qqsa_.fasta

./group133/reference/d1cbs__-d1dzka_.fasta

./group133/reference/d1cbs__-d1euoa_.fasta

./group133/reference/d1cbs__-d1ew3a_.fasta

./group133/reference/d1cbs__-d1g85a_.fasta

./group133/reference/d1cbs__-d1i4ua_.fasta

./group133/reference/d1cbs__-d1jv4a_.fasta

./group133/reference/d1cbs__-d1jzua_.fasta

./group133/reference/d1cbs__-d1koia_.fasta

./group133/reference/d1cbs__-d1kt7a_.fasta

./group133/reference/d1dzka_-d1euoa_.fasta

./group133/reference/d1dzka_-d1ftpa_.fasta

./group133/reference/d1dzka_-d1gkab_.fasta

./group133/reference/d1dzka_-d1hms__.fasta

./group133/reference/d1dzka_-d1ifc__.fasta

./group133/reference/d1dzka_-d1jzua_.fasta

./group133/reference/d1dzka_-d1koia_.fasta

./group133/reference/d1dzka_-d1kt7a_.fasta

./group133/reference/d1dzka_-d1qfta_.fasta

./group133/reference/d1dzka_-d1qqsa_.fasta

./group133/reference/d1euoa_-d1ew3a_.fasta

./group133/reference/d1euoa_-d1g85a_.fasta

./group133/reference/d1euoa_-d1gkab_.fasta

./group133/reference/d1euoa_-d1i4ua_.fasta

./group133/reference/d1euoa_-d1ifc__.fasta

./group133/reference/d1euoa_-d1jv4a_.fasta

./group133/reference/d1euoa_-d1jzua_.fasta

./group133/reference/d1euoa_-d1kt7a_.fasta

./group133/reference/d1euoa_-d1p6pa_.fasta

./group133/reference/d1euoa_-d1qqsa_.fasta

./group133/reference/d1ew3a_-d1ggla_.fasta

./group133/reference/d1ew3a_-d1gkab_.fasta

./group133/reference/d1ew3a_-d1i4ua_.fasta

./group133/reference/d1ew3a_-d1ifc__.fasta

./group133/reference/d1ew3a_-d1koia_.fasta

./group133/reference/d1ew3a_-d1o1va_.fasta

./group133/reference/d1ew3a_-d1qfta_.fasta

./group133/reference/d1ew3a_-d1qqsa_.fasta

./group133/reference/d1ftpa_-d1g85a_.fasta

./group133/reference/d1ftpa_-d1i4ua_.fasta

./group133/reference/d1ftpa_-d1jzua_.fasta

./group133/reference/d1ftpa_-d1kt7a_.fasta

./group133/reference/d1ftpa_-d1qqsa_.fasta

./group133/reference/d1g85a_-d1ggla_.fasta

./group133/reference/d1g85a_-d1gkab_.fasta

./group133/reference/d1g85a_-d1hms__.fasta

./group133/reference/d1g85a_-d1i4ua_.fasta

./group133/reference/d1g85a_-d1ifc__.fasta

./group133/reference/d1g85a_-d1jzua_.fasta

./group133/reference/d1g85a_-d1koia_.fasta

./group133/reference/d1g85a_-d1kqwa_.fasta

./group133/reference/d1g85a_-d1kt7a_.fasta

./group133/reference/d1g85a_-d1o1va_.fasta

./group133/reference/d1g85a_-d1qfta_.fasta

./group133/reference/d1g85a_-d1qqsa_.fasta

./group133/reference/d1ggla_-d1i4ua_.fasta

./group133/reference/d1ggla_-d1kt7a_.fasta

./group133/reference/d1gkab_-d1ifc__.fasta

./group133/reference/d1gkab_-d1jv4a_.fasta

./group133/reference/d1gkab_-d1koia_.fasta

./group133/reference/d1gkab_-d1o1va_.fasta

./group133/reference/d1gkab_-d1p6pa_.fasta

./group133/reference/d1gkab_-d1qfta_.fasta

./group133/reference/d1gkab_-d1qqsa_.fasta

./group133/reference/d1hms__-d1i4ua_.fasta

./group133/reference/d1hms__-d1jv4a_.fasta

./group133/reference/d1hms__-d1jzua_.fasta

./group133/reference/d1hms__-d1kt7a_.fasta

./group133/reference/d1hms__-d1qqsa_.fasta

./group133/reference/d1i4ua_-d1ifc__.fasta

./group133/reference/d1i4ua_-d1jv4a_.fasta

./group133/reference/d1i4ua_-d1koia_.fasta

./group133/reference/d1i4ua_-d1kqwa_.fasta

./group133/reference/d1i4ua_-d1kt7a_.fasta

./group133/reference/d1i4ua_-d1o1va_.fasta

./group133/reference/d1i4ua_-d1p6pa_.fasta

./group133/reference/d1i4ua_-d1qfta_.fasta

./group133/reference/d1i4ua_-d1qqsa_.fasta

./group133/reference/d1ifc__-d1jv4a_.fasta

./group133/reference/d1ifc__-d1kt7a_.fasta

./group133/reference/d1ifc__-d1mdc__.fasta

./group133/reference/d1jv4a_-d1koia_.fasta

./group133/reference/d1jv4a_-d1kt7a_.fasta

./group133/reference/d1jv4a_-d1o1va_.fasta

./group133/reference/d1jv4a_-d1p6pa_.fasta

./group133/reference/d1jv4a_-d1qfta_.fasta

./group133/reference/d1jv4a_-d1qqsa_.fasta

./group133/reference/d1jzua_-d1koia_.fasta

./group133/reference/d1jzua_-d1kt7a_.fasta

./group133/reference/d1jzua_-d1o1va_.fasta

./group133/reference/d1jzua_-d1p6pa_.fasta

./group133/reference/d1jzua_-d1qfta_.fasta

./group133/reference/d1jzua_-d1qqsa_.fasta

./group133/reference/d1koia_-d1kt7a_.fasta

./group133/reference/d1koia_-d1p6pa_.fasta

./group133/reference/d1koia_-d1qfta_.fasta

./group133/reference/d1koia_-d1qqsa_.fasta

./group133/reference/d1kqwa_-d1kt7a_.fasta

./group133/reference/d1kt7a_-d1p6pa_.fasta

./group133/reference/d1kt7a_-d1qqsa_.fasta

./group133/reference/d1mdc__-d1qfta_.fasta

./group133/reference/d1mdc__-d1qqsa_.fasta

./group133/reference/d1p6pa_-d1qqsa_.fasta

./group136/reference/d1e8ua_-d1eur__.fasta

./group136/reference/d1e8ua_-d1n1ta2.fasta

./group136/reference/d1e8ua_-d1nsca_.fasta

./group136/reference/d1e8ua_-d2bat__.fasta

./group136/reference/d1e8ua_-d2sli_2.fasta

./group136/reference/d1e8ua_-d3sil__.fasta

./group136/reference/d1eur__-d1f8ea_.fasta

./group136/reference/d1eur__-d1nsca_.fasta

./group136/reference/d1eur__-d2bat__.fasta

./group136/reference/d1f8ea_-d1n1ta2.fasta

./group136/reference/d1n1ta2-d1nsca_.fasta

./group136/reference/d1n1ta2-d2bat__.fasta

./group136/reference/d1nsca_-d2sli_2.fasta

./group136/reference/d1nsca_-d3sil__.fasta

./group136/reference/d2bat__-d2sli_2.fasta

./group137/reference/d1nr0a1-d1nr0a2.fasta

./group137/reference/d1nr0a1-d1tbga_.fasta

./group137/reference/d1nr0a2-d1p22a2.fasta

./group137/reference/d1nr0a2-d1tbga_.fasta

./group138/reference/d1bag_1-d1e43a1.fasta

./group138/reference/d1bag_1-d1g5aa1.fasta

./group138/reference/d1bag_1-d1gcya1.fasta

./group138/reference/d1bag_1-d1ht6a1.fasta

./group138/reference/d1bag_1-d1iv8a1.fasta

./group138/reference/d1bag_1-d1ji2a2.fasta

./group138/reference/d1bag_1-d1ktba1.fasta

./group138/reference/d1bag_1-d1kwga1.fasta

./group138/reference/d1bag_1-d1m53a1.fasta

./group138/reference/d1bag_1-d1m7xa2.fasta

./group138/reference/d1bag_1-d1mxga1.fasta

./group138/reference/d1bag_1-d1uok_1.fasta

./group138/reference/d1bag_1-d7taa_1.fasta

./group138/reference/d1bf2_2-d1ea9c2.fasta

./group138/reference/d1bf2_2-d1g5aa1.fasta

./group138/reference/d1bf2_2-d1g94a1.fasta

./group138/reference/d1bf2_2-d1gcya1.fasta

./group138/reference/d1bf2_2-d1gjwa1.fasta

./group138/reference/d1bf2_2-d1hx0a1.fasta

./group138/reference/d1bf2_2-d1iv8a1.fasta

./group138/reference/d1bf2_2-d1j0ha2.fasta

./group138/reference/d1bf2_2-d1jae_1.fasta

./group138/reference/d1bf2_2-d1ji2a2.fasta

./group138/reference/d1bf2_2-d1m7xa2.fasta

./group138/reference/d1bf2_2-d1qhoa3.fasta

./group138/reference/d1bf2_2-d1uasa1.fasta

./group138/reference/d1bf2_2-d1uok_1.fasta

./group138/reference/d1bf2_2-d7taa_1.fasta

./group138/reference/d1e43a1-d1gcya1.fasta

./group138/reference/d1e43a1-d1gjwa1.fasta

./group138/reference/d1e43a1-d1hx0a1.fasta

./group138/reference/d1e43a1-d1iv8a1.fasta

./group138/reference/d1e43a1-d1jae_1.fasta

./group138/reference/d1e43a1-d1ji1a2.fasta

./group138/reference/d1e43a1-d1ji2a2.fasta

./group138/reference/d1e43a1-d1ktba1.fasta

./group138/reference/d1e43a1-d1m53a1.fasta

./group138/reference/d1e43a1-d1m7xa2.fasta

./group138/reference/d1e43a1-d1qhoa3.fasta

./group138/reference/d1e43a1-d1uasa1.fasta

./group138/reference/d1e43a1-d7taa_1.fasta

./group138/reference/d1ea9c2-d1g5aa1.fasta

./group138/reference/d1ea9c2-d1g94a1.fasta

./group138/reference/d1ea9c2-d1gcya1.fasta

./group138/reference/d1ea9c2-d1gjwa1.fasta

./group138/reference/d1ea9c2-d1j0ha2.fasta

./group138/reference/d1ea9c2-d1jae_1.fasta

./group138/reference/d1ea9c2-d1ktba1.fasta

./group138/reference/d1ea9c2-d1kwga1.fasta

./group138/reference/d1ea9c2-d1m53a1.fasta

./group138/reference/d1ea9c2-d1m7xa2.fasta

./group138/reference/d1ea9c2-d1mxga1.fasta

./group138/reference/d1ea9c2-d1qhoa3.fasta

./group138/reference/d1ea9c2-d1uasa1.fasta

./group138/reference/d1ea9c2-d1uok_1.fasta

./group138/reference/d1ea9c2-d7taa_1.fasta

./group138/reference/d1eh9a2-d1gjwa1.fasta

./group138/reference/d1eh9a2-d1j0ha2.fasta

./group138/reference/d1eh9a2-d1kwga1.fasta

./group138/reference/d1eh9a2-d1m7xa2.fasta

./group138/reference/d1eh9a2-d1mxga1.fasta

./group138/reference/d1eh9a2-d1uasa1.fasta

./group138/reference/d1eh9a2-d1uok_1.fasta

./group138/reference/d1g5aa1-d1g94a1.fasta

./group138/reference/d1g5aa1-d1gjwa1.fasta

./group138/reference/d1g5aa1-d1hx0a1.fasta

./group138/reference/d1g5aa1-d1iv8a1.fasta

./group138/reference/d1g5aa1-d1j0ha2.fasta

./group138/reference/d1g5aa1-d1jae_1.fasta

./group138/reference/d1g5aa1-d1ji1a2.fasta

./group138/reference/d1g5aa1-d1ktba1.fasta

./group138/reference/d1g5aa1-d1kwga1.fasta

./group138/reference/d1g5aa1-d1mxga1.fasta

./group138/reference/d1g5aa1-d1qhoa3.fasta

./group138/reference/d1g5aa1-d1uasa1.fasta

./group138/reference/d1g5aa1-d7taa_1.fasta

./group138/reference/d1g94a1-d1gcya1.fasta

./group138/reference/d1g94a1-d1gjwa1.fasta

./group138/reference/d1g94a1-d1ht6a1.fasta

./group138/reference/d1g94a1-d1iv8a1.fasta

./group138/reference/d1g94a1-d1j0ha2.fasta

./group138/reference/d1g94a1-d1ji1a2.fasta

./group138/reference/d1g94a1-d1ji2a2.fasta

./group138/reference/d1g94a1-d1kwga1.fasta

./group138/reference/d1g94a1-d1m53a1.fasta

./group138/reference/d1g94a1-d1m7xa2.fasta

./group138/reference/d1g94a1-d1mxga1.fasta

./group138/reference/d1g94a1-d7taa_1.fasta

./group138/reference/d1gcya1-d1ji1a2.fasta

./group138/reference/d1gcya1-d1ji2a2.fasta

./group138/reference/d1gcya1-d1ktba1.fasta

./group138/reference/d1gcya1-d1m53a1.fasta

./group138/reference/d1gcya1-d1m7xa2.fasta

./group138/reference/d1gcya1-d1mxga1.fasta

./group138/reference/d1gcya1-d1uasa1.fasta

./group138/reference/d1gcya1-d7taa_1.fasta

./group138/reference/d1gjwa1-d1ht6a1.fasta

./group138/reference/d1gjwa1-d1hx0a1.fasta

./group138/reference/d1gjwa1-d1iv8a1.fasta

./group138/reference/d1gjwa1-d1j0ha2.fasta

./group138/reference/d1gjwa1-d1jae_1.fasta

./group138/reference/d1gjwa1-d1ji1a2.fasta

./group138/reference/d1gjwa1-d1m53a1.fasta

./group138/reference/d1gjwa1-d1qhoa3.fasta

./group138/reference/d1gjwa1-d1uasa1.fasta

./group138/reference/d1gjwa1-d1uok_1.fasta

./group138/reference/d1ht6a1-d1iv8a1.fasta

./group138/reference/d1ht6a1-d1j0ha2.fasta

./group138/reference/d1ht6a1-d1ji1a2.fasta

./group138/reference/d1ht6a1-d1ji2a2.fasta

./group138/reference/d1ht6a1-d1ktba1.fasta

./group138/reference/d1ht6a1-d1m53a1.fasta

./group138/reference/d1ht6a1-d1uasa1.fasta

./group138/reference/d1hx0a1-d1iv8a1.fasta

./group138/reference/d1hx0a1-d1j0ha2.fasta

./group138/reference/d1hx0a1-d1kwga1.fasta

./group138/reference/d1hx0a1-d1m53a1.fasta

./group138/reference/d1hx0a1-d1m7xa2.fasta

./group138/reference/d1hx0a1-d1uasa1.fasta

./group138/reference/d1hx0a1-d7taa_1.fasta

./group138/reference/d1iv8a1-d1j0ha2.fasta

./group138/reference/d1iv8a1-d1jae_1.fasta

./group138/reference/d1iv8a1-d1kwga1.fasta

./group138/reference/d1iv8a1-d1m53a1.fasta

./group138/reference/d1iv8a1-d1m7xa2.fasta

./group138/reference/d1iv8a1-d1qhoa3.fasta

./group138/reference/d1iv8a1-d1uasa1.fasta

./group138/reference/d1iv8a1-d1uok_1.fasta

./group138/reference/d1j0ha2-d1ji1a2.fasta

./group138/reference/d1j0ha2-d1ktba1.fasta

./group138/reference/d1j0ha2-d1kwga1.fasta

./group138/reference/d1j0ha2-d1m7xa2.fasta

./group138/reference/d1j0ha2-d1mxga1.fasta

./group138/reference/d1j0ha2-d1qhoa3.fasta

./group138/reference/d1j0ha2-d1uasa1.fasta

./group138/reference/d1jae_1-d1ji2a2.fasta

./group138/reference/d1jae_1-d1kwga1.fasta

./group138/reference/d1jae_1-d1m53a1.fasta

./group138/reference/d1jae_1-d1m7xa2.fasta

./group138/reference/d1jae_1-d1uasa1.fasta

./group138/reference/d1jae_1-d1uok_1.fasta

./group138/reference/d1ji1a2-d1ktba1.fasta

./group138/reference/d1ji1a2-d1kwga1.fasta

./group138/reference/d1ji1a2-d1m53a1.fasta

./group138/reference/d1ji1a2-d1m7xa2.fasta

./group138/reference/d1ji1a2-d1mxga1.fasta

./group138/reference/d1ji1a2-d1qhoa3.fasta

./group138/reference/d1ji1a2-d1uasa1.fasta

./group138/reference/d1ji1a2-d1uok_1.fasta

./group138/reference/d1ji2a2-d1ktba1.fasta

./group138/reference/d1ji2a2-d1kwga1.fasta

./group138/reference/d1ji2a2-d1qhoa3.fasta

./group138/reference/d1ji2a2-d1uasa1.fasta

./group138/reference/d1ji2a2-d7taa_1.fasta

./group138/reference/d1ktba1-d1m53a1.fasta

./group138/reference/d1ktba1-d1qhoa3.fasta

./group138/reference/d1ktba1-d1uok_1.fasta

./group138/reference/d1ktba1-d7taa_1.fasta

./group138/reference/d1kwga1-d1m53a1.fasta

./group138/reference/d1kwga1-d1m7xa2.fasta

./group138/reference/d1kwga1-d1qhoa3.fasta

./group138/reference/d1kwga1-d1uok_1.fasta

./group138/reference/d1kwga1-d7taa_1.fasta

./group138/reference/d1m53a1-d1mxga1.fasta

./group138/reference/d1m53a1-d7taa_1.fasta

./group138/reference/d1m7xa2-d1mxga1.fasta

./group138/reference/d1m7xa2-d1qhoa3.fasta

./group138/reference/d1m7xa2-d1uok_1.fasta

./group138/reference/d1mxga1-d1qhoa3.fasta

./group138/reference/d1mxga1-d7taa_1.fasta

./group138/reference/d1qhoa3-d1uasa1.fasta

./group138/reference/d1qhoa3-d1uok_1.fasta

./group138/reference/d1uasa1-d1uok_1.fasta

./group141/reference/d1ciy_2-d1i5pa2.fasta

./group143/reference/d1bhe__-d1bn8a_.fasta

./group143/reference/d1bhe__-d1czfa_.fasta

./group143/reference/d1bhe__-d1daba_.fasta

./group143/reference/d1bhe__-d1jtaa_.fasta

./group143/reference/d1bhe__-d1qcxa_.fasta

./group143/reference/d1bhe__-d1qjva_.fasta

./group143/reference/d1bhe__-d1rmg__.fasta

./group143/reference/d1bn8a_-d1czfa_.fasta

./group143/reference/d1bn8a_-d1dbga_.fasta

./group143/reference/d1bn8a_-d1ee6a_.fasta

./group143/reference/d1bn8a_-d1k5ca_.fasta

./group143/reference/d1bn8a_-d1rmg__.fasta

./group143/reference/d1czfa_-d1daba_.fasta

./group143/reference/d1czfa_-d1ee6a_.fasta

./group143/reference/d1czfa_-d1jtaa_.fasta

./group143/reference/d1czfa_-d1qcxa_.fasta

./group143/reference/d1czfa_-d1qjva_.fasta

./group143/reference/d1czfa_-d1rmg__.fasta

./group143/reference/d1daba_-d1dbga_.fasta

./group143/reference/d1daba_-d1hg8a_.fasta

./group143/reference/d1daba_-d1jtaa_.fasta

./group143/reference/d1daba_-d1k5ca_.fasta

./group143/reference/d1dbga_-d1hg8a_.fasta

./group143/reference/d1dbga_-d1jtaa_.fasta

./group143/reference/d1dbga_-d1k5ca_.fasta

./group143/reference/d1dbga_-d1qcxa_.fasta

./group143/reference/d1ee6a_-d1hg8a_.fasta

./group143/reference/d1ee6a_-d1jtaa_.fasta

./group143/reference/d1ee6a_-d1k5ca_.fasta

./group143/reference/d1ee6a_-d1qcxa_.fasta

./group143/reference/d1ee6a_-d1qjva_.fasta

./group143/reference/d1hg8a_-d1qcxa_.fasta

./group143/reference/d1hg8a_-d1qjva_.fasta

./group143/reference/d1jtaa_-d1k5ca_.fasta

./group143/reference/d1jtaa_-d1qjva_.fasta

./group143/reference/d1k5ca_-d1qcxa_.fasta

./group143/reference/d1k5ca_-d1qjva_.fasta

./group143/reference/d1k5ca_-d1rmg__.fasta

./group143/reference/d1qcxa_-d1qjva_.fasta

./group143/reference/d1qjva_-d1rmg__.fasta

./group144/reference/d1kk6a_-d1qrea_.fasta

./group144/reference/d1krra_-d3tdt__.fasta

./group144/reference/d1qrea_-d1xat__.fasta

./group144/reference/d1xat__-d3tdt__.fasta

./group145/reference/d1dgwa_-d1dzra_.fasta

./group145/reference/d1dgwa_-d1ep0a_.fasta

./group145/reference/d1dgwa_-d1fi2a_.fasta

./group145/reference/d1dgwa_-d1fxza2.fasta

./group145/reference/d1dgwa_-d1lkna_.fasta

./group145/reference/d1dgwa_-d1lrha_.fasta

./group145/reference/d1dgwa_-d1m4oa_.fasta

./group145/reference/d1dgwa_-d1nxma_.fasta

./group145/reference/d1dgwa_-d1o4ta_.fasta

./group145/reference/d1dgwa_-d1od5a2.fasta

./group145/reference/d1dgwa_-d1pmi__.fasta

./group145/reference/d1dzra_-d1fi2a_.fasta

./group145/reference/d1dzra_-d1fxza2.fasta

./group145/reference/d1dzra_-d1lkna_.fasta

./group145/reference/d1dzra_-d1lrha_.fasta

./group145/reference/d1dzra_-d1o4ta_.fasta

./group145/reference/d1dzra_-d1od5a2.fasta

./group145/reference/d1dzra_-d2phla1.fasta

./group145/reference/d1ep0a_-d1fi2a_.fasta

./group145/reference/d1ep0a_-d1fxza2.fasta

./group145/reference/d1ep0a_-d1lrha_.fasta

./group145/reference/d1ep0a_-d1o4ta_.fasta

./group145/reference/d1ep0a_-d1od5a2.fasta

./group145/reference/d1ep0a_-d2phla1.fasta

./group145/reference/d1fi2a_-d1fxza2.fasta

./group145/reference/d1fi2a_-d1lkna_.fasta

./group145/reference/d1fi2a_-d1m4oa_.fasta

./group145/reference/d1fi2a_-d1nxma_.fasta

./group145/reference/d1fi2a_-d1o4ta_.fasta

./group145/reference/d1fi2a_-d1od5a2.fasta

./group145/reference/d1fi2a_-d1pmi__.fasta

./group145/reference/d1fi2a_-d2phla1.fasta

./group145/reference/d1fxza2-d1lkna_.fasta

./group145/reference/d1fxza2-d1nxma_.fasta

./group145/reference/d1fxza2-d1o4ta_.fasta

./group145/reference/d1fxza2-d1pmi__.fasta

./group145/reference/d1fxza2-d2phla1.fasta

./group145/reference/d1lkna_-d1m4oa_.fasta

./group145/reference/d1lkna_-d1nxma_.fasta

./group145/reference/d1lkna_-d1o4ta_.fasta

./group145/reference/d1lkna_-d1od5a2.fasta

./group145/reference/d1lkna_-d1pmi__.fasta

./group145/reference/d1lrha_-d1m4oa_.fasta

./group145/reference/d1lrha_-d1o4ta_.fasta

./group145/reference/d1lrha_-d1od5a2.fasta

./group145/reference/d1lrha_-d1pmi__.fasta

./group145/reference/d1lrha_-d2phla1.fasta

./group145/reference/d1m4oa_-d1nxma_.fasta

./group145/reference/d1m4oa_-d1o4ta_.fasta

./group145/reference/d1m4oa_-d1pmi__.fasta

./group145/reference/d1m4oa_-d2phla1.fasta

./group145/reference/d1nxma_-d1o4ta_.fasta

./group145/reference/d1nxma_-d1od5a2.fasta

./group145/reference/d1nxma_-d1pmi__.fasta

./group145/reference/d1nxma_-d2phla1.fasta

./group145/reference/d1o4ta_-d1od5a2.fasta

./group145/reference/d1o4ta_-d1pmi__.fasta

./group145/reference/d1o4ta_-d2phla1.fasta

./group145/reference/d1od5a2-d1pmi__.fasta

./group145/reference/d1od5a2-d2phla1.fasta

./group146/reference/d1ds1a_-d1gp6a_.fasta

./group146/reference/d1ds1a_-d1gy9a_.fasta

./group146/reference/d1ds1a_-d1odma_.fasta

./group146/reference/d1gp6a_-d1gy9a_.fasta

./group147/reference/d1cx4a1-d1ft9a2.fasta

./group147/reference/d1cx4a1-d1i5za2.fasta

./group147/reference/d1ft9a2-d1o7fa2.fasta

./group147/reference/d1ft9a2-d1o7fa3.fasta

./group147/reference/d1ft9a2-d1rgs_1.fasta

./group147/reference/d1ft9a2-d1rgs_2.fasta

./group147/reference/d1i5za2-d1rgs_2.fasta

./group148/reference/d1fyc__-d1htp__.fasta

./group148/reference/d1ghk__-d1htp__.fasta

./group148/reference/d1htp__-d1k8ma_.fasta

./group149/reference/d1b6ra1-d1dv1a1.fasta

./group149/reference/d1b6ra1-d1e2wa2.fasta

./group149/reference/d1dv1a1-d1hcz_2.fasta

./group149/reference/d1e2wa2-d1kjqa1.fasta

./group152/reference/d1dun__-d1ogha_.fasta

./group154/reference/d1a53__-d1dbta_.fasta

./group154/reference/d1a53__-d1dqwa_.fasta

./group154/reference/d1a53__-d1eixa_.fasta

./group154/reference/d1a53__-d1km3a_.fasta

./group154/reference/d1a53__-d1kv8a_.fasta

./group154/reference/d1a53__-d1nsj__.fasta

./group154/reference/d1a53__-d1rpxa_.fasta

./group154/reference/d1dbta_-d1i4na_.fasta

./group154/reference/d1dbta_-d1kv8a_.fasta

./group154/reference/d1dbta_-d1nsj__.fasta

./group154/reference/d1dbta_-d1rpxa_.fasta

./group154/reference/d1dqwa_-d1i4na_.fasta

./group154/reference/d1dqwa_-d1km3a_.fasta

./group154/reference/d1dqwa_-d1kv8a_.fasta

./group154/reference/d1dqwa_-d1pii_1.fasta

./group154/reference/d1dqwa_-d1rpxa_.fasta

./group154/reference/d1dqwa_-d1thfd_.fasta

./group154/reference/d1eixa_-d1i4na_.fasta

./group154/reference/d1eixa_-d1kv8a_.fasta

./group154/reference/d1eixa_-d1nsj__.fasta

./group154/reference/d1eixa_-d1pii_2.fasta

./group154/reference/d1eixa_-d1rpxa_.fasta

./group154/reference/d1eixa_-d1thfd_.fasta

./group154/reference/d1i4na_-d1km3a_.fasta

./group154/reference/d1i4na_-d1kv8a_.fasta

./group154/reference/d1i4na_-d1nsj__.fasta

./group154/reference/d1i4na_-d1rpxa_.fasta

./group154/reference/d1i4na_-d1thfd_.fasta

./group154/reference/d1km3a_-d1pii_1.fasta

./group154/reference/d1km3a_-d1rpxa_.fasta

./group154/reference/d1km3a_-d1thfd_.fasta

./group154/reference/d1kv8a_-d1pii_1.fasta

./group154/reference/d1kv8a_-d1rpxa_.fasta

./group154/reference/d1kv8a_-d1thfd_.fasta

./group154/reference/d1nsj__-d1pii_1.fasta

./group154/reference/d1nsj__-d1rpxa_.fasta

./group154/reference/d1nsj__-d1thfd_.fasta

./group154/reference/d1pii_1-d1pii_2.fasta

./group154/reference/d1pii_1-d1rpxa_.fasta

./group154/reference/d1pii_1-d1thfd_.fasta

./group154/reference/d1pii_2-d1rpxa_.fasta

./group154/reference/d1pii_2-d1thfd_.fasta

./group155/reference/d1ep3a_-d1o94a1.fasta

./group155/reference/d1ep3a_-d1oyb__.fasta

./group155/reference/d1gvoa_-d2dora_.fasta

./group155/reference/d1oyb__-d2dora_.fasta

./group157/reference/d1bf2_3-d1cz1a_.fasta

./group157/reference/d1bf2_3-d1eswa_.fasta

./group157/reference/d1bf2_3-d1g5aa2.fasta

./group157/reference/d1bf2_3-d1iexa1.fasta

./group157/reference/d1bf2_3-d1j18a2.fasta

./group157/reference/d1bf2_3-d1kwga2.fasta

./group157/reference/d1bf2_3-d1qvba_.fasta

./group157/reference/d1cbg__-d1cz1a_.fasta

./group157/reference/d1cbg__-d1e43a2.fasta

./group157/reference/d1cbg__-d1kwga2.fasta

./group157/reference/d1cbg__-d1pama4.fasta

./group157/reference/d1cbg__-d1qba_3.fasta

./group157/reference/d1cbg__-d1qhoa4.fasta

./group157/reference/d1cz1a_-d1e43a2.fasta

./group157/reference/d1cz1a_-d1e4ia_.fasta

./group157/reference/d1cz1a_-d1e4mm_.fasta

./group157/reference/d1cz1a_-d1eh9a3.fasta

./group157/reference/d1cz1a_-d1eswa_.fasta

./group157/reference/d1cz1a_-d1hxja_.fasta

./group157/reference/d1cz1a_-d1j0ha3.fasta

./group157/reference/d1cz1a_-d1ji1a3.fasta

./group157/reference/d1cz1a_-d1kwga2.fasta

./group157/reference/d1cz1a_-d1lwha2.fasta

./group157/reference/d1cz1a_-d1pama4.fasta

./group157/reference/d1cz1a_-d1qba_3.fasta

./group157/reference/d1cz1a_-d1qhoa4.fasta

./group157/reference/d1cz1a_-d1qvba_.fasta

./group157/reference/d1cz1a_-d1ug6a_.fasta

./group157/reference/d1e43a2-d1eswa_.fasta

./group157/reference/d1e43a2-d1gjwa2.fasta

./group157/reference/d1e43a2-d1iexa1.fasta

./group157/reference/d1e43a2-d1j18a2.fasta

./group157/reference/d1e43a2-d1kwga2.fasta

./group157/reference/d1e4ia_-d1eswa_.fasta

./group157/reference/d1e4ia_-d1g5aa2.fasta

./group157/reference/d1e4ia_-d1j0ha3.fasta

./group157/reference/d1e4ia_-d1kwga2.fasta

./group157/reference/d1e4ia_-d1uok_2.fasta

./group157/reference/d1e4mm_-d1eh9a3.fasta

./group157/reference/d1e4mm_-d1kwga2.fasta

./group157/reference/d1e4mm_-d1pama4.fasta

./group157/reference/d1e4mm_-d1qba_3.fasta

./group157/reference/d1eh9a3-d1eswa_.fasta

./group157/reference/d1eh9a3-d1g5aa2.fasta

./group157/reference/d1eh9a3-d1iexa1.fasta

./group157/reference/d1eh9a3-d1j18a2.fasta

./group157/reference/d1eh9a3-d1kwga2.fasta

./group157/reference/d1eh9a3-d1qba_3.fasta

./group157/reference/d1eh9a3-d1qvba_.fasta

./group157/reference/d1eswa_-d1g5aa2.fasta

./group157/reference/d1eswa_-d1gjwa2.fasta

./group157/reference/d1eswa_-d1hxja_.fasta

./group157/reference/d1eswa_-d1j0ha3.fasta

./group157/reference/d1eswa_-d1j18a2.fasta

./group157/reference/d1eswa_-d1ji1a3.fasta

./group157/reference/d1eswa_-d1kwga2.fasta

./group157/reference/d1eswa_-d1lwha2.fasta

./group157/reference/d1eswa_-d1m53a2.fasta

./group157/reference/d1eswa_-d1pama4.fasta

./group157/reference/d1eswa_-d1qhoa4.fasta

./group157/reference/d1eswa_-d1ug6a_.fasta

./group157/reference/d1eswa_-d1uok_2.fasta

./group157/reference/d1eswa_-d7taa_2.fasta

./group157/reference/d1g5aa2-d1gjwa2.fasta

./group157/reference/d1gjwa2-d1m53a2.fasta

./group157/reference/d1gjwa2-d1pama4.fasta

./group157/reference/d1gjwa2-d1qhoa4.fasta

./group157/reference/d1hxja_-d1j18a2.fasta

./group157/reference/d1hxja_-d1kwga2.fasta

./group157/reference/d1hxja_-d1lwha2.fasta

./group157/reference/d1hxja_-d1m53a2.fasta

./group157/reference/d1hxja_-d1pama4.fasta

./group157/reference/d1hxja_-d1qba_3.fasta

./group157/reference/d1hxja_-d1qhoa4.fasta

./group157/reference/d1hxja_-d1uok_2.fasta

./group157/reference/d1iexa1-d1j0ha3.fasta

./group157/reference/d1iexa1-d1j18a2.fasta

./group157/reference/d1iexa1-d1m53a2.fasta

./group157/reference/d1iexa1-d1qba_3.fasta

./group157/reference/d1iexa1-d1qhoa4.fasta

./group157/reference/d1j0ha3-d1j18a2.fasta

./group157/reference/d1j0ha3-d1kwga2.fasta

./group157/reference/d1j0ha3-d1qba_3.fasta

./group157/reference/d1j0ha3-d1qvba_.fasta

./group157/reference/d1j0ha3-d1ug6a_.fasta

./group157/reference/d1j18a2-d1kwga2.fasta

./group157/reference/d1j18a2-d1qba_3.fasta

./group157/reference/d1j18a2-d1qvba_.fasta

./group157/reference/d1j18a2-d1ug6a_.fasta

./group157/reference/d1j18a2-d7taa_2.fasta

./group157/reference/d1ji1a3-d1kwga2.fasta

./group157/reference/d1ji1a3-d1qvba_.fasta

./group157/reference/d1kwga2-d1lwha2.fasta

./group157/reference/d1kwga2-d1pama4.fasta

./group157/reference/d1kwga2-d1qba_3.fasta

./group157/reference/d1kwga2-d1qvba_.fasta

./group157/reference/d1kwga2-d1ug6a_.fasta

./group157/reference/d1kwga2-d1uok_2.fasta

./group157/reference/d1lwha2-d1qba_3.fasta

./group157/reference/d1lwha2-d1qvba_.fasta

./group157/reference/d1m53a2-d1qvba_.fasta

./group157/reference/d1pama4-d1qvba_.fasta

./group157/reference/d1qba_3-d1qvba_.fasta

./group157/reference/d1qhoa4-d1ug6a_.fasta

./group157/reference/d1qvba_-d7taa_2.fasta

./group158/reference/d1a4ma_-d1bf6a_.fasta

./group158/reference/d1a4ma_-d1i0da_.fasta

./group158/reference/d1a4ma_-d1j5sa_.fasta

./group158/reference/d1a4ma_-d1k6wa2.fasta

./group158/reference/d1a4ma_-d1m7ja3.fasta

./group158/reference/d1a4ma_-d1p1ma2.fasta

./group158/reference/d1bf6a_-d1itua_.fasta

./group158/reference/d1bf6a_-d1j5sa_.fasta

./group158/reference/d1bf6a_-d1k6wa2.fasta

./group158/reference/d1bf6a_-d1m7ja3.fasta

./group158/reference/d1bf6a_-d1p1ma2.fasta

./group158/reference/d1i0da_-d1itua_.fasta

./group158/reference/d1i0da_-d1k6wa2.fasta

./group158/reference/d1i0da_-d1m7ja3.fasta

./group158/reference/d1i0da_-d1p1ma2.fasta

./group158/reference/d1itua_-d1j5sa_.fasta

./group158/reference/d1itua_-d1k6wa2.fasta

./group158/reference/d1itua_-d1m7ja3.fasta

./group158/reference/d1itua_-d1p1ma2.fasta

./group158/reference/d1j5sa_-d1m7ja3.fasta

./group158/reference/d1k6wa2-d1m7ja3.fasta

./group158/reference/d1k6wa2-d1p1ma2.fasta

./group158/reference/d1m7ja3-d1p1ma2.fasta

./group159/reference/d1adoa_-d1dosa_.fasta

./group159/reference/d1adoa_-d1euaa_.fasta

./group159/reference/d1adoa_-d1f74a_.fasta

./group159/reference/d1adoa_-d1hl2a_.fasta

./group159/reference/d1adoa_-d1jcla_.fasta

./group159/reference/d1adoa_-d1mzha_.fasta

./group159/reference/d1adoa_-d1n7ka_.fasta

./group159/reference/d1adoa_-d1o0ya_.fasta

./group159/reference/d1adoa_-d1ub3a_.fasta

./group159/reference/d1dhpa_-d1dosa_.fasta

./group159/reference/d1dhpa_-d1euaa_.fasta

./group159/reference/d1dhpa_-d1gzga_.fasta

./group159/reference/d1dhpa_-d1jcla_.fasta

./group159/reference/d1dhpa_-d1mzha_.fasta

./group159/reference/d1dhpa_-d1n7ka_.fasta

./group159/reference/d1dhpa_-d1n8fa_.fasta

./group159/reference/d1dhpa_-d1o0ya_.fasta

./group159/reference/d1dhpa_-d1ohla_.fasta

./group159/reference/d1dhpa_-d1ub3a_.fasta

./group159/reference/d1dosa_-d1epxa_.fasta

./group159/reference/d1dosa_-d1jcla_.fasta

./group159/reference/d1dosa_-d1n8fa_.fasta

./group159/reference/d1dosa_-d1ub3a_.fasta

./group159/reference/d1epxa_-d1f74a_.fasta

./group159/reference/d1epxa_-d1gzga_.fasta

./group159/reference/d1epxa_-d1mzha_.fasta

./group159/reference/d1epxa_-d1n7ka_.fasta

./group159/reference/d1epxa_-d1o0ya_.fasta

./group159/reference/d1epxa_-d1ohla_.fasta

./group159/reference/d1epxa_-d1ub3a_.fasta

./group159/reference/d1euaa_-d1f74a_.fasta

./group159/reference/d1euaa_-d1gzga_.fasta

./group159/reference/d1euaa_-d1hl2a_.fasta

./group159/reference/d1euaa_-d1jcla_.fasta

./group159/reference/d1euaa_-d1l6wa_.fasta

./group159/reference/d1euaa_-d1mzha_.fasta

./group159/reference/d1euaa_-d1n7ka_.fasta

./group159/reference/d1euaa_-d1n8fa_.fasta

./group159/reference/d1euaa_-d1nvma2.fasta

./group159/reference/d1euaa_-d1o0ya_.fasta

./group159/reference/d1euaa_-d1ohla_.fasta

./group159/reference/d1euaa_-d1qfea_.fasta

./group159/reference/d1euaa_-d1ub3a_.fasta

./group159/reference/d1f74a_-d1gzga_.fasta

./group159/reference/d1f74a_-d1i2oa_.fasta

./group159/reference/d1f74a_-d1l6wa_.fasta

./group159/reference/d1f74a_-d1mzha_.fasta

./group159/reference/d1f74a_-d1n7ka_.fasta

./group159/reference/d1f74a_-d1nvma2.fasta

./group159/reference/d1f74a_-d1o0ya_.fasta

./group159/reference/d1f74a_-d1qfea_.fasta

./group159/reference/d1f74a_-d1ub3a_.fasta

./group159/reference/d1gzga_-d1hl2a_.fasta

./group159/reference/d1gzga_-d1jcla_.fasta

./group159/reference/d1gzga_-d1l6wa_.fasta

./group159/reference/d1gzga_-d1mzha_.fasta

./group159/reference/d1gzga_-d1n7ka_.fasta

./group159/reference/d1gzga_-d1nvma2.fasta

./group159/reference/d1gzga_-d1o0ya_.fasta

./group159/reference/d1gzga_-d1qfea_.fasta

./group159/reference/d1gzga_-d1ub3a_.fasta

./group159/reference/d1hl2a_-d1jcla_.fasta

./group159/reference/d1hl2a_-d1l6wa_.fasta

./group159/reference/d1hl2a_-d1mzha_.fasta

./group159/reference/d1hl2a_-d1n7ka_.fasta

./group159/reference/d1hl2a_-d1nvma2.fasta

./group159/reference/d1hl2a_-d1o0ya_.fasta

./group159/reference/d1hl2a_-d1ohla_.fasta

./group159/reference/d1hl2a_-d1qfea_.fasta

./group159/reference/d1hl2a_-d1ub3a_.fasta

./group159/reference/d1i2oa_-d1jcla_.fasta

./group159/reference/d1i2oa_-d1mzha_.fasta

./group159/reference/d1i2oa_-d1n8fa_.fasta

./group159/reference/d1i2oa_-d1nvma2.fasta

./group159/reference/d1i2oa_-d1ub3a_.fasta

./group159/reference/d1jcla_-d1l6wa_.fasta

./group159/reference/d1jcla_-d1nvma2.fasta

./group159/reference/d1jcla_-d1ohla_.fasta

./group159/reference/d1jcla_-d1qfea_.fasta

./group159/reference/d1l6wa_-d1mzha_.fasta

./group159/reference/d1l6wa_-d1n7ka_.fasta

./group159/reference/d1l6wa_-d1nvma2.fasta

./group159/reference/d1l6wa_-d1o0ya_.fasta

./group159/reference/d1l6wa_-d1ohla_.fasta

./group159/reference/d1l6wa_-d1qfea_.fasta

./group159/reference/d1l6wa_-d1ub3a_.fasta

./group159/reference/d1mzha_-d1n8fa_.fasta

./group159/reference/d1mzha_-d1nvma2.fasta

./group159/reference/d1mzha_-d1ohla_.fasta

./group159/reference/d1mzha_-d1qfea_.fasta

./group159/reference/d1n7ka_-d1nvma2.fasta

./group159/reference/d1n7ka_-d1ohla_.fasta

./group159/reference/d1n7ka_-d1qfea_.fasta

./group159/reference/d1nvma2-d1o0ya_.fasta

./group159/reference/d1nvma2-d1qfea_.fasta

./group159/reference/d1nvma2-d1ub3a_.fasta

./group159/reference/d1o0ya_-d1ohla_.fasta

./group159/reference/d1o0ya_-d1qfea_.fasta

./group159/reference/d1ohla_-d1qfea_.fasta

./group159/reference/d1ohla_-d1ub3a_.fasta

./group159/reference/d1qfea_-d1ub3a_.fasta

./group160/reference/d1e9ia1-d1ec7a1.fasta

./group160/reference/d1e9ia1-d1jpdx1.fasta

./group160/reference/d1e9ia1-d1jpma1.fasta

./group160/reference/d1e9ia1-d1muca1.fasta

./group160/reference/d1e9ia1-d2chr_1.fasta

./group160/reference/d1e9ia1-d2mnr_1.fasta

./group160/reference/d1ec7a1-d1jpdx1.fasta

./group160/reference/d1ec7a1-d1jpma1.fasta

./group160/reference/d1ec7a1-d1muca1.fasta

./group160/reference/d1ec7a1-d1onea1.fasta

./group160/reference/d1ec7a1-d2chr_1.fasta

./group160/reference/d1jpdx1-d1onea1.fasta

./group160/reference/d1jpdx1-d2mnr_1.fasta

./group160/reference/d1jpma1-d1onea1.fasta

./group160/reference/d1muca1-d1onea1.fasta

./group160/reference/d1onea1-d2chr_1.fasta

./group160/reference/d1onea1-d2mnr_1.fasta

./group160/reference/d2chr_1-d2mnr_1.fasta

./group161/reference/d1dxea_-d1f8ma_.fasta

./group161/reference/d1dxea_-d1kbla1.fasta

./group161/reference/d1dxea_-d1muma_.fasta

./group161/reference/d1f8ma_-d1izca_.fasta

./group161/reference/d1f8ma_-d1kbla1.fasta

./group161/reference/d1f8ma_-d1m3ua_.fasta

./group161/reference/d1izca_-d1kbla1.fasta

./group161/reference/d1izca_-d1m3ua_.fasta

./group161/reference/d1izca_-d1muma_.fasta

./group161/reference/d1kbla1-d1m3ua_.fasta

./group161/reference/d1m3ua_-d1muma_.fasta

./group162/reference/d1a0ca_-d1qtwa_.fasta

./group162/reference/d1muwa_-d1qtwa_.fasta

./group163/reference/d1ezwa_-d1lucb_.fasta

./group164/reference/d1o1za_-d2plc__.fasta

./group164/reference/d1o1za_-d2ptd__.fasta

./group165/reference/d1ccwb_-d1eexa_.fasta

./group165/reference/d1ccwb_-d7reqa1.fasta

./group165/reference/d1ccwb_-d7reqb1.fasta

./group165/reference/d1eexa_-d7reqb1.fasta

./group166/reference/d1b16a_-d1bgva1.fasta

./group166/reference/d1b16a_-d1e6ua_.fasta

./group166/reference/d1b16a_-d1ek6a_.fasta

./group166/reference/d1b16a_-d1eno__.fasta

./group166/reference/d1b16a_-d1eny__.fasta

./group166/reference/d1b16a_-d1gz3a1.fasta

./group166/reference/d1b16a_-d1hwxa1.fasta

./group166/reference/d1b16a_-d1kepa_.fasta

./group166/reference/d1b16a_-d1kewa_.fasta

./group166/reference/d1b16a_-d1n2sa_.fasta

./group166/reference/d1b16a_-d1qg6a_.fasta

./group166/reference/d1bgva1-d1e6ua_.fasta

./group166/reference/d1bgva1-d1ek6a_.fasta

./group166/reference/d1bgva1-d1eny__.fasta

./group166/reference/d1bgva1-d1fmca_.fasta

./group166/reference/d1bgva1-d1g0oa_.fasta

./group166/reference/d1bgva1-d1gcoa_.fasta

./group166/reference/d1bgva1-d1gega_.fasta

./group166/reference/d1bgva1-d1gz3a1.fasta

./group166/reference/d1bgva1-d1h5qa_.fasta

./group166/reference/d1bgva1-d1hdca_.fasta

./group166/reference/d1bgva1-d1hxha_.fasta

./group166/reference/d1bgva1-d1iy8a_.fasta

./group166/reference/d1bgva1-d1ja9a_.fasta

./group166/reference/d1bgva1-d1n2sa_.fasta

./group166/reference/d1bgva1-d1n5da_.fasta

./group166/reference/d1bgva1-d1o0sa1.fasta

./group166/reference/d1bgva1-d1oaa__.fasta

./group166/reference/d1bgva1-d1qg6a_.fasta

./group166/reference/d1bgva1-d2ae2a_.fasta

./group166/reference/d1e6ua_-d1ek6a_.fasta

./group166/reference/d1e6ua_-d1eno__.fasta

./group166/reference/d1e6ua_-d1eny__.fasta

./group166/reference/d1e6ua_-d1fmca_.fasta

./group166/reference/d1e6ua_-d1g0oa_.fasta

./group166/reference/d1e6ua_-d1gcoa_.fasta

./group166/reference/d1e6ua_-d1gega_.fasta

./group166/reference/d1e6ua_-d1gz3a1.fasta

./group166/reference/d1e6ua_-d1h5qa_.fasta

./group166/reference/d1e6ua_-d1hdca_.fasta

./group166/reference/d1e6ua_-d1hwxa1.fasta

./group166/reference/d1e6ua_-d1hxha_.fasta

./group166/reference/d1e6ua_-d1iy8a_.fasta

./group166/reference/d1e6ua_-d1ja9a_.fasta

./group166/reference/d1e6ua_-d1n2sa_.fasta

./group166/reference/d1e6ua_-d1n5da_.fasta

./group166/reference/d1e6ua_-d1o0sa1.fasta

./group166/reference/d1e6ua_-d1oaa__.fasta

./group166/reference/d1e6ua_-d1qg6a_.fasta

./group166/reference/d1e6ua_-d2ae2a_.fasta

./group166/reference/d1ek6a_-d1eno__.fasta

./group166/reference/d1ek6a_-d1eny__.fasta

./group166/reference/d1ek6a_-d1fmca_.fasta

./group166/reference/d1ek6a_-d1g0oa_.fasta

./group166/reference/d1ek6a_-d1gcoa_.fasta

./group166/reference/d1ek6a_-d1gega_.fasta

./group166/reference/d1ek6a_-d1gz3a1.fasta

./group166/reference/d1ek6a_-d1h5qa_.fasta

./group166/reference/d1ek6a_-d1hdca_.fasta

./group166/reference/d1ek6a_-d1hwxa1.fasta

./group166/reference/d1ek6a_-d1hxha_.fasta

./group166/reference/d1ek6a_-d1iy8a_.fasta

./group166/reference/d1ek6a_-d1n2sa_.fasta

./group166/reference/d1ek6a_-d1n5da_.fasta

./group166/reference/d1ek6a_-d1o0sa1.fasta

./group166/reference/d1ek6a_-d1oaa__.fasta

./group166/reference/d1ek6a_-d1qg6a_.fasta

./group166/reference/d1ek6a_-d2ae2a_.fasta

./group166/reference/d1eno__-d1g0oa_.fasta

./group166/reference/d1eno__-d1hwxa1.fasta

./group166/reference/d1eno__-d1kepa_.fasta

./group166/reference/d1eno__-d1kewa_.fasta

./group166/reference/d1eno__-d1n2sa_.fasta

./group166/reference/d1eno__-d1n5da_.fasta

./group166/reference/d1eny__-d1gcoa_.fasta

./group166/reference/d1eny__-d1gz3a1.fasta

./group166/reference/d1eny__-d1h5qa_.fasta

./group166/reference/d1eny__-d1hwxa1.fasta

./group166/reference/d1eny__-d1kepa_.fasta

./group166/reference/d1eny__-d1kewa_.fasta

./group166/reference/d1eny__-d1n2sa_.fasta

./group166/reference/d1eny__-d1o0sa1.fasta

./group166/reference/d1eny__-d1oaa__.fasta

./group166/reference/d1fmca_-d1hwxa1.fasta

./group166/reference/d1fmca_-d1kepa_.fasta

./group166/reference/d1fmca_-d1kewa_.fasta

./group166/reference/d1fmca_-d1n2sa_.fasta

./group166/reference/d1g0oa_-d1hwxa1.fasta

./group166/reference/d1g0oa_-d1kepa_.fasta

./group166/reference/d1g0oa_-d1kewa_.fasta

./group166/reference/d1g0oa_-d1n2sa_.fasta

./group166/reference/d1g0oa_-d1oaa__.fasta

./group166/reference/d1gcoa_-d1gz3a1.fasta

./group166/reference/d1gcoa_-d1hwxa1.fasta

./group166/reference/d1gcoa_-d1kepa_.fasta

./group166/reference/d1gcoa_-d1kewa_.fasta

./group166/reference/d1gcoa_-d1n2sa_.fasta

./group166/reference/d1gcoa_-d1qg6a_.fasta

./group166/reference/d1gega_-d1gz3a1.fasta

./group166/reference/d1gega_-d1hwxa1.fasta

./group166/reference/d1gega_-d1kewa_.fasta

./group166/reference/d1gega_-d1o0sa1.fasta

./group166/reference/d1gega_-d1oaa__.fasta

./group166/reference/d1gz3a1-d1h5qa_.fasta

./group166/reference/d1gz3a1-d1hdca_.fasta

./group166/reference/d1gz3a1-d1hwxa1.fasta

./group166/reference/d1gz3a1-d1hxha_.fasta

./group166/reference/d1gz3a1-d1kepa_.fasta

./group166/reference/d1gz3a1-d1kewa_.fasta

./group166/reference/d1gz3a1-d1n2sa_.fasta

./group166/reference/d1gz3a1-d1oaa__.fasta

./group166/reference/d1gz3a1-d1qg6a_.fasta

./group166/reference/d1h5qa_-d1n2sa_.fasta

./group166/reference/d1h5qa_-d1o0sa1.fasta

./group166/reference/d1h5qa_-d1oaa__.fasta

./group166/reference/d1hdca_-d1hwxa1.fasta

./group166/reference/d1hdca_-d1kepa_.fasta

./group166/reference/d1hdca_-d1kewa_.fasta

./group166/reference/d1hdca_-d1n2sa_.fasta

./group166/reference/d1hdca_-d1o0sa1.fasta

./group166/reference/d1hwxa1-d1hxha_.fasta

./group166/reference/d1hwxa1-d1iy8a_.fasta

./group166/reference/d1hwxa1-d1ja9a_.fasta

./group166/reference/d1hwxa1-d1kepa_.fasta

./group166/reference/d1hwxa1-d1n2sa_.fasta

./group166/reference/d1hwxa1-d1n5da_.fasta

./group166/reference/d1hwxa1-d1o0sa1.fasta

./group166/reference/d1hwxa1-d1oaa__.fasta

./group166/reference/d1hwxa1-d1qg6a_.fasta

./group166/reference/d1hwxa1-d2ae2a_.fasta

./group166/reference/d1hxha_-d1kepa_.fasta

./group166/reference/d1hxha_-d1kewa_.fasta

./group166/reference/d1hxha_-d1n2sa_.fasta

./group166/reference/d1iy8a_-d1kepa_.fasta

./group166/reference/d1iy8a_-d1kewa_.fasta

./group166/reference/d1iy8a_-d1n2sa_.fasta

./group166/reference/d1ja9a_-d1kepa_.fasta

./group166/reference/d1ja9a_-d1n2sa_.fasta

./group166/reference/d1kepa_-d1n2sa_.fasta

./group166/reference/d1kepa_-d1oaa__.fasta

./group166/reference/d1kepa_-d1qg6a_.fasta

./group166/reference/d1kepa_-d2ae2a_.fasta

./group166/reference/d1kewa_-d1n2sa_.fasta

./group166/reference/d1kewa_-d1o0sa1.fasta

./group166/reference/d1kewa_-d1oaa__.fasta

./group166/reference/d1kewa_-d1qg6a_.fasta

./group166/reference/d1kewa_-d2ae2a_.fasta

./group166/reference/d1n2sa_-d1n5da_.fasta

./group166/reference/d1n2sa_-d1o0sa1.fasta

./group166/reference/d1n2sa_-d1oaa__.fasta

./group166/reference/d1n2sa_-d1qg6a_.fasta

./group166/reference/d1n2sa_-d2ae2a_.fasta

./group166/reference/d1n5da_-d1o0sa1.fasta

./group166/reference/d1n5da_-d1qg6a_.fasta

./group166/reference/d1o0sa1-d1qg6a_.fasta

./group166/reference/d1o0sa1-d2ae2a_.fasta

./group166/reference/d1oaa__-d1qg6a_.fasta

./group167/reference/d1cjca1-d1fcda2.fasta

./group167/reference/d1cjca1-d1feca2.fasta

./group167/reference/d1cjca1-d1fl2a2.fasta

./group167/reference/d1cjca1-d1gesa2.fasta

./group167/reference/d1cjca1-d1h6va2.fasta

./group167/reference/d1cjca1-d1jeha2.fasta

./group167/reference/d1cjca1-d1lvl_2.fasta

./group167/reference/d1cjca1-d1m6ia2.fasta

./group167/reference/d1cjca1-d1mo9a2.fasta

./group167/reference/d1cjca1-d1nhp_2.fasta

./group167/reference/d1cjca1-d1o94a2.fasta

./group167/reference/d1cjca1-d1ojt_2.fasta

./group167/reference/d1cjca1-d1onfa2.fasta

./group167/reference/d1cjca1-d1vdc_2.fasta

./group167/reference/d1d7ya2-d1fcda2.fasta

./group167/reference/d1d7ya2-d1gtea3.fasta

./group167/reference/d1d7ya2-d1h6va2.fasta

./group167/reference/d1d7ya2-d1mo9a2.fasta

./group167/reference/d1d7ya2-d1o94a2.fasta

./group167/reference/d1d7ya2-d1onfa2.fasta

./group167/reference/d1d7ya2-d1trb_2.fasta

./group167/reference/d1d7ya2-d1vdc_2.fasta

./group167/reference/d1ebda2-d1fcda2.fasta

./group167/reference/d1ebda2-d1gtea3.fasta

./group167/reference/d1ebda2-d1m6ia2.fasta

./group167/reference/d1ebda2-d1o94a2.fasta

./group167/reference/d1ebda2-d1trb_2.fasta

./group167/reference/d1ebda2-d1vdc_2.fasta

./group167/reference/d1fcda2-d1feca2.fasta

./group167/reference/d1fcda2-d1fl2a2.fasta

./group167/reference/d1fcda2-d1gesa2.fasta

./group167/reference/d1fcda2-d1gtea3.fasta

./group167/reference/d1fcda2-d1h6va2.fasta

./group167/reference/d1fcda2-d1jeha2.fasta

./group167/reference/d1fcda2-d1lvl_2.fasta

./group167/reference/d1fcda2-d1m6ia2.fasta

./group167/reference/d1fcda2-d1mo9a2.fasta

./group167/reference/d1fcda2-d1o94a2.fasta

./group167/reference/d1fcda2-d1trb_2.fasta

./group167/reference/d1fcda2-d1vdc_2.fasta

./group167/reference/d1fcda2-d3grs_2.fasta

./group167/reference/d1fcda2-d3lada2.fasta

./group167/reference/d1feca2-d1fl2a2.fasta

./group167/reference/d1feca2-d1m6ia2.fasta

./group167/reference/d1feca2-d1o94a2.fasta

./group167/reference/d1feca2-d1vdc_2.fasta

./group167/reference/d1feca2-d3lada2.fasta

./group167/reference/d1fl2a2-d1gtea3.fasta

./group167/reference/d1fl2a2-d1h6va2.fasta

./group167/reference/d1fl2a2-d1o94a2.fasta

./group167/reference/d1fl2a2-d1ojt_2.fasta

./group167/reference/d1fl2a2-d1onfa2.fasta

./group167/reference/d1gesa2-d1gtea3.fasta

./group167/reference/d1gesa2-d1mo9a2.fasta

./group167/reference/d1gesa2-d1o94a2.fasta

./group167/reference/d1gesa2-d1vdc_2.fasta

./group167/reference/d1gtea3-d1jeha2.fasta

./group167/reference/d1gtea3-d1lvl_2.fasta

./group167/reference/d1gtea3-d1m6ia2.fasta

./group167/reference/d1gtea3-d1mo9a2.fasta

./group167/reference/d1gtea3-d1nhp_2.fasta

./group167/reference/d1gtea3-d1o94a2.fasta

./group167/reference/d1gtea3-d1ojt_2.fasta

./group167/reference/d1gtea3-d1onfa2.fasta

./group167/reference/d1gtea3-d1vdc_2.fasta

./group167/reference/d1gtea3-d3lada2.fasta

./group167/reference/d1h6va2-d1jeha2.fasta

./group167/reference/d1h6va2-d1m6ia2.fasta

./group167/reference/d1h6va2-d1o94a2.fasta

./group167/reference/d1h6va2-d1trb_2.fasta

./group167/reference/d1h6va2-d1vdc_2.fasta

./group167/reference/d1jeha2-d1m6ia2.fasta

./group167/reference/d1jeha2-d1mo9a2.fasta

./group167/reference/d1jeha2-d1o94a2.fasta

./group167/reference/d1jeha2-d1trb_2.fasta

./group167/reference/d1lvl_2-d1m6ia2.fasta

./group167/reference/d1lvl_2-d1o94a2.fasta

./group167/reference/d1lvl_2-d1vdc_2.fasta

./group167/reference/d1m6ia2-d1mo9a2.fasta

./group167/reference/d1m6ia2-d1nhp_2.fasta

./group167/reference/d1m6ia2-d1o94a2.fasta

./group167/reference/d1m6ia2-d1ojt_2.fasta

./group167/reference/d1m6ia2-d1trb_2.fasta

./group167/reference/d1m6ia2-d1vdc_2.fasta

./group167/reference/d1m6ia2-d3grs_2.fasta

./group167/reference/d1mo9a2-d1o94a2.fasta

./group167/reference/d1mo9a2-d1ojt_2.fasta

./group167/reference/d1mo9a2-d1onfa2.fasta

./group167/reference/d1mo9a2-d1trb_2.fasta

./group167/reference/d1mo9a2-d1vdc_2.fasta

./group167/reference/d1mo9a2-d3lada2.fasta

./group167/reference/d1nhp_2-d1o94a2.fasta

./group167/reference/d1nhp_2-d1ojt_2.fasta

./group167/reference/d1nhp_2-d1vdc_2.fasta

./group167/reference/d1o94a2-d1ojt_2.fasta

./group167/reference/d1o94a2-d1onfa2.fasta

./group167/reference/d1o94a2-d1trb_2.fasta

./group167/reference/d1o94a2-d1vdc_2.fasta

./group167/reference/d1o94a2-d3lada2.fasta

./group167/reference/d1ojt_2-d1onfa2.fasta

./group167/reference/d1ojt_2-d1trb_2.fasta

./group167/reference/d1ojt_2-d1vdc_2.fasta

./group167/reference/d1onfa2-d1trb_2.fasta

./group167/reference/d1onfa2-d1vdc_2.fasta

./group167/reference/d1trb_2-d3lada2.fasta

./group167/reference/d1vdc_2-d3grs_2.fasta

./group167/reference/d1vdc_2-d3lada2.fasta

./group170/reference/d1a4ya_-d1io0a_.fasta

./group170/reference/d1a4ya_-d1pgva_.fasta

./group170/reference/d1fqva2-d1io0a_.fasta

./group170/reference/d1fqva2-d1pgva_.fasta

./group170/reference/d1fqva2-d1yrga_.fasta

./group170/reference/d1io0a_-d1yrga_.fasta

./group170/reference/d1pgva_-d1yrga_.fasta

./group171/reference/d1a9na_-d1igra1.fasta

./group171/reference/d1a9na_-d1m6ba2.fasta

./group171/reference/d1a9na_-d1n8yc2.fasta

./group171/reference/d1a9na_-d1nqla1.fasta

./group171/reference/d1a9na_-d1nqla2.fasta

./group171/reference/d1dcea3-d1igra1.fasta

./group171/reference/d1dcea3-d1m6ba2.fasta

./group171/reference/d1dcea3-d1n8yc2.fasta

./group171/reference/d1dcea3-d1nqla1.fasta

./group171/reference/d1dcea3-d1nqla2.fasta

./group171/reference/d1h6ta2-d1igra1.fasta

./group171/reference/d1h6ta2-d1jl5a_.fasta

./group171/reference/d1h6ta2-d1m6ba2.fasta

./group171/reference/d1h6ta2-d1n8yc2.fasta

./group171/reference/d1h6ta2-d1nqla1.fasta

./group171/reference/d1h6ta2-d1nqla2.fasta

./group171/reference/d1h6ua2-d1igra1.fasta

./group171/reference/d1h6ua2-d1m6ba2.fasta

./group171/reference/d1h6ua2-d1n8yc2.fasta

./group171/reference/d1h6ua2-d1nqla2.fasta

./group171/reference/d1h6ua2-d1ogqa_.fasta

./group171/reference/d1igra1-d1koha1.fasta

./group171/reference/d1igra1-d1ogqa_.fasta

./group171/reference/d1igra1-d1ozna_.fasta

./group171/reference/d1igra1-d1p9ag_.fasta

./group171/reference/d1jl5a_-d1m6ba2.fasta

./group171/reference/d1jl5a_-d1n8yc2.fasta

./group171/reference/d1koha1-d1m6ba2.fasta

./group171/reference/d1koha1-d1n8yc2.fasta

./group171/reference/d1koha1-d1nqla1.fasta

./group171/reference/d1koha1-d1nqla2.fasta

./group171/reference/d1m6ba2-d1ogqa_.fasta

./group171/reference/d1m6ba2-d1ozna_.fasta

./group171/reference/d1m6ba2-d1p9ag_.fasta

./group171/reference/d1n8yc2-d1ogqa_.fasta

./group171/reference/d1n8yc2-d1p9ag_.fasta

./group171/reference/d1nqla1-d1ogqa_.fasta

./group171/reference/d1nqla1-d1p9ag_.fasta

./group171/reference/d1nqla2-d1ogqa_.fasta

./group171/reference/d1nqla2-d1ozna_.fasta

./group171/reference/d1nqla2-d1p9ag_.fasta

./group172/reference/d1dcia_-d1on3a1.fasta

./group172/reference/d1dcia_-d1on3a2.fasta

./group172/reference/d1dcia_-d1tyfa_.fasta

./group172/reference/d1hzda_-d1on3a1.fasta

./group172/reference/d1hzda_-d1on3a2.fasta

./group172/reference/d1hzda_-d1tyfa_.fasta

./group172/reference/d1nzya_-d1on3a1.fasta

./group172/reference/d1nzya_-d1on3a2.fasta

./group172/reference/d1nzya_-d1tyfa_.fasta

./group172/reference/d1o8ua_-d1on3a1.fasta

./group172/reference/d1o8ua_-d1on3a2.fasta

./group172/reference/d1o8ua_-d1tyfa_.fasta

./group172/reference/d1on3a1-d1tyfa_.fasta

./group172/reference/d1on3a2-d1tyfa_.fasta

./group173/reference/d1cdza_-d1l0ba1.fasta

./group173/reference/d1dgtb3-d1l0ba1.fasta

./group173/reference/d1in1a_-d1l0ba1.fasta

./group175/reference/d1l9ga_-d1laue_.fasta

./group175/reference/d1l9ga_-d1oe4a_.fasta

./group175/reference/d1l9ga_-d3euga_.fasta

./group175/reference/d1laue_-d1muga_.fasta

./group175/reference/d1laue_-d1oe4a_.fasta

./group175/reference/d1muga_-d1oe4a_.fasta

./group175/reference/d1muga_-d3euga_.fasta

./group175/reference/d1oe4a_-d3euga_.fasta

./group176/reference/d1a04a2-d1m2fa_.fasta

./group176/reference/d1a04a2-d1ntr__.fasta

./group176/reference/d1a2oa1-d1m2fa_.fasta

./group176/reference/d1b00a_-d1m2fa_.fasta

./group176/reference/d1b00a_-d1qo0d_.fasta

./group176/reference/d1dbwa_-d1dz3a_.fasta

./group176/reference/d1dbwa_-d1m2fa_.fasta

./group176/reference/d1dbwa_-d1qo0d_.fasta

./group176/reference/d1dz3a_-d1m2fa_.fasta

./group176/reference/d1dz3a_-d1qo0d_.fasta

./group176/reference/d1m2fa_-d1nat__.fasta

./group176/reference/d1m2fa_-d1ntr__.fasta

./group176/reference/d1mvoa_-d1qo0d_.fasta

./group176/reference/d1nat__-d1qo0d_.fasta

./group176/reference/d1ntr__-d1qo0d_.fasta

./group176/reference/d1qo0d_-d1tmy__.fasta

./group177/reference/d1ag9a_-d1bvyf_.fasta

./group177/reference/d1ag9a_-d1d4aa_.fasta

./group177/reference/d1ag9a_-d1e5da1.fasta

./group177/reference/d1ag9a_-d1qr2a_.fasta

./group177/reference/d1bvyf_-d1e5da1.fasta

./group177/reference/d1bvyf_-d1fuea_.fasta

./group177/reference/d1bvyf_-d1oboa_.fasta

./group177/reference/d1bvyf_-d1qr2a_.fasta

./group177/reference/d1bvyf_-d2fcr__.fasta

./group177/reference/d1bvyf_-d5nul__.fasta

./group177/reference/d1d4aa_-d1e5da1.fasta

./group177/reference/d1d4aa_-d1f4pa_.fasta

./group177/reference/d1d4aa_-d1fuea_.fasta

./group177/reference/d1d4aa_-d1oboa_.fasta

./group177/reference/d1d4aa_-d5nul__.fasta

./group177/reference/d1e5da1-d1f4pa_.fasta

./group177/reference/d1e5da1-d1fuea_.fasta

./group177/reference/d1e5da1-d1ja1a2.fasta

./group177/reference/d1e5da1-d1oboa_.fasta

./group177/reference/d1e5da1-d1qr2a_.fasta

./group177/reference/d1e5da1-d2fcr__.fasta

./group177/reference/d1f4pa_-d1qr2a_.fasta

./group177/reference/d1fuea_-d1qr2a_.fasta

./group177/reference/d1fuea_-d5nul__.fasta

./group177/reference/d1ja1a2-d1qr2a_.fasta

./group177/reference/d1ja1a2-d2fcr__.fasta

./group177/reference/d1ja1a2-d5nul__.fasta

./group177/reference/d1oboa_-d1qr2a_.fasta

./group177/reference/d1qr2a_-d5nul__.fasta

./group178/reference/d1bmta2-d7reqa2.fasta

./group178/reference/d1bmta2-d7reqb2.fasta

./group178/reference/d1ccwa_-d7reqb2.fasta

./group178/reference/d7reqa2-d7reqb2.fasta

./group179/reference/d1esc__-d1k7ca_.fasta

./group181/reference/d1cf9a1-d1gpma2.fasta

./group181/reference/d1cf9a1-d1i7qb_.fasta

./group181/reference/d1cf9a1-d1ka9h_.fasta

./group181/reference/d1cf9a1-d1kwga3.fasta

./group181/reference/d1cf9a1-d1pe0a_.fasta

./group181/reference/d1cf9a1-d1qdlb_.fasta

./group181/reference/d1gpma2-d1kwga3.fasta

./group181/reference/d1gpma2-d1pe0a_.fasta

./group181/reference/d1i7qb_-d1k9vf_.fasta

./group181/reference/d1i7qb_-d1kwga3.fasta

./group181/reference/d1i7qb_-d1l9xa_.fasta

./group181/reference/d1i7qb_-d1pe0a_.fasta

./group181/reference/d1k9vf_-d1kwga3.fasta

./group181/reference/d1k9vf_-d1pe0a_.fasta

./group181/reference/d1ka9h_-d1kwga3.fasta

./group181/reference/d1ka9h_-d1l9xa_.fasta

./group181/reference/d1ka9h_-d1qdlb_.fasta

./group181/reference/d1kwga3-d1l9xa_.fasta

./group181/reference/d1kwga3-d1pe0a_.fasta

./group181/reference/d1kwga3-d1qdlb_.fasta

./group181/reference/d1l9xa_-d1pe0a_.fasta

./group181/reference/d1l9xa_-d1qdlb_.fasta

./group181/reference/d1pe0a_-d1qdlb_.fasta

./group182/reference/d1a8p_2-d1ep3b2.fasta

./group182/reference/d1a8p_2-d1gvha3.fasta

./group182/reference/d1a8p_2-d1i7pa2.fasta

./group182/reference/d1a8p_2-d1qfja2.fasta

./group182/reference/d1a8p_2-d2cnd_2.fasta

./group182/reference/d1a8p_2-d2pia_2.fasta

./group182/reference/d1cqxa3-d1ddga2.fasta

./group182/reference/d1cqxa3-d1ep3b2.fasta

./group182/reference/d1cqxa3-d1i7pa2.fasta

./group182/reference/d1cqxa3-d1ja1a3.fasta

./group182/reference/d1cqxa3-d1que_2.fasta

./group182/reference/d1ddga2-d1ep3b2.fasta

./group182/reference/d1ddga2-d1gvha3.fasta

./group182/reference/d1ddga2-d1qfja2.fasta

./group182/reference/d1ddga2-d2pia_2.fasta

./group182/reference/d1ep3b2-d1gvha3.fasta

./group182/reference/d1ep3b2-d1ja1a3.fasta

./group182/reference/d1ep3b2-d1krha2.fasta

./group182/reference/d1ep3b2-d1qfja2.fasta

./group182/reference/d1ep3b2-d1que_2.fasta

./group182/reference/d1ep3b2-d2cnd_2.fasta

./group182/reference/d1ep3b2-d2pia_2.fasta

./group182/reference/d1f20a2-d1fdr_2.fasta

./group182/reference/d1f20a2-d1gvha3.fasta

./group182/reference/d1f20a2-d1qfja2.fasta

./group182/reference/d1f20a2-d2cnd_2.fasta

./group182/reference/d1f20a2-d2pia_2.fasta

./group182/reference/d1fdr_2-d1gvha3.fasta

./group182/reference/d1fdr_2-d1ja1a3.fasta

./group182/reference/d1fdr_2-d1qfja2.fasta

./group182/reference/d1fdr_2-d2cnd_2.fasta

./group182/reference/d1fdr_2-d2pia_2.fasta

./group182/reference/d1gvha3-d1ja1a3.fasta

./group182/reference/d1gvha3-d1qfja2.fasta

./group182/reference/d1gvha3-d1que_2.fasta

./group182/reference/d1gvha3-d2cnd_2.fasta

./group182/reference/d1i7pa2-d1ja1a3.fasta

./group182/reference/d1i7pa2-d1qfja2.fasta

./group182/reference/d1ja1a3-d2cnd_2.fasta

./group182/reference/d1ja1a3-d2pia_2.fasta

./group182/reference/d1qfja2-d1que_2.fasta

./group182/reference/d1qfja2-d2pia_2.fasta

./group182/reference/d1que_2-d2pia_2.fasta

./group183/reference/d1a8h_2-d1coza_.fasta

./group183/reference/d1a8h_2-d1f7ua2.fasta

./group183/reference/d1a8h_2-d1g8fa2.fasta

./group183/reference/d1a8h_2-d1gtra2.fasta

./group183/reference/d1a8h_2-d1ihoa_.fasta

./group183/reference/d1a8h_2-d1j09a2.fasta

./group183/reference/d1a8h_2-d1jila_.fasta

./group183/reference/d1a8h_2-d1k4ma_.fasta

./group183/reference/d1a8h_2-d1n2ea_.fasta

./group183/reference/d1coza_-d1f7ua2.fasta

./group183/reference/d1coza_-d1g8fa2.fasta

./group183/reference/d1coza_-d1gtra2.fasta

./group183/reference/d1coza_-d1j09a2.fasta

./group183/reference/d1coza_-d1jhda2.fasta

./group183/reference/d1coza_-d1jila_.fasta

./group183/reference/d1coza_-d1n2ea_.fasta

./group183/reference/d1ej2a_-d1f7ua2.fasta

./group183/reference/d1ej2a_-d1g8fa2.fasta

./group183/reference/d1ej2a_-d1gtra2.fasta

./group183/reference/d1ej2a_-d1ihoa_.fasta

./group183/reference/d1ej2a_-d1j09a2.fasta

./group183/reference/d1ej2a_-d1jhda2.fasta

./group183/reference/d1ej2a_-d1jila_.fasta

./group183/reference/d1ej2a_-d1n2ea_.fasta

./group183/reference/d1ej2a_-d1qjca_.fasta

./group183/reference/d1f7ua2-d1g8fa2.fasta

./group183/reference/d1f7ua2-d1gtra2.fasta

./group183/reference/d1f7ua2-d1ihoa_.fasta

./group183/reference/d1f7ua2-d1j09a2.fasta

./group183/reference/d1f7ua2-d1jila_.fasta

./group183/reference/d1f7ua2-d1qjca_.fasta

./group183/reference/d1g8fa2-d1gtra2.fasta

./group183/reference/d1g8fa2-d1ihoa_.fasta

./group183/reference/d1g8fa2-d1jila_.fasta

./group183/reference/d1g8fa2-d1k4ma_.fasta

./group183/reference/d1g8fa2-d1n2ea_.fasta

./group183/reference/d1g8fa2-d1qjca_.fasta

./group183/reference/d1gtra2-d1ihoa_.fasta

./group183/reference/d1gtra2-d1jhda2.fasta

./group183/reference/d1gtra2-d1n2ea_.fasta

./group183/reference/d1gtra2-d1qjca_.fasta

./group183/reference/d1ihoa_-d1j09a2.fasta

./group183/reference/d1ihoa_-d1jhda2.fasta

./group183/reference/d1ihoa_-d1jila_.fasta

./group183/reference/d1ihoa_-d1k4ma_.fasta

./group183/reference/d1ihoa_-d1qjca_.fasta

./group183/reference/d1j09a2-d1jhda2.fasta

./group183/reference/d1j09a2-d1jila_.fasta

./group183/reference/d1j09a2-d1n2ea_.fasta

./group183/reference/d1j09a2-d1qjca_.fasta

./group183/reference/d1jhda2-d1k4ma_.fasta

./group183/reference/d1jila_-d1k4ma_.fasta

./group183/reference/d1jila_-d1n2ea_.fasta

./group183/reference/d1k4ma_-d1n2ea_.fasta

./group183/reference/d1k4ma_-d1qjca_.fasta

./group183/reference/d1n2ea_-d1qjca_.fasta

./group184/reference/d1efpa1-d1efvb_.fasta

./group184/reference/d1efpa1-d1jmva_.fasta

./group184/reference/d1efpa1-d1o97d1.fasta

./group184/reference/d1efpa1-d1sur__.fasta

./group184/reference/d1efva1-d1efvb_.fasta

./group184/reference/d1efva1-d1j20a1.fasta

./group184/reference/d1efva1-d1jmva_.fasta

./group184/reference/d1efvb_-d1j20a1.fasta

./group184/reference/d1efvb_-d1jmva_.fasta

./group184/reference/d1efvb_-d1k92a1.fasta

./group184/reference/d1efvb_-d1kqpa_.fasta

./group184/reference/d1efvb_-d1o97d1.fasta

./group184/reference/d1j20a1-d1jmva_.fasta

./group184/reference/d1j20a1-d1kqpa_.fasta

./group184/reference/d1j20a1-d1sur__.fasta

./group184/reference/d1jmva_-d1k92a1.fasta

./group184/reference/d1jmva_-d1o97d1.fasta

./group184/reference/d1jmva_-d1sur__.fasta

./group184/reference/d1k92a1-d1kqpa_.fasta

./group184/reference/d1k92a1-d1sur__.fasta

./group186/reference/d1a9xa3-d1b6ra2.fasta

./group186/reference/d1a9xa3-d1e4ea1.fasta

./group186/reference/d1a9xa3-d1ehia1.fasta

./group186/reference/d1a9xa3-d1gsa_1.fasta

./group186/reference/d1a9xa3-d1gsoa2.fasta

./group186/reference/d1a9xa3-d1iow_1.fasta

./group186/reference/d1a9xa3-d1kjqa2.fasta

./group186/reference/d1a9xa3-d2hgsa1.fasta

./group186/reference/d1a9xa4-d1b6ra2.fasta

./group186/reference/d1a9xa4-d1e4ea1.fasta

./group186/reference/d1a9xa4-d1ehia1.fasta

./group186/reference/d1a9xa4-d1gsa_1.fasta

./group186/reference/d1a9xa4-d1gsoa2.fasta

./group186/reference/d1a9xa4-d1i7na1.fasta

./group186/reference/d1a9xa4-d1kjqa2.fasta

./group186/reference/d1a9xa4-d1m0wa1.fasta

./group186/reference/d1a9xa4-d2hgsa1.fasta

./group186/reference/d1b6ra2-d1gsa_1.fasta

./group186/reference/d1b6ra2-d1gsoa2.fasta

./group186/reference/d1b6ra2-d1i7na1.fasta

./group186/reference/d1b6ra2-d1iow_1.fasta

./group186/reference/d1b6ra2-d2hgsa1.fasta

./group186/reference/d1e4ea1-d1gsoa2.fasta

./group186/reference/d1e4ea1-d1kjqa2.fasta

./group186/reference/d1e4ea1-d2hgsa1.fasta

./group186/reference/d1ehia1-d1kjqa2.fasta

./group186/reference/d1ehia1-d2hgsa1.fasta

./group186/reference/d1gsa_1-d1i7na1.fasta

./group186/reference/d1gsa_1-d1iow_1.fasta

./group186/reference/d1gsa_1-d1kjqa2.fasta

./group186/reference/d1gsa_1-d1m0wa1.fasta

./group186/reference/d1gsa_1-d2hgsa1.fasta

./group186/reference/d1gsoa2-d1iow_1.fasta

./group186/reference/d1i7na1-d1iow_1.fasta

./group186/reference/d1i7na1-d2hgsa1.fasta

./group186/reference/d1iow_1-d1kjqa2.fasta

./group186/reference/d1iow_1-d1m0wa1.fasta

./group186/reference/d1iow_1-d2hgsa1.fasta

./group186/reference/d1kjqa2-d1m0wa1.fasta

./group186/reference/d1kjqa2-d2hgsa1.fasta

./group187/reference/d1bfd_1-d1d4oa_.fasta

./group187/reference/d1bfd_1-d1dhs__.fasta

./group187/reference/d1bfd_1-d1efva2.fasta

./group187/reference/d1bfd_1-d1jsca1.fasta

./group187/reference/d1bfd_1-d1m2ka_.fasta

./group187/reference/d1bfd_1-d1o97d2.fasta

./group187/reference/d1bfd_1-d1ovma1.fasta

./group187/reference/d1bfd_1-d1pvda1.fasta

./group187/reference/d1bfd_1-d1zpda1.fasta

./group187/reference/d1d4oa_-d1dhs__.fasta

./group187/reference/d1d4oa_-d1jsca1.fasta

./group187/reference/d1d4oa_-d1o97d2.fasta

./group187/reference/d1d4oa_-d1ovma1.fasta

./group187/reference/d1d4oa_-d1pvda1.fasta

./group187/reference/d1d4oa_-d1zpda1.fasta

./group187/reference/d1dhs__-d1efva2.fasta

./group187/reference/d1dhs__-d1hzzc_.fasta

./group187/reference/d1dhs__-d1jsca1.fasta

./group187/reference/d1dhs__-d1m2ka_.fasta

./group187/reference/d1dhs__-d1o97d2.fasta

./group187/reference/d1dhs__-d1poxa1.fasta

./group187/reference/d1efva2-d1hzzc_.fasta

./group187/reference/d1efva2-d1m2ka_.fasta

./group187/reference/d1efva2-d1poxa1.fasta

./group187/reference/d1efva2-d1pvda1.fasta

./group187/reference/d1hzzc_-d1jsca1.fasta

./group187/reference/d1hzzc_-d1m2ka_.fasta

./group187/reference/d1hzzc_-d1o97d2.fasta

./group187/reference/d1hzzc_-d1pvda1.fasta

./group187/reference/d1hzzc_-d1zpda1.fasta

./group187/reference/d1jsca1-d1ovma1.fasta

./group187/reference/d1jsca1-d1zpda1.fasta

./group187/reference/d1m2ka_-d1ovma1.fasta

./group187/reference/d1m2ka_-d1poxa1.fasta

./group187/reference/d1m2ka_-d1zpda1.fasta

./group187/reference/d1o97d2-d1poxa1.fasta

./group187/reference/d1o97d2-d1pvda1.fasta

./group187/reference/d1o97d2-d1zpda1.fasta

./group187/reference/d1ovma1-d1poxa1.fasta

./group187/reference/d1poxa1-d1pvda1.fasta

./group187/reference/d1poxa1-d1zpda1.fasta

./group187/reference/d1pvda1-d1zpda1.fasta

./group188/reference/d1fsz_1-d1tuba1.fasta

./group188/reference/d1fsz_1-d1tubb1.fasta

./group188/reference/d1ofua1-d1tuba1.fasta

./group188/reference/d1ofua1-d1tubb1.fasta

./group188/reference/d1oi2a1-d1tuba1.fasta

./group189/reference/d1nbaa_-d1nf9a_.fasta

./group189/reference/d1nbaa_-d1yaca_.fasta

./group190/reference/d1bfd_2-d1bfd_3.fasta

./group190/reference/d1bfd_2-d1dtwb1.fasta

./group190/reference/d1bfd_2-d1gpua2.fasta

./group190/reference/d1bfd_2-d1keka1.fasta

./group190/reference/d1bfd_2-d1keka2.fasta

./group190/reference/d1bfd_2-d1ovma3.fasta

./group190/reference/d1bfd_2-d1poxa3.fasta

./group190/reference/d1bfd_2-d1pvda3.fasta

./group190/reference/d1bfd_2-d1qgda1.fasta

./group190/reference/d1bfd_2-d1qgda2.fasta

./group190/reference/d1bfd_2-d1zpda2.fasta

./group190/reference/d1bfd_2-d1zpda3.fasta

./group190/reference/d1bfd_3-d1dtwb1.fasta

./group190/reference/d1bfd_3-d1gpua2.fasta

./group190/reference/d1bfd_3-d1jsca2.fasta

./group190/reference/d1bfd_3-d1keka1.fasta

./group190/reference/d1bfd_3-d1keka2.fasta

./group190/reference/d1bfd_3-d1ovma2.fasta

./group190/reference/d1bfd_3-d1poxa2.fasta

./group190/reference/d1bfd_3-d1pvda3.fasta

./group190/reference/d1bfd_3-d1qgda1.fasta

./group190/reference/d1bfd_3-d1zpda2.fasta

./group190/reference/d1dtwb1-d1gpua2.fasta

./group190/reference/d1dtwb1-d1jsca2.fasta

./group190/reference/d1dtwb1-d1ovma2.fasta

./group190/reference/d1dtwb1-d1pvda2.fasta

./group190/reference/d1dtwb1-d1pvda3.fasta

./group190/reference/d1dtwb1-d1qgda1.fasta

./group190/reference/d1dtwb1-d1qgda2.fasta

./group190/reference/d1dtwb1-d1zpda2.fasta

./group190/reference/d1dtwb1-d1zpda3.fasta

./group190/reference/d1gpua2-d1ovma2.fasta

./group190/reference/d1gpua2-d1ovma3.fasta

./group190/reference/d1gpua2-d1poxa3.fasta

./group190/reference/d1gpua2-d1pvda2.fasta

./group190/reference/d1gpua2-d1qgda2.fasta

./group190/reference/d1gpua2-d1zpda2.fasta

./group190/reference/d1gpua2-d1zpda3.fasta

./group190/reference/d1jsca2-d1keka1.fasta

./group190/reference/d1jsca2-d1keka2.fasta

./group190/reference/d1jsca2-d1ovma3.fasta

./group190/reference/d1jsca2-d1poxa3.fasta

./group190/reference/d1jsca2-d1pvda3.fasta

./group190/reference/d1jsca2-d1qgda1.fasta

./group190/reference/d1jsca2-d1zpda3.fasta

./group190/reference/d1keka1-d1ovma2.fasta

./group190/reference/d1keka1-d1ovma3.fasta

./group190/reference/d1keka1-d1pvda2.fasta

./group190/reference/d1keka1-d1pvda3.fasta

./group190/reference/d1keka1-d1qgda1.fasta

./group190/reference/d1keka1-d1zpda2.fasta

./group190/reference/d1keka1-d1zpda3.fasta

./group190/reference/d1keka2-d1ovma3.fasta

./group190/reference/d1keka2-d1poxa2.fasta

./group190/reference/d1keka2-d1poxa3.fasta

./group190/reference/d1keka2-d1pvda2.fasta

./group190/reference/d1keka2-d1pvda3.fasta

./group190/reference/d1keka2-d1qgda2.fasta

./group190/reference/d1keka2-d1zpda3.fasta

./group190/reference/d1ovma2-d1ovma3.fasta

./group190/reference/d1ovma2-d1poxa2.fasta

./group190/reference/d1ovma2-d1qgda2.fasta

./group190/reference/d1ovma2-d1zpda3.fasta

./group190/reference/d1ovma3-d1qgda1.fasta

./group190/reference/d1ovma3-d1qgda2.fasta

./group190/reference/d1poxa2-d1poxa3.fasta

./group190/reference/d1poxa2-d1zpda2.fasta

./group190/reference/d1poxa2-d1zpda3.fasta

./group190/reference/d1poxa3-d1pvda2.fasta

./group190/reference/d1poxa3-d1pvda3.fasta

./group190/reference/d1poxa3-d1qgda2.fasta

./group190/reference/d1poxa3-d1zpda2.fasta

./group190/reference/d1pvda2-d1qgda1.fasta

./group190/reference/d1pvda2-d1zpda3.fasta

./group190/reference/d1pvda3-d1qgda2.fasta

./group190/reference/d1pvda3-d1zpda2.fasta

./group190/reference/d1qgda1-d1qgda2.fasta

./group190/reference/d1qgda1-d1zpda3.fasta

./group190/reference/d1qgda2-d1zpda2.fasta

./group190/reference/d1zpda2-d1zpda3.fasta

./group191/reference/d1bg2__-d1cp2a_.fasta

./group191/reference/d1bg2__-d1d2na_.fasta

./group191/reference/d1bg2__-d1e79a3.fasta

./group191/reference/d1bg2__-d1eg7a_.fasta

./group191/reference/d1bg2__-d1fmja_.fasta

./group191/reference/d1bg2__-d1fnna2.fasta

./group191/reference/d1bg2__-d1g2912.fasta

./group191/reference/d1bg2__-d1jj7a_.fasta

./group191/reference/d1bg2__-d1mt0a_.fasta

./group191/reference/d1bg2__-d1n25a_.fasta

./group191/reference/d1bg2__-d1njfa_.fasta

./group191/reference/d1bg2__-d1ofha_.fasta

./group191/reference/d1bg2__-d1qf5a_.fasta

./group191/reference/d1bg2__-d1uaaa1.fasta

./group191/reference/d1cp2a_-d1d2na_.fasta

./group191/reference/d1cp2a_-d1dj3a_.fasta

./group191/reference/d1cp2a_-d1e32a2.fasta

./group191/reference/d1cp2a_-d1e79a3.fasta

./group191/reference/d1cp2a_-d1fnna2.fasta

./group191/reference/d1cp2a_-d1g2912.fasta

./group191/reference/d1cp2a_-d1g3qa_.fasta

./group191/reference/d1cp2a_-d1g6ha_.fasta

./group191/reference/d1cp2a_-d1iwea_.fasta

./group191/reference/d1cp2a_-d1jj7a_.fasta

./group191/reference/d1cp2a_-d1n25a_.fasta

./group191/reference/d1cp2a_-d1njfa_.fasta

./group191/reference/d1cp2a_-d1ofha_.fasta

./group191/reference/d1cp2a_-d1oxsc2.fasta

./group191/reference/d1cp2a_-d1qf5a_.fasta

./group191/reference/d1cp2a_-d1uaaa1.fasta

./group191/reference/d1d2na_-d1dj3a_.fasta

./group191/reference/d1d2na_-d1e32a2.fasta

./group191/reference/d1d2na_-d1e79a3.fasta

./group191/reference/d1d2na_-d1f60a3.fasta

./group191/reference/d1d2na_-d1fmja_.fasta

./group191/reference/d1d2na_-d1fnna2.fasta

./group191/reference/d1d2na_-d1g2912.fasta

./group191/reference/d1d2na_-d1g6ha_.fasta

./group191/reference/d1d2na_-d1hyqa_.fasta

./group191/reference/d1d2na_-d1j99a_.fasta

./group191/reference/d1d2na_-d1jj7a_.fasta

./group191/reference/d1d2na_-d1mt0a_.fasta

./group191/reference/d1d2na_-d1ofha_.fasta

./group191/reference/d1d2na_-d1oxsc2.fasta

./group191/reference/d1d2na_-d1pf4a1.fasta

./group191/reference/d1d2na_-d1qf5a_.fasta

./group191/reference/d1d2na_-d1uaaa1.fasta

./group191/reference/d1dj3a_-d1e32a2.fasta

./group191/reference/d1dj3a_-d1eg7a_.fasta

./group191/reference/d1dj3a_-d1f60a3.fasta

./group191/reference/d1dj3a_-d1fnna2.fasta

./group191/reference/d1dj3a_-d1g2912.fasta

./group191/reference/d1dj3a_-d1g3qa_.fasta

./group191/reference/d1dj3a_-d1g6ha_.fasta

./group191/reference/d1dj3a_-d1hyqa_.fasta

./group191/reference/d1dj3a_-d1j99a_.fasta

./group191/reference/d1dj3a_-d1jj7a_.fasta

./group191/reference/d1dj3a_-d1mt0a_.fasta

./group191/reference/d1dj3a_-d1n25a_.fasta

./group191/reference/d1dj3a_-d1njfa_.fasta

./group191/reference/d1dj3a_-d1ofha_.fasta

./group191/reference/d1dj3a_-d1oxsc2.fasta

./group191/reference/d1dj3a_-d1pf4a1.fasta

./group191/reference/d1dj3a_-d1uaaa1.fasta

./group191/reference/d1e32a2-d1e79a3.fasta

./group191/reference/d1e32a2-d1eg7a_.fasta

./group191/reference/d1e32a2-d1f60a3.fasta

./group191/reference/d1e32a2-d1fmja_.fasta

./group191/reference/d1e32a2-d1fnna2.fasta

./group191/reference/d1e32a2-d1g3qa_.fasta

./group191/reference/d1e32a2-d1g6ha_.fasta

./group191/reference/d1e32a2-d1hyqa_.fasta

./group191/reference/d1e32a2-d1jj7a_.fasta

./group191/reference/d1e32a2-d1mt0a_.fasta

./group191/reference/d1e32a2-d1n25a_.fasta

./group191/reference/d1e32a2-d1oxsc2.fasta

./group191/reference/d1e32a2-d1pf4a1.fasta

./group191/reference/d1e32a2-d1qf5a_.fasta

./group191/reference/d1e32a2-d1uaaa1.fasta

./group191/reference/d1e79a3-d1fmja_.fasta

./group191/reference/d1e79a3-d1fnna2.fasta

./group191/reference/d1e79a3-d1g2912.fasta

./group191/reference/d1e79a3-d1g3qa_.fasta

./group191/reference/d1e79a3-d1g6ha_.fasta

./group191/reference/d1e79a3-d1hyqa_.fasta

./group191/reference/d1e79a3-d1j99a_.fasta

./group191/reference/d1e79a3-d1jj7a_.fasta

./group191/reference/d1e79a3-d1mt0a_.fasta

./group191/reference/d1e79a3-d1n25a_.fasta

./group191/reference/d1e79a3-d1njfa_.fasta

./group191/reference/d1e79a3-d1oxsc2.fasta

./group191/reference/d1e79a3-d1uaaa1.fasta

./group191/reference/d1eg7a_-d1f60a3.fasta

./group191/reference/d1eg7a_-d1fnna2.fasta

./group191/reference/d1eg7a_-d1g2912.fasta

./group191/reference/d1eg7a_-d1g3qa_.fasta

./group191/reference/d1eg7a_-d1g6ha_.fasta

./group191/reference/d1eg7a_-d1hyqa_.fasta

./group191/reference/d1eg7a_-d1iwea_.fasta

./group191/reference/d1eg7a_-d1j99a_.fasta

./group191/reference/d1eg7a_-d1jj7a_.fasta

./group191/reference/d1eg7a_-d1mt0a_.fasta

./group191/reference/d1eg7a_-d1n25a_.fasta

./group191/reference/d1eg7a_-d1njfa_.fasta

./group191/reference/d1eg7a_-d1oxsc2.fasta

./group191/reference/d1eg7a_-d1qf5a_.fasta

./group191/reference/d1eg7a_-d1uaaa1.fasta

./group191/reference/d1f60a3-d1fnna2.fasta

./group191/reference/d1f60a3-d1g2912.fasta

./group191/reference/d1f60a3-d1g3qa_.fasta

./group191/reference/d1f60a3-d1iwea_.fasta

./group191/reference/d1f60a3-d1mt0a_.fasta

./group191/reference/d1f60a3-d1ofha_.fasta

./group191/reference/d1f60a3-d1oxsc2.fasta

./group191/reference/d1f60a3-d1pf4a1.fasta

./group191/reference/d1fmja_-d1g2912.fasta

./group191/reference/d1fmja_-d1g3qa_.fasta

./group191/reference/d1fmja_-d1iwea_.fasta

./group191/reference/d1fmja_-d1jj7a_.fasta

./group191/reference/d1fmja_-d1njfa_.fasta

./group191/reference/d1fmja_-d1ofha_.fasta

./group191/reference/d1fmja_-d1oxsc2.fasta

./group191/reference/d1fmja_-d1qf5a_.fasta

./group191/reference/d1fmja_-d1uaaa1.fasta

./group191/reference/d1fnna2-d1g2912.fasta

./group191/reference/d1fnna2-d1g3qa_.fasta

./group191/reference/d1fnna2-d1g6ha_.fasta

./group191/reference/d1fnna2-d1hyqa_.fasta

./group191/reference/d1fnna2-d1iwea_.fasta

./group191/reference/d1fnna2-d1j99a_.fasta

./group191/reference/d1fnna2-d1jj7a_.fasta

./group191/reference/d1fnna2-d1mt0a_.fasta

./group191/reference/d1fnna2-d1n25a_.fasta

./group191/reference/d1fnna2-d1njfa_.fasta

./group191/reference/d1fnna2-d1ofha_.fasta

./group191/reference/d1fnna2-d1oxsc2.fasta

./group191/reference/d1fnna2-d1qf5a_.fasta

./group191/reference/d1fnna2-d1uaaa1.fasta

./group191/reference/d1g2912-d1g3qa_.fasta

./group191/reference/d1g2912-d1hyqa_.fasta

./group191/reference/d1g2912-d1n25a_.fasta

./group191/reference/d1g2912-d1njfa_.fasta

./group191/reference/d1g2912-d1ofha_.fasta

./group191/reference/d1g2912-d1qf5a_.fasta

./group191/reference/d1g3qa_-d1g6ha_.fasta

./group191/reference/d1g3qa_-d1iwea_.fasta

./group191/reference/d1g3qa_-d1jj7a_.fasta

./group191/reference/d1g3qa_-d1mt0a_.fasta

./group191/reference/d1g3qa_-d1n25a_.fasta

./group191/reference/d1g3qa_-d1njfa_.fasta

./group191/reference/d1g3qa_-d1ofha_.fasta

./group191/reference/d1g3qa_-d1oxsc2.fasta

./group191/reference/d1g3qa_-d1qf5a_.fasta

./group191/reference/d1g6ha_-d1hyqa_.fasta

./group191/reference/d1g6ha_-d1iwea_.fasta

./group191/reference/d1g6ha_-d1n25a_.fasta

./group191/reference/d1g6ha_-d1njfa_.fasta

./group191/reference/d1g6ha_-d1ofha_.fasta

./group191/reference/d1g6ha_-d1qf5a_.fasta

./group191/reference/d1g6ha_-d1uaaa1.fasta

./group191/reference/d1hyqa_-d1iwea_.fasta

./group191/reference/d1hyqa_-d1jj7a_.fasta

./group191/reference/d1hyqa_-d1mt0a_.fasta

./group191/reference/d1hyqa_-d1n25a_.fasta

./group191/reference/d1hyqa_-d1njfa_.fasta

./group191/reference/d1hyqa_-d1ofha_.fasta

./group191/reference/d1hyqa_-d1oxsc2.fasta

./group191/reference/d1hyqa_-d1qf5a_.fasta

./group191/reference/d1hyqa_-d1uaaa1.fasta

./group191/reference/d1iwea_-d1j99a_.fasta

./group191/reference/d1iwea_-d1jj7a_.fasta

./group191/reference/d1iwea_-d1mt0a_.fasta

./group191/reference/d1iwea_-d1n25a_.fasta

./group191/reference/d1iwea_-d1njfa_.fasta

./group191/reference/d1iwea_-d1ofha_.fasta

./group191/reference/d1iwea_-d1oxsc2.fasta

./group191/reference/d1iwea_-d1uaaa1.fasta

./group191/reference/d1j99a_-d1jj7a_.fasta

./group191/reference/d1j99a_-d1mt0a_.fasta

./group191/reference/d1j99a_-d1n25a_.fasta

./group191/reference/d1j99a_-d1njfa_.fasta

./group191/reference/d1j99a_-d1qf5a_.fasta

./group191/reference/d1j99a_-d1uaaa1.fasta

./group191/reference/d1jj7a_-d1n25a_.fasta

./group191/reference/d1jj7a_-d1njfa_.fasta

./group191/reference/d1jj7a_-d1ofha_.fasta

./group191/reference/d1mt0a_-d1n25a_.fasta

./group191/reference/d1mt0a_-d1njfa_.fasta

./group191/reference/d1mt0a_-d1ofha_.fasta

./group191/reference/d1mt0a_-d1qf5a_.fasta

./group191/reference/d1mt0a_-d1uaaa1.fasta

./group191/reference/d1n25a_-d1njfa_.fasta

./group191/reference/d1n25a_-d1ofha_.fasta

./group191/reference/d1n25a_-d1oxsc2.fasta

./group191/reference/d1n25a_-d1qf5a_.fasta

./group191/reference/d1n25a_-d1uaaa1.fasta

./group191/reference/d1njfa_-d1oxsc2.fasta

./group191/reference/d1njfa_-d1pf4a1.fasta

./group191/reference/d1njfa_-d1uaaa1.fasta

./group191/reference/d1ofha_-d1oxsc2.fasta

./group191/reference/d1ofha_-d1qf5a_.fasta

./group191/reference/d1ofha_-d1uaaa1.fasta

./group191/reference/d1oxsc2-d1pf4a1.fasta

./group191/reference/d1oxsc2-d1qf5a_.fasta

./group191/reference/d1pf4a1-d1qf5a_.fasta

./group191/reference/d1qf5a_-d1uaaa1.fasta

./group192/reference/d1ga6a_-d1ic6a_.fasta

./group192/reference/d1ga6a_-d1ot5a2.fasta

./group192/reference/d1ga6a_-d1p8ja2.fasta

./group193/reference/d1c3pa_-d2ceva_.fasta

./group194/reference/d1b5sa_-d1l5aa1.fasta

./group194/reference/d1b5sa_-d1l5aa2.fasta

./group194/reference/d1b5sa_-d1nocb_.fasta

./group194/reference/d1b5sa_-d3cla__.fasta

./group194/reference/d1eaf__-d1l5aa1.fasta

./group194/reference/d1eaf__-d1l5aa2.fasta

./group194/reference/d1eaf__-d1nocb_.fasta

./group194/reference/d1l5aa1-d1l5aa2.fasta

./group194/reference/d1l5aa1-d1nocb_.fasta

./group194/reference/d1l5aa1-d3cla__.fasta

./group194/reference/d1l5aa2-d1nocb_.fasta

./group194/reference/d1l5aa2-d3cla__.fasta

./group195/reference/d1jf8a_-d1phr__.fasta

./group196/reference/d1d5ra2-d1eeoa_.fasta

./group196/reference/d1d5ra2-d1fpza_.fasta

./group196/reference/d1d5ra2-d1ikza_.fasta

./group196/reference/d1d5ra2-d1jlna_.fasta

./group196/reference/d1d5ra2-d1lara1.fasta

./group196/reference/d1d5ra2-d1mkp__.fasta

./group196/reference/d1d5ra2-d1vhra_.fasta

./group196/reference/d1eeoa_-d1fpza_.fasta

./group196/reference/d1eeoa_-d1ikza_.fasta

./group196/reference/d1eeoa_-d1lyva_.fasta

./group196/reference/d1eeoa_-d1mkp__.fasta

./group196/reference/d1eeoa_-d1vhra_.fasta

./group196/reference/d1fpza_-d1ikza_.fasta

./group196/reference/d1fpza_-d1jlna_.fasta

./group196/reference/d1fpza_-d1lara1.fasta

./group196/reference/d1fpza_-d1lara2.fasta

./group196/reference/d1fpza_-d1lyva_.fasta

./group196/reference/d1fpza_-d1ohea1.fasta

./group196/reference/d1fpza_-d1vhra_.fasta

./group196/reference/d1ikza_-d1lara1.fasta

./group196/reference/d1ikza_-d1lara2.fasta

./group196/reference/d1ikza_-d1lyva_.fasta

./group196/reference/d1ikza_-d1ohea1.fasta

./group196/reference/d1jlna_-d1mkp__.fasta

./group196/reference/d1jlna_-d1vhra_.fasta

./group196/reference/d1lara1-d1vhra_.fasta

./group196/reference/d1lara2-d1mkp__.fasta

./group196/reference/d1lara2-d1vhra_.fasta

./group196/reference/d1lyva_-d1mkp__.fasta

./group196/reference/d1lyva_-d1ohea1.fasta

./group196/reference/d1lyva_-d1vhra_.fasta

./group196/reference/d1ohea1-d1vhra_.fasta

./group197/reference/d1hzma_-d1rhs_1.fasta

./group198/reference/d1a8l_1-d1a8y_2.fasta

./group198/reference/d1a8l_1-d1bjx__.fasta

./group198/reference/d1a8l_1-d1eeja1.fasta

./group198/reference/d1a8l_1-d1ep7a_.fasta

./group198/reference/d1a8l_1-d1erv__.fasta

./group198/reference/d1a8l_1-d1f9ma_.fasta

./group198/reference/d1a8l_1-d1fb6a_.fasta

./group198/reference/d1a8l_1-d1gh2a_.fasta

./group198/reference/d1a8l_1-d1jfua_.fasta

./group198/reference/d1a8l_1-d1knga_.fasta

./group198/reference/d1a8l_1-d1kte__.fasta

./group198/reference/d1a8l_1-d1m2da_.fasta

./group198/reference/d1a8l_1-d1mek__.fasta

./group198/reference/d1a8l_1-d1n8ja_.fasta

./group198/reference/d1a8l_1-d1quwa_.fasta

./group198/reference/d1a8l_1-d1thx__.fasta

./group198/reference/d1a8l_1-d2trxa_.fasta

./group198/reference/d1a8l_2-d1a8y_1.fasta

./group198/reference/d1a8l_2-d1bjx__.fasta

./group198/reference/d1a8l_2-d1eema2.fasta

./group198/reference/d1a8l_2-d1ep7a_.fasta

./group198/reference/d1a8l_2-d1erv__.fasta

./group198/reference/d1a8l_2-d1foha3.fasta

./group198/reference/d1a8l_2-d1g7ea_.fasta

./group198/reference/d1a8l_2-d1gh2a_.fasta

./group198/reference/d1a8l_2-d1hyua3.fasta

./group198/reference/d1a8l_2-d1knga_.fasta

./group198/reference/d1a8l_2-d1m2da_.fasta

./group198/reference/d1a8l_2-d1n8ja_.fasta

./group198/reference/d1a8l_2-d1quwa_.fasta

./group198/reference/d1a8l_2-d1thx__.fasta

./group198/reference/d1a8y_1-d1a8y_2.fasta

./group198/reference/d1a8y_1-d1bjx__.fasta

./group198/reference/d1a8y_1-d1erv__.fasta

./group198/reference/d1a8y_1-d1fb6a_.fasta

./group198/reference/d1a8y_1-d1g7ea_.fasta

./group198/reference/d1a8y_1-d1gh2a_.fasta

./group198/reference/d1a8y_1-d1hd2a_.fasta

./group198/reference/d1a8y_1-d1hyua3.fasta

./group198/reference/d1a8y_1-d1knga_.fasta

./group198/reference/d1a8y_1-d1kte__.fasta

./group198/reference/d1a8y_1-d1n8ja_.fasta

./group198/reference/d1a8y_1-d1quwa_.fasta

./group198/reference/d1a8y_1-d1thx__.fasta

./group198/reference/d1a8y_2-d1ep7a_.fasta

./group198/reference/d1a8y_2-d1erv__.fasta

./group198/reference/d1a8y_2-d1f9ma_.fasta

./group198/reference/d1a8y_2-d1g7ea_.fasta

./group198/reference/d1a8y_2-d1hyua3.fasta

./group198/reference/d1a8y_2-d1jfua_.fasta

./group198/reference/d1a8y_2-d1m2da_.fasta

./group198/reference/d1a8y_2-d1mek__.fasta

./group198/reference/d1a8y_2-d1thx__.fasta

./group198/reference/d1bjx__-d1eema2.fasta

./group198/reference/d1bjx__-d1ep7a_.fasta

./group198/reference/d1bjx__-d1erv__.fasta

./group198/reference/d1bjx__-d1f9ma_.fasta

./group198/reference/d1bjx__-d1fb6a_.fasta

./group198/reference/d1bjx__-d1foha3.fasta

./group198/reference/d1bjx__-d1gh2a_.fasta

./group198/reference/d1bjx__-d1hd2a_.fasta

./group198/reference/d1bjx__-d1hyua3.fasta

./group198/reference/d1bjx__-d1kte__.fasta

./group198/reference/d1bjx__-d1m2da_.fasta

./group198/reference/d1bjx__-d1mek__.fasta

./group198/reference/d1bjx__-d1n8ja_.fasta

./group198/reference/d1bjx__-d1quwa_.fasta

./group198/reference/d1bjx__-d1thx__.fasta

./group198/reference/d1bjx__-d2trxa_.fasta

./group198/reference/d1eeja1-d1eema2.fasta

./group198/reference/d1eeja1-d1ep7a_.fasta

./group198/reference/d1eeja1-d1f9ma_.fasta

./group198/reference/d1eeja1-d1fb6a_.fasta

./group198/reference/d1eeja1-d1foha3.fasta

./group198/reference/d1eeja1-d1g7ea_.fasta

./group198/reference/d1eeja1-d1gh2a_.fasta

./group198/reference/d1eeja1-d1hd2a_.fasta

./group198/reference/d1eeja1-d1hyua3.fasta

./group198/reference/d1eeja1-d1jfua_.fasta

./group198/reference/d1eeja1-d1knga_.fasta

./group198/reference/d1eeja1-d1m2da_.fasta

./group198/reference/d1eeja1-d1mek__.fasta

./group198/reference/d1eeja1-d1quwa_.fasta

./group198/reference/d1eema2-d1foha3.fasta

./group198/reference/d1eema2-d1knga_.fasta

./group198/reference/d1eema2-d1kte__.fasta

./group198/reference/d1eema2-d1thx__.fasta

./group198/reference/d1eema2-d2trxa_.fasta

./group198/reference/d1ep7a_-d1foha3.fasta

./group198/reference/d1ep7a_-d1g7ea_.fasta

./group198/reference/d1ep7a_-d1hd2a_.fasta

./group198/reference/d1ep7a_-d1hyua3.fasta

./group198/reference/d1ep7a_-d1m2da_.fasta

./group198/reference/d1ep7a_-d1n8ja_.fasta

./group198/reference/d1erv__-d1g7ea_.fasta

./group198/reference/d1erv__-d1hd2a_.fasta

./group198/reference/d1erv__-d1hyua3.fasta

./group198/reference/d1erv__-d1knga_.fasta

./group198/reference/d1erv__-d1kte__.fasta

./group198/reference/d1erv__-d1n8ja_.fasta

./group198/reference/d1f9ma_-d1g7ea_.fasta

./group198/reference/d1f9ma_-d1hd2a_.fasta

./group198/reference/d1f9ma_-d1hyua3.fasta

./group198/reference/d1f9ma_-d1jfua_.fasta

./group198/reference/d1f9ma_-d1kte__.fasta

./group198/reference/d1f9ma_-d1n8ja_.fasta

./group198/reference/d1fb6a_-d1foha3.fasta

./group198/reference/d1fb6a_-d1g7ea_.fasta

./group198/reference/d1fb6a_-d1hd2a_.fasta

./group198/reference/d1fb6a_-d1hyua3.fasta

./group198/reference/d1fb6a_-d1knga_.fasta

./group198/reference/d1fb6a_-d1kte__.fasta

./group198/reference/d1fb6a_-d1n8ja_.fasta

./group198/reference/d1foha3-d1g7ea_.fasta

./group198/reference/d1foha3-d1hd2a_.fasta

./group198/reference/d1foha3-d1jfua_.fasta

./group198/reference/d1foha3-d1knga_.fasta

./group198/reference/d1foha3-d1mek__.fasta

./group198/reference/d1foha3-d1n8ja_.fasta

./group198/reference/d1foha3-d1thx__.fasta

./group198/reference/d1g7ea_-d1gh2a_.fasta

./group198/reference/d1g7ea_-d1hd2a_.fasta

./group198/reference/d1g7ea_-d1hyua3.fasta

./group198/reference/d1g7ea_-d1jfua_.fasta

./group198/reference/d1g7ea_-d1kte__.fasta

./group198/reference/d1g7ea_-d1m2da_.fasta

./group198/reference/d1g7ea_-d1thx__.fasta

./group198/reference/d1gh2a_-d1jfua_.fasta

./group198/reference/d1gh2a_-d1knga_.fasta

./group198/reference/d1gh2a_-d1kte__.fasta

./group198/reference/d1gh2a_-d1m2da_.fasta

./group198/reference/d1gh2a_-d1n8ja_.fasta

./group198/reference/d1hd2a_-d1hyua3.fasta

./group198/reference/d1hd2a_-d1jfua_.fasta

./group198/reference/d1hd2a_-d1knga_.fasta

./group198/reference/d1hd2a_-d1m2da_.fasta

./group198/reference/d1hd2a_-d1mek__.fasta

./group198/reference/d1hd2a_-d1quwa_.fasta

./group198/reference/d1hd2a_-d1thx__.fasta

./group198/reference/d1hd2a_-d2trxa_.fasta

./group198/reference/d1hyua3-d1jfua_.fasta

./group198/reference/d1hyua3-d1knga_.fasta

./group198/reference/d1hyua3-d1kte__.fasta

./group198/reference/d1hyua3-d1m2da_.fasta

./group198/reference/d1hyua3-d1n8ja_.fasta

./group198/reference/d1hyua3-d1quwa_.fasta

./group198/reference/d1jfua_-d1kte__.fasta

./group198/reference/d1jfua_-d1mek__.fasta

./group198/reference/d1jfua_-d1n8ja_.fasta

./group198/reference/d1knga_-d1kte__.fasta

./group198/reference/d1knga_-d1mek__.fasta

./group198/reference/d1knga_-d1n8ja_.fasta

./group198/reference/d1kte__-d1m2da_.fasta

./group198/reference/d1kte__-d1mek__.fasta

./group198/reference/d1kte__-d1quwa_.fasta

./group198/reference/d1kte__-d1thx__.fasta

./group198/reference/d1kte__-d2trxa_.fasta

./group198/reference/d1mek__-d1n8ja_.fasta

./group198/reference/d1n8ja_-d1thx__.fasta

./group198/reference/d1n8ja_-d2trxa_.fasta

./group199/reference/d1dtwb2-d1gpua3.fasta

./group199/reference/d1dtwb2-d1keka3.fasta

./group199/reference/d1dtwb2-d1l8aa3.fasta

./group199/reference/d1gpua3-d1keka3.fasta

./group199/reference/d1itza3-d1keka3.fasta

./group199/reference/d1keka3-d1l8aa3.fasta

./group199/reference/d1keka3-d1qgda3.fasta

./group201/reference/d1gyta1-d1lam_1.fasta

./group201/reference/d1hjza_-d1lam_1.fasta

./group202/reference/d1atia1-d1qe0a1.fasta

./group202/reference/d1h4vb1-d1nj1a1.fasta

./group202/reference/d1h4vb1-d1nj8a1.fasta

./group202/reference/d1h4vb1-d1qf6a1.fasta

./group202/reference/d1hc7a1-d1kmma1.fasta

./group202/reference/d1hc7a1-d1qe0a1.fasta

./group202/reference/d1kmma1-d1nj1a1.fasta

./group202/reference/d1kmma1-d1nj8a1.fasta

./group202/reference/d1kmma1-d1qf6a1.fasta

./group202/reference/d1nj1a1-d1qe0a1.fasta

./group202/reference/d1nj8a1-d1qe0a1.fasta

./group202/reference/d1qe0a1-d1qf6a1.fasta

./group203/reference/d1avqa_-d1cfr__.fasta

./group203/reference/d1avqa_-d1d02a_.fasta

./group203/reference/d1avqa_-d1dmua_.fasta

./group203/reference/d1avqa_-d1f1za2.fasta

./group203/reference/d1avqa_-d1gefa_.fasta

./group203/reference/d1avqa_-d1j23a_.fasta

./group203/reference/d1avqa_-d1knva_.fasta

./group203/reference/d1avqa_-d1m0da_.fasta

./group203/reference/d1avqa_-d2foka4.fasta

./group203/reference/d1avqa_-d3pvia_.fasta

./group203/reference/d1cfr__-d1d02a_.fasta

./group203/reference/d1cfr__-d1dmua_.fasta

./group203/reference/d1cfr__-d1f1za2.fasta

./group203/reference/d1cfr__-d1fiua_.fasta

./group203/reference/d1cfr__-d1gefa_.fasta

./group203/reference/d1cfr__-d1j23a_.fasta

./group203/reference/d1cfr__-d1m0da_.fasta

./group203/reference/d1cfr__-d1vsra_.fasta

./group203/reference/d1cfr__-d2foka4.fasta

./group203/reference/d1ckqa_-d1dmua_.fasta

./group203/reference/d1ckqa_-d1f1za2.fasta

./group203/reference/d1ckqa_-d1fiua_.fasta

./group203/reference/d1ckqa_-d1gefa_.fasta

./group203/reference/d1ckqa_-d1j23a_.fasta

./group203/reference/d1ckqa_-d1vsra_.fasta

./group203/reference/d1ckqa_-d2foka4.fasta

./group203/reference/d1d02a_-d1dmua_.fasta

./group203/reference/d1d02a_-d1fiua_.fasta

./group203/reference/d1d02a_-d1j23a_.fasta

./group203/reference/d1d02a_-d1m0da_.fasta

./group203/reference/d1d02a_-d1vsra_.fasta

./group203/reference/d1d02a_-d2foka4.fasta

./group203/reference/d1d02a_-d3pvia_.fasta

./group203/reference/d1dmua_-d1f1za2.fasta

./group203/reference/d1dmua_-d1j23a_.fasta

./group203/reference/d1dmua_-d1knva_.fasta

./group203/reference/d1dmua_-d1m0da_.fasta

./group203/reference/d1dmua_-d1vsra_.fasta

./group203/reference/d1dmua_-d2foka4.fasta

./group203/reference/d1f1za2-d1fiua_.fasta

./group203/reference/d1f1za2-d1gefa_.fasta

./group203/reference/d1f1za2-d1j23a_.fasta

./group203/reference/d1f1za2-d2foka4.fasta

./group203/reference/d1f1za2-d3pvia_.fasta

./group203/reference/d1fiua_-d1j23a_.fasta

./group203/reference/d1fiua_-d1knva_.fasta

./group203/reference/d1fiua_-d1m0da_.fasta

./group203/reference/d1fiua_-d1vsra_.fasta

./group203/reference/d1fiua_-d2foka4.fasta

./group203/reference/d1gefa_-d1j23a_.fasta

./group203/reference/d1gefa_-d1knva_.fasta

./group203/reference/d1gefa_-d1m0da_.fasta

./group203/reference/d1j23a_-d1knva_.fasta

./group203/reference/d1j23a_-d1vsra_.fasta

./group203/reference/d1j23a_-d2foka4.fasta

./group203/reference/d1knva_-d1m0da_.fasta

./group203/reference/d1knva_-d2foka4.fasta

./group203/reference/d1m0da_-d2foka4.fasta

./group203/reference/d1vsra_-d2foka4.fasta

./group205/reference/d1bdg_2-d1bu6o2.fasta

./group205/reference/d1bdg_2-d1bupa1.fasta

./group205/reference/d1bdg_2-d1bupa2.fasta

./group205/reference/d1bdg_2-d1e4ft1.fasta

./group205/reference/d1bdg_2-d1g99a2.fasta

./group205/reference/d1bdg_2-d1huxa_.fasta

./group205/reference/d1bdg_2-d1j6za2.fasta

./group205/reference/d1bdg_2-d1jcfa1.fasta

./group205/reference/d1bdg_2-d1nbwa3.fasta

./group205/reference/d1bu6o1-d1bu6o2.fasta

./group205/reference/d1bu6o1-d1bupa1.fasta

./group205/reference/d1bu6o1-d1bupa2.fasta

./group205/reference/d1bu6o1-d1czan1.fasta

./group205/reference/d1bu6o1-d1czan3.fasta

./group205/reference/d1bu6o1-d1e4ft1.fasta

./group205/reference/d1bu6o1-d1g99a1.fasta

./group205/reference/d1bu6o1-d1g99a2.fasta

./group205/reference/d1bu6o1-d1j6za2.fasta

./group205/reference/d1bu6o1-d1jcfa1.fasta

./group205/reference/d1bu6o1-d1mwma2.fasta

./group205/reference/d1bu6o1-d1nbwa3.fasta

./group205/reference/d1bu6o2-d1bupa1.fasta

./group205/reference/d1bu6o2-d1bupa2.fasta

./group205/reference/d1bu6o2-d1czan2.fasta

./group205/reference/d1bu6o2-d1czan3.fasta

./group205/reference/d1bu6o2-d1g99a1.fasta

./group205/reference/d1bu6o2-d1g99a2.fasta

./group205/reference/d1bu6o2-d1huxa_.fasta

./group205/reference/d1bu6o2-d1ig8a2.fasta

./group205/reference/d1bu6o2-d1j6za2.fasta

./group205/reference/d1bu6o2-d1nbwa3.fasta

./group205/reference/d1bupa1-d1bupa2.fasta

./group205/reference/d1bupa1-d1czan1.fasta

./group205/reference/d1bupa1-d1czan3.fasta

./group205/reference/d1bupa1-d1e4ft1.fasta

./group205/reference/d1bupa1-d1g99a2.fasta

./group205/reference/d1bupa1-d1huxa_.fasta

./group205/reference/d1bupa1-d1ig8a1.fasta

./group205/reference/d1bupa1-d1j6za2.fasta

./group205/reference/d1bupa1-d1mwma2.fasta

./group205/reference/d1bupa1-d1nbwa3.fasta

./group205/reference/d1bupa2-d1czan1.fasta

./group205/reference/d1bupa2-d1czan3.fasta

./group205/reference/d1bupa2-d1e4ft1.fasta

./group205/reference/d1bupa2-d1g99a1.fasta

./group205/reference/d1bupa2-d1huxa_.fasta

./group205/reference/d1bupa2-d1ig8a1.fasta

./group205/reference/d1bupa2-d1j6za2.fasta

./group205/reference/d1bupa2-d1jcfa1.fasta

./group205/reference/d1bupa2-d1mwma2.fasta

./group205/reference/d1czan1-d1e4ft1.fasta

./group205/reference/d1czan1-d1g99a1.fasta

./group205/reference/d1czan1-d1huxa_.fasta

./group205/reference/d1czan1-d1ig8a2.fasta

./group205/reference/d1czan1-d1j6za2.fasta

./group205/reference/d1czan1-d1mwma2.fasta

./group205/reference/d1czan1-d1nbwa3.fasta

./group205/reference/d1czan2-d1e4ft1.fasta

./group205/reference/d1czan2-d1g99a2.fasta

./group205/reference/d1czan2-d1huxa_.fasta

./group205/reference/d1czan2-d1ig8a1.fasta

./group205/reference/d1czan2-d1j6za2.fasta

./group205/reference/d1czan2-d1jcfa1.fasta

./group205/reference/d1czan2-d1mwma2.fasta

./group205/reference/d1czan2-d1nbwa3.fasta

./group205/reference/d1czan3-d1e4ft1.fasta

./group205/reference/d1czan3-d1g99a2.fasta

./group205/reference/d1czan3-d1huxa_.fasta

./group205/reference/d1czan3-d1ig8a2.fasta

./group205/reference/d1czan3-d1j6za2.fasta

./group205/reference/d1czan3-d1jcfa1.fasta

./group205/reference/d1czan3-d1mwma2.fasta

./group205/reference/d1czan3-d1nbwa3.fasta

./group205/reference/d1e4ft1-d1g99a1.fasta

./group205/reference/d1e4ft1-d1ig8a1.fasta

./group205/reference/d1e4ft1-d1ig8a2.fasta

./group205/reference/d1e4ft1-d1j6za2.fasta

./group205/reference/d1e4ft1-d1jcfa1.fasta

./group205/reference/d1e4ft1-d1mwma2.fasta

./group205/reference/d1e4ft1-d1nbwa3.fasta

./group205/reference/d1g99a1-d1huxa_.fasta

./group205/reference/d1g99a1-d1ig8a1.fasta

./group205/reference/d1g99a1-d1jcfa1.fasta

./group205/reference/d1g99a2-d1huxa_.fasta

./group205/reference/d1g99a2-d1ig8a1.fasta

./group205/reference/d1g99a2-d1jcfa1.fasta

./group205/reference/d1g99a2-d1mwma2.fasta

./group205/reference/d1g99a2-d1nbwa3.fasta

./group205/reference/d1huxa_-d1ig8a1.fasta

./group205/reference/d1huxa_-d1ig8a2.fasta

./group205/reference/d1huxa_-d1j6za2.fasta

./group205/reference/d1huxa_-d1jcfa1.fasta

./group205/reference/d1huxa_-d1mwma2.fasta

./group205/reference/d1huxa_-d1nbwa3.fasta

./group205/reference/d1ig8a1-d1j6za2.fasta

./group205/reference/d1ig8a1-d1jcfa1.fasta

./group205/reference/d1ig8a1-d1mwma2.fasta

./group205/reference/d1ig8a2-d1j6za2.fasta

./group205/reference/d1ig8a2-d1jcfa1.fasta

./group205/reference/d1j6za2-d1jcfa1.fasta

./group205/reference/d1j6za2-d1mwma2.fasta

./group205/reference/d1j6za2-d1nbwa3.fasta

./group205/reference/d1jcfa1-d1mwma2.fasta

./group205/reference/d1jcfa1-d1nbwa3.fasta

./group205/reference/d1mwma2-d1nbwa3.fasta

./group206/reference/d1hjra_-d1i39a_.fasta

./group206/reference/d1hjra_-d1io2a_.fasta

./group206/reference/d1hjra_-d1j54a_.fasta

./group206/reference/d1hjra_-d1l3sa1.fasta

./group206/reference/d1hjra_-d1tgoa1.fasta

./group206/reference/d1i39a_-d1ih7a1.fasta

./group206/reference/d1i39a_-d1j54a_.fasta

./group206/reference/d1i39a_-d1jl1a_.fasta

./group206/reference/d1i39a_-d1qtma1.fasta

./group206/reference/d1i39a_-d1tgoa1.fasta

./group206/reference/d1ih7a1-d1io2a_.fasta

./group206/reference/d1ih7a1-d1j54a_.fasta

./group206/reference/d1ih7a1-d1jl1a_.fasta

./group206/reference/d1ih7a1-d1kfsa1.fasta

./group206/reference/d1ih7a1-d1t7pa1.fasta

./group206/reference/d1io2a_-d1j54a_.fasta

./group206/reference/d1io2a_-d1jl1a_.fasta

./group206/reference/d1io2a_-d1l3sa1.fasta

./group206/reference/d1io2a_-d1t7pa1.fasta

./group206/reference/d1io2a_-d1tgoa1.fasta

./group206/reference/d1j54a_-d1jl1a_.fasta

./group206/reference/d1j54a_-d1kfsa1.fasta

./group206/reference/d1j54a_-d1l3sa1.fasta

./group206/reference/d1j54a_-d1qtma1.fasta

./group206/reference/d1j54a_-d1t7pa1.fasta

./group206/reference/d1j54a_-d1tgoa1.fasta

./group206/reference/d1jl1a_-d1qtma1.fasta

./group206/reference/d1l3sa1-d1qtma1.fasta

./group206/reference/d1l3sa1-d1t7pa1.fasta

./group206/reference/d1l3sa1-d1tgoa1.fasta

./group206/reference/d1qtma1-d1t7pa1.fasta

./group206/reference/d1qtma1-d1tgoa1.fasta

./group206/reference/d1t7pa1-d1tgoa1.fasta

./group207/reference/d1dt9a1-d1jj2m_.fasta

./group207/reference/d1fjgk_-d1jj2m_.fasta

./group208/reference/d1b8oa_-d1k9sa_.fasta

./group208/reference/d1g2oa_-d1k9sa_.fasta

./group209/reference/d1gyta2-d1loka_.fasta

./group209/reference/d1h8la2-d1lam_2.fasta

./group209/reference/d1h8la2-d1loka_.fasta

./group209/reference/d1lam_2-d1loka_.fasta

./group209/reference/d1loka_-d1m4la_.fasta

./group210/reference/d1a4ia2-d1bgva2.fasta

./group210/reference/d1a4ia2-d1lu9a2.fasta

./group210/reference/d1a4ia2-d1npya2.fasta

./group210/reference/d1a4ia2-d1nvta2.fasta

./group210/reference/d1b0aa2-d1leha2.fasta

./group210/reference/d1b0aa2-d1lu9a2.fasta

./group210/reference/d1b0aa2-d1npya2.fasta

./group210/reference/d1b0aa2-d1nvta2.fasta

./group210/reference/d1b0aa2-d1o0sa2.fasta

./group210/reference/d1bgva2-d1c1da2.fasta

./group210/reference/d1bgva2-d1leha2.fasta

./group210/reference/d1edza2-d1gtma2.fasta

./group210/reference/d1edza2-d1leha2.fasta

./group210/reference/d1edza2-d1lu9a2.fasta

./group210/reference/d1edza2-d1npya2.fasta

./group210/reference/d1edza2-d1nvta2.fasta

./group210/reference/d1gtma2-d1nvta2.fasta

./group210/reference/d1hwxa2-d1npya2.fasta

./group210/reference/d1hwxa2-d1nvta2.fasta

./group210/reference/d1lu9a2-d1nvta2.fasta

./group210/reference/d1nyta2-d1o0sa2.fasta

./group211/reference/d1bif_2-d1nd6a_.fasta

./group211/reference/d1e58a_-d1nd6a_.fasta

./group211/reference/d1fzta_-d1nd6a_.fasta

./group211/reference/d1h2ea_-d1nd6a_.fasta

./group212/reference/d1bd3a_-d1bzya_.fasta

./group212/reference/d1bd3a_-d1ecfa1.fasta

./group212/reference/d1bd3a_-d1g2qa_.fasta

./group212/reference/d1bd3a_-d1lh0a_.fasta

./group212/reference/d1bd3a_-d1qb7a_.fasta

./group212/reference/d1bzya_-d1g2qa_.fasta

./group212/reference/d1bzya_-d1gph11.fasta

./group212/reference/d1bzya_-d1i5ea_.fasta

./group212/reference/d1bzya_-d1l1qa_.fasta

./group212/reference/d1bzya_-d1lh0a_.fasta

./group212/reference/d1bzya_-d1qb7a_.fasta

./group212/reference/d1dqna_-d1ecfa1.fasta

./group212/reference/d1dqna_-d1g2qa_.fasta

./group212/reference/d1dqna_-d1gph11.fasta

./group212/reference/d1dqna_-d1l1qa_.fasta

./group212/reference/d1dqna_-d1lh0a_.fasta

./group212/reference/d1dqna_-d1qb7a_.fasta

./group212/reference/d1ecfa1-d1g2qa_.fasta

./group212/reference/d1ecfa1-d1i5ea_.fasta

./group212/reference/d1ecfa1-d1qb7a_.fasta

./group212/reference/d1fsga_-d1g2qa_.fasta

./group212/reference/d1fsga_-d1gph11.fasta

./group212/reference/d1fsga_-d1l1qa_.fasta

./group212/reference/d1fsga_-d1lh0a_.fasta

./group212/reference/d1fsga_-d1qb7a_.fasta

./group212/reference/d1g2qa_-d1gph11.fasta

./group212/reference/d1g2qa_-d1i5ea_.fasta

./group212/reference/d1g2qa_-d1lh0a_.fasta

./group212/reference/d1gph11-d1i5ea_.fasta

./group212/reference/d1gph11-d1l1qa_.fasta

./group212/reference/d1gph11-d1qb7a_.fasta

./group212/reference/d1i5ea_-d1l1qa_.fasta

./group212/reference/d1i5ea_-d1qb7a_.fasta

./group212/reference/d1l1qa_-d1lh0a_.fasta

./group213/reference/d1atza_-d1m1xb2.fasta

./group213/reference/d1ijba_-d1m1xb2.fasta

./group213/reference/d1ijba_-d1mf7a_.fasta

./group213/reference/d1ijba_-d1qc5a_.fasta

./group213/reference/d1m1xb2-d1mf7a_.fasta

./group213/reference/d1m1xb2-d1mjna_.fasta

./group213/reference/d1m1xb2-d1qc5a_.fasta

./group214/reference/d1af7_2-d1dcta_.fasta

./group214/reference/d1af7_2-d1dl5a1.fasta

./group214/reference/d1af7_2-d1f3la_.fasta

./group214/reference/d1af7_2-d1fp2a2.fasta

./group214/reference/d1af7_2-d1g6q1_.fasta

./group214/reference/d1af7_2-d1hnna_.fasta

./group214/reference/d1af7_2-d1i1na_.fasta

./group214/reference/d1af7_2-d1i9ga_.fasta

./group214/reference/d1af7_2-d1jg1a_.fasta

./group214/reference/d1af7_2-d1jqea_.fasta

./group214/reference/d1af7_2-d1kywa2.fasta

./group214/reference/d1af7_2-d1l9ka_.fasta

./group214/reference/d1af7_2-d1nt2a_.fasta

./group214/reference/d1af7_2-d1nv8a_.fasta

./group214/reference/d1af7_2-d1nw3a_.fasta

./group214/reference/d1af7_2-d1oria_.fasta

./group214/reference/d1af7_2-d1p1ca_.fasta

./group214/reference/d1af7_2-d1qama_.fasta

./group214/reference/d1af7_2-d1yub__.fasta

./group214/reference/d1af7_2-d6mhta_.fasta

./group214/reference/d1dcta_-d1dl5a1.fasta

./group214/reference/d1dcta_-d1f3la_.fasta

./group214/reference/d1dcta_-d1g38a_.fasta

./group214/reference/d1dcta_-d1g6q1_.fasta

./group214/reference/d1dcta_-d1hnna_.fasta

./group214/reference/d1dcta_-d1i1na_.fasta

./group214/reference/d1dcta_-d1i4wa_.fasta

./group214/reference/d1dcta_-d1i9ga_.fasta

./group214/reference/d1dcta_-d1iy9a_.fasta

./group214/reference/d1dcta_-d1jg1a_.fasta

./group214/reference/d1dcta_-d1jqea_.fasta

./group214/reference/d1dcta_-d1kywa2.fasta

./group214/reference/d1dcta_-d1l9ka_.fasta

./group214/reference/d1dcta_-d1nt2a_.fasta

./group214/reference/d1dcta_-d1nv8a_.fasta

./group214/reference/d1dcta_-d1nw3a_.fasta

./group214/reference/d1dcta_-d1oria_.fasta

./group214/reference/d1dcta_-d1yub__.fasta

./group214/reference/d1dl5a1-d1f3la_.fasta

./group214/reference/d1dl5a1-d1fp2a2.fasta

./group214/reference/d1dl5a1-d1g38a_.fasta

./group214/reference/d1dl5a1-d1hnna_.fasta

./group214/reference/d1dl5a1-d1i4wa_.fasta

./group214/reference/d1dl5a1-d1jqea_.fasta

./group214/reference/d1dl5a1-d1kywa2.fasta

./group214/reference/d1dl5a1-d1l9ka_.fasta

./group214/reference/d1dl5a1-d1nw3a_.fasta

./group214/reference/d1dl5a1-d1oria_.fasta

./group214/reference/d1dl5a1-d1p1ca_.fasta

./group214/reference/d1dl5a1-d1qama_.fasta

./group214/reference/d1dl5a1-d1xvaa_.fasta

./group214/reference/d1dl5a1-d1yub__.fasta

./group214/reference/d1dl5a1-d6mhta_.fasta

./group214/reference/d1f3la_-d1fp2a2.fasta

./group214/reference/d1f3la_-d1g38a_.fasta

./group214/reference/d1f3la_-d1hnna_.fasta

./group214/reference/d1f3la_-d1i4wa_.fasta

./group214/reference/d1f3la_-d1jg1a_.fasta

./group214/reference/d1f3la_-d1jqea_.fasta

./group214/reference/d1f3la_-d1kywa2.fasta

./group214/reference/d1f3la_-d1l9ka_.fasta

./group214/reference/d1f3la_-d1nt2a_.fasta

./group214/reference/d1f3la_-d1nw3a_.fasta

./group214/reference/d1f3la_-d1p1ca_.fasta

./group214/reference/d1f3la_-d1qama_.fasta

./group214/reference/d1f3la_-d1yub__.fasta

./group214/reference/d1f3la_-d6mhta_.fasta

./group214/reference/d1fp2a2-d1g38a_.fasta

./group214/reference/d1fp2a2-d1g6q1_.fasta

./group214/reference/d1fp2a2-d1hnna_.fasta

./group214/reference/d1fp2a2-d1i1na_.fasta

./group214/reference/d1fp2a2-d1i4wa_.fasta

./group214/reference/d1fp2a2-d1i9ga_.fasta

./group214/reference/d1fp2a2-d1iy9a_.fasta

./group214/reference/d1fp2a2-d1jqea_.fasta

./group214/reference/d1fp2a2-d1l9ka_.fasta

./group214/reference/d1fp2a2-d1nt2a_.fasta

./group214/reference/d1fp2a2-d1nv8a_.fasta

./group214/reference/d1fp2a2-d1nw3a_.fasta

./group214/reference/d1fp2a2-d1oria_.fasta

./group214/reference/d1fp2a2-d1p1ca_.fasta

./group214/reference/d1fp2a2-d1qama_.fasta

./group214/reference/d1fp2a2-d1xvaa_.fasta

./group214/reference/d1fp2a2-d1yub__.fasta

./group214/reference/d1fp2a2-d6mhta_.fasta

./group214/reference/d1g38a_-d1g6q1_.fasta

./group214/reference/d1g38a_-d1hnna_.fasta

./group214/reference/d1g38a_-d1i1na_.fasta

./group214/reference/d1g38a_-d1i4wa_.fasta

./group214/reference/d1g38a_-d1iy9a_.fasta

./group214/reference/d1g38a_-d1jqea_.fasta

./group214/reference/d1g38a_-d1kywa2.fasta

./group214/reference/d1g38a_-d1l9ka_.fasta

./group214/reference/d1g38a_-d1nt2a_.fasta

./group214/reference/d1g38a_-d1nv8a_.fasta

./group214/reference/d1g38a_-d1nw3a_.fasta

./group214/reference/d1g38a_-d1oria_.fasta

./group214/reference/d1g38a_-d1p1ca_.fasta

./group214/reference/d1g38a_-d1qama_.fasta

./group214/reference/d1g38a_-d6mhta_.fasta

./group214/reference/d1g6q1_-d1hnna_.fasta

./group214/reference/d1g6q1_-d1i4wa_.fasta

./group214/reference/d1g6q1_-d1iy9a_.fasta

./group214/reference/d1g6q1_-d1jqea_.fasta

./group214/reference/d1g6q1_-d1kywa2.fasta

./group214/reference/d1g6q1_-d1l9ka_.fasta

./group214/reference/d1g6q1_-d1p1ca_.fasta

./group214/reference/d1g6q1_-d1qama_.fasta

./group214/reference/d1g6q1_-d1yub__.fasta

./group214/reference/d1g6q1_-d6mhta_.fasta

./group214/reference/d1hnna_-d1i1na_.fasta

./group214/reference/d1hnna_-d1i4wa_.fasta

./group214/reference/d1hnna_-d1i9ga_.fasta

./group214/reference/d1hnna_-d1jg1a_.fasta

./group214/reference/d1hnna_-d1jqea_.fasta

./group214/reference/d1hnna_-d1kywa2.fasta

./group214/reference/d1hnna_-d1l9ka_.fasta

./group214/reference/d1hnna_-d1nt2a_.fasta

./group214/reference/d1hnna_-d1nv8a_.fasta

./group214/reference/d1hnna_-d1nw3a_.fasta

./group214/reference/d1hnna_-d1oria_.fasta

./group214/reference/d1hnna_-d1p1ca_.fasta

./group214/reference/d1hnna_-d1qama_.fasta

./group214/reference/d1hnna_-d1xvaa_.fasta

./group214/reference/d1hnna_-d1yub__.fasta

./group214/reference/d1hnna_-d6mhta_.fasta

./group214/reference/d1i1na_-d1i4wa_.fasta

./group214/reference/d1i1na_-d1jqea_.fasta

./group214/reference/d1i1na_-d1l9ka_.fasta

./group214/reference/d1i1na_-d1nt2a_.fasta

./group214/reference/d1i1na_-d1nv8a_.fasta

./group214/reference/d1i1na_-d1nw3a_.fasta

./group214/reference/d1i1na_-d1qama_.fasta

./group214/reference/d1i1na_-d1xvaa_.fasta

./group214/reference/d1i1na_-d6mhta_.fasta

./group214/reference/d1i4wa_-d1i9ga_.fasta

./group214/reference/d1i4wa_-d1jg1a_.fasta

./group214/reference/d1i4wa_-d1jqea_.fasta

./group214/reference/d1i4wa_-d1kywa2.fasta

./group214/reference/d1i4wa_-d1l9ka_.fasta

./group214/reference/d1i4wa_-d1nt2a_.fasta

./group214/reference/d1i4wa_-d1oria_.fasta

./group214/reference/d1i4wa_-d1p1ca_.fasta

./group214/reference/d1i4wa_-d1qama_.fasta

./group214/reference/d1i4wa_-d1yub__.fasta

./group214/reference/d1i9ga_-d1iy9a_.fasta

./group214/reference/d1i9ga_-d1jqea_.fasta

./group214/reference/d1i9ga_-d1kywa2.fasta

./group214/reference/d1i9ga_-d1l9ka_.fasta

./group214/reference/d1i9ga_-d1nt2a_.fasta

./group214/reference/d1i9ga_-d1nv8a_.fasta

./group214/reference/d1i9ga_-d1nw3a_.fasta

./group214/reference/d1i9ga_-d1p1ca_.fasta

./group214/reference/d1i9ga_-d1qama_.fasta

./group214/reference/d1i9ga_-d1xvaa_.fasta

./group214/reference/d1i9ga_-d1yub__.fasta

./group214/reference/d1i9ga_-d6mhta_.fasta

./group214/reference/d1iy9a_-d1jqea_.fasta

./group214/reference/d1iy9a_-d1kywa2.fasta

./group214/reference/d1iy9a_-d1l9ka_.fasta

./group214/reference/d1iy9a_-d1nt2a_.fasta

./group214/reference/d1iy9a_-d1nv8a_.fasta

./group214/reference/d1iy9a_-d1nw3a_.fasta

./group214/reference/d1iy9a_-d1oria_.fasta

./group214/reference/d1iy9a_-d1p1ca_.fasta

./group214/reference/d1iy9a_-d1qama_.fasta

./group214/reference/d1iy9a_-d1xvaa_.fasta

./group214/reference/d1iy9a_-d1yub__.fasta

./group214/reference/d1jg1a_-d1jqea_.fasta

./group214/reference/d1jg1a_-d1l9ka_.fasta

./group214/reference/d1jg1a_-d1nv8a_.fasta

./group214/reference/d1jg1a_-d1nw3a_.fasta

./group214/reference/d1jg1a_-d1oria_.fasta

./group214/reference/d1jg1a_-d1p1ca_.fasta

./group214/reference/d1jg1a_-d1qama_.fasta

./group214/reference/d1jg1a_-d1xvaa_.fasta

./group214/reference/d1jg1a_-d1yub__.fasta

./group214/reference/d1jg1a_-d6mhta_.fasta

./group214/reference/d1jqea_-d1kywa2.fasta

./group214/reference/d1jqea_-d1l9ka_.fasta

./group214/reference/d1jqea_-d1nt2a_.fasta

./group214/reference/d1jqea_-d1nv8a_.fasta

./group214/reference/d1jqea_-d1oria_.fasta

./group214/reference/d1jqea_-d1p1ca_.fasta

./group214/reference/d1jqea_-d1qama_.fasta

./group214/reference/d1jqea_-d6mhta_.fasta

./group214/reference/d1kywa2-d1l9ka_.fasta

./group214/reference/d1kywa2-d1nt2a_.fasta

./group214/reference/d1kywa2-d1nv8a_.fasta

./group214/reference/d1kywa2-d1nw3a_.fasta

./group214/reference/d1kywa2-d1oria_.fasta

./group214/reference/d1kywa2-d1p1ca_.fasta

./group214/reference/d1kywa2-d1xvaa_.fasta

./group214/reference/d1kywa2-d6mhta_.fasta

./group214/reference/d1l9ka_-d1nt2a_.fasta

./group214/reference/d1l9ka_-d1nw3a_.fasta

./group214/reference/d1l9ka_-d1oria_.fasta

./group214/reference/d1l9ka_-d1p1ca_.fasta

./group214/reference/d1l9ka_-d1xvaa_.fasta

./group214/reference/d1l9ka_-d6mhta_.fasta

./group214/reference/d1nt2a_-d1nv8a_.fasta

./group214/reference/d1nt2a_-d1nw3a_.fasta

./group214/reference/d1nt2a_-d1oria_.fasta

./group214/reference/d1nt2a_-d1p1ca_.fasta

./group214/reference/d1nt2a_-d1qama_.fasta

./group214/reference/d1nt2a_-d1xvaa_.fasta

./group214/reference/d1nt2a_-d1yub__.fasta

./group214/reference/d1nt2a_-d6mhta_.fasta

./group214/reference/d1nv8a_-d1nw3a_.fasta

./group214/reference/d1nv8a_-d1oria_.fasta

./group214/reference/d1nv8a_-d1p1ca_.fasta

./group214/reference/d1nv8a_-d1qama_.fasta

./group214/reference/d1nv8a_-d1xvaa_.fasta

./group214/reference/d1nv8a_-d1yub__.fasta

./group214/reference/d1nv8a_-d6mhta_.fasta

./group214/reference/d1nw3a_-d1oria_.fasta

./group214/reference/d1nw3a_-d1p1ca_.fasta

./group214/reference/d1nw3a_-d1qama_.fasta

./group214/reference/d1nw3a_-d1xvaa_.fasta

./group214/reference/d1nw3a_-d1yub__.fasta

./group214/reference/d1nw3a_-d6mhta_.fasta

./group214/reference/d1oria_-d1qama_.fasta

./group214/reference/d1oria_-d1xvaa_.fasta

./group214/reference/d1oria_-d1yub__.fasta

./group214/reference/d1oria_-d6mhta_.fasta

./group214/reference/d1p1ca_-d1xvaa_.fasta

./group214/reference/d1p1ca_-d1yub__.fasta

./group214/reference/d1p1ca_-d6mhta_.fasta

./group214/reference/d1qama_-d1xvaa_.fasta

./group214/reference/d1qama_-d6mhta_.fasta

./group214/reference/d1xvaa_-d1yub__.fasta

./group215/reference/d1ajsa_-d1b9ha_.fasta

./group215/reference/d1ajsa_-d1bj4a_.fasta

./group215/reference/d1ajsa_-d1bs0a_.fasta

./group215/reference/d1ajsa_-d1c4ka2.fasta

./group215/reference/d1ajsa_-d1c7na_.fasta

./group215/reference/d1ajsa_-d1e5ea_.fasta

./group215/reference/d1ajsa_-d1elua_.fasta

./group215/reference/d1ajsa_-d1gdea_.fasta

./group215/reference/d1ajsa_-d1j32a_.fasta

./group215/reference/d1ajsa_-d1jf9a_.fasta

./group215/reference/d1ajsa_-d1kl1a_.fasta

./group215/reference/d1ajsa_-d1n8pa_.fasta

./group215/reference/d1ajsa_-d1o4sa_.fasta

./group215/reference/d1ajsa_-d1tpla_.fasta

./group215/reference/d1ajsa_-d2dkb__.fasta

./group215/reference/d1ajsa_-d2gsaa_.fasta

./group215/reference/d1ajsa_-d2oata_.fasta

./group215/reference/d1b9ha_-d1bj4a_.fasta

./group215/reference/d1b9ha_-d1bs0a_.fasta

./group215/reference/d1b9ha_-d1e5ea_.fasta

./group215/reference/d1b9ha_-d1elua_.fasta

./group215/reference/d1b9ha_-d1gdea_.fasta

./group215/reference/d1b9ha_-d1gtxa_.fasta

./group215/reference/d1b9ha_-d1ibja_.fasta

./group215/reference/d1b9ha_-d1j32a_.fasta

./group215/reference/d1b9ha_-d1jf9a_.fasta

./group215/reference/d1b9ha_-d1kl1a_.fasta

./group215/reference/d1b9ha_-d1n8pa_.fasta

./group215/reference/d1b9ha_-d1o4sa_.fasta

./group215/reference/d1b9ha_-d1qisa_.fasta

./group215/reference/d1b9ha_-d1tpla_.fasta

./group215/reference/d1b9ha_-d1yaaa_.fasta

./group215/reference/d1b9ha_-d2ay1a_.fasta

./group215/reference/d1b9ha_-d2dkb__.fasta

./group215/reference/d1b9ha_-d2gsaa_.fasta

./group215/reference/d1b9ha_-d2oata_.fasta

./group215/reference/d1b9ha_-d3tata_.fasta

./group215/reference/d1b9ha_-d7aata_.fasta

./group215/reference/d1bj4a_-d1bs0a_.fasta

./group215/reference/d1bj4a_-d1c4ka2.fasta

./group215/reference/d1bj4a_-d1c7na_.fasta

./group215/reference/d1bj4a_-d1e5ea_.fasta

./group215/reference/d1bj4a_-d1elua_.fasta

./group215/reference/d1bj4a_-d1gdea_.fasta

./group215/reference/d1bj4a_-d1gtxa_.fasta

./group215/reference/d1bj4a_-d1ibja_.fasta

./group215/reference/d1bj4a_-d1j32a_.fasta

./group215/reference/d1bj4a_-d1jf9a_.fasta

./group215/reference/d1bj4a_-d1n8pa_.fasta

./group215/reference/d1bj4a_-d1o4sa_.fasta

./group215/reference/d1bj4a_-d1qisa_.fasta

./group215/reference/d1bj4a_-d1tpla_.fasta

./group215/reference/d1bj4a_-d1yaaa_.fasta

./group215/reference/d1bj4a_-d2ay1a_.fasta

./group215/reference/d1bj4a_-d2dkb__.fasta

./group215/reference/d1bj4a_-d2gsaa_.fasta

./group215/reference/d1bj4a_-d2oata_.fasta

./group215/reference/d1bj4a_-d3tata_.fasta

./group215/reference/d1bj4a_-d7aata_.fasta

./group215/reference/d1bs0a_-d1c4ka2.fasta

./group215/reference/d1bs0a_-d1c7na_.fasta

./group215/reference/d1bs0a_-d1e5ea_.fasta

./group215/reference/d1bs0a_-d1elua_.fasta

./group215/reference/d1bs0a_-d1gdea_.fasta

./group215/reference/d1bs0a_-d1gtxa_.fasta

./group215/reference/d1bs0a_-d1ibja_.fasta

./group215/reference/d1bs0a_-d1j32a_.fasta

./group215/reference/d1bs0a_-d1jf9a_.fasta

./group215/reference/d1bs0a_-d1kl1a_.fasta

./group215/reference/d1bs0a_-d1n8pa_.fasta

./group215/reference/d1bs0a_-d1o4sa_.fasta

./group215/reference/d1bs0a_-d1qisa_.fasta

./group215/reference/d1bs0a_-d1tpla_.fasta

./group215/reference/d1bs0a_-d2ay1a_.fasta

./group215/reference/d1bs0a_-d2dkb__.fasta

./group215/reference/d1bs0a_-d2gsaa_.fasta

./group215/reference/d1bs0a_-d2oata_.fasta

./group215/reference/d1bs0a_-d3tata_.fasta

./group215/reference/d1bs0a_-d7aata_.fasta

./group215/reference/d1c4ka2-d1e5ea_.fasta

./group215/reference/d1c4ka2-d1elua_.fasta

./group215/reference/d1c4ka2-d1gtxa_.fasta

./group215/reference/d1c4ka2-d1ibja_.fasta

./group215/reference/d1c4ka2-d1jf9a_.fasta

./group215/reference/d1c4ka2-d1kl1a_.fasta

./group215/reference/d1c4ka2-d1n8pa_.fasta

./group215/reference/d1c4ka2-d1o4sa_.fasta

./group215/reference/d1c4ka2-d1qisa_.fasta

./group215/reference/d1c4ka2-d1tpla_.fasta

./group215/reference/d1c4ka2-d1yaaa_.fasta

./group215/reference/d1c4ka2-d2gsaa_.fasta

./group215/reference/d1c4ka2-d2oata_.fasta

./group215/reference/d1c4ka2-d3tata_.fasta

./group215/reference/d1c7na_-d1e5ea_.fasta

./group215/reference/d1c7na_-d1elua_.fasta

./group215/reference/d1c7na_-d1gtxa_.fasta

./group215/reference/d1c7na_-d1ibja_.fasta

./group215/reference/d1c7na_-d1j32a_.fasta

./group215/reference/d1c7na_-d1jf9a_.fasta

./group215/reference/d1c7na_-d1kl1a_.fasta

./group215/reference/d1c7na_-d1n8pa_.fasta

./group215/reference/d1c7na_-d1qisa_.fasta

./group215/reference/d1c7na_-d1tpla_.fasta

./group215/reference/d1c7na_-d1yaaa_.fasta

./group215/reference/d1c7na_-d2ay1a_.fasta

./group215/reference/d1c7na_-d2dkb__.fasta

./group215/reference/d1c7na_-d2gsaa_.fasta

./group215/reference/d1c7na_-d2oata_.fasta

./group215/reference/d1c7na_-d3tata_.fasta

./group215/reference/d1e5ea_-d1elua_.fasta

./group215/reference/d1e5ea_-d1gdea_.fasta

./group215/reference/d1e5ea_-d1gtxa_.fasta

./group215/reference/d1e5ea_-d1j32a_.fasta

./group215/reference/d1e5ea_-d1jf9a_.fasta

./group215/reference/d1e5ea_-d1kl1a_.fasta

./group215/reference/d1e5ea_-d1o4sa_.fasta

./group215/reference/d1e5ea_-d1qisa_.fasta

./group215/reference/d1e5ea_-d1tpla_.fasta

./group215/reference/d1e5ea_-d1yaaa_.fasta

./group215/reference/d1e5ea_-d2ay1a_.fasta

./group215/reference/d1e5ea_-d2dkb__.fasta

./group215/reference/d1e5ea_-d2gsaa_.fasta

./group215/reference/d1e5ea_-d2oata_.fasta

./group215/reference/d1e5ea_-d3tata_.fasta

./group215/reference/d1e5ea_-d7aata_.fasta

./group215/reference/d1elua_-d1gdea_.fasta

./group215/reference/d1elua_-d1gtxa_.fasta

./group215/reference/d1elua_-d1ibja_.fasta

./group215/reference/d1elua_-d1kl1a_.fasta

./group215/reference/d1elua_-d1n8pa_.fasta

./group215/reference/d1elua_-d1o4sa_.fasta

./group215/reference/d1elua_-d1qisa_.fasta

./group215/reference/d1elua_-d1yaaa_.fasta

./group215/reference/d1elua_-d2ay1a_.fasta

./group215/reference/d1elua_-d2dkb__.fasta

./group215/reference/d1elua_-d2gsaa_.fasta

./group215/reference/d1elua_-d2oata_.fasta

./group215/reference/d1elua_-d3tata_.fasta

./group215/reference/d1elua_-d7aata_.fasta

./group215/reference/d1gdea_-d1ibja_.fasta

./group215/reference/d1gdea_-d1jf9a_.fasta

./group215/reference/d1gdea_-d1kl1a_.fasta

./group215/reference/d1gdea_-d1n8pa_.fasta

./group215/reference/d1gdea_-d1qisa_.fasta

./group215/reference/d1gdea_-d1tpla_.fasta

./group215/reference/d1gdea_-d1yaaa_.fasta

./group215/reference/d1gdea_-d2ay1a_.fasta

./group215/reference/d1gdea_-d2dkb__.fasta

./group215/reference/d1gdea_-d2gsaa_.fasta

./group215/reference/d1gdea_-d2oata_.fasta

./group215/reference/d1gdea_-d7aata_.fasta

./group215/reference/d1gtxa_-d1ibja_.fasta

./group215/reference/d1gtxa_-d1j32a_.fasta

./group215/reference/d1gtxa_-d1jf9a_.fasta

./group215/reference/d1gtxa_-d1kl1a_.fasta

./group215/reference/d1gtxa_-d1n8pa_.fasta

./group215/reference/d1gtxa_-d1o4sa_.fasta

./group215/reference/d1gtxa_-d1tpla_.fasta

./group215/reference/d1gtxa_-d2ay1a_.fasta

./group215/reference/d1ibja_-d1j32a_.fasta

./group215/reference/d1ibja_-d1jf9a_.fasta

./group215/reference/d1ibja_-d1kl1a_.fasta

./group215/reference/d1ibja_-d1qisa_.fasta

./group215/reference/d1ibja_-d1tpla_.fasta

./group215/reference/d1ibja_-d1yaaa_.fasta

./group215/reference/d1ibja_-d2ay1a_.fasta

./group215/reference/d1ibja_-d2dkb__.fasta

./group215/reference/d1ibja_-d2gsaa_.fasta

./group215/reference/d1ibja_-d2oata_.fasta

./group215/reference/d1ibja_-d3tata_.fasta

./group215/reference/d1ibja_-d7aata_.fasta

./group215/reference/d1j32a_-d1jf9a_.fasta

./group215/reference/d1j32a_-d1kl1a_.fasta

./group215/reference/d1j32a_-d1n8pa_.fasta

./group215/reference/d1j32a_-d1qisa_.fasta

./group215/reference/d1j32a_-d1tpla_.fasta

./group215/reference/d1j32a_-d1yaaa_.fasta

./group215/reference/d1j32a_-d2ay1a_.fasta

./group215/reference/d1j32a_-d2dkb__.fasta

./group215/reference/d1j32a_-d2gsaa_.fasta

./group215/reference/d1j32a_-d2oata_.fasta

./group215/reference/d1j32a_-d3tata_.fasta

./group215/reference/d1j32a_-d7aata_.fasta

./group215/reference/d1jf9a_-d1kl1a_.fasta

./group215/reference/d1jf9a_-d1n8pa_.fasta

./group215/reference/d1jf9a_-d1o4sa_.fasta

./group215/reference/d1jf9a_-d1qisa_.fasta

./group215/reference/d1jf9a_-d1tpla_.fasta

./group215/reference/d1jf9a_-d1yaaa_.fasta

./group215/reference/d1jf9a_-d2ay1a_.fasta

./group215/reference/d1jf9a_-d2dkb__.fasta

./group215/reference/d1jf9a_-d2gsaa_.fasta

./group215/reference/d1jf9a_-d2oata_.fasta

./group215/reference/d1jf9a_-d3tata_.fasta

./group215/reference/d1jf9a_-d7aata_.fasta

./group215/reference/d1kl1a_-d1n8pa_.fasta

./group215/reference/d1kl1a_-d1o4sa_.fasta

./group215/reference/d1kl1a_-d1qisa_.fasta

./group215/reference/d1kl1a_-d1tpla_.fasta

./group215/reference/d1kl1a_-d1yaaa_.fasta

./group215/reference/d1kl1a_-d2ay1a_.fasta

./group215/reference/d1kl1a_-d2dkb__.fasta

./group215/reference/d1kl1a_-d2gsaa_.fasta

./group215/reference/d1kl1a_-d2oata_.fasta

./group215/reference/d1kl1a_-d7aata_.fasta

./group215/reference/d1n8pa_-d1o4sa_.fasta

./group215/reference/d1n8pa_-d1qisa_.fasta

./group215/reference/d1n8pa_-d1tpla_.fasta

./group215/reference/d1n8pa_-d1yaaa_.fasta

./group215/reference/d1n8pa_-d2ay1a_.fasta

./group215/reference/d1n8pa_-d2dkb__.fasta

./group215/reference/d1n8pa_-d2gsaa_.fasta

./group215/reference/d1n8pa_-d2oata_.fasta

./group215/reference/d1n8pa_-d3tata_.fasta

./group215/reference/d1n8pa_-d7aata_.fasta

./group215/reference/d1o4sa_-d1qisa_.fasta

./group215/reference/d1o4sa_-d1tpla_.fasta

./group215/reference/d1o4sa_-d1yaaa_.fasta

./group215/reference/d1o4sa_-d2ay1a_.fasta

./group215/reference/d1o4sa_-d2dkb__.fasta

./group215/reference/d1o4sa_-d2gsaa_.fasta

./group215/reference/d1o4sa_-d2oata_.fasta

./group215/reference/d1o4sa_-d3tata_.fasta

./group215/reference/d1o4sa_-d7aata_.fasta

./group215/reference/d1qisa_-d1tpla_.fasta

./group215/reference/d1qisa_-d2dkb__.fasta

./group215/reference/d1qisa_-d2gsaa_.fasta

./group215/reference/d1qisa_-d2oata_.fasta

./group215/reference/d1tpla_-d1yaaa_.fasta

./group215/reference/d1tpla_-d2ay1a_.fasta

./group215/reference/d1tpla_-d2dkb__.fasta

./group215/reference/d1tpla_-d2gsaa_.fasta

./group215/reference/d1tpla_-d2oata_.fasta

./group215/reference/d1tpla_-d3tata_.fasta

./group215/reference/d1tpla_-d7aata_.fasta

./group215/reference/d1yaaa_-d2dkb__.fasta

./group215/reference/d1yaaa_-d2gsaa_.fasta

./group215/reference/d1yaaa_-d2oata_.fasta

./group215/reference/d2ay1a_-d2dkb__.fasta

./group215/reference/d2ay1a_-d2gsaa_.fasta

./group215/reference/d2ay1a_-d2oata_.fasta

./group215/reference/d2dkb__-d3tata_.fasta

./group215/reference/d2dkb__-d7aata_.fasta

./group215/reference/d2gsaa_-d3tata_.fasta

./group215/reference/d2gsaa_-d7aata_.fasta

./group215/reference/d2oata_-d3tata_.fasta

./group216/reference/d1e5ka_-d1fxoa_.fasta

./group216/reference/d1e5ka_-d1hm9a2.fasta

./group216/reference/d1e5ka_-d1i52a_.fasta

./group216/reference/d1fxoa_-d1gx4a_.fasta

./group216/reference/d1fxoa_-d1h7ea_.fasta

./group216/reference/d1fxoa_-d1hm9a2.fasta

./group216/reference/d1fxoa_-d1hv9a2.fasta

./group216/reference/d1fxoa_-d1i52a_.fasta

./group216/reference/d1fxoa_-d1nf5b_.fasta

./group216/reference/d1gx4a_-d1i52a_.fasta

./group216/reference/d1h7ea_-d1hm9a2.fasta

./group216/reference/d1h7ea_-d1hv9a2.fasta

./group216/reference/d1h7ea_-d1i52a_.fasta

./group216/reference/d1h7ea_-d1nf5b_.fasta

./group216/reference/d1hm9a2-d1i52a_.fasta

./group216/reference/d1hv9a2-d1i52a_.fasta

./group216/reference/d1i52a_-d1nf5b_.fasta

./group217/reference/d1ac5__-d1bn7a_.fasta

./group217/reference/d1ac5__-d1c4xa_.fasta

./group217/reference/d1ac5__-d1dqza_.fasta

./group217/reference/d1ac5__-d1ek1a2.fasta

./group217/reference/d1ac5__-d1ex9a_.fasta

./group217/reference/d1ac5__-d1gkla_.fasta

./group217/reference/d1ac5__-d1iz7a_.fasta

./group217/reference/d1ac5__-d1jjia_.fasta

./group217/reference/d1ac5__-d1ju3a2.fasta

./group217/reference/d1ac5__-d1ku0a_.fasta

./group217/reference/d1ac5__-d1l7aa_.fasta

./group217/reference/d1ac5__-d1llfa_.fasta

./group217/reference/d1ac5__-d1mtza_.fasta

./group217/reference/d1ac5__-d1mx1a_.fasta

./group217/reference/d1ac5__-d1qlwa_.fasta

./group217/reference/d1ac5__-d1qtra_.fasta

./group217/reference/d1ac5__-d1tca__.fasta

./group217/reference/d1bn7a_-d1bu8a2.fasta

./group217/reference/d1bn7a_-d1cpy__.fasta

./group217/reference/d1bn7a_-d1dqza_.fasta

./group217/reference/d1bn7a_-d1ex9a_.fasta

./group217/reference/d1bn7a_-d1ivya_.fasta

./group217/reference/d1bn7a_-d1jjia_.fasta

./group217/reference/d1bn7a_-d1jkma_.fasta

./group217/reference/d1bn7a_-d1ju3a2.fasta

./group217/reference/d1bn7a_-d1ku0a_.fasta

./group217/reference/d1bn7a_-d1l7aa_.fasta

./group217/reference/d1bn7a_-d1lnsa3.fasta

./group217/reference/d1bn7a_-d1lzla_.fasta

./group217/reference/d1bn7a_-d1mtza_.fasta

./group217/reference/d1bn7a_-d1qlwa_.fasta

./group217/reference/d1bn7a_-d1tca__.fasta

./group217/reference/d1bu8a2-d1c4xa_.fasta

./group217/reference/d1bu8a2-d1cpy__.fasta

./group217/reference/d1bu8a2-d1dqza_.fasta

./group217/reference/d1bu8a2-d1ea5a_.fasta

./group217/reference/d1bu8a2-d1ek1a2.fasta

./group217/reference/d1bu8a2-d1ex9a_.fasta

./group217/reference/d1bu8a2-d1gkla_.fasta

./group217/reference/d1bu8a2-d1iz7a_.fasta

./group217/reference/d1bu8a2-d1jjia_.fasta

./group217/reference/d1bu8a2-d1jkma_.fasta

./group217/reference/d1bu8a2-d1ku0a_.fasta

./group217/reference/d1bu8a2-d1l7aa_.fasta

./group217/reference/d1bu8a2-d1llfa_.fasta

./group217/reference/d1bu8a2-d1lnsa3.fasta

./group217/reference/d1bu8a2-d1mtza_.fasta

./group217/reference/d1bu8a2-d1mx1a_.fasta

./group217/reference/d1bu8a2-d1qlwa_.fasta

./group217/reference/d1bu8a2-d1qtra_.fasta

./group217/reference/d1bu8a2-d1tca__.fasta

./group217/reference/d1c4xa_-d1cpy__.fasta

./group217/reference/d1c4xa_-d1dqza_.fasta

./group217/reference/d1c4xa_-d1ea5a_.fasta

./group217/reference/d1c4xa_-d1ex9a_.fasta

./group217/reference/d1c4xa_-d1gkla_.fasta

./group217/reference/d1c4xa_-d1ivya_.fasta

./group217/reference/d1c4xa_-d1iz7a_.fasta

./group217/reference/d1c4xa_-d1jjia_.fasta

./group217/reference/d1c4xa_-d1jkma_.fasta

./group217/reference/d1c4xa_-d1ju3a2.fasta

./group217/reference/d1c4xa_-d1l7aa_.fasta

./group217/reference/d1c4xa_-d1llfa_.fasta

./group217/reference/d1c4xa_-d1lnsa3.fasta

./group217/reference/d1c4xa_-d1lzla_.fasta

./group217/reference/d1c4xa_-d1mx1a_.fasta

./group217/reference/d1c4xa_-d1qlwa_.fasta

./group217/reference/d1c4xa_-d1tca__.fasta

./group217/reference/d1cpy__-d1dqza_.fasta

./group217/reference/d1cpy__-d1ea5a_.fasta

./group217/reference/d1cpy__-d1ek1a2.fasta

./group217/reference/d1cpy__-d1ex9a_.fasta

./group217/reference/d1cpy__-d1gkla_.fasta

./group217/reference/d1cpy__-d1iz7a_.fasta

./group217/reference/d1cpy__-d1jjia_.fasta

./group217/reference/d1cpy__-d1jkma_.fasta

./group217/reference/d1cpy__-d1ju3a2.fasta

./group217/reference/d1cpy__-d1ku0a_.fasta

./group217/reference/d1cpy__-d1l7aa_.fasta

./group217/reference/d1cpy__-d1llfa_.fasta

./group217/reference/d1cpy__-d1lnsa3.fasta

./group217/reference/d1cpy__-d1lzla_.fasta

./group217/reference/d1cpy__-d1mtza_.fasta

./group217/reference/d1cpy__-d1mx1a_.fasta

./group217/reference/d1cpy__-d1qlwa_.fasta

./group217/reference/d1cpy__-d1qtra_.fasta

./group217/reference/d1dqza_-d1ea5a_.fasta

./group217/reference/d1dqza_-d1ek1a2.fasta

./group217/reference/d1dqza_-d1ex9a_.fasta

./group217/reference/d1dqza_-d1gkla_.fasta

./group217/reference/d1dqza_-d1ivya_.fasta

./group217/reference/d1dqza_-d1iz7a_.fasta

./group217/reference/d1dqza_-d1jkma_.fasta

./group217/reference/d1dqza_-d1ju3a2.fasta

./group217/reference/d1dqza_-d1ku0a_.fasta

./group217/reference/d1dqza_-d1l7aa_.fasta

./group217/reference/d1dqza_-d1llfa_.fasta

./group217/reference/d1dqza_-d1lnsa3.fasta

./group217/reference/d1dqza_-d1lzla_.fasta

./group217/reference/d1dqza_-d1mtza_.fasta

./group217/reference/d1dqza_-d1mx1a_.fasta

./group217/reference/d1dqza_-d1qlwa_.fasta

./group217/reference/d1dqza_-d1qtra_.fasta

./group217/reference/d1dqza_-d1tca__.fasta

./group217/reference/d1ea5a_-d1ek1a2.fasta

./group217/reference/d1ea5a_-d1ex9a_.fasta

./group217/reference/d1ea5a_-d1gkla_.fasta

./group217/reference/d1ea5a_-d1ivya_.fasta

./group217/reference/d1ea5a_-d1jjia_.fasta

./group217/reference/d1ea5a_-d1ju3a2.fasta

./group217/reference/d1ea5a_-d1ku0a_.fasta

./group217/reference/d1ea5a_-d1l7aa_.fasta

./group217/reference/d1ea5a_-d1lzla_.fasta

./group217/reference/d1ea5a_-d1mtza_.fasta

./group217/reference/d1ea5a_-d1qtra_.fasta

./group217/reference/d1ek1a2-d1ex9a_.fasta

./group217/reference/d1ek1a2-d1gkla_.fasta

./group217/reference/d1ek1a2-d1ivya_.fasta

./group217/reference/d1ek1a2-d1jjia_.fasta

./group217/reference/d1ek1a2-d1jkma_.fasta

./group217/reference/d1ek1a2-d1ju3a2.fasta

./group217/reference/d1ek1a2-d1ku0a_.fasta

./group217/reference/d1ek1a2-d1l7aa_.fasta

./group217/reference/d1ek1a2-d1llfa_.fasta

./group217/reference/d1ek1a2-d1lnsa3.fasta

./group217/reference/d1ek1a2-d1lzla_.fasta

./group217/reference/d1ek1a2-d1mtza_.fasta

./group217/reference/d1ek1a2-d1mx1a_.fasta

./group217/reference/d1ek1a2-d1qlwa_.fasta

./group217/reference/d1ek1a2-d1qtra_.fasta

./group217/reference/d1ek1a2-d1tca__.fasta

./group217/reference/d1ex9a_-d1gkla_.fasta

./group217/reference/d1ex9a_-d1ivya_.fasta

./group217/reference/d1ex9a_-d1iz7a_.fasta

./group217/reference/d1ex9a_-d1jjia_.fasta

./group217/reference/d1ex9a_-d1jkma_.fasta

./group217/reference/d1ex9a_-d1ju3a2.fasta

./group217/reference/d1ex9a_-d1l7aa_.fasta

./group217/reference/d1ex9a_-d1llfa_.fasta

./group217/reference/d1ex9a_-d1lzla_.fasta

./group217/reference/d1ex9a_-d1mtza_.fasta

./group217/reference/d1ex9a_-d1mx1a_.fasta

./group217/reference/d1ex9a_-d1qtra_.fasta

./group217/reference/d1ex9a_-d1tca__.fasta

./group217/reference/d1gkla_-d1ivya_.fasta

./group217/reference/d1gkla_-d1jjia_.fasta

./group217/reference/d1gkla_-d1jkma_.fasta

./group217/reference/d1gkla_-d1ju3a2.fasta

./group217/reference/d1gkla_-d1ku0a_.fasta

./group217/reference/d1gkla_-d1l7aa_.fasta

./group217/reference/d1gkla_-d1llfa_.fasta

./group217/reference/d1gkla_-d1lnsa3.fasta

./group217/reference/d1gkla_-d1lzla_.fasta

./group217/reference/d1gkla_-d1mtza_.fasta

./group217/reference/d1gkla_-d1mx1a_.fasta

./group217/reference/d1gkla_-d1qlwa_.fasta

./group217/reference/d1gkla_-d1qtra_.fasta

./group217/reference/d1gkla_-d1tca__.fasta

./group217/reference/d1ivya_-d1iz7a_.fasta

./group217/reference/d1ivya_-d1jjia_.fasta

./group217/reference/d1ivya_-d1jkma_.fasta

./group217/reference/d1ivya_-d1ju3a2.fasta

./group217/reference/d1ivya_-d1ku0a_.fasta

./group217/reference/d1ivya_-d1l7aa_.fasta

./group217/reference/d1ivya_-d1llfa_.fasta

./group217/reference/d1ivya_-d1lnsa3.fasta

./group217/reference/d1ivya_-d1lzla_.fasta

./group217/reference/d1ivya_-d1mtza_.fasta

./group217/reference/d1ivya_-d1mx1a_.fasta

./group217/reference/d1ivya_-d1qlwa_.fasta

./group217/reference/d1ivya_-d1qtra_.fasta

./group217/reference/d1iz7a_-d1jjia_.fasta

./group217/reference/d1iz7a_-d1jkma_.fasta

./group217/reference/d1iz7a_-d1ju3a2.fasta

./group217/reference/d1iz7a_-d1ku0a_.fasta

./group217/reference/d1iz7a_-d1l7aa_.fasta

./group217/reference/d1iz7a_-d1mtza_.fasta

./group217/reference/d1iz7a_-d1qlwa_.fasta

./group217/reference/d1iz7a_-d1qtra_.fasta

./group217/reference/d1iz7a_-d1tca__.fasta

./group217/reference/d1jjia_-d1ju3a2.fasta

./group217/reference/d1jjia_-d1ku0a_.fasta

./group217/reference/d1jjia_-d1l7aa_.fasta

./group217/reference/d1jjia_-d1lnsa3.fasta

./group217/reference/d1jjia_-d1mtza_.fasta

./group217/reference/d1jjia_-d1mx1a_.fasta

./group217/reference/d1jjia_-d1qlwa_.fasta

./group217/reference/d1jjia_-d1qtra_.fasta

./group217/reference/d1jjia_-d1tca__.fasta

./group217/reference/d1jkma_-d1ju3a2.fasta

./group217/reference/d1jkma_-d1ku0a_.fasta

./group217/reference/d1jkma_-d1lnsa3.fasta

./group217/reference/d1jkma_-d1qlwa_.fasta

./group217/reference/d1jkma_-d1qtra_.fasta

./group217/reference/d1jkma_-d1tca__.fasta

./group217/reference/d1ju3a2-d1ku0a_.fasta

./group217/reference/d1ju3a2-d1llfa_.fasta

./group217/reference/d1ju3a2-d1lzla_.fasta

./group217/reference/d1ju3a2-d1mx1a_.fasta

./group217/reference/d1ju3a2-d1qlwa_.fasta

./group217/reference/d1ju3a2-d1qtra_.fasta

./group217/reference/d1ku0a_-d1l7aa_.fasta

./group217/reference/d1ku0a_-d1lnsa3.fasta

./group217/reference/d1ku0a_-d1lzla_.fasta

./group217/reference/d1ku0a_-d1mtza_.fasta

./group217/reference/d1ku0a_-d1mx1a_.fasta

./group217/reference/d1ku0a_-d1qlwa_.fasta

./group217/reference/d1ku0a_-d1qtra_.fasta

./group217/reference/d1ku0a_-d1tca__.fasta

./group217/reference/d1l7aa_-d1llfa_.fasta

./group217/reference/d1l7aa_-d1lnsa3.fasta

./group217/reference/d1l7aa_-d1mtza_.fasta

./group217/reference/d1l7aa_-d1mx1a_.fasta

./group217/reference/d1l7aa_-d1qlwa_.fasta

./group217/reference/d1l7aa_-d1qtra_.fasta

./group217/reference/d1l7aa_-d1tca__.fasta

./group217/reference/d1llfa_-d1lnsa3.fasta

./group217/reference/d1llfa_-d1lzla_.fasta

./group217/reference/d1llfa_-d1mtza_.fasta

./group217/reference/d1llfa_-d1qtra_.fasta

./group217/reference/d1lnsa3-d1lzla_.fasta

./group217/reference/d1lnsa3-d1mtza_.fasta

./group217/reference/d1lnsa3-d1qlwa_.fasta

./group217/reference/d1lnsa3-d1qtra_.fasta

./group217/reference/d1lzla_-d1mtza_.fasta

./group217/reference/d1lzla_-d1qlwa_.fasta

./group217/reference/d1lzla_-d1qtra_.fasta

./group217/reference/d1lzla_-d1tca__.fasta

./group217/reference/d1mtza_-d1mx1a_.fasta

./group217/reference/d1mtza_-d1qlwa_.fasta

./group217/reference/d1mtza_-d1tca__.fasta

./group217/reference/d1mx1a_-d1qtra_.fasta

./group217/reference/d1mx1a_-d1tca__.fasta

./group217/reference/d1qlwa_-d1qtra_.fasta

./group217/reference/d1qlwa_-d1tca__.fasta

./group217/reference/d1qtra_-d1tca__.fasta

./group219/reference/d1bx4a_-d1o14a_.fasta

./group219/reference/d1o14a_-d1rkd__.fasta

./group221/reference/d1e4bp_-d1gt7a_.fasta

./group221/reference/d1gt7a_-d1k0wa_.fasta

./group223/reference/d1cnza_-d1itwa_.fasta

./group223/reference/d1itwa_-d1lwda_.fasta

./group223/reference/d1itwa_-d1xaa__.fasta

./group224/reference/d1a1s_2-d1duvg1.fasta

./group224/reference/d1a1s_2-d1js1x1.fasta

./group224/reference/d1a1s_2-d1ml4a1.fasta

./group224/reference/d1duvg1-d1duvg2.fasta

./group224/reference/d1duvg1-d1ekxa2.fasta

./group224/reference/d1duvg1-d1ml4a2.fasta

./group224/reference/d1duvg1-d1otha2.fasta

./group224/reference/d1duvg2-d1ekxa2.fasta

./group224/reference/d1duvg2-d1js1x1.fasta

./group224/reference/d1duvg2-d1ml4a1.fasta

./group224/reference/d1duvg2-d1otha1.fasta

./group224/reference/d1ekxa2-d1js1x2.fasta

./group224/reference/d1ekxa2-d1ml4a1.fasta

./group224/reference/d1ekxa2-d1otha1.fasta

./group224/reference/d1ekxa2-d1otha2.fasta

./group224/reference/d1js1x1-d1ml4a2.fasta

./group224/reference/d1js1x1-d1otha2.fasta

./group224/reference/d1js1x2-d1ml4a2.fasta

./group224/reference/d1ml4a1-d1ml4a2.fasta

./group224/reference/d1ml4a1-d1otha2.fasta

./group224/reference/d1ml4a2-d1otha1.fasta

./group224/reference/d1otha1-d1otha2.fasta

./group225/reference/d1b74a1-d1b74a2.fasta

./group225/reference/d1b74a1-d1jfla2.fasta

./group225/reference/d1b74a2-d1jfla1.fasta

./group225/reference/d1b74a2-d1jfla2.fasta

./group226/reference/d1f2da_-d1j6na_.fasta

./group226/reference/d1f2da_-d1qopb_.fasta

./group226/reference/d1f2da_-d1tdj_1.fasta

./group226/reference/d1j0aa_-d1j6na_.fasta

./group226/reference/d1j0aa_-d1qopb_.fasta

./group226/reference/d1j0aa_-d1tdj_1.fasta

./group227/reference/d1iata_-d1moq__.fasta

./group229/reference/d1ad3a_-d1ez0a_.fasta

./group229/reference/d1ez0a_-d1ky8a_.fasta

./group230/reference/d1k2yx1-d1k2yx2.fasta

./group230/reference/d1k2yx1-d1kfia1.fasta

./group230/reference/d1k2yx2-d1k2yx3.fasta

./group230/reference/d1k2yx2-d1kfia1.fasta

./group230/reference/d1k2yx2-d3pmga1.fasta

./group230/reference/d1k2yx2-d3pmga3.fasta

./group230/reference/d1k2yx3-d3pmga1.fasta

./group230/reference/d1k2yx3-d3pmga2.fasta

./group230/reference/d1k2yx3-d3pmga3.fasta

./group232/reference/d1jixa_-d1l5wa_.fasta

./group234/reference/d1doza_-d1qgoa_.fasta

./group234/reference/d1lbqa_-d1qgoa_.fasta

./group235/reference/d1efdn_-d1m1na_.fasta

./group235/reference/d1efdn_-d1m1nb_.fasta

./group235/reference/d1efdn_-d1miob_.fasta

./group235/reference/d1efdn_-d1n2za_.fasta

./group235/reference/d1efdn_-d1psza_.fasta

./group235/reference/d1efdn_-d1toaa_.fasta

./group235/reference/d1m1na_-d1m1nb_.fasta

./group235/reference/d1m1na_-d1n2za_.fasta

./group235/reference/d1m1na_-d1toaa_.fasta

./group235/reference/d1m1nb_-d1psza_.fasta

./group235/reference/d1m1nb_-d1toaa_.fasta

./group235/reference/d1miob_-d1psza_.fasta

./group235/reference/d1miob_-d1toaa_.fasta

./group235/reference/d1n2za_-d1toaa_.fasta

./group236/reference/d1byka_-d1dp4a_.fasta

./group236/reference/d1byka_-d1gca__.fasta

./group236/reference/d1byka_-d1jx6a_.fasta

./group236/reference/d1byka_-d1jyea_.fasta

./group236/reference/d1byka_-d1pea__.fasta

./group236/reference/d1byka_-d1rpja_.fasta

./group236/reference/d1byka_-d2dri__.fasta

./group236/reference/d1byka_-d2liv__.fasta

./group236/reference/d1byka_-d8abp__.fasta

./group236/reference/d1dp4a_-d1gca__.fasta

./group236/reference/d1dp4a_-d1jx6a_.fasta

./group236/reference/d1dp4a_-d1jyea_.fasta

./group236/reference/d1dp4a_-d1pea__.fasta

./group236/reference/d1dp4a_-d1rpja_.fasta

./group236/reference/d1dp4a_-d2dri__.fasta

./group236/reference/d1dp4a_-d2liv__.fasta

./group236/reference/d1gca__-d1jx6a_.fasta

./group236/reference/d1gca__-d1pea__.fasta

./group236/reference/d1jx6a_-d1jyea_.fasta

./group236/reference/d1jx6a_-d1pea__.fasta

./group236/reference/d1jx6a_-d1rpja_.fasta

./group236/reference/d1jx6a_-d2liv__.fasta

./group236/reference/d1jx6a_-d8abp__.fasta

./group236/reference/d1jyea_-d1rpja_.fasta

./group236/reference/d1jyea_-d8abp__.fasta

./group236/reference/d1pea__-d1rpja_.fasta

./group236/reference/d1pea__-d2dri__.fasta

./group236/reference/d1pea__-d2liv__.fasta

./group236/reference/d1pea__-d8abp__.fasta

./group236/reference/d1rpja_-d2liv__.fasta

./group236/reference/d2dri__-d2liv__.fasta

./group236/reference/d2liv__-d8abp__.fasta

./group237/reference/d1a8e__-d1a99a_.fasta

./group237/reference/d1a8e__-d1amf__.fasta

./group237/reference/d1a8e__-d1atg__.fasta

./group237/reference/d1a8e__-d1eu8a_.fasta

./group237/reference/d1a8e__-d1i6aa_.fasta

./group237/reference/d1a8e__-d1ixh__.fasta

./group237/reference/d1a8e__-d1lst__.fasta

./group237/reference/d1a8e__-d1wdna_.fasta

./group237/reference/d1a8e__-d3mbp__.fasta

./group237/reference/d1a8e__-d3thia_.fasta

./group237/reference/d1a99a_-d1al3__.fasta

./group237/reference/d1a99a_-d1amf__.fasta

./group237/reference/d1a99a_-d1atg__.fasta

./group237/reference/d1a99a_-d1eu8a_.fasta

./group237/reference/d1a99a_-d1i6aa_.fasta

./group237/reference/d1a99a_-d1ixh__.fasta

./group237/reference/d1a99a_-d1jeta_.fasta

./group237/reference/d1a99a_-d1mqda_.fasta

./group237/reference/d1a99a_-d1sbp__.fasta

./group237/reference/d1a99a_-d3thia_.fasta

./group237/reference/d1al3__-d1amf__.fasta

./group237/reference/d1al3__-d1dpe__.fasta

./group237/reference/d1al3__-d1eu8a_.fasta

./group237/reference/d1al3__-d1ixh__.fasta

./group237/reference/d1al3__-d1lst__.fasta

./group237/reference/d1al3__-d1mqda_.fasta

./group237/reference/d1al3__-d1pot__.fasta

./group237/reference/d1al3__-d1sbp__.fasta

./group237/reference/d1al3__-d1wdna_.fasta

./group237/reference/d1al3__-d3mbp__.fasta

./group237/reference/d1al3__-d3thia_.fasta

./group237/reference/d1amf__-d1dpe__.fasta

./group237/reference/d1amf__-d1eu8a_.fasta

./group237/reference/d1amf__-d1ixh__.fasta

./group237/reference/d1amf__-d1j1na_.fasta

./group237/reference/d1amf__-d1lst__.fasta

./group237/reference/d1amf__-d1mqda_.fasta

./group237/reference/d1amf__-d1pot__.fasta

./group237/reference/d1amf__-d1sbp__.fasta

./group237/reference/d1amf__-d1wdna_.fasta

./group237/reference/d1amf__-d3mbp__.fasta

./group237/reference/d1amf__-d3thia_.fasta

./group237/reference/d1atg__-d1dpe__.fasta

./group237/reference/d1atg__-d1eu8a_.fasta

./group237/reference/d1atg__-d1ixh__.fasta

./group237/reference/d1atg__-d1j1na_.fasta

./group237/reference/d1atg__-d1lst__.fasta

./group237/reference/d1atg__-d1pot__.fasta

./group237/reference/d1atg__-d1sbp__.fasta

./group237/reference/d1atg__-d3mbp__.fasta

./group237/reference/d1atg__-d3thia_.fasta

./group237/reference/d1dpe__-d1pot__.fasta

./group237/reference/d1dpe__-d1sbp__.fasta

./group237/reference/d1dpe__-d3mbp__.fasta

./group237/reference/d1eu8a_-d1i6aa_.fasta

./group237/reference/d1eu8a_-d1ixh__.fasta

./group237/reference/d1eu8a_-d1j1na_.fasta

./group237/reference/d1eu8a_-d1lst__.fasta

./group237/reference/d1eu8a_-d1pot__.fasta

./group237/reference/d1eu8a_-d1sbp__.fasta

./group237/reference/d1eu8a_-d1wdna_.fasta

./group237/reference/d1eu8a_-d3thia_.fasta

./group237/reference/d1i6aa_-d1jeta_.fasta

./group237/reference/d1i6aa_-d1lst__.fasta

./group237/reference/d1i6aa_-d1mqda_.fasta

./group237/reference/d1i6aa_-d1pot__.fasta

./group237/reference/d1i6aa_-d1sbp__.fasta

./group237/reference/d1i6aa_-d1wdna_.fasta

./group237/reference/d1i6aa_-d3mbp__.fasta

./group237/reference/d1ixh__-d1j1na_.fasta

./group237/reference/d1ixh__-d1lst__.fasta

./group237/reference/d1ixh__-d1mqda_.fasta

./group237/reference/d1ixh__-d1pot__.fasta

./group237/reference/d1ixh__-d1sbp__.fasta

./group237/reference/d1ixh__-d1wdna_.fasta

./group237/reference/d1ixh__-d3mbp__.fasta

./group237/reference/d1ixh__-d3thia_.fasta

./group237/reference/d1j1na_-d1jeta_.fasta

./group237/reference/d1j1na_-d1lst__.fasta

./group237/reference/d1j1na_-d1sbp__.fasta

./group237/reference/d1j1na_-d1wdna_.fasta

./group237/reference/d1j1na_-d3mbp__.fasta

./group237/reference/d1j1na_-d3thia_.fasta

./group237/reference/d1jeta_-d1lst__.fasta

./group237/reference/d1jeta_-d1mqda_.fasta

./group237/reference/d1jeta_-d1wdna_.fasta

./group237/reference/d1jeta_-d3mbp__.fasta

./group237/reference/d1jeta_-d3thia_.fasta

./group237/reference/d1lst__-d1sbp__.fasta

./group237/reference/d1lst__-d3mbp__.fasta

./group237/reference/d1lst__-d3thia_.fasta

./group237/reference/d1mqda_-d1pot__.fasta

./group237/reference/d1mqda_-d1sbp__.fasta

./group237/reference/d1pot__-d1sbp__.fasta

./group237/reference/d1pot__-d1wdna_.fasta

./group237/reference/d1pot__-d3mbp__.fasta

./group237/reference/d1pot__-d3thia_.fasta

./group237/reference/d1sbp__-d1wdna_.fasta

./group237/reference/d1sbp__-d3mbp__.fasta

./group237/reference/d1sbp__-d3thia_.fasta

./group237/reference/d1wdna_-d3mbp__.fasta

./group237/reference/d3mbp__-d3thia_.fasta

./group238/reference/d1afwa2-d1bi5a2.fasta

./group238/reference/d1afwa2-d1e5ma2.fasta

./group238/reference/d1afwa2-d1ek4a2.fasta

./group238/reference/d1afwa2-d1hnja1.fasta

./group238/reference/d1afwa2-d1hnja2.fasta

./group238/reference/d1afwa2-d1hzpa1.fasta

./group238/reference/d1afwa2-d1hzpa2.fasta

./group238/reference/d1afwa2-d1mzja1.fasta

./group238/reference/d1afwa2-d1mzja2.fasta

./group238/reference/d1afwa2-d1ox0a2.fasta

./group238/reference/d1afwa2-d1ub7a2.fasta

./group238/reference/d1bi5a2-d1e5ma2.fasta

./group238/reference/d1bi5a2-d1ek4a1.fasta

./group238/reference/d1bi5a2-d1ek4a2.fasta

./group238/reference/d1bi5a2-d1hnja1.fasta

./group238/reference/d1bi5a2-d1hzpa1.fasta

./group238/reference/d1bi5a2-d1hzpa2.fasta

./group238/reference/d1bi5a2-d1kas_1.fasta

./group238/reference/d1bi5a2-d1m3ka1.fasta

./group238/reference/d1bi5a2-d1m3ka2.fasta

./group238/reference/d1bi5a2-d1mzja1.fasta

./group238/reference/d1bi5a2-d1ox0a1.fasta

./group238/reference/d1bi5a2-d1ox0a2.fasta

./group238/reference/d1e5ma1-d1ek4a2.fasta

./group238/reference/d1e5ma1-d1hnja2.fasta

./group238/reference/d1e5ma1-d1hzpa2.fasta

./group238/reference/d1e5ma1-d1m3ka1.fasta

./group238/reference/d1e5ma1-d1m3ka2.fasta

./group238/reference/d1e5ma1-d1mzja2.fasta

./group238/reference/d1e5ma1-d1ox0a2.fasta

./group238/reference/d1e5ma1-d1ub7a2.fasta

./group238/reference/d1e5ma2-d1ek4a1.fasta

./group238/reference/d1e5ma2-d1hnja2.fasta

./group238/reference/d1e5ma2-d1hzpa1.fasta

./group238/reference/d1e5ma2-d1hzpa2.fasta

./group238/reference/d1e5ma2-d1kas_1.fasta

./group238/reference/d1e5ma2-d1m3ka1.fasta

./group238/reference/d1e5ma2-d1m3ka2.fasta

./group238/reference/d1e5ma2-d1mzja2.fasta

./group238/reference/d1e5ma2-d1ox0a1.fasta

./group238/reference/d1ek4a1-d1ek4a2.fasta

./group238/reference/d1ek4a1-d1hnja1.fasta

./group238/reference/d1ek4a1-d1hnja2.fasta

./group238/reference/d1ek4a1-d1hzpa2.fasta

./group238/reference/d1ek4a1-d1m3ka1.fasta

./group238/reference/d1ek4a1-d1m3ka2.fasta

./group238/reference/d1ek4a1-d1mzja1.fasta

./group238/reference/d1ek4a1-d1mzja2.fasta

./group238/reference/d1ek4a1-d1ox0a2.fasta

./group238/reference/d1ek4a1-d1ub7a2.fasta

./group238/reference/d1ek4a2-d1hnja2.fasta

./group238/reference/d1ek4a2-d1hzpa1.fasta

./group238/reference/d1ek4a2-d1hzpa2.fasta

./group238/reference/d1ek4a2-d1kas_1.fasta

./group238/reference/d1ek4a2-d1m3ka2.fasta

./group238/reference/d1ek4a2-d1mzja1.fasta

./group238/reference/d1ek4a2-d1mzja2.fasta

./group238/reference/d1ek4a2-d1ox0a1.fasta

./group238/reference/d1ek4a2-d1ub7a2.fasta

./group238/reference/d1hnja1-d1hnja2.fasta

./group238/reference/d1hnja1-d1hzpa2.fasta

./group238/reference/d1hnja1-d1m3ka1.fasta

./group238/reference/d1hnja1-d1m3ka2.fasta

./group238/reference/d1hnja1-d1mzja2.fasta

./group238/reference/d1hnja1-d1ox0a2.fasta

./group238/reference/d1hnja2-d1hzpa1.fasta

./group238/reference/d1hnja2-d1m3ka1.fasta

./group238/reference/d1hnja2-d1m3ka2.fasta

./group238/reference/d1hnja2-d1ox0a1.fasta

./group238/reference/d1hnja2-d1ox0a2.fasta

./group238/reference/d1hzpa1-d1hzpa2.fasta

./group238/reference/d1hzpa1-d1m3ka2.fasta

./group238/reference/d1hzpa1-d1mzja2.fasta

./group238/reference/d1hzpa1-d1ox0a2.fasta

./group238/reference/d1hzpa1-d1ub7a2.fasta

./group238/reference/d1hzpa2-d1kas_1.fasta

./group238/reference/d1hzpa2-d1m3ka1.fasta

./group238/reference/d1hzpa2-d1m3ka2.fasta

./group238/reference/d1hzpa2-d1mzja1.fasta

./group238/reference/d1hzpa2-d1ox0a1.fasta

./group238/reference/d1hzpa2-d1ox0a2.fasta

./group238/reference/d1kas_1-d1m3ka2.fasta

./group238/reference/d1kas_1-d1mzja2.fasta

./group238/reference/d1kas_1-d1ox0a2.fasta

./group238/reference/d1kas_1-d1ub7a2.fasta

./group238/reference/d1m3ka1-d1m3ka2.fasta

./group238/reference/d1m3ka1-d1mzja1.fasta

./group238/reference/d1m3ka1-d1mzja2.fasta

./group238/reference/d1m3ka1-d1ox0a1.fasta

./group238/reference/d1m3ka1-d1ox0a2.fasta

./group238/reference/d1m3ka2-d1ox0a1.fasta

./group238/reference/d1m3ka2-d1ox0a2.fasta

./group238/reference/d1m3ka2-d1ub7a2.fasta

./group238/reference/d1mzja1-d1mzja2.fasta

./group238/reference/d1mzja1-d1ox0a2.fasta

./group238/reference/d1mzja2-d1ox0a1.fasta

./group238/reference/d1mzja2-d1ox0a2.fasta

./group238/reference/d1ox0a1-d1ox0a2.fasta

./group238/reference/d1ox0a1-d1ub7a2.fasta

./group238/reference/d1ox0a2-d1ub7a2.fasta

./group239/reference/d1aln_1-d1aln_2.fasta

./group239/reference/d1aln_1-d1uaqa_.fasta

./group239/reference/d1aln_2-d1uaqa_.fasta

./group240/reference/d1a2pa_-d1i0va_.fasta

./group240/reference/d1i0va_-d1lnia_.fasta

./group241/reference/d153l__-d1b9oa_.fasta

./group241/reference/d153l__-d1dxja_.fasta

./group241/reference/d153l__-d1gd6a_.fasta

./group241/reference/d153l__-d1k28a3.fasta

./group241/reference/d153l__-d1qgia_.fasta

./group241/reference/d153l__-d2eql__.fasta

./group241/reference/d153l__-d3lzt__.fasta

./group241/reference/d1b9oa_-d1qsaa2.fasta

./group241/reference/d1chka_-d1dxja_.fasta

./group241/reference/d1chka_-d1k28a3.fasta

./group241/reference/d1chka_-d1lw9a_.fasta

./group241/reference/d1chka_-d3lzt__.fasta

./group241/reference/d1dxja_-d1gd6a_.fasta

./group241/reference/d1dxja_-d1lw9a_.fasta

./group241/reference/d1dxja_-d3lzt__.fasta

./group241/reference/d1gd6a_-d1k28a3.fasta

./group241/reference/d1gd6a_-d1lw9a_.fasta

./group241/reference/d1gd6a_-d1qgia_.fasta

./group241/reference/d1gd6a_-d1qsaa2.fasta

./group241/reference/d1k28a3-d1qgia_.fasta

./group241/reference/d1k28a3-d1qsaa2.fasta

./group241/reference/d1k28a3-d3lzt__.fasta

./group241/reference/d1lw9a_-d1qgia_.fasta

./group241/reference/d1lw9a_-d2eql__.fasta

./group241/reference/d1lw9a_-d3lzt__.fasta

./group241/reference/d1qgia_-d1qsaa2.fasta

./group241/reference/d1qgia_-d2eql__.fasta

./group241/reference/d1qgia_-d3lzt__.fasta

./group241/reference/d1qsaa2-d2eql__.fasta

./group241/reference/d1qsaa2-d3lzt__.fasta

./group242/reference/d1avpa_-d1cv8__.fasta

./group242/reference/d1avpa_-d1euva_.fasta

./group242/reference/d1avpa_-d1f13a4.fasta

./group242/reference/d1avpa_-d1g0da4.fasta

./group242/reference/d1avpa_-d1gmya_.fasta

./group242/reference/d1avpa_-d1gx3a_.fasta

./group242/reference/d1avpa_-d1iwda_.fasta

./group242/reference/d1avpa_-d1nbfa_.fasta

./group242/reference/d1avpa_-d1qmya_.fasta

./group242/reference/d1avpa_-d3gcb__.fasta

./group242/reference/d1avpa_-d7pcka_.fasta

./group242/reference/d1cv8__-d1cvza_.fasta

./group242/reference/d1cv8__-d1deua_.fasta

./group242/reference/d1cv8__-d1euva_.fasta

./group242/reference/d1cv8__-d1fh0a_.fasta

./group242/reference/d1cv8__-d1g0da4.fasta

./group242/reference/d1cv8__-d1gmya_.fasta

./group242/reference/d1cv8__-d1iwda_.fasta

./group242/reference/d1cv8__-d1me4a_.fasta

./group242/reference/d1cv8__-d1nbfa_.fasta

./group242/reference/d1cv8__-d3gcb__.fasta

./group242/reference/d1cv8__-d7pcka_.fasta

./group242/reference/d1cvza_-d1e2ta_.fasta

./group242/reference/d1cvza_-d1f13a4.fasta

./group242/reference/d1cvza_-d1g0da4.fasta

./group242/reference/d1cvza_-d1l9na4.fasta

./group242/reference/d1cvza_-d1nbfa_.fasta

./group242/reference/d1cvza_-d1qmya_.fasta

./group242/reference/d1deua_-d1f13a4.fasta

./group242/reference/d1deua_-d1g0da4.fasta

./group242/reference/d1deua_-d1gx3a_.fasta

./group242/reference/d1deua_-d1nbfa_.fasta

./group242/reference/d1deua_-d1qmya_.fasta

./group242/reference/d1deua_-d2cb5a_.fasta

./group242/reference/d1deua_-d3gcb__.fasta

./group242/reference/d1e2ta_-d1euva_.fasta

./group242/reference/d1e2ta_-d1f13a4.fasta

./group242/reference/d1e2ta_-d1fh0a_.fasta

./group242/reference/d1e2ta_-d1g0da4.fasta

./group242/reference/d1e2ta_-d1iu4a_.fasta

./group242/reference/d1e2ta_-d1l9na4.fasta

./group242/reference/d1e2ta_-d1me4a_.fasta

./group242/reference/d1e2ta_-d3gcb__.fasta

./group242/reference/d1euva_-d1fh0a_.fasta

./group242/reference/d1euva_-d1gx3a_.fasta

./group242/reference/d1euva_-d7pcka_.fasta

./group242/reference/d1f13a4-d1fh0a_.fasta

./group242/reference/d1f13a4-d1gmya_.fasta

./group242/reference/d1f13a4-d1gx3a_.fasta

./group242/reference/d1f13a4-d1me4a_.fasta

./group242/reference/d1f13a4-d2cb5a_.fasta

./group242/reference/d1f13a4-d3gcb__.fasta

./group242/reference/d1f13a4-d7pcka_.fasta

./group242/reference/d1fh0a_-d1g0da4.fasta

./group242/reference/d1fh0a_-d1gx3a_.fasta

./group242/reference/d1fh0a_-d1l9na4.fasta

./group242/reference/d1fh0a_-d1nbfa_.fasta

./group242/reference/d1fh0a_-d1qmya_.fasta

./group242/reference/d1fh0a_-d2cb5a_.fasta

./group242/reference/d1g0da4-d1gmya_.fasta

./group242/reference/d1g0da4-d1gx3a_.fasta

./group242/reference/d1g0da4-d1me4a_.fasta

./group242/reference/d1g0da4-d1nbfa_.fasta

./group242/reference/d1g0da4-d3gcb__.fasta

./group242/reference/d1g0da4-d7pcka_.fasta

./group242/reference/d1gmya_-d1l9na4.fasta

./group242/reference/d1gmya_-d1qmya_.fasta

./group242/reference/d1gx3a_-d1iwda_.fasta

./group242/reference/d1gx3a_-d1l9na4.fasta

./group242/reference/d1gx3a_-d1me4a_.fasta

./group242/reference/d1gx3a_-d1qmya_.fasta

./group242/reference/d1gx3a_-d3gcb__.fasta

./group242/reference/d1iu4a_-d7pcka_.fasta

./group242/reference/d1iwda_-d1l9na4.fasta

./group242/reference/d1iwda_-d1nbfa_.fasta

./group242/reference/d1iwda_-d2cb5a_.fasta

./group242/reference/d1iwda_-d3gcb__.fasta

./group242/reference/d1l9na4-d1me4a_.fasta

./group242/reference/d1l9na4-d1nbfa_.fasta

./group242/reference/d1l9na4-d1qmya_.fasta

./group242/reference/d1l9na4-d2cb5a_.fasta

./group242/reference/d1l9na4-d3gcb__.fasta

./group242/reference/d1l9na4-d7pcka_.fasta

./group242/reference/d1me4a_-d1nbfa_.fasta

./group242/reference/d1me4a_-d2cb5a_.fasta

./group242/reference/d1nbfa_-d1qmya_.fasta

./group242/reference/d1nbfa_-d2cb5a_.fasta

./group242/reference/d1nbfa_-d7pcka_.fasta

./group242/reference/d1qmya_-d2cb5a_.fasta

./group242/reference/d1qmya_-d3gcb__.fasta

./group243/reference/d1a73a_-d1ql0a_.fasta

./group243/reference/d1e7la2-d1fr2b_.fasta

./group243/reference/d1e7la2-d1ql0a_.fasta

./group243/reference/d1fr2b_-d1ql0a_.fasta

./group245/reference/d1b3aa_-d1tvxa_.fasta

./group245/reference/d1doka_-d1qg7a_.fasta

./group245/reference/d1el0a_-d1f2la_.fasta

./group245/reference/d1el0a_-d1m8aa_.fasta

./group245/reference/d1el0a_-d1qg7a_.fasta

./group245/reference/d1el0a_-d1tvxa_.fasta

./group245/reference/d1f2la_-d1tvxa_.fasta

./group245/reference/d1g2ta_-d1qg7a_.fasta

./group245/reference/d1g2ta_-d3il8__.fasta

./group245/reference/d1j9oa_-d1qg7a_.fasta

./group245/reference/d1m8aa_-d1qg7a_.fasta

./group246/reference/d1bf4a_-d1dz1a_.fasta

./group246/reference/d1bf4a_-d1g6za_.fasta

./group246/reference/d1bf4a_-d1knaa_.fasta

./group246/reference/d1dz1a_-d1g6za_.fasta

./group247/reference/d1bb8__-d1d9na_.fasta

./group247/reference/d1bb8__-d1gcca_.fasta

./group247/reference/d1bb8__-d1kjka_.fasta

./group247/reference/d1d9na_-d1kjka_.fasta

./group247/reference/d1gcca_-d1kjka_.fasta

./group247/reference/d1kjka_-d1qk9a_.fasta

./group248/reference/d1jj2l_-d1jj2r_.fasta

./group248/reference/d1jj2l_-d1n88a_.fasta

./group249/reference/d1guqa2-d1kpf__.fasta

./group250/reference/d1a6f__-d1b63a1.fasta

./group250/reference/d1a6f__-d1n0ua3.fasta

./group250/reference/d1a6f__-d1pkp_1.fasta

./group250/reference/d1b63a1-d1fjgi_.fasta

./group250/reference/d1b63a1-d1kija1.fasta

./group250/reference/d1b63a1-d1kkha1.fasta

./group250/reference/d1b63a1-d1mu5a2.fasta

./group250/reference/d1b63a1-d1ueka1.fasta

./group250/reference/d1d6ta_-d1fjgi_.fasta

./group250/reference/d1d6ta_-d1h72c1.fasta

./group250/reference/d1d6ta_-d1n0ua3.fasta

./group250/reference/d1d6ta_-d1pkp_1.fasta

./group250/reference/d1dar_3-d1h72c1.fasta

./group250/reference/d1dar_3-d1kija1.fasta

./group250/reference/d1dar_3-d1kkha1.fasta

./group250/reference/d1dar_3-d1mu5a2.fasta

./group250/reference/d1dar_3-d1n0ua3.fasta

./group250/reference/d1dar_3-d1p42a1.fasta

./group250/reference/d1dar_3-d1p42a2.fasta

./group250/reference/d1dar_3-d1pkp_1.fasta

./group250/reference/d1dar_3-d1ueka1.fasta

./group250/reference/d1ei1a1-d1fjgi_.fasta

./group250/reference/d1ei1a1-d1kkha1.fasta

./group250/reference/d1ei1a1-d1mu5a2.fasta

./group250/reference/d1ei1a1-d1ueka1.fasta

./group250/reference/d1fjgi_-d1h72c1.fasta

./group250/reference/d1fjgi_-d1kija1.fasta

./group250/reference/d1fjgi_-d1kkha1.fasta

./group250/reference/d1fjgi_-d1n0ua3.fasta

./group250/reference/d1fjgi_-d1p42a1.fasta

./group250/reference/d1fjgi_-d1p42a2.fasta

./group250/reference/d1fjgi_-d1pkp_1.fasta

./group250/reference/d1fjgi_-d1ueka1.fasta

./group250/reference/d1h72c1-d1n0ua3.fasta

./group250/reference/d1h72c1-d1p42a1.fasta

./group250/reference/d1h72c1-d1p42a2.fasta

./group250/reference/d1h72c1-d1pkp_1.fasta

./group250/reference/d1h72c1-d1ueka1.fasta

./group250/reference/d1kija1-d1kkha1.fasta

./group250/reference/d1kija1-d1mu5a2.fasta

./group250/reference/d1kija1-d1ueka1.fasta

./group250/reference/d1kkha1-d1n0ua3.fasta

./group250/reference/d1kkha1-d1p42a1.fasta

./group250/reference/d1kkha1-d1p42a2.fasta

./group250/reference/d1kkha1-d1ueka1.fasta

./group250/reference/d1mu5a2-d1p42a1.fasta

./group250/reference/d1n0ua3-d1p42a1.fasta

./group250/reference/d1n0ua3-d1p42a2.fasta

./group250/reference/d1p42a1-d1p42a2.fasta

./group250/reference/d1p42a1-d1ueka1.fasta

./group250/reference/d1p42a2-d1pkp_1.fasta

./group250/reference/d1p42a2-d1ueka1.fasta

./group251/reference/d1a5r__-d1gg3a3.fasta

./group251/reference/d1a5r__-d1gnua_.fasta

./group251/reference/d1a5r__-d1h4ra3.fasta

./group251/reference/d1a5r__-d1j8ca_.fasta

./group251/reference/d1a5r__-d1lm8b_.fasta

./group251/reference/d1a5r__-d1m94a_.fasta

./group251/reference/d1c1yb_-d1gg3a3.fasta

./group251/reference/d1c1yb_-d1h4ra3.fasta

./group251/reference/d1c1yb_-d1h8ca_.fasta

./group251/reference/d1c1yb_-d1j8ca_.fasta

./group251/reference/d1c1yb_-d1l7ya_.fasta

./group251/reference/d1c1yb_-d1lfda_.fasta

./group251/reference/d1c1yb_-d1m94a_.fasta

./group251/reference/d1c1yb_-d1mg8a_.fasta

./group251/reference/d1c1yb_-d1rlf__.fasta

./group251/reference/d1euvb_-d1gnua_.fasta

./group251/reference/d1euvb_-d1h4ra3.fasta

./group251/reference/d1euvb_-d1j8ca_.fasta

./group251/reference/d1euvb_-d1lm8b_.fasta

./group251/reference/d1euvb_-d1m94a_.fasta

./group251/reference/d1gg3a3-d1l7ya_.fasta

./group251/reference/d1gg3a3-d1lfda_.fasta

./group251/reference/d1gg3a3-d1lm8b_.fasta

./group251/reference/d1gg3a3-d1mg8a_.fasta

./group251/reference/d1gg3a3-d1rlf__.fasta

./group251/reference/d1gnua_-d1l7ya_.fasta

./group251/reference/d1gnua_-d1lfda_.fasta

./group251/reference/d1gnua_-d1rlf__.fasta

./group251/reference/d1h4ra3-d1i42a_.fasta

./group251/reference/d1h4ra3-d1j8ca_.fasta

./group251/reference/d1h4ra3-d1lfda_.fasta

./group251/reference/d1h4ra3-d1lm8b_.fasta

./group251/reference/d1h4ra3-d1m94a_.fasta

./group251/reference/d1h4ra3-d1mg8a_.fasta

./group251/reference/d1h4ra3-d1rlf__.fasta

./group251/reference/d1h8ca_-d1j8ca_.fasta

./group251/reference/d1h8ca_-d1l7ya_.fasta

./group251/reference/d1h8ca_-d1lfda_.fasta

./group251/reference/d1h8ca_-d1m94a_.fasta

./group251/reference/d1h8ca_-d1mg8a_.fasta

./group251/reference/d1h8ca_-d1rlf__.fasta

./group251/reference/d1i42a_-d1j8ca_.fasta

./group251/reference/d1i42a_-d1l7ya_.fasta

./group251/reference/d1i42a_-d1m94a_.fasta

./group251/reference/d1i42a_-d1mg8a_.fasta

./group251/reference/d1i42a_-d1rlf__.fasta

./group251/reference/d1j8ca_-d1l7ya_.fasta

./group251/reference/d1j8ca_-d1rlf__.fasta

./group251/reference/d1l7ya_-d1lfda_.fasta

./group251/reference/d1l7ya_-d1lm8b_.fasta

./group251/reference/d1l7ya_-d1mg8a_.fasta

./group251/reference/d1lfda_-d1mg8a_.fasta

./group251/reference/d1lm8b_-d1m94a_.fasta

./group251/reference/d1lm8b_-d1mg8a_.fasta

./group251/reference/d1lm8b_-d1rlf__.fasta

./group251/reference/d1m94a_-d1mg8a_.fasta

./group251/reference/d1m94a_-d1rlf__.fasta

./group251/reference/d1mg8a_-d1rlf__.fasta

./group252/reference/d1c9fa_-d1ip9a_.fasta

./group252/reference/d1c9fa_-d1pqsa_.fasta

./group252/reference/d1d4ba_-d1ip9a_.fasta

./group252/reference/d1f2ri_-d1ip9a_.fasta

./group252/reference/d1ip9a_-d1pqsa_.fasta

./group253/reference/d1fm0d_-d1jsba_.fasta

./group254/reference/d1ayfa_-d1doi__.fasta

./group254/reference/d1ayfa_-d1feha2.fasta

./group254/reference/d1ayfa_-d1fo4a2.fasta

./group254/reference/d1ayfa_-d1hlra2.fasta

./group254/reference/d1ayfa_-d1jq4a_.fasta

./group254/reference/d1ayfa_-d1jroa2.fasta

./group254/reference/d1ayfa_-d1kf6b2.fasta

./group254/reference/d1ayfa_-d1n62a2.fasta

./group254/reference/d1ayfa_-d1nekb2.fasta

./group254/reference/d1ayfa_-d1qlab2.fasta

./group254/reference/d1ayfa_-d2pia_3.fasta

./group254/reference/d1b9ra_-d1czpa_.fasta

./group254/reference/d1b9ra_-d1fo4a2.fasta

./group254/reference/d1b9ra_-d1hlra2.fasta

./group254/reference/d1b9ra_-d1jq4a_.fasta

./group254/reference/d1b9ra_-d1jroa2.fasta

./group254/reference/d1b9ra_-d1kf6b2.fasta

./group254/reference/d1b9ra_-d1krha3.fasta

./group254/reference/d1b9ra_-d1n62a2.fasta

./group254/reference/d1b9ra_-d1nekb2.fasta

./group254/reference/d1b9ra_-d2pia_3.fasta

./group254/reference/d1czpa_-d1fo4a2.fasta

./group254/reference/d1czpa_-d1jroa2.fasta

./group254/reference/d1czpa_-d1n62a2.fasta

./group254/reference/d1czpa_-d1nekb2.fasta

./group254/reference/d1czpa_-d1qlab2.fasta

./group254/reference/d1doi__-d1fo4a2.fasta

./group254/reference/d1doi__-d1jroa2.fasta

./group254/reference/d1doi__-d1l5pa_.fasta

./group254/reference/d1doi__-d1n62a2.fasta

./group254/reference/d1doi__-d1nekb2.fasta

./group254/reference/d1doi__-d1qlab2.fasta

./group254/reference/d1e9ma_-d1fo4a2.fasta

./group254/reference/d1e9ma_-d1hlra2.fasta

./group254/reference/d1e9ma_-d1jroa2.fasta

./group254/reference/d1e9ma_-d1kf6b2.fasta

./group254/reference/d1e9ma_-d1krha3.fasta

./group254/reference/d1e9ma_-d1n62a2.fasta

./group254/reference/d1e9ma_-d1qlab2.fasta

./group254/reference/d1e9ma_-d2pia_3.fasta

./group254/reference/d1feha2-d1kf6b2.fasta

./group254/reference/d1feha2-d1krha3.fasta

./group254/reference/d1feha2-d1l5pa_.fasta

./group254/reference/d1feha2-d1nekb2.fasta

./group254/reference/d1fo4a2-d1i7ha_.fasta

./group254/reference/d1fo4a2-d1jq4a_.fasta

./group254/reference/d1fo4a2-d1krha3.fasta

./group254/reference/d1fo4a2-d1l5pa_.fasta

./group254/reference/d1fo4a2-d1put__.fasta

./group254/reference/d1fo4a2-d1qlab2.fasta

./group254/reference/d1fo4a2-d2pia_3.fasta

./group254/reference/d1hlra2-d1i7ha_.fasta

./group254/reference/d1hlra2-d1jq4a_.fasta

./group254/reference/d1hlra2-d1krha3.fasta

./group254/reference/d1hlra2-d1put__.fasta

./group254/reference/d1i7ha_-d1l5pa_.fasta

./group254/reference/d1i7ha_-d1n62a2.fasta

./group254/reference/d1i7ha_-d1nekb2.fasta

./group254/reference/d1i7ha_-d1qlab2.fasta

./group254/reference/d1i7ha_-d2pia_3.fasta

./group254/reference/d1jq4a_-d1jroa2.fasta

./group254/reference/d1jq4a_-d1kf6b2.fasta

./group254/reference/d1jq4a_-d1put__.fasta

./group254/reference/d1jq4a_-d2pia_3.fasta

./group254/reference/d1jroa2-d1krha3.fasta

./group254/reference/d1jroa2-d1nekb2.fasta

./group254/reference/d1jroa2-d1put__.fasta

./group254/reference/d1jroa2-d1qlab2.fasta

./group254/reference/d1kf6b2-d1krha3.fasta

./group254/reference/d1kf6b2-d1n62a2.fasta

./group254/reference/d1kf6b2-d2pia_3.fasta

./group254/reference/d1krha3-d1n62a2.fasta

./group254/reference/d1krha3-d1nekb2.fasta

./group254/reference/d1krha3-d1qlab2.fasta

./group254/reference/d1l5pa_-d1n62a2.fasta

./group254/reference/d1l5pa_-d1nekb2.fasta

./group254/reference/d1l5pa_-d2pia_3.fasta

./group254/reference/d1n62a2-d1nekb2.fasta

./group254/reference/d1n62a2-d1put__.fasta

./group254/reference/d1n62a2-d1qlab2.fasta

./group254/reference/d1n62a2-d2pia_3.fasta

./group254/reference/d1nekb2-d1put__.fasta

./group254/reference/d1nekb2-d2pia_3.fasta

./group254/reference/d1put__-d1qlab2.fasta

./group254/reference/d1put__-d2pia_3.fasta

./group255/reference/d1bmlc3-d1qqra_.fasta

./group255/reference/d1qqra_-d2sak__.fasta

./group256/reference/d1esfa2-d1m4va2.fasta

./group256/reference/d1esfa2-d3tss_2.fasta

./group256/reference/d1et9a2-d1m4va2.fasta

./group256/reference/d1eu3a2-d1m4va2.fasta

./group257/reference/d1an9a2-d1b5qa2.fasta

./group257/reference/d1an9a2-d1gosa2.fasta

./group257/reference/d1an9a2-d1i8ta2.fasta

./group257/reference/d1an9a2-d1ju2a2.fasta

./group257/reference/d1an9a2-d1l9ea2.fasta

./group257/reference/d1an9a2-d1mxta2.fasta

./group257/reference/d1an9a2-d1ng4a2.fasta

./group257/reference/d1b5qa2-d1f8ra2.fasta

./group257/reference/d1b5qa2-d1k0ia2.fasta

./group257/reference/d1b5qa2-d1kdga2.fasta

./group257/reference/d1b5qa2-d1ng4a2.fasta

./group257/reference/d1c0pa2-d1d5ta2.fasta

./group257/reference/d1c0pa2-d1i8ta2.fasta

./group257/reference/d1c0pa2-d1k0ia2.fasta

./group257/reference/d1c0pa2-d1kdga2.fasta

./group257/reference/d1c0pa2-d1l9ea2.fasta

./group257/reference/d1d5ta2-d1gosa2.fasta

./group257/reference/d1d5ta2-d1ju2a2.fasta

./group257/reference/d1d5ta2-d1l9ea2.fasta

./group257/reference/d1d5ta2-d1mxta2.fasta

./group257/reference/d1f8ra2-d1gosa2.fasta

./group257/reference/d1f8ra2-d1i8ta2.fasta

./group257/reference/d1f8ra2-d1ng4a2.fasta

./group257/reference/d1gosa2-d1gpea2.fasta

./group257/reference/d1gosa2-d1l9ea2.fasta

./group257/reference/d1gpea2-d1ju2a2.fasta

./group257/reference/d1gpea2-d1kdga2.fasta

./group257/reference/d1gpea2-d1mxta2.fasta

./group257/reference/d1ju2a2-d1kdga2.fasta

./group257/reference/d1k0ia2-d1l9ea2.fasta

./group257/reference/d1k0ia2-d1mxta2.fasta

./group257/reference/d1kdga2-d1l9ea2.fasta

./group257/reference/d1kdga2-d1mxta2.fasta

./group257/reference/d1l9ea2-d1ng4a2.fasta

./group257/reference/d1mxta2-d1ng4a2.fasta

./group258/reference/d1mola_-d1stfi_.fasta

./group259/reference/d1a2va2-d1a2va3.fasta

./group259/reference/d1a2va2-d1ivwa3.fasta

./group259/reference/d1a2va2-d1ksia3.fasta

./group259/reference/d1a2va2-d1oaca3.fasta

./group259/reference/d1a2va3-d1ksia2.fasta

./group259/reference/d1a2va3-d1ksia3.fasta

./group259/reference/d1a2va3-d1oaca2.fasta

./group259/reference/d1ivwa2-d1ivwa3.fasta

./group259/reference/d1ivwa2-d1ksia2.fasta

./group259/reference/d1ivwa2-d1ksia3.fasta

./group259/reference/d1ivwa2-d1oaca3.fasta

./group259/reference/d1ivwa3-d1ksia2.fasta

./group259/reference/d1ivwa3-d1ksia3.fasta

./group259/reference/d1ksia2-d1ksia3.fasta

./group259/reference/d1ksia2-d1oaca3.fasta

./group259/reference/d1ksia3-d1oaca2.fasta

./group260/reference/d1gy6a_-d1idpa_.fasta

./group260/reference/d1gy6a_-d1m98a2.fasta

./group260/reference/d1gy6a_-d1mwxa1.fasta

./group260/reference/d1gy6a_-d1ocva_.fasta

./group260/reference/d1gy7a_-d1m98a2.fasta

./group260/reference/d1gy7a_-d1nwwa_.fasta

./group260/reference/d1gy7a_-d1ocva_.fasta

./group260/reference/d1gy7a_-d1oh0a_.fasta

./group260/reference/d1idpa_-d1jkga_.fasta

./group260/reference/d1idpa_-d1m98a2.fasta

./group260/reference/d1idpa_-d1mwxa1.fasta

./group260/reference/d1idpa_-d1nwwa_.fasta

./group260/reference/d1idpa_-d1o7nb_.fasta

./group260/reference/d1jkga_-d1m98a2.fasta

./group260/reference/d1jkga_-d1mwxa1.fasta

./group260/reference/d1jkga_-d1nwwa_.fasta

./group260/reference/d1jkga_-d1ocva_.fasta

./group260/reference/d1jkga_-d1oh0a_.fasta

./group260/reference/d1m98a2-d1oh0a_.fasta

./group260/reference/d1nwwa_-d1ocva_.fasta

./group260/reference/d1nwwa_-d1oh0a_.fasta

./group260/reference/d1o7nb_-d1oh0a_.fasta

./group261/reference/d1c16a2-d1cd1a2.fasta

./group261/reference/d1c16a2-d1fngb2.fasta

./group261/reference/d1c16a2-d1gzqa2.fasta

./group261/reference/d1c16a2-d1hdma2.fasta

./group261/reference/d1c16a2-d1iaka2.fasta

./group261/reference/d1c16a2-d1jfma_.fasta

./group261/reference/d1c16a2-d1lqva_.fasta

./group261/reference/d1cd1a2-d1de4a2.fasta

./group261/reference/d1cd1a2-d1hyrc2.fasta

./group261/reference/d1cd1a2-d1k5na2.fasta

./group261/reference/d1cd1a2-d1zaga2.fasta

./group261/reference/d1de4a2-d1fnga2.fasta

./group261/reference/d1de4a2-d1fngb2.fasta

./group261/reference/d1de4a2-d1gzqa2.fasta

./group261/reference/d1de4a2-d1hdma2.fasta

./group261/reference/d1de4a2-d1iaka2.fasta

./group261/reference/d1de4a2-d1jfma_.fasta

./group261/reference/d1de4a2-d1lqva_.fasta

./group261/reference/d1fnga2-d1fngb2.fasta

./group261/reference/d1fnga2-d1hdma2.fasta

./group261/reference/d1fnga2-d1hdmb2.fasta

./group261/reference/d1fnga2-d1hyrc2.fasta

./group261/reference/d1fnga2-d1jfma_.fasta

./group261/reference/d1fnga2-d1k5na2.fasta

./group261/reference/d1fnga2-d1zaga2.fasta

./group261/reference/d1fnga2-d3frua2.fasta

./group261/reference/d1fngb2-d1gzqa2.fasta

./group261/reference/d1fngb2-d1hdma2.fasta

./group261/reference/d1fngb2-d1hdmb2.fasta

./group261/reference/d1fngb2-d1hyrc2.fasta

./group261/reference/d1fngb2-d1iaka2.fasta

./group261/reference/d1fngb2-d1k5na2.fasta

./group261/reference/d1fngb2-d1lqva_.fasta

./group261/reference/d1fngb2-d1zaga2.fasta

./group261/reference/d1fngb2-d3frua2.fasta

./group261/reference/d1gzqa2-d1hdmb2.fasta

./group261/reference/d1gzqa2-d1hyrc2.fasta

./group261/reference/d1gzqa2-d1iaka2.fasta

./group261/reference/d1gzqa2-d1jfma_.fasta

./group261/reference/d1gzqa2-d3frua2.fasta

./group261/reference/d1hdma2-d1hdmb2.fasta

./group261/reference/d1hdma2-d1hyrc2.fasta

./group261/reference/d1hdma2-d1jfma_.fasta

./group261/reference/d1hdma2-d1k5na2.fasta

./group261/reference/d1hdma2-d1lqva_.fasta

./group261/reference/d1hdma2-d1zaga2.fasta

./group261/reference/d1hdmb2-d1iaka2.fasta

./group261/reference/d1hdmb2-d1jfma_.fasta

./group261/reference/d1hdmb2-d1lqva_.fasta

./group261/reference/d1hdmb2-d1zaga2.fasta

./group261/reference/d1hdmb2-d3frua2.fasta

./group261/reference/d1hyrc2-d1jfma_.fasta

./group261/reference/d1hyrc2-d1lqva_.fasta

./group261/reference/d1iaka2-d1k5na2.fasta

./group261/reference/d1iaka2-d1zaga2.fasta

./group261/reference/d1jfma_-d1k5na2.fasta

./group261/reference/d1jfma_-d1lqva_.fasta

./group261/reference/d1jfma_-d1zaga2.fasta

./group261/reference/d1jfma_-d3frua2.fasta

./group261/reference/d1k5na2-d1lqva_.fasta

./group261/reference/d1lqva_-d1zaga2.fasta

./group261/reference/d1lqva_-d3frua2.fasta

./group262/reference/d1fzya_-d1kppa_.fasta

./group262/reference/d1i7ka_-d1kppa_.fasta

./group262/reference/d1j7da_-d1kppa_.fasta

./group262/reference/d1jata_-d1kppa_.fasta

./group262/reference/d1kppa_-d1qcqa_.fasta

./group262/reference/d1kppa_-d2aak__.fasta

./group262/reference/d1kppa_-d2ucz__.fasta

./group263/reference/d1dzoa_-d1hpwa_.fasta

./group263/reference/d1dzoa_-d1oqva_.fasta

./group264/reference/d1bkf__-d1j6ya_.fasta

./group264/reference/d1bkf__-d1jnsa_.fasta

./group264/reference/d1bkf__-d1m5ya2.fasta

./group264/reference/d1bkf__-d1m5ya3.fasta

./group264/reference/d1eq3a_-d1fd9a_.fasta

./group264/reference/d1fd9a_-d1hxva_.fasta

./group264/reference/d1fd9a_-d1j6ya_.fasta

./group264/reference/d1fd9a_-d1jnsa_.fasta

./group264/reference/d1fd9a_-d1m5ya3.fasta

./group264/reference/d1fd9a_-d1pina2.fasta

./group264/reference/d1hxva_-d1jnsa_.fasta

./group264/reference/d1hxva_-d1kt1a3.fasta

./group264/reference/d1hxva_-d1pina2.fasta

./group264/reference/d1ix5a_-d1kt1a3.fasta

./group264/reference/d1ix5a_-d1m5ya2.fasta

./group264/reference/d1ix5a_-d1m5ya3.fasta

./group264/reference/d1ix5a_-d1pina2.fasta

./group264/reference/d1j6ya_-d1jvwa_.fasta

./group264/reference/d1j6ya_-d1l1pa_.fasta

./group264/reference/d1j6ya_-d1pbk__.fasta

./group264/reference/d1jnsa_-d1kt1a3.fasta

./group264/reference/d1jnsa_-d1l1pa_.fasta

./group264/reference/d1jvwa_-d1l1pa_.fasta

./group264/reference/d1jvwa_-d1m5ya2.fasta

./group264/reference/d1jvwa_-d1m5ya3.fasta

./group264/reference/d1kt1a3-d1pina2.fasta

./group264/reference/d1l1pa_-d1m5ya3.fasta

./group264/reference/d1l1pa_-d1pina2.fasta

./group264/reference/d1m5ya2-d1pbk__.fasta

./group264/reference/d1m5ya3-d1pbk__.fasta

./group264/reference/d1pbk__-d1pina2.fasta

./group265/reference/d1edqa3-d1jnda2.fasta

./group265/reference/d1goia3-d1kfwa2.fasta

./group265/reference/d1itxa2-d1jnda2.fasta

./group265/reference/d1jnda2-d1kfwa2.fasta

./group265/reference/d1jnda2-d1ll7a2.fasta

./group266/reference/d1cjxa1-d1ecsa_.fasta

./group266/reference/d1cjxa1-d1f1ua1.fasta

./group266/reference/d1cjxa1-d1f1ua2.fasta

./group266/reference/d1cjxa1-d1kw3b1.fasta

./group266/reference/d1cjxa1-d1kw3b2.fasta

./group266/reference/d1cjxa1-d1lqpa_.fasta

./group266/reference/d1cjxa1-d1mpya1.fasta

./group266/reference/d1cjxa1-d1qtoa_.fasta

./group266/reference/d1ecsa_-d1f1ua1.fasta

./group266/reference/d1ecsa_-d1f1ua2.fasta

./group266/reference/d1ecsa_-d1mpya1.fasta

./group266/reference/d1ecsa_-d1mpya2.fasta

./group266/reference/d1ecsa_-d1qipa_.fasta

./group266/reference/d1f1ua1-d1f1ua2.fasta

./group266/reference/d1f1ua1-d1kw3b1.fasta

./group266/reference/d1f1ua1-d1mpya2.fasta

./group266/reference/d1f1ua1-d1qtoa_.fasta

./group266/reference/d1f1ua2-d1kw3b1.fasta

./group266/reference/d1f1ua2-d1mpya1.fasta

./group266/reference/d1kw3b1-d1kw3b2.fasta

./group266/reference/d1kw3b1-d1mpya1.fasta

./group266/reference/d1kw3b1-d1mpya2.fasta

./group266/reference/d1kw3b1-d1qtoa_.fasta

./group266/reference/d1kw3b2-d1qtoa_.fasta

./group266/reference/d1mpya1-d1mpya2.fasta

./group266/reference/d1mpya1-d1qipa_.fasta

./group266/reference/d1mpya1-d1qtoa_.fasta

./group266/reference/d1mpya2-d1qipa_.fasta

./group266/reference/d1mpya2-d1qtoa_.fasta

./group266/reference/d1qipa_-d1qtoa_.fasta

./group267/reference/d1c8ua1-d1c8ua2.fasta

./group267/reference/d1c8ua2-d1iq6a_.fasta

./group267/reference/d1c8ua2-d1mkaa_.fasta

./group267/reference/d1iq6a_-d1lo7a_.fasta

./group267/reference/d1iq6a_-d1mkaa_.fasta

./group271/reference/d1buoa_-d1hv2a_.fasta

./group271/reference/d1buoa_-d1nn7a_.fasta

./group271/reference/d1fs1b2-d1nn7a_.fasta

./group274/reference/d1di2a_-d1fjge2.fasta

./group274/reference/d1di2a_-d1kn0a_.fasta

./group274/reference/d1di2a_-d1pkp_2.fasta

./group274/reference/d1fjge2-d1kn0a_.fasta

./group274/reference/d1fjge2-d1qu6a1.fasta

./group274/reference/d1fjge2-d1qu6a2.fasta

./group274/reference/d1fjge2-d1stu__.fasta

./group274/reference/d1kn0a_-d1o0wa2.fasta

./group274/reference/d1kn0a_-d1pkp_2.fasta

./group274/reference/d1kn0a_-d1qu6a1.fasta

./group274/reference/d1kn0a_-d1qu6a2.fasta

./group274/reference/d1kn0a_-d1stu__.fasta

./group274/reference/d1pkp_2-d1stu__.fasta

./group275/reference/d1dtja_-d1vig__.fasta

./group275/reference/d1dtja_-d2fmr__.fasta

./group275/reference/d1j4wa1-d2fmr__.fasta

./group275/reference/d1k1ga_-d1vig__.fasta

./group275/reference/d1k1ga_-d2fmr__.fasta

./group275/reference/d1vig__-d2fmr__.fasta

./group276/reference/d1egaa2-d1k0ra3.fasta

./group276/reference/d1fjgc1-d1k0ra3.fasta

./group276/reference/d1hh2p2-d1k0ra3.fasta

./group277/reference/d1jpma2-d1onea2.fasta

./group277/reference/d1onea2-d2chr_2.fasta

./group277/reference/d2chr_2-d2mnr_2.fasta

./group278/reference/d1blu__-d1h0hb_.fasta

./group278/reference/d1feha3-d1iqza_.fasta

./group278/reference/d1fxd__-d1hfel2.fasta

./group278/reference/d1fxra_-d1h0hb_.fasta

./group278/reference/d1fxra_-d1keka5.fasta

./group278/reference/d1fxra_-d2fdn__.fasta

./group278/reference/d1h0hb_-d1h98a_.fasta

./group278/reference/d1h0hb_-d1jb0c_.fasta

./group278/reference/d1h0hb_-d1keka5.fasta

./group278/reference/d1h0hb_-d7fd1a_.fasta

./group278/reference/d1h98a_-d1iqza_.fasta

./group278/reference/d1iqza_-d1keka5.fasta

./group278/reference/d1iqza_-d7fd1a_.fasta

./group278/reference/d1keka5-d7fd1a_.fasta

./group278/reference/d1kqfb1-d7fd1a_.fasta

./group279/reference/d1jqga2-d1scjb_.fasta

./group279/reference/d1kn6a_-d1kwma2.fasta

./group279/reference/d1kn6a_-d1scjb_.fasta

./group279/reference/d1kwma2-d1scjb_.fasta

./group280/reference/d1lq9a_-d1nwja_.fasta

./group281/reference/d1nh8a2-d1p1la_.fasta

./group281/reference/d1nh8a2-d2pii__.fasta

./group281/reference/d1p1la_-d2pii__.fasta

./group283/reference/d1b7fa2-d1fj7a_.fasta

./group283/reference/d1b7fa2-d1fjeb2.fasta

./group283/reference/d1b7fa2-d1hd1a_.fasta

./group283/reference/d1b7fa2-d1iqta_.fasta

./group283/reference/d1b7fa2-d1o0pa_.fasta

./group283/reference/d1b7fa2-d1owxa_.fasta

./group283/reference/d1b7fa2-d2msta_.fasta

./group283/reference/d1cvja1-d1iqta_.fasta

./group283/reference/d1cvja1-d1o0pa_.fasta

./group283/reference/d1cvja1-d2u2fa_.fasta

./group283/reference/d1cvja2-d1koha2.fasta

./group283/reference/d1cvja2-d1o0pa_.fasta

./group283/reference/d1cvja2-d1qm9a1.fasta

./group283/reference/d1fj7a_-d1fxla2.fasta

./group283/reference/d1fj7a_-d1koha2.fasta

./group283/reference/d1fj7a_-d1l3ka2.fasta

./group283/reference/d1fj7a_-d1oo0b_.fasta

./group283/reference/d1fj7a_-d1owxa_.fasta

./group283/reference/d1fj7a_-d1u2fa_.fasta

./group283/reference/d1fj7a_-d2u1a__.fasta

./group283/reference/d1fj7a_-d2u2fa_.fasta

./group283/reference/d1fjeb2-d1fxla2.fasta

./group283/reference/d1fjeb2-d1koha2.fasta

./group283/reference/d1fjeb2-d1l3ka2.fasta

./group283/reference/d1fjeb2-d1o0pa_.fasta

./group283/reference/d1fjeb2-d1qm9a1.fasta

./group283/reference/d1fxla1-d1u2fa_.fasta

./group283/reference/d1fxla2-d1iqta_.fasta

./group283/reference/d1fxla2-d1u2fa_.fasta

./group283/reference/d1hd1a_-d1oo0b_.fasta

./group283/reference/d1hd1a_-d1qm9a1.fasta

./group283/reference/d1hd1a_-d1qm9a2.fasta

./group283/reference/d1hd1a_-d1u2fa_.fasta

./group283/reference/d1hd1a_-d2u1a__.fasta

./group283/reference/d1iqta_-d1oo0b_.fasta

./group283/reference/d1iqta_-d1qm9a1.fasta

./group283/reference/d1iqta_-d1qm9a2.fasta

./group283/reference/d1iqta_-d1u2fa_.fasta

./group283/reference/d1iqta_-d2u1a__.fasta

./group283/reference/d1iqta_-d2u2fa_.fasta

./group283/reference/d1koha2-d1l3ka1.fasta

./group283/reference/d1koha2-d1nu4a_.fasta

./group283/reference/d1koha2-d1oo0b_.fasta

./group283/reference/d1koha2-d1qm9a1.fasta

./group283/reference/d1koha2-d2u2fa_.fasta

./group283/reference/d1l3ka1-d1owxa_.fasta

./group283/reference/d1l3ka1-d1qm9a1.fasta

./group283/reference/d1l3ka1-d1qm9a2.fasta

./group283/reference/d1l3ka1-d1u2fa_.fasta

./group283/reference/d1l3ka2-d1o0pa_.fasta

./group283/reference/d1l3ka2-d1owxa_.fasta

./group283/reference/d1l3ka2-d1qm9a1.fasta

./group283/reference/d1nu4a_-d1oo0b_.fasta

./group283/reference/d1nu4a_-d1owxa_.fasta

./group283/reference/d1nu4a_-d1u2fa_.fasta

./group283/reference/d1nu4a_-d2msta_.fasta

./group283/reference/d1o0pa_-d1oo0b_.fasta

./group283/reference/d1o0pa_-d1owxa_.fasta

./group283/reference/d1o0pa_-d2u1a__.fasta

./group283/reference/d1o0pa_-d2u2fa_.fasta

./group283/reference/d1oo0b_-d1owxa_.fasta

./group283/reference/d1oo0b_-d1qm9a2.fasta

./group283/reference/d1oo0b_-d2msta_.fasta

./group283/reference/d1owxa_-d1qm9a1.fasta

./group283/reference/d1owxa_-d1qm9a2.fasta

./group283/reference/d1owxa_-d2msta_.fasta

./group283/reference/d1owxa_-d2u2fa_.fasta

./group283/reference/d1qm9a1-d2msta_.fasta

./group283/reference/d1qm9a1-d2u2fa_.fasta

./group283/reference/d1qm9a2-d2msta_.fasta

./group283/reference/d1u2fa_-d2msta_.fasta

./group284/reference/d1dar_4-d1n0ua4.fasta

./group285/reference/d1aw0__-d1mwza_.fasta

./group285/reference/d1cc8a_-d1cpza_.fasta

./group285/reference/d1cc8a_-d1mwza_.fasta

./group285/reference/d1cpza_-d1fe0a_.fasta

./group285/reference/d1fe0a_-d1k0va_.fasta

./group285/reference/d1fe0a_-d1mwza_.fasta

./group286/reference/d1phza1-d1tdj_2.fasta

./group287/reference/d1h72c2-d1kkha2.fasta

./group287/reference/d1h72c2-d1kvka2.fasta

./group287/reference/d1h72c2-d1ueka2.fasta

./group287/reference/d1kkha2-d1ueka2.fasta

./group287/reference/d1kvka2-d1ueka2.fasta

./group289/reference/d1dj0a1-d1dj0a2.fasta

./group289/reference/d1dj0a2-d1k8wa4.fasta

./group290/reference/d1fjgd_-d1h3fa2.fasta

./group294/reference/d1dbfa_-d1onia_.fasta

./group294/reference/d1dbfa_-d1qd9a_.fasta

./group294/reference/d1onia_-d1ufya_.fasta

./group295/reference/d1fsz_2-d1tuba2.fasta

./group295/reference/d1fsz_2-d1tubb2.fasta

./group295/reference/d1ofua2-d1tuba2.fasta

./group295/reference/d1ofua2-d1tubb2.fasta

./group296/reference/d1ck9a_-d1e7ka_.fasta

./group296/reference/d1ck9a_-d1jj2f_.fasta

./group297/reference/d1dpta_-d1gyxa_.fasta

./group297/reference/d1dpta_-d1otfa_.fasta

./group297/reference/d1dpta_-d1otga_.fasta

./group297/reference/d1gd0a_-d1otfa_.fasta

./group297/reference/d1gd0a_-d1otga_.fasta

./group297/reference/d1gyxa_-d1otfa_.fasta

./group297/reference/d1gyxa_-d1otga_.fasta

./group297/reference/d1hfoa_-d1otfa_.fasta

./group297/reference/d1otfa_-d1otga_.fasta

./group298/reference/d1b7go2-d1dih_2.fasta

./group298/reference/d1b7go2-d1ebfa2.fasta

./group298/reference/d1b7go2-d1j5pa3.fasta

./group298/reference/d1b7go2-d1mb4a2.fasta

./group298/reference/d1b7go2-d1nvmb2.fasta

./group298/reference/d1cf2o2-d1ebfa2.fasta

./group298/reference/d1cf2o2-d1f06a2.fasta

./group298/reference/d1cf2o2-d1j5pa3.fasta

./group298/reference/d1cf2o2-d1mb4a2.fasta

./group298/reference/d1dih_2-d1e5qa2.fasta

./group298/reference/d1dih_2-d1mb4a2.fasta

./group298/reference/d1dih_2-d1p1ja2.fasta

./group298/reference/d1e5qa2-d1f06a2.fasta

./group298/reference/d1ebfa2-d1j5pa3.fasta

./group298/reference/d1ebfa2-d1lc0a2.fasta

./group298/reference/d1ebfa2-d1p1ja2.fasta

./group298/reference/d1f06a2-d1j5pa3.fasta

./group298/reference/d1f06a2-d1mb4a2.fasta

./group298/reference/d1h6da2-d1lc0a2.fasta

./group298/reference/d1j5pa3-d1lc0a2.fasta

./group298/reference/d1j5pa3-d1mb4a2.fasta

./group299/reference/d1d7ya3-d1dxla3.fasta

./group299/reference/d1d7ya3-d1ebda3.fasta

./group299/reference/d1d7ya3-d1feca3.fasta

./group299/reference/d1d7ya3-d1h6va3.fasta

./group299/reference/d1d7ya3-d1lvl_3.fasta

./group299/reference/d1d7ya3-d1mo9a3.fasta

./group299/reference/d1d7ya3-d1nhp_3.fasta

./group299/reference/d1d7ya3-d1ojt_3.fasta

./group299/reference/d1d7ya3-d3grs_3.fasta

./group299/reference/d1d7ya3-d3lada3.fasta

./group299/reference/d1dxla3-d1mo9a3.fasta

./group299/reference/d1ebda3-d1fcda3.fasta

./group299/reference/d1feca3-d1mo9a3.fasta

./group299/reference/d1feca3-d1nhp_3.fasta

./group299/reference/d1feca3-d1ojt_3.fasta

./group299/reference/d1h6va3-d1nhp_3.fasta

./group299/reference/d1lvl_3-d1mo9a3.fasta

./group299/reference/d1lvl_3-d1nhp_3.fasta

./group299/reference/d1nhp_3-d3grs_3.fasta

./group299/reference/d1nhp_3-d3lada3.fasta

./group300/reference/d1fo4a4-d1jroa3.fasta

./group301/reference/d1f08a_-d1l2ma_.fasta

./group301/reference/d1l2ma_-d1m55a_.fasta

./group302/reference/d1f5va_-d1vfra_.fasta

./group303/reference/d1ast__-d1atla_.fasta

./group303/reference/d1ast__-d1bkca_.fasta

./group303/reference/d1ast__-d1buda_.fasta

./group303/reference/d1ast__-d1c7ka_.fasta

./group303/reference/d1ast__-d1dmta_.fasta

./group303/reference/d1ast__-d1eb6a_.fasta

./group303/reference/d1ast__-d1gkda_.fasta

./group303/reference/d1ast__-d1hs6a3.fasta

./group303/reference/d1ast__-d1i1ip_.fasta

./group303/reference/d1ast__-d1j36a_.fasta

./group303/reference/d1ast__-d1j7na1.fasta

./group303/reference/d1ast__-d1j7na2.fasta

./group303/reference/d1ast__-d1jk3a_.fasta

./group303/reference/d1ast__-d1k9xa_.fasta

./group303/reference/d1ast__-d1kufa_.fasta

./group303/reference/d1atla_-d1bqba_.fasta

./group303/reference/d1atla_-d1c7ka_.fasta

./group303/reference/d1atla_-d1eb6a_.fasta

./group303/reference/d1atla_-d1ezm__.fasta

./group303/reference/d1atla_-d1g12a_.fasta

./group303/reference/d1atla_-d1gkda_.fasta

./group303/reference/d1atla_-d1hs6a3.fasta

./group303/reference/d1atla_-d1j7na1.fasta

./group303/reference/d1atla_-d1j7na2.fasta

./group303/reference/d1atla_-d1jk3a_.fasta

./group303/reference/d1atla_-d1k7ia2.fasta

./group303/reference/d1atla_-d1k9xa_.fasta

./group303/reference/d1atla_-d1kapp2.fasta

./group303/reference/d1atla_-d1keia_.fasta

./group303/reference/d1bkca_-d1bqba_.fasta

./group303/reference/d1bkca_-d1c7ka_.fasta

./group303/reference/d1bkca_-d1dmta_.fasta

./group303/reference/d1bkca_-d1eb6a_.fasta

./group303/reference/d1bkca_-d1ezm__.fasta

./group303/reference/d1bkca_-d1g12a_.fasta

./group303/reference/d1bkca_-d1gkda_.fasta

./group303/reference/d1bkca_-d1hs6a3.fasta

./group303/reference/d1bkca_-d1j7na1.fasta

./group303/reference/d1bkca_-d1j7na2.fasta

./group303/reference/d1bkca_-d1jk3a_.fasta

./group303/reference/d1bkca_-d1k7ia2.fasta

./group303/reference/d1bkca_-d1k9xa_.fasta

./group303/reference/d1bkca_-d1kapp2.fasta

./group303/reference/d1bkca_-d1keia_.fasta

./group303/reference/d1bqba_-d1buda_.fasta

./group303/reference/d1bqba_-d1c7ka_.fasta

./group303/reference/d1bqba_-d1dmta_.fasta

./group303/reference/d1bqba_-d1eb6a_.fasta

./group303/reference/d1bqba_-d1g12a_.fasta

./group303/reference/d1bqba_-d1gkda_.fasta

./group303/reference/d1bqba_-d1hs6a3.fasta

./group303/reference/d1bqba_-d1i1ip_.fasta

./group303/reference/d1bqba_-d1j36a_.fasta

./group303/reference/d1bqba_-d1j7na1.fasta

./group303/reference/d1bqba_-d1jk3a_.fasta

./group303/reference/d1bqba_-d1k7ia2.fasta

./group303/reference/d1bqba_-d1kapp2.fasta

./group303/reference/d1buda_-d1c7ka_.fasta

./group303/reference/d1buda_-d1ezm__.fasta

./group303/reference/d1buda_-d1g12a_.fasta

./group303/reference/d1buda_-d1gkda_.fasta

./group303/reference/d1buda_-d1i1ip_.fasta

./group303/reference/d1buda_-d1j36a_.fasta

./group303/reference/d1buda_-d1j7na1.fasta

./group303/reference/d1buda_-d1j7na2.fasta

./group303/reference/d1buda_-d1jk3a_.fasta

./group303/reference/d1buda_-d1k7ia2.fasta

./group303/reference/d1buda_-d1kapp2.fasta

./group303/reference/d1buda_-d1keia_.fasta

./group303/reference/d1c7ka_-d1dmta_.fasta

./group303/reference/d1c7ka_-d1ezm__.fasta

./group303/reference/d1c7ka_-d1g12a_.fasta

./group303/reference/d1c7ka_-d1hs6a3.fasta

./group303/reference/d1c7ka_-d1i1ip_.fasta

./group303/reference/d1c7ka_-d1j7na1.fasta

./group303/reference/d1c7ka_-d1k7ia2.fasta

./group303/reference/d1c7ka_-d1k9xa_.fasta

./group303/reference/d1c7ka_-d1kufa_.fasta

./group303/reference/d1dmta_-d1eb6a_.fasta

./group303/reference/d1dmta_-d1ezm__.fasta

./group303/reference/d1dmta_-d1g12a_.fasta

./group303/reference/d1dmta_-d1gkda_.fasta

./group303/reference/d1dmta_-d1hs6a3.fasta

./group303/reference/d1dmta_-d1i1ip_.fasta

./group303/reference/d1dmta_-d1j7na1.fasta

./group303/reference/d1dmta_-d1j7na2.fasta

./group303/reference/d1dmta_-d1jk3a_.fasta

./group303/reference/d1dmta_-d1k7ia2.fasta

./group303/reference/d1dmta_-d1kapp2.fasta

./group303/reference/d1dmta_-d1keia_.fasta

./group303/reference/d1dmta_-d1kufa_.fasta

./group303/reference/d1eb6a_-d1ezm__.fasta

./group303/reference/d1eb6a_-d1gkda_.fasta

./group303/reference/d1eb6a_-d1j7na1.fasta

./group303/reference/d1eb6a_-d1j7na2.fasta

./group303/reference/d1eb6a_-d1jk3a_.fasta

./group303/reference/d1eb6a_-d1k7ia2.fasta

./group303/reference/d1eb6a_-d1kapp2.fasta

./group303/reference/d1eb6a_-d1keia_.fasta

./group303/reference/d1eb6a_-d1kufa_.fasta

./group303/reference/d1ezm__-d1g12a_.fasta

./group303/reference/d1ezm__-d1gkda_.fasta

./group303/reference/d1ezm__-d1i1ip_.fasta

./group303/reference/d1ezm__-d1j7na1.fasta

./group303/reference/d1ezm__-d1jk3a_.fasta

./group303/reference/d1ezm__-d1kufa_.fasta

./group303/reference/d1g12a_-d1gkda_.fasta

./group303/reference/d1g12a_-d1hs6a3.fasta

./group303/reference/d1g12a_-d1i1ip_.fasta

./group303/reference/d1g12a_-d1j36a_.fasta

./group303/reference/d1g12a_-d1j7na2.fasta

./group303/reference/d1g12a_-d1jk3a_.fasta

./group303/reference/d1g12a_-d1k7ia2.fasta

./group303/reference/d1g12a_-d1kapp2.fasta

./group303/reference/d1g12a_-d1keia_.fasta

./group303/reference/d1g12a_-d1kufa_.fasta

./group303/reference/d1gkda_-d1i1ip_.fasta

./group303/reference/d1gkda_-d1j36a_.fasta

./group303/reference/d1gkda_-d1j7na1.fasta

./group303/reference/d1gkda_-d1j7na2.fasta

./group303/reference/d1gkda_-d1k9xa_.fasta

./group303/reference/d1gkda_-d1keia_.fasta

./group303/reference/d1gkda_-d1kufa_.fasta

./group303/reference/d1hs6a3-d1i1ip_.fasta

./group303/reference/d1hs6a3-d1j7na2.fasta

./group303/reference/d1hs6a3-d1kapp2.fasta

./group303/reference/d1hs6a3-d1kufa_.fasta

./group303/reference/d1i1ip_-d1j36a_.fasta

./group303/reference/d1i1ip_-d1k7ia2.fasta

./group303/reference/d1i1ip_-d1k9xa_.fasta

./group303/reference/d1i1ip_-d1kapp2.fasta

./group303/reference/d1i1ip_-d1keia_.fasta

./group303/reference/d1j36a_-d1j7na1.fasta

./group303/reference/d1j36a_-d1j7na2.fasta

./group303/reference/d1j36a_-d1jk3a_.fasta

./group303/reference/d1j36a_-d1k9xa_.fasta

./group303/reference/d1j36a_-d1kapp2.fasta

./group303/reference/d1j36a_-d1keia_.fasta

./group303/reference/d1j7na1-d1j7na2.fasta

./group303/reference/d1j7na1-d1jk3a_.fasta

./group303/reference/d1j7na1-d1k7ia2.fasta

./group303/reference/d1j7na1-d1k9xa_.fasta

./group303/reference/d1j7na1-d1kufa_.fasta

./group303/reference/d1j7na2-d1jk3a_.fasta

./group303/reference/d1j7na2-d1k7ia2.fasta

./group303/reference/d1j7na2-d1k9xa_.fasta

./group303/reference/d1j7na2-d1keia_.fasta

./group303/reference/d1j7na2-d1kufa_.fasta

./group303/reference/d1jk3a_-d1k9xa_.fasta

./group303/reference/d1jk3a_-d1keia_.fasta

./group303/reference/d1jk3a_-d1kufa_.fasta

./group303/reference/d1k7ia2-d1k9xa_.fasta

./group303/reference/d1k7ia2-d1keia_.fasta

./group303/reference/d1k7ia2-d1kufa_.fasta

./group303/reference/d1k9xa_-d1kapp2.fasta

./group303/reference/d1k9xa_-d1keia_.fasta

./group303/reference/d1k9xa_-d1kufa_.fasta

./group303/reference/d1kapp2-d1keia_.fasta

./group303/reference/d1kapp2-d1kufa_.fasta

./group303/reference/d1keia_-d1kufa_.fasta

./group305/reference/d1a09a_-d2cbla3.fasta

./group305/reference/d1a81a1-d2cbla3.fasta

./group305/reference/d1a81a2-d2cbla3.fasta

./group305/reference/d1ayaa_-d2cbla3.fasta

./group305/reference/d1d4ta_-d2cbla3.fasta

./group305/reference/d1fu6a_-d2cbla3.fasta

./group305/reference/d1jwoa_-d2cbla3.fasta

./group305/reference/d1jyra_-d2cbla3.fasta

./group305/reference/d1lkka_-d2cbla3.fasta

./group305/reference/d1luia_-d2cbla3.fasta

./group305/reference/d1m61a_-d2cbla3.fasta

./group305/reference/d1mil__-d2cbla3.fasta

./group305/reference/d1opka2-d2cbla3.fasta

./group305/reference/d2cbla3-d2plda_.fasta

./group307/reference/d1dq3a3-d1dq3a4.fasta

./group307/reference/d1dq3a3-d1g9za_.fasta

./group307/reference/d1dq3a3-d1m5xa_.fasta

./group307/reference/d1dq3a4-d1g9za_.fasta

./group307/reference/d1dq3a4-d1m5xa_.fasta

./group308/reference/d1a8ra_-d1b66a_.fasta

./group308/reference/d1a8ra_-d1b9la_.fasta

./group308/reference/d1a8ra_-d1dhn__.fasta

./group308/reference/d1b66a_-d1b9la_.fasta

./group308/reference/d1b66a_-d1dhn__.fasta

./group308/reference/d1b66a_-d1uox_1.fasta

./group308/reference/d1b9la_-d1uox_1.fasta

./group308/reference/d1dhn__-d1uox_1.fasta

./group309/reference/d12asa_-d1eova2.fasta

./group309/reference/d12asa_-d1jjca_.fasta

./group309/reference/d12asa_-d1jjcb5.fasta

./group309/reference/d12asa_-d1qf6a4.fasta

./group309/reference/d12asa_-d1seta2.fasta

./group309/reference/d1b8aa2-d1jjca_.fasta

./group309/reference/d1b8aa2-d1jjcb5.fasta

./group309/reference/d1b8aa2-d1nj1a3.fasta

./group309/reference/d1b8aa2-d1qf6a4.fasta

./group309/reference/d1b8aa2-d1seta2.fasta

./group309/reference/d1eova2-d1jjca_.fasta

./group309/reference/d1eova2-d1nj1a3.fasta

./group309/reference/d1eova2-d1qf6a4.fasta

./group309/reference/d1eova2-d1seta2.fasta

./group309/reference/d1jjca_-d1nj1a3.fasta

./group309/reference/d1jjca_-d1qf6a4.fasta

./group309/reference/d1jjca_-d1seta2.fasta

./group309/reference/d1jjcb5-d1nj1a3.fasta

./group309/reference/d1jjcb5-d1qf6a4.fasta

./group309/reference/d1jjcb5-d1seta2.fasta

./group309/reference/d1nj1a3-d1qf6a4.fasta

./group309/reference/d1nj1a3-d1seta2.fasta

./group309/reference/d1qf6a4-d1seta2.fasta

./group310/reference/d1cjwa_-d1iica2.fasta

./group310/reference/d1cjwa_-d1iyka1.fasta

./group310/reference/d1cjwa_-d1iyka2.fasta

./group310/reference/d1cjwa_-d1lrza2.fasta

./group310/reference/d1cjwa_-d1m4ia_.fasta

./group310/reference/d1cjwa_-d1mk4a_.fasta

./group310/reference/d1cjwa_-d1n71a_.fasta

./group310/reference/d1cjwa_-d1qsma_.fasta

./group310/reference/d1cjwa_-d1qsta_.fasta

./group310/reference/d1cjwa_-d1ufha_.fasta

./group310/reference/d1fy7a_-d1iica2.fasta

./group310/reference/d1fy7a_-d1iyka1.fasta

./group310/reference/d1fy7a_-d1iyka2.fasta

./group310/reference/d1fy7a_-d1n71a_.fasta

./group310/reference/d1fy7a_-d1qsma_.fasta

./group310/reference/d1fy7a_-d1qsta_.fasta

./group310/reference/d1fy7a_-d1ufha_.fasta

./group310/reference/d1iica2-d1iyka1.fasta

./group310/reference/d1iica2-d1m4ia_.fasta

./group310/reference/d1iica2-d1mk4a_.fasta

./group310/reference/d1iica2-d1n71a_.fasta

./group310/reference/d1iica2-d1qsma_.fasta

./group310/reference/d1iica2-d1qsta_.fasta

./group310/reference/d1iyka1-d1lrza2.fasta

./group310/reference/d1iyka1-d1mk4a_.fasta

./group310/reference/d1iyka1-d1n71a_.fasta

./group310/reference/d1iyka1-d1qsma_.fasta

./group310/reference/d1iyka1-d1qsta_.fasta

./group310/reference/d1iyka2-d1mk4a_.fasta

./group310/reference/d1iyka2-d1n71a_.fasta

./group310/reference/d1iyka2-d1qsma_.fasta

./group310/reference/d1iyka2-d1qsta_.fasta

./group310/reference/d1lrza2-d1mk4a_.fasta

./group310/reference/d1lrza2-d1n71a_.fasta

./group310/reference/d1lrza2-d1qsta_.fasta

./group310/reference/d1lrza2-d1ufha_.fasta

./group310/reference/d1m4ia_-d1mk4a_.fasta

./group310/reference/d1m4ia_-d1qsma_.fasta

./group310/reference/d1m4ia_-d1qsta_.fasta

./group310/reference/d1m4ia_-d1ufha_.fasta

./group310/reference/d1mk4a_-d1n71a_.fasta

./group310/reference/d1mk4a_-d1qsma_.fasta

./group310/reference/d1mk4a_-d1qsta_.fasta

./group310/reference/d1mk4a_-d1ufha_.fasta

./group310/reference/d1n71a_-d1qsma_.fasta

./group310/reference/d1n71a_-d1qsta_.fasta

./group310/reference/d1n71a_-d1ufha_.fasta

./group310/reference/d1qsma_-d1qsta_.fasta

./group310/reference/d1qsma_-d1ufha_.fasta

./group310/reference/d1qsta_-d1ufha_.fasta

./group311/reference/d1ak7__-d1d0na3.fasta

./group311/reference/d1ak7__-d1d0na5.fasta

./group311/reference/d1ak7__-d1jhwa3.fasta

./group311/reference/d1cfya_-d1d0na4.fasta

./group311/reference/d1cfya_-d1d0na5.fasta

./group311/reference/d1cfya_-d1d0na6.fasta

./group311/reference/d1cfya_-d1hqz1_.fasta

./group311/reference/d1cfya_-d1jhwa3.fasta

./group311/reference/d1d0na3-d1d0na4.fasta

./group311/reference/d1d0na3-d1d4xg_.fasta

./group311/reference/d1d0na3-d1f7sa_.fasta

./group311/reference/d1d0na3-d1m4ja_.fasta

./group311/reference/d1d0na4-d1d0na6.fasta

./group311/reference/d1d0na4-d1f7sa_.fasta

./group311/reference/d1d0na4-d1hqz1_.fasta

./group311/reference/d1d0na4-d1jhwa3.fasta

./group311/reference/d1d0na4-d1m4ja_.fasta

./group311/reference/d1d0na5-d1d4xg_.fasta

./group311/reference/d1d0na5-d1f7sa_.fasta

./group311/reference/d1d4xg_-d1f7sa_.fasta

./group311/reference/d1d4xg_-d1hqz1_.fasta

./group311/reference/d1d4xg_-d1jhwa3.fasta

./group311/reference/d1f7sa_-d1hqz1_.fasta

./group311/reference/d1hqz1_-d1jhwa3.fasta

./group311/reference/d1hqz1_-d1m4ja_.fasta

./group311/reference/d1jhwa3-d1m4ja_.fasta

./group313/reference/d1f5ma_-d1mc0a1.fasta

./group313/reference/d1f5ma_-d1mc0a2.fasta

./group313/reference/d1f5ma_-d1mkma2.fasta

./group313/reference/d1mc0a1-d1mkma2.fasta

./group314/reference/d1bywa_-d1ew0a_.fasta

./group314/reference/d1bywa_-d1ll8a_.fasta

./group314/reference/d1bywa_-d1lswa_.fasta

./group314/reference/d1bywa_-d3pyp__.fasta

./group314/reference/d1ew0a_-d1ll8a_.fasta

./group314/reference/d1ew0a_-d3pyp__.fasta

./group314/reference/d1jnua_-d1ll8a_.fasta

./group314/reference/d1jnua_-d1lswa_.fasta

./group314/reference/d1jnua_-d3pyp__.fasta

./group314/reference/d1ll8a_-d1n9la_.fasta

./group314/reference/d1ll8a_-d3pyp__.fasta

./group314/reference/d1lswa_-d3pyp__.fasta

./group314/reference/d1n9la_-d3pyp__.fasta

./group315/reference/d1a3aa_-d1hynp_.fasta

./group315/reference/d1a6ja_-d1hynp_.fasta

./group316/reference/d1hzta_-d1jkna_.fasta

./group316/reference/d1hzta_-d1k2ea_.fasta

./group316/reference/d1jkna_-d1k2ea_.fasta

./group316/reference/d1k2ea_-d1ktga_.fasta

./group317/reference/d1b5ea_-d1f28a_.fasta

./group319/reference/d1b63a2-d1bxda_.fasta

./group319/reference/d1b63a2-d1ei1a2.fasta

./group319/reference/d1b63a2-d1i58a_.fasta

./group319/reference/d1b63a2-d1id0a_.fasta

./group319/reference/d1b63a2-d1kija2.fasta

./group319/reference/d1bxda_-d1byqa_.fasta

./group319/reference/d1bxda_-d1kija2.fasta

./group319/reference/d1byqa_-d1ei1a2.fasta

./group319/reference/d1byqa_-d1id0a_.fasta

./group319/reference/d1byqa_-d1kija2.fasta

./group321/reference/d1bwda_-d1g61a_.fasta

./group321/reference/d1bwda_-d1g62a_.fasta

./group321/reference/d1bwda_-d1h70a_.fasta

./group321/reference/d1h70a_-d1jdw__.fasta

./group324/reference/d1aisa2-d1mpga2.fasta

./group324/reference/d1ko9a2-d1mpga2.fasta

./group324/reference/d1ko9a2-d1ytba1.fasta

./group324/reference/d1mpga2-d1ytba1.fasta

./group325/reference/d1k2yx4-d1kfia4.fasta

./group325/reference/d1k2yx4-d3pmga4.fasta

./group326/reference/d1fm4a_-d1kcma_.fasta

./group326/reference/d1icxa_-d1kcma_.fasta

./group326/reference/d1jssa_-d1ln1a_.fasta

./group327/reference/d1mxa_2-d1mxa_3.fasta

./group327/reference/d1mxa_3-d1qm4a2.fasta

./group328/reference/d1b77a1-d1dmla1.fasta

./group328/reference/d1b77a1-d1iz5a1.fasta

./group328/reference/d1b77a1-d1iz5a2.fasta

./group328/reference/d1b77a1-d1plq_1.fasta

./group328/reference/d1b77a1-d1plq_2.fasta

./group328/reference/d1b77a1-d2pola2.fasta

./group328/reference/d1b77a1-d2pola3.fasta

./group328/reference/d1b77a2-d1iz5a1.fasta

./group328/reference/d1b77a2-d1plq_2.fasta

./group328/reference/d1b77a2-d2pola1.fasta

./group328/reference/d1b77a2-d2pola3.fasta

./group328/reference/d1dmla1-d1iz5a1.fasta

./group328/reference/d1dmla1-d1plq_1.fasta

./group328/reference/d1dmla1-d2pola1.fasta

./group328/reference/d1dmla1-d2pola3.fasta

./group328/reference/d1iz5a1-d1iz5a2.fasta

./group328/reference/d1iz5a1-d1plq_2.fasta

./group328/reference/d1iz5a1-d2pola1.fasta

./group328/reference/d1iz5a1-d2pola2.fasta

./group328/reference/d1iz5a1-d2pola3.fasta

./group328/reference/d1iz5a2-d1plq_1.fasta

./group328/reference/d1iz5a2-d2pola1.fasta

./group328/reference/d1plq_1-d1plq_2.fasta

./group328/reference/d1plq_1-d2pola2.fasta

./group328/reference/d1plq_1-d2pola3.fasta

./group328/reference/d1plq_2-d2pola1.fasta

./group328/reference/d1plq_2-d2pola3.fasta

./group328/reference/d2pola1-d2pola2.fasta

./group330/reference/d1ckv__-d1g10a_.fasta

./group330/reference/d1ckv__-d1hqi__.fasta

./group330/reference/d1g10a_-d1hqi__.fasta

./group331/reference/d1jj2e1-d1jj2e2.fasta

./group331/reference/d1jj2e1-d1rl6a2.fasta

./group331/reference/d1rl6a1-d1rl6a2.fasta

./group332/reference/d1a9xa5-d1e4ea2.fasta

./group332/reference/d1a9xa5-d1ehia2.fasta

./group332/reference/d1a9xa5-d1eucb2.fasta

./group332/reference/d1a9xa5-d1i7na2.fasta

./group332/reference/d1a9xa5-d1iow_2.fasta

./group332/reference/d1a9xa5-d1jkjb2.fasta

./group332/reference/d1a9xa6-d1e4ea2.fasta

./group332/reference/d1a9xa6-d1ehia2.fasta

./group332/reference/d1a9xa6-d1eucb2.fasta

./group332/reference/d1a9xa6-d1i7na2.fasta

./group332/reference/d1a9xa6-d1jkjb2.fasta

./group332/reference/d1a9xa6-d1kbla3.fasta

./group332/reference/d1e4ea2-d1eucb2.fasta

./group332/reference/d1e4ea2-d1gsa_2.fasta

./group332/reference/d1e4ea2-d1i7na2.fasta

./group332/reference/d1e4ea2-d1jkjb2.fasta

./group332/reference/d1ehia2-d1eucb2.fasta

./group332/reference/d1ehia2-d1gsa_2.fasta

./group332/reference/d1ehia2-d1i7na2.fasta

./group332/reference/d1ehia2-d1jkjb2.fasta

./group332/reference/d1ehia2-d1kbla3.fasta

./group332/reference/d1eucb2-d1gsa_2.fasta

./group332/reference/d1eucb2-d1iow_2.fasta

./group332/reference/d1eucb2-d1kbla3.fasta

./group332/reference/d1gsa_2-d1i7na2.fasta

./group332/reference/d1gsa_2-d1iow_2.fasta

./group332/reference/d1gsa_2-d1jkjb2.fasta

./group332/reference/d1i7na2-d1iow_2.fasta

./group332/reference/d1i7na2-d1jkjb2.fasta

./group332/reference/d1i7na2-d1kbla3.fasta

./group332/reference/d1iow_2-d1jkjb2.fasta

./group332/reference/d1iow_2-d1kbla3.fasta

./group332/reference/d1jkjb2-d1kbla3.fasta

./group333/reference/d1apme_-d1j7la_.fasta

./group333/reference/d1b6cb_-d1j7la_.fasta

./group333/reference/d1b6cb_-d1tkia_.fasta

./group333/reference/d1blxa_-d1j7la_.fasta

./group333/reference/d1csn__-d1j7la_.fasta

./group333/reference/d1csn__-d1k2pa_.fasta

./group333/reference/d1csn__-d1koba_.fasta

./group333/reference/d1csn__-d1m2ra_.fasta

./group333/reference/d1csn__-d1opja_.fasta

./group333/reference/d1csn__-d1p38__.fasta

./group333/reference/d1csn__-d1p4oa_.fasta

./group333/reference/d1csn__-d1phk__.fasta

./group333/reference/d1csn__-d1tkia_.fasta

./group333/reference/d1j7la_-d1k2pa_.fasta

./group333/reference/d1j7la_-d1koba_.fasta

./group333/reference/d1j7la_-d1m2ra_.fasta

./group333/reference/d1j7la_-d1opja_.fasta

./group333/reference/d1j7la_-d1p38__.fasta

./group333/reference/d1j7la_-d1p4oa_.fasta

./group333/reference/d1j7la_-d1phk__.fasta

./group333/reference/d1j7la_-d1tkia_.fasta

./group333/reference/d1k2pa_-d1m2ra_.fasta

./group333/reference/d1k2pa_-d1tkia_.fasta

./group333/reference/d1m2ra_-d1opja_.fasta

./group333/reference/d1opja_-d1tkia_.fasta

./group333/reference/d1p4oa_-d1tkia_.fasta

./group334/reference/d1diqa2-d1f0xa2.fasta

./group334/reference/d1diqa2-d1hska1.fasta

./group334/reference/d1diqa2-d1i19a2.fasta

./group334/reference/d1diqa2-d1jroa4.fasta

./group334/reference/d1diqa2-d1uxy_1.fasta

./group334/reference/d1f0xa2-d1fo4a6.fasta

./group334/reference/d1f0xa2-d1hska1.fasta

./group334/reference/d1f0xa2-d1i19a2.fasta

./group334/reference/d1f0xa2-d1jroa4.fasta

./group334/reference/d1f0xa2-d1n62c2.fasta

./group334/reference/d1f0xa2-d1uxy_1.fasta

./group334/reference/d1fo4a6-d1hska1.fasta

./group334/reference/d1fo4a6-d1i19a2.fasta

./group334/reference/d1fo4a6-d1n62c2.fasta

./group334/reference/d1fo4a6-d1uxy_1.fasta

./group334/reference/d1hska1-d1i19a2.fasta

./group334/reference/d1hska1-d1jroa4.fasta

./group334/reference/d1hska1-d1n62c2.fasta

./group334/reference/d1i19a2-d1jroa4.fasta

./group334/reference/d1i19a2-d1uxy_1.fasta

./group334/reference/d1jroa4-d1uxy_1.fasta

./group334/reference/d1n62c2-d1uxy_1.fasta

./group335/reference/d1f7la_-d1qr0a1.fasta

./group335/reference/d1ftha_-d1qr0a1.fasta

./group336/reference/d1ct9a2-d1iru1_.fasta

./group336/reference/d1ct9a2-d1iru2_.fasta

./group336/reference/d1ct9a2-d1iruk_.fasta

./group336/reference/d1ct9a2-d1j2pa_.fasta

./group336/reference/d1ct9a2-d1j2qh_.fasta

./group336/reference/d1ct9a2-d1ryp1_.fasta

./group336/reference/d1ct9a2-d1ryp2_.fasta

./group336/reference/d1ct9a2-d1rypc_.fasta

./group336/reference/d1ct9a2-d1rypd_.fasta

./group336/reference/d1ct9a2-d1rypf_.fasta

./group336/reference/d1ct9a2-d1ryph_.fasta

./group336/reference/d1ct9a2-d1rypi_.fasta

./group336/reference/d1ct9a2-d1rypk_.fasta

./group336/reference/d1ecfa2-d1iru1_.fasta

./group336/reference/d1ecfa2-d1iru2_.fasta

./group336/reference/d1ecfa2-d1iruk_.fasta

./group336/reference/d1ecfa2-d1j2pa_.fasta

./group336/reference/d1ecfa2-d1j2qh_.fasta

./group336/reference/d1ecfa2-d1ryp1_.fasta

./group336/reference/d1ecfa2-d1rypc_.fasta

./group336/reference/d1ecfa2-d1rypd_.fasta

./group336/reference/d1ecfa2-d1rype_.fasta

./group336/reference/d1ecfa2-d1rypf_.fasta

./group336/reference/d1ecfa2-d1rypg_.fasta

./group336/reference/d1ecfa2-d1rypi_.fasta

./group336/reference/d1ecfa2-d1rypj_.fasta

./group336/reference/d1ecfa2-d1rypl_.fasta

./group336/reference/d1gdoa_-d1iru2_.fasta

./group336/reference/d1gdoa_-d1iruk_.fasta

./group336/reference/d1gdoa_-d1j2pa_.fasta

./group336/reference/d1gdoa_-d1j2qh_.fasta

./group336/reference/d1gdoa_-d1ofda3.fasta

./group336/reference/d1gdoa_-d1pmaa_.fasta

./group336/reference/d1gdoa_-d1ryp1_.fasta

./group336/reference/d1gdoa_-d1ryp2_.fasta

./group336/reference/d1gdoa_-d1rypa_.fasta

./group336/reference/d1gdoa_-d1rypb_.fasta

./group336/reference/d1gdoa_-d1rypc_.fasta

./group336/reference/d1gdoa_-d1rypd_.fasta

./group336/reference/d1gdoa_-d1rype_.fasta

./group336/reference/d1gdoa_-d1rypf_.fasta

./group336/reference/d1gdoa_-d1rypg_.fasta

./group336/reference/d1gdoa_-d1ryph_.fasta

./group336/reference/d1gdoa_-d1rypi_.fasta

./group336/reference/d1gdoa_-d1rypj_.fasta

./group336/reference/d1gdoa_-d1rypk_.fasta

./group336/reference/d1gdoa_-d1rypl_.fasta

./group336/reference/d1gph12-d1iru2_.fasta

./group336/reference/d1gph12-d1iruk_.fasta

./group336/reference/d1gph12-d1j2pa_.fasta

./group336/reference/d1gph12-d1j2qh_.fasta

./group336/reference/d1gph12-d1pmaa_.fasta

./group336/reference/d1gph12-d1ryp1_.fasta

./group336/reference/d1gph12-d1rypa_.fasta

./group336/reference/d1gph12-d1rypb_.fasta

./group336/reference/d1gph12-d1rypc_.fasta

./group336/reference/d1gph12-d1rypd_.fasta

./group336/reference/d1gph12-d1rype_.fasta

./group336/reference/d1gph12-d1rypf_.fasta

./group336/reference/d1gph12-d1rypg_.fasta

./group336/reference/d1gph12-d1rypi_.fasta

./group336/reference/d1gph12-d1rypj_.fasta

./group336/reference/d1gph12-d1rypk_.fasta

./group336/reference/d1gph12-d1rypl_.fasta

./group336/reference/d1iru1_-d1ofda3.fasta

./group336/reference/d1iru1_-d1pmaa_.fasta

./group336/reference/d1iru1_-d1ryp2_.fasta

./group336/reference/d1iru1_-d1rypa_.fasta

./group336/reference/d1iru1_-d1rypd_.fasta

./group336/reference/d1iru1_-d1rypf_.fasta

./group336/reference/d1iru1_-d1rypi_.fasta

./group336/reference/d1iru2_-d1iruk_.fasta

./group336/reference/d1iru2_-d1j2pa_.fasta

./group336/reference/d1iru2_-d1ofda3.fasta

./group336/reference/d1iru2_-d1rypa_.fasta

./group336/reference/d1iru2_-d1rypb_.fasta

./group336/reference/d1iru2_-d1rypc_.fasta

./group336/reference/d1iru2_-d1rypd_.fasta

./group336/reference/d1iru2_-d1rype_.fasta

./group336/reference/d1iru2_-d1rypf_.fasta

./group336/reference/d1iru2_-d1rypg_.fasta

./group336/reference/d1iru2_-d1rypj_.fasta

./group336/reference/d1iru2_-d1rypk_.fasta

./group336/reference/d1iruk_-d1ofda3.fasta

./group336/reference/d1iruk_-d1ryp2_.fasta

./group336/reference/d1iruk_-d1rypa_.fasta

./group336/reference/d1iruk_-d1rypc_.fasta

./group336/reference/d1iruk_-d1rypd_.fasta

./group336/reference/d1iruk_-d1rype_.fasta

./group336/reference/d1iruk_-d1rypf_.fasta

./group336/reference/d1iruk_-d1rypg_.fasta

./group336/reference/d1iruk_-d1ryph_.fasta

./group336/reference/d1iruk_-d1rypi_.fasta

./group336/reference/d1iruk_-d1rypl_.fasta

./group336/reference/d1j2pa_-d1ryp2_.fasta

./group336/reference/d1j2pa_-d1ryph_.fasta

./group336/reference/d1j2pa_-d1rypk_.fasta

./group336/reference/d1j2pa_-d1rypl_.fasta

./group336/reference/d1j2qh_-d1ryp2_.fasta

./group336/reference/d1ofda3-d1ryp1_.fasta

./group336/reference/d1ofda3-d1ryp2_.fasta

./group336/reference/d1ofda3-d1rypj_.fasta

./group336/reference/d1ofda3-d1rypl_.fasta

./group336/reference/d1pmaa_-d1ryp2_.fasta

./group336/reference/d1pmaa_-d1ryph_.fasta

./group336/reference/d1pmaa_-d1rypk_.fasta

./group336/reference/d1pmaa_-d1rypl_.fasta

./group336/reference/d1ryp1_-d1ryp2_.fasta

./group336/reference/d1ryp1_-d1rypi_.fasta

./group336/reference/d1ryp1_-d1rypj_.fasta

./group336/reference/d1ryp1_-d1rypl_.fasta

./group336/reference/d1ryp2_-d1rypa_.fasta

./group336/reference/d1ryp2_-d1rypb_.fasta

./group336/reference/d1ryp2_-d1rypc_.fasta

./group336/reference/d1ryp2_-d1rypd_.fasta

./group336/reference/d1ryp2_-d1rype_.fasta

./group336/reference/d1ryp2_-d1rypf_.fasta

./group336/reference/d1ryp2_-d1rypg_.fasta

./group336/reference/d1ryp2_-d1ryph_.fasta

./group336/reference/d1ryp2_-d1rypk_.fasta

./group336/reference/d1ryp2_-d1rypl_.fasta

./group336/reference/d1rypa_-d1ryph_.fasta

./group336/reference/d1rypa_-d1rypi_.fasta

./group336/reference/d1rypa_-d1rypj_.fasta

./group336/reference/d1rypa_-d1rypk_.fasta

./group336/reference/d1rypa_-d1rypl_.fasta

./group336/reference/d1rypb_-d1ryph_.fasta

./group336/reference/d1rypb_-d1rypj_.fasta

./group336/reference/d1rypb_-d1rypl_.fasta

./group336/reference/d1rypc_-d1ryph_.fasta

./group336/reference/d1rypc_-d1rypj_.fasta

./group336/reference/d1rypc_-d1rypk_.fasta

./group336/reference/d1rypc_-d1rypl_.fasta

./group336/reference/d1rypd_-d1ryph_.fasta

./group336/reference/d1rypd_-d1rypl_.fasta

./group336/reference/d1rype_-d1ryph_.fasta

./group336/reference/d1rype_-d1rypj_.fasta

./group336/reference/d1rype_-d1rypk_.fasta

./group336/reference/d1rype_-d1rypl_.fasta

./group336/reference/d1rypf_-d1ryph_.fasta

./group336/reference/d1rypf_-d1rypl_.fasta

./group336/reference/d1rypg_-d1ryph_.fasta

./group336/reference/d1rypg_-d1rypj_.fasta

./group336/reference/d1rypg_-d1rypk_.fasta

./group336/reference/d1rypg_-d1rypl_.fasta

./group336/reference/d1rypi_-d1rypj_.fasta

./group337/reference/d1a7ta_-d1e5da2.fasta

./group337/reference/d1a7ta_-d1k07a_.fasta

./group337/reference/d1a7ta_-d1smla_.fasta

./group337/reference/d1e5da2-d1jjea_.fasta

./group337/reference/d1e5da2-d1k07a_.fasta

./group337/reference/d1e5da2-d1m2xa_.fasta

./group337/reference/d1e5da2-d1qh5a_.fasta

./group337/reference/d1e5da2-d1smla_.fasta

./group337/reference/d1jjea_-d1k07a_.fasta

./group337/reference/d1jjea_-d1qh5a_.fasta

./group337/reference/d1jjea_-d1smla_.fasta

./group337/reference/d1k07a_-d1m2xa_.fasta

./group337/reference/d1k07a_-d1qh5a_.fasta

./group337/reference/d1m2xa_-d1qh5a_.fasta

./group337/reference/d1m2xa_-d1smla_.fasta

./group337/reference/d1qh5a_-d1smla_.fasta

./group338/reference/d1g5ba_-d1ii7a_.fasta

./group338/reference/d1g5ba_-d1nnwa_.fasta

./group338/reference/d1g5ba_-d1utea_.fasta

./group338/reference/d1g5ba_-d4kbpa2.fasta

./group338/reference/d1ii7a_-d1jk7a_.fasta

./group338/reference/d1ii7a_-d4kbpa2.fasta

./group338/reference/d1jk7a_-d1nnwa_.fasta

./group338/reference/d1jk7a_-d1utea_.fasta

./group338/reference/d1jk7a_-d4kbpa2.fasta

./group338/reference/d1nnwa_-d1utea_.fasta

./group338/reference/d1utea_-d4kbpa2.fasta

./group339/reference/d1a5z_2-d1b8pa2.fasta

./group339/reference/d1b8pa2-d1ceqa2.fasta

./group339/reference/d1b8pa2-d1ez4a2.fasta

./group339/reference/d1b8pa2-d1guya2.fasta

./group339/reference/d1b8pa2-d1i0za2.fasta

./group339/reference/d1b8pa2-d1ldna2.fasta

./group339/reference/d1b8pa2-d1llda2.fasta

./group339/reference/d1b8pa2-d1o6za2.fasta

./group339/reference/d1ceqa2-d7mdha2.fasta

./group339/reference/d1guya2-d5mdha2.fasta

./group339/reference/d1i0za2-d5mdha2.fasta

./group339/reference/d1ldna2-d5mdha2.fasta

./group339/reference/d1llda2-d5mdha2.fasta

./group339/reference/d2cmd_2-d5mdha2.fasta

./group341/reference/d1a26_2-d1f0la2.fasta

./group341/reference/d1a26_2-d1giqa2.fasta

./group341/reference/d1a26_2-d1ikpa2.fasta

./group341/reference/d1a26_2-d1qs1a1.fasta

./group341/reference/d1f0la2-d1giqa1.fasta

./group341/reference/d1f0la2-d1giqa2.fasta

./group341/reference/d1f0la2-d1qs1a1.fasta

./group341/reference/d1f0la2-d1qs1a2.fasta

./group341/reference/d1giqa1-d1giqa2.fasta

./group341/reference/d1giqa1-d1gxya_.fasta

./group341/reference/d1giqa1-d1ikpa2.fasta

./group341/reference/d1giqa2-d1gxya_.fasta

./group341/reference/d1giqa2-d1ikpa2.fasta

./group341/reference/d1gxya_-d1ikpa2.fasta

./group341/reference/d1gxya_-d1qs1a2.fasta

./group341/reference/d1ikpa2-d1qs1a1.fasta

./group341/reference/d1ikpa2-d1qs1a2.fasta

./group342/reference/d1chua3-d1jnra3.fasta

./group342/reference/d1jnra3-d1kf6a3.fasta

./group342/reference/d1jnra3-d1kssa3.fasta

./group342/reference/d1jnra3-d1neka3.fasta

./group342/reference/d1jnra3-d1qlaa3.fasta

./group342/reference/d1jnra3-d1qo8a3.fasta

./group343/reference/d1b08a1-d1b6e__.fasta

./group343/reference/d1b08a1-d1e87a_.fasta

./group343/reference/d1b08a1-d1f00i3.fasta

./group343/reference/d1b08a1-d1hq8a_.fasta

./group343/reference/d1b08a1-d1prea1.fasta

./group343/reference/d1b08a1-d1prtb2.fasta

./group343/reference/d1b6e__-d1cwva5.fasta

./group343/reference/d1b6e__-d1f00i3.fasta

./group343/reference/d1b6e__-d1h8ua_.fasta

./group343/reference/d1b6e__-d1kg0c_.fasta

./group343/reference/d1b6e__-d1koe__.fasta

./group343/reference/d1b6e__-d1prea1.fasta

./group343/reference/d1b6e__-d1prtb2.fasta

./group343/reference/d1byfa_-d1cwva5.fasta

./group343/reference/d1byfa_-d1e87a_.fasta

./group343/reference/d1byfa_-d1f00i3.fasta

./group343/reference/d1byfa_-d1h8ua_.fasta

./group343/reference/d1byfa_-d1hq8a_.fasta

./group343/reference/d1byfa_-d1j34a_.fasta

./group343/reference/d1byfa_-d1jwib_.fasta

./group343/reference/d1byfa_-d1jzna_.fasta

./group343/reference/d1byfa_-d1kg0c_.fasta

./group343/reference/d1byfa_-d1tn3__.fasta

./group343/reference/d1byfa_-d1tsg__.fasta

./group343/reference/d1cwva5-d1dv8a_.fasta

./group343/reference/d1cwva5-d1e87a_.fasta

./group343/reference/d1cwva5-d1f00i3.fasta

./group343/reference/d1cwva5-d1g1ta1.fasta

./group343/reference/d1cwva5-d1h8ua_.fasta

./group343/reference/d1cwva5-d1hq8a_.fasta

./group343/reference/d1cwva5-d1j34a_.fasta

./group343/reference/d1cwva5-d1jzna_.fasta

./group343/reference/d1cwva5-d1kg0c_.fasta

./group343/reference/d1cwva5-d1prea1.fasta

./group343/reference/d1cwva5-d1qdda_.fasta

./group343/reference/d1cwva5-d1tn3__.fasta

./group343/reference/d1cwva5-d1tsg__.fasta

./group343/reference/d1dv8a_-d1f00i3.fasta

./group343/reference/d1dv8a_-d1kg0c_.fasta

./group343/reference/d1dv8a_-d1prtb2.fasta

./group343/reference/d1dv8a_-d1tsg__.fasta

./group343/reference/d1e87a_-d1f00i3.fasta

./group343/reference/d1e87a_-d1h8ua_.fasta

./group343/reference/d1e87a_-d1koe__.fasta

./group343/reference/d1f00i3-d1g1ta1.fasta

./group343/reference/d1f00i3-d1h8ua_.fasta

./group343/reference/d1f00i3-d1hq8a_.fasta

./group343/reference/d1f00i3-d1j34a_.fasta

./group343/reference/d1f00i3-d1j34b_.fasta

./group343/reference/d1f00i3-d1jwib_.fasta

./group343/reference/d1f00i3-d1jzna_.fasta

./group343/reference/d1f00i3-d1k9ja_.fasta

./group343/reference/d1f00i3-d1kg0c_.fasta

./group343/reference/d1f00i3-d1koe__.fasta

./group343/reference/d1f00i3-d1tn3__.fasta

./group343/reference/d1f00i3-d1tsg__.fasta

./group343/reference/d1g1ta1-d1kg0c_.fasta

./group343/reference/d1g1ta1-d1prea1.fasta

./group343/reference/d1g1ta1-d1prtb2.fasta

./group343/reference/d1h8ua_-d1hq8a_.fasta

./group343/reference/d1h8ua_-d1kg0c_.fasta

./group343/reference/d1h8ua_-d1koe__.fasta

./group343/reference/d1h8ua_-d1tsg__.fasta

./group343/reference/d1hq8a_-d1kg0c_.fasta

./group343/reference/d1hq8a_-d1koe__.fasta

./group343/reference/d1hq8a_-d1prtb2.fasta

./group343/reference/d1hq8a_-d1tsg__.fasta

./group343/reference/d1j34a_-d1kg0c_.fasta

./group343/reference/d1j34a_-d1koe__.fasta

./group343/reference/d1j34a_-d1prea1.fasta

./group343/reference/d1j34a_-d1tsg__.fasta

./group343/reference/d1j34b_-d1kg0c_.fasta

./group343/reference/d1j34b_-d1koe__.fasta

./group343/reference/d1j34b_-d1prea1.fasta

./group343/reference/d1j34b_-d1prtb2.fasta

./group343/reference/d1j34b_-d1tsg__.fasta

./group343/reference/d1jwib_-d1kg0c_.fasta

./group343/reference/d1jwib_-d1koe__.fasta

./group343/reference/d1jwib_-d1tsg__.fasta

./group343/reference/d1jzna_-d1kg0c_.fasta

./group343/reference/d1jzna_-d1koe__.fasta

./group343/reference/d1jzna_-d1prtb2.fasta

./group343/reference/d1jzna_-d1tsg__.fasta

./group343/reference/d1k9ja_-d1kg0c_.fasta

./group343/reference/d1k9ja_-d1prea1.fasta

./group343/reference/d1k9ja_-d1prtb2.fasta

./group343/reference/d1kg0c_-d1koe__.fasta

./group343/reference/d1kg0c_-d1prtb2.fasta

./group343/reference/d1kg0c_-d1qdda_.fasta

./group343/reference/d1kg0c_-d1tn3__.fasta

./group343/reference/d1kg0c_-d1tsg__.fasta

./group343/reference/d1koe__-d1prea1.fasta

./group343/reference/d1koe__-d1prtb2.fasta

./group343/reference/d1koe__-d1qdda_.fasta

./group343/reference/d1koe__-d1tn3__.fasta

./group343/reference/d1li1a1-d1prea1.fasta

./group343/reference/d1li1a1-d1prtb2.fasta

./group343/reference/d1li1a1-d1qdda_.fasta

./group343/reference/d1li1a1-d1tn3__.fasta

./group343/reference/d1li1a1-d1tsg__.fasta

./group343/reference/d1li1a2-d1tn3__.fasta

./group343/reference/d1prea1-d1tn3__.fasta

./group343/reference/d1prtb2-d1qdda_.fasta

./group343/reference/d1prtb2-d1tn3__.fasta

./group343/reference/d1prtb2-d1tsg__.fasta

./group343/reference/d1tn3__-d1tsg__.fasta

./group346/reference/d1bsg__-d1ci9a_.fasta

./group346/reference/d1bsg__-d1ei5a3.fasta

./group346/reference/d1bsg__-d1es5a_.fasta

./group346/reference/d1bsg__-d1qmea4.fasta

./group346/reference/d1buea_-d1ci9a_.fasta

./group346/reference/d1buea_-d1ei5a3.fasta

./group346/reference/d1buea_-d1es5a_.fasta

./group346/reference/d1buea_-d1qmea4.fasta

./group346/reference/d1ci9a_-d1e25a_.fasta

./group346/reference/d1ci9a_-d1g6aa_.fasta

./group346/reference/d1ci9a_-d1ghpa_.fasta

./group346/reference/d1ci9a_-d1iyoa_.fasta

./group346/reference/d1ci9a_-d1m40a_.fasta

./group346/reference/d1ci9a_-d1qmea4.fasta

./group346/reference/d1ci9a_-d4blma_.fasta

./group346/reference/d1e25a_-d1ei5a3.fasta

./group346/reference/d1e25a_-d1es5a_.fasta

./group346/reference/d1e25a_-d1qmea4.fasta

./group346/reference/d1ei5a3-d1es5a_.fasta

./group346/reference/d1ei5a3-d1g6aa_.fasta

./group346/reference/d1ei5a3-d1ghpa_.fasta

./group346/reference/d1ei5a3-d1iyoa_.fasta

./group346/reference/d1ei5a3-d1m40a_.fasta

./group346/reference/d1ei5a3-d1mfoa_.fasta

./group346/reference/d1ei5a3-d1qmea4.fasta

./group346/reference/d1ei5a3-d4blma_.fasta

./group346/reference/d1es5a_-d1g6aa_.fasta

./group346/reference/d1es5a_-d1m40a_.fasta

./group346/reference/d1es5a_-d1qmea4.fasta

./group346/reference/d1es5a_-d4blma_.fasta

./group346/reference/d1g6aa_-d1qmea4.fasta

./group346/reference/d1ghpa_-d1qmea4.fasta

./group346/reference/d1iyoa_-d1qmea4.fasta

./group346/reference/d1m40a_-d1qmea4.fasta

./group346/reference/d1mfoa_-d1qmea4.fasta

./group346/reference/d1qmea4-d4blma_.fasta

./group348/reference/d1is2a3-d1ivha2.fasta

./group348/reference/d1is2a3-d3mdda2.fasta

./group349/reference/d1g0ha_-d1inp__.fasta

./group349/reference/d1inp__-d1lbva_.fasta

./group350/reference/d1hhsa_-d1mml__.fasta

./group350/reference/d1hhsa_-d1tgoa2.fasta

./group350/reference/d1hhsa_-d1vrta2.fasta

./group350/reference/d1jiha_-d1l3sa2.fasta

./group350/reference/d1jiha_-d1tgoa2.fasta

./group350/reference/d1l3sa2-d1mml__.fasta

./group350/reference/d1mml__-d1tgoa2.fasta

./group350/reference/d1tgoa2-d1vrta2.fasta

./group351/reference/d1daaa_-d1ekfa_.fasta

./group352/reference/d1feza_-d1k1ea_.fasta

./group352/reference/d1feza_-d1l6ra_.fasta

./group352/reference/d1feza_-d1mh9a_.fasta

./group352/reference/d1feza_-d1o08a_.fasta

./group352/reference/d1feza_-d1qq5a_.fasta

./group352/reference/d1feza_-d1zrn__.fasta

./group352/reference/d1k1ea_-d1mh9a_.fasta

./group352/reference/d1k1ea_-d1o08a_.fasta

./group352/reference/d1k1ea_-d1qq5a_.fasta

./group352/reference/d1k1ea_-d1zrn__.fasta

./group352/reference/d1l6ra_-d1mh9a_.fasta

./group352/reference/d1l6ra_-d1o08a_.fasta

./group352/reference/d1l6ra_-d1zrn__.fasta

./group352/reference/d1mh9a_-d1o08a_.fasta

./group352/reference/d1mh9a_-d1qq5a_.fasta

./group352/reference/d1mh9a_-d1zrn__.fasta

./group352/reference/d1o08a_-d1zrn__.fasta

./group356/reference/d1ddba_-d1f16a_.fasta

./group356/reference/d1k3ka_-d1o0la_.fasta

./group358/reference/d1mm4a_-d1qj8a_.fasta

./group358/reference/d1p4ta_-d1qj8a_.fasta

./group359/reference/d1a0tp_-d1hxxa_.fasta

./group359/reference/d1a0tp_-d2por__.fasta

./group359/reference/d1by5a_-d1kmoa_.fasta

./group359/reference/d1by5a_-d3prn__.fasta

./group359/reference/d1hxxa_-d2mpra_.fasta

./group359/reference/d1hxxa_-d2por__.fasta

./group359/reference/d1hxxa_-d3prn__.fasta

./group359/reference/d1kmoa_-d2por__.fasta

./group359/reference/d2mpra_-d2por__.fasta

./group361/reference/d1agg__-d1c6wa_.fasta

./group361/reference/d1agg__-d1d1ha_.fasta

./group361/reference/d1agg__-d1g9pa_.fasta

./group361/reference/d1agg__-d1ju8a_.fasta

./group361/reference/d1agg__-d1niya_.fasta

./group361/reference/d1agg__-d1vtx__.fasta

./group361/reference/d1axh__-d1koza_.fasta

./group361/reference/d1axh__-d1kqha_.fasta

./group361/reference/d1c6wa_-d1ju8a_.fasta

./group361/reference/d1c6wa_-d1niya_.fasta

./group361/reference/d1cixa_-d1ju8a_.fasta

./group361/reference/d1d1ha_-d1ju8a_.fasta

./group361/reference/d1d1ha_-d1vtx__.fasta

./group361/reference/d1dl0a_-d1ju8a_.fasta

./group361/reference/d1dl0a_-d1kqha_.fasta

./group361/reference/d1dl0a_-d1lqra_.fasta

./group361/reference/d1dl0a_-d1vtx__.fasta

./group361/reference/d1eit__-d1ju8a_.fasta

./group361/reference/d1eit__-d1lqra_.fasta

./group361/reference/d1eit__-d1niya_.fasta

./group361/reference/d1emxa_-d1ju8a_.fasta

./group361/reference/d1emxa_-d1lqra_.fasta

./group361/reference/d1g9pa_-d1ju8a_.fasta

./group361/reference/d1i25a_-d1i26a_.fasta

./group361/reference/d1i25a_-d1kqha_.fasta

./group361/reference/d1i25a_-d1niya_.fasta

./group361/reference/d1i26a_-d1ju8a_.fasta

./group361/reference/d1ju8a_-d1lupa_.fasta

./group361/reference/d1ju8a_-d1niya_.fasta

./group361/reference/d1ju8a_-d1qk7a_.fasta

./group361/reference/d1ju8a_-d1vtx__.fasta

./group361/reference/d1niya_-d1vtx__.fasta

./group361/reference/d1qk6a_-d1vtx__.fasta

./group361/reference/d1qk7a_-d1vtx__.fasta

./group362/reference/d1aho__-d1mm0a_.fasta

./group362/reference/d1bcg__-d1gps__.fasta

./group362/reference/d1bcg__-d1jkza_.fasta

./group362/reference/d1bcg__-d1mm0a_.fasta

./group362/reference/d1bcg__-d1qkya_.fasta

./group362/reference/d1bmr__-d1mm0a_.fasta

./group362/reference/d1c55a_-d1mm0a_.fasta

./group362/reference/d1c55a_-d1sis__.fasta

./group362/reference/d1cmr__-d1nrb__.fasta

./group362/reference/d1gps__-d1npia_.fasta

./group362/reference/d1gps__-d1nrb__.fasta

./group362/reference/d1gps__-d1sis__.fasta

./group362/reference/d1i2ua_-d1mm0a_.fasta

./group362/reference/d1jkza_-d1mm0a_.fasta

./group362/reference/d1jkza_-d1npia_.fasta

./group362/reference/d1jkza_-d1qkya_.fasta

./group362/reference/d1mm0a_-d1nrb__.fasta

./group362/reference/d1mm0a_-d1scy__.fasta

./group362/reference/d1myn__-d1qkya_.fasta

./group362/reference/d1ne5a_-d1npia_.fasta

./group362/reference/d1sis__-d1tsk__.fasta

./group363/reference/d1h59b_-d1n8yc4.fasta

./group363/reference/d1h59b_-d1nqla3.fasta

./group363/reference/d1h59b_-d1nqla4.fasta

./group364/reference/d1imt_1-d1imt_2.fasta

./group365/reference/d1b9wa1-d1b9wa2.fasta

./group365/reference/d1b9wa1-d1cvua2.fasta

./group365/reference/d1b9wa1-d1dx5i1.fasta

./group365/reference/d1b9wa1-d1emo_1.fasta

./group365/reference/d1b9wa1-d1fjsl_.fasta

./group365/reference/d1b9wa1-d1hae__.fasta

./group365/reference/d1b9wa1-d1hz8a2.fasta

./group365/reference/d1b9wa1-d1ijqa2.fasta

./group365/reference/d1b9wa1-d1ioxa_.fasta

./group365/reference/d1b9wa1-d1klil_.fasta

./group365/reference/d1b9wa1-d1klo_1.fasta

./group365/reference/d1b9wa1-d1klo_3.fasta

./group365/reference/d1b9wa1-d1lmja1.fasta

./group365/reference/d1b9wa1-d1m1xb5.fasta

./group365/reference/d1b9wa1-d1urk_1.fasta

./group365/reference/d1b9wa2-d1klo_3.fasta

./group365/reference/d1b9wa2-d1ob1c1.fasta

./group365/reference/d1b9wa2-d1tpg_1.fasta

./group365/reference/d1b9wa2-d3tgf__.fasta

./group365/reference/d1cvua2-d1klil_.fasta

./group365/reference/d1cvua2-d1klo_2.fasta

./group365/reference/d1cvua2-d1ob1c1.fasta

./group365/reference/d1dx5i1-d1ob1c1.fasta

./group365/reference/d1emo_1-d1klo_1.fasta

./group365/reference/d1fjsl_-d1klo_2.fasta

./group365/reference/d1fjsl_-d1m1xb5.fasta

./group365/reference/d1fjsl_-d1ob1c1.fasta

./group365/reference/d1fjsl_-d1urk_1.fasta

./group365/reference/d1hz8a1-d1ioxa_.fasta

./group365/reference/d1hz8a1-d1klo_1.fasta

./group365/reference/d1hz8a1-d1klo_2.fasta

./group365/reference/d1hz8a1-d1l3ya_.fasta

./group365/reference/d1hz8a1-d1ob1c1.fasta

./group365/reference/d1hz8a1-d1tpg_1.fasta

./group365/reference/d1hz8a1-d1urk_1.fasta

./group365/reference/d1hz8a2-d1ob1c1.fasta

./group365/reference/d1hz8a2-d1xdtr_.fasta

./group365/reference/d1ijqa2-d1klo_1.fasta

./group365/reference/d1ijqa2-d1klo_2.fasta

./group365/reference/d1ijqa2-d1klo_3.fasta

./group365/reference/d1ijqa2-d1ob1c1.fasta

./group365/reference/d1ijqa2-d1urk_1.fasta

./group365/reference/d1ijqa2-d1xdtr_.fasta

./group365/reference/d1ioxa_-d1ob1c1.fasta

./group365/reference/d1jl9a_-d1ob1c1.fasta

./group365/reference/d1jl9a_-d1urk_1.fasta

./group365/reference/d1klil_-d1klo_1.fasta

./group365/reference/d1klil_-d1urk_1.fasta

./group365/reference/d1klo_1-d1l3ya_.fasta

./group365/reference/d1klo_1-d1urk_1.fasta

./group365/reference/d1klo_2-d1m1xb5.fasta

./group365/reference/d1klo_2-d1xdtr_.fasta

./group365/reference/d1klo_3-d1m1xb5.fasta

./group365/reference/d1m1xb5-d1rfnb_.fasta

./group365/reference/d1m1xb5-d3tgf__.fasta

./group365/reference/d1ob1c1-d1urk_1.fasta

./group365/reference/d1ob1c1-d3tgf__.fasta

./group365/reference/d1rfnb_-d1tpg_1.fasta

./group365/reference/d1rfnb_-d1urk_1.fasta

./group365/reference/d1urk_1-d1xdtr_.fasta

./group366/reference/d1dec__-d1skz_1.fasta

./group366/reference/d1dec__-d1skz_2.fasta

./group366/reference/d1e0fi_-d1skz_2.fasta

./group367/reference/d1f94a_-d1m9za_.fasta

./group367/reference/d1jgka_-d1m9za_.fasta

./group367/reference/d1m9za_-d3ebx__.fasta

./group368/reference/d1aapa_-d1d0da_.fasta

./group368/reference/d1aapa_-d1tocr1.fasta

./group368/reference/d1bf0__-d1d0da_.fasta

./group368/reference/d1bf0__-d1tocr1.fasta

./group368/reference/d1bf0__-d1tocr2.fasta

./group368/reference/d1bik_1-d1d0da_.fasta

./group368/reference/d1bik_2-d1d0da_.fasta

./group368/reference/d1bunb_-d1tocr1.fasta

./group368/reference/d1d0da_-d1jc6a_.fasta

./group368/reference/d1d0da_-d1ktha_.fasta

./group368/reference/d1d0da_-d1tocr2.fasta

./group368/reference/d1jc6a_-d1tocr1.fasta

./group368/reference/d1ktha_-d1tocr1.fasta

./group368/reference/d1tfxc_-d1tocr1.fasta

./group369/reference/d1atx__-d1b8wa_.fasta

./group369/reference/d1atx__-d1dfna_.fasta

./group369/reference/d1atx__-d1ewsa_.fasta

./group369/reference/d1atx__-d1ijva_.fasta

./group369/reference/d1b8wa_-d1dfna_.fasta

./group369/reference/d1b8wa_-d1ewsa_.fasta

./group369/reference/d1bnb__-d1ewsa_.fasta

./group369/reference/d1bnb__-d1sh1__.fasta

./group369/reference/d1d6ba_-d1dfna_.fasta

./group369/reference/d1d6ba_-d1ewsa_.fasta

./group369/reference/d1dfna_-d1sh1__.fasta

./group369/reference/d1dfna_-d2bds__.fasta

./group369/reference/d1e4ta_-d1ewsa_.fasta

./group369/reference/d1e4ta_-d1sh1__.fasta

./group369/reference/d1ewsa_-d1fd3a_.fasta

./group369/reference/d1ewsa_-d1h5oa_.fasta

./group369/reference/d1fd3a_-d1sh1__.fasta

./group369/reference/d1ijva_-d2bds__.fasta

./group370/reference/d1bhta1-d1hkya_.fasta

./group370/reference/d1bhta1-d1i8na_.fasta

./group370/reference/d1hkya_-d1i8na_.fasta

./group373/reference/d1h8pa1-d2hpqp_.fasta

./group373/reference/d1l6ja5-d2hpqp_.fasta

./group374/reference/d1ce3a_-d1ldtl_.fasta

./group374/reference/d1ce3a_-d1pce__.fasta

./group374/reference/d1ce3a_-d1sgpi_.fasta

./group374/reference/d1ce3a_-d1tbrr1.fasta

./group374/reference/d1ce3a_-d1tbrr2.fasta

./group374/reference/d1ce3a_-d1tgsi_.fasta

./group374/reference/d1iw4a_-d1nuba3.fasta

./group374/reference/d1iw4a_-d1pce__.fasta

./group374/reference/d1iw4a_-d1tbrr1.fasta

./group374/reference/d1ldtl_-d4sgbi_.fasta

./group374/reference/d1pce__-d4sgbi_.fasta

./group376/reference/d1aoca_-d1fltv_.fasta

./group376/reference/d1aoca_-d1hcnb_.fasta

./group376/reference/d1aoca_-d2tgi__.fasta

./group376/reference/d1bnda_-d1fl7b_.fasta

./group376/reference/d1bnda_-d1hcnb_.fasta

./group376/reference/d1bnda_-d1lxia_.fasta

./group376/reference/d1bnda_-d2tgi__.fasta

./group376/reference/d1fl7b_-d1fltv_.fasta

./group376/reference/d1fltv_-d1hcnb_.fasta

./group376/reference/d1fltv_-d1jpya_.fasta

./group376/reference/d1fltv_-d1lxia_.fasta

./group376/reference/d1fltv_-d2tgi__.fasta

./group376/reference/d1hcna_-d1hcnb_.fasta

./group376/reference/d1hcna_-d1jpya_.fasta

./group376/reference/d1jpya_-d1lxia_.fasta

./group377/reference/d1ckla1-d1h03p1.fasta

./group377/reference/d1ckla1-d1ly2a1.fasta

./group377/reference/d1ckla1-d1quba2.fasta

./group377/reference/d1ckla1-d1quba3.fasta

./group377/reference/d1ckla1-d1quba4.fasta

./group377/reference/d1ckla2-d1g40a1.fasta

./group377/reference/d1ckla2-d1gkna1.fasta

./group377/reference/d1ckla2-d1hfi__.fasta

./group377/reference/d1ckla2-d1nwva1.fasta

./group377/reference/d1ckla2-d1quba3.fasta

./group377/reference/d1g40a1-d1g40a4.fasta

./group377/reference/d1g40a1-d1gkga2.fasta

./group377/reference/d1g40a1-d1h03p2.fasta

./group377/reference/d1g40a1-d1ly2a1.fasta

./group377/reference/d1g40a1-d1ly2a2.fasta

./group377/reference/d1g40a2-d1quba3.fasta

./group377/reference/d1g40a2-d1quba5.fasta

./group377/reference/d1g40a3-d1quba3.fasta

./group377/reference/d1g40a4-d1gkna1.fasta

./group377/reference/d1gkga2-d1gpza2.fasta

./group377/reference/d1gkga2-d1quba1.fasta

./group377/reference/d1gkga2-d1quba5.fasta

./group377/reference/d1gkna1-d1gkna2.fasta

./group377/reference/d1gkna1-d1gpza2.fasta

./group377/reference/d1gkna1-d1ly2a2.fasta

./group377/reference/d1gkna1-d1quba2.fasta

./group377/reference/d1gkna1-d1quba4.fasta

./group377/reference/d1gkna1-d1quba5.fasta

./group377/reference/d1gpza2-d1hcc__.fasta

./group377/reference/d1h03p2-d1nwva1.fasta

./group377/reference/d1hcc__-d1quba3.fasta

./group377/reference/d1ly2a1-d1quba1.fasta

./group377/reference/d1ly2a1-d1quba3.fasta

./group377/reference/d1ly2a2-d1nwva1.fasta

./group377/reference/d1nwva1-d1quba2.fasta

./group377/reference/d1nwva1-d1quba4.fasta

./group377/reference/d1quba1-d1quba4.fasta

./group377/reference/d1quba1-d1quba5.fasta

./group380/reference/d1d4va3-d1exta3.fasta

./group380/reference/d1d4va3-d1jmab1.fasta

./group380/reference/d1exta2-d1oqdk_.fasta

./group380/reference/d1exta2-d1oqek_.fasta

./group380/reference/d1exta3-d1oqdk_.fasta

./group380/reference/d1exta3-d1oqek_.fasta

./group380/reference/d1jmab1-d1oqek_.fasta

./group381/reference/d1e88a3-d1tpg_2.fasta

./group383/reference/d1bhi__-d1fv5a_.fasta

./group383/reference/d1bhi__-d1rmd_1.fasta

./group383/reference/d1bhi__-d1yuja_.fasta

./group383/reference/d1bhi__-d2drpa1.fasta

./group383/reference/d1fu9a_-d1rmd_1.fasta

./group383/reference/d1fu9a_-d1tf3a2.fasta

./group383/reference/d1fu9a_-d1tf3a3.fasta

./group383/reference/d1fu9a_-d1ubdc3.fasta

./group383/reference/d1fu9a_-d1yuja_.fasta

./group383/reference/d1fu9a_-d1zfd__.fasta

./group383/reference/d1fu9a_-d2drpa1.fasta

./group383/reference/d1fu9a_-d2glia1.fasta

./group383/reference/d1fu9a_-d2glia2.fasta

./group383/reference/d1fu9a_-d2glia4.fasta

./group383/reference/d1fu9a_-d5znf__.fasta

./group383/reference/d1fv5a_-d1rmd_1.fasta

./group383/reference/d1fv5a_-d1yuja_.fasta

./group383/reference/d1ncs__-d1rmd_1.fasta

./group383/reference/d1njqa_-d2drpa1.fasta

./group383/reference/d1njqa_-d2glia1.fasta

./group383/reference/d1paa__-d1rmd_1.fasta

./group383/reference/d1rmd_1-d1tf3a3.fasta

./group383/reference/d1rmd_1-d1ubdc3.fasta

./group383/reference/d1rmd_1-d1yuja_.fasta

./group383/reference/d1rmd_1-d1zfd__.fasta

./group383/reference/d1rmd_1-d2drpa1.fasta

./group383/reference/d1rmd_1-d2glia2.fasta

./group383/reference/d1rmd_1-d2glia4.fasta

./group383/reference/d1rmd_1-d5znf__.fasta

./group383/reference/d1tf3a1-d2drpa1.fasta

./group383/reference/d1tf3a1-d2glia1.fasta

./group383/reference/d1tf3a3-d2glia2.fasta

./group383/reference/d1tf6a4-d1yuja_.fasta

./group383/reference/d1tf6a4-d2drpa1.fasta

./group383/reference/d1ubdc3-d2drpa1.fasta

./group383/reference/d1yuja_-d2glia1.fasta

./group383/reference/d1yuja_-d2glia2.fasta

./group383/reference/d1yuja_-d2glia4.fasta

./group383/reference/d2drpa1-d2glia2.fasta

./group383/reference/d2glia2-d5znf__.fasta

./group385/reference/d1a7i_2-d1d4ua2.fasta

./group385/reference/d1a7i_2-d1fjgn_.fasta

./group385/reference/d1a7i_2-d1g47a2.fasta

./group385/reference/d1a7i_2-d1gnf__.fasta

./group385/reference/d1a7i_2-d1j2oa1.fasta

./group385/reference/d1a7i_2-d1jj2t_.fasta

./group385/reference/d1a7i_2-d1m3va1.fasta

./group385/reference/d1a7i_2-d1m3va2.fasta

./group385/reference/d1a7i_2-d1nypa1.fasta

./group385/reference/d1a7i_2-d1nypa2.fasta

./group385/reference/d1a7i_2-d3gata_.fasta

./group385/reference/d1a7i_2-d7gata_.fasta

./group385/reference/d1d4ua2-d1dsza_.fasta

./group385/reference/d1d4ua2-d1g47a2.fasta

./group385/reference/d1d4ua2-d1jj2t_.fasta

./group385/reference/d1d4ua2-d1l1za3.fasta

./group385/reference/d1d4ua2-d1m3va2.fasta

./group385/reference/d1d4ua2-d1nypa1.fasta

./group385/reference/d1d4ua2-d3gata_.fasta

./group385/reference/d1d4ua2-d7gata_.fasta

./group385/reference/d1dsza_-d1g47a1.fasta

./group385/reference/d1dsza_-d1k3xa3.fasta

./group385/reference/d1dsza_-d1l1za3.fasta

./group385/reference/d1dsza_-d1m3va1.fasta

./group385/reference/d1dsza_-d1m3va2.fasta

./group385/reference/d1dsza_-d1nypa1.fasta

./group385/reference/d1dsza_-d1nypa2.fasta

./group385/reference/d1dsza_-d3gata_.fasta

./group385/reference/d1fjgn_-d1g47a1.fasta

./group385/reference/d1fjgn_-d1ibia2.fasta

./group385/reference/d1fjgn_-d1j2oa1.fasta

./group385/reference/d1fjgn_-d1j2oa2.fasta

./group385/reference/d1fjgn_-d1jj2t_.fasta

./group385/reference/d1fjgn_-d1kb2a_.fasta

./group385/reference/d1fjgn_-d1nypa2.fasta

./group385/reference/d1fjgn_-d3gata_.fasta

./group385/reference/d1g47a1-d1gnf__.fasta

./group385/reference/d1g47a1-d1jj2t_.fasta

./group385/reference/d1g47a1-d1k3xa3.fasta

./group385/reference/d1g47a1-d1lata_.fasta

./group385/reference/d1g47a1-d1lv3a_.fasta

./group385/reference/d1g47a1-d1m3va2.fasta

./group385/reference/d1g47a1-d2nllb_.fasta

./group385/reference/d1g47a2-d1gnf__.fasta

./group385/reference/d1g47a2-d1jj2t_.fasta

./group385/reference/d1g47a2-d1k3xa3.fasta

./group385/reference/d1g47a2-d1kb2a_.fasta

./group385/reference/d1g47a2-d1lata_.fasta

./group385/reference/d1g47a2-d1lo1a_.fasta

./group385/reference/d1g47a2-d1lv3a_.fasta

./group385/reference/d1g47a2-d1m3va1.fasta

./group385/reference/d1g47a2-d1nypa1.fasta

./group385/reference/d1g47a2-d2nllb_.fasta

./group385/reference/d1g47a2-d3gata_.fasta

./group385/reference/d1gnf__-d1j2oa1.fasta

./group385/reference/d1gnf__-d1nypa2.fasta

./group385/reference/d1ibia2-d1j2oa1.fasta

./group385/reference/d1ibia2-d1nypa1.fasta

./group385/reference/d1ibia2-d7gata_.fasta

./group385/reference/d1iml_2-d1jj2t_.fasta

./group385/reference/d1iml_2-d1m3va1.fasta

./group385/reference/d1iml_2-d1nypa1.fasta

./group385/reference/d1j2oa1-d1j2oa2.fasta

./group385/reference/d1j2oa1-d1jj2t_.fasta

./group385/reference/d1j2oa1-d1lata_.fasta

./group385/reference/d1j2oa1-d1lo1a_.fasta

./group385/reference/d1j2oa1-d1m3va2.fasta

./group385/reference/d1j2oa1-d1nypa2.fasta

./group385/reference/d1j2oa1-d2nllb_.fasta

./group385/reference/d1j2oa1-d3gata_.fasta

./group385/reference/d1j2oa1-d7gata_.fasta

./group385/reference/d1j2oa2-d3gata_.fasta

./group385/reference/d1jj2t_-d1kb2a_.fasta

./group385/reference/d1jj2t_-d1lata_.fasta

./group385/reference/d1jj2t_-d1lo1a_.fasta

./group385/reference/d1jj2t_-d1m3va1.fasta

./group385/reference/d1jj2t_-d1nypa1.fasta

./group385/reference/d1jj2t_-d1nypa2.fasta

./group385/reference/d1jj2t_-d2nllb_.fasta

./group385/reference/d1jj2t_-d3gata_.fasta

./group385/reference/d1jj2t_-d7gata_.fasta

./group385/reference/d1k3xa3-d1kb2a_.fasta

./group385/reference/d1k3xa3-d1lv3a_.fasta

./group385/reference/d1k3xa3-d1nypa2.fasta

./group385/reference/d1k3xa3-d3gata_.fasta

./group385/reference/d1k3xa3-d7gata_.fasta

./group385/reference/d1kb2a_-d1l1za3.fasta

./group385/reference/d1kb2a_-d1m3va1.fasta

./group385/reference/d1kb2a_-d1m3va2.fasta

./group385/reference/d1kb2a_-d1nypa1.fasta

./group385/reference/d1kb2a_-d1nypa2.fasta

./group385/reference/d1kb2a_-d3gata_.fasta

./group385/reference/d1kb2a_-d7gata_.fasta

./group385/reference/d1l1za3-d1lo1a_.fasta

./group385/reference/d1l1za3-d1lv3a_.fasta

./group385/reference/d1l1za3-d1m3va2.fasta

./group385/reference/d1l1za3-d1nypa1.fasta

./group385/reference/d1l1za3-d2nllb_.fasta

./group385/reference/d1l1za3-d7gata_.fasta

./group385/reference/d1lata_-d1m3va1.fasta

./group385/reference/d1lata_-d1m3va2.fasta

./group385/reference/d1lata_-d1nypa1.fasta

./group385/reference/d1lata_-d1nypa2.fasta

./group385/reference/d1lata_-d3gata_.fasta

./group385/reference/d1lata_-d7gata_.fasta

./group385/reference/d1lo1a_-d1m3va2.fasta

./group385/reference/d1lv3a_-d3gata_.fasta

./group385/reference/d1m3va1-d2nllb_.fasta

./group385/reference/d1m3va1-d7gata_.fasta

./group385/reference/d1m3va2-d1nypa1.fasta

./group385/reference/d1m3va2-d2nllb_.fasta

./group385/reference/d1nypa1-d3gata_.fasta

./group385/reference/d1nypa1-d7gata_.fasta

./group385/reference/d1nypa2-d2nllb_.fasta

./group385/reference/d1nypa2-d3gata_.fasta

./group385/reference/d1nypa2-d7gata_.fasta

./group385/reference/d2nllb_-d3gata_.fasta

./group385/reference/d2nllb_-d7gata_.fasta

./group388/reference/d1d0qa_-d1dl6a_.fasta

./group388/reference/d1d0qa_-d1i50i1.fasta

./group388/reference/d1d0qa_-d1qyp__.fasta

./group388/reference/d1d0qa_-d1tfi__.fasta

./group388/reference/d1d0qa_-d1yua_2.fasta

./group388/reference/d1dl6a_-d1i50i1.fasta

./group388/reference/d1dl6a_-d1yua_1.fasta

./group388/reference/d1i50i1-d1qyp__.fasta

./group388/reference/d1i50i1-d1tfi__.fasta

./group388/reference/d1i50i2-d1yua_1.fasta

./group388/reference/d1tfi__-d1yua_1.fasta

./group389/reference/d1dxga_-d1ocrf_.fasta

./group389/reference/d1h7va_-d1ocrf_.fasta

./group389/reference/d1lkoa2-d1ocrf_.fasta

./group390/reference/d1jj2y_-d1jj2z_.fasta

./group390/reference/d1jj2z_-d1nvha_.fasta

./group391/reference/d1chc__-d1n87a_.fasta

./group391/reference/d1e4ua_-d1ldjb_.fasta

./group391/reference/d1e4ua_-d1n87a_.fasta

./group391/reference/d1fbva4-d1n87a_.fasta

./group391/reference/d1g25a_-d1ldjb_.fasta

./group391/reference/d1g25a_-d1n87a_.fasta

./group391/reference/d1iyma_-d1n87a_.fasta

./group391/reference/d1jm7a_-d1n87a_.fasta

./group391/reference/d1jm7b_-d1n87a_.fasta

./group391/reference/d1ldjb_-d1n87a_.fasta

./group391/reference/d1n87a_-d1rmd_2.fasta

./group392/reference/d1dmc__-d1m0ga_.fasta

./group392/reference/d1dmc__-d1qjka_.fasta

./group392/reference/d1fmya_-d1m0ga_.fasta

./group392/reference/d1fmya_-d1qjka_.fasta

./group392/reference/d1m0ga_-d1qjka_.fasta

./group393/reference/d1e53a_-d1kbea_.fasta

./group394/reference/d1dvpa2-d1mm2a_.fasta

./group394/reference/d1f62a_-d1vfya_.fasta

./group394/reference/d1mm2a_-d1vfya_.fasta

./group396/reference/d1a8p_1-d1cqxa2.fasta

./group396/reference/d1a8p_1-d1f20a1.fasta

./group396/reference/d1a8p_1-d1i7pa1.fasta

./group396/reference/d1a8p_1-d1i8da1.fasta

./group396/reference/d1a8p_1-d1i8da2.fasta

./group396/reference/d1a8p_1-d1jb9a1.fasta

./group396/reference/d1a8p_1-d1kzla1.fasta

./group396/reference/d1a8p_1-d1kzla2.fasta

./group396/reference/d1cqxa2-d1ddga1.fasta

./group396/reference/d1cqxa2-d1f20a1.fasta

./group396/reference/d1cqxa2-d1i7pa1.fasta

./group396/reference/d1cqxa2-d1i8da1.fasta

./group396/reference/d1cqxa2-d1ja1a1.fasta

./group396/reference/d1cqxa2-d1kzla1.fasta

./group396/reference/d1cqxa2-d1que_1.fasta

./group396/reference/d1cqxa2-d2cnd_1.fasta

./group396/reference/d1ddga1-d1ep3b1.fasta

./group396/reference/d1ddga1-d1i7pa1.fasta

./group396/reference/d1ddga1-d1i8da1.fasta

./group396/reference/d1ddga1-d1i8da2.fasta

./group396/reference/d1ddga1-d1krha1.fasta

./group396/reference/d1ddga1-d2cnd_1.fasta

./group396/reference/d1ep3b1-d1f20a1.fasta

./group396/reference/d1ep3b1-d1i7pa1.fasta

./group396/reference/d1ep3b1-d1i8da1.fasta

./group396/reference/d1ep3b1-d1i8da2.fasta

./group396/reference/d1ep3b1-d1ja1a1.fasta

./group396/reference/d1ep3b1-d1krha1.fasta

./group396/reference/d1ep3b1-d1kzla1.fasta

./group396/reference/d1ep3b1-d1kzla2.fasta

./group396/reference/d1f20a1-d1i8da1.fasta

./group396/reference/d1f20a1-d1kzla1.fasta

./group396/reference/d1f20a1-d1kzla2.fasta

./group396/reference/d1f20a1-d2cnd_1.fasta

./group396/reference/d1f20a1-d2pia_1.fasta

./group396/reference/d1fnc_1-d1i8da1.fasta

./group396/reference/d1fnc_1-d1i8da2.fasta

./group396/reference/d1fnc_1-d1kzla1.fasta

./group396/reference/d1fnc_1-d1kzla2.fasta

./group396/reference/d1fnc_1-d2cnd_1.fasta

./group396/reference/d1i7pa1-d1i8da1.fasta

./group396/reference/d1i7pa1-d1i8da2.fasta

./group396/reference/d1i7pa1-d1ja1a1.fasta

./group396/reference/d1i7pa1-d1kzla1.fasta

./group396/reference/d1i7pa1-d1kzla2.fasta

./group396/reference/d1i7pa1-d2pia_1.fasta

./group396/reference/d1i8da1-d1ja1a1.fasta

./group396/reference/d1i8da1-d1jb9a1.fasta

./group396/reference/d1i8da1-d1krha1.fasta

./group396/reference/d1i8da1-d1que_1.fasta

./group396/reference/d1i8da1-d2pia_1.fasta

./group396/reference/d1i8da2-d1ja1a1.fasta

./group396/reference/d1i8da2-d1que_1.fasta

./group396/reference/d1i8da2-d2cnd_1.fasta

./group396/reference/d1ja1a1-d1krha1.fasta

./group396/reference/d1ja1a1-d1kzla2.fasta

./group396/reference/d1ja1a1-d2cnd_1.fasta

./group396/reference/d1ja1a1-d2pia_1.fasta

./group396/reference/d1jb9a1-d1kzla1.fasta

./group396/reference/d1jb9a1-d1kzla2.fasta

./group396/reference/d1jb9a1-d2cnd_1.fasta

./group396/reference/d1jb9a1-d2pia_1.fasta

./group396/reference/d1krha1-d1kzla1.fasta

./group396/reference/d1krha1-d1kzla2.fasta

./group396/reference/d1krha1-d2pia_1.fasta

./group396/reference/d1kzla1-d1que_1.fasta

./group396/reference/d1kzla1-d2cnd_1.fasta

./group396/reference/d1kzla2-d1que_1.fasta

./group396/reference/d1kzla2-d2cnd_1.fasta

./group396/reference/d1kzla2-d2pia_1.fasta

./group396/reference/d1que_1-d2pia_1.fasta

./group396/reference/d2cnd_1-d2pia_1.fasta

./group397/reference/d1ezva1-d1ezva2.fasta

./group397/reference/d1ezva1-d1ezvb1.fasta

./group397/reference/d1ezva1-d1hr6a1.fasta

./group397/reference/d1ezva1-d1hr6b2.fasta

./group397/reference/d1ezva1-d1l0la2.fasta

./group397/reference/d1ezva1-d1l0lb1.fasta

./group397/reference/d1ezva1-d1l0lb2.fasta

./group397/reference/d1ezva2-d1ezvb1.fasta

./group397/reference/d1ezva2-d1ezvb2.fasta

./group397/reference/d1ezva2-d1hr6b1.fasta

./group397/reference/d1ezva2-d1l0lb1.fasta

./group397/reference/d1ezvb1-d1ezvb2.fasta

./group397/reference/d1ezvb1-d1hr6a2.fasta

./group397/reference/d1ezvb1-d1hr6b2.fasta

./group397/reference/d1ezvb1-d1l0la1.fasta

./group397/reference/d1ezvb1-d1l0la2.fasta

./group397/reference/d1ezvb2-d1hr6a2.fasta

./group397/reference/d1ezvb2-d1hr6b2.fasta

./group397/reference/d1ezvb2-d1l0la2.fasta

./group397/reference/d1hr6a1-d1l0la2.fasta

./group397/reference/d1hr6a1-d1l0lb2.fasta

./group397/reference/d1hr6a2-d1l0la1.fasta

./group397/reference/d1hr6b1-d1l0lb2.fasta

./group397/reference/d1hr6b2-d1l0la1.fasta

./group397/reference/d1hr6b2-d1l0lb1.fasta

./group397/reference/d1l0la1-d1l0lb2.fasta

./group397/reference/d1l0la2-d1l0lb1.fasta

./group398/reference/d1gjja1-d1jeia_.fasta

./group400/reference/d1h3za_-d1mhna_.fasta

./group400/reference/d1h3za_-d1oi1a1.fasta

./group400/reference/d1h3za_-d1oi1a2.fasta

./group400/reference/d1mhna_-d1oi1a1.fasta

./group401/reference/d1jdqa_-d1pava_.fasta

./group401/reference/d1je3a_-d1pava_.fasta

./group402/reference/d1gw5m2-d1gw5s_.fasta

./group402/reference/d1gw5m2-d1h8ma_.fasta

./group403/reference/d1hc7a3-d1nj1a2.fasta

./group404/reference/d1jeqa1-d1kcfa1.fasta

./group405/reference/d1ayl_2-d1khba2.fasta

./group405/reference/d1ii2a2-d1khba2.fasta

./group407/reference/d1hn0a4-d1jova_.fasta

./group407/reference/d1hn0a4-d1nsza_.fasta

./group407/reference/d1j0ma3-d1nsza_.fasta

./group407/reference/d1jova_-d1n7oa3.fasta

./group407/reference/d1jova_-d1nsza_.fasta

./group407/reference/d1jz8a4-d1nsza_.fasta

./group407/reference/d1n7oa3-d1nsza_.fasta

./group408/reference/d1a02n1-d1k3ia1.fasta

./group408/reference/d1a02n1-d1l9na1.fasta

./group408/reference/d1a02n1-d1m7xa1.fasta

./group408/reference/d1a02n1-d1nepa_.fasta

./group408/reference/d1a02n1-d1qfha1.fasta

./group408/reference/d1bf2_1-d1clc_2.fasta

./group408/reference/d1bf2_1-d1ea9c1.fasta

./group408/reference/d1bf2_1-d1eut_1.fasta

./group408/reference/d1bf2_1-d1g0da1.fasta

./group408/reference/d1bf2_1-d1j0ha1.fasta

./group408/reference/d1bf2_1-d1ji1a1.fasta

./group408/reference/d1bf2_1-d1ji2a1.fasta

./group408/reference/d1bf2_1-d1jmxa4.fasta

./group408/reference/d1bf2_1-d1k3ia1.fasta

./group408/reference/d1bf2_1-d1l9na1.fasta

./group408/reference/d1bf2_1-d1my7a_.fasta

./group408/reference/d1bf2_1-d1qfha1.fasta

./group408/reference/d1bf2_1-d1qfha2.fasta

./group408/reference/d1bf2_1-d1soxa1.fasta

./group408/reference/d1clc_2-d1edqa1.fasta

./group408/reference/d1clc_2-d1g0da1.fasta

./group408/reference/d1clc_2-d1k3ia1.fasta

./group408/reference/d1clc_2-d1kmta_.fasta

./group408/reference/d1clc_2-d1l9na1.fasta

./group408/reference/d1clc_2-d1qba_1.fasta

./group408/reference/d1clc_2-d1qfha1.fasta

./group408/reference/d1clc_2-d1qfha2.fasta

./group408/reference/d1ea9c1-d1eut_1.fasta

./group408/reference/d1ea9c1-d1m7xa1.fasta

./group408/reference/d1ea9c1-d1my7a_.fasta

./group408/reference/d1edqa1-d1eut_1.fasta

./group408/reference/d1edqa1-d1g0da1.fasta

./group408/reference/d1edqa1-d1g4ma1.fasta

./group408/reference/d1edqa1-d1imhc1.fasta

./group408/reference/d1edqa1-d1j0ha1.fasta

./group408/reference/d1edqa1-d1ji2a1.fasta

./group408/reference/d1edqa1-d1jjua4.fasta

./group408/reference/d1edqa1-d1jmxa4.fasta

./group408/reference/d1edqa1-d1k3ia1.fasta

./group408/reference/d1edqa1-d1ktja_.fasta

./group408/reference/d1edqa1-d1l9na1.fasta

./group408/reference/d1edqa1-d1nepa_.fasta

./group408/reference/d1edqa1-d1qba_1.fasta

./group408/reference/d1edqa1-d1qfha1.fasta

./group408/reference/d1edqa1-d1qfha2.fasta

./group408/reference/d1eut_1-d1g0da1.fasta

./group408/reference/d1eut_1-d1imhc1.fasta

./group408/reference/d1eut_1-d1j0ha1.fasta

./group408/reference/d1eut_1-d1ji1a1.fasta

./group408/reference/d1eut_1-d1jjua4.fasta

./group408/reference/d1eut_1-d1jmxa4.fasta

./group408/reference/d1eut_1-d1kmta_.fasta

./group408/reference/d1eut_1-d1l9na1.fasta

./group408/reference/d1eut_1-d1nepa_.fasta

./group408/reference/d1eut_1-d1qfha1.fasta

./group408/reference/d1eut_1-d1soxa1.fasta

./group408/reference/d1g0da1-d1k3ia1.fasta

./group408/reference/d1g0da1-d1kmta_.fasta

./group408/reference/d1g0da1-d1m7xa1.fasta

./group408/reference/d1g0da1-d1my7a_.fasta

./group408/reference/d1g0da1-d1nepa_.fasta

./group408/reference/d1g0da1-d1qfha1.fasta

./group408/reference/d1g4ma1-d1ji2a1.fasta

./group408/reference/d1g4ma1-d1my7a_.fasta

./group408/reference/d1g4ma1-d1qfha1.fasta

./group408/reference/d1imhc1-d1j0ha1.fasta

./group408/reference/d1imhc1-d1ji2a1.fasta

./group408/reference/d1imhc1-d1k3ia1.fasta

./group408/reference/d1imhc1-d1kmta_.fasta

./group408/reference/d1imhc1-d1ktja_.fasta

./group408/reference/d1imhc1-d1l9na1.fasta

./group408/reference/d1imhc1-d1nepa_.fasta

./group408/reference/d1imhc1-d1qba_1.fasta

./group408/reference/d1j0ha1-d1k3ia1.fasta

./group408/reference/d1j0ha1-d1l9na1.fasta

./group408/reference/d1j0ha1-d1m7xa1.fasta

./group408/reference/d1j0ha1-d1qba_1.fasta

./group408/reference/d1j0ha1-d1soxa1.fasta

./group408/reference/d1ji1a1-d1ji2a1.fasta

./group408/reference/d1ji1a1-d1ktja_.fasta

./group408/reference/d1ji1a1-d1l9na1.fasta

./group408/reference/d1ji1a1-d1m7xa1.fasta

./group408/reference/d1ji2a1-d1jmxa4.fasta

./group408/reference/d1ji2a1-d1ktja_.fasta

./group408/reference/d1ji2a1-d1l9na1.fasta

./group408/reference/d1ji2a1-d1m7xa1.fasta

./group408/reference/d1ji2a1-d1my7a_.fasta

./group408/reference/d1ji2a1-d1qba_1.fasta

./group408/reference/d1ji2a1-d1qfha2.fasta

./group408/reference/d1ji2a1-d1soxa1.fasta

./group408/reference/d1jjua4-d1k3ia1.fasta

./group408/reference/d1jjua4-d1kmta_.fasta

./group408/reference/d1jjua4-d1l9na1.fasta

./group408/reference/d1jjua4-d1qfha1.fasta

./group408/reference/d1jmxa4-d1kmta_.fasta

./group408/reference/d1jmxa4-d1ktja_.fasta

./group408/reference/d1jmxa4-d1m7xa1.fasta

./group408/reference/d1jmxa4-d1qfha1.fasta

./group408/reference/d1jmxa4-d1qfha2.fasta

./group408/reference/d1k3ia1-d1kmta_.fasta

./group408/reference/d1k3ia1-d1m7xa1.fasta

./group408/reference/d1k3ia1-d1my7a_.fasta

./group408/reference/d1k3ia1-d1nepa_.fasta

./group408/reference/d1k3ia1-d1qba_1.fasta

./group408/reference/d1k3ia1-d1qfha1.fasta

./group408/reference/d1k3ia1-d1qfha2.fasta

./group408/reference/d1kmta_-d1ktja_.fasta

./group408/reference/d1kmta_-d1nepa_.fasta

./group408/reference/d1kmta_-d1qba_1.fasta

./group408/reference/d1ktja_-d1qba_1.fasta

./group408/reference/d1ktja_-d1qfha1.fasta

./group408/reference/d1ktja_-d1qfha2.fasta

./group408/reference/d1l9na1-d1m7xa1.fasta

./group408/reference/d1l9na1-d1my7a_.fasta

./group408/reference/d1l9na1-d1nepa_.fasta

./group408/reference/d1l9na1-d1qfha2.fasta

./group408/reference/d1m7xa1-d1nepa_.fasta

./group408/reference/d1m7xa1-d1qfha2.fasta

./group408/reference/d1my7a_-d1nepa_.fasta

./group408/reference/d1my7a_-d1qba_1.fasta

./group408/reference/d1my7a_-d1qfha2.fasta

./group408/reference/d1nepa_-d1qba_1.fasta

./group408/reference/d1qba_1-d1qfha1.fasta

./group408/reference/d1qba_1-d1soxa1.fasta

./group409/reference/d1f5aa4-d1jaja_.fasta

./group409/reference/d1f5aa4-d1knya2.fasta

./group409/reference/d1fa0a4-d1jaja_.fasta

./group409/reference/d1fa0a4-d1knya2.fasta

./group409/reference/d1jaja_-d1knya2.fasta

./group411/reference/d1k4cc_-d1orsc_.fasta

./group411/reference/d1orsc_-d1p7ba2.fasta

./group412/reference/d1kf6c_-d1kf6d_.fasta

./group412/reference/d1kf6c_-d1nekc_.fasta

./group412/reference/d1kf6c_-d1nekd_.fasta

./group412/reference/d1kf6d_-d1nekc_.fasta

./group412/reference/d1kf6d_-d1nekd_.fasta

./group412/reference/d1kf6d_-d1qlac_.fasta

./group412/reference/d1nekc_-d1nekd_.fasta

./group412/reference/d1nekc_-d1qlac_.fasta

./group414/reference/d1fftb2-d1m56b2.fasta

./group414/reference/d1fftb2-d1ocrb2.fasta

./group416/reference/d1k3xa2-d1k82a2.fasta

./group419/reference/d1aym1_-d1aym3_.fasta

./group419/reference/d1aym1_-d1b35b_.fasta

./group419/reference/d1aym1_-d1bev3_.fasta

./group419/reference/d1aym1_-d1c8na_.fasta

./group419/reference/d1aym1_-d1f2na_.fasta

./group419/reference/d1aym1_-d1ihma_.fasta

./group419/reference/d1aym1_-d1opoa_.fasta

./group419/reference/d1aym1_-d1pvc3_.fasta

./group419/reference/d1aym1_-d1qqp3_.fasta

./group419/reference/d1aym1_-d1smva_.fasta

./group419/reference/d1aym1_-d1tmf1_.fasta

./group419/reference/d1aym1_-d2mev3_.fasta

./group419/reference/d1aym3_-d1b35a_.fasta

./group419/reference/d1aym3_-d1b35b_.fasta

./group419/reference/d1aym3_-d1bev1_.fasta

./group419/reference/d1aym3_-d1bmv11.fasta

./group419/reference/d1aym3_-d1c8na_.fasta

./group419/reference/d1aym3_-d1d4m1_.fasta

./group419/reference/d1aym3_-d1ihma_.fasta

./group419/reference/d1aym3_-d1k5ma_.fasta

./group419/reference/d1aym3_-d1ny722.fasta

./group419/reference/d1aym3_-d1opoa_.fasta

./group419/reference/d1aym3_-d1pvc1_.fasta

./group419/reference/d1aym3_-d1smva_.fasta

./group419/reference/d1aym3_-d2mev1_.fasta

./group419/reference/d1b35a_-d1b35b_.fasta

./group419/reference/d1b35a_-d1bev1_.fasta

./group419/reference/d1b35a_-d1bev3_.fasta

./group419/reference/d1b35a_-d1bmv11.fasta

./group419/reference/d1b35a_-d1c8na_.fasta

./group419/reference/d1b35a_-d1ddla_.fasta

./group419/reference/d1b35a_-d1ihma_.fasta

./group419/reference/d1b35a_-d1ng0a_.fasta

./group419/reference/d1b35a_-d1ny711.fasta

./group419/reference/d1b35a_-d1pvc3_.fasta

./group419/reference/d1b35a_-d1tmf1_.fasta

./group419/reference/d1b35a_-d2mev1_.fasta

./group419/reference/d1b35a_-d2mev3_.fasta

./group419/reference/d1b35b_-d1bev1_.fasta

./group419/reference/d1b35b_-d1bev3_.fasta

./group419/reference/d1b35b_-d1bmv11.fasta

./group419/reference/d1b35b_-d1d4m1_.fasta

./group419/reference/d1b35b_-d1ihma_.fasta

./group419/reference/d1b35b_-d1k5ma_.fasta

./group419/reference/d1b35b_-d1opoa_.fasta

./group419/reference/d1b35b_-d1pvc1_.fasta

./group419/reference/d1b35b_-d1pvc3_.fasta

./group419/reference/d1b35b_-d1qqp3_.fasta

./group419/reference/d1b35b_-d1tmf1_.fasta

./group419/reference/d1b35b_-d2mev1_.fasta

./group419/reference/d1b35b_-d2mev3_.fasta

./group419/reference/d1bev1_-d1bev3_.fasta

./group419/reference/d1bev1_-d1c8na_.fasta

./group419/reference/d1bev1_-d1ihma_.fasta

./group419/reference/d1bev1_-d1ny722.fasta

./group419/reference/d1bev1_-d1opoa_.fasta

./group419/reference/d1bev1_-d1pvc3_.fasta

./group419/reference/d1bev1_-d1qqp3_.fasta

./group419/reference/d1bev1_-d1smva_.fasta

./group419/reference/d1bev1_-d2mev3_.fasta

./group419/reference/d1bev3_-d1bmv11.fasta

./group419/reference/d1bev3_-d1c8na_.fasta

./group419/reference/d1bev3_-d1d4m1_.fasta

./group419/reference/d1bev3_-d1f2na_.fasta

./group419/reference/d1bev3_-d1k5ma_.fasta

./group419/reference/d1bev3_-d1ng0a_.fasta

./group419/reference/d1bev3_-d1ny711.fasta

./group419/reference/d1bev3_-d1opoa_.fasta

./group419/reference/d1bev3_-d1pvc1_.fasta

./group419/reference/d1bev3_-d1smva_.fasta

./group419/reference/d1bev3_-d1tmf1_.fasta

./group419/reference/d1bev3_-d2mev1_.fasta

./group419/reference/d1bmv11-d1f2na_.fasta

./group419/reference/d1bmv11-d1ihma_.fasta

./group419/reference/d1bmv11-d1ny722.fasta

./group419/reference/d1bmv11-d1qqp3_.fasta

./group419/reference/d1bmv11-d1tmf1_.fasta

./group419/reference/d1bmv11-d2mev1_.fasta

./group419/reference/d1bmv11-d2mev3_.fasta

./group419/reference/d1c8na_-d1d4m1_.fasta

./group419/reference/d1c8na_-d1ddla_.fasta

./group419/reference/d1c8na_-d1ihma_.fasta

./group419/reference/d1c8na_-d1ny722.fasta

./group419/reference/d1c8na_-d1pvc1_.fasta

./group419/reference/d1c8na_-d1pvc3_.fasta

./group419/reference/d1c8na_-d1tmf1_.fasta

./group419/reference/d1c8na_-d2mev1_.fasta

./group419/reference/d1c8na_-d2mev3_.fasta

./group419/reference/d1d4m1_-d1opoa_.fasta

./group419/reference/d1d4m1_-d1pvc3_.fasta

./group419/reference/d1d4m1_-d1qqp3_.fasta

./group419/reference/d1d4m1_-d1tmf1_.fasta

./group419/reference/d1d4m1_-d2bbva_.fasta

./group419/reference/d1d4m1_-d2mev1_.fasta

./group419/reference/d1d4m1_-d2mev3_.fasta

./group419/reference/d1ddla_-d1f2na_.fasta

./group419/reference/d1ddla_-d1ihma_.fasta

./group419/reference/d1ddla_-d1opoa_.fasta

./group419/reference/d1ddla_-d1smva_.fasta

./group419/reference/d1ddla_-d1tmf1_.fasta

./group419/reference/d1ddla_-d2bbva_.fasta

./group419/reference/d1f2na_-d1ihma_.fasta

./group419/reference/d1f2na_-d1ny711.fasta

./group419/reference/d1f2na_-d1opoa_.fasta

./group419/reference/d1f2na_-d1pvc3_.fasta

./group419/reference/d1f2na_-d1qqp3_.fasta

./group419/reference/d1f2na_-d2bbva_.fasta

./group419/reference/d1f2na_-d2mev1_.fasta

./group419/reference/d1f2na_-d2mev3_.fasta

./group419/reference/d1ihma_-d1k5ma_.fasta

./group419/reference/d1ihma_-d1ng0a_.fasta

./group419/reference/d1ihma_-d1ny711.fasta

./group419/reference/d1ihma_-d1ny722.fasta

./group419/reference/d1ihma_-d1opoa_.fasta

./group419/reference/d1ihma_-d1pvc1_.fasta

./group419/reference/d1ihma_-d1qqp3_.fasta

./group419/reference/d1ihma_-d1smva_.fasta

./group419/reference/d1ihma_-d1tmf1_.fasta

./group419/reference/d1ihma_-d2bbva_.fasta

./group419/reference/d1ihma_-d2mev1_.fasta

./group419/reference/d1ihma_-d2mev3_.fasta

./group419/reference/d1k5ma_-d1opoa_.fasta

./group419/reference/d1k5ma_-d1pvc3_.fasta

./group419/reference/d1k5ma_-d1qqp3_.fasta

./group419/reference/d1k5ma_-d1smva_.fasta

./group419/reference/d1k5ma_-d1tmf1_.fasta

./group419/reference/d1k5ma_-d2mev3_.fasta

./group419/reference/d1ng0a_-d1ny711.fasta

./group419/reference/d1ng0a_-d1pvc3_.fasta

./group419/reference/d1ng0a_-d1qqp3_.fasta

./group419/reference/d1ng0a_-d2bbva_.fasta

./group419/reference/d1ng0a_-d2mev1_.fasta

./group419/reference/d1ng0a_-d2mev3_.fasta

./group419/reference/d1ny711-d1qqp3_.fasta

./group419/reference/d1ny711-d2mev1_.fasta

./group419/reference/d1ny722-d1pvc3_.fasta

./group419/reference/d1ny722-d1qqp3_.fasta

./group419/reference/d1ny722-d1tmf1_.fasta

./group419/reference/d1ny722-d2mev1_.fasta

./group419/reference/d1opoa_-d1pvc1_.fasta

./group419/reference/d1opoa_-d1pvc3_.fasta

./group419/reference/d1opoa_-d1qqp3_.fasta

./group419/reference/d1opoa_-d1tmf1_.fasta

./group419/reference/d1opoa_-d2mev3_.fasta

./group419/reference/d1pvc1_-d1pvc3_.fasta

./group419/reference/d1pvc1_-d1qqp3_.fasta

./group419/reference/d1pvc1_-d1smva_.fasta

./group419/reference/d1pvc1_-d1tmf1_.fasta

./group419/reference/d1pvc1_-d2mev3_.fasta

./group419/reference/d1pvc3_-d2bbva_.fasta

./group419/reference/d1pvc3_-d2mev1_.fasta

./group419/reference/d1qqp3_-d1tmf1_.fasta

./group419/reference/d1smva_-d2bbva_.fasta

./group419/reference/d1smva_-d2mev1_.fasta

./group419/reference/d1smva_-d2mev3_.fasta

./group419/reference/d1tmf1_-d2mev3_.fasta

./group419/reference/d2bbva_-d2mev3_.fasta

./group419/reference/d2mev1_-d2mev3_.fasta

./group420/reference/d1dnv__-d1lp3a_.fasta

./group420/reference/d1gff2_-d1lp3a_.fasta

./group421/reference/d1dzla_-d1vpsa_.fasta

./group423/reference/d1iw7f1-d1ku3a_.fasta

./group423/reference/d1ku3a_-d1l0oc_.fasta

./group423/reference/d1l0oc_-d1or7a1.fasta

./group424/reference/d1g8fa1-d1jhda1.fasta

./group424/reference/d1iq8a3-d1jhda1.fasta
