# Supplementary material for: PR2ALIGN: a stand-alone software program and a web-server for protein sequence alignment using weighted biochemical properties of amino acids
Source: BMC Res Notes. 2015 May 7;8:187. doi: 10.1186/s13104-015-1152-6 (PMC4477417; doi:10.1186/s13104-015-1152-6)
Supplement: Additional file 10: — All SABmark SUP sequence pairs for 40-50% sequence identity range. [file 13104_2015_1152_MOESM10_ESM.docx]

All SABmark SUP sequence pairs for 40-50% sequence identity range.

./group1/reference/d1b8da_-d1jboa_.fasta

./group1/reference/d1cg5a_-d1gcva_.fasta

./group1/reference/d1cg5a_-d1irda_.fasta

./group1/reference/d1cg5a_-d1la6a_.fasta

./group1/reference/d1cqxa1-d1gvha1.fasta

./group1/reference/d1d8ua_-d2gdm__.fasta

./group1/reference/d1gcva_-d1irda_.fasta

./group1/reference/d1irda_-d1irdb_.fasta

./group1/reference/d1irda_-d1la6a_.fasta

./group1/reference/d1irdb_-d1la6a_.fasta

./group1/reference/d1it2a_-d2lhb__.fasta

./group5/reference/d1ap6a1-d1ix9a1.fasta

./group5/reference/d1b06a1-d1bsma1.fasta

./group5/reference/d1ix9a1-d1qnna1.fasta

./group6/reference/d1co6a_-d1cot__.fasta

./group6/reference/d1co6a_-d1hroa_.fasta

./group6/reference/d1co6a_-d1ql3a_.fasta

./group6/reference/d1co6a_-d1qn2a_.fasta

./group6/reference/d1co6a_-d1ycc__.fasta

./group6/reference/d1cot__-d1cxc__.fasta

./group6/reference/d1cot__-d1ql3a_.fasta

./group6/reference/d1cot__-d1qn2a_.fasta

./group6/reference/d1cot__-d3c2c__.fasta

./group6/reference/d1cxc__-d3c2c__.fasta

./group6/reference/d1e29a_-d1f1ca_.fasta

./group6/reference/d1eb7a1-d1iqca1.fasta

./group6/reference/d1hroa_-d1ql3a_.fasta

./group6/reference/d1hroa_-d1qn2a_.fasta

./group6/reference/d1hroa_-d1ycc__.fasta

./group6/reference/d1ql3a_-d1qn2a_.fasta

./group6/reference/d1ql3a_-d3c2c__.fasta

./group6/reference/d1qn2a_-d3c2c__.fasta

./group7/reference/d1bl0a2-d1d5ya2.fasta

./group7/reference/d1fjla_-d1ig7a_.fasta

./group7/reference/d1fjla_-d1jgga_.fasta

./group7/reference/d1ig7a_-d1jgga_.fasta

./group7/reference/d1jgga_-d1qrya_.fasta

./group8/reference/d1eh6a1-d1sfe_1.fasta

./group10/reference/d1d5va_-d1e17a_.fasta

./group10/reference/d1hks__-d2hts__.fasta

./group10/reference/d1md0a_-d1puee_.fasta

./group13/reference/d1k82a1-d1l1za1.fasta

./group14/reference/d1g4da_-d1tns__.fasta

./group17/reference/d1gab__-d1gjta_.fasta

./group18/reference/d1erp__-d2erl__.fasta

./group21/reference/d1hsm__-d1lwma_.fasta

./group21/reference/d1hsm__-d1qrva_.fasta

./group21/reference/d1lwma_-d1qrva_.fasta

./group22/reference/d1b67a_-d1n1ja_.fasta

./group23/reference/d1gqaa_-d1jafa_.fasta

./group24/reference/d1ei7a_-d1rmva_.fasta

./group25/reference/d1jqia1-d3mdda1.fasta

./group26/reference/d1jiga_-d1qgha_.fasta

./group32/reference/d1eqfa1-d1eqfa2.fasta

./group33/reference/d1efaa1-d1uxd__.fasta

./group34/reference/d1nkpa_-d1nkpb_.fasta

./group35/reference/d1auib_-d1exra_.fasta

./group35/reference/d1rro__-d2pvba_.fasta

./group35/reference/d1rro__-d5pal__.fasta

./group36/reference/d1bhda_-d1bkra_.fasta

./group36/reference/d1bhda_-d1mb8a2.fasta

./group37/reference/d1baza_-d1mnta_.fasta

./group38/reference/d1a0fa1-d1pmt_1.fasta

./group38/reference/d1aw9_1-d1axda1.fasta

./group38/reference/d1aw9_1-d1gnwa1.fasta

./group38/reference/d1gula1-d1k3ya1.fasta

./group39/reference/d1brwa1-d2tpt_1.fasta

./group43/reference/d1fo4a1-d1hlra1.fasta

./group43/reference/d1fo4a1-d1n62a1.fasta

./group44/reference/d1b0xa_-d1b4fa_.fasta

./group49/reference/d1axn__-d1bo9a_.fasta

./group49/reference/d1axn__-d1dm5a_.fasta

./group49/reference/d1axn__-d1g5na_.fasta

./group49/reference/d1axn__-d1hm6a_.fasta

./group49/reference/d1bo9a_-d1dm5a_.fasta

./group49/reference/d1bo9a_-d1g5na_.fasta

./group49/reference/d1dm5a_-d1g5na_.fasta

./group49/reference/d1dm5a_-d1hm6a_.fasta

./group49/reference/d1g5na_-d1hm6a_.fasta

./group52/reference/d1bu2a1-d1g3nc1.fasta

./group56/reference/d1agre_-d1cmza_.fasta

./group57/reference/d1aru__-d1llp__.fasta

./group57/reference/d1aru__-d1mn2__.fasta

./group57/reference/d1jdra_-d1oafa_.fasta

./group57/reference/d1llp__-d1mn2__.fasta

./group58/reference/d1orna_-d2abk__.fasta

./group59/reference/d1dnpa1-d1iqra1.fasta

./group59/reference/d1dnpa1-d1np7a1.fasta

./group59/reference/d1iqra1-d1qnf_1.fasta

./group61/reference/d1ayx__-d1gai__.fasta

./group64/reference/d1a59__-d1aj8a_.fasta

./group64/reference/d1a59__-d1ioma_.fasta

./group64/reference/d1aj8a_-d1ioma_.fasta

./group64/reference/d1aj8a_-d1o7xa_.fasta

./group64/reference/d1ioma_-d1o7xa_.fasta

./group69/reference/d1bd8__-d1ihba_.fasta

./group71/reference/d1dvpa1-d1elka_.fasta

./group75/reference/d1fura_-d1j3ua_.fasta

./group75/reference/d1fura_-d1jswa_.fasta

./group76/reference/d1aokb_-d1buna_.fasta

./group76/reference/d1aokb_-d1g4ia_.fasta

./group76/reference/d1aokb_-d1lfja_.fasta

./group76/reference/d1aokb_-d1mc2a_.fasta

./group76/reference/d1aokb_-d1poc__.fasta

./group76/reference/d1buna_-d1mc2a_.fasta

./group76/reference/d1buna_-d1poc__.fasta

./group76/reference/d1buna_-d1psj__.fasta

./group76/reference/d1g4ia_-d1kvoa_.fasta

./group76/reference/d1g4ia_-d1mc2a_.fasta

./group76/reference/d1g4ia_-d1poc__.fasta

./group76/reference/d1g4ia_-d1psj__.fasta

./group76/reference/d1kvoa_-d1mc2a_.fasta

./group76/reference/d1kvoa_-d1poc__.fasta

./group76/reference/d1kvoa_-d1psj__.fasta

./group76/reference/d1le6a_-d1poc__.fasta

./group76/reference/d1lfja_-d1poc__.fasta

./group76/reference/d1mc2a_-d1poc__.fasta

./group76/reference/d1poc__-d1psj__.fasta

./group77/reference/d19hca_-d3caoa_.fasta

./group77/reference/d1aqe__-d2cy3__.fasta

./group77/reference/d1aqe__-d3caoa_.fasta

./group77/reference/d1fgja_-d1m1qa_.fasta

./group77/reference/d1fgja_-d1qo8a1.fasta

./group77/reference/d1ft5a_-d1m1qa_.fasta

./group77/reference/d1gyoa_-d2cy3__.fasta

./group77/reference/d1hh5a_-d1m1qa_.fasta

./group77/reference/d1hh5a_-d1wad__.fasta

./group77/reference/d1hh5a_-d3caoa_.fasta

./group77/reference/d1kssa1-d1qo8a1.fasta

./group77/reference/d1wad__-d2cy3__.fasta

./group77/reference/d1wad__-d3cyr__.fasta

./group77/reference/d2ctha_-d3cyr__.fasta

./group77/reference/d2cy3__-d3cyr__.fasta

./group78/reference/d1akjd_-d1nezg_.fasta

./group78/reference/d1b88a_-d1h5ba_.fasta

./group78/reference/d1b88a_-d1ogad1.fasta

./group78/reference/d1g9mh1-d1mfa_2.fasta

./group78/reference/d1g9mh1-d1mqkh_.fasta

./group78/reference/d1g9mh1-d1ncwh1.fasta

./group78/reference/d1h5ba_-d1ogad1.fasta

./group78/reference/d1mfa_2-d1mqkh_.fasta

./group78/reference/d1mfa_2-d1nlbh1.fasta

./group78/reference/d1mqkh_-d1ncwh1.fasta

./group78/reference/d1mqkh_-d1nlbh1.fasta

./group78/reference/d1mqkh_-d2f5bh1.fasta

./group78/reference/d1mqkh_-d2rhe__.fasta

./group78/reference/d1ncwh1-d1nlbh1.fasta

./group78/reference/d1nfdb1-d2rhe__.fasta

./group79/reference/d1bqua1-d1cd9b1.fasta

./group81/reference/d1g0da3-d1kv3a3.fasta

./group83/reference/d1akp__-d1j48a_.fasta

./group83/reference/d1j48a_-d1noa__.fasta

./group83/reference/d1noa__-d2mcm__.fasta

./group87/reference/d1g43a_-d1nbca_.fasta

./group89/reference/d1a3qa2-d1bvoa_.fasta

./group90/reference/d1acz__-d1pama2.fasta

./group90/reference/d1j18a1-d1qhoa2.fasta

./group90/reference/d1pama2-d1qhoa2.fasta

./group92/reference/d1fwxa1-d1kbva1.fasta

./group92/reference/d1kcw_1-d1kcw_5.fasta

./group92/reference/d1kcw_2-d1kcw_4.fasta

./group92/reference/d1kcw_2-d1kcw_6.fasta

./group92/reference/d1m56b1-d1ocrb1.fasta

./group93/reference/d1k5wa_-d3rpba_.fasta

./group95/reference/d1h4ax1-d1h4ax2.fasta

./group95/reference/d1h4ax1-d1ha4a_.fasta

./group95/reference/d1h4ax1-d2bb2_2.fasta

./group95/reference/d1ha4a_-d2bb2_2.fasta

./group99/reference/d1ciy_1-d1dlc_1.fasta

./group99/reference/d1czsa_-d1d7pm_.fasta

./group99/reference/d1czsa_-d1kexa_.fasta

./group99/reference/d1d7pm_-d1kexa_.fasta

./group100/reference/d1jsda_-d1jsma_.fasta

./group100/reference/d1jsma_-d2viua_.fasta

./group101/reference/d1h7za_-d1kaca_.fasta

./group101/reference/d1h7za_-d1qhva_.fasta

./group101/reference/d1kaca_-d1qhva_.fasta

./group102/reference/d1tnra_-d2tnfa_.fasta

./group105/reference/d1a8d_1-d3btaa1.fasta

./group105/reference/d1bk1__-d1xnb__.fasta

./group105/reference/d1fx5a_-d1gzca_.fasta

./group105/reference/d1fx5a_-d1n3oa_.fasta

./group105/reference/d1fx5a_-d1nls__.fasta

./group105/reference/d1fx5a_-d2pela_.fasta

./group105/reference/d1gzca_-d1n3oa_.fasta

./group105/reference/d1gzca_-d1nls__.fasta

./group105/reference/d1gzca_-d2pela_.fasta

./group105/reference/d1n3oa_-d1nls__.fasta

./group105/reference/d1n3oa_-d2pela_.fasta

./group105/reference/d1nls__-d2pela_.fasta

./group108/reference/d1awj__-d1gl5a_.fasta

./group108/reference/d1bbza_-d1fmk_1.fasta

./group108/reference/d1bbza_-d1h92a_.fasta

./group108/reference/d1bbza_-d1qcfa1.fasta

./group108/reference/d1fmk_1-d1gl5a_.fasta

./group108/reference/d1fmk_1-d1h92a_.fasta

./group108/reference/d1fmk_1-d1oeba_.fasta

./group108/reference/d1gcqa_-d1jo8a_.fasta

./group108/reference/d1gcqa_-d1oeba_.fasta

./group108/reference/d1gcqa_-d2hsp__.fasta

./group108/reference/d1gl5a_-d1jqqa_.fasta

./group108/reference/d1gl5a_-d1qcfa1.fasta

./group108/reference/d1i07a_-d1jqqa_.fasta

./group108/reference/d1jo8a_-d1neb__.fasta

./group108/reference/d1jo8a_-d1oeba_.fasta

./group108/reference/d1jo8a_-d2hsp__.fasta

./group108/reference/d1k4us_-d1qcfa1.fasta

./group108/reference/d1k4us_-d2hsp__.fasta

./group108/reference/d1oeba_-d1qcfa1.fasta

./group108/reference/d1qcfa1-d1ycsb2.fasta

./group111/reference/d1aono_-d1p3ha_.fasta

./group112/reference/d1be9a_-d1qava_.fasta

./group112/reference/d1d5ga_-d1qlca_.fasta

./group112/reference/d1qava_-d1qlca_.fasta

./group113/reference/d1h641_-d1i8fa_.fasta

./group113/reference/d1h641_-d1mgqa_.fasta

./group113/reference/d1i8fa_-d1mgqa_.fasta

./group113/reference/d1mgqa_-d1n9ra_.fasta

./group114/reference/d1fnua1-d3seb_1.fasta

./group115/reference/d1br9__-d1ueab_.fasta

./group118/reference/d1guta_-d1h9ma1.fasta

./group118/reference/d1guta_-d1h9ma2.fasta

./group118/reference/d1h9ma1-d1h9ma2.fasta

./group118/reference/d1h9ma1-d1h9ra1.fasta

./group119/reference/d1bfg__-d1ihka_.fasta

./group119/reference/d1bfg__-d1ijta_.fasta

./group119/reference/d1bfg__-d1qqla_.fasta

./group119/reference/d1ihka_-d1ijta_.fasta

./group119/reference/d1ihka_-d1nuna_.fasta

./group119/reference/d1ihka_-d1qqla_.fasta

./group119/reference/d1nuna_-d1qqla_.fasta

./group120/reference/d1abrb2-d1hwmb2.fasta

./group120/reference/d1abrb2-d1m2tb2.fasta

./group120/reference/d1ggpb1-d1hwmb1.fasta

./group120/reference/d1ggpb1-d1m2tb1.fasta

./group121/reference/d1avwb_-d1wba__.fasta

./group123/reference/d1exma1-d1f60a1.fasta

./group124/reference/d1d2ea2-d1exma2.fasta

./group125/reference/d1ci0a_-d1nrga_.fasta

./group126/reference/d1agja_-d1qtfa_.fasta

./group126/reference/d1azza_-d1ltoa_.fasta

./group126/reference/d1azza_-d2hlca_.fasta

./group126/reference/d1bio__-d1cgha_.fasta

./group126/reference/d1bio__-d1mzaa_.fasta

./group126/reference/d1bio__-d1orfa_.fasta

./group126/reference/d1bqya_-d1hj9a_.fasta

./group126/reference/d1cgha_-d1mzaa_.fasta

./group126/reference/d1cgha_-d1orfa_.fasta

./group126/reference/d1ddja_-d1eaxa_.fasta

./group126/reference/d1ddja_-d1hj9a_.fasta

./group126/reference/d1ddja_-d1ltoa_.fasta

./group126/reference/d1eaxa_-d1ekbb_.fasta

./group126/reference/d1eaxa_-d1hj9a_.fasta

./group126/reference/d1eaxa_-d1ltoa_.fasta

./group126/reference/d1eaxa_-d1rfna_.fasta

./group126/reference/d1ekbb_-d1klih_.fasta

./group126/reference/d1fjsa_-d1klih_.fasta

./group126/reference/d1fjsa_-d1rfna_.fasta

./group126/reference/d1gdna_-d1sgt__.fasta

./group126/reference/d1gvkb_-d1hj9a_.fasta

./group126/reference/d1gvkb_-d1ltoa_.fasta

./group126/reference/d1gvza_-d1orfa_.fasta

./group126/reference/d1hj9a_-d1klih_.fasta

./group126/reference/d1hj9a_-d1ltoa_.fasta

./group126/reference/d1hj9a_-d1mzaa_.fasta

./group126/reference/d1hj9a_-d1rfna_.fasta

./group126/reference/d1klih_-d1rfna_.fasta

./group126/reference/d1mzaa_-d1orfa_.fasta

./group126/reference/d1rfna_-d1sgt__.fasta

./group127/reference/d1e79d2-d1fx0b2.fasta

./group130/reference/d1h0ha1-d1kqfa1.fasta

./group131/reference/d1btn__-d1dro__.fasta

./group131/reference/d1eaza_-d1faoa_.fasta

./group132/reference/d1a49a1-d1e0ta1.fasta

./group133/reference/d1b56__-d1hms__.fasta

./group133/reference/d1cbs__-d1hms__.fasta

./group133/reference/d1dzka_-d1g85a_.fasta

./group133/reference/d1ew3a_-d1jv4a_.fasta

./group133/reference/d1ftpa_-d1hms__.fasta

./group133/reference/d1ggla_-d1kqwa_.fasta

./group133/reference/d1o1va_-d1p6pa_.fasta

./group138/reference/d1g94a1-d1hx0a1.fasta

./group138/reference/d1g94a1-d1jae_1.fasta

./group138/reference/d1hx0a1-d1jae_1.fasta

./group139/reference/d1i5hw_-d1jmqa_.fasta

./group139/reference/d1jmqa_-d1pina1.fasta

./group143/reference/d1bn8a_-d1jtaa_.fasta

./group143/reference/d1czfa_-d1hg8a_.fasta

./group143/reference/d1czfa_-d1k5ca_.fasta

./group144/reference/d1kk6a_-d1krra_.fasta

./group144/reference/d1kk6a_-d1ocxa_.fasta

./group144/reference/d1kk6a_-d1xat__.fasta

./group144/reference/d1krra_-d1ocxa_.fasta

./group145/reference/d1fxza2-d1od5a2.fasta

./group147/reference/d1cx4a1-d1rgs_1.fasta

./group149/reference/d1e2wa2-d1hcz_2.fasta

./group150/reference/d1gpr__-d2f3ga_.fasta

./group150/reference/d1gpr__-d2gpr__.fasta

./group152/reference/d1dun__-d1f7da_.fasta

./group153/reference/d1lyxa_-d1n55a_.fasta

./group154/reference/d1dbta_-d1eixa_.fasta

./group155/reference/d1gvoa_-d1oyb__.fasta

./group156/reference/d1hqta_-d1j96a_.fasta

./group157/reference/d1cbg__-d1hxja_.fasta

./group157/reference/d1e4ia_-d1hxja_.fasta

./group157/reference/d1e4ia_-d1ug6a_.fasta

./group157/reference/d1e4mm_-d1hxja_.fasta

./group157/reference/d1j0ha3-d1ji1a3.fasta

./group157/reference/d1pama4-d1qhoa4.fasta

./group159/reference/d1mzha_-d1o0ya_.fasta

./group159/reference/d1mzha_-d1ub3a_.fasta

./group159/reference/d1o0ya_-d1ub3a_.fasta

./group160/reference/d1e9ia1-d1onea1.fasta

./group160/reference/d1muca1-d2chr_1.fasta

./group166/reference/d1gz3a1-d1o0sa1.fasta

./group166/reference/d1hdca_-d1iy8a_.fasta

./group166/reference/d1kepa_-d1kewa_.fasta

./group167/reference/d1gesa2-d3grs_2.fasta

./group167/reference/d1jeha2-d3lada2.fasta

./group167/reference/d1ojt_2-d3lada2.fasta

./group167/reference/d1trb_2-d1vdc_2.fasta

./group168/reference/d1dysa_-d1oc7a_.fasta

./group171/reference/d1m6ba2-d1n8yc2.fasta

./group171/reference/d1m6ba2-d1nqla2.fasta

./group171/reference/d1n8yc2-d1nqla2.fasta

./group174/reference/d1c2ya_-d1hqka_.fasta

./group174/reference/d1c2ya_-d1rvv1_.fasta

./group174/reference/d1ejba_-d1hqka_.fasta

./group174/reference/d1hqka_-d1rvv1_.fasta

./group177/reference/d1ag9a_-d1fuea_.fasta

./group177/reference/d1ag9a_-d1oboa_.fasta

./group177/reference/d1fuea_-d1oboa_.fasta

./group177/reference/d1oboa_-d2fcr__.fasta

./group180/reference/d1gqoa_-d1gtza_.fasta

./group180/reference/d1gqoa_-d1j2ya_.fasta

./group180/reference/d1gtza_-d1j2ya_.fasta

./group182/reference/d1ddga2-d1f20a2.fasta

./group182/reference/d1ddga2-d1ja1a3.fasta

./group184/reference/d1efpa1-d1efva1.fasta

./group186/reference/d1a9xa3-d1a9xa4.fasta

./group186/reference/d1e4ea1-d1ehia1.fasta

./group187/reference/d1efva2-d1o97d2.fasta

./group188/reference/d1fsz_1-d1ofua1.fasta

./group188/reference/d1tuba1-d1tubb1.fasta

./group190/reference/d1ovma2-d1zpda2.fasta

./group191/reference/d1dj3a_-d1qf5a_.fasta

./group191/reference/d1g3qa_-d1hyqa_.fasta

./group191/reference/d1iwea_-d1qf5a_.fasta

./group191/reference/d1jj7a_-d1mt0a_.fasta

./group192/reference/d1ea7a_-d1gci__.fasta

./group192/reference/d1ea7a_-d1thm__.fasta

./group192/reference/d1gci__-d1ic6a_.fasta

./group192/reference/d1ot5a2-d1p8ja2.fasta

./group193/reference/d1d3va_-d2ceva_.fasta

./group194/reference/d1b5sa_-d1eaf__.fasta

./group194/reference/d1nocb_-d3cla__.fasta

./group195/reference/d1d1qa_-d1phr__.fasta

./group196/reference/d1eeoa_-d1lara1.fasta

./group196/reference/d1ikza_-d1mkp__.fasta

./group196/reference/d1ikza_-d1vhra_.fasta

./group196/reference/d1lara1-d1lara2.fasta

./group198/reference/d1ep7a_-d1erv__.fasta

./group198/reference/d1erv__-d1gh2a_.fasta

./group198/reference/d1quwa_-d2trxa_.fasta

./group198/reference/d1thx__-d2trxa_.fasta

./group199/reference/d1gpua3-d1itza3.fasta

./group199/reference/d1gpua3-d1qgda3.fasta

./group200/reference/d1a3wa3-d1a49a3.fasta

./group202/reference/d1hc7a1-d1nj1a1.fasta

./group202/reference/d1hc7a1-d1nj8a1.fasta

./group202/reference/d1nj1a1-d1nj8a1.fasta

./group205/reference/d1bdg_2-d1czan2.fasta

./group205/reference/d1czan3-d1ig8a1.fasta

./group206/reference/d1i39a_-d1io2a_.fasta

./group208/reference/d1b8oa_-d1g2oa_.fasta

./group209/reference/d1kwma1-d1m4la_.fasta

./group212/reference/d1bzya_-d1fsga_.fasta

./group212/reference/d1ecfa1-d1gph11.fasta

./group214/reference/d1f3la_-d1g6q1_.fasta

./group214/reference/d1f3la_-d1oria_.fasta

./group214/reference/d1fp2a2-d1kywa2.fasta

./group214/reference/d1qama_-d1yub__.fasta

./group215/reference/d1ajsa_-d1yaaa_.fasta

./group215/reference/d1ajsa_-d3tata_.fasta

./group215/reference/d1ajsa_-d7aata_.fasta

./group215/reference/d1e5ea_-d1n8pa_.fasta

./group215/reference/d1gdea_-d1j32a_.fasta

./group215/reference/d1gdea_-d1o4sa_.fasta

./group215/reference/d1ibja_-d1n8pa_.fasta

./group215/reference/d1qisa_-d2ay1a_.fasta

./group215/reference/d1qisa_-d3tata_.fasta

./group215/reference/d1yaaa_-d3tata_.fasta

./group215/reference/d1yaaa_-d7aata_.fasta

./group215/reference/d3tata_-d7aata_.fasta

./group216/reference/d1hm9a2-d1hv9a2.fasta

./group217/reference/d1ea5a_-d1mx1a_.fasta

./group218/reference/d1aoea_-d1dyr__.fasta

./group220/reference/d1b7ba_-d1e19a_.fasta

./group223/reference/d1cnza_-d1xaa__.fasta

./group224/reference/d1a1s_2-d1otha2.fasta

./group224/reference/d1duvg1-d1otha1.fasta

./group224/reference/d1ekxa2-d1ml4a2.fasta

./group229/reference/d1a4sa_-d1o04a_.fasta

./group231/reference/d16pk__-d1hdia_.fasta

./group231/reference/d1hdia_-d1php__.fasta

./group235/reference/d1m1nb_-d1miob_.fasta

./group238/reference/d1afwa2-d1m3ka2.fasta

./group238/reference/d1e5ma1-d1ox0a1.fasta

./group238/reference/d1e5ma2-d1ek4a2.fasta

./group238/reference/d1ek4a2-d1ox0a2.fasta

./group238/reference/d1hnja1-d1hzpa1.fasta

./group238/reference/d1hnja1-d1mzja1.fasta

./group238/reference/d1hnja2-d1ub7a2.fasta

./group238/reference/d1hzpa1-d1mzja1.fasta

./group238/reference/d1hzpa2-d1mzja2.fasta

./group238/reference/d1kas_1-d1ox0a1.fasta

./group238/reference/d1mzja2-d1ub7a2.fasta

./group241/reference/d1b9oa_-d2eql__.fasta

./group241/reference/d1gd6a_-d2eql__.fasta

./group241/reference/d1gd6a_-d3lzt__.fasta

./group241/reference/d1k28a3-d1lw9a_.fasta

./group242/reference/d1cvza_-d1fh0a_.fasta

./group242/reference/d1cvza_-d7pcka_.fasta

./group242/reference/d1e2ta_-d1gx3a_.fasta

./group242/reference/d1fh0a_-d1me4a_.fasta

./group242/reference/d1me4a_-d7pcka_.fasta

./group242/reference/d2cb5a_-d3gcb__.fasta

./group245/reference/d1b3aa_-d1g2ta_.fasta

./group245/reference/d1b3aa_-d1j9oa_.fasta

./group245/reference/d1b3aa_-d2hcc__.fasta

./group245/reference/d1doka_-d1eiha_.fasta

./group245/reference/d1doka_-d1f2la_.fasta

./group245/reference/d1eiha_-d1g2ta_.fasta

./group245/reference/d1eiha_-d2hcc__.fasta

./group245/reference/d1el0a_-d2hcc__.fasta

./group245/reference/d1g2ta_-d1j9oa_.fasta

./group245/reference/d1tvxa_-d3il8__.fasta

./group250/reference/d1ei1a1-d1kija1.fasta

./group251/reference/d1a5r__-d1euvb_.fasta

./group252/reference/d1d4ba_-d1f2ri_.fasta

./group254/reference/d1b9ra_-d1e9ma_.fasta

./group254/reference/d1czpa_-d1doi__.fasta

./group254/reference/d1e9ma_-d1i7ha_.fasta

./group254/reference/d1e9ma_-d1put__.fasta

./group254/reference/d1fo4a2-d1hlra2.fasta

./group254/reference/d1fo4a2-d1jroa2.fasta

./group254/reference/d1hlra2-d1n62a2.fasta

./group256/reference/d1enfa2-d1esfa2.fasta

./group256/reference/d1esfa2-d1eu3a2.fasta

./group260/reference/d1gy6a_-d1gy7a_.fasta

./group261/reference/d1c16a2-d1k5na2.fasta

./group262/reference/d1c4zd_-d1qcqa_.fasta

./group262/reference/d1fzya_-d1jata_.fasta

./group262/reference/d1fzya_-d1qcqa_.fasta

./group262/reference/d1i7ka_-d1jata_.fasta

./group262/reference/d1i7ka_-d1qcqa_.fasta

./group262/reference/d1i7ka_-d2aak__.fasta

./group262/reference/d1jata_-d1qcqa_.fasta

./group262/reference/d1qcqa_-d2ucz__.fasta

./group262/reference/d2aak__-d2ucz__.fasta

./group264/reference/d1bkf__-d1jvwa_.fasta

./group264/reference/d1bkf__-d1pbk__.fasta

./group264/reference/d1eq3a_-d1j6ya_.fasta

./group264/reference/d1jvwa_-d1pbk__.fasta

./group265/reference/d1goia3-d1itxa2.fasta

./group271/reference/d1nn7a_-d1t1da_.fasta

./group271/reference/d1nn7a_-d3kvt__.fasta

./group271/reference/d1t1da_-d3kvt__.fasta

./group273/reference/d1b06a2-d1gv3a2.fasta

./group273/reference/d1b06a2-d1isaa2.fasta

./group273/reference/d1b06a2-d1ja8a2.fasta

./group273/reference/d1b06a2-d1kkca2.fasta

./group273/reference/d1b06a2-d1ma1a2.fasta

./group273/reference/d1b06a2-d1qnna2.fasta

./group273/reference/d1bsma2-d1isaa2.fasta

./group273/reference/d1bsma2-d1ix9a2.fasta

./group273/reference/d1bsma2-d1ja8a2.fasta

./group273/reference/d1bsma2-d1kkca2.fasta

./group273/reference/d1bsma2-d1qnna2.fasta

./group273/reference/d1coja2-d1ja8a2.fasta

./group273/reference/d1coja2-d1ma1a2.fasta

./group273/reference/d1gv3a2-d1isaa2.fasta

./group273/reference/d1gv3a2-d1ja8a2.fasta

./group273/reference/d1gv3a2-d1kkca2.fasta

./group273/reference/d1gv3a2-d1ma1a2.fasta

./group273/reference/d1gv3a2-d1qnna2.fasta

./group273/reference/d1isaa2-d1ix9a2.fasta

./group273/reference/d1isaa2-d1ja8a2.fasta

./group273/reference/d1isaa2-d1qnna2.fasta

./group273/reference/d1ix9a2-d1ja8a2.fasta

./group273/reference/d1ix9a2-d1kkca2.fasta

./group273/reference/d1ix9a2-d1ma1a2.fasta

./group273/reference/d1ix9a2-d1qnna2.fasta

./group273/reference/d1ja8a2-d1ma1a2.fasta

./group273/reference/d1kkca2-d1qnna2.fasta

./group273/reference/d1ma1a2-d1qnna2.fasta

./group276/reference/d1hh2p2-d1k0ra2.fasta

./group278/reference/d1fxd__-d1fxra_.fasta

./group278/reference/d1h0hb_-d1kqfb1.fasta

./group278/reference/d1h98a_-d2fdn__.fasta

./group278/reference/d1h98a_-d7fd1a_.fasta

./group281/reference/d1nzaa_-d1p1la_.fasta

./group282/reference/d1ehwa_-d1k44a_.fasta

./group282/reference/d1ehwa_-d1nhkl_.fasta

./group282/reference/d1hlwa_-d1nhkl_.fasta

./group283/reference/d1b7fa2-d1fxla2.fasta

./group283/reference/d1hd1a_-d1iqta_.fasta

./group283/reference/d1hd1a_-d1l3ka1.fasta

./group283/reference/d1hd1a_-d2msta_.fasta

./group283/reference/d1iqta_-d1l3ka2.fasta

./group283/reference/d1iqta_-d2msta_.fasta

./group283/reference/d1l3ka1-d2msta_.fasta

./group283/reference/d1l3ka2-d2msta_.fasta

./group285/reference/d1cc8a_-d1fe0a_.fasta

./group288/reference/d1e6yb2-d1hbnb2.fasta

./group291/reference/d1dd5a_-d1eh1a_.fasta

./group291/reference/d1dd5a_-d1ge9a_.fasta

./group291/reference/d1dd5a_-d1is1a_.fasta

./group291/reference/d1eh1a_-d1ge9a_.fasta

./group291/reference/d1ge9a_-d1is1a_.fasta

./group292/reference/d1bwvs_-d1gk8i_.fasta

./group292/reference/d1bwvs_-d1rblm_.fasta

./group292/reference/d1gk8i_-d1rblm_.fasta

./group294/reference/d1dbfa_-d1ufya_.fasta

./group294/reference/d1jd1a_-d1onia_.fasta

./group294/reference/d1jd1a_-d1qd9a_.fasta

./group294/reference/d1onia_-d1qd9a_.fasta

./group297/reference/d1gd0a_-d1hfoa_.fasta

./group298/reference/d1b7go2-d1cf2o2.fasta

./group299/reference/d1dxla3-d1ebda3.fasta

./group299/reference/d1dxla3-d3lada3.fasta

./group299/reference/d1ebda3-d3lada3.fasta

./group299/reference/d1feca3-d3grs_3.fasta

./group299/reference/d1h6va3-d3grs_3.fasta

./group299/reference/d1lvl_3-d3lada3.fasta

./group299/reference/d1ojt_3-d3lada3.fasta

./group305/reference/d1a09a_-d1a81a2.fasta

./group305/reference/d1a09a_-d1m61a_.fasta

./group305/reference/d1a81a2-d1opka2.fasta

./group305/reference/d1ayaa_-d1jyra_.fasta

./group305/reference/d1ju5a_-d1jwoa_.fasta

./group305/reference/d1ju5a_-d1mil__.fasta

./group305/reference/d1jyra_-d1lkka_.fasta

./group306/reference/d1k1ca_-d1opd__.fasta

./group306/reference/d1k1ca_-d1ptf__.fasta

./group309/reference/d1b8aa2-d1eova2.fasta

./group311/reference/d1cfya_-d1f7sa_.fasta

./group311/reference/d1d0na4-d1d4xg_.fasta

./group312/reference/d1acf__-d1ypra_.fasta

./group317/reference/d1f28a_-d1tis__.fasta

./group319/reference/d1ei1a2-d1kija2.fasta

./group320/reference/d1dixa_-d1ucaa_.fasta

./group321/reference/d1bwda_-d1jdw__.fasta

./group322/reference/d1c22a_-d1o0xa_.fasta

./group323/reference/d1m15a2-d1qh4a2.fasta

./group324/reference/d1aisa1-d1ytba1.fasta

./group324/reference/d1aisa1-d1ytba2.fasta

./group324/reference/d1aisa2-d1ytba1.fasta

./group324/reference/d1aisa2-d1ytba2.fasta

./group326/reference/d1fm4a_-d1icxa_.fasta

./group327/reference/d1mxa_2-d1qm4a2.fasta

./group333/reference/d1k2pa_-d1opja_.fasta

./group333/reference/d1koba_-d1tkia_.fasta

./group333/reference/d1opja_-d1p4oa_.fasta

./group335/reference/d1f7la_-d1ftha_.fasta

./group336/reference/d1ecfa2-d1gph12.fasta

./group336/reference/d1iru1_-d1ryp1_.fasta

./group336/reference/d1iru2_-d1ryp2_.fasta

./group336/reference/d1iruk_-d1rypk_.fasta

./group336/reference/d1j2pa_-d1rypd_.fasta

./group336/reference/d1j2pa_-d1rype_.fasta

./group336/reference/d1pmaa_-d1rypb_.fasta

./group336/reference/d1pmaa_-d1rype_.fasta

./group339/reference/d1a5z_2-d1ldna2.fasta

./group339/reference/d1a5z_2-d1llda2.fasta

./group339/reference/d1b8pa2-d5mdha2.fasta

./group339/reference/d1ceqa2-d1guya2.fasta

./group339/reference/d1ez4a2-d1ldna2.fasta

./group339/reference/d1i0za2-d1llda2.fasta

./group339/reference/d1ldna2-d1llda2.fasta

./group339/reference/d5mdha2-d7mdha2.fasta

./group340/reference/d1abra_-d1hwma_.fasta

./group340/reference/d1abra_-d1ift__.fasta

./group340/reference/d1abra_-d1m2ta_.fasta

./group341/reference/d1giqa2-d1qs1a2.fasta

./group342/reference/d1chua3-d1kf6a3.fasta

./group342/reference/d1chua3-d1qlaa3.fasta

./group342/reference/d1kf6a3-d1qlaa3.fasta

./group342/reference/d1kssa3-d1qo8a3.fasta

./group342/reference/d1neka3-d1qlaa3.fasta

./group343/reference/d1b6e__-d1hq8a_.fasta

./group343/reference/d1j34a_-d1jwib_.fasta

./group343/reference/d1li1a1-d1li1a2.fasta

./group344/reference/d1fzcb1-d1jc9a_.fasta

./group344/reference/d1fzda_-d1jc9a_.fasta

./group346/reference/d1bsg__-d1buea_.fasta

./group346/reference/d1bsg__-d1iyoa_.fasta

./group346/reference/d1bsg__-d1m40a_.fasta

./group346/reference/d1bsg__-d1mfoa_.fasta

./group346/reference/d1bsg__-d4blma_.fasta

./group346/reference/d1buea_-d1iyoa_.fasta

./group346/reference/d1buea_-d4blma_.fasta

./group346/reference/d1g6aa_-d1m40a_.fasta

./group346/reference/d1ghpa_-d4blma_.fasta

./group346/reference/d1iyoa_-d1m40a_.fasta

./group346/reference/d1iyoa_-d1mfoa_.fasta

./group346/reference/d1iyoa_-d4blma_.fasta

./group346/reference/d1m40a_-d1mfoa_.fasta

./group346/reference/d1mfoa_-d4blma_.fasta

./group347/reference/d1a4ea_-d1cf9a2.fasta

./group347/reference/d1a4ea_-d1gwea_.fasta

./group347/reference/d1cf9a2-d1dgfa_.fasta

./group347/reference/d1cf9a2-d1m7sa_.fasta

./group347/reference/d1dgfa_-d1gwea_.fasta

./group347/reference/d1dgfa_-d1m7sa_.fasta

./group347/reference/d1gwea_-d1m7sa_.fasta

./group348/reference/d1buca2-d1ivha2.fasta

./group352/reference/d1qq5a_-d1zrn__.fasta

./group360/reference/d1en2a2-d1hev__.fasta

./group360/reference/d1en2a2-d1mmc__.fasta

./group360/reference/d1hev__-d1mmc__.fasta

./group361/reference/d1agg__-d1lupa_.fasta

./group361/reference/d1axh__-d1g9pa_.fasta

./group361/reference/d1c6wa_-d1d1ha_.fasta

./group361/reference/d1cixa_-d1lupa_.fasta

./group361/reference/d1cixa_-d1nixa_.fasta

./group361/reference/d1d1ha_-d1emxa_.fasta

./group361/reference/d1d1ha_-d1g9pa_.fasta

./group361/reference/d1d1ha_-d1koza_.fasta

./group361/reference/d1eit__-d1kqha_.fasta

./group361/reference/d1emxa_-d1nixa_.fasta

./group361/reference/d1emxa_-d1niya_.fasta

./group361/reference/d1emxa_-d1qk6a_.fasta

./group361/reference/d1g9pa_-d1i26a_.fasta

./group361/reference/d1g9pa_-d1koza_.fasta

./group361/reference/d1g9pa_-d1kqha_.fasta

./group361/reference/d1g9pa_-d1lupa_.fasta

./group361/reference/d1g9pa_-d1qk7a_.fasta

./group361/reference/d1i25a_-d1lupa_.fasta

./group361/reference/d1koza_-d1lqra_.fasta

./group361/reference/d1lupa_-d1nixa_.fasta

./group361/reference/d1lupa_-d1niya_.fasta

./group361/reference/d1nixa_-d1niya_.fasta

./group361/reference/d1nixa_-d1qk7a_.fasta

./group361/reference/d1niya_-d1qk6a_.fasta

./group361/reference/d1niya_-d1qk7a_.fasta

./group361/reference/d1qk6a_-d1qk7a_.fasta

./group362/reference/d1aho__-d1bcg__.fasta

./group362/reference/d1aho__-d1bmr__.fasta

./group362/reference/d1aho__-d2sn3__.fasta

./group362/reference/d1bcg__-d1npia_.fasta

./group362/reference/d1bcg__-d1nrb__.fasta

./group362/reference/d1bmr__-d1i2ua_.fasta

./group362/reference/d1bmr__-d1myn__.fasta

./group362/reference/d1bmr__-d2sn3__.fasta

./group362/reference/d1c55a_-d1ne5a_.fasta

./group362/reference/d1c55a_-d1sco__.fasta

./group362/reference/d1c55a_-d1scy__.fasta

./group362/reference/d1c55a_-d1tsk__.fasta

./group362/reference/d1cmr__-d1gps__.fasta

./group362/reference/d1fjna_-d1i2ua_.fasta

./group362/reference/d1fjna_-d1ica__.fasta

./group362/reference/d1fjna_-d1myn__.fasta

./group362/reference/d1fjna_-d1tsk__.fasta

./group362/reference/d1gps__-d1ne5a_.fasta

./group362/reference/d1i2ua_-d1jxca_.fasta

./group362/reference/d1i2ua_-d1myn__.fasta

./group362/reference/d1i2ua_-d1ne5a_.fasta

./group362/reference/d1i2ua_-d1nrb__.fasta

./group362/reference/d1i2ua_-d1sis__.fasta

./group362/reference/d1i2ua_-d1tsk__.fasta

./group362/reference/d1i2ua_-d2sn3__.fasta

./group362/reference/d1jkza_-d1ne5a_.fasta

./group362/reference/d1jxca_-d1nrb__.fasta

./group362/reference/d1jxca_-d1qkya_.fasta

./group362/reference/d1myn__-d2sn3__.fasta

./group362/reference/d1ne5a_-d1sco__.fasta

./group362/reference/d1ne5a_-d1sis__.fasta

./group362/reference/d1npia_-d1nrb__.fasta

./group362/reference/d1npia_-d2sn3__.fasta

./group362/reference/d1nrb__-d2sn3__.fasta

./group362/reference/d1sco__-d1tsk__.fasta

./group362/reference/d1scy__-d1sis__.fasta

./group363/reference/d1igra3-d1n8yc4.fasta

./group363/reference/d1m6ba3-d1n8yc4.fasta

./group363/reference/d1m6ba3-d1nqla3.fasta

./group363/reference/d1m6ba4-d1n8yc4.fasta

./group363/reference/d1n8yc4-d1nqla4.fasta

./group365/reference/d1b9wa1-d1ob1c1.fasta

./group365/reference/d1b9wa2-d1rfnb_.fasta

./group365/reference/d1b9wa2-d1xdtr_.fasta

./group365/reference/d1cvua2-d1hae__.fasta

./group365/reference/d1cvua2-d1l3ya_.fasta

./group365/reference/d1cvua2-d1m1xb5.fasta

./group365/reference/d1emo_1-d1hz8a2.fasta

./group365/reference/d1emo_1-d1lmja1.fasta

./group365/reference/d1hae__-d1xdtr_.fasta

./group365/reference/d1hz8a1-d1hz8a2.fasta

./group365/reference/d1hz8a2-d1klo_2.fasta

./group365/reference/d1hz8a2-d1lmja1.fasta

./group365/reference/d1ijqa2-d1tpg_1.fasta

./group365/reference/d1ioxa_-d1jl9a_.fasta

./group365/reference/d1ioxa_-d1m1xb5.fasta

./group365/reference/d1ioxa_-d1xdtr_.fasta

./group365/reference/d1jl9a_-d1xdtr_.fasta

./group365/reference/d1jl9a_-d3tgf__.fasta

./group365/reference/d1klo_1-d1klo_2.fasta

./group365/reference/d1klo_2-d1l3ya_.fasta

./group365/reference/d1l3ya_-d1m1xb5.fasta

./group365/reference/d1l3ya_-d1xdtr_.fasta

./group365/reference/d1lmja1-d1m1xb5.fasta

./group365/reference/d1lmja1-d1tpg_1.fasta

./group365/reference/d1tpg_1-d1urk_1.fasta

./group365/reference/d1xdtr_-d3tgf__.fasta

./group366/reference/d1bx7__-d1skz_2.fasta

./group367/reference/d1fas__-d1hc9a_.fasta

./group367/reference/d1fas__-d1jgka_.fasta

./group367/reference/d1ff4a_-d1hc9a_.fasta

./group367/reference/d1hc9a_-d1kbaa_.fasta

./group367/reference/d1hc9a_-d1tgxa_.fasta

./group367/reference/d1jgka_-d1kbaa_.fasta

./group367/reference/d1jgka_-d1tgxa_.fasta

./group367/reference/d1tfs__-d3ebx__.fasta

./group368/reference/d1aapa_-d1bik_1.fasta

./group368/reference/d1aapa_-d1bik_2.fasta

./group368/reference/d1aapa_-d1g6xa_.fasta

./group368/reference/d1aapa_-d1irha_.fasta

./group368/reference/d1aapa_-d1jc6a_.fasta

./group368/reference/d1aapa_-d1ktha_.fasta

./group368/reference/d1aapa_-d1tfxc_.fasta

./group368/reference/d1bik_1-d1bik_2.fasta

./group368/reference/d1bik_1-d1g6xa_.fasta

./group368/reference/d1bik_2-d1g6xa_.fasta

./group368/reference/d1bik_2-d1irha_.fasta

./group368/reference/d1g6xa_-d1irha_.fasta

./group368/reference/d1g6xa_-d1jc6a_.fasta

./group368/reference/d1irha_-d1jc6a_.fasta

./group368/reference/d1irha_-d1ktha_.fasta

./group368/reference/d1irha_-d1tfxc_.fasta

./group368/reference/d1jc6a_-d1tfxc_.fasta

./group368/reference/d1ktha_-d1tfxc_.fasta

./group369/reference/d1b8wa_-d1d6ba_.fasta

./group369/reference/d1b8wa_-d1ijva_.fasta

./group369/reference/d1bnb__-d1e4ta_.fasta

./group369/reference/d1bnb__-d1fd3a_.fasta

./group369/reference/d1bnb__-d1kj6a_.fasta

./group369/reference/d1d6ba_-d1kj6a_.fasta

./group369/reference/d1e4ta_-d1h5oa_.fasta

./group369/reference/d1fd3a_-d1ijva_.fasta

./group371/reference/d1ajj__-d1d2la_.fasta

./group371/reference/d1ajj__-d1f5ya1.fasta

./group371/reference/d1ajj__-d1f5ya2.fasta

./group371/reference/d1ajj__-d1k7ba_.fasta

./group371/reference/d1ajj__-d1n7da7.fasta

./group371/reference/d1ajj__-d1n7daa.fasta

./group371/reference/d1d2ja_-d1d2la_.fasta

./group371/reference/d1d2ja_-d1j8ea_.fasta

./group371/reference/d1d2ja_-d1k7ba_.fasta

./group371/reference/d1d2ja_-d1n7da7.fasta

./group371/reference/d1d2la_-d1f5ya1.fasta

./group371/reference/d1d2la_-d1f5ya2.fasta

./group371/reference/d1d2la_-d1k7ba_.fasta

./group371/reference/d1f5ya1-d1f5ya2.fasta

./group371/reference/d1f5ya1-d1k7ba_.fasta

./group371/reference/d1f5ya1-d1n7da7.fasta

./group371/reference/d1f5ya2-d1j8ea_.fasta

./group371/reference/d1f5ya2-d1n7da7.fasta

./group371/reference/d1j8ea_-d1k7ba_.fasta

./group371/reference/d1j8ea_-d1n7da7.fasta

./group371/reference/d1k7ba_-d1n7daa.fasta

./group372/reference/d1bhp__-d1nbla_.fasta

./group373/reference/d1bhta2-d1i71a_.fasta

./group373/reference/d1bhta2-d1pmla_.fasta

./group373/reference/d1bhta2-d5hpga_.fasta

./group373/reference/d1h8pa1-d1i71a_.fasta

./group373/reference/d1h8pa1-d1l6ja3.fasta

./group373/reference/d1h8pa2-d1i71a_.fasta

./group373/reference/d1h8pa2-d1ki0a3.fasta

./group373/reference/d1h8pa2-d1l6ja3.fasta

./group373/reference/d1h8pa2-d1l6ja5.fasta

./group373/reference/d1i71a_-d1ki0a2.fasta

./group373/reference/d1i71a_-d1pmla_.fasta

./group373/reference/d1kdu__-d1pmla_.fasta

./group373/reference/d1kdu__-d5hpga_.fasta

./group373/reference/d1ki0a2-d1pmla_.fasta

./group373/reference/d1ki0a3-d1pmla_.fasta

./group373/reference/d1pmla_-d2hpqp_.fasta

./group374/reference/d1ldtl_-d1lr7a2.fasta

./group374/reference/d1ldtl_-d1pce__.fasta

./group374/reference/d1ldtl_-d1tbrr1.fasta

./group374/reference/d1ldtl_-d1tgsi_.fasta

./group374/reference/d1lr7a2-d1pce__.fasta

./group374/reference/d1lr7a2-d1sgpi_.fasta

./group374/reference/d1lr7a2-d1tbrr1.fasta

./group374/reference/d1lr7a2-d1tbrr2.fasta

./group374/reference/d1lr7a2-d1tgsi_.fasta

./group374/reference/d1pce__-d1tbrr1.fasta

./group374/reference/d1pce__-d1tbrr2.fasta

./group374/reference/d1sgpi_-d1tbrr2.fasta

./group374/reference/d1tbrr1-d1tbrr2.fasta

./group374/reference/d1tbrr1-d1tgsi_.fasta

./group374/reference/d1tbrr2-d1tgsi_.fasta

./group375/reference/d1e9ta_-d2pspa2.fasta

./group375/reference/d1hi7a_-d2pspa2.fasta

./group376/reference/d1fl7b_-d1hcnb_.fasta

./group377/reference/d1ckla2-d1g40a2.fasta

./group377/reference/d1g40a2-d1g40a3.fasta

./group377/reference/d1g40a2-d1gkna2.fasta

./group377/reference/d1g40a2-d1h03p1.fasta

./group377/reference/d1g40a2-d1quba1.fasta

./group377/reference/d1g40a3-d1hcc__.fasta

./group377/reference/d1gkna1-d1nwva1.fasta

./group377/reference/d1h03p1-d1quba2.fasta

./group377/reference/d1ly2a2-d1quba5.fasta

./group379/reference/d1atb__-d1ccva_.fasta

./group379/reference/d1atb__-d1eaic_.fasta

./group379/reference/d1ccva_-d1coua_.fasta

./group380/reference/d1d4va2-d1exta2.fasta

./group381/reference/d1e88a3-d1fbr_1.fasta

./group382/reference/d1hlqa_-d1hpi__.fasta

./group382/reference/d1hpi__-d1iuaa_.fasta

./group382/reference/d1hpi__-d2hipa_.fasta

./group383/reference/d1bhi__-d1tf3a2.fasta

./group383/reference/d1bhi__-d1ubdc3.fasta

./group383/reference/d1ncs__-d1ubdc3.fasta

./group383/reference/d1paa__-d1zfd__.fasta

./group383/reference/d1tf3a2-d1ubdc3.fasta

./group383/reference/d1tf3a2-d1zfd__.fasta

./group383/reference/d1ubdc3-d2glia4.fasta

./group383/reference/d1zfd__-d2glia4.fasta

./group384/reference/d1d66a1-d1hwtc1.fasta

./group384/reference/d1d66a1-d1pyia1.fasta

./group385/reference/d1a7i_2-d1ibia2.fasta

./group385/reference/d1d4ua2-d1fjgn_.fasta

./group385/reference/d1d4ua2-d1lv3a_.fasta

./group385/reference/d1dsza_-d1kb2a_.fasta

./group385/reference/d1g47a2-d1nypa2.fasta

./group385/reference/d1gnf__-d3gata_.fasta

./group385/reference/d1gnf__-d7gata_.fasta

./group385/reference/d1ibia2-d1iml_2.fasta

./group385/reference/d1ibia2-d1nypa2.fasta

./group385/reference/d1iml_2-d1nypa2.fasta

./group385/reference/d1j2oa1-d1m3va1.fasta

./group385/reference/d1kb2a_-d1lata_.fasta

./group385/reference/d1kb2a_-d1lo1a_.fasta

./group385/reference/d1kb2a_-d2nllb_.fasta

./group385/reference/d1lv3a_-d1nypa1.fasta

./group386/reference/d1a6bb_-d1eska_.fasta

./group387/reference/d1zaka2-d1zin_2.fasta

./group389/reference/d1h7va_-d1lkoa2.fasta

./group389/reference/d1lkoa2-d1rb9__.fasta

./group391/reference/d1bor__-d1fbva4.fasta

./group392/reference/d1fmya_-d1jjda_.fasta

./group394/reference/d1dvpa2-d1joca1.fasta

./group394/reference/d1dvpa2-d1vfya_.fasta

./group394/reference/d1f62a_-d1fp0a1.fasta

./group394/reference/d1f62a_-d1mm2a_.fasta

./group394/reference/d1joca1-d1vfya_.fasta

./group395/reference/d1e31a_-d1g73c_.fasta

./group395/reference/d1e31a_-d1qbha_.fasta

./group395/reference/d1g73c_-d1i3oe_.fasta

./group395/reference/d1g73c_-d1qbha_.fasta

./group395/reference/d1i3oe_-d1jd5a_.fasta

./group395/reference/d1i3oe_-d1qbha_.fasta

./group395/reference/d1jd5a_-d1qbha_.fasta

./group396/reference/d1i8da1-d1kzla1.fasta

./group397/reference/d1hr6b1-d1l0la1.fasta

./group399/reference/d1i2ta_-d1jgna_.fasta

./group400/reference/d1oi1a1-d1oi1a2.fasta

./group416/reference/d1ee8a2-d1k82a2.fasta

./group416/reference/d1l1za2-d1nnja2.fasta

./group417/reference/d1m9sa2-d1m9sa4.fasta

./group419/reference/d1aym1_-d1bev1_.fasta

./group419/reference/d1aym1_-d1d4m1_.fasta

./group419/reference/d1aym1_-d1k5ma_.fasta

./group419/reference/d1aym1_-d1pvc1_.fasta

./group419/reference/d1aym3_-d1bev3_.fasta

./group419/reference/d1bev1_-d1d4m1_.fasta

./group419/reference/d1bev1_-d1k5ma_.fasta

./group419/reference/d1bev1_-d1pvc1_.fasta

./group419/reference/d1bev3_-d1pvc3_.fasta

./group419/reference/d1bmv11-d1ny711.fasta

./group419/reference/d1d4m1_-d1k5ma_.fasta

./group420/reference/d1gff2_-d2bpa2_.fasta
