# Supplementary material for: PR2ALIGN: a stand-alone software program and a web-server for protein sequence alignment using weighted biochemical properties of amino acids
Source: BMC Res Notes. 2015 May 7;8:187. doi: 10.1186/s13104-015-1152-6 (PMC4477417; doi:10.1186/s13104-015-1152-6)

**Supplementary Figure 1.** **A comparison of pair-wise sequence alignments between SABmark domains d1k61a and d2ezl.**

**(A)**The SABmark reference alignment obtained using structural superposition. Percentage sequence identity: 8.3%.
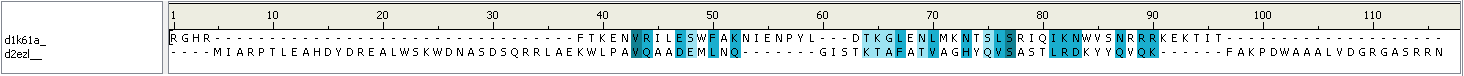


**(B)** The alignment obtained using PR2ALIGN with the four default amino acid properties and parameters optimized for 0-10% sequence identity range. The percentage of correctly recovered alignment positions (Eq.8): 49.9%.


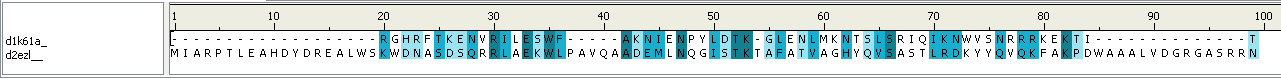


**(C)** The alignment obtained using the VTML200 amino acid similarity matrix and parameters optimized for 0-10% sequence identity range. The percentage of correctly recovered alignment positions (Eq.8): 0%.


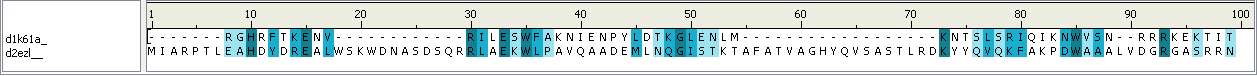


**Supplementary Figure 2.** **A comparison of pair-wise sequence alignments between SABmark domains d1jkza_ and d1mm0a_.**

**(A)**The SABmark reference alignment obtained using structural superposition. Percentage sequence identity: 19.4%.
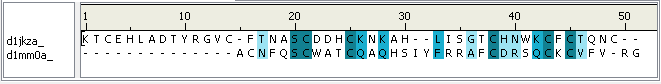


**(B)** The alignment obtained using PR2ALIGN with the four default amino acid properties and parameters optimized for 10-20% sequence identity range. The percentage of correctly recovered alignment positions (Eq.8): 80.7%.


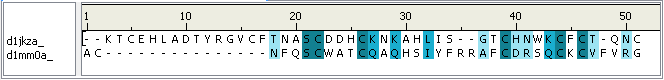


**(C)** The alignment obtained using the VTML200 amino acid similarity matrix and parameters optimized for 10-20% sequence identity range. The percentage of correctly recovered alignment positions (Eq.8): 0%.


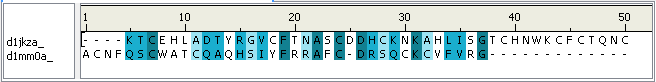


**Supplementary Figure 3.** **A comparison of pair-wise sequence alignments between SABmark domains d1dsva and d1eska.**

**(A)**The SABmark reference alignment obtained using structural superposition. Percentage sequence identity: 29.4%.


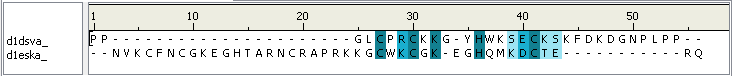


**(B)** The alignment obtained using PR2ALIGN with the four default amino acid properties and parameters optimized for 20-30% sequence identity range. The percentage of correctly recovered alignment positions (Eq.8): 85.8%.


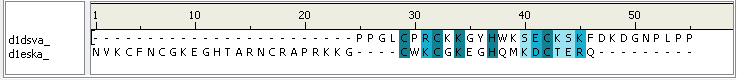


**(C)** The alignment obtained using the VTML200 amino acid similarity matrix and parameters optimized for 20-30% sequence identity range. The percentage of correctly recovered alignment positions (Eq.8): 0%.


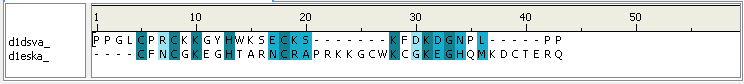


**Supplementary Figure 4.** **A comparison of pair-wise sequence alignments between SABmark domains d1myn and d1scy.**

**(A)**The SABmark reference alignment obtained using structural superposition. Percentage sequence identity: 31.8%.


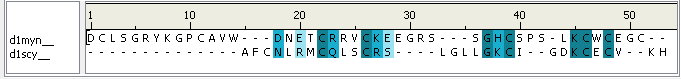


**(B)** The alignment obtained using PR2ALIGN with the four default amino acid properties and parameters optimized for 30-40% sequence identity range. The percentage of correctly recovered alignment positions (Eq.8): 85.2%.


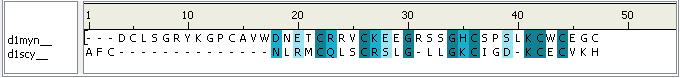


**(C)** The alignment obtained using the VTML200 amino acid similarity matrix and parameters optimized for 30-40% sequence identity range. The percentage of correctly recovered alignment positions (Eq.8): 20.0%.


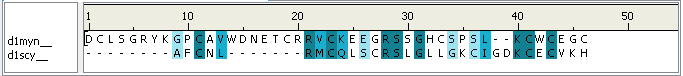


**Supplementary Figure 5.** **A comparison of pair-wise sequence alignments between SABmark domains d1a6bb and d1eska.**

**(A)**The SABmark reference alignment obtained using structural superposition. Percentage sequence identity: 41.2%.


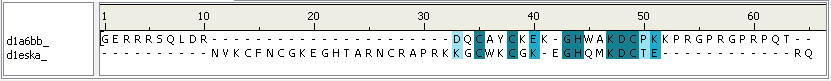


**(B)** The alignment obtained using PR2ALIGN with the four default amino acid properties and parameters optimized for 40-50% sequence identity range. The percentage of correctly recovered alignment positions (Eq.8): 73.7%.


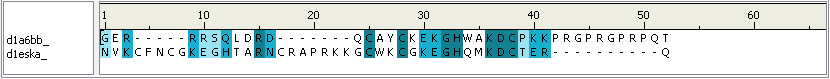


**(C)** The alignment obtained using the VTML200 amino acid similarity matrix and parameters optimized for 40-50% sequence identity range. The percentage of correctly recovered alignment positions (Eq.8): 0%.


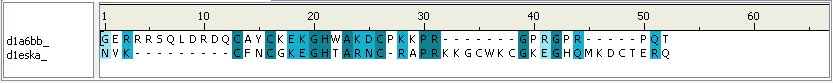

Supplement: Additional file 11: — Supplementary Figures S1-S5. [file 13104_2015_1152_MOESM11_ESM.docx]
